# Proceedings of the 3rd IPLeiria’s International Health Congress: Leiria, Portugal. 6-7 May 2016

**DOI:** 10.1186/s12913-016-1423-5

**Published:** 2016-07-06

**Authors:** Catarina Cardoso Tomás, Emanuel Oliveira, D. Sousa, M. Uba-Chupel, G. Furtado, C. Rocha, A. Teixeira, P. Ferreira, Celeste Alves, Stefan Gisin, Elisabete Catarino, Nelma Carvalho, Tiago Coucelo, Luís Bonfim, Carina Silva, Débora Franco, Jesús Alcoba González, Helena G. Jardim, Rita Silva, Cristina L. Baixinho, Mª Helena Presado, Mª Fátima Marques, Mário E. Cardoso, Marina Cunha, Joana Mendes, Ana Xavier, Ana Galhardo, Margarida Couto, João G. Frade, Carla Nunes, João R. Mesquita, Maria S. Nascimento, Guilherme Gonçalves, Conceição Castro, Alice Mártires, Mª João Monteiro, Conceição Rainho, Francisco P. Caballero, Fatima M. Monago, Jose T. Guerrero, Rocio M. Monago, Africa P. Trigo, Milagros L. Gutierrez, Gemma M. Milanés, Mercedes G. Reina, Ana G. Villanueva, Ana S. Piñero, Isabel R. Aliseda, Francisco B. Ramirez, Andrea Ribeiro, Ana Quelhas, Conceição Manso, Francisco P. Caballero, Jose T. Guerrero, Fatima M. Monago, Rafael B. Santos, Nuria R. Jimenez, Cristina G. Nuñez, Inmaculada R. Gomez, Mª Jose L. Fernandez, Laura A. Marquez, Ana L. Moreno, Mª Jesus Tena Huertas, Francisco B. Ramirez, Daniel Seabra, Mª Céu Salvador, Luciene Braga, Pedro Parreira, Anabela Salgueiro-Oliveira, Cristina Arreguy-Sena, Bibiana F. Oliveira, Mª Adriana Henriques, Joana Santos, Sara Lebre, Alda Marques, Clarinda Festas, Sandra Rodrigues, Andrea Ribeiro, José Lumini, Ana G. Figueiredo, Francisco J. Hernandez-Martinez, Liliana Campi, Mª Pino Quintana-Montesdeoca, Juan F. Jimenez-Diaz, Bienvenida C. Rodriguez-De-Vera, Alexandra Parente, Mª Augusta Mata, Ana Mª Pereira, Adília Fernandes, Manuel Brás, Mª Rosário Pinto, Pedro Parreira, Marta L. Basto, Ana C. Rei, Lisete M. Mónico, Gilberta Sousa, Clementina Morna, Otília Freitas, Gregório Freitas, Ana Jardim, Rita Vasconcelos, Lina G. Horta, Roger S. Rosa, Luís F. Kranz, Rita C. Nugem, Mariana S. Siqueira, Ronaldo Bordin, Rosiane Kniess, Josimari T. Lacerda, Joana Guedes, Idalina Machado, Sidalina Almeida, Adriano Zilhão, Helder Alves, Óscar Ribeiro, Ana P. Amaral, Ana Santos, Joana Monteiro, Mª Clara Rocha, Rui Cruz, Ana P. Amaral, Marina Lourenço, Mª Clara Rocha, Rui Cruz, Sandra Antunes, Verónica Mendonça, Isabel Andrade, Nádia Osório, Ana Valado, Armando Caseiro, António Gabriel, Anabela C. Martins, Fernando Mendes, Lídia Cabral, Manuela Ferreira, Amadeu Gonçalves, Tatiana D. Luz, Leonardo Luz, Raul Martins, Alice Morgado, Maria L. Vale-Dias, Rui Porta-Nova, Tânia C. Fleig, Éboni M. Reuter, Miriam B. Froemming, Sabrina L. Guerreiro, Lisiane L. Carvalho, Daniel Guedelha, P. Coelho, A. Pereira, António Calha, Raul Cordeiro, Ana Gonçalves, Ana Certo, Ana Galvão, Mª Augusta Mata, Aline Welter, Elayne Pereira, Sandra Ribeiro, Marcia Kretzer, Juan-Fernando Jiménez-Díaz, Carla Jiménez-Rodríguez, Francisco-José Hernández-Martínez, Bienvenida-Del-Carmen Rodríguez-De-Vera, Alexandre Marques-Rodrigues, Patrícia Coelho, Tiago Bernardes, Alexandre Pereira, Patrícia Sousa, João G. Filho, Nazare Nazario, Marcia Kretzer, Odete Amaral, António Garrido, Nélio Veiga, Carla Nunes, Ana R. Pedro, Carlos Pereira, António Almeia, Helder M. Fernandes, Carlos Vasconcelos, Nelson Sousa, Victor M. Reis, M. João Monteiro, Romeu Mendes, Isabel C. Pinto, Tânia Pires, João Gama, Vera Preto, Norberto Silva, Carlos Magalhães, Matilde Martins, Mafalda Duarte, Constança Paúl, Ignácio Martín, Arminda A. Pinheiro, Sandra Xavier, Julieta Azevedo, Elisabete Bento, Cristiana Marques, Mariana Marques, António Macedo, Ana T. Pereira, José P. Almeida, António Almeida, Josiane Alves, Nelson Sousa, Francisco Saavedra, Romeu Mendes, Ana S. Maia, Michelle T. Oliveira, Anderson R. Sousa, Paulo P. Ferreira, Luci S. Lopes, Eujcely C. Santiago, Sílvia Monteiro, Ângelo Jesus, Armanda Colaço, António Carvalho, Rita P. Silva, Agostinho Cruz, Ana Ferreira, Catarina Marques, João P. Figueiredo, Susana Paixão, Ana Ferreira, Carla Lopes, Fernando Moreira, João P. Figueiredo, Ana Ferreira, Diana Ribeiro, Fernando Moreira, João P. Figueiredo, Susana Paixão, Telma Fernandes, Diogo Amado, Jéssica Leal, Marcelo Azevedo, Sónia Ramalho, Catarina Mangas, Jaime Ribeiro, Rita Gonçalves, Amélia F Nunes, Ana R. Tuna, Carlos R. Martins, Henriqueta D. Forte, Cláudia Costa, José A. Tenedório, Paula Santana, J. A. Andrade, J. L. Pinto, C. Campofiorito, S. Nunes, A. Carmo, A. Kaliniczenco, B. Alves, F. Mendes, C. Jesus, F. Fonseca, F. Gehrke, Carlos Albuquerque, Rita Batista, Madalena Cunha, António Madureira, Olivério Ribeiro, Rosa Martins, Teresa Madeira, Catarina Peixoto-Plácido, Nuno Santos, Osvaldo Santos, Astrid Bergland, Asta Bye, Carla Lopes, Violeta Alarcão, Beatriz Goulão, Nuno Mendonça, Paulo Nicola, João G. Clara, João Gomes, Ana Querido, Catarina Tomás, Daniel Carvalho, Marina Cordeiro, Marlene C. Rosa, Alda Marques, Daniela Brandão, Óscar Ribeiro, Lia Araújo, Constança Paúl, Beatriz Minghelli, Sylvina Richaud, Ana L. Mendes, Joana Marta-Simões, Inês A. Trindade, Cláudia Ferreira, Teresa Carvalho, Marina Cunha, José Pinto-Gouveia, Morgana C. Fernandes, Roger S. Rosa, Rita C. Nugem, Luís F. Kranz, Mariana S. Siqueira, Ronaldo Bordin, Anabela C. Martins, Anabela Medeiros, Rafaela Pimentel, Andreia Fernandes, Carlos Mendonça, Isabel Andrade, Susana Andrade, Ruth L. Menezes, Rafael Bravo, Marta Miranda, Lierni Ugartemendia, José Mª Tena, Francisco L. Pérez-Caballero, Lorena Fuentes-Broto, Ana B. Rodríguez, Barriga Carmen, M. A. Carneiro, J. N. Domingues, S. Paixão, J. Figueiredo, V. B. Nascimento, C. Jesus, F Mendes, F. Gehrke, B. Alves, L. Azzalis, F. Fonseca, Ana R. Martins, Amélia Nunes, Arminda Jorge, Nélio Veiga, Ana Amorim, André Silva, Liliana Martinho, Luís Monteiro, Rafael Silva, Carina Coelho, Odete Amaral, Inês Coelho, Carlos Pereira, André Correia, Diana Rodrigues, Nídia Marante, Pedro Silva, Sara Carvalho, André Rts Araujo, Maximiano Ribeiro, Paula Coutinho, Sandra Ventura, Fátima Roque, Cristina Calvo, Manoela Reses, Jorge Conde, Ana Ferreira, João Figueiredo, David Silva, Luís Seiça, Raquel Soares, Ricardo Mourão, Teresa Kraus, Ana C. Abreu, José M. Padilha, Júlia M. Alves, Paulino Sousa, Manuel Oliveira, Joana Sousa, Sónia Novais, Felismina Mendes, Joana Pinto, Joana Cruz, Alda Marques, Hugo Duarte, Maria Dos Anjos Dixe, Pedro Sousa, Inês Cruz, Fernanda Bastos, Filipe Pereira, Francisco L. Carvalho, Teresa T. Oliveira, Vítor R. Raposo, Conceição Rainho, José C. Ribeiro, Isabel Barroso, Vítor Rodrigues, Carmo Neves, Teresa C. Oliveira, Bárbara Oliveira, Mª Carminda Morais, Pilar Baylina, Rogério Rodrigues, Zaida Azeredo, Corália Vicente, Hélia Dias, Margarida Sim-Sim, Pedro Parreira, Anabela Salgueiro-Oliveira, Amélia Castilho, Rosa Melo, João Graveto, José Gomes, Marina Vaquinhas, Carla Carvalho, Lisete Mónico, Nuno Brito, Cassilda Sarroeira, José Amendoeira, Fátima Cunha, Anabela Cândido, Patrícia Fernandes, Helena R. Silva, Elsa Silva, Isabel Barroso, Leila Lapa, Cristina Antunes, Ana Gonçalves, Ana Galvão, Mª José Gomes, Susana R. Escanciano, Maria Freitas, Pedro Parreira, João Marôco, Ana R. Fernandes, Cremilde Cabral, Samuel Alves, Pedro Sousa, António Ferreira, Fernanda Príncipe, Ulla-Maija Seppänen, Margarida Ferreira, Maribel Carvalhais, Marilene Silva, Manuela Ferreira, Joana Silva, Jéssica Neves, Diana Costa, Bruno Santos, Soraia Duarte, Sílvia Marques, Sónia Ramalho, Isabel Mendes, Clarisse Louro, Eva Menino, Maria Dixe, Sara S. Dias, Marina Cordeiro, Catarina Tomás, Ana Querido, Daniel Carvalho, João Gomes, Frederico C. Valim, Joyce O. Costa, Lúcia G. Bernardes, Helena Prebianchi, Marlene Cristina Rosa, Narcisa Gonçalves, Maria M. Martins, Paulina Kurcgant, André Vieira, Sandrina Bento, Sérgio Deodato, Isabel Rabiais, Laura Reis, Ana Torres, Sérgio Soares, Margarida Ferreira, Pedro Graça, Céu Leitão, Renato Abreu, Fernando Bellém, Ana Almeida, Edna Ribeiro-Varandas, Ana Tavares, João G. Frade, Carolina Henriques, Eva Menino, Clarisse Louro, Célia Jordão, Sofia Neco, Carminda Morais, Pedro Ferreira, Carla R. Silva, Alice Brito, Antónia Silva, Hugo Duarte, Maria Dos Anjos Dixe, Pedro Sousa, Gabriela Postolache, Raul Oliveira, Isabel Moreira, Luísa Pedro, Sónia Vicente, Samuel Domingos, Octavian Postolache, Darlen Silva, João G. Filho, Nazare Nazario, Marcia Kretzer, Dulcineia Schneider, Fátima M. Marques, Pedro Parreira, Carla Carvalho, Lisete M. Mónico, Carlos Pinto, Sara Vicente, São João Breda, José H. Gomes, Rosa Melo, Pedro Parreira, Anabela Salgueiro, João Graveto, Marina Vaquinhas, Amélia Castilho, Ângelo Jesus, Nuno Duarte, José C. Lopes, Hélder Nunes, Agostinho Cruz, Anabela Salgueiro-Oliveira, Pedro Parreira, Marta L. Basto, Luciene M. Braga, António Ferreira, Beatriz Araújo, José M. Alves, Margarida Ferreira, Maribel Carvalhais, Marilene Silva, Sónia Novais, Ana S. Sousa, Cândida Ferrito, Pedro L. Ferreira, Alexandre Rodrigues, Margarida Ferreira, Isabel Oliveira, Manuela Ferreira, Jéssica Neves, Diana Costa, Soraia Duarte, Joana Silva, Bruno Santos, Cristina Martins, Ana P. Macedo, Odete Araújo, Cláudia Augusto, Fátima Braga, Lisa Gomes, Maria A. Silva, Rafaela Rosário, Luís Pimenta, Diana Carreira, Patrícia Teles, Teresa Barros, Catarina Tomás, Ana Querido, Daniel Carvalho, João Gomes, Marina Cordeiro, Daniel Carvalho, Ana Querido, Catarina Tomás, João Gomes, Marina Cordeiro, Cristina Jácome, Alda Marques, Sylvie Capelas, Andreia Hall, Dina Alves, Marisa Lousada, Mª Helena Loureiro, Ana Camarneiro, Margarida Silva, Aida Mendes, Ana Pedreiro, Anne G.Silva, Elza S. Coelho, Flávio Melo, Fernando Ribeiro, Rui Torres, Rui Costa, Tânia Pinho, Cristina Jácome, Alda Marques, Bárbara Cruz, Daniel Seabra, Diogo Carreiras, Maria Ventura, x Cruz, Dina Brooks, Alda Marques, M Rosário Pinto, Pedro Parreira, Marta Lima-Basto, Miguel Neves, Lisete M. Mónico, Carla Bizarro, Marina Cunha, Ana Galhardo, Couto Margarida, Ana P. Amorim, Eduardo Silva, Susana Cruz, José M. Padilha, Jorge Valente, José T. Guerrero, Francisco P. Caballero, Rafael B. Santos, Estefania P. Gonzalez, Fátima M. Monago, Lierni U. Ugalde, Marta M. Vélez, Maria J. Tena, José T. Guerrero, Rafael Bravo, Francisco L. Pérez-Caballero, Isabel A. Becerra, Mª Elizabeth Agudelo, Guadalupe Acedo, Roberto Bajo, Isabel Malheiro, Filomena Gaspar, Luísa Barros, Guilherme Furtado, Mateus Uba-Chupel, Mariana Marques, Luís Rama, Margarida Braga, José P. Ferreira, Ana Mª Teixeira, João Cruz, Tiago Barbosa, Ângela Simões, Luís Coelho, Alexandre Rodrigues, Juan-Fernando Jiménez-Díaz, Francisco Martinez-Hernandez, Bienvenida Rodriguez-De-Vera, Pedro Ferreira, Alexandrina Rodrigues, André Ramalho, João Petrica, Pedro Mendes, João Serrano, Inês Santo, António Rosado, Paula Mendonça, Kátia Freitas, Dora Ferreira, António Brito, Renato Fernandes, Sofia Gomes, Fernando Moreira, Cláudia Pinho, Rita Oliveira, Ana I. Oliveira, Paula Mendonça, Ana P. Casimiro, Patrícia Martins, Iryna Silva, Diana Evangelista, Catarina Leitão, Fábia Velosa, Nélio Carecho, Luís Coelho, Eva Menino, Anjos Dixe, Helena Catarino, Fátima Soares, Ester Gama, Clementina Gordo, Eliana Moreira, Cristiana Midões, Marlene Santos, Sara Machado, Vânia P. Oliveira, Marlene Santos, Ana Querido, Anjos Dixe, Rita Marques, Zaida Charepe, Ana Antunes, Sofia Santos, Marlene C. Rosa, Marlene C. Rosa, Silvana F. Marques, Beatriz Minghelli, Eulália CaroMinghelli, Mª José Luís, Teresa Brandão, Pedro Mendes, Daniel Marinho, João Petrica, Diogo Monteiro, Rui Paulo, João Serrano, Inês Santo, Lina Monteiro, Fátima Ramalho, Rita Santos-Rocha, Sónia Morgado, Teresa Bento, Gilberta Sousa, Otília Freitas, Isabel Silva, Gregório Freitas, Clementina Morna, Rita Vasconcelos, Tatiana Azevedo, Salete Soares, Jacinta Pisco, Paulo P. Ferreira, Efrain O. Olszewer, Michelle T. Oliveira, Anderson R. Sousa, Ana S. Maia, Sebastião T. Oliveira, Erica Santos, Ana I. Oliveira, Carla Maia, Fernando Moreira, Joana Santos, Maria F. Mendes, Rita F. Oliveira, Cláudia Pinho, Eduarda Barreira, Ana Pereira, Josiana A. Vaz, André Novo, Luís D. Silva, Bruno Maia, Eduardo Ferreira, Filipa Pires, Renato Andrade, Luís Camarinha, Luís D. Silva, Bruno Maia, Eduardo Ferreira, Filipa Pires, Renato Andrade, Luís Camarinha, Ana F. César, Mariana Poço, David Ventura, Raquel Loura, Pedro Gomes, Catarina Gomes, Cláudia Silva, Elsa Melo, João Lindo, Joana Domingos, Zaida Mendes, Susana Poeta, Tiago Carvalho, Catarina Tomás, Helena Catarino, Mª Anjos Dixe, André Ramalho, António Rosado, Pedro Mendes, Rui Paulo, Inês Garcia, João Petrica, Sandra Rodrigues, Rui Meneses, Carlos Afonso, Luís Faria, Adérito Seixas, Marina Cordeiro, Paulo Granjo, José C. Gomes, Nelba R. Souza, Guilherme E. Furtado, Saulo V. Rocha, Paula Silva, Joana Carvalho, Marina Ana Morais, Sofia Santos, Paula Lebre, Ana Antunes, António Calha, Ana Xavier, Marina Cunha, José Pinto-Gouveia, Liana Alencar, Madalena Cunha, António Madureira, Ilda Cardoso, Ana Galhardo, Fernanda Daniel, Vítor Rodrigues, Leonardo Luz, Tatiana Luz, Maurício R. Ramos, Dayse C. Medeiros, Bruno M. Carmo, André Seabra, Cristina Padez, Manuel C. Silva, António Rodrigues, Patrícia Coelho, Alexandre Coelho, Madson Caminha, Filipe Matheus, Elenice Mendes, Jony Correia, Marcia Kretzer, Francisco J. Hernandez-Martinez, Juan F. Jimenez-Diaz, Bienvendida C. Rodriguez-De-Vera, Carla Jimenez-Rodriguez, Yadira Armas-Gonzalez, Cátia Rodrigues, Rosa Pedroso, Jennifer Apolinário-Hagen, Viktor Vehreschild, Milene Veloso, Celina Magalhães, Isabel Cabral, Maira Ferraz, Filipe Nave, Emília Costa, Filomena Matos, José Pacheco, António Dias, Carlos Pereira, João Duarte, Madalena Cunha, Daniel Silva, Lisete M. Mónico, Valentim R. Alferes, Mª São João Brêda, Carla Carvalho, Pedro M. Parreira, Mª Carminda Morais, Pedro Ferreira, Rui Pimenta, José Boavida, Isabel C. Pinto, Tânia Pires, Catarina Silva, Maria Ribeiro, Maria Viega-Branco, Filomena Pereira, Ana Mª Pereira, Fabrícia M. Almeida, Gustavo L. Estevez, Sandra Ribeiro, Marcia R. Kretzer, Paulo V. João, Paulo Nogueira, Sandra Novais, Ana Pereira, Lara Carneiro, Maria Mota, Rui Cruz, Luiz Santiago, Carlos Fontes-Ribeiro, Guilherme Furtado, Saulo V. Rocha, André P. Coutinho, João S. Neto, Lélia R. Vasconcelos, Nelba R. Souza, Estélio Dantas, Alexandra Dinis, Sérgio Carvalho, Paula Castilho, José Pinto-Gouveia, Alexandra Sarreira-Santos, Amélia Figueiredo, Lurdes Medeiros-Garcia, Paulo Seabra, Rosa Rodrigues, Mª Carminda Morais, Paula O. Fernandes, Conceição Santiago, Mª Henriqueta Figueiredo, Marta L. Basto, Teresa Guimarães, André Coelho, Anabela Graça, Ana M. Silva, Ana R. Fonseca, Luz Vale-Dias, Bárbara Minas, Graciete Franco-Borges, Cristina Simões, Sofia Santos, Ana Serra, Maria Matos, Luís Jesus, Ana S. Tavares, Ana Almeida, Céu Leitão, Edna Varandas, Renato Abreu, Fernando Bellém, Inês A. Trindade, Cláudia Ferreira, José Pinto-Gouveia, Joana Marta-Simões, Odete Amaral, Cristiana Miranda, Pedro Guimarães, Rodrigo Gonçalves, Nélio Veiga, Carlos Pereira, Tânia C. Fleig, Elisabete A. San-Martin, Cássia L. Goulart, Paloma B. Schneiders, Natacha F. Miranda, Lisiane L. Carvalho, Andrea G. Silva, Joana Topa, Conceição Nogueira, Sofia Neves, Rita Ventura, Cristina Nazaré, Daniela Brandão, Alberto Freitas, Óscar Ribeiro, Constança Paúl, Cristiana Mercê, Marco Branco, Pedro Almeida, Daniela Nascimento, Juliana Pereira, David Catela, Helga Rafael, Alcinda C. Reis, Ana Mendes, Ana R. Valente, Marisa Lousada, Diana Sousa, Ana L. Baltazar, Mª Helena Loureiro, Ana Oliveira, José Aparício, Alda Marques, Alda Marques, Ana Oliveira, Joana Neves, Rodrigo Ayoub, Luís Sousa, Cristina Marques-Vieira, Sandy Severino, Helena José, Inês Cadorio, Marisa Lousada, Marina Cunha, Diogo Andrade, Ana Galhardo, Margarida Couto, Fernando Mendes, Cátia Domingues, Susann Schukg, Ana M. Abrantes, Ana C. Gonçalves, Tiago Sales, Ricardo Teixo, Rita Silva, Jéssica Estrela, Mafalda Laranjo, João Casalta-Lopes, Clara Rocha, Paulo C. Simões, Ana B. Sarmento-Ribeiro, Mª Filomena Botelho, Manuel S. Rosa, Virgínia Fonseca, Diogo Colaço, Vanessa Neves, Carlos Jesus, Camilla Hesse, Clara Rocha, Nádia Osório, Ana Valado, Armando Caseiro, António Gabriel, Lola Svensson, Fernando Mendes, Wafa A. Siba, Cristina Pereira, Jorge Tomaz, Teresa Carvalho, José Pinto-Gouveia, Marina Cunha, Diana Duarte, Nuno V. Lopes, Rui Fonseca-Pinto, Diana Duarte, Nuno V. Lopes, Rui Fonseca-Pinto, Anabela C. Martins, Piedade Brandão, Laura Martins, Margarida Cardoso, Nuno Morais, Joana Cruz, Nuno Alves, Paula Faria, Artur Mateus, Pedro Morouço, Nuno Alves, Nelson Ferreira, Artur Mateus, Paula Faria, Pedro Morouço, Isabel Malheiro, Filomena Gaspar, Luísa Barros, Pedro Parreira, Andreia Cardoso, Lisete Mónico, Carla Carvalho, Albino Lopes, Anabela Salgueiro-Oliveira, Adérito Seixas, Valter Soares, Tiago Dias, Ricardo Vardasca, Joaquim Gabriel, Sandra Rodrigues, Hugo Paredes, Arsénio Reis, Sara Marinho, Vítor Filipe, Jorge Lains, João Barroso, Carolina Da Motta, Célia B. Carvalho, José Pinto-Gouveia, Ermelindo Peixoto, Ana A. Gomes, Vanessa Costa, Diana Couto, Daniel R. Marques, José A. Leitão, José Tavares, Maria H. Azevedo, Carlos F. Silva, João Freitas, Pedro Parreira, João Marôco, Miguel A. Garcia-Gordillo, Daniel Collado-Mateo, Gang Chen, Angelo Iezzi, José A. Sala, José A. Parraça, Narcis Gusi, Jani Sousa, Mariana Marques, Jacinto Jardim, Anabela Pereira, Sónia Simões, Marina Cunha, Pedro Sardo, Jenifer Guedes, João Lindo, Paulo Machado, Elsa Melo, Célia B. Carvalho, Joana Benevides, Marina Sousa, Joana Cabral, Carolina Da Motta, Ana T. Pereira, Sandra Xavier, Julieta Azevedo, Elisabete Bento, Cristiana Marques, Rosa Carvalho, Mariana Marques, António Macedo, Ana M. Silva, Juliana Alves, Ana A. Gomes, Daniel R. Marques, Mª Helena Azevedo, Carlos Silva, Ana Mendes, Huei D. Lee, Newton Spolaôr, Jefferson T. Oliva, Wu F. Chung, Rui Fonseca-Pinto, Keila Bairros, Cláudia D. Silva, Clóvis A. Souza, Silvana S. Schroeder, Elsa Araújo, Helena Monteiro, Ricardo Costa, Sara S. Dias, Jorge Torgal, Carolina G. Henriques, Luísa Santos, Elisa F. Caceiro, Sónia A. Ramalho, Rita Oliveira, Vera Afreixo, João Santos, Priscilla Mota, Agostinho Cruz, Francisco Pimentel, Rita Marques, Mª Anjos Dixe, Ana Querido, Patrícia Sousa, Joana Benevides, Carolina Da Motta, Marina Sousa, Suzana N. Caldeira, Célia B. Carvalho, Ana Querido, Catarina Tomás, Daniel Carvalho, João Gomes, Marina Cordeiro, Joyce O. Costa, Frederico C. Valim, Lígia C. Ribeiro, Zaida Charepe, Ana Querido, Mª Henriqueta Figueiredo, Priscila S. Aquino, Samila G. Ribeiro, Ana B. Pinheiro, Paula A. Lessa, Mirna F. Oliveira, Luísa S. Brito, Ítalo N. Pinto, Alessandra S. Furtado, Régia B. Castro, Caroline Q. Aquino, Eveliny S. Martins, Ana B Pinheiro, Priscila S. Aquino, Lara L. Oliveira, Patrícia C. Pinheiro, Caroline R. Sousa, Vívien A. Freitas, Tatiane M. Silva, Adman S. Lima, Caroline Q. Aquino, Karizia V. Andrade, Camila A. Oliveira, Eglidia F. Vidal, Ana Ganho-Ávila, Mariana Moura-Ramos, Óscar Gonçalves, Jorge Almeida, Armando Silva, Irma Brito, João Amado, António Rodrigo, Sofia Santos, Fernando Gomes, Marlene C. Rosa, Silvana F. Marques, Sara Luís, Luís Cavalheiro, Pedro Ferreira, Rui Gonçalves, Rui S. Lopes, Luís Cavalheiro, Pedro Ferreira, Rui Gonçalves, Bruno H. Fiorin, Marina S. Santos, Edmar S. Oliveira, Rita L. Moreira, Elizabete A. Oliveira, Braulio L. Filho, Lara Palmeira, Teresa Garcia, José Pinto-Gouveia, Marina Cunha, Sara Cardoso, Lara Palmeira, Marina Cunha, José Pinto-Gouveia, Joana Marta-Simões, Ana L. Mendes, Inês A. Trindade, Sara Oliveira, Cláudia Ferreira, Ana L. Mendes, Joana Marta-Simões, Inês A. Trindade, Cláudia Ferreira, Filipe Nave, Mariana Campos, Iris Gaudêncio, Fernando Martins, Lino Ferreira, Nuno Lopes, Rui Fonseca-Pinto, Rogério Rodrigues, Zaida Azeredo, Corália Vicente, Joana Silva, Patrícia Sousa, Rita Marques, Isabel Mendes, Rogério Rodrigues, Zaida Azeredo, Corália Vicente, Ricardo Vardasca, Ana R. Marques, Adérito Seixas, Rui Carvalho, Joaquim Gabriel, Paulo P. Ferreira, Michelle T. Oliveira, Anderson R. Sousa, Ana S. Maia, Sebastião T. Oliveira, Pablo O. Costa, Maiza M. Silva, Cristina Arreguy-Sena, Nathália Alvarenga-Martins, Paulo F. Pinto, Denize C. Oliveira, Pedro D. Parreira, Antônio T. Gomes, Luciene M. Braga, Odete Araújo, Isabel Lage, José Cabrita, Laetitia Teixeira, Rita Marques, Mª Anjos Dixe, Ana Querido, Patrícia Sousa, Sara Silva, Eugénio Cordeiro, João Pimentel, Vera Ferro-Lebres, Juliana A. Souza, Mariline Tavares, Mª Anjos Dixe, Pedro Sousa, Rui Passadouro, Teresa Peralta, Carlos Ferreira, Georgina Lourenço, João Serrano, João Petrica, Rui Paulo, Samuel Honório, Pedro Mendes, Alexandra Simões, Lucinda Carvalho, Alexandre Pereira, Sara Silva, Paulino Sousa, José M. Padilha, Daniela Figueiredo, Carolina Valente, Alda Marques, Patrícia Ribas, Joana Sousa, Frederico Brandão, Cesar Sousa, Matilde Martins, Patrícia Sousa, Rita Marques, Francisco Mendes, Rosina Fernandes, Emília Martins, Cátia Magalhães, Patrícia Araújo, Carla Grande, Mª Augusta Mata, Juan G. Vieitez, Bruna Bianchini, Nazare Nazario, João G. Filho, Marcia Kretzer, Tânia Costa, Armando Almeida, Gabriel Baffour, Armando Almeida, Tânia Costa, Gabriel Baffour, Zaida Azeredo, Carlos Laranjeira, Magda Guerra, Ana P. Barbeiro, Regina Ferreira, Sara Lopes, Liliana Nunes, Ana Mendes, Julian Martins, Dulcineia Schneider, Marcia Kretzer, Flávio Magajewski, Célia Soares, António Marques, Marco Batista, Ruth J. Castuera, Helena Mesquita, António Faustino, Jorge Santos, Samuel Honório, Betina P. Vizzotto, Leticia Frigo, Hedioneia F. Pivetta, Dolores Sardo, Cristina Martins, Wilson Abreu, Mª Céu Figueiredo, Marco Batista, Ruth Jimenez-Castuera, João Petrica, João Serrano, Samuel Honório, Rui Paulo, Pedro Mendes, Patrícia Sousa, Rita Marques, António Faustino, Paulo Silveira, João Serrano, Rui Paulo, Pedro Mendes, Samuel Honório, Catarina Oliveira, Fernanda Bastos, Inês Cruz, Cláudia K. Rodriguez, Márcia R. Kretzer, Nazaré O. Nazário, Pedro Cruz, Daniela C. Vaz, Rui B. Ruben, Francisco Avelelas, Susana Silva, Mª Jorge Campos, Maria Almeida, Liliana Gonçalves, Lígia Antunes, Pedro Sardo, Jenifer Guedes, João Simões, Paulo Machado, Elsa Melo, Susana Cardoso, Osvaldo Santos, Carla Nunes, Isabel Loureiro, Flávia Santos, Gilberto Alves, Cláudia Soar, Teresa O. Marsi, Ernestina Silva, Dora Pedrosa, Andrea Leça, Daniel Silva, Ana Galvão, Maria Gomes, Paula Fernandes, Ana Noné, Jaime Combadão, Cátia Ramalhete, Paulo Figueiredo, Patrícia Caeiro, Karine C. Fontana, Josimari T. Lacerda, Patrícia O. Machado, Raphaelle Borges, Flávio Barbosa, Dayse Sá, Germana Brunhoso, Graça Aparício, Amâncio Carvalho, Ana P. Garcia, Paula O. Fernandes, Adriana Santos, Nélio Veiga, Carina Brás, Inês Carvalho, Joana Batalha, Margarida Glória, Filipa Bexiga, Inês Coelho, Odete Amaral, Carlos Pereira, Cláudia Pinho, Nilson Paraíso, Ana I. Oliveira, Cristóvão F. Lima, Alberto P. Dias, Pedro Silva, Mário Espada, Mário Marques, Ana Pereira, Ana Mª Pereira, Mª Veiga-Branco, Filomena Pereira, Maria Ribeiro, Vera Lima, Ana I. Oliveira, Cláudia Pinho, Graça Cruz, Rita F. Oliveira, Luísa Barreiros, Fernando Moreira, Ana Camarneiro, Mª Helena Loureiro, Margarida Silva, Catarina Duarte, Ângelo Jesus, Agostinho Cruz, Maria Mota, Sandra Novais, Paulo Nogueira, Ana Pereira, Lara Carneiro, Paulo V. João, Teresa Maneca Lima, Anabela Salgueiro-Oliveira, Marina Vaquinhas, Pedro Parreira, Rosa Melo, João Graveto, Amélia Castilho, José H. Gomes, María S. Medina, Valeriana G. Blanco, Osvaldo Santos, Elisa Lopes, Ana Virgolino, Alexandra Dinis, Sara Ambrósio, Inês Almeida, Tatiana Marques, Mª João Heitor, Miguel A. Garcia-Gordillo, Daniel Collado-Mateo, Pedro R. Olivares, José A. Parraça, José A. Sala, Amélia Castilho, João Graveto, Pedro Parreira, Anabela Oliveira, José H. Gomes, Rosa Melo, Marina Vaquinhas, Mónica Cheio, Agostinho Cruz, Olívia R. Pereira, Sara Pinto, Adriana Oliveira, M. Conceição Manso, Carla Sousa, Ana F. Vinha, Mª Manuela Machado, Margarida Vieira, Beatriz Fernandes, Teresa Tomás, Diogo Quirino, Gustavo Desouzart, Rui Matos, Magali Bordini, Pedro Mouroço, Ana R. Matos, Mauro Serapioni, Teresa Guimarães, Virgínia Fonseca, André Costa, João Ribeiro, João Lobato, Inmaculada Z. Martin, Anita Björklund, Aida I. Tavares, Pedro Ferreira, Rui Passadouro, Sónia Morgado, Nuno Tavares, João Valente, Anabela C. Martins, Patrícia Araújo, Rosina Fernandes, Francisco Mendes, Cátia Magalhães, Emília Martins, Pedro Mendes, Rui Paulo, António Faustino, Helena Mesquita, Samuel Honório, Marco Batista, Josimari T. Lacerda, Angela B. Ortiga, Mª Cristina Calvo, Sônia Natal, Marta Pereira, Manuela Ferreira, Ana R. Prata, Paula Nelas, João Duarte, Juliana Carneiro, Ana I. Oliveira, Cláudia Pinho, Cristina Couto, Rita F. Oliveira, Fernando Moreira, Ana S. Maia, Michelle T. Oliveira, Anderson R. Sousa, Paulo P. Ferreira, Géssica M. Souza, Lívia F. Almada, Milena A. Conceição, Eujcely C. Santiago, Sandra Rodrigues, Gabriela Domingues, Irina Ferreira, Luís Faria, Adérito Seixas, Ana R. Costa, Ângelo Jesus, Américo Cardoso, Alexandra Meireles, Armanda Colaço, Agostinho Cruz, Viviane L. Vieira, Kellem R. Vincha, Ana Mª Cervato-Mancuso, Melissa Faria, Cláudia Reis, Marco P. Cova, Rita T. Ascenso, Henrique A. Almeida, Eunice G. Oliveira, Miguel Santana, Rafael Pereira, Eunice G. Oliveira, Henrique A. Almeida, Rita T. Ascenso, Rita Jesus, Rodrigo Tapadas, Carolina Tim-Tim, Catarina Cezanne, Matilde Lagoa, Sara S. Dias, Jorge Torgal, João Lopes, Henrique Almeida, Sandra Amado, Luís Carrão, Madalena Cunha, Luís Saboga-Nunes, Carlos Albuquerque, Olivério Ribeiro, Suzete Oliveira, Mª Carminda Morais, Emília Martins, Francisco Mendes, Rosina Fernandes, Cátia Magalhães, Patrícia Araújo, Ana R. Pedro, Odete Amaral, Ana Escoval, Victor Assunção, Henrique Luís, Luís Luís, Jennifer Apolinário-Hagen, Viktor Vehreschild, Ulrike Fotschl, Gerald Lirk, Anabela C. Martins, Isabel Andrade, Fernando Mendes, Verónica Mendonça, Sandra Antunes, Isabel Andrade, Nádia Osório, Ana Valado, Armando Caseiro, António Gabriel, Anabela C. Martins, Fernando Mendes, Paula A. Silva, Lisete M. Mónico, Pedro M. Parreira, Carla Carvalho, Carla Carvalho, Pedro M. Parreira, Lisete M. Mónico, Joana Ruivo, Vânia Silva, Paulino Sousa, José M. Padilha, Vera Ferraz, Graça Aparício, João Duarte, Carlos Vasconcelos, António Almeida, Joel Neves, Telma Correia, Helena Amorim, Romeu Mendes, Luís Saboga-Nunes, Madalena Cunha, Carlos Albuquerque, Elsa S. Pereira, Leonino S. Santos, Ana S. Reis, Helena R. Silva, João Rombo, Jorge C. Fernandes, Patrícia Fernandes, Jaime Ribeiro, Catarina Mangas, Ana Freire, Sara Silva, Irene Francisco, Ana Oliveira, Helena Catarino, Mª Anjos Dixe, Mª Clarisse Louro, Saudade Lopes, Anjos Dixe, Mª Anjos Dixe, Eva Menino, Helena Catarino, Fátima Soares, Ana P. Oliveira, Sara Gordo, Teresa Kraus, Catarina Tomás, Paulo Queirós, Teresa Rodrigues, Pedro Sousa, João G. Frade, Catarina Lobão, Cynthia B. Moura, Laysa C. Dreyer, Vanize Meneghetti, Priscila P. Cabral, Francisca Pinto, Paulino Sousa, Mª Raquel Esteves, Sofia Galvão, Ite Tytgat, Isabel Andrade, Nádia Osório, Ana Valado, Armando Caseiro, António Gabriel, Anabela C. Martins, Fernando Mendes, Mónica Casas-Novas, Helena Bernardo, Isabel Andrade, Gracinda Sousa, Ana P. Sousa, Clara Rocha, Pedro Belo, Nádia Osório, Ana Valado, Armando Caseiro, António Gabriel, Anabela C. Martins, Fernando Mendes, Fátima Martins, Montserrat Pulido-Fuentes, Isabel Barroso, Gil Cabral, M. João Monteiro, Conceição Rainho, Alessandro Prado, Yara M. Carvalho, Maria Campos, Liliana Moreira, José Ferreira, Ana Teixeira, Luís Rama, Maria Campos, Liliana Moreira, José Ferreira, Ana Teixeira, Luís Rama

**Affiliations:** Health Sciences Research Unit: Nursing, Collegue of College of Health Technology of Coimbra, Coimbra, Portugal; Sisters Hospitallers of the Sacred Heart of Jesus, Casa de Saúde Rainha Santa Isabel, Coimbra, Portugal; Research Unit for Sport and Physical Activity, Faculty of Sport Sciences and Physical Education, University of Coimbra, Coimbra, Portugal; Complementary Sciences- INESCC, Coimbra, Portugal; CUF Hospitals, Lisbon, Portugal; Breast Unit, Champalimaud Clinical Center, Lisbon, Portugal; Swiss Centre for Medical Simulation & Swiss Association of Simulation in Healthcare, 4031 Basel, Switzerland; Basel University Hospital, 4056 Basel, Switzerland; HELPO Association, 2750-318 Cascais, Portugal; Centro de Linguística, Universidade de Lisboa, Lisboa, Portugal; Centro Superior de Estudios Universitarios La Salle, Madrid, Spain; Universidade da Madeira, 9000-082 Funchal, Portugal; Serviço de Saúde da Região Autónoma da Madeira, E.P.E., 9004-514 Funchal, Portugal; Escola Superior de Enfermagem de Lisboa, 1700-063 Lisboa, Portugal; Instituto Superior Miguel Torga, Coimbra, 3000-132 Coimbra Portugal; Centro de Investigação do Núcleo de Estudos e Intervenção Cognitivo Comportamental, Universidade de Coimbra, Coimbra, 3001-802 Portugal; Escola Superior de Saúde de Leiria & Unidade de Investigação em Saúde, Instituto Politécnico de Leiria, 2411-901 Leiria, Portugal; National School of Public Health & Centro de Investigação em Saúde Pública, Universidade Nova de Lisboa, 1600-560 Lisboa, Portugal; Agrarian Superior School, Polytechnic Institute of Viseu, 3500-606 Viseu, Portugal; Laboratory of Microbiology, Department of Biological Sciences, Faculty of Pharmacy, University of Porto, 4050-313 Porto, Portugal; Unit for Multidisciplinary Biomedical Research, Institute of Biomedical Sciences Abel Salazar, University of Porto, 4050-313 Porto, Portugal; Agrupamento de Centros de Saúde (ACES) Baixo Mondego, 3080-199 Figueira da Foz, Portugal; Universidade de Trás-os-Montes e Alto Douro, Vila Real, 5001-801 Portugal; Centro de Salud La Paz, Badajoz, 06011 Badajoz España; Centro de Salud San Fernando, Badajoz, 06006 Badajoz España; Complejo Hospitalario Universitario de Badajoz, Badajoz, 06080 Badajoz España; Hospital Don Benito-Villanueva, Don Benito, 06400 Badajoz España; Centro de Salud Villanueva Norte, Villanueva de la Serena, 06700 Badajoz España; Centro de Salud Gevora, 06180 Gévora, Badajoz España; Universidade Fernando Pessoa, Porto, 4249-004 Portugal; Centro de Salud La Paz, Badajoz, 06011 España; Complejo Hospitalario Universitario de Badajoz, Badajoz, 06080 Badajoz España; Centro de Salud San Fernando, Badajoz, 06006 Badajoz España; Universidad de Extremadura, Badajoz, 06071 Badajoz España; Faculty of Psychology and Education Sciences, University of Coimbra, Coimbra, 3001-802 Coimbra Portugal; Universidade Federal de Viçosa, Viçosa, Minas Gerais 36570-900 Brasil; Escola Superior de Enfermagem de Coimbra, 3046-851 Coimbra, Portugal; Universidade Federal de Juiz de Fora, Juiz de Fora, Minas Gerais 36036-330 Brasil; Centro Hospitalar e Universitário de Coimbra, 3000-075 Coimbra, Portugal; Escola Superior de Enfermagem de Lisboa, 1700-063 Lisboa, Portugal; School of Health Sciences, University of Aveiro, 3810-193 Aveiro, Portugal; Universidade Fernando Pessoa, 4249-004 Porto, Portugal; Escola Superior de Saúde Dr. Lopes Dias, Instituto Politécnico de Castelo Branco, 6000-767 Castelo Branco, Portugal; Cabildo de Lanzarote, 35500 Arrecife, Lanzarote, Las Palmas España; Servicio Canario de la Salud, 35500 Arrecife, Lanzarote, Las Palmas España; Universidad de Las Palmas de Gran Canaria, 35001 Las Palmas de Gran Canaria, Las Palmas España; Escola Superior de Saúde, Instituto Politécnico de Bragança, 5300-121 Bragança, Portugal; Escola Superior de Saúde de Santarém, Santarém, 2005-075 Portugal; Escola Superior de Enfermagem de Coimbra, Coimbra, 3046-851 Portugal; Escola Superior de Enfermagem de Lisboa, Lisboa, 1700-063 Portugal; Hospital Distrital de Santarém, EPE, Santarém, 2005-177 Portugal; Universidade de Coimbra, 3004-504 Coimbra, Portugal; Universidade da Madeira, 9000-082 Funchal, Portugal; Federal University of Rio Grande do Sul, Porto Alegre Rio Grande do Sul, 90050-170 Brasil; Universidade Federal de Santa Catarina, Florianópolis, 88040-900 Brasil; Instituto Superior de Serviço Social do Porto, 44600-362 Sra. da Hora, Portugal; Escola Superior de Tecnologia da Saúde de Coimbra, São Martinho do Bispo, 3046-854 Coimbra, Portugal; Escola Superior de Tecnologia da Saúde de Coimbra, São Martinho do Bispo, 3046-854 Coimbra, Portugal; Escola Superior de Tecnologia da Saúde de Coimbra, São Martinho do Bispo, 3046-854 Coimbra, Portugal; Escola Superior de Saúde, Instituto Politécnico de Viseu, 3504-510 Viseu, Portugal; Universidade Federal de Alagoas, Maceió, Alagoas, 57072-900 Brasil; Faculdade de Ciências do Desporto e Educação Física, Universidade de Coimbra, 3040-248 Coimbra, Portugal; Faculty of Psychology and Education Sciences, University of Coimbra, Coimbra, 3000-115 Portugal; Escola Superior da Saúde da Cruz Vermelha Portuguesa, Lisboa, 1300-125 Portugal; Universidade de Santa Cruz do Sul, Santa Cruz do Sul, Rio Grande do Sul 96815-900 Brasil; Escola Superior de Saúde Dr. Lopes Dias, Instituto Politécnico de Castelo Branco, 6000-767 Castelo Branco, Portugal; Instituto Politécnico de Portalegre, 7301-901 Portalegre, Portugal; Instituto Politécnico de Bragança, Bragança, 5300-121 Portugal; Universidade do Sul de Santa Catarina, Palhoça, Santa Catarina 88137-270 Brasil; Secretaria Municipal de Saúde de Palhoça, Palhoça, Santa Catarina 88132-149 Brasil; Universidad de Las Palmas de Gran Canaria, 35001 Las Palmas de Gran Canaria, Las Palmas España; Cabildo de Lanzarote, 35500 Lanzarote, Las Palmas España; Universidade de Aveiro, 3810-193 Aveiro, Portugal; Escola Superior de Saúde Dr. Lopes Dias, Castelo Branco, 6000-767 Portugal; Universidade do Sul de Santa Catarina, Palhoça, Santa Catarina 88137-270 Brasil; Centro de estudos em educação, tecnologias e saúde, Instituto Politécnico de Viseu, 3504-510 Viseu, Portugal; Casa de Saúde de São Mateus, Viseu, 3500-106 Portugal; Universidade Católica Portuguesa, Viseu, 3504-505 Portugal; Escola Nacional de Saúde Pública, Lisboa, 1600-560 Portugal; Research Centre Sports Sciences, Health Sciences and Human Development, University of Trás-os-Montes e Alto Douro, Vila Real, 5001-801 Portugal; Polytechnic Institute of Viseu, Viseu, 3504-510 Portugal; Unidade de Saúde Pública do ACES Douro I—Marão e Douro Norte, Administração Regional de Saúde do Norte, IP, 5000-524 Vila Real, Portugal; Núcleo de Investigação e Intervenção no Idoso, Departamento de Tecnologias de Diagnóstico e Terapêutica, Escola Superior de Saúde, Instituto Politécnico de Bragança, Bragança, 5300-253 Portugal; Centro de Investigação de Montanha, Escola Superior Agrária, Instituto Politécnico de Bragança, 5300-253 Bragança, Portugal; Escola Superior de Saúde, Instituto Politécnico de Bragança, 5300-253 Bragança, Portugal; Programa de Prevenção e Controlo de Infeções e de Resistência aos Antimicrobianos, Unidade Local de Saúde do Nordeste, Bragança, 5301-852 Portugal; Departamento de Urgência e Emergência Unidade, Unidade Local de Saúde do Nordeste, Bragança, 5301-852 Portugal; Departamento de Enfermagem, Escola Superior de Saúde, Instituto Politécnico de Bragança, 5300-121 Bragança, Portugal; Centro de Investigação em Desporto, Saúde e Desenvolvimento Humano, Departamento de Enfermagem, Escola Superior de Saúde, Instituto Politécnico de Bragança, 5300-121 Bragança, Portugal; Health Superior School of Alto Ave, 4830-345 Póvoa de Lanhoso, Portugal; Unidade de Investigação e Formação sobre Adultos e Idosos, Instituto de Ciências Biomédicas Abel Salazar, Universidade do Porto, 4050-313 Porto, Portugal; Center for Health Technology and Services Research, Faculdade de Medicina, Universidade do Porto, 4200-450 Porto, Portugal; Universidade de Aveiro, 3810-193 Aveiro, Portugal; Escola Superior de Enfermagem, Universidade do Minho, Braga, 4710-057 Portugal; Faculty of Medicine, University of Coimbra, 3000-354 Coimbra, Portugal; Centro de responsabilidade integrado, Serviço Psiquiatria, Centro Hospitalar e Universitário de Coimbra, 3000-354 Coimbra, Portugal; University of Trás-os-Montes e Alto Douro, Vila Real, 5001-801 Portugal; Research Centre Sports Sciences, Health Sciences and Human Development, University of Trás-os-Montes e Alto Douro, Vila Real, 5001-801 Portugal; Unidade de Saúde Pública, ACES Douro I, Marão e Douro Norte, Administração Regional de Saúde do Norte, 5000-524 Vila Real, Portugal; Faculdade Nobre, Feira de Santana, Bahia 44001-008 Brasil; Secretaria de Saúde do Estado da Bahia, Salvador, Bahia 41745-900 Brasil; Hospital Estadual da Criança, Vila Valqueire, Rio de Janeiro Brasil; Escola Superior de Tecnologia da Saúde, Instituto Politécnico do Porto, 4400-330 Vila Nova de Gaia, Portugal; Centro Hospitalar de São João, EPE, Porto, 4200-319 Porto Portugal; Unidade Local de Saúde de Matosinhos, EPE, 4454 509 Senhora da Hora, Portugal; College of Health Technology, Polytechnic Institute of Coimbra, São Martinho do Bispo, 3046-854 Coimbra, Portugal; College of Health Technology, Polytechnic Institute of Coimbra, São Martinho do Bispo, 3046-854 Coimbra, Portugal; College of Health Technology, Polytechnic Institute of Coimbra, São Martinho do Bispo, 3046-854 Coimbra, Portugal; Escola Superior de Saúde de Leiria, Instituto Politécnico de Leiria, 2411-901 Leiria, Portugal; School of Health Sciences, Polytechnic institute of Leiria, 2411-901 Leiria, Portugal; Unidade de Investigação Inclusão e Acessibilidade em Acção “iACT” e Escola Superior Educação e Ciências Sociais, Instituto Politécnico de Leiria, 2411-901 Leiria, Portugal; Unidade de Investigação em Saúde, Escola Superior de Saúde de Leiria eCADR& Centro de Investigação Didática e Tecnologia na Formação de Formadores, Universidade de Aveiro, 3810-193 Aveiro, Portugal; Instituto Politécnico de Leiria, 2411-901 Leiria, Portugal; Universidade da Beira Interior, Covilhã, 6201-001 Portugal; Unidade de Saúde Pública, Agrupamento de Centros de Saúde Cova da Beira, 6200-251 Covilhã, Portugal; Centre of Studies on Geography and Spatial Planning, University of Coimbra, 3000-043 Coimbra, Portugal; Research Centre for Geography and Regional Planning, Universidade Nova de Lisboa, 1069-061 Lisboa, Portugal; Biomedical Sciences Department, Paulista University, São Paulo, 04026-002 Brazil; Pharmacy Department, Paulista University, São Paulo, 04026-002 Brazil; Molecular Laboratory of Diagnosis, ABC Medical School, Santo André, São Paulo 09080-650 Brazil; Biomedical Science Department, College of Health Technology of Coimbra, Polytechnic Institute of Coimbra, São Martinho do Bispo, 3046-854 Coimbra, Portugal; Escola Superior de Saúde, Instituto Politécnico de Viseu, 3504-510 Viseu, Portugal; Hospital Sousa Martins, 6300-858 Guarda, Portugal; Instituto de Medicina Preventiva e Saúde Pública, Faculdade de Medicina, Universidade de Lisboa, 1649-028 Lisboa, Portugal; Instituto de Saúde Ambiental, Faculdade de Medicina, Universidade de Lisboa, 1649-028 Lisboa, Portugal; Faculdade de Medicina, Universidade de Lisboa, 1649-028 Lisboa, Portugal; Oslo and Akershus University College of Applied Sciences, 0167 Oslo, Norway; Newcastle University Institute for Ageing, Newcastle upon Tyne, NE4 5PL United Kingdom; Hospital de Santo André, Centro Hospitalar de Leiria, 2410-197 Leiria, Portugal; School of Health Sciences, Polytechnic institute of Leiria, 2411-901 Leiria, Portugal; Secção Autónoma das Ciências da Saúde, Universidade de Aveiro, 3810-193 Aveiro, Portugal; School of Health Sciences, University of Aveiro, 3810-193 Aveiro, Portugal; Unidade de Investigação e Formação sobre Adultos e Idosos, Instituto de Ciências Biomédicas Abel Salazar, Universidade do Porto, 4050-313 Porto, Portugal; Faculdade de Medicina da Universidade do Porto, 4200-450 Porto, Portugal; Universidade de Aveiro, 3810-193 Aveiro, Portugal; Instituto Superior De Serviço Social Do Porto, 44600-362 Sra. da Hora, Portugal; Escola Superior de Educação de Viseu, 3504-501 Viseu, Portugal; Center for Health Technology and Services Research, Faculdade de Medicina, Universidade do Porto, 4200-450 Porto, Portugal; Research in Education and Community Intervention, Escola Superior de Saúde Jean Piaget, Silves, 8300-025 Portugal; Escola Superior de Saúde Jean Piaget, Silves, 8300-025 Portugal; Cognitive and Behavioural Centre for Research and Intervention, Faculty of Psychology and Education Sciences, University of Coimbra, 3000-115 Coimbra, Portugal; Centro de Investigação do Núcleo de Estudos e Intervenção Cognitivo-Comportamental, Faculdade de Psicologia e Ciências da Educação, Universidade de Coimbra, 3001-802 Coimbra, Portugal; Instituto Superior Miguel Torga, Coimbra, 3000-132 Portugal; Federal University of Rio Grande do Sul, Porto Alegre, Rio Grande do Sul 90050-170 Brasil; Escola Superior de Tecnologia da Saúde de Coimbra, São Martinho do Bispo, 3046-854 Coimbra, Portugal; Municipality of Nordeste, 9630 Nordeste, Azores Portugal; University of Brasilia, Brasília, Distrito Federal 70910-900 Brasil; Universidad de Extremadura, Badajoz, 06071 España; Chrononutrition Laboratory, Department of Physiology, Faculty of Science, University of Extremadura, Badajoz, 06071 España; Department of Anestesiology, Complejo Hospitalario Universitario de Badajoz, 06080 Badajoz, España; Centro de Salud La Paz, Badajoz, 06011 España; Aragon Institute for Health Research, Zaragoza, 50009 España; ABC Medical School, Santo André, São Paulo 09210-180 Brazil; Department of Complementary Sciences, College of Health Technology of Coimbra, Polytechnic Institute of Coimbra, São Martinho do Bispo, 3046-854 Coimbra, Portugal; Biomedical Science Department, College of Health Technology of Coimbra, Polytechnic Institute of Coimbra, São Martinho do Bispo, 3046-854 Coimbra, Portugal; Biomedical Sciences Department, Paulista University, São Paulo, 04026-002 Brazil; Federal University of São Paulo, Diadema campus, Diadema, São Paulo 09913-030 Brazil; Universidade da Beira Interior, Covilhã, 6201-001 Portugal; Centro Hospitalar Cova da Beira, Covilhã, 6200-251 Portugal; Departamento de Ciências da Saúde, Universidade Católica Portuguesa, Viseu, 3504-505 Portugal; Centro de Investigação Interdisciplinar em Saúde, Universidade Católica Portuguesa, 3504-505 Viseu, Portugal; Escola Superior de Saúde, Instituto Politécnico de Viseu, 3504-510 Viseu, Portugal; Unidade de Saúde Familiar Grão Vasco, Viseu, 3500-177 Portugal; Centro de estudos em educação, tecnologias e saúde, Instituto Politécnico de Viseu, 3504-510 Viseu, Portugal; School of Health Sciences, Polytechnic Institute of Guarda, 6301-559 Guarda, Portugal; Research Unit for Inland Development, Polytechnic Institute of Guarda, 6301-559 Guarda, Portugal; Centro de Ciências da Saúde, Departamento de Saúde Pública, Universidade Federal de Santa Catarina, Florianópolis, Santa Catarina 88040-900 Brasil; College of Health Technology, Polytechnic Institute of Coimbra, 340-162 Coimbra, Portugal; School of Health Sciences, Polytechnic institute of Leiria, 2411-901 Leiria, Portugal; Oporto Portuguese Institute of Oncology FG, EPE, 4200-072 Porto, Portugal; Porto Nursing School, 4200-072 Porto, Portugal; Oporto Central Hospital, 4099-001 Porto, Portugal; Escola Superior Escola Superior de Enfermagem do Porto, 4200-072 Porto, Portugal; Center for Health Technology and Services Research, Faculdade de Medicina, Universidade do Porto, 4200-450 Porto, Portugal; Centro Hospitalar do Porto, 4099-001 Porto, Portugal; Instituto de Ciências da Saúde, Universidade Católica Portuguesa, 1649-023 Lisboa, Portugal; Escola Superior de Enfermagem de S. João de Deus, Universidade de Évora, 7004-516 Évora, Portugal; School of Health Sciences, University of Aveiro, 3810-193 Aveiro, Portugal; Health Research Unit & School of Health Sciences, Polytechnic institute of Leiria, 2411-901 Leiria, Portugal; Escola Superior de Enfermagem do Porto, Porto, 4200-072 Portugal; Faculdade de Economia, Universidade de Coimbra, 3004-512 Coimbra, Portugal; Universidade de Trás-os-Montes e Alto Douro, Vila Real, 5001-801 Portugal; Centro Hospitalar de Trás-os-Montes e Alto Douro, Vila Real, 5000-508 Portugal; Faculdade de Economia, Universidade de Coimbra, 3004-512 Coimbra, Portugal; Centro Hospitalar do Porto, 4099-001 Porto, Portugal; Escola Superior de Saúde, Instituto Politécnico de Viana do Castelo, 4900-347 Viana do Castelo, Portugal; Centro de Estudos e Investigação em Saúde, Universidade de Coimbra, 3004-512 Coimbra, Portugal; Escola Superior de Tecnologia da Saúde do Porto, Instituto Politécnico do Porto, Vila Nova de Gaia, 4400-330 Portugal; Escola Superior de Enfermagem de Coimbra, Coimbra, 3046-851 Portugal; Research in Education and Community Intervention, Escola Superior de Saúde Jean Piaget, 4405-678 Gulpilhares, Vila Nova de Gaia Portugal; Instituto de Ciências Biomédicas Abel Salazar, Universidade do Porto, 4050-313 Porto, Portugal; Escola Superior de Saúde, Instituto Politécnico de Santarém, Santarém, 2005-075 Portugal; Escola Superior de Enfermagem S. João de Deus, Universidade de Évora, 7000-811 Évora, Portugal; Escola Superior de Enfermagem de Coimbra, 3046-851 Coimbra, Portugal; Faculdade de Psicologia e Ciências da Educação, Universidade de Coimbra, 3001-802 Coimbra, Portugal; Instituto Politécnico de Viana do Castelo, 4900-347 Viana do Castelo, Portugal; Escola Superior de Saúde, Instituto Politécnico de Santarém, 2005-075 Santarém, Portugal; Unidade de Investigação do Instituto Politécnico de Santarém, 2001-904 Santarém, Portugal; Unidade de Monitorização de Indicadores em Saúde, Instituto Politécnico de Santarém, 2005-075 Santarém, Portugal; Life Quality Research Centre, Instituto Politécnico de Santarém, 2001-904 Santarém, Portugal; Hospital Prof. Doutor Fernando Fonseca E.P.E., Amadora, 2720-276 Portugal; Universidade de Trás-os-Montes e Alto Douro, Vila Real, 5001-801 Portugal; Instituto Politécnico de Bragança, Bragança, 5300-121 Bragança Portugal; Universidad de León, 24004 León, España; Instituto de Ciências da Saúde, Universidade Católica Portuguesa, Lisboa, 1649-023 Portugal; Escola Superior de Enfermagem de Coimbra, 3046-851 Coimbra, Portugal; Instituto Universitário de Ciências Psicológicas, Sociais e da Vida, 1149-041 Lisboa, Portugal; School of Health Sciences, Polytechnic institute of Leiria, 2411-901 Leiria, Portugal; Escola Superior de Enfermagem da Cruz Vermelha Portuguesa de Oliveira de Azeméis,, 3720-126 Oliveira de Azeméis, Portugal; Oulu University of Applied Sciences, 90250 Oulu, Finlândia; Unidade de Saúde Familiar - Espaço Saúde, Porto, 4100-503 Portugal; Escola Superior de Enfermagem da Cruz Vermelha Portuguesa, 3720-126 Oliveira de Azeméis, Portugal; Administração Regional de Saúde de Lisboa e Vale do Tejo, 1749-096 Lisboa, Portugal; Health Research Unit & School of Health Sciences, Polytechnic institute of Leiria, 2411-901 Leiria, Portugal; Escola Superior de Enfermagem de Coimbra, 3046-851 Coimbra, Portugal; Health Research Unit & School of Health Sciences, Polytechnic institute of Leiria, 2411-901 Leiria, Portugal; Departamento de Saúde Pública & Centro de Estudos de Doenças Crónicas, Faculdade de Ciências Médicas, Universidade Nova de Lisboa, 1169-056 Lisboa, Portugal; School of Health Sciences, Polytechnic institute of Leiria, 2411-901 Leiria, Portugal; Hospital de Santo André, Centro Hospitalar de Leiria, 2410-197 Leiria, Portugal; Faculdade de Medicina, Universidade José do Rosario Vellano, Divinópolis, Minas Gerais 35502-634 Brasil; Pontifícia Universidade Católica de Campinas, Campinas, São Paulo 13086-900 Brasil; Secção Autónoma das Ciências da Saúde, Universidade de Aveiro, Aveiro, 3810-193 Portugal; Instituto de Ciências Biomédicas Abel Salazar, Universidade do Porto, 4050-313 Porto, Portugal; Escola Superior de Enfermagem do Porto, 4200-072 Porto, Portugal; Universidade de São Paulo, 05403-000 São Paulo, Brasil; Escola Superior de Saúde Dr. Lopes Dias, Castelo Branco, 6000-767 Portugal; Universidade Católica Portuguesa, Lisboa, 1649-023 Portugal; Escola Superior de Enfermagem do Porto, 4200-072 Porto, Portugal; Escola Superior de Enfermagem da Cruz Vermelha Portuguesa de Oliveira de Azeméis, 3720-126 Oliveira de Azeméis, Portugal; Academia de Teatro, 4465-095 S. Mamede de Infesta, Portugal; Escola Superior de Tecnologia da Saúde de Lisboa, 1990-096 Lisboa, Portugal; Unidade de Investigação em Saúde, Escola Superior de Saúde de Leiria, 2411-901 Leiria, Portugal; Hospital Santa Maria Maior de Barcelos, 4754-909 Barcelos, Portugal; Centro de Estudos e Investigação em Saúde, Faculdade de Economia, Universidade de Coimbra, 3004-512 Coimbra, Portugal; Universidade Católica Portuguesa/Porto, 4202-401 Porto, Portugal; Escola Superior de Enfermagem do Porto, 4200-072 Porto, Portugal; Health Research Unit & School of Health Sciences, Polytechnic institute of Leiria, 2411-901 Leiria, Portugal; Instituto de Medicina Molecular, Universidade de Lisboa, 1649-028 Lisboa, Portugal; Faculdade de Motricidade Humana, Universidade de Lisboa, Cruz quebrada, 1499-002 Lisboa Portugal; Faculdade de Medicina, Universidade de Porto, 4200-319 Porto, Portugal; Escola Superior de Tecnologias de Saúde, Instituto Politécnico de Lisboa, 1990-096 Lisboa, Portugal; Escola Superior de Saúde, Universidade Atlântica, 2745-615 Barcarena, Portugal; Instituto de Telecomunicações, Lisboa, 1049-001 Portugal; Universidade do Sul de Santa Catarina, Palhoça, Santa Catarina 88137-270 Brasil; Universidade Federal de Santa Catarina, Florianópolis, Santa Catarina 88040-900 Brasil; Escola Superior de Enfermagem de Lisboa, Lisboa, 1700-063 Portugal; Escola Superior de Enfermagem de Coimbra, 3046-851 Coimbra, Portugal; Faculdade de Psicologia e Ciências da Educação, Universidade de Coimbra, 3001-802 Coimbra, Portugal; Escola Superior de Enfermagem de Coimbra, Coimbra, 3046-851 Portugal; Escola Superior de Tecnologia da Saúde, Instituto Politécnico do Porto, 4400-330 Vila Nova de Gaia, Portugal; Farmácia Outeiro do Linho, 4440-762 Valongo, Portugal; Hospital Militar Regional n.°1, Porto, 4150-113 Portugal; Centro Hospitalar do Porto, 4099-001 Porto, Portugal; Escola Superior de Enfermagem de Coimbra, 3046-851 Coimbra, Portugal; Universidade Federal de Viçosa, Viçosa, Minas Gerais 36570-900 Brasil; Escola Superior de Enfermagem da Cruz Vermelha Portuguesa de Oliveira de Azeméis, 3720-126 Oliveira de Azeméis, Portugal; Universidade Católica Portuguesa, Porto, 4202-401 Portugal; Unidade de Saúde Familiar - Espaço Saúde, Porto, 4100-503 Portugal; Administração Regional de Saúde do Norte, Porto, 4000-447 Portugal; Universidade Católica Portuguesa, Porto, 4202-401 Portugal; Centro Hospitalar De São João, E.P.E., Porto, 4200-319 Portugal; Escola Superior de Saúde, Instituto Politécnico de Setúbal, 2910-761 Setúbal, Portugal; Faculdade de Economia, Universidade de Coimbra, 3004-512 Coimbra, Portugal; Universidade de Aveiro, 3810-193 Aveiro, Portugal; Escola Superior de Enfermagem da Cruz Vermelha Portuguesa de Oliveira de Azeméis, 3720-126 Oliveira de Azeméis, Portugal; Ordem dos Enfermeiros da zona Centro, Coimbra, 3000-076 Portugal; Escola Superior de Enfermagem da Cruz Vermelha Portuguesa, 3720-126 Oliveira de Azeméis, Portugal; Escola Superior de Enfermagem, Universidade do Minho, Braga, 4710-057 Portugal; School of Health Sciences, Polytechnic institute of Leiria, 2411-901 Leiria, Portugal; School of Health Sciences, Polytechnic institute of Leiria, 2411-901 Leiria, Portugal; Hospital de Santo André, Centro Hospitalar de Leiria, 2410-197 Leiria, Portugal; Hospital de Santo André, Centro Hospitalar de Leiria, 2410-197 Leiria, Portugal; School of Health Sciences, Polytechnic institute of Leiria, 2411-901 Leiria, Portugal; Research Centre in Physical Activity, Health and Leisure, Faculty of Sports, University of Porto, 4200-450 Porto, Portugal; Lab 3R – Respiratory Research and Rehabilitation Laboratory, School of Health Sciences, University of Aveiro, 3810-193 Aveiro, Portugal; Unidade de Surdos, Escola de Referência para a Educação Bilingue, 3830-195 Ílhavo, Portugal; Center for Research and Development in Mathematics and Applications, University of Aveiro, 3810-193 Aveiro, Portugal; Department of Mathematics, University of Aveiro, 3810-193 Aveiro, Portugal; School of Health Care, Polytechnic Institute of Setúbal, 2910-761 Setúbal, Portugal; Centro de Linguística da Universidade de Lisboa, Faculdade de Letras, Universidade de Lisboa, 1600-214 Lisboa, Portugal; Center for Health Technology and Services Research, School of Health Sciences, University of Aveiro, Aveiro, 3810-193 Portugal; Escola Superior de Enfermagem de Coimbra, 3046-851 Coimbra, Portugal; Unidade de Investigação em Ciências da Saúde: Enfermagem, Escola Superior de Enfermagem de Coimbra, 3046-851 Coimbra, Portugal; Universidade Federal de Santa Catarina, Florianópolis, Santa Catarina 88040-900 Brasil; School of Health Sciences, University of Aveiro, Aveiro, 3810-193 Aveiro, Portugal; Peroneo Centro Terapêutico Lda, Amieiro, Montemor-o-velho, 3140-021 Arazede Portugal; Institute for Research in Biomedicine, University of Aveiro, Aveiro, 3810-292 Portugal; Department of Physiotherapy, North Polytechnic Institute of Health, Gandra, 4585-116 Portugal; Center for Health Technology and Services Research, University of Aveiro, Aveiro, 3810-193 Portugal; School of Health Sciences, University of Aveiro, 3810-193 Aveiro, Portugal; Research Centre in Physical Activity, Health and Leisure, Faculty of Sports, University of Porto, 4200-450 Porto, Portugal; Irmandade da Misericórdia de Albergaria-a-Velha, 3850-069 Albergaria-a-Velha, Portugal; Faculdade de Psicologia e Ciências da Educação, Universidade de Coimbra, 3001-802 Coimbra, Portugal; Gabinete de Apoio Psicológico, Instituto Superior Miguel Torga, Coimbra, 3000-132 Portugal; School of Health Sciences, University of Aveiro, 3810-193 Aveiro, Portugal; Rehabilitation Science Institute and Department of Physical Therapy, University of Toronto, Toronto, Ontario M5S 1A1 Canada; Escola Superior de Saúde de Santarém, Santarém, 2005-075 Portugal; Escola Superior de Enfermagem de Coimbra, Coimbra, 3046-851 Portugal; Unidade de Investigação & Desenvolvimento em Enfermagem, Escola Superior de Enfermagem de Lisboa, Lisboa, 1700-063 Portugal; Hospital Distrital de Santarém, EPE, Santarém, 2005-177 Portugal; Universidade de Coimbra, 3004-504 Coimbra, Portugal; Instituto Superior Miguel Torga, Coimbra, 3000-132 Portugal; Centro de Tratamento Internacional VillaRamadas, 2460-355 Cela, Alcobaça, Portugal; Administração Regional de Saúde do Norte, Porto, 4000-447 Portugal; Escola Superior de Enfermagem do Porto, Porto, 4200-072 Portugal; Faculdade de Economia, Universidade do Porto, 4200-464 Porto, Portugal; Complejo Hospitalario Universitario de Badajoz, Badajoz, 06080 España; Centro de Salud La Paz, Badajoz, 06011 España; Universidad de Extremadura, Badajoz, 06071 España; Centro de Salud San Fernando, Badajoz, 06006 España; Department of Anestesiology, Complejo Hospitalario Universitario de Badajoz, 06080 Badajoz, España; Universidad de Extremadura, Badajoz, 06071 España; Centro de Salud La Paz, Badajoz, 06011 España; Escola Superior de Enfermagem de Lisboa, 1700-063 Lisboa, Portugal; Faculdade de Psicologia, Universidade de Lisboa, 1649-013 Lisboa, Portugal; Centro de Investigação do Desporto e da Atividade Física, Faculdade de Ciências do Desporto e Educação Física, 3040-156 Coimbra, Portugal; Fundação Capes, Ministério da Educação, 70.040-020 Brasília, Distrito Federal Brasil; Faculty of Medicine, University of Porto, Porto, 4200-450 Portugal; School of Education and Social Sciences, Polytechnic Institute of Leiria, Leiria, 2411-901 Portugal; Life Quality Research Centre – IPLeiria Branch, Polytechnic Institute of Leiria, Leiria, 2411-901 Portugal; Universidade de Aveiro, 3810-193 Aveiro, Portugal; Universidad de Las Palmas de Gran Canaria, 35001 Las Palmas de Gran Canaria, España; Centro de Estudos e Investigação em Saúde, Faculdade de Economia, Universidade de Coimbra, 3004-512 Coimbra, Portugal; Unidade de Cuidados na Comunidade da Ponte da Barca, 4980-620 Ponte da Barca, Portugal; Escola Superior de Educação, Instituto Politécnico de Castelo Branco, 6000-767 Castelo Branco, Portugal; Centro de Estudos em Educação, Tecnologias e Saúde, Escola Superior de Saúde, Instituto Politécnico de Viseu, 3504-510 Viseu, Portugal; Studio Osteopatico Franchi, 6512 Giubiasco, Switzerland; Faculty of Human Kinetics, University of Lisbon, 1649-028 Lisboa, Portugal; Escola Superior de Tecnologia da Saúde de Lisboa, Instituto Politécnico de Lisboa, 1549-020 Lisboa, Portugal; Escola Superior Desporto De Rio Maior, Instituto Politécnico de Santarém, 2040-413 Rio Maior, Portugal; Escola Superior de Tecnologia da Saúde do Porto, Instituto Politécnico do Porto, Vila Nova de Gaia, 4400-330 Portugal; Department of Legal Medicine and Forensic Sciences, Faculty of Medicine, University of Porto, 4200-319 Porto, Portugal; Portugal & Pharmaceutical Services, Centro Hospitalar de Vila Nova de Gaia/Espinho, EPE, Vila Nova de Gaia, 4400-129 Portugal; Núcleo de Investigação e Intervenção em Farmácia, Centro de Investigação em Saúde e Ambiente, Instituto Politécnico do Porto, Vila Nova de Gaia, 4400-330 Portugal; Secção Autónoma de Ciências da Saúde, Universidade de Aveiro, 3810-193 Aveiro, Portugal; Escola Superior de Tecnologia da Saúde de Lisboa, Instituto Politécnico de Lisboa, 1549-020 Lisboa, Portugal; Hospital SAMS – Serviços de Assistência Médico-social do Sindicato dos Bancários, 1849-017 Lisboa, Portugal; North Tyneside General Hospital, Tyne and Wear, NE29 8NH Newcastle UK; Life Quality Research Centre, Instituto Politécnico de Castelo Branco, 6000-767 Castelo Branco, Portugal; School of Education and Social Sciences, Polytechnic Institute of Leiria, 2411-901 Leiria, Portugal; Universidade Federal de Santa Catarina, Florianópolis, Santa Catarina 88040-900 Brasil; Health Research Unit & School of Health Sciences, Polytechnic institute of Leiria, 2411-901 Leiria, Portugal; Unidade de Saúde Pública, ACES Pinhal Litoral, ARS Centro, 3100-462 Pombal, Portugal; Hospital de Santo André, Centro Hospitalar de Leiria, 2410-197 Leiria, Portugal; Escola Superior de Tecnologia da Saúde, Instituto Politécnico do Porto, 4400-330 Vila Nova de Gaia, Portugal; Escola Superior de Tecnologia da Saúde, Instituto Politécnico do Porto, 4400-330 Vila Nova de Gaia, Portugal; Health Research Unit, School of Health Sciences, Polytechnic institute of Leiria, 2411-901 Leiria, Portugal; Hospital de Santa Maria, Centro Hospitalar Lisboa Norte, 1649-035 Lisboa, Portugal; Universidade Católica Portuguesa, Lisboa, 1649-023 Portugal; Associação Portuguesa de Pais e Amigos do Cidadão Deficiente Mental, 6200-050 Covilhã, Portugal; Faculdade de Motricidade Humana, Universidade de Lisboa, Cruz quebrada, 1499-002 Lisboa Portugal; Secção Autónoma das Ciências da Saúde, Universidade de Aveiro, Aveiro, 3810-193 Portugal; Secção Autónoma das Ciências da Saúde, Universidade de Aveiro, 3810-193 Aveiro, Portugal; Unidade de Cuidados na Comunidade, Centro de Saúde de Anadia, 3780-780 Anadia, Portugal; Research in Education and Community Intervention, Escola Superior de Saúde Jean Piaget, Silves, 8300-025 Portugal; Fisiatris - Recuperação Física, Lda, Sacavém, 2685-010 Portugal; Faculdade de Motricidade Humana, Universidade de Lisboa, Cruz quebrada, 1499-002 Lisboa Portugal; Escola Superior de Educação, Instituto Politécnico de Castelo Branco, 6000-767 Castelo Branco, Portugal; Research Centre of Sports Sciences, Health Sciences and Human Development, Universidade da Beira Interior, Covilhã, 6201-001 Covilhã Portugal; Centro de Estudos em Educação, Tecnologias e Saúde, Escola Superior de Saúde, Instituto Politécnico de Viseu, 3504-510 Viseu, Portugal; Escola Superior de Desporto de Rio Maior, Instituto Politécnico de Santarém, 2040-413 Rio Maior, Portugal; Research, Education and Community Intervention, Instituto Piaget, 3515-776 Galifonge, Viseu Portugal; Studio Osteopatico Franchi, 6512 Giubiasco, Switzerland; Escola Superior de Desporto de Rio Maior, Instituto Politécnico de Santarém, 2040-413 Rio Maior, Portugal; Centro Interdisciplinar de Estudo da Performance Humana, Faculdade de Motricidade Humana, Universidade Nova de Lisboa, 1499-002 Cruz Quebrada, Portugal; Centro de Investigação e Qualidade de Vida, Instituto Politécnico de Santarém, 2040-413 Rio Maior, Portugal; Research Centre in Sports Sciences, Health and Human Development, University of Trás-os-Montes and Alto Douro, Vila Real, 5001-801 Portugal; Universidade da Madeira, 9000-082 Funchal, Portugal; Lar Cantinho dos Avós - Santa Casa da Misericórdia de Melgaço, 4960-570 Melgaço, Portugal; Escola Superior de Saúde, Instituto Politécnico de Viana do Castelo, 4900-347 Viana do Castelo, Portugal; Unidade Local de Saúde do Alto Minho, EPE, 4904-858 Viana do Castelo, Portugal; Secretaria de Saúde do Estado da Bahia, Salvador, Bahia 41745-900 Brasil; Fundação de amparo à pesquisa e inovação do Espírito Santo, Vitória, Esprito Santo 29066-380 Brasil; Universidade Estadual de Feira de Santana, Feira de Santana, Bahia 44036-900 Brasil; Faculdade Nobre, Feira de Santana, Bahia 44001-008 Brasil; Escola Superior de Tecnologia da Saúde do Porto, Instituto Politécnico do Porto, Vila Nova de Gaia, 4400-330 Portugal; Centro de Investigação em Saúde e Ambiente, Instituto Politécnico do Porto, Vila Nova de Gaia, 4400-330 Portugal; Hospital de Santo Antonio, Centro Hospitalar do Porto, EPE, 4099-001 Porto, Portugal; Department of Legal Medicine and Forensic Sciences, Faculty of Medicine, University of Porto, 4200-319 Porto, Portugal; Pharmaceutical Services, Centro Hospitalar de Vila Nova de Gaia/Espinho, EPE, Vila Nova de Gaia, 4400-129 Portugal; Centro Hospitalar de São João, EPE, Porto, 4200-319 Portugal; Escola Superior de Saúde, Instituto Politécnico de Bragança, Bragança, 5300-253 Portugal; Unidade Local de Saúde da Guarda, E.P.E, 6300-858 Guarda, Portugal; Clínica Espregueira-Mendes Sports Centre, FIFA Medical Centre of Excellence, 4350-415 Porto, Portugal; Faculdade de Desporto, Universidade do Porto, 4200-450 Porto, Portugal; Unidade Local de Saúde da Guarda, E.P.E, 6300-858 Guarda, Portugal; Clínica Espregueira-Mendes Sports Centre, FIFA Medical Centre of Excellence, 4350-415 Porto, Portugal; Faculdade de Desporto, Universidade do Porto, 4200-450 Porto, Portugal; Centro de Ação Social do Concelho de Ílhavo, 3830-201 Ílhavo, Portugal; Zelar, Serviços de Apoio Domiciliário, 3810-232 Aveiro, Portugal; Walk’In Clinics, Esgueira, 3800-042 Aveiro, Portugal; São João de Deus Clinic, 1749-098 Lisboa, Portugal; Arrabida Hospital, 4400-346 Vila Nova de Gaia, Portugal; Yasmin Residence, 2500-064 Caldas da Rainha, Portugal; Faro unit, Algarve Hospital, 8000-386 Faro, Portugal; School of Health Sciences, University of Aveiro, Aveiro, 3810-193 Portugal; School of Health Sciences, Polytechnic institute of Leiria, Leiria, 2411-901 Portugal; Health Research Unit, Polytechnic institute of Leiria, Leiria, 2411-901 Portugal; Escola Superior de Educação, Instituto Politécnico de Castelo Branco, 6000-767 Castelo Branco, Portugal; Faculty of Human Kinetics, University of Lisbon, 1649-028 Lisboa, Portugal; Research, Education and Community Intervention, Instituto Piaget, 3515-776 Galifonge, Viseu Portugal; Studio Osteopatico Franchi, 6512 Giubiasco, Switzerland; Centro de Estudos em Educação, Tecnologias e Saúde, Escola Superior de Saúde, Instituto Politécnico de Viseu, 3504-510 Viseu, Portugal; Universidade Fernando Pessoa, Porto, 4249-004 Portugal; School of Health Sciences, Polytechnic institute of Leiria, 2411-901 Leiria, Portugal; Instituto de Ciências Sociais da Universidade de Lisboa, Universidade de Lisboa, 1600-189 Lisboa, Portugal; Centro de Investigação do Desporto e da Atividade Física, Faculdade de Ciências do Desporto e Educação Física, 3040-156 Coimbra, Portugal; Núcleo de Estudos em Saúde da População, Universidade Estadual do Sudoeste da Bahia, Itapetinga, Bahia 45700-000 Brasil; Faculdade de Desporto, Universidade do Porto, 4200-450 Porto, Portugal; Faculdade de Motricidade Humana, Universidade de Lisboa, Cruz quebrada, 1499-002 Lisboa Portugal; Casa de Saúde da Idanha, Instituto das Irmãs Hospitaleiras, 2605-077 Belas, Portugal; Instituto Politécnico de Portalegre, 7301-901 Portalegre, Portugal; Cognitive-Behavioural Research Centre, Faculty of Psychology and Education Sciences, University of Coimbra, 3001-802 Coimbra, Portugal; Instituto Superior Miguel Torga, Coimbra, 3000-132 Portugal; Centro de estudos em educação, tecnologias e saúde, Escola Superior de Saúde, Instituto Politécnico de Viseu, 3504-510 Viseu, Portugal; Escola Superior de Saúde, Instituto Politécnico de Viseu, 3504-510 Viseu, Portugal; Instituto Superior Miguel Torga, Coimbra, 3000-132 Portugal; Faculty of Medicine, University of Coimbra, 3000-548 Coimbra, Portugal; Centro de Investigação do Núcleo de Estudos e Intervenção Cognitivo Comportamental, Universidade de Coimbra, 3001-802 Coimbra, Portugal; Centre for Health Studies and Research, University of Coimbra, 3004-512 Coimbra, Portugal; Universidade Federal de Alagoas, Maceió, 57072-900 Alagoas Brasil; Instituto Federal de Alagoas, Maceió, 57035-660 Alagoas Brasil; Centro Universitário Cesmac, Maceió, 57051-160 Alagoas Brasil; Universidade do Porto, Porto, 4099-002 Portugal; Universidade de Coimbra, Coimbra, 3004-504 Portugal; Escola Superior de Saúde Dr. Lopes Dias, Instituto Politécnico de Castelo Branco, Castelo Branco, 6000-767 Portugal; Universidade do Sul de Santa Catarina, Palhoça, Santa Catarina 88137-270 Brasil; Secretaria Municipal de Saúde de Palhoça, Palhoça, Santa Catarina 88132-149 Brasil; Cabildo de Lanzarote, 35500 Arrecife, Lanzarote, Las Palmas España; Universidad de Las Palmas de Gran Canaria, 35001 Las Palmas de Gran Canaria, Las Palmas España; Servicio Canario de la Salud, 35500 Arrecife, Lanzarote, Las Palmas España; Escola Superior de Enfermagem de Coimbra, Coimbra, 3046-851 Portugal; Department for Health Psychology, FernUniversität in Hagen, 58084 Hagen, Germany; Universidade Federal do Pará, Belém, Pará 66075-110 Brasil; Escola Superior de Saúde, Universidade do Algarve, Faro, 8000-510 Portugal; Centro de Estudos em Educação, Tecnologias e Saúde, Escola Superior de Saúde, Instituto Politécnico de Viseu, 3504-510 Viseu, Portugal; Faculdade de Psicologia e Ciências da Educação, Universidade de Coimbra, 3001-802 Coimbra, Portugal; Escola Superior de Enfermagem de Coimbra, Coimbra, 3046-851 Portugal; Centro de Estudos e Investigação em Saúde, Faculdade de Economia,, Universidade de Coimbra, 3004-512 Coimbra, Portugal; Escola Superior de Saúde, Instituto Politécnico de Viana do Castelo, 4900-347 Viana do Castelo, Portugal; Direção geral de Saúde (DGS), 1049-005 Lisboa, Portugal; Instituto Politécnico de Bragança, Bragança, 5300-253 Portugal; Centro de Investigação de Montanha, Escola Superior Agrária, 5300-253 Bragança, Portugal; Escola Superior Agrária, Instituto Politécnico de Bragança, 5300-253 Bragança, Portugal; Centro de Estudos Transdisciplinares para o Desenvolvimento, Universidade de Trás-os-Montes e Alto Douro, 5000-801 Vila Real, Portugal; Escola Superior de Saúde, Instituto Politécnico de Bragança, 5300-121 Bragança, Portugal; Faculdade de Medicina, Universidade de Lisboa, 1649-028 Lisboa, Portugal; Universidade do Sul de Santa Catarina, Palhoça, Santa Catarina 88137-270 Brasil; Research Centre for Sports Sciences, Health Sciences and Human Development, University of Trás-os-Montes e Alto Douro, Vila Real, 5001-801 Portugal; Department of Sport Sciences, Exercise and Health, University of Trás-os-Montes e Alto Douro, Vila Real, 5001-801 Portugal; Polytechnic Institute of Setúbal, 2910-761 Setúbal, Portugal; College of Health Technology of Coimbra, Polytechnic Institute of Coimbra, São Martinho do Bispo, 3046-854 Coimbra Portugal; Universidade da Beira Interior, Covilhã, 6201-001 Portugal; Departamento de Farmacologia e Terapêutica, Faculdade de Medicina, Universidade de Coimbra, Coimbra, 3000-548 Portugal; Fundação Capes, Ministério da Educação, 70.040-020 Brasília, Distrito Federal Brasil; Faculdade de Ciências do Desporto e Educação Física, Universidade de Coimbra, 3040-248 Coimbra, Portugal; Universidade Estadual do Sudoeste da Bahia, Itapetinga, Bahia 45700-000 Brasil; Universidade Federal de Santa Catarina, Florianópolis, Santa Catarina 88040-900 Brasil; Universidade Tiradentes, Aracaju, Sergipe & Laboratório de Biociências da Motricidade Humana, 49030-620 Aracaju, Brazil; Cognitive-Behavioural Research Centre, Faculty of Psychology and Education Sciences, University of Coimbra, 3001-802 Coimbra, Portugal; Escola Superior de Enfermagem de Lisboa, Lisboa, 1700-063 Portugal; Instituto de Ciências da Saúde, Universidade Católica Portuguesa, Lisboa, 1649-023 Portugal; Escola Superior de Saúde, Instituto Politécnico de Viana do Castelo, 4900-347 Viana do Castelo, Portugal; Escola Superior de Tecnologia e Gestão, Instituto Politécnico de Bragança, 5300-253 Bragança, Portugal; Escola Superior de Saúde de Santarém, Santarém, 2005-075 Portugal; Escola Superior de Enfermagem do Porto, Porto, 4200-072 Portugal; Escola Superior de Enfermagem de Lisboa, Lisboa, 1700-063 Portugal; Escola Superior de Tecnologia da Saúde de Lisboa, Instituto Politécnico de Lisboa, 1549-020 Lisboa, Portugal; Faculdade de Psicologia e Ciências da Educação, Universidade de Coimbra, 3001-802 Coimbra, Portugal; Universidade de Lisboa, 1649-003 Lisboa, Portugal; Universidade de Aveiro, Aveiro, 3810-193 Portugal; Escola superior de tecnologia da saúde de Lisboa, 1990-096 Lisboa, Portugal; Cognitive and Behavioural Centre for Research and Intervention, Faculty of Psychology and Education Sciences, University of Coimbra, 3000-115 Coimbra, Portugal; Escola Superior de Saúde, Instituto Politécnico de Viseu, 3504-510 Viseu, Portugal; Universidade Católica Portuguesa, Viseu, 3504-505 Portugal; Universidade de Santa Cruz do Sul, Santa Cruz do Sul, Rio Grande do Sul 96815-900 Brasil; Instituto Superior da Maia, 4475-690 Avioso São Pedro, Portugal; Centro Interdisciplinar de Estudos de Género, Instituto Superior de Ciências Sociais e Políticas, 1300-663 Lisboa, Portugal; Faculdade de Psicologia e Ciências da Educação, Universidade de Coimbra, 3001-802 Coimbra, Portugal; College of Health Technology, Polytechnic Institute of Coimbra, São Martinho do Bispo, 3046-854 Coimbra Portugal; Unidade de Investigação e Formação sobre Adultos e Idosos, Instituto de Ciências Biomédicas Abel Salazar, Universidade do Porto, 4050-313 Porto, Portugal; Faculdade de Medicina da Universidade do Porto, 4200-450 Porto, Portugal; Center for Health Technology and Services Research, Faculdade de Medicina, Universidade do Porto, 4200-450 Porto, Portugal; Universidade de Aveiro, 3810–193 Aveiro, Portugal e Instituto Superior De Serviço Social Do Porto, 44600-362 Sra. da Hora, Portugal; Escola Superior de Desporto de Rio Maior, Instituto Politécnico de Santarém, 2040-413 Rio Maior, Portugal; Escola Superior de Enfermagem de Lisboa, Lisboa, 1700-063 Portugal; Escola Superior de Saúde, Instituto Politécnico de Santarém, 2005-075 Santarém, Portugal; School of Health Sciences, Polytechnic Institute of Setúbal, 2910-761 Setúbal, Portugal; Institute of Electronics and Informatics Engineering of Aveiro, 3810-193 Aveiro, Portugal; Department of Education, University of Aveiro, 3810-193 Aveiro, Portugal; Center for Health Technology and Services Research & School of Health Sciences, University of Aveiro, 3810-193 Aveiro, Portugal; Escola Superior de Tecnologia da Saúde de Coimbra, São Martinho do Bispo, 3046-854 Coimbra, Portugal; School of Health Sciences, University of Aveiro, 3810-193 Aveiro, Portugal; Pediatrics Emergency Department, Hospital Lusíadas, Porto, 4050 113 Portugal; School of Health Sciences, University of Aveiro, 3810-193 Aveiro, Portugal; Centro Hospitalar do Baixo Vouga, Aveiro, 3814-501 Portugal; Hospital Curry Cabral, Centro Hospitalar Lisboa Central, 1069-166 Lisboa, Portugal; Instituto de Ciências da Saúde, Universidade Católica Portuguesa, Lisboa, 1649-023 Portugal; Centro de Formação Multiperfil, Luanda, PR - 56Q Angola; Center for Health Technology and Services Research, University of Aveiro, Aveiro, 3810-193 Aveiro Portugal; School of Health Sciences, University of Aveiro, 3810-193 Aveiro, Portugal; Escola Superior de Tecnologia da Saúde de Coimbra, São Martinho do Bispo, 3046-854 Coimbra, Portugal; Centre of Investigation in Environment, Genetics and Oncobiology, Faculty of Medicine, University of Coimbra, 3000-548 Coimbra, Portugal; Biomedical Laboratory Sciences Department, University of Gothenburg, S-405 30 Gothenburg, Sweden; Biophysics and Biomathematics Institute, Institute for Biomedical Imaging and Life Sciences, Faculty of Medicine, University of Coimbra, 3000-354 Coimbra, Portugal; Oncobiology and Haematology Laboratory of Applied Molecular Biology & Clinical University of Hematology, Faculty of Medicine, University of Coimbra, 3000-354 Coimbra, Portugal; Radiation Oncology Department, Coimbra Hospital and Universitary Centre, 3000-075 Coimbra, Portugal; Clinical University of Hematology, Coimbra Hospital and Universitary Centre, 3000-075 Coimbra, Portugal; Imunology Institute, Faculty of Medicine, University of Coimbra, 3000-354 Coimbra, Portugal; Escola Superior de Tecnologia da Saúde de Lisboa, Instituto Politécnico de Lisboa, Lisboa, 1549-020 Portugal; Biomedical Science Department, College of Health Technology of Coimbra, Polytechnic Institute of Coimbra, São Martinho do Bispo, 3046-854 Coimbra, Portugal; Department of Clinical Chemistry and Transfusion Medicine, Sahlgrenska University Hospital, Göteborg, 413 45 Göteborg Switzerland; Complementary Sciences Department, College of Health Technology of Coimbra, Polytechnic Institute of Coimbra, São Martinho do Bispo, 3046-854 Coimbra, Portugal; Medical Laboratory Sciences Department, Al-Quds University, Abu Dis, P.O Box 89, Abu Dis, Palestinian National Authority; Blood Bank Service, Coimbra Hospital and Universitary Center, 3000-075 Coimbra, Portugal; Centro de Investigação do Núcleo de Estudos e Intervenção Cognitivo-Comportamental, Faculdade de Psicologia e Ciências da Educação, Universidade de Coimbra, 3001-802 Coimbra, Portugal; Instituto Superior Miguel Torga, Coimbra, 3000-132 Portugal; Escola Superior de Tecnologia e Gestão, Instituto Politécnico de Leiria, 2411-901 Leiria, Portugal; Centre for the Research and Technology of Agro-Environmental and Biological Sciences, University of Trás-os-Montes and Alto Douro, 5001-801 Vila Real, Portugal; Instituto de Telecomunicações, Instituto Politécnico de Leiria, 2411-901 Leiria, Portugal; Escola Superior de Tecnologia e Gestão, Instituto Politécnico de Leiria, 2411-901 Leiria, Portugal; Centre for the Research and Technology of Agro-Environmental and Biological Sciences, University of Trás-os-Montes and Alto Douro, 5001-801 Vila Real, Portugal; Instituto de Telecomunicações, Instituto Politécnico de Leiria, 2411-901 Leiria, Portugal; Escola Superior de Tecnologia da Saúde de Coimbra, São Martinho do Bispo, 3046-854 Coimbra Portugal; Escola Superior de Saúde, Universidade de Aveiro, 3810-193 Aveiro, Portugal; Center for Health Technology and Services Research, Universidade do Porto, 4200-450 Porto, Portugal; Instituto de Ciências Biomédicas Abel Salazar, Universidade do Porto, Porto, 4050-313 Porto Portugal; Interdisciplinary Centre of Marine and Environmental Research, Universidade do Porto, 4050-123 Porto, Portugal; School of Health Sciences, Polytechnic Institute of Leiria, 2411-901 Leiria, Portugal; School of Health Sciences, University of Aveiro, 3810-193 Aveiro, Portugal; Centre for Rapid and Sustainable Product Development, Polytechnic Institute of Leiria, 2430-028 Marinha Grande, Portugal; Centre for Rapid and Sustainable Product Development, Polytechnic Institute of Leiria, 2430-028 Marinha Grande, Portugal; Escola Superior de Enfermagem de Lisboa, 1700-063 Lisboa, Portugal; Faculdade de Psicologia, Universidade de Lisboa, 1649-013 Lisboa, Portugal; Escola Superior de Enfermagem de Coimbra, 3046-851 Coimbra, Portugal; Birmingham Hospitals, Birmingham, B15 2GW United Kingdom; Faculdade de Psicologia e Ciências da Educação, Universidade de Coimbra, 3001-802 Coimbra, Portugal; Instituto Superior de Ciências Sociais e Políticas, 1300-663 Lisboa, Portugal; Universidade Fernando Pessoa, Porto, 4249-004 Porto Portugal; Faculdade de Engenharia, Universidade do Porto, 4200-465 Porto, Portugal; Universidade de Trás-os-Montes e Alto Douro, Vila Real, 5001-801 Vila Real, Portugal; Centro de Medicina de Reabilitação da Região Centro - Rovisco Pais, 3064-908 Tocha, Portugal; Instituto de Engenharia de Sistemas e Computadores, Tecnologia e Ciência, 4200-465 Porto, Portugal; Cognitive-Behavioural Research Centre, Faculty of Psychology and Education Sciences, University of Coimbra, 3001-802 Coimbra, Portugal; Azores University, São Miguel, 9501-855 Ponta Delgada Portugal; Department of Education and Psychology, University of Aveiro, Aveiro, 3810-193 Aveiro Portugal; Center for Health Technology and Services Research, Faculty of Medicine, University of Porto, Porto, 4200-450 Porto Portugal; Faculty of Psychology and Education Sciences, University of Coimbra, Coimbra, 3000-115 Coimbra Portugal; Centro de investigação em educação e ciências do comportamento, Universidade de Aveiro, Aveiro, 3810-193 Aveiro Portugal; Instituto de Imagem Biomédica e Ciências da Vida, Faculdade de Medicina, Universidade de Coimbra, Coimbra, 3000-548 Coimbra Portugal; Centro de Investigação do Núcleo de Estudos e Intervenção Cognitivo Comportamental, Universidade de Coimbra, Coimbra, 3001-802 Coimbra Portugal; Instituto de Ciências da Saúde, Universidade Católica Portuguesa, Lisboa, 1649-023 Lisboa Portugal; Escola Superior de Enfermagem de Coimbra, 3046-851 Coimbra, Portugal; Instituto Universitário de Ciências Psicológicas, Sociais e da Vida, 1149-041 Lisboa, Portugal; University of Extremadura, Badajoz, 06071 Badajoz España; Flinders Health Economics Group, Flinders University, Adelaide, 5001 Australia; Centre for Health Economics, Monash University, Victoria, 3800 Australia; Universidade de Évora, 7004-516 Évora, Portugal; Instituto Superior Miguel Torga, Coimbra, 3000-132 Coimbra Portugal; Centro Hospitalar e Universitário de Coimbra, 3000-075 Coimbra, Portugal; Centro de Literaturas e Culturas Lusófonas e Europeias, Universidade de Lisboa, 1600-214 Lisboa, Portugal; Universidade de Aveiro, 3810-193 Aveiro, Portugal; School of Health Sciences, University of Aveiro, Aveiro, 3810-193 Aveiro Portugal; Centro Hospitalar do Baixo Vouga, Aveiro, 3814-501 Aveiro, Portugal; Escola Superior de Enfermagem do Porto, Porto, 4200-072 Porto Portugal; Cognitive-Behavioural Research Centre, Faculty of Psychology and Education Sciences, University of Coimbra, 3001-802 Coimbra, Portugal; Azores University, São Miguel, 9501-855 Ponta Delgada Portugal; Department of Psychological Medicine, Faculty of Medicine, University of Coimbra, 3000-354 Coimbra, Portugal; Unidade de Saúde Familiar – Topázio, Eiras, 3020-171 Coimbra Portugal; Instituto Superior Miguel Torga, Coimbra, 3000 Coimbra Portugal; University of Aveiro, Aveiro, 3810-193 Aveiro Portugal; Center for Health Technology and Services Research, Faculty of Medicine, University of Porto, Porto, 4200-450 Porto Portugal; Instituto de Imagem Biomédica e Ciências da Vida, Faculdade de Medicina, Universidade de Coimbra, 3000-548 Coimbra, Portugal; Escola Superior de Tecnologia e Gestão, Instituto Politécnico de Leiria, 2411-901 Leiria, Portugal; Laboratory of Bioinformatics, Graduate Program in Engineering and Computing, West Paraná State University, Cascavel, Paraná 85819-110 Brasil; Universidade Estadual de Campinas, Campinas, São Paulo 13083-970 Brasil; Instituto de Telecomunicações, Instituto Politécnico de Leiria, 2411-901 Leiria, Portugal; Fundação Universidade Regional de Blumenau, Blumenau, Santa Catarina 89012-900 Brasil; Departamento de Saúde Pública, Faculdade de Ciências Médicas, Universidade Nova de Lisboa, 1169-056 Lisboa, Portugal; Centro de Estudos de Doenças Crónicas, Universidade Nova de Lisboa, 1150-082 Lisboa, Portugal; Unidade de Investigação em Saúde, Escola Superior de Saúde de Leiria, 2411-901 Leiria, Portugal; Health Research Unit, Polytechnic institute of Leiria, 2411-901 Leiria, Portugal; School of Health Sciences, Polytechnic institute of Leiria, 2411-901 Leiria, Portugal; Escola Superior de Tecnologia da Saúde, Instituto Politécnico do Porto, 4400-330 Vila Nova de Gaia, Portugal; Universidade de Aveiro, 3810-193 Aveiro, Portugal; Hospital de Santa Maria, Centro Hospitalar Lisboa Norte, 1649-035 Lisboa, Portugal; Health Research Unit & School of Health Sciences, Polytechnic Institute of Leiria, 2411-901 Leiria, Portugal; Universidade Católica Portuguesa, Lisboa, 1649-023 Lisboa Portugal; Azores University, São Miguel, 9501-855 Ponta Delgada Portugal; Cognitive-Behavioural Research Centre, Faculty of Psychology and Education Sciences, University of Coimbra, 3001-802 Coimbra, Portugal; Centro Interdisciplinar de Ciências Sociais, Universidade dos Açores, São Miguel, 9501-855 Ponta Delgada, Região Autónoma dos Açores Portugal; School of Health Sciences, Polytechnic institute of Leiria, 2411-901 Leiria, Portugal; Hospital de Santo André, Centro Hospitalar de Leiria, 2410-197 Leiria, Portugal; Faculdade de Medicina, Universidade José do Rosario Vellano, Divinópolis, Minas Gerais, 35502-634 Brasil; Universidade Católica Portuguesa, Lisboa, 1649-023 Lisboa, Portugal; Health Research Unit, School of Health Sciences, Polytechnic Institute of Leiria, 2411-901 Leiria, Portugal; Escola Superior de Enfermagem do Porto, Porto, 4200-072 Porto Portugal; Federal University of Ceará, Fortaleza, Ceará 60020-181 Brazil; Federal University of Piauí, Teresina, Piauí, 64049-550 Brazil; Federal Institute of Education, Science and Technology of Ceará, Fortaleza, Ceará, 60040-531 Brazil; Federal University of Cariri, Juazeiro do Norte, Ceará, 63048-080 Brazil; Federal University of Ceará, Fortaleza, Ceará 60020-181 Brazil; Federal University of Cariri, Juazeiro do Norte, Ceará 63048-080 Brazil; Centro de Investigação em Psicologia, Escola de Psicologia, Universidade do Minho, 4710-057 Braga, Portugal; Cognitive and Behavioural Centre for Research and Intervention, Faculty of Psychology and Educational Sciences, University of Coimbra, 3000-115 Coimbra, Portugal; PROACTION Laboratory, 3001-802 Coimbra, Portugal; Escola Superior de Enfermagem de Coimbra, 3046-851 Coimbra, Portugal; Universidade Católica Portuguesa, Porto, 4202-401 Porto Portugal; Faculdade de Motricidade Humana, Universidade de Lisboa, Cruz quebrada, 1499-002 Lisboa Portugal; Secção Autónoma das Ciências da Saúde, Universidade de Aveiro, 3810-193 Aveiro, Portugal; Unidade de Cuidados na Comunidade, Centro de Saúde de Anadia, 3780-780 Anadia, Portugal; Cáritas Diocesana de Coimbra - Centro Rainha Santa Isabel, 3030-382 Coimbra, Portugal; Escola Superior de Tecnologia da Saúde de Coimbra, São Martinho do Bispo, 3046-854 Coimbra Portugal; Faculdade de Economia, Universidade de Coimbra, 3004-512 Coimbra, Portugal; Centro de Estudos e Investigação em Saúde, Faculdade de Economia, Universidade de Coimbra, 3004-512 Coimbra, Portugal; Cáritas Diocesana de Coimbra, Centro Rainha Santa Isabel, 3030-382 Coimbra Portugal; Fundação Mário da Cunha Brito, 3360-908 S. Pedro de Alva, Portugal; Escola Superior de Tecnologia da Saúde de Coimbra, Instituto Politécnico de Coimbra, São Martinho do Bispo, 3046-854 Coimbra, Portugal; Centro de Estudos e Investigação em Saúde da Universidade Coimbra, Faculdade de Economia, Universidade de Coimbra, 3004-512 Coimbra, Portugal; Universidade Federal de São Paulo, São Paulo, 04021-001 Brasil; Escola superior de Ciências da Santa Casa de Misericórdia de Vitória, Vitória- Esprito Santo, 29045-402 Brasil; Faculdade Católica Salesiana do Espírito Santo, Vitória- Esprito Santo, 29017-950 Brasil; Cognitive-Behavioural Research Centre, Faculty of Psychology and Education Sciences, University of Coimbra, Coimbra, 3001-802 Coimbra Portugal; Instituto Superior Miguel Torga, Coimbra, 3000-132 Coimbra Portugal; Cognitive-Behavioural Research Centre, Faculty of Psychology and Education Sciences, University of Coimbra, Coimbra, 3001-802 Coimbra Portugal; Instituto Superior Miguel Torga, Coimbra, 3000-132 Coimbra Portugal; Cognitive and Behavioural Centre for Research and Intervention, Faculty of Psychology and Education Sciences, University of Coimbra, 3000-115 Coimbra, Portugal; Cognitive and Behavioural Centre for Research and Intervention, Faculty of Psychology and Education Sciences, University of Coimbra, Coimbra, 3000-115 Coimbra Portugal; Centro de Estudos em Saúde, Escola Superior de Saúde, Universidade do Algarve, 8000-510 Faro, Portugal; Escola Superior de Tecnologia e Gestão, Instituto Politécnico de Leiria, 2411-901 Leiria, Portugal; Instituto de Telecomunicações, Instituto Politécnico de Leiria, 2411-901 Leiria, Portugal; Centre for the Research and Technology of Agro-Environmental and Biological Sciences, University of Trás-os-Montes and Alto Douro, 5001-801 Vila Real, Portugal; Escola Superior de Enfermagem de Coimbra, Coimbra, 3046-851 Coimbra Portugal; Research in Education and Community Intervention, Escola Superior de Saúde Jean Piaget, 4405-678 Gulpilhares, Vila Nova de Gaia Portugal; Instituto de Ciências Biomédicas Abel Salazar, Universidade do Porto, 4050-313 Porto, Portugal; Universidade Católica Portuguesa, Lisboa, 1649-023 Lisboa Portugal; Hospital de Santa Maria, Centro Hospitalar Lisboa Norte, 1649-035 Lisboa, Portugal; Escola Superior de Enfermagem de Coimbra, Coimbra, 3046-851 Coimbra Portugal; Research in Education and Community Intervention, Escola Superior de Saúde Jean Piaget, 4405-678 Gulpilhares, Vila Nova de Gaia Portugal; Instituto de Ciências Biomédicas Abel Salazar, Universidade do Porto, Porto, 4050-313 Porto Portugal; Faculdade de Engenharia, Universidade do Porto, 4200-465 Porto, Portugal; Universidade Fernando Pessoa, Porto, 4249-004 Porto Portugal; Centro Hospitalar do Porto, Porto, 4099-001 Porto Portugal; Secretaria de Saúde do Estado da Bahia, Salvador, Bahia 41745-900 Brasil; Universidade Estadual de Feira de Santana, Feira de Santana, Bahia 44036-900 Brasil; Faculdade Nobre, Feira de Santana, Bahia 44001-008 Brasil; Universidade Federal de Juiz de Fora, Juiz de Fora, Minas Gerais 36036-330 Brasil; Universidade do Estado do Rio de Janeiro, Rio de Janeiro, 20550-900 Brasil; Escola Superior de Enfermagem de Coimbra, Coimbra, 3046-851 Portugal; Universidade Federal de Viçosa, Viçosa, Minas Gerais 36570-900 Brasil; Escola Superior de Enfermagem, Universidade do Minho, Braga, 4710-057 Portugal; Faculdade de Farmácia, Universidade de Lisboa, 1649-003 Lisboa, Portugal; Instituto de Ciências Biomédicas Abel Salazar, Universidade do Porto, 4050-313 Porto, Portugal; Hospital de Santa Maria, Centro Hospitalar Lisboa Norte, 1649-035 Lisboa, Portugal; Health Research Unit & School of Health Sciences, Polytechnic institute of Leiria, 2411-901 Leiria, Portugal; Universidade Católica Portuguesa, Lisboa, 1649-023 Portugal; Unidade de Saúde Pública, Centro Hospitalar do Baixo Vouga, Aveiro, 3814-501 Portugal; Escola Superior de Saúde, Instituto Politécnico de Bragança, Bragança, 5300-253 Portugal; School of Health Sciences, Polytechnic institute of Leiria, 2411-901 Leiria, Portugal; Hospital de Santo André, Centro Hospitalar de Leiria, 2410-197 Leiria, Portugal; Escola Superior de Educação, Instituto Politécnico de Castelo Branco, 6000-767 Castelo Branco, Portugal; Escola Superior de Saúde Dr. Lopes Dias, Instituto Politécnico de Castelo Branco, 6000-767 Castelo Branco, Portugal; Centro Hospitalar do Porto, Porto, 4099-001 Portugal; Escola Superior de Enfermagem do Porto, Porto, 4200-072 Portugal; Center for Health Technology and Services Research, Faculty of Medicine, University of Porto, Porto, 4099-002 Portugal; School of Health Sciences, University of Aveiro, 3810-193 Aveiro, Portugal; Escola Superior de Saúde, Instituto Politécnico de Bragança, 5300-253 Bragança, Portugal; Centro Hospitalar Tâmega e Sousa, Penafiel, 4564-007 Portugal; Centro de Investigação em Desporto, Saúde e Desenvolvimento Humano, Escola Superior de Saúde, Instituto Politécnico de Bragança, Bragança, 5300-253 Portugal; Universidade Católica Portuguesa, Lisboa, 1649-023 Portugal; Hospital de Santa Maria, Centro Hospitalar Lisboa Norte, 1649-035 Lisboa, Portugal; Centro de Estudos em Educação, Tecnologias e Saúde, Escola Superior de Educação de Viseu, 3504-501 Viseu, Portugal; Universidade Católica Portuguesa – Porto, 4202-401 Porto, Portugal; Unidade Local de Saúde do Nordeste, Bragança, 5301-852 Portugal; Escola Superior de Saúde, Instituto Politécnico de Bragança, 5300-121 Bragança, Portugal; Universidad de León, 24004 León, España; Universidade do Sul de Santa Catarina, Palhoça, Santa Catarina 88137-270 Brasil; Instituto de Ciências da Saúde, Universidade Católica Portuguesa, Porto, 4202-401 Porto Portugal; Escola Superior de Enfermagem do Porto, 4200-072 Porto, Portugal; Instituto de Ciências da Saúde, Universidade Católica Portuguesa, Porto, 4202-401 Porto Portugal; Escola Superior de Enfermagem do Porto, 4200-072 Porto, Portugal; Research in Education and Community Intervention, Escola Superior de Saúde Jean Piaget, 4405-678 Gulpilhares, Vila Nova de Gaia Portugal; Escola Superior de Saúde, Instituto Politécnico de Santarém, Santarém, 2005-075 Santarém, Portugal; School of Health Sciences, Polytechnic Institute of Setúbal, 2910-761 Setúbal, Portugal; Universidade do Sul de Santa Catarina, Palhoça, Santa Catarina 88137-270 Brasil; Universidade Federal de Santa Catarina, Florianópolis, Santa Catarina 88040-900 Brasil; School of Health Sciences, Polytechnic Institute of Setúbal, 2910-761 Setúbal, Portugal; Instituto Politécnico de Castelo Branco, 6000-767 Castelo Branco, Portugal; Research in Education and Community Intervention, Instituto Piaget, 3515-776 Galifonge, Viseu Portugal; Universidad de Extremadura, 10003 Cáceres, España; Centro Universitário Franciscano, Santa Maria, Rio Grande do Sul 97010-032 Brasil; Universidade Federal de Santa Maria, Santa Maria, Rio Grande do Sul 97105-900 Brasil; Escola Superior de Enfermagem do Porto, 4200-072 Porto, Portugal; Escola Superior de Enfermagem, Universidade do Minho, Braga, 4710-057 Portugal; Escola Superior de Enfermagem do Porto, 4200-072 Porto, Portugal; Instituto Politécnico de Castelo Branco, 6000-767 Castelo Branco, Portugal; Universidad de Extremadura, 10003 Cáceres, España; Universidade Católica Portuguesa, Lisboa, 1649-023 Portugal; Hospital de Santa Maria, Centro Hospitalar Lisboa Norte, 1649-035 Lisboa, Portugal; Escola Superior de Educação, Instituto Politécnico de Castelo Branco, 6000-767 Castelo Branco, Portugal; Unidade de Transplante Hepático e Pancreático, Centro Hospitalar do Porto, 4099-001 Porto, Portugal; Escola Superior de Enfermagem do Porto, 4200-072 Porto, Portugal; Universidade Estadual Paulista ‘Júlio de Mesquita Filho”, 01049-010 São Paulo, Brazil; Universidade do Sul de Santa Catarina, Palhoça, Santa Catarina 88137-270 Brazil; Coimbra Chemistry Centre, Chemistry Department, University of Coimbra, 3004-535 Coimbra, Portugal; Health Research Unit & School of Health Sciences, Polytechnic institute of Leiria, 2411-901 Leiria, Portugal; Escola Superior de Tecnologia e Gestão, Instituto Politécnico de Leiria, 2411-901 Leiria, Portugal; MARE, Instituto Politécnico de Leiria, Peniche, 2520-641 Portugal; Escola Superior de Enfermagem de Coimbra, 3046-851 Coimbra, Portugal; Hospital Arcebispo João Crisóstomo, Cantanhede, 3060 Cantanhede Portugal; Hospital José Luciano de Castro, Anadia, 3780-226 Anadia Portugal; School of Health Sciences, University of Aveiro, Aveiro, 3810-193 Portugal; Hospital de Aveiro, Centro Hospitalar do Baixo Vouga E.P.E., Aveiro, 3814-501 Portugal; Escola Superior de Enfermagem do Porto, Porto, 4200-072 Portugal; Escola Nacional de Saúde Pública, Lisboa, 1600-560 Portugal; Instituto de Medicina Preventiva, Faculdade de Medicina de Lisboa, Universidade de Lisboa, 1649-028 Lisboa, Portugal; Faculty of Health Sciences, University of Beira Interior, Covilhã, 6200-506 Covilhã Portugal; Health Sciences Research Centre, University of Beira Interior, Covilhã, 6200-506 Covilhã Portugal; Centre for Neuroscience and Cell Biology, University of Coimbra, 3004-504 Coimbra, Portugal; Universidade do Vale do Paraíba, São José dos Campos, São Paulo 12244-000 Brasil; Centro de Estudos em Educação, Tecnologias e Saúde, Escola Superior de Saúde, Instituto Politécnico de Viseu, 3504-510 Viseu, Portugal; Maternidade Daniel de Matos, Centro Hospitalar e Universitário de Coimbra, 3000-075 Coimbra, Portugal; Hospital Dr. Nélio Mendonça, Região Autónoma da Madeira, Funchal, 9004-514 Portugal; Escola Superior de Saúde, Instituto Politécnico de Bragança, 5300-253 Bragança, Portugal; Escola Superior de Tecnologia e Gestão, Instituto Politécnico de Bragança, 5300-253 Bragança, Portugal; Universidade New Atlântica, 2745-615 Barcarena, Portugal; Universidade Federal de Santa Catarina, Florianópolis, 88040-900 Brasil; Universidade Federal do Amapá, Macapá, Amapá 68903-419 Brasil; Universidade Federal do Sergipe, São Cristóvão, Sergipe 49100-000 Brasil; Centro Hospitalar de Trás –os-Montes e Alto Douro, Vila Real, 5000-508 Portugal; Centro de estudos em educação, tecnologias e saúde, Escola Superior de Saúde, Instituto Politécnico de Viseu, 3504-510 Viseu, Portugal; Escola Superior de Enfermagem de Vila Real, Universidade de Trás –os-Montes e Alto Douro, Vila Real, 5000-232 Lordelo Portugal; Health Sciences Research Centre, Faculty of Health Sciences, University of Beira Interior, Covilhã, 6201-001 Portugal; Unidade de Investigação Aplicada em Gestão, Instituto Politécnico de Bragança, Bragança, 5300-253 Portugal; Research Unit in Business Sciences, University of Beira Interior, Covilhã, 6201-001 Portugal; Departamento de Ciências da Saúde, Universidade Católica Portuguesa, Viseu, 3504-505 Portugal; Centro de Investigação Interdisciplinar em Saúde, Universidade Católica Portuguesa, 3504-505 Viseu, Portugal; Unidade de Saúde Familiar Grão Vasco, Viseu, 3500-177 Portugal; Centro de estudos em educação, tecnologias e saúde, Instituto Politécnico de Viseu, 3504-510 Viseu, Portugal; Escola Superior de Tecnologia da Saúde do Porto, Instituto Politécnico do Porto, Vila Nova de Gaia, 4400-330 Vila Nova de Gaia, Portugal; Universidade Federal da Bahia, Salvador da Bahia, 0170-115 Brazil; Centro de Investigação e de Tecnologias Agro-Ambientais e Biológicas, Universidade de Trás-os-Montes e Alto Douro, 5001-801 Vila Real, Portugal & Universidade do Minho, 4704-553 Braga, Portugal; Universidade da Beira Interior, Covilhã, 6201-001 Covilhã, Portugal; Associação de Melhoramentos Pró Outeiro, Oliveira de Azeméis, 3720-514 Santiago de Riba-Ul Portugal; School of Education, Polytechnic Institute of Setúbal, 2910-761 Setúbal, Portugal; Centro Interdisciplinar da Performance Humana, Faculdade de Motricidade Humana, Universidade de Lisboa, 1499-002 Cruz Quebrada, Portugal; Research Centre Sports Sciences, Health Sciences and Human Development, University of Trás-os-Montes e Alto Douro, Vila Real, 5001-801 Vila Real, Portugal; Escola Superior de Saúde de Bragança, Instituto Politécnico de Bragança, 5300-121 Bragança, Portugal; Faculdade de Medicina, Universidade de Lisboa, 1649-028 Lisboa, Portugal; Escola Superior de Tecnologia da Saúde do Porto, Instituto Politécnico do Porto, Vila Nova de Gaia, 4400-330 Vila Nova de Gaia, Portugal; Centro de Investigação em Saúde e Ambiente, Instituto Politécnico do Porto, Vila Nova de Gaia, 4400-330 Vila Nova de Gaia, Portugal; Secção Autónoma das Ciências da Saúde, Universidade de Aveiro, Aveiro, 3810-193 Aveiro, Portugal; UCBIO-REQUIMTE Rede de Química e Tecnologia, 4051-401 Porto, Portugal; Departamento de Ciências Químicas, Faculdade de Farmácia, Universidade do Porto, Porto, 4050-313 Porto Portugal; Department of Legal Medicine and Forensic Sciences, Faculty of Medicine, University of Porto, Porto, 4200-319 Porto Portugal; Pharmaceutical Services, Centro Hospitalar de Vila Nova de Gaia/Espinho, EPE, Vila Nova de Gaia, 4400-129 Vila Nova de Gaia, Portugal; Escola Superior de Enfermagem de Coimbra, 3046-851 Coimbra, Portugal; University of Aveiro, Aveiro, 3810-193 Aveiro Portugal; Escola Superior de Tecnologia da Saúde do Porto, Instituto Politécnico do Porto, Vila Nova de Gaia, 4400-330 Vila Nova de Gaia Portugal; Research Centre for Sports Sciences, Health Sciences and Human Development, University of Trás-os-Montes e Alto Douro, Vila Real, 5001-801 Vila Real Portugal; Department of Sport Sciences, Exercise and Health, University of Trás-os-Montes e Alto Douro, Vila Real, 5001-801 Vila Real Portugal; Polytechnic Institute of Setúbal, 2910-761 Setúbal, Portugal; Centro de Estudos Sociais, Universidade de Coimbra, 3000-995 Coimbra, Portugal; Escola Superior de Enfermagem de Coimbra, 3046-851 Coimbra, Portugal; GyM Prevención, Burgos, 09007 Burgos, España; Universidad de Burgos, 09001 Burgos, España; Instituto de Medicina Preventiva e Saúde Pública, Faculdade de Medicina, Universidade de Lisboa, 1649-028 Lisboa, Portugal; Instituto de Saúde Ambiental, Faculdade de Medicina, Universidade de Lisboa, 1649-028 Lisboa, Portugal; Faculdade de Medicina da Universidade de Lisboa, Lisboa, 1649-028 Lisboa Portugal; Hospital Beatriz Ângelo, Loures, 2674-514 Loures Portugal; University of Extremadura, Badajoz, 06071 Badajoz España; Universidad Autonoma de Chile, Talca, 1670 Talca Chile; Universidade de Évora, 7004-516 Évora, Portugal; Escola Superior de Enfermagem de Coimbra, 3046-851 Coimbra, Portugal; Escola Superior de Tecnologia da Saúde, Instituto Politécnico do Porto, 4400-330 Vila Nova de Gaia, Portugal; Escola Superior de Saúde, Instituto Politécnico de Bragança, Bragança, 5300-121 Bragança Portugal; Unidade de Investigação UFP em Energia, Ambiente e Saúde & Centro de Estudos em Biomedicina, Fundação Fernando Pessoa, Porto, 4249-004 Porto Portugal; REQUIMTE/LAQV, Departamento de Ciências Químicas, Faculdade de Farmácia, Universidade do Porto, 4050-313 Porto, Portugal; Escola Superior de Enfermagem, Universidade do Minho, Braga, 4710-057 Braga Portugal; Universidade Católica Portuguesa, Porto, 4202-401 Porto Portugal; Escola Superior de Tecnologia da Saúde de Lisboa, Instituto Politécnico de Lisboa, 1549-020 Lisboa, Portugal; School of Health Sciences, Polytechnic institute of Leiria, 2411-901 Leiria, Portugal; Life Quality Research Centre, Polytechnic Institute of Santarém, 2001-904 Santarém, Portugal; Polytechnic Institute of Leiria, 2411-901 Leiria, Portugal; Centre for Rapid and Sustainable Product Development, Polytechnic Institute of Leiria, 2430-028 Marinha Grande, Portugal; Centro de Estudos Sociais, Universidade de Coimbra, 3000-995 Coimbra, Portugal; Escola superior de tecnologia da saúde de Lisboa, 1990-096 Lisboa, Portugal; Jönköping University Foundation, 551 11 Jönköping, Sweden; Centro de Estudos e Investigação em Saúde, Faculdade de Economia, Universidade de Coimbra, 3004-512 Coimbra, Portugal; Faculdade de Economia, Universidade de Coimbra, 3004-512 Coimbra, Portugal; ACES Pinhal Litoral, ARS Centro, Coimbra, 3001-553 Coimbra Portugal; Escola Superior de Desporto de Rio Maior, Instituto Politécnico de Santarém, 2040-413 Rio Maior, Portugal; Instituto Superior de Ciências Policiais e Segurança Interna, 1349-040 Lisboa, Portugal; Escola Superior de Tecnologia da Saúde de Coimbra, Instituto Politécnico de Coimbra, São Martinho do Bispo, 3046-854 Coimbra Portugal; Universidade Católica Portuguesa, Porto, 4202-401 Porto Portugal; Centro de Estudos em Educação, Tecnologias e Saúde, Escola Superior de Educação de Viseu, 3504-501 Viseu, Portugal; Escola Superior de Educação, Instituto Politécnico de Castelo Branco, 6000-767 Castelo Branco, Portugal; Research, Education and Community Intervention, Instituto Piaget, 3515-776 Galifonge, Viseu Portugal; Universidade Federal de Santa Catarina, Florianópolis, 88040-900 Brasil; Secretaria de estado da saúde de Santa Catarina, Florianópolis, Santa Catarina 88015-130 Brasil; Instituto Politécnico de Leiria, 2411-901 Leiria, Portugal; Escola Superior de Saúde, Instituto Politécnico de Viseu, 3504-510 Viseu, Portugal; Hospital Pêro da Covilhã, Centro Hospitalar Cova da Beira EPE, 6200-251 Covilhã, Portugal; Escola Superior de Tecnologia da Saúde do Porto, Instituto Politécnico do Porto, Vila Nova de Gaia, 4400-330 Vila Nova de Gaia Portugal; Centro de Investigação em Saúde e Ambiente, Instituto Politécnico do Porto, Vila Nova de Gaia, 4400-330 Vila Nova de Gaia Portugal; Serviços farmacêuticos, Hospital Pedro Hispano, Unidade Local de Saúde de Matosinhos, EPE, 4454 509 Senhora da Hora, Portugal; Department of Legal Medicine and Forensic Sciences, Faculty of Medicine, University of Porto, 4200-319 Porto, Portugal; Pharmaceutical Services, Centro Hospitalar de Vila Nova de Gaia/Espinho, EPE, Vila Nova de Gaia, 4400-129 Vila Nova de Gaia, Portugal; Faculdade Nobre, Feira de Santana, Bahia, 44001-008 Brasil; Secretaria de Saúde do Estado da Bahia, Salvador, Bahia, 41745-900 Brasil; Hospital Estadual da Criança, Vila Valqueire, Rio de Janeiro Brasil; Universidade Fernando Pessoa, Porto, 4249-004 Porto Portugal; Escola Superior de Tecnologia da Saúde, Instituto Politécnico do Porto, Vila Nova de Gaia, 4400-330 Vila Nova de Gaia Portugal; Instituto Português de Oncologia do Porto FG, EPE, Porto, 4200-072 Porto Portugal; Centro Hospitalar do Porto, Porto, 4099-001 Porto Portugal; Centro Hospitalar São João, Porto, 4200-319 Porto Portugal; Universidade de São Paulo, São Paulo, 05403-000 Brasil; Escola Superior de Tecnologia da Saúde de Coimbra, São Martinho do Bispo, 3046-854 Coimbra Portugal; VOID – Software Development, 2415-767 Leiria, Portugal; Centre for Research in Informatics and Communications, Polytechnic Institute of Leiria, 2411-901 Leiria, Portugal; Escola Superior de Tecnologia e Gestão, Instituto Politécnico de Leiria, 2411-901 Leiria, Portugal; Instituto de Engenharia de Sistemas e Computadores de Coimbra, 3000-033 Coimbra, Portugal; Escola Superior de Tecnologia e Gestão, Instituto Politécnico de Leiria, 2411-901 Leiria, Portugal; Instituto de Engenharia de Sistemas e Computadores de Coimbra, 3000-033 Coimbra, Portugal; Centre for Research in Informatics and Communications, Polytechnic Institute of Leiria, 2411-901 Leiria, Portugal; Nova Medical School, Universidade Nova de Lisboa, 1169-056 Lisboa, Portugal; Centro de Estudos de Doenças Crónicas, Universidade Nova de Lisboa, 1150-082 Lisboa, Portugal; Unidade de Investigação em Saúde, Escola Superior de Saúde de Leiria, 2411-901 Leiria, Portugal; Escola Superior de Tecnologia e Gestão, Instituto Politécnico de Leiria, 2411-901 Leiria, Portugal; School of Health Sciences, Polytechnic Institute of Leiria, 2411-901 Leiria, Portugal; Centro de estudos em educação, tecnologias e saúde, Escola Superior de Saúde, Instituto Politécnico de Viseu, 3504-510 Viseu, Portugal; Escola Nacional de Saúde Publica, Lisboa, 1600-560 Lisboa Portugal; Escola Superior de Saúde, Instituto Politécnico de Viseu, 3504-510 Viseu, Portugal; Escola Superior de Tecnologia e Saúde, Instituto Politécnico do Porto, 4400-330 Vila Nova de Gaia, Portugal; Escola Superior de Saúde, Instituto Politécnico de Viana do Castelo, 4900-347 Viana do Castelo, Portugal; Centro de Estudos em Educação, Tecnologias e Saúde, Escola Superior de Educação de Viseu, 3504-501 Viseu, Portugal; Universidade Católica Portuguesa, Porto, 4202-401 Porto Portugal; Escola Nacional de Saúde Pública, Lisboa, 1600-560 Lisboa Portugal; Escola Superior de Saúde, Instituto Politécnico de Viseu, 3504-510 Viseu, Portugal; Escola Superior de Saúde, Instituto Politécnico de Portalegre, Portalegre, 7301-901 Portalegre Portugal; Faculdade de Medicina Dentária, Universidade de Lisboa, Lisboa, 1649-003 Lisboa Portugal; Escola Superior de Saúde, Instituto Politécnico de Leiria, 2411-901 Leiria, Portugal; Department for Health Psychology, FernUniversität in Hagen, 58084 Hagen, Germany; Department of Biomedical Sciences, University of Applied Sciences Salzburg, A-5412 Puch, Salzburg Austria; Department of Medical and Bioinformatics, University of Applied Sciences Upper Austria, Hagenberg, A-4232 Hagenberg Austria; Escola Superior de Tecnologia da Saúde de Coimbra, São Martinho do Bispo, 3046-854 Coimbra, Portugal; Escola Superior de Tecnologia da Saúde de Coimbra, São Martinho do Bispo, 3046-854 Coimbra, Portugal; Universidad de Extremadura, 10003 Cáceres, España; Faculdade de Psicologia e Ciências da Educação, Universidade de Coimbra, 3001-802 Coimbra, Portugal; Escola Superior de Enfermagem de Coimbra, Coimbra, 3046-851 Portugal; Faculdade de Psicologia e Ciências da Educação, Universidade de Coimbra, 3001-802 Coimbra, Portugal; Escola Superior de Enfermagem de Coimbra, Coimbra, 3046-851 Portugal; Escola Superior de Enfermagem do Porto, 4200-072 Porto, Portugal; Center for Health Technology and Services Research, Faculty of Medicine, University of Porto, 4099-002 Porto, Portugal; Centro Hospitalar de Trás–os-Montes e Alto Douro, Vila Real, 5000-508 Portugal; Centro de estudos em educação, tecnologias e saúde, Escola Superior de Saúde, Instituto Politécnico de Viseu, 3504-510 Viseu, Portugal; Polytechnic Institute of Viseu, Viseu, 3504-510 Portugal; University of Trás-os-Montes e Alto Douro, Vila Real, 5001-801 Portugal; Research Centre Sports Sciences, Health Sciences and Human Development, University of Trás-os-Montes e Alto Douro, Vila Real, 5001-801 Portugal; Unidade de Saúde Pública, ACES Douro I, Marão e Douro Norte, Administração Regional de Saúde do Norte, 5000-524 Vila Real, Portugal; Centro de Investigação em Saúde Pública, Escola Nacional de Saúde Publica, 1600-560 Lisboa, Portugal; Centro de estudos em educação, tecnologias e saúde, Escola Superior de Saúde, Instituto Politécnico de Viseu, 3504-510 Viseu, Portugal; Hospital Prof. Doutor Fernando Fonseca E.P.E., Amadora, 2720-276 Portugal; Escola Superior de Saúde de Leiria eCADR& Centro de Investigação Didática e Tecnologia na Formação de Formadores, Universidade de Aveiro, 3810-193 Aveiro, Portugal; Unidade de Investigação Inclusão e Acessibilidade em Acção “iACT” e Escola Superior Educação e Ciências Sociais, Instituto Politécnico de Leiria, 2411-901 Leiria, Portugal; Instituto Politécnico de Leiria, 2411-901 Leiria, Portugal; Unidade de Saúde Pública, Centro Hospitalar do Baixo Vouga, Aveiro, 3814-501 Portugal; Health Research Unit & School of Health Sciences, Polytechnic institute of Leiria, 2411-901 Leiria, Portugal; Health Research Unit & School of Health Sciences, Polytechnic institute of Leiria, 2411-901 Leiria, Portugal; Health Research Unit & School of Health Sciences, Polytechnic institute of Leiria, 2411-901 Leiria, Portugal; Unidade de Saúde Pública - ACES Pinhal Litoral, ARS Centro, 3100-462 Pombal, Portugal; Consulta Externa de Pediatria, Centro Hospitalar de Leiria, 2410-197 Leiria, Portugal; Health Research Unit & School of Health Sciences, Polytechnic institute of Leiria, 2411-901 Leiria, Portugal; Escola Superior de Enfermagem de Coimbra, 3046-851 Coimbra, Portugal; Escola Superior Escola Superior de Enfermagem do Porto, 4200-072 Porto, Portugal; Health Research Unit & School of Health Sciences, Polytechnic institute of Leiria, 2411-901 Leiria, Portugal; Universidade do Oeste do Paraná, Foz do Iguaçu, Paraná 85851-100 Brasil; Centro de Investigação Interdisciplinar em Saúde, Universidade Católica Portuguesa/Porto, 4202-401 Porto, Portugal; Instituto de Investigação e Formação Avançada em Ciências e Tecnologias da Saúde, Cooperativa De Ensino Superior Politécnico Universitário, Instituto Politécnico de Saúde do Norte, 4585-116 Gandra, Portugal; Escola Superior de Enfermagem do Porto, Porto, 4200-072 Porto, Portugal; Centre for Health Technology and Services Research, Faculdade de Medicina da Universidade do Porto, 4200-450 Porto, Portugal; Escola Superior de Tecnologia da Saúde de Coimbra, São Martinho do Bispo, 3046-854 Coimbra, Portugal; University Thomas More, B - 2800 Mechelen, Belgium; Escola Superior de Tecnologia da Saúde de Coimbra, São Martinho do Bispo, 3046-854 Coimbra, Portugal; Instituto Português do Sangue e da Transplantação, Lisboa, 1000-208 Portugal; Escola Superior de Enfermagem, Universidade do Minho, Braga, 4710-057 Portugal; Facultad de Terapia Ocupacional, Logopedia y Enfermería, Universidad de Castilla-La Mancha, 45600 Talavera de la Reina, Toledo España; Universidade de Trás-os-Montes e Alto Douro, Vila Real, 5001-801 Portugal; Centro Hospitalar de Trás-os-Montes e Alto Douro, Vila Real, 5000-508 Portugal; Universidade de São Paulo, São Paulo, 05403-000 Brasil; Faculdade de Ciências do Desporto e Educação Física, Universidade de Coimbra, 3040-248 Coimbra, Portugal; Faculdade de Ciências do Desporto e Educação Física, Universidade de Coimbra, 3040-248 Coimbra, Portugal

## Abstract

S1 Health literacy and health education in adolescence

Catarina Cardoso Tomás

S2 The effect of a walking program on the quality of life and well-being of people with schizophrenia

Emanuel Oliveira, D. Sousa, M. Uba-Chupel, G. Furtado, C. Rocha, A. Teixeira, P. Ferreira

S3 Diagnosis and innovative treatments - the way to a better medical practice

Celeste Alves

S4 Simulation-based learning and how it is a high contribution

Stefan Gisin

S5 Formative research about acceptability, utilization and promotion of a home fortification programme with micronutrient powders (MNP) in the Autonomous Region of Príncipe, São Tomé and Príncipe

Elisabete Catarino, Nelma Carvalho, Tiago Coucelo, Luís Bonfim, Carina Silva

S6 Safety culture of the patient: a reflexion about the therapeutic approach on the patient with vocal pathology

Débora Franco

S7 About wine, fortune cookies and patient experience

Jesús Alcoba González

O1 The psychological impact on the emergency crews after the disaster event on February 20, 2010

Helena G. Jardim, Rita Silva

O2 Musculoskeletal disorders in midwives

Cristina L. Baixinho, Mª Helena Presado, Mª Fátima Marques, Mário E. Cardoso

O3 Negative childhood experiences and fears of compassion: Implications for psychological difficulties in adolescence

Marina Cunha, Joana Mendes, Ana Xavier, Ana Galhardo, Margarida Couto

O4 Optimal age to give the first dose of measles vaccine in Portugal

João G. Frade, Carla Nunes, João R. Mesquita, Maria S. Nascimento, Guilherme Gonçalves

O5 Functional assessment of elderly in primary care

Conceição Castro, Alice Mártires, Mª João Monteiro, Conceição Rainho

O6 Smoking and coronary events in a population of Spanish health-care centre: An observational study

Francisco P. Caballero, Fatima M. Monago, Jose T. Guerrero, Rocio M. Monago, Africa P. Trigo, Milagros L. Gutierrez, Gemma M. Milanés, Mercedes G. Reina, Ana G. Villanueva, Ana S. Piñero, Isabel R. Aliseda, Francisco B. Ramirez

O7 Prevalence of musculoskeletal injuries in Portuguese musicians

Andrea Ribeiro, Ana Quelhas, Conceição Manso

O8 Hip fractures, psychotropic drug consumption and comorbidity in patients of a primary care practice in Spain

Francisco P. Caballero, Jose T. Guerrero, Fatima M. Monago, Rafael B. Santos, Nuria R. Jimenez, Cristina G. Nuñez, Inmaculada R. Gomez, Mª Jose L. Fernandez, Laura A. Marquez, Ana L. Moreno, Mª Jesus Tena Huertas, Francisco B. Ramirez

O9 The role of self-criticism and shame in social anxiety in a clinical SAD sample

Daniel Seabra, Mª Céu Salvador

O10 Obstruction and infiltration: a proposal of a quality indicator

Luciene Braga, Pedro Parreira, Anabela Salgueiro-Oliveira, Cristina Arreguy-Sena, Bibiana F. Oliveira, Mª Adriana Henriques

O11 Balance and anxiety and depression symptoms in old age people

Joana Santos, Sara Lebre, Alda Marques

O12 Prevalence of postural changes and risk factors in school children and adolescents in a northern region (Porto)

Clarinda Festas, Sandra Rodrigues, Andrea Ribeiro, José Lumini

O13 Ischemic stroke vs. haemorrhagic stroke survival rate

Ana G. Figueiredo

O14 Chronobiological factors as responsible for the appearance of locomotor pathology in adolescents

Francisco J. Hernandez-Martinez, Liliana Campi, Mª Pino Quintana-Montesdeoca, Juan F. Jimenez-Diaz, Bienvenida C. Rodriguez-De-Vera

O15 Risk of malnutrition in the elderly of Bragança

Alexandra Parente, Mª Augusta Mata, Ana Mª Pereira, Adília Fernandes, Manuel Brás

O16 A Lifestyle Educational Programme for primary care diabetic patients: the design of a complex nursing intervention

Mª Rosário Pinto, Pedro Parreira, Marta L. Basto, Ana C. Rei, Lisete M. Mónico

O17 Medication adherence in elderly people

Gilberta Sousa, Clementina Morna, Otília Freitas, Gregório Freitas, Ana Jardim, Rita Vasconcelos

O18 Hospitalization for cervical cancer of residents in the metropolitan region of Porto Alegre, Southern Brazil, 2012 to 2014

Lina G. Horta, Roger S. Rosa, Luís F. Kranz, Rita C. Nugem, Mariana S. Siqueira, Ronaldo Bordin

O19 Oncologic assistance of high complexity: evaluation of regulating accesses

Rosiane Kniess, Josimari T. Lacerda

O20 Perceived barriers for using health care services by the older population as seen by the social sector: findings from the Vila Nova de Gaia Gerontological Plan

Joana Guedes, Idalina Machado, Sidalina Almeida, Adriano Zilhão, Helder Alves, Óscar Ribeiro

O21 Sleep difficulties and depressive symptoms in college students

Ana P. Amaral, Ana Santos, Joana Monteiro, Mª Clara Rocha, Rui Cruz

O22 Psychopathological symptoms and medication use in higher education

Ana P. Amaral, Marina Lourenço, Mª Clara Rocha, Rui Cruz

O23 Sexually transmitted diseases in higher education institutions

Sandra Antunes, Verónica Mendonça, Isabel Andrade, Nádia Osório, Ana Valado, Armando Caseiro, António Gabriel, Anabela C. Martins, Fernando Mendes

O24 Alcohol consumption and suicide ideation in higher education students

Lídia Cabral, Manuela Ferreira, Amadeu Gonçalves

O25 Quality of life in university students

Tatiana D. Luz, Leonardo Luz, Raul Martins

O26 Male and female adolescent antisocial behaviour: characterizing vulnerabilities in a Portuguese sample

Alice Morgado, Maria L. Vale-Dias

O27 Risk factors for mental health in higher education students of health sciences

Rui Porta-Nova

O28 International classification of functioning disability and health as reflexive reasoning in primary attention in health

Tânia C. Fleig, Éboni M. Reuter, Miriam B. Froemming, Sabrina L. Guerreiro, Lisiane L. Carvalho

O29 Risk factors and cardiovascular disease in Portalegre

Daniel Guedelha, P. Coelho, A. Pereira

O30 Health status of the elderly population living in Portalegre historic city centre: A longitudinal study

António Calha, Raul Cordeiro

O31 Student’s sleep in higher education: sleep quality among students of the IPB

Ana Gonçalves, Ana Certo, Ana Galvão, Mª Augusta Mata

O32 Trend in mortality from cervical cancer in the metropolitan area of Florianópolis, state of Santa Catarina, Brazil, 2000 to 2013

Aline Welter, Elayne Pereira, Sandra Ribeiro, Marcia Kretzer

O33 Adherence to treatment in the elderly in an urban environment in Spain

Juan-Fernando Jiménez-Díaz, Carla Jiménez-Rodríguez, Francisco-José Hernández-Martínez, Bienvenida-Del-Carmen Rodríguez-De-Vera, Alexandre Marques-Rodrigues

O34 Beira Baixa Blood Pressure Study (Study PABB)

Patrícia Coelho, Tiago Bernardes, Alexandre Pereira

O35 Trends in cervical cancer mortality statistics in Santa Catarina State, Brazil, by age group and macro-region, from 2000 to 2013

Patrícia Sousa, João G. Filho, Nazare Nazario, Marcia Kretzer

O36 Sleep problems among Portuguese adolescents: a public health issue

Odete Amaral, António Garrido, Nélio Veiga, Carla Nunes, Ana R. Pedro, Carlos Pereira

O37 Association between body fat and health-related quality of life in patients with type 2 diabetes

António Almeia, Helder M. Fernandes, Carlos Vasconcelos, Nelson Sousa, Victor M. Reis, M. João Monteiro, Romeu Mendes

O38 Therapy adherence and polypharmacy in non-institutionalized elderly from Amares county, Portugal

Isabel C. Pinto, Tânia Pires, João Gama

O39 Prevalence of surgical site infection in adults at a hospital unit in the North of Portugal

Vera Preto, Norberto Silva, Carlos Magalhães, Matilde Martins

O40 Frailty phenotype in old age: implications to intervention

Mafalda Duarte, Constança Paúl, Ignácio Martín

O41 Portuguese women: sexual symptoms in perimenopause

Arminda A. Pinheiro

O42 Predictive ability of the Perinatal Depression Screening and Prevention Tool – preliminary results of the categorical approach

Sandra Xavier, Julieta Azevedo, Elisabete Bento, Cristiana Marques, Mariana Marques, António Macedo, Ana T. Pereira

O43 Aging and muscle strength in patients with type 2 diabetes: cross sectional analysis

José P. Almeida, António Almeida, Josiane Alves, Nelson Sousa, Francisco Saavedra, Romeu Mendes

O44 Accessibility of the elderly in the prevention of hypertension in a family health unit

Ana S. Maia, Michelle T. Oliveira, Anderson R. Sousa, Paulo P. Ferreira, Luci S. Lopes, Eujcely C. Santiago

O45 Community Health screenings and self-reported chronic diseases

Sílvia Monteiro, Ângelo Jesus, Armanda Colaço, António Carvalho, Rita P. Silva, Agostinho Cruz

O46 Evaluation of indoor air quality in Kindergartens

Ana Ferreira, Catarina Marques, João P. Figueiredo, Susana Paixão

O47 Atmospheric exposure to chemical agents under the occupational activity of pathology technicians

Ana Ferreira, Carla Lopes, Fernando Moreira, João P. Figueiredo

O48 Occupational exposure to air pollutants in night entertainment venues workers

Ana Ferreira, Diana Ribeiro, Fernando Moreira, João P. Figueiredo, Susana Paixão

O49 Beliefs and attitudes of young people towards breastfeeding

Telma Fernandes, Diogo Amado, Jéssica Leal, Marcelo Azevedo, Sónia Ramalho

O50 Profiling informal caregivers: surveying needs in the care of the elderly

Catarina Mangas, Jaime Ribeiro, Rita Gonçalves

O51 Visual health in teenagers

Amélia F Nunes, Ana R. Tuna, Carlos R. Martins, Henriqueta D. Forte

O52 Amenable mortality and the geographic accessibility to healthcare in Portugal

Cláudia Costa, José A. Tenedório, Paula Santana

O53 Bacterial contamination of door handles in a São Paulo See Metropolitan Cathedral public restrooms in Brazil

J. A. Andrade, J. L. Pinto, C. Campofiorito, S. Nunes, A. Carmo, A. Kaliniczenco, B. Alves, F. Mendes, C. Jesus, F. Fonseca, F. Gehrke

O54 Adherence of patients to rehabilitation programmes

Carlos Albuquerque, Rita Batista, Madalena Cunha, António Madureira, Olivério Ribeiro, Rosa Martins

O55 Prevalence of malnutrition among Portuguese elderly living in nursing homes: preliminary results of the PEN-3S project

Teresa Madeira, Catarina Peixoto-Plácido, Nuno Santos, Osvaldo Santos, Astrid Bergland, Asta Bye, Carla Lopes, Violeta Alarcão, Beatriz Goulão, Nuno Mendonça, Paulo Nicola, João G. Clara

O56 Relation between emotional intelligence and mental illness in health students

João Gomes, Ana Querido, Catarina Tomás, Daniel Carvalho, Marina Cordeiro

P1 Fall risk factors in people older than 50 years old – a pilot report

Marlene C. Rosa, Alda Marques

P2 What about the Portuguese oldest old? A global overview using census data

Daniela Brandão, Óscar Ribeiro, Lia Araújo, Constança Paúl

P3 Prevalence of injuries in senior amateur volleyball athletes in Alentejo and Algarve clubs, Portugal: factors associated

Beatriz Minghelli, Sylvina Richaud

P4 Shame feelings and quality of life: the role of acceptance and decentring

Ana L. Mendes, Joana Marta-Simões, Inês A. Trindade, Cláudia Ferreira

P5 Assessment of social support during deployment in portuguese colonial war veterans

Teresa Carvalho, Marina Cunha, José Pinto-Gouveia

P6 Hospitalization for acute viral bronchiolitis of residents in the metropolitan region of Porto Alegre, Southern Brazil, 2012 to 2014

Morgana C. Fernandes, Roger S. Rosa, Rita C. Nugem, Luís F. Kranz, Mariana S. Siqueira, Ronaldo Bordin

P7 Falls-risk screening – an opportunity for preventing falls in the elderly from Nordeste

Anabela C. Martins, Anabela Medeiros, Rafaela Pimentel, Andreia Fernandes, Carlos Mendonça, Isabel Andrade, Susana Andrade, Ruth L. Menezes

P8 Aging provokes chronodisruption in mature people in temperature circadian rhythm

Rafael Bravo, Marta Miranda, Lierni Ugartemendia, José Mª Tena, Francisco L. Pérez-Caballero, Lorena Fuentes-Broto, Ana B. Rodríguez, Barriga Carmen

P9 The influence of climate and pollution factors in dengue cases of great ABC region, São Paulo

M. A. Carneiro, J. N. Domingues, S. Paixão, J. Figueiredo, V. B. Nascimento, C. Jesus, F Mendes, F. Gehrke, B. Alves, L. Azzalis, F. Fonseca

P10 Visual function and impact of visual therapy in children with learning disabilities: a pilot study

Ana R. Martins, Amélia Nunes, Arminda Jorge

P11 Edentulism and the need of oral rehabilitation among institutionalized elderly

Nélio Veiga, Ana Amorim, André Silva, Liliana Martinho, Luís Monteiro, Rafael Silva, Carina Coelho, Odete Amaral, Inês Coelho, Carlos Pereira, André Correia

P12 Therapy adherence of outpatients in the pharmacy services of a hospital unit

Diana Rodrigues, Nídia Marante, Pedro Silva, Sara Carvalho, André Rts Araujo, Maximiano Ribeiro, Paula Coutinho, Sandra Ventura, Fátima Roque

P13 Universal access and comprehensive care of oral health: an availability study

Cristina Calvo, Manoela Reses

P14 Is the respiratory function of children a predictor of air quality? Coimbra as a case study

Jorge Conde, Ana Ferreira, João Figueiredo

P15 Meaning-in-life of college students

David Silva, Luís Seiça, Raquel Soares, Ricardo Mourão, Teresa Kraus

O57 Training needs for nurses in palliative care

Ana C. Abreu, José M. Padilha, Júlia M. Alves

O58 Impact of computerized information systems in the global nurses’ workload: nurses’ perceptions and real-time

Paulino Sousa, Manuel Oliveira, Joana Sousa

O59 The perspective of health care professionals on self-care in hereditary neurodegenerative disease: a qualitative study

Sónia Novais, Felismina Mendes

O60 Contribution for health-related physical fitness reference values in healthy adolescents

Joana Pinto, Joana Cruz, Alda Marques

School of Health Sciences, University of Aveiro, 3810-193 Aveiro, Portugal

O61 Perception of learning, satisfaction and self-efficacy of nursing students about High-Fidelity Simulation

Hugo Duarte, Maria Dos Anjos Dixe, Pedro Sousa

O62 Analysis of statements of diagnosis about health deviation in self-care requisites customized in a Nursing Practice Support System (SAPE®): Management of therapeutic regimen

Inês Cruz, Fernanda Bastos, Filipe Pereira

O63 Hybrid management and hospital governance: doctors and nurses as managers

Francisco L. Carvalho, Teresa T. Oliveira, Vítor R. Raposo

O64 Time management in health professionals

Conceição Rainho, José C. Ribeiro, Isabel Barroso, Vítor Rodrigues

O65 Financial rewards and wellbeing in primary health care

Carmo Neves, Teresa C. Oliveira

O66 Patient safety promotion in the operating room

Bárbara Oliveira, Mª Carminda Morais, Pilar Baylina

O67 Difficulties and needs of pre-graduate nursing students in the area of Geriatrics/Gerontology

Rogério Rodrigues, Zaida Azeredo, Corália Vicente

O68 Teaching and learning sexuality in nursing education

Hélia Dias, Margarida Sim-Sim

O69 Entrepreneurial Motivations Questionnaire: AFC and CFA in academy

Pedro Parreira, Anabela Salgueiro-Oliveira, Amélia Castilho, Rosa Melo, João Graveto, José Gomes, Marina Vaquinhas, Carla Carvalho, Lisete Mónico, Nuno Brito

O70 Nursing intervention to patient with Permanent Pacemakers and Implantable Cardioverter Defibrillators: a qualitative analysis

Cassilda Sarroeira, José Amendoeira, Fátima Cunha, Anabela Cândido, Patrícia Fernandes, Helena R. Silva, Elsa Silva

O71 Alcohol consumption among nursing students: where does education fail?

Isabel Barroso, Leila Lapa, Cristina Antunes

O72 Labour stress in nursing

Ana Gonçalves, Ana Galvão, Mª José Gomes, Susana R. Escanciano

O73 The influence of safe staff nursing in patient satisfaction with nursing care

Maria Freitas, Pedro Parreira, João Marôco

O74 Intention to use eHealth strategies with nursing students

Ana R. Fernandes, Cremilde Cabral, Samuel Alves, Pedro Sousa

O75 Community Based Mental Health: contributions of an interdisciplinary international program for students in higher health education

António Ferreira, Fernanda Príncipe, Ulla-Maija Seppänen, Margarida Ferreira, Maribel Carvalhais, Marilene Silva

O76 Study of satisfaction at work of graduates in nursing: 2002-2014

Manuela Ferreira, Joana Silva, Jéssica Neves, Diana Costa, Bruno Santos, Soraia Duarte

O77 Health professionals’ attitudes towards breastfeeding

Sílvia Marques, Sónia Ramalho, Isabel Mendes

O78 Continuity of nursing care to person with type 2 diabetes

Clarisse Louro, Eva Menino, Maria Dixe, Sara S. Dias

O79 Stigma toward mental illness among future health professionals

Marina Cordeiro, Catarina Tomás, Ana Querido, Daniel Carvalho, João Gomes

O80 Working with fears and anxieties of medical students in search of a humanized care

Frederico C. Valim, Joyce O. Costa, Lúcia G. Bernardes

P16 Surgical paediatrics patients’ psycho prophylaxis at a teaching hospital

Helena Prebianchi

P17 Patient-perceived outcomes in physiotherapy – a pilot study

Marlene Cristina Rosa

P18 Building competencies for managers in nursing

Narcisa Gonçalves, Maria M. Martins, Paulina Kurcgant

P19 Theoretical basis underlying physiotherapy practice in stroke rehabilitation

André Vieira

P20 When the life-cycle ends: the nurse’s confrontation with death

Sandrina Bento, Sérgio Deodato, Isabel Rabiais

P21 Nursing students’ opinion about the supervision relationship during their first clinical experience

Laura Reis

P22 Nursing Relational Laboratory: Pedagogical, dialogic and critical project

Ana Torres, Sérgio Soares, Margarida Ferreira, Pedro Graça

P23 Job satisfaction of bioscientists at a Lisbon hospital

Céu Leitão, Renato Abreu, Fernando Bellém, Ana Almeida, Edna Ribeiro-Varandas, Ana Tavares

P24 Sociodemographic and professional profile of nurses and its relation with the importance of family in nursing practices

João G. Frade, Carolina Henriques, Eva Menino, Clarisse Louro, Célia Jordão

P25 Professional satisfaction of rehabilitation nurses

Sofia Neco, Carminda Morais, Pedro Ferreira

P26 The person living with a stoma: the formalization of knowledge in nursing

Carla R. Silva, Alice Brito, Antónia Silva

P27 Validation of the Portuguese versions of the nursing students’ perceptions of learning and learner satisfaction with simulation tool

Hugo Duarte, Maria Dos Anjos Dixe, Pedro Sousa

P28 Physiotherapists’ perceived knowledge on technologies for electronic health records for physiotherapy

Gabriela Postolache, Raul Oliveira, Isabel Moreira, Luísa Pedro, Sónia Vicente, Samuel Domingos, Octavian Postolache

P29 Quality of life and physical activity of medicine undergraduate students in the University of Southern Santa Catarina, Brazil

Darlen Silva, João G. Filho, Nazare Nazario, Marcia Kretzer, Dulcineia Schneider

P30 The curricular skills for decision making education in a Nursing Degree

Fátima M. Marques

P31 Effect of nurses’ mobilization in satisfaction at work and turnover: An empirical study in the hospital setting

Pedro Parreira, Carla Carvalho, Lisete M. Mónico, Carlos Pinto, Sara Vicente, São João Breda

P32 Entrepreneurial skills of students of polytechnic higher education in Portugal: Business influences

José H. Gomes, Rosa Melo, Pedro Parreira, Anabela Salgueiro, João Graveto, Marina Vaquinhas, Amélia Castilho

P33 Design and assessment of e-learning modules for Pharmacology

Ângelo Jesus, Nuno Duarte, José C. Lopes, Hélder Nunes, Agostinho Cruz

P34 Perspective of nurses involved in an action-research study on the changes observed in care provision: results from a focus group

Anabela Salgueiro-Oliveira, Pedro Parreira, Marta L. Basto, Luciene M. Braga

P35 Use of peer feedback by nursing students during clinical training: teacher’s perception

António Ferreira, Beatriz Araújo, José M. Alves, Margarida Ferreira, Maribel Carvalhais, Marilene Silva, Sónia Novais

P36 What’s new on endotracheal suctioning recommendations

Ana S. Sousa, Cândida Ferrito

P37 Assessment of the nurses satisfaction on the Central Region of Portugal

Pedro L. Ferreira, Alexandre Rodrigues, Margarida Ferreira, Isabel Oliveira

P38 Study of graduate’s satisfaction with the school of nursing

Manuela Ferreira, Jéssica Neves, Diana Costa, Soraia Duarte, Joana Silva, Bruno Santos

P39 Partnership between the school of nursing and the hospital: Supervisors´ perspectives

Cristina Martins, Ana P. Macedo, Odete Araújo, Cláudia Augusto, Fátima Braga, Lisa Gomes, Maria A. Silva, Rafaela Rosário

P40 Coping strategies of college students

Luís Pimenta, Diana Carreira, Patrícia Teles, Teresa Barros

P41 Emotional intelligence and mental health stigma in health students

Catarina Tomás, Ana Querido, Daniel Carvalho, João Gomes, Marina Cordeiro

P42 Stigma of mental health assessment: Comparison between health courses

Daniel Carvalho, Ana Querido, Catarina Tomás, João Gomes, Marina Cordeiro

O81 Short- and long-term effects of pulmonary rehabilitation in mild COPD

Cristina Jácome, Alda Marques

O82 Phonological awareness programme for preschool children

Sylvie Capelas, Andreia Hall, Dina Alves, Marisa Lousada

O83 REforma ATIVA: An efficient health promotion program to be implemented during retirement

Mª Helena Loureiro, Ana Camarneiro, Margarida Silva, Aida Mendes, Ana Pedreiro

O84 Intervention for men who batter women, a case report

Anne G.Silva, Elza S. Coelho

O85 Immediate effects of Bowen Therapy on muscle tone and flexibility

Flávio Melo, Fernando Ribeiro, Rui Torres, Rui Costa

O86 Predictive equation for incremental shuttle walk test in adolescents

Tânia Pinho, Cristina Jácome, Alda Marques

O87 Life satisfaction and psychopathology in institutionalized elderly people: The results of an adapted Mindfulness-Based Stress Reduction program

Bárbara Cruz, Daniel Seabra, Diogo Carreira, Maria Ventura

O88 Outcome changes in COPD rehabilitation: exploring the relationship between physical activity and health-related outcomes

Joana Cruz, Dina Brooks, Alda Marques

O89 Assessing the effectiveness of a Complex Nursing Intervention

M Rosário Pinto, Pedro Parreira, Marta Lima-Basto, Miguel Neves, Lisete M. Mónico

O90 Psychotherapeutic intervention in addiction disorders: Change in psychopathological symptoms and emotional states

Carla Bizarro, Marina Cunha, Ana Galhardo, Couto Margarida, Ana P. Amorim, Eduardo Silva

O91 Economic impact of a nursing intervention program to promote self-management in COPD

Susana Cruz, José M. Padilha, Jorge Valente

O92 Multimodal acute pain management during uterine artery embolization in treatment of uterine myomas

José T. Guerrero, Francisco P. Caballero, Rafael B. Santos, Estefania P. Gonzalez, Fátima M. Monago, Lierni U. Ugalde, Marta M. Vélez, Maria J. Tena

O93 Fluid administration strategies in major surgery: Goal-directed therapy

José T. Guerrero, Rafael Bravo, Francisco L. Pérez-Caballero, Isabel A. Becerra, Mª Elizabeth Agudelo, Guadalupe Acedo, Roberto Bajo

O94 Development and implementation of a self-management educational programme using lay-led’s in adolescents Spina Bifida: A pilot study

Isabel Malheiro, Filomena Gaspar, Luísa Barros

O95 Influence of chair-based yoga exercises on salivary anti-microbial proteins in institutionalized frail-elderly women: a preliminary study

Guilherme Furtado, Mateus Uba-Chupel, Mariana Marques, Luís Rama, Margarida Braga, José P. Ferreira, Ana Mª Teixeira

O96 High intensity interval training vs moderate intensity continuous training impact on diabetes 2

João Cruz, Tiago Barbosa, Ângela Simões, Luís Coelho

O97 Family caregiver of people with pressure ulcer: Nursing intervention plan

Alexandre Rodrigues, Juan-Fernando Jiménez-Díaz, Francisco Martinez-Hernández, Bienvenida Rodriguez-De-Vera, Pedro Ferreira, Alexandrina Rodrigues

O98 Chronic effects of exercise on motor memory consolidation in elderly people

André Ramalho, João Petrica, Pedro Mendes, João Serrano, Inês Santo, António Rosado

O99 Impression cytology of the ocular surface: Collection technique and sample processing

Paula Mendonça, Kátia Freitas

O100 Does sport practice affect the reaction time in neuromuscular activity?

Dora Ferreira, António Brito, Renato Fernandes

O101 Efficiency of the enteral administration of fibbers in the treatment of chronic obstipation

Sofia Gomes, Fernando Moreira, Cláudia Pinho, Rita Oliveira, Ana I. Oliveira

O102 Fast decalcifier in compact bone and spongy bone

Paula Mendonça, Ana P. Casimiro, Patrícia Martins, Iryna Silva

O103 Health promotion in the elderly – Intervention project in dementia

Diana Evangelista

O104 Prevention of musculoskeletal disorders through an exercise protocol held in labour context

Catarina Leitão, Fábia Velosa, Nélio Carecho, Luís Coelho

O105 Knowledge of teachers and other education agents on diabetes type 1: Effectiveness of an intervention program

Eva Menino, Anjos Dixe, Helena Catarino, Fátima Soares, Ester Gama, Clementina Gordo

O106 Treatment of diabetic peripheral neuropathic pain: a systematic review of clinical trials of phase II and III

Eliana Moreira, Cristiana Midões, Marlene Santos

O107 New drugs for osteoporosis treatment: Systematic review of clinical trials of phase II and III

Sara Machado, Vânia P. Oliveira, Marlene Santos

O108 Promoting hope at the end of life: Effectiveness of an Intervention Programme

Ana Querido, Anjos Dixe, Rita Marques, Zaida Charepe

P43 Psychomotor therapy effects on adaptive behaviour and motor proficiency of adults with intellectual disability

Ana Antunes, Sofia Santos

P44 The effect of exercise therapy in multiple sclerosis – a single study case

Marlene C. Rosa

P45 Physical condition and self-efficacy in people with fall risk – a preliminary study

Marlene C. Rosa, Silvana F. Marques

P46 Shock waves: their effectiveness in improving the symptoms of calcifying tendinitis of the shoulder

Beatriz Minghelli, Eulália Caro

P47 Pacifier – construction and pilot application of a parenting intervention for parents of babies until six months in primary health care

Mª José Luís, Teresa Brandão

P48 The influence of Motor Imagery in fine motor skills of individuals with disabilities

Pedro Mendes, Daniel Marinho, João Petrica, Diogo Monteiro, Rui Paulo, João Serrano, Inês Santo

P49 Evaluation of the effects of a walking programme on the fall risk factors in older people – a longitudinal pilot study

Lina Monteiro, Fátima Ramalho, Rita Santos-Rocha, Sónia Morgado, Teresa Bento

P50 Nursing intervention programme in lifestyles of adolescents

Gilberta Sousa, Otília Freitas, Isabel Silva, Gregório Freitas, Clementina Morna, Rita Vasconcelos

P51 The person submitted to hip replacement rehabilitation, at home

Tatiana Azevedo, Salete Soares, Jacinta Pisco

P52 Effects of Melatonin use in the treatment of neurovegetative diseases

Paulo P. Ferreira, Efrain O. Olszewer, Michelle T. Oliveira, Anderson R. Sousa, Ana S. Maia, Sebastião T. Oliveira

P53 Review of Phytotherapy and other natural substances in alcohol abuse and alcoholism

Erica Santos, Ana I. Oliveira, Carla Maia, Fernando Moreira, Joana Santos, Maria F. Mendes, Rita F. Oliveira, Cláudia Pinho

P54 Dietary programme impact on biochemical markers in diabetics: systematic review

Eduarda Barreira, Ana Pereira, Josiana A. Vaz, André Novo

P55 Biological approaches to knee osteoarthritis: platelet-rich plasma and hyaluronic acid

Luís D. Silva, Bruno Maia, Eduardo Ferreira, Filipa Pires, Renato Andrade, Luís Camarinha

P56 Platelet-rich plasma and hyaluronic acid intra-articular injections for the treatment of ankle osteoarthritis

Luís D. Silva, Bruno Maia, Eduardo Ferreira, Filipa Pires, Renato Andrade, Luís Camarinha

P57 The impact of preventive measures in the incidence of diabetic foot ulcers: a systematic review

Ana F. César, Mariana Poço, David Ventura, Raquel Loura, Pedro Gomes, Catarina Gomes, Cláudia Silva, Elsa Melo, João Lindo

P58 Dating violence among young adolescents

Joana Domingos, Zaida Mendes, Susana Poeta, Tiago Carvalho, Catarina Tomás, Helena Catarino, Mª Anjos Dixe

P59 Physical activity and motor memory in pedal dexterity

André Ramalho, António Rosado, Pedro Mendes, Rui Paulo, Inês Garcia, João Petrica

P60 The effects of whole body vibration on the electromyographic activity of thigh muscles

Sandra Rodrigues, Rui Meneses, Carlos Afonso, Luís Faria, Adérito Seixas

P61 Mental health promotion in the workplace

Marina Cordeiro, Paulo Granjo, José C. Gomes

P62 Influence of physical exercise on the self-perception of body image in elderly women: A systematic review of qualitative studies

Nelba R. Souza, Guilherme E. Furtado, Saulo V. Rocha, Paula Silva, Joana Carvalho

O109 Psychometric properties of the Portuguese version of the Éxamen Geronto-Psychomoteur (P-EGP)

Marina Ana Morais, Sofia Santos, Paula Lebre, Ana Antunes

O110 Symptoms of depression in the elderly population of Portugal, Spain and Italy

António Calha

O111 Emotion regulation strategies and psychopathology symptoms: A comparison between adolescents with and without deliberate self-harm

Ana Xavier, Marina Cunha, José Pinto-Gouveia

O112 Prevalence of physical disability in people with leprosy

Liana Alencar, Madalena Cunha, António Madureira

O113 Quality of life and self-esteem in type 1 and type 2 diabetes mellitus patients

Ilda Cardoso, Ana Galhardo, Fernanda Daniel, Vítor Rodrigues

O114 Cross-cultural comparison of gross motor coordination in children from Brazil and Portugal

Leonardo Luz, Tatiana Luz, Maurício R. Ramos, Dayse C. Medeiros, Bruno M. Carmo, André Seabra, Cristina Padez, Manuel C. Silva

O115 Electrocardiographic differences between African and Caucasian people

António Rodrigues, Patrícia Coelho, Alexandre Coelho

O116 Factors associated with domestic, sexual and other types of violence in the city of Palhoça - Brazil

Madson Caminha, Filipe Matheus, Elenice Mendes, Jony Correia, Marcia Kretzer

O117 Tinnitus prevalence study of users of a hospital of public management - Spain

Francisco J. Hernandez-Martinez, Juan F. Jimenez-Diaz, Bienvendida C. Rodriguez-De-Vera, Carla Jimenez-Rodriguez, Yadira Armas-Gonzalez

O118 Difficulties experienced by parents of children with diabetes mellitus of preschool age in therapeutic and nutritional management

Cátia Rodrigues, Rosa Pedroso

O119 E-mental health - “nice to have” or “must have”? Exploring the attitudes towards e-mental health in the general population

Jennifer Apolinário-Hagen, Viktor Vehreschild

O120 Violence against children and adolescents and the role of health professionals: Knowing how to identify and care

Milene Veloso, Celina Magalhães, Isabel Cabral, Maira Ferraz

O121 Marital violence. A study in the Algarve population

Filipe Nave, Emília Costa, Filomena Matos, José Pacheco

O122 Clinical factors and adherence to treatment in ischemic heart disease

António Dias, Carlos Pereira, João Duarte, Madalena Cunha, Daniel Silva

O123 Can religiosity improve optimism in participants in states of illness, when controlling for life satisfaction?

Lisete M. Mónico, Valentim R. Alferes, Mª São João Brêda, Carla Carvalho, Pedro M. Parreira

O124 Empowerment, knowledge and quality of life of people with diabetes type 2 in the Alto Minho Health Local Unit

Mª Carminda Morais, Pedro Ferreira, Rui Pimenta, José Boavida

O125 Antihypertensive therapy adherence among hypertensive patients from Bragança county, Portugal

Isabel C. Pinto, Tânia Pires, Catarina Silva

O126 Subjective perception of sexual achievement - An exploratory study on people with overweight

Maria Ribeiro, Maria Viega-Branco, Filomena Pereira, Ana Mª Pereira

O127 Physical activity level and associated factors in hypertensive individuals registered in the family health strategy of a basic health unit from the city of Palhoça, Santa Catarina, Brazil

Fabrícia M. Almeida, Gustavo L. Estevez, Sandra Ribeiro, Marcia R. Kretzer

O128 Perception of functional fitness and health in non-institutionalised elderly from rural areas

Paulo V. João, Paulo Nogueira, Sandra Novais, Ana Pereira, Lara Carneiro, Maria Mota

O129 Medication adherence in patients with type 2 diabetes mellitus treated at primary health care in Coimbra

Rui Cruz, Luiz Santiago, Carlos Fontes-Ribeiro

O130 Multivariate association between body mass index and multi-comorbidities in elderly people living in low socio-economic status context

Guilherme Furtado, Saulo V. Rocha, André P. Coutinho, João S. Neto, Lélia R. Vasconcelos, Nelba R. Souza, Estélio Dantas

O131 Metacognition, rumination and experiential avoidance in Borderline Personality Disorder

Alexandra Dinis, Sérgio Carvalho, Paula Castilho, José Pinto-Gouveia

O132 Health issues in a vulnerable population: nursing consultation in a public bathhouse in Lisbon

Alexandra Sarreira-Santos, Amélia Figueiredo, Lurdes Medeiros-Garcia, Paulo Seabra

O133 The perception of quality of life in people with multiple sclerosis accompanied in External Consultation of the Local Health Unit of Alto Minho

Rosa Rodrigues, Mª Carminda Morais, Paula O. Fernandes

O134 Representation of interaction established between immigrant women and nurse during pregnancy to postpartum, from the perspective of immigrant women

Conceição Santiago, Mª Henriqueta Figueiredo, Marta L. Basto

O135 Illness perceptions and medication adherence in hypertension

Teresa Guimarães, André Coelho, Anabela Graça, Ana M. Silva, Ana R. Fonseca

O136 A Portuguese study on adults’ intimate partner violence, interpersonal trust and hope

Luz Vale-Dias, Bárbara Minas, Graciete Franco-Borges

P63 QOL’ predictors of people with intellectual disability and general population

Cristina Simões, Sofia Santos

P64 Content validation of the Communication Disability Profile (CDP) - Portuguese Version

Ana Serra, Maria Matos, Luís Jesus

P65 Study of biochemical and haematological changes in football players

Ana S. Tavares, Ana Almeida, Céu Leitão, Edna Varandas, Renato Abreu, Fernando Bellém

P66 Body image dissatisfaction in inflammatory bowel disease: exploring the role of chronic illness-related shame

Inês A. Trindade, Cláudia Ferreira, José Pinto-Gouveia, Joana Marta-Simões

P67 Obesity and sleep in the adult population - a systematic review

Odete Amaral, Cristiana Miranda, Pedro Guimarães, Rodrigo Gonçalves, Nélio Veiga, Carlos Pereira

P68 Frequency of daytime sleepiness and obstructive sleep apnea risk in COPD patients

Tânia C. Fleig, Elisabete A. San-Martin, Cássia L. Goulart, Paloma B. Schneiders, Natacha F. Miranda, Lisiane L. Carvalho, Andrea G. Silva

P69 Working with immigrant-origin clients: discourses and practices of health professionals

Joana Topa, Conceição Nogueira, Sofia Neves

P70 Systemic Lupus Erythematosus – what are audiovestibular changes?

Rita Ventura, Cristina Nazaré

P71 Mental disorders in the oldest old: findings from the Portuguese national hospitalization database

Daniela Brandão, Alberto Freitas, Óscar Ribeiro, Constança Paúl

P72 Recurrence analysis in postural control in children with cerebral palsy

Cristiana Mercê, Marco Branco, Pedro Almeida, Daniela Nascimento, Juliana Pereira, David Catela

P73 The experience of self-care in the elderly with COPD: contributions to reflect proximity care

Helga Rafael

P74 Culturally competent nurses: managing unpredictability in clinical practice with immigrants

Alcinda C. Reis

O137 Paediatric speech and language screening: An instrument for health professionals

Ana Mendes, Ana R. Valente, Marisa Lousada

O138 Anthropometric and nutritional assessment in bodybuilders

Diana Sousa, Ana L. Baltazar, Mª Helena Loureiro

O139 Computerized adventitious respiratory sounds in children with lower respiratory tract infections

Ana Oliveira, José Aparício, Alda Marques

O140 Role of computerized respiratory sounds as a marker in LRTI

Alda Marques, Ana Oliveira, Joana Neves, Rodrigo Ayoub

O141 Confirmatory factor analysis of the Personal Wellbeing Index in people with chronic kidney disease

Luís Sousa, Cristina Marques-Vieira, Sandy Severino, Helena José

O142 Phonological awareness skills in school aged children

Inês Cadorio, Marisa Lousada

O143 Assessment of early memories of warmth and safeness in interaction with peers: its relationship with psychopathology in adolescence

Marina Cunha, Diogo Andrade, Ana Galhardo, Margarida Couto

O144 The molecular effects induced by single shot irradiation on a diffuse large B cell lymphoma cell line

Fernando Mendes, Cátia Domingues, Susann Schukg, Ana M. Abrantes, Ana C. Gonçalves, Tiago Sales, Ricardo Teixo, Rita Silva, Jéssica Estrela, Mafalda Laranjo, João Casalta-Lopes, Clara Rocha, Paulo C. Simões, Ana B. Sarmento-Ribeiro, Mª Filomena Botelho, Manuel S. Rosa

O145 Morpho-functional characterization of cardiac chambers by Transthoracic Echocardiography, in young athletes of gymnastics competition

Virgínia Fonseca, Diogo Colaço, Vanessa Neves

O146 Prevalence of the antibodies of the new histo-blood system – FORS system

Carlos Jesus, Camilla Hesse, Clara Rocha, Nádia Osório, Ana Valado, Armando Caseiro, António Gabriel, Lola Svensson, Fernando Mendes, Wafa A. Siba, Cristina Pereira, Jorge Tomaz

O147 Assessment of the war-related perceived threat in Portuguese Colonial War Veterans

Teresa Carvalho, José Pinto-Gouveia, Marina Cunha

O148 Pulse transit time estimation for continuous blood pressure measurement: A comparative study

Diana Duarte, Nuno V. Lopes, Rui Fonseca-Pinto

O149 Blood pressure assessment during standard clinical manoeuvres: A non-invasive PPT based approach

Diana Duarte, Nuno V. Lopes, Rui Fonseca-Pinto

O150 Development and initial validation of the Activities and Participation Profile related to Mobility (APPM)

Anabela C. Martins

O151 MEASYCare-2010 Standard–A geriatric evaluation system in primary health care: Reliability and validity of the latest version in Portugal

Piedade Brandão, Laura Martins, Margarida Cardoso

O152 Interrater and intrarater reliability and agreement of the range of shoulder flexion in the standing upright position through photographic assessment

Nuno Morais, Joana Cruz

O153 Three-dimensional biofabrication techniques for tissue regeneration

Nuno Alves, Paula Faria, Artur Mateus, Pedro Morouço

O154 A new computer tool for biofabrication applied to tissue engineering

Nuno Alves, Nelson Ferreira, Artur Mateus, Paula Faria, Pedro Morouço

O155 Development and psychometric qualities of a scale to measure the functional independence of adolescents with motor impairment

Isabel Malheiro, Filomena Gaspar, Luísa Barros

O156 Organizational Trust in Health services: Exploratory and Confirmatory factor analysis of the Organizational Trust Inventory- Short Form (OTI-SF)

Pedro Parreira, Andreia Cardoso, Lisete Mónico, Carla Carvalho, Albino Lopes, Anabela Salgueiro-Oliveira

O157 Thermal symmetry: An indicator of occupational task asymmetries in physiotherapy

Adérito Seixas, Valter Soares, Tiago Dias, Ricardo Vardasca, Joaquim Gabriel, Sandra Rodrigues

O158 A study of ICT active monitoring adoption in stroke rehabilitation

Hugo Paredes, Arsénio Reis, Sara Marinho, Vítor Filipe, João Barroso

O159 Paranoia Checklist (Portuguese Version): Preliminary studies in a mixed sample of patients and healthy controls

Carolina Da Motta, Célia B. Carvalho, José Pinto-Gouveia, Ermelindo Peixoto

O160 Reliability and validity of the Composite Scale on Morningness: European Portuguese version, in adolescents and young adults

Ana A. Gomes, Vanessa Costa, Diana Couto, Daniel R. Marques, José A. Leitão, José Tavares, Maria H. Azevedo, Carlos F. Silva

O161 Evaluation scale of patient satisfaction with nursing care: Psychometric properties evaluation

João Freitas, Pedro Parreira, João Marôco

O162 Impact of fibromyalgia on quality of life: Comparing results from generic instruments and FIQR

Miguel A. Garcia-Gordillo, Daniel Collado-Mateo, Gang Chen, Angelo Iezzi, José A. Sala, José A. Parraça, Narcis Gusi

O163 Preliminary study of the adaptation and validation of the Rating Scale of Resilient Self: Resilience, self-harm and suicidal ideation in adolescents

Jani Sousa, Mariana Marques, Jacinto Jardim, Anabela Pereira, Sónia Simões, Marina Cunha

O164 Development of the first pressure ulcer in inpatient setting: Focus on length of stay

Pedro Sardo, Jenifer Guedes, João Lindo, Paulo Machado, Elsa Melo

O165 Forms of Self-Criticizing and Self-Reassuring Scale: Adaptation and early findings in a sample of Portuguese children

Célia B. Carvalho, Joana Benevides, Marina Sousa, Joana Cabral, Carolina Da Motta

O166 Predictive ability of the Perinatal Depression Screening and Prevention Tool – Preliminary results of the dimensional approach

Ana T. Pereira, Sandra Xavier, Julieta Azevedo, Elisabete Bento, Cristiana Marques, Rosa Carvalho, Mariana Marques, António Macedo

O167 Psychometric properties of the BaSIQS-Basic Scale on insomnia symptoms and quality of sleep, in adults and in the elderly

Ana M. Silva, Juliana Alves, Ana A. Gomes, Daniel R. Marques, Mª Helena Azevedo, Carlos Silva

O168 Enlightening the human decision in health: The skin melanocytic classification challenge

Ana Mendes, Huei D. Lee, Newton Spolaôr, Jefferson T. Oliva, Wu F. Chung, Rui Fonseca-Pinto

O169 Test-retest reliability household life study and health questionnaire Pomerode (SHIP-BRAZIL)

Keila Bairros, Cláudia D. Silva, Clóvis A. Souza, Silvana S. Schroeder

O170 Characterization of sun exposure behaviours among medical students from Nova Medical School

Elsa Araújo, Helena Monteiro, Ricardo Costa, Sara S. Dias, Jorge Torgal

O171 Spirituality in pregnant women

Carolina G. Henriques, Luísa Santos, Elisa F. Caceiro, Sónia A. Ramalho

O172 Polypharmacy in older patients with cancer

Rita Oliveira, Vera Afreixo, João Santos, Priscilla Mota, Agostinho Cruz, Francisco Pimentel

O173 Quality of life of caregivers of people with advanced chronic disease: Translation and validation of the quality of life in life threatening illness - family carer version (QOLLTI-C-PT)

Rita Marques, Mª Anjos Dixe, Ana Querido, Patrícia Sousa

O174 The psychometric properties of the brief Other as Shamer Scale for Children (OAS-C): preliminary validation studies in a sample of Portuguese children

Joana Benevides, Carolina Da Motta, Marina Sousa, Suzana N. Caldeira, Célia B. Carvalho

O175 Measuring emotional intelligence in health care students – Revalidation of WLEIS-P

Ana Querido, Catarina Tomás, Daniel Carvalho, João Gomes, Marina Cordeiro

O176 Health indicators in prenatal assistance: The impact of computerization and of under-production in basic health centres

Joyce O. Costa, Frederico C. Valim, Lígia C. Ribeiro

O177 Hope genogram: Assessment of resources and interaction patterns in the family of the child with cerebral palsy

Zaida Charepe, Ana Querido, Mª Henriqueta Figueiredo

O178 The influence of childbirth type in postpartum quality of life

Priscila S. Aquino, Samila G. Ribeiro, Ana B. Pinheiro, Paula A. Lessa, Mirna F. Oliveira, Luísa S. Brito, Ítalo N. Pinto, Alessandra S. Furtado, Régia B. Castro, Caroline Q. Aquino, Eveliny S. Martins

O179 Women’s beliefs about pap smear test and cervical cancer: influence of social determinants

Ana B Pinheiro, Priscila S. Aquino, Lara L. Oliveira, Patrícia C. Pinheiro, Caroline R. Sousa, Vívien A. Freitas, Tatiane M. Silva, Adman S. Lima, Caroline Q. Aquino, Karizia V. Andrade, Camila A. Oliveira, Eglidia F. Vidal

O180 Validity of the Portuguese version of the ASI-3: Is anxiety sensitivity a unidimensional or multidimensional construct?

Ana Ganho-Ávila, Mariana Moura-Ramos, Óscar Gonçalves, Jorge Almeida

O181 Lifestyles of higher education students: the influence of self-esteem and psychological well-being

Armando Silva, Irma Brito, João Amado

P75 Assessing the quality of life of persons with significant intellectual disability: Portuguese version of Escala de San Martín

António Rodrigo, Sofia Santos, Fernando Gomes

P76 Childhood obesity and breastfeeding - A systematic review

Marlene C. Rosa, Silvana F. Marques

P77 Cross-cultural adaptation of the Foot and Ankle Ability Measure (FAAM) for the Portuguese population

Sara Luís, Luís Cavalheiro, Pedro Ferreira, Rui Gonçalves

P78 Cross-cultural adaptation of the Patient-Rated Wrist Evaluation score (PRWE) for the Portuguese population

Rui S. Lopes, Luís Cavalheiro, Pedro Ferreira, Rui Gonçalves

P79 Cross-cultural adaptation of the Myocardial Infraction Dimensional Assessment Scale (MIDAS) for Brazilian Portuguese language

Bruno H. Fiorin, Marina S. Santos, Edmar S. Oliveira, Rita L. Moreira, Elizabete A. Oliveira, Braulio L. Filho

P80 The revised Portuguese version of the Three-Factor Eating Questionnaire: A confirmatory factor analysis

Lara Palmeira, Teresa Garcia, José Pinto-Gouveia, Marina Cunha

P81 Assessing weight-related psychological inflexibility: An exploratory factor analysis of the AAQW’s Portuguese version

Sara Cardoso, Lara Palmeira, Marina Cunha; José Pinto-Gouveia

P82 Validation of the Body Appreciation Scale-2 for Portuguese women

Joana Marta-Simões, Ana L. Mendes, Inês A. Trindade, Sara Oliveira, Cláudia Ferreira

P83 The Portuguese validation of the Dietary Intent Scale

Ana L. Mendes, Joana Marta-Simões, Inês A. Trindade, Cláudia Ferreira

P84 Construction and validation of the Inventory of Marital Violence (IVC)

Filipe Nave

P85 Portable continuous blood pressure monitor system

Mariana Campos, Iris Gaudêncio, Fernando Martins, Lino Ferreira, Nuno Lopes, Rui Fonseca-Pinto

P86 Construction and validation of the Scale of Perception of the Difficulties in Caring for the Elderly (SPDCE)

Rogério Rodrigues, Zaida Azeredo, Corália Vicente

P87 Development and validation of a comfort rating scale for the elderly hospitalized with chronic illness

Joana Silva, Patrícia Sousa, Rita Marques

P88 Construction and validation of the Postpartum Paternal Quality of Life Questionnaire (PP-QOL)

Isabel Mendes, Rogério Rodrigues, Zaida Azeredo, Corália Vicente

P89 Infrared thermal imaging: A tool for assessing diabetic foot ulcers

Ricardo Vardasca, Ana R. Marques, Adérito Seixas, Rui Carvalho, Joaquim Gabriel

P90 Pressure ulcers in an intensive care unit: An experience report

Paulo P. Ferreira, Michelle T. Oliveira, Anderson R. Sousa, Ana S. Maia, Sebastião T. Oliveira, Pablo O. Costa, Maiza M. Silva

P91 Validation of figures used in evocations: instrument to capture representations

Cristina Arreguy-Sena, Nathália Alvarenga-Martins, Paulo F. Pinto, Denize C. Oliveira, Pedro D. Parreira, Antônio T. Gomes, Luciene M. Braga

P92 Telephone assistance to decrease burden in informal caregivers of stroke older people: Monitoring and diagnostic evaluation

Odete Araújo, Isabel Lage, José Cabrita, Laetitia Teixeira

P93 Hope of informal caregivers of people with chronic and advanced disease

Rita Marques, Mª Anjos Dixe, Ana Querido, Patrícia Sousa

P94 Functionality and quality information from the Portuguese National Epidemiological Surveillance System

Sara Silva, Eugénio Cordeiro, João Pimentel

P95 Resting metabolic rate objectively measured vs. Harris and Benedict formula

Vera Ferro-Lebres, Juliana A. Souza, Mariline Tavares

O182 Characteristics of non-urgent patients: Cross-sectional study of an emergency department

Mª Anjos Dixe, Pedro Sousa, Rui Passadouro, Teresa Peralta, Carlos Ferreira, Georgina Lourenço

O183 Physical fitness and health in children of the 1st Cycle of Education

João Serrano, João Petrica, Rui Paulo, Samuel Honório, Pedro Mendes

O184 The impact of physical activity on sleep quality, in children

Alexandra Simões, Lucinda Carvalho, Alexandre Pereira

O185 What is the potential for using Information and Communication Technologies in Arterial Hypertension self-management?

Sara Silva, Paulino Sousa, José M. Padilha

O186 Exploring psychosocial factors associated with risk of falling in older patients undergoing haemodialysis

Daniela Figueiredo, Carolina Valente, Alda Marques

O187 Development of pressure ulcers on the face in patients undergoing non-invasive ventilation

Patrícia Ribas, Joana Sousa, Frederico Brandão, Cesar Sousa, Matilde Martins

O188 The elder hospitalized: Limiting factors of comfort

Patrícia Sousa, Rita Marques

O189 Physical activity and health state self-perception by Portuguese adults

Francisco Mendes, Rosina Fernandes, Emília Martins, Cátia Magalhães, Patrícia Araújo

O190 Satisfaction with social support in the elderly of the district of Bragança

Carla Grande, Mª Augusta Mata, Juan G. Vieitez

O191 Prevalence of death by traumatic brain injury and associated factors in intensive care unit of a general hospital, Brazil

Bruna Bianchini, Nazare Nazario, João G. Filho, Marcia Kretzer

O192 Relation between family caregivers burden and health status of elderly dependents

Tânia Costa, Armando Almeida, Gabriel Baffour

O193 Phenomena sensitive to nursing care in day centre

Armando Almeida, Tânia Costa, Gabriel Baffour

O194 Frailty: what do the elderly think?

Zaida Azeredo, Carlos Laranjeira, Magda Guerra, Ana P. Barbeiro

O195 The therapeutic self-care as a nursing-sensitive outcome: A correlational study

Regina Ferreira

O196 Phonetic-phonological acquisition for the European Portuguese from 18 months to 6 years and 12 months

Sara Lopes, Liliana Nunes, Ana Mendes

O197 Quality of life of patients undergoing liver transplant surgery

Julian Martins, Dulcineia Schneider, Marcia Kretzer, Flávio Magajewski

O198 Professional competences in health: views of older people from different European Countries

Célia Soares, António Marques

O199 Life satisfaction of working adults due to the number of hours of weekly exercise

Marco Batista, Ruth J. Castuera, Helena Mesquita, António Faustino, Jorge Santos, Samuel Honório

O200 Therapeutic itinerary of women with breast cancer in Santa Maria City/RS

Betina P. Vizzotto, Leticia Frigo, Hedioneia F. Pivetta

O201 The breastfeeding prevalence at 4 months: Maternal experience as a determining factor

Dolores Sardo

O202 The impact of the transition to parenthood in health and well-being

Cristina Martins, Wilson Abreu, Mª Céu Figueiredo

P96 Self-determined motivation and well-being in Portuguese active adults of both genders

Marco Batista, Ruth Jimenez-Castuera, João Petrica, João Serrano, Samuel Honório, Rui Paulo, Pedro Mendes

P97 The geriatric care: ways and means of comforting

Patrícia Sousa, Rita Marques

P98 The influence of relative age, subcutaneous adiposity and physical growth on Castelo Branco under-15 soccer players 2015

António Faustino, Paulo Silveira, João Serrano, Rui Paulo, Pedro Mendes, Samuel Honório

P99 Data for the diagnostic process focused on self-care – managing medication regime: An integrative literature review

Catarina Oliveira, Fernanda Bastos, Inês Cruz

P100 Art therapy as mental health promotion for children

Cláudia K. Rodriguez, Márcia R. Kretzer, Nazaré O. Nazário

P101 Chemical characterization of fungal chitosan for industrial applications

Pedro Cruz, Daniela C. Vaz, Rui B. Ruben, Francisco Avelelas, Susana Silva, Mª Jorge Campos

P102 The impact of caring older people at home

Maria Almeida, Liliana Gonçalves, Lígia Antunes

P103 Development of the first pressure ulcer in an inpatient setting: Focus on patients’ characteristics

Pedro Sardo, Jenifer Guedes, João Simões, Paulo Machado, Elsa Melo

P104 Association between General Self-efficacy and Physical Activity among Adolescents

Susana Cardoso, Osvaldo Santos, Carla Nunes, Isabel Loureiro

O203 Characterization of the habits of online acquisition of medicinal products in Portugal

Flávia Santos, Gilberto Alves

O204 Waiting room – A space for health education

Cláudia Soar, Teresa O. Marsi

O205 Safey culture evaluation in hospitalized children

Ernestina Silva, Dora Pedrosa, Andrea Leça, Daniel Silva

O206 Sexual Self-awareness and Body Image

Ana Galvão, Maria Gomes, Paula Fernandes, Ana Noné

O207 Perception of a Portuguese population regarding the acquisition and consumption of functional foods

Jaime Combadão, Cátia Ramalhete, Paulo Figueiredo, Patrícia Caeiro

O208 The work process in primary health care: evaluation in municipalities of southern Brazil

Karine C. Fontana, Josimari T. Lacerda, Patrícia O. Machado

O209 Exploration and evaluation of potential probiotic lactic acid bacteria isolated from Amazon buffalo milk

Raphaelle Borges, Flávio Barbosa, Dayse Sá

O210 Road safety for children: Using children’s observation, as a passenger

Germana Brunhoso, Graça Aparício, Amâncio Carvalho

O211 Perception and application of quality-by-design by the Pharmaceutical industry in Portugal

Ana P. Garcia, Paula O. Fernandes, Adriana Santos

O212 Oral health among Portuguese children and adolescents: a public health issue

Nélio Veiga, Carina Brás, Inês Carvalho, Joana Batalha, Margarida Glória, Filipa Bexiga, Inês Coelho, Odete Amaral, Carlos Pereira

O213 Plant species as a medicinal resource in Igatu-Chapada Diamantina (Bahia, Brazil)

Cláudia Pinho, Nilson Paraíso, Ana I. Oliveira, Cristóvão F. Lima, Alberto P. Dias

O214 Characterization of cognitive and functional performance in everyday tasks: Implications for health in institutionalised older adults

Pedro Silva, Mário Espada, Mário Marques, Ana Pereira

O215 BMI and the perception of the importance given to sexuality in obese and overweight people

Ana Mª Pereira, Mª Veiga-Branco, Filomena Pereira, Maria Ribeiro

O216 Analysis and comparison of microbiological contaminations of two different composition pacifiers

Vera Lima, Ana I. Oliveira, Cláudia Pinho, Graça Cruz, Rita F. Oliveira, Luísa Barreiros, Fernando Moreira

O217 Experiences of couple relationships in the transition to retirement

Ana Camarneiro, Mª Helena Loureiro, Margarida Silva

O218 Preventive and corrective treatment of drug-induced calcium deficiency: an analysis in a community pharmacy setting

Catarina Duarte, Ângelo Jesus, Agostinho Cruz

O219 Profile of mood states in physically active elderly subjects: Is there a relation with health perception?

Maria Mota, Sandra Novais, Paulo Nogueira, Ana Pereira, Lara Carneiro, Paulo V. João

O220 (Un)Safety behaviour at work: the role of education towards a health and safety culture

Teresa Maneca Lima

O221 Analysis of the entrepreneurial profile of students attending higher education in Portugal: the Carland Entrepreneurship Index application

Anabela Salgueiro-Oliveira, Marina Vaquinhas, Pedro Parreira, Rosa Melo, João Graveto, Amélia Castilho, José H. Gomes

O222 Evaluation of welfare and quality of life of pregnant working women regarding the age of the pregnant

María S. Medina, Valeriana G. Blanco

O223 Psychological wellbeing protection among unemployed and temporary workers: Uncovering effective community-based interventions with a Delphi panel

Osvaldo Santos, Elisa Lopes, Ana Virgolino, Alexandra Dinis, Sara Ambrósio, Inês Almeida, Tatiana Marques, Mª João Heitor

O224 Chilean population norms derived from the Health-related quality of life SF-6D

Miguel A. Garcia-Gordillo, Daniel Collado-Mateo, Pedro R. Olivares, José A. Parraça, José A. Sala

O225 Motivation of college students toward Entrepreneurship: The influence of social and economic instability

Amélia Castilho, João Graveto, Pedro Parreira, Anabela Oliveira, José H. Gomes, Rosa Melo, Marina Vaquinhas

O226 Use of aromatic and medicinal plants, drugs and herbal products in Bragança city

Mónia Cheio, Agostinho Cruz, Olívia R. Pereira

O227 Edible flowers as new novel foods concept for health promotion

Sara Pinto, Adriana Oliveira, M. Conceição Manso, Carla Sousa, Ana F. Vinha

O228 The influence of leisure activities on the health and welfare of older people living in nursing homes

Mª Manuela Machado, Margarida Vieira

O229 Risk of falling, fear of falling and functionality in community-dwelling older adults

Beatriz Fernandes, Teresa Tomás, Diogo Quirino

O230 Musculoskeletal pain and postural habits in children and teenage students

Gustavo Desouzart, Rui Matos, Magali Bordini, Pedro Mouroço

O231 What's different in Southern Europe? The question of citizens’ participation in health systems

Ana R. Matos, Mauro Serapioni

O232 Occupational stress in Portuguese police officers

Teresa Guimarães, Virgínia Fonseca, André Costa, João Ribeiro, João Lobato

O233 Is occupational therapy culturally relevant to promote mental health in Burkina Faso?

Inmaculada Z. Martin, Anita Björklund

P105 Pay-for-performance satisfaction and quality in primary care

Aida I. Tavares, Pedro Ferreira, Rui Passadouro

P106 Economic development through life expectancy lenses

Sónia Morgado

P107 What is the effectiveness of exercise on smoking cessation to prevent clinical complications of smoking?

Nuno Tavares, João Valente, Anabela C. Martins

P108 A systematic review of the effects of yoga on mental health

Patrícia Araújo, Rosina Fernandes, Francisco Mendes, Cátia Magalhães, Emília Martins

P109 Healthy lifestyle: comparison between higher education students that lived until adult age in rural and urban environment

Pedro Mendes, Rui Paulo, António Faustino, Helena Mesquita, Samuel Honório, Marco Batista

P110 Evaluation of the Mobile Emergency Care Service (SAMU) in Brazil

Josimari T. Lacerda, Angela B. Ortiga, Mª Cristina Calvo, Sônia Natal

P111 Bioactive compounds - antioxidant activity of tropical fruits

Marta Pereira

P112 Use of non-pharmacological methods to relieve pain in labour

Manuela Ferreira, Ana R. Prata, Paula Nelas, João Duarte

P113 Mechanical safety of pacifiers sold in Portuguese pharmacies and childcare stores

Juliana Carneiro, Ana I. Oliveira, Cláudia Pinho, Cristina Couto, Rita F. Oliveira, Fernando Moreira

P114 The importance of prenatal consultation: Information to pregnant women given on a unit of primary care

Ana S. Maia, Michelle T. Oliveira, Anderson R. Sousa, Paulo P. Ferreira, Géssica M. Souza, Lívia F. Almada, Milena A. Conceição, Eujcely C. Santiago

P115 Influence of different backpack loading conditions on neck and lumbar muscles activity of elementary school children

Sandra Rodrigues, Gabriela Domingues, Irina Ferreira, Luís Faria, Adérito Seixas

P116 Efficacy and safety of dry extract Hedera helix in the treatment of productive cough

Ana R. Costa, Ângelo Jesus, Américo Cardoso, Alexandra Meireles, Armanda Colaço, Agostinho Cruz

P117 A portrait of the evaluation processes of education groups in primary health care

Viviane L. Vieira, Kellem R. Vincha, Ana Mª Cervato-Mancuso

P118 Benefits of vitamins C and E in sensorineural hearing loss: a review

Melissa Faria, Cláudia Reis

P119 BODY SNAPSHOT – a web-integrated anthropometric evaluation system

Marco P. Cova, Rita T. Ascenso, Henrique A. Almeida, Eunice G. Oliveira

P120 Anthropometric evaluation and variation during pregnancy

Miguel Santana, Rafael Pereira, Eunice G. Oliveira, Henrique A. Almeida, Rita T. Ascenso

P121 Knowledge of college students on the amendments of their eating habits and physical activity index in the transition to higher education

Rita Jesus, Rodrigo Tapadas, Carolina Tim-Tim, Catarina Cezanne, Matilde Lagoa, Sara S. Dias, Jorge Torgal

P122 Muscular activity of a rally race car driver

João Lopes, Henrique Almeida, Sandra Amado, Luís Carrão

O234 Literacy and results in health

Madalena Cunha, Luís Saboga-Nunes, Carlos Albuquerque, Olivério Ribeiro

O235 Literacy promotion and empowerment of type 2 diabetics elderly in four family health units of the group of health centers of Dão Lafões

Suzete Oliveira, Mª Carminda Morais

O236 Mediterranean diet, health and life quality among Portuguese children

Emília Martins, Francisco Mendes, Rosina Fernandes, Cátia Magalhães, Patrícia Araújo

O237 Health literacy, from data to action - translation, validation and application of the European Health Literacy Survey in Portugal (HLS-EU-PT)

Ana R. Pedro, Odete Amaral, Ana Escoval

O238 Oral health literacy evaluation in a Portuguese military population

Victor Assunção, Henrique Luís, Luís Luís

O239 Preferences to Internet-based cognitive behavioural therapy – do attachment orientations matter?

Jennifer Apolinário-Hagen, Viktor Vehreschild

O240 A comparative transnational study in health literacy between Austria and Portugal

Ulrike Fotschl, Gerald Lirk, Anabela C. Martins, Isabel Andrade, Fernando Mendes

O241 Health literacy and social behaviours: relationship with sexually transmitted diseases?

Verónica Mendonça, Sandra Antunes, Isabel Andrade, Nádia Osório, Ana Valado, Armando Caseiro, António Gabriel, Anabela C. Martins, Fernando Mendes

O242 Parenting styles and attachment to parents: what relationships?

Paula A. Silva, Lisete M. Mónico, Pedro M. Parreira, Carla Carvalho

O243 Work-life balance in health professionals and professors: comparative study of workers with shift work and fixed schedule

Carla Carvalho, Pedro M. Parreira, Lisete M. Mónico, Joana Ruivo

O244 Technology literacy in self-management of diabetes

Vânia Silva, Paulino Sousa, José M. Padilha

O245 Satisfaction with therapeutic education and its relationship with clinical variables in children with type 1 diabetes

Vera Ferraz, Graça Aparício, João Duarte

O246 Nutrition-related knowledge in middle-age and older patients with type 2 diabetes

Carlos Vasconcelos, António Almeida, Joel Neves, Telma Correia, Helena Amorim, Romeu Mendes

O247 Validating the HLS-EU-(PT) questionnaire to measure health literacy in adolescents (CrAdLiSa project: HLS-EU-PT)

Luís Saboga-Nunes, Madalena Cunha, Carlos Albuquerque

O248 Health education in people with coronary heart disease: Experience of the cardiology department of a hospital on the outskirts of Lisbon

Elsa S. Pereira, Leonino S. Santos, Ana S. Reis, Helena R. Silva, João Rombo, Jorge C. Fernandes, Patrícia Fernandes

O249 Information and training needs of informal caregivers of individuals with stroke sequelae: a qualitative survey

Jaime Ribeiro, Catarina Mangas, Ana Freire

O250 Prevention of psychoactive substances consumption in students from 6th grade of Albergaria-a-Velha´s School Group

Sara Silva, Irene Francisco, Ana Oliveira

O251 Promoting healthy sexuality: shared responsibility for family, youth and educators

Helena Catarino, Mª Anjos Dixe, Mª Clarisse Louro

O252 Sexual risk behaviour in adolescents and young people

Saudade Lopes, Anjos Dixe

O253 Knowledge of school staff on type 1 diabetes

Mª Anjos Dixe, Eva Menino, Helena Catarino, Fátima Soares, Ana P. Oliveira, Sara Gordo, Teresa Kraus

O254 Sexual health in adolescents: the impact of information search in literacy

Catarina Tomás, Paulo Queirós, Teresa Rodrigues

P123 Improving basic life support skills in adolescents through a training programme

Pedro Sousa, João G. Frade, Catarina Lobão

P124 Difficulties in sexual education reported by basic education teachers in the city of Foz do Iguaçu - Brazil

Cynthia B. Moura, Laysa C. Dreyer, Vanize Meneghetti, Priscila P. Cabral

P125 Breast cancer survivors: subjects and resources for information. A qualitative systematic review

Francisca Pinto, Paulino Sousa, Mª Raquel Esteves

P126 Relationship between health literacy and prevalence of STI in Biomedical Laboratory Science students

Sofia Galvão, Ite Tytgat, Isabel Andrade, Nádia Osório, Ana Valado, Armando Caseiro, António Gabriel, Anabela C. Martins, Fernando Mendes

P127 Health literacy, risk behaviours and sexually transmitted diseases among blood donors

Mónica Casas-Novas, Helena Bernardo, Isabel Andrade, Gracinda Sousa, Ana P. Sousa, Clara Rocha, Pedro Belo, Nádia Osório, Ana Valado, Armando Caseiro, António Gabriel, Anabela C. Martins, Fernando Mendes

P128 Promoting literacy in pregnancy health-care

Fátima Martins, Montserrat Pulido-Fuentes

P129 The lifestyles of the operating assistants of education

Isabel Barroso, Gil Cabral, M. João Monteiro, Conceição Rainho

P130 Experiences of service-learning health and the literary art: reflections about the health education

Alessandro Prado, Yara M. Carvalho

P131 Life long swimming – a European Erasmus + project

Maria Campos, Liliana Moreira, José Ferreira, Ana Teixeira, Luís Rama

## Session 1: Citizenship in health

### S1 Health literacy and health education in adolescence

#### Catarina Cardoso Tomás

##### Health Sciences Research Unit: Nursing, Collegue of College of Health Technology of Coimbra, Coimbra, Portugal

Health literacy, a more complex concept than knowledge, is a required capacity to obtain, understand, integrate and act on health information [1], in order to enhance individual and community health, which is defined by different levels, according to the autonomy and personal capacitation in decision making [2].

Medium levels of Health literacy in an adolescent population were found in a study conducted in 2013/2014, being higher in sexual and reproductive health and lower in substance use. It was also noticed that the higher levels of health literacy were in the area adolescents refer to have receipt more health information. The health literacy competence with higher scores was communication skills, and the lower scores were in the capacity to analyze factors that influence health. Higher levels were also found in younger teenagers, but in a higher school level, confirming the importance of health education in these age and development stage. Adolescents seek more information in health professionals and parents, being friends more valued as a source information in older adolescents, which enhance the importance of peer education mainly in older adolescents [3].

As a set of competences based on knowledge, health literacy should be developed through education interventions, encompassing the cultural and social context of individuals, since the society, culture and education system where the individual is inserted can define the way the development and enforcement of the health literacy competences [4]. The valued sources of information should be taken into account, as well as needs of information in some topics referred by adolescents in an efficient health education.

References

1. Borzekowski D. Considering Children and Health Literacy: A Theoretical Approach. Pediatrics. 2009; 124: S282-S288.

2. Nutbeam D. The evolving concept of health literacy. Soc Sci Med. 2008; 67: 2072-2078.

3. Tomás C. Literacia em Saúde na Adolescência [Doctoral Thesis]. OPorto: Abel Salazar Institute of Biomedical Sciences of Oporto University; 2014.

4. Committee on Health Literacy. Health Literacy: A Prescription To End Confusion. Washington, D. C.: The National Academies Press, 2004.

## Session 2: Evaluation & intervention in health

### S2 The effect of a walking program on the quality of life and well-being of people with schizophrenia

#### Emanuel Oliveira^1,2^, D. Sousa^1^, M. Uba-Chupel^2^, G. Furtado^2^, C. Rocha^3^, A. Teixeira^2^, P. Ferreira ^2^

##### ^1^Sisters Hospitallers of the Sacred Heart of Jesus, Casa de Saúde Rainha Santa Isabel, Coimbra, Portugal; ^2^Research Unit for Sport and Physical Activity, Faculty of Sport Sciences and Physical Education, University of Coimbra, Coimbra, Portugal; ^3^Complementary Sciences- INESCC, Coimbra, Portugal

###### **Correspondence:** Emanuel Oliveira – Sisters Hospitallers of the Sacred Heart of Jesus, Casa de Saúde Rainha Santa Isabel, Coimbra, Portugal

Schizophrenia is a serious and chronic mental illness which has a profound effect on the health and well-being related with the well-known nature of psychotic symptoms. The exercise has the potential to improve the life of people with schizophrenia improving physical health and alleviating psychiatric symptoms. However, most people with schizophrenia remains sedentary and lack of access to exercise programs are barriers to achieve health benefits. The aim of this study is to evaluate the effect of exercise on I) the type of intervention in mental health, II) in salivary levels of alpha-amylase and cortisol and serum levels of S100B and BDNF, and on III) the quality of life and self-perception of the physical domain of people with schizophrenia. The sample consisted of 31 females in long-term institutions in the Casa de Saúde Rainha Santa Isabel, with age between 25 and 63, and with diagnosis of schizophrenia according to the Diagnostic and Statistical Manual of Mental Disorders (DSM-IV-TR). Physical fitness was assessed by the six-minute walk distance test (6MWD). Biological variables were determined by ELISA (Enzyme-Linked Immunosorbent Assay). Psychological variables were assessed using SF-36, PSPP-SCV, RSES and SWLS tests. Walking exercise has a positive impact on physical fitness (6MWD – p = 0.001) and physical components of the psychological tests ([SF-36] physical functioning p < 0.05; [PSPP-SCV] functionality p < 0.05 and SWLS p < 0.05 of people with schizophrenia. The walking program enhances the quality of life and self-perception of the physical domain and physical fitness of people with schizophrenia.

### S3 Diagnosis and innovative treatments - the way to a better medical practice

#### Celeste Alves^1,2^

##### ^1^CUF Hospitals, Lisbon, Portugal; ^2^Breast Unit, Champalimaud Clinical Center, Lisbon, Portugal

The advancement of technology in diagnostic equipment was the major driving force in the advancement of medical knowledge. In few decades, imaging methods went from two planes conventional radiology to computed tomography in volumetric acquisition and multiplanar reconstruction, with morphological analysis and quantification of densities. With magnetic resonance we are able to perform the analysis of tissue composition and to identify it´s changes in context with each disease. Nowadays, molecular imaging methods enable the correlation of metabolism and diseases. The research and knowledge acquired about the genome and the development of genomics has given to the medical community the opportunity to study genetic alterations linked to a large number of diseases.

New technology allows physicians a better diagnosis of a significant number of syndromes and diseases, has seen in chronic degenerative diseases and in oncological diseases. This big technological progress is responsible for the advancement of personalized medicine and new treatments, taking into account the biological and genetic characteristics of each individual diseases. All the progress made in screening methods has also lead the medical community to a more efficient study of diseases with high prevalence in the general population.

The link between technological advancement seen today and the early diagnosis of a large number of oncological diseases as well as the development of a personalized medicine contributes not only to the well-being of each patient but also to the practice of sustainable medicine.

### S4 Simulation-based learning and how it is a high contribution

#### Stefan Gisin (stefan@gisin.net)^1,2^

##### ^1^Swiss Centre for Medical Simulation & Swiss Association of Simulation in Healthcare, 4031 Basel, Switzerland; ^2^Basel University Hospital, 4056 Basel, Switzerland

Dr. Stefan Gisin is staff anaesthesiologist at the University Hospital in Basel/Switzerland, deployed in various specialties of anaesthesia (including cardiac, thoracic, neuro, obstetric), he has a special interest in pre-hospital emergency medicine and is flying doctor for the Swiss Helicopter Rescue Service (Rega) and passionate ATLS Course Director.

Stefan Gisin is head of the Swiss Center for Medical Simulation in Basel and implemented a multitude course concepts for pre- and postgraduate simulation-based education, focussing on interdisciplinary and multiprofessional team training. In collaboration with the Swiss Department for Development and Collaboration (DEZA) he is a consultant in several healthcare education projects in Eastern European countries. He serves as faculty for international simulation instructor courses (EuSim, PAEDSIM, InFact) and is on the advisory board of PAEDSIM eV. Stefan Gisin is currently President of SASH (Swiss Association of Simulation in Healthcare) and Vice-President of SESAM (Society in Europe for Simulation applied to Medicine).

### S5 Formative research about acceptability, utilization and promotion of a home fortification programme with micronutrient powders (MNP) in the Autonomous Region of Príncipe, São Tomé and Príncipe

#### Elisabete Catarino, Nelma Carvalho, Tiago Coucelo, Luís Bonfim, Carina Silva

##### HELPO Association, 2750-318 Cascais, Portugal

###### **Correspondence:** Elisabete Catarino (elisabetecatarino@helpo.pt) – HELPO Association, 2750-318 Cascais, Portugal

Ensuring adequate micronutrient status in children is a challenge, particular in developing countries. In São Tomé e Príncipe, the Demographic and Health Survey reveals that more than half (62 %) of children aged between 6 and 59 are anaemic [1]. Documents guiding the Copenhagen Consensus [2] are very clear in demonstrating that without micronutrient status correction there is no full development of human potential, in particular the cognitive capacity [3-5]. It is thus essential that mothers know the consequences to better understand the necessity of a balanced nutrition. Since 2011, WHO [6] has been promoting the use of micronutrient powders (MNP) in countries with elevated anaemia prevalence in children, considering them as a highly recommendable public health measure in fighting this disease. In 2014, home supplementation with micronutrient powders was proposed as a National Nutrition Policy strategy in São Tomé and Príncipe [7].

The objective of this study is to describe families and communities’ reaction to the use of MNPs with emphasis on their acceptance, utilization and promotion. This formative research contributes to adjust MNP messages to the culture and preferences of the target public before the wider distribution of the MNPs.

The results of our study showed a good acceptance and correct utilization of MNP. Regarding an effective communication, participants chose the colour of the package with an image of a saotomean baby/child. The name brand “Vitaferro” was voted as most popular as it is easy to pronounce and relates easily to MNP and to anaemia. Some mothers also suggested a positive message motivating MNP use in the communication materials.

**References**

1. INE, MSAS. Macro I. “Inquérito Demográfico e Sanitário”, São Tomé e Príncipe, IDS STP 2008-2009. Calverton, Maryland, USA; 2010.

2. Hoddinott J, Rosegrant M, Torero M. “Challenge Paper on Hunger and Malnutrition”. Washington DC: Copenhagen Consensus 2012; 2012. Available in: http://www.copenhagenconsensus.com/sites/default/files/hungerandmalnutrition.pdf

3. Adu-Afarwuah S, Lartey A, Brown KH, Zlotkin S, Briend A, Dewey KG. “Home fortification of complementary foods with micronutrient supplements is well accepted and has positive effects on infant iron status in Ghana”. American Journal of Clinical Nutrition. 2008; 87(4):929-38.

4. Bilukha O, Howard C, Wilkinson C, Bamrah S, Husain F. “Effects of multi-micronutrient home fortification on anaemia and growth in Bhutanese refugee children”. Food and Nutrition Bulletin. 2011; 32(3):264-76.

5. De-Regil LM, Suchdev PS, Vist GE, Walleser S, Peña-Rosas JP. “Home fortification of foods with multiple micronutrient powders for health and nutrition in children under two years of age”. Evidence-Based Child Health. 2013; 8(1):112-201.

6. WHO. “Guideline: Use of Multiple Micronutrient Powders for Home Fortification of Foods Consumed by Infants and Children 6-23 Months of Age”. Geneva; 2011.

7. Osei A, Septiari A, Suryantan J, Hossain MM, Chiwile F, Sari M, et al. “Using formative research to inform the design of a home fortification with micronutrient powders (MNP) Programme in Aileu District, Timor-Leste”. Food and nutrition bulletin. 2014; 35(1):68-82.

**Keywords**: Children, micronutrient status, anaemia, micronutrient powders, MNP

## Session 3: Health policy & health management

### S6 Safety culture of the patient: a reflexion about the therapeutic approach on the patient with vocal pathology

#### Débora Franco

##### Centro de Linguística, Universidade de Lisboa, Lisboa, Portugal

Clinical practice raises issues pertaining to vocal quality, such as the incidence and prevalence of dysphonia and the relapses in the process of vocal rehabilitation. These issues merit a commitment of critical analysis to improve healthcare quality. The vocal use of each speaker, the existence of bad habits and the presence of other pathologies are factors which compromise vocal health and can lead to dysphonia.

It has been concluded that body composition characteristics can influence the postural adaptations that an individual adopts over time, h an impact on vocal health and glottic dynamics. Fatter individuals will have compromised vital capacity and pneumophonic coordination, increasing the irregularities of vocal vibration and decreasing the vocal intensity. These individuals can develop postural adjustments to improve the efficiency of the respiratory tract. However, a head in hyper-extension will favour jaw descent with an impact on vocal quality. The increase in cervical lordosis will increase the thoracic kyphosis and the kyphotic index, with differences having been observed between normal and dysphonic individuals for these variables.

It is believed that a greater effectiveness of the intervention, reducing the relapses, is possible if body morphology analysis is also included in the evaluation protocols, permitting: (a) an identification of risk factors of vocal pathology; (b) the forwarding to precise diagnostic exams; (c) team planning of treatment; (d) postural intervention to be included in the rehabilitation of dysphonia. Building a culture of safety of the patient is finding our mission as health professionals.

### S7 About wine, fortune cookies and patient experience

#### Jesús Alcoba González

##### Centro Superior de Estudios Universitarios La Salle, Madrid, Spain

Experience economy is the last segment in the evolution of the market, and it is characterized by the fact that consumers do not acquire goods, products or services, but experiences that they integrate in their biography, and consequently in their identity. Customer Experience, possibly the latest revolution in business thinking along with the digital transformation, seeks the design and management of truly customer-centric experiences. This revolution is spreading across different sectors, among which the health sector should necessarily be considered. This talk covers the fundamental ideas within the concept of customer experience, as well as it provides information and suggestions about how to design and deliver an optimal patient experience.

**Keywords:** Experience economy, customer experience, patient experience, health care, customer centric culture.

## Oral & poster presentations

### Acessibility to Health Care & Determinants of Demand for Health Services

#### O1 The psychological impact on the emergency crews after the disaster event on February 20, 2010

##### Helena G. Jardim^1^, Rita Silva^2^

###### ^1^Universidade da Madeira, 9000-082 Funchal, Portugal; ^2^Serviço de Saúde da Região Autónoma da Madeira, E.P.E., 9004-514 Funchal, Portugal

####### **Correspondence:** Helena G. Jardim (hjardim@uma.pt) – Universidade da Madeira, 9000-082 Funchal, Portugal

Disasters affect emergency teams to varying degrees, causing trauma. The risk factors for mental health can be determined according to the experience of professionals in coping with these situations. The gravity of the situation, as well as the behaviour and response of those present will be different, depending on the extent of the catastrophe, such as the number of deaths, destruction, duration and degree of rapidity of the phenomenon.

The purpose of the study was to assess the psychological impact on emergency crews after the natural disaster of February 20 2010, in the isle of Madeira, Portugal. The professional group was comprised of 405 individuals: firemen (41.7 %), military (32.8 %), police (17.3 %) and health professionals (8.1 %). The assessment tools used were: the list of life events and the peri-traumatic experiences questionnaire, adult version. Ethical issues were considered as well as informed consent.

The average age was 34.5 years, mostly male, married and in residence at the time of the event. The data show that there were significant differences between the profession and the response to the event, as well as in relation to Post Traumatic Stress Disorder (PTSD), this being most evident in the group of firemen and military (p < 0.001; p < 0.001).

The results suggest the vital need for specific training for these professional groups in the field of mental health, aimed at the prevention of psychological disorders.

**Keywords**

Natural Disasters, peritraumatic experiences, post-traumatic Stress Disorder

#### O2 Musculoskeletal disorders in midwives

##### Cristina L. Baixinho, Mª Helena Presado, Mª Fátima Marques, Mário E. Cardoso

###### Escola Superior de Enfermagem de Lisboa, 1700-063 Lisboa, Portugal

####### **Correspondence:** Cristina L. Baixinho (crbaixinho@esel.pt) – Escola Superior de Enfermagem de Lisboa, 1700-063 Lisboa, Portugal

**Background**

The prevalence of musculoskeletal disorders related to work (MDRW) is high in midwives. The risk factors are associated with the physical environment, the nature of the occupation, the parturient, the type of delivery and the new-born. Objective: Identifying the safety practices adopted by the midwives to prevent the MDRW.

**Methods**

Qualitative exploratory study. The inclusion criteria for the 12 participants were, the professional title of the midwives, if they work in childbirth blocks. For results analysis, Bardin’s propose of content analysis was used. The anonymity and confidentiality of the results was guaranteed.

**Results**

Concerns about equipment and materials manipulation as to prevent low back overload in the initial positioning of the parturient and the mobilization of the new-born stood out. Speech analysis revealed difficulty in adopting preventive measures for the expulsive period, because the movement of pronation supination of the upper limbs needed to facilitate the birth is done by increasing muscular tension, making it difficult to maintain alignment and body balance.

**Conclusions**

Participants report difficulties in maintaining the principles of biomechanics especially in the expulsive period caused by the complications in controlling the parturient behaviour.

**Keywords**

Musculoskeletal disorders, midwives, biomechanics

#### O3 Negative childhood experiences and fears of compassion: Implications for psychological difficulties in adolescence

##### Marina Cunha^1^, Joana Mendes^1^, Ana Xavier^2^, Ana Galhardo^1^, Margarida Couto^1^

###### ^1^Instituto Superior Miguel Torga, Coimbra, 3000-132 Coimbra, Portugal; ^2^Centro de Investigação do Núcleo de Estudos e Intervenção Cognitivo Comportamental, Universidade de Coimbra, Coimbra, 3001-802, Portugal

####### **Correspondence:** Marina Cunha (marina_cunha@ismt.pt) – Instituto Superior Miguel Torga, Coimbra, 3000-132 Coimbra, Portugal

**Background**

Adverse childhood experiences are not only linked to mental health difficulties but also to the inability to generate positive and affiliative emotions. Individuals from these adverse backgrounds may also fear and avoid compassionate feelings from others and being compassionate for others and for themselves. Objective: This study aims to test whether the impact of early negative memories in childhood on psychological difficulties is mediated by fears of compassion (for others, from others and for self).

**Methods**

The sample consists of 178 adolescents with ages between 12 and 18 years old (M = 15.53, SD = 1.96) from middle and secondary schools in the central region of Portugal. Participants answered the following self-report questionnaires: The Early Life Experiences Scale; the Fears of Compassion Scales; and the Strengths and Difficulties Questionnaire.

**Results**

Results from Path Analysis showed that the full model accounted for 36 % of the psychological difficulties among adolescents. The recall of threatening, subordination and devaluation experiences in childhood with parents had a direct impact on psychological difficulties. These early adverse experiences also had an indirect effect in psychological difficulties through both fears of compassion from others and for self. A multi-group analysis for gender was tested and the meditational model was equivalent for both genders.

**Conclusions**

These findings suggest that addressing adverse emotional memories in childhood and fears of compassion (in others and self-to-self relationships) may be a valuable approach for preventive and intervention actions in order to promote psychological adjustment in youths.

**Keywords**

Adolescence, psychological difficulties, negative emotional memories, fear of compassion, Path analysis

#### O4 Optimal age to give the first dose of measles vaccine in Portugal

##### João G. Frade^1^, Carla Nunes^2^, João R. Mesquita^3^, Maria S. Nascimento^4^, Guilherme Gonçalves^5^

###### ^1^Escola Superior de Saúde de Leiria & Unidade de Investigação em Saúde, Instituto Politécnico de Leiria, 2411-901 Leiria, Portugal; ^2^National School of Public Health & Centro de Investigação em Saúde Pública, Universidade Nova de Lisboa, 1600-560 Lisboa, Portugal; ^3^Agrarian Superior School, Polytechnic Institute of Viseu, 3500-606 Viseu, Portugal; ^4^Laboratory of Microbiology, Department of Biological Sciences, Faculty of Pharmacy, University of Porto, 4050-313 Porto, Portugal; ^5^Unit for Multidisciplinary Biomedical Research, Institute of Biomedical Sciences Abel Salazar, University of Porto, 4050-313 Porto, Portugal

####### **Correspondence:** João G. Frade (joao.frade@ipleiria.pt) – Escola Superior de Saúde de Leiria & Unidade de Investigação em Saúde, Instituto Politécnico de Leiria, 2411-901 Leiria, Portugal

**Background**

The optimum age to give the first dose of the combined measles-mumps-rubella vaccine (MMR) must balance the risk of disease and the age of specific immunity induced by the vaccine.

**Methods**

A seroepidemiological study was conducted with 206 newborns, in the Obstetric service of a Hospital of the District of Leiria. The study followed all ethical procedures and was conducted under the approval of the ethical committee of the Hospital. At birth, a cord serum sample was collected. Measles antibodies were measured in cord sera, using Siemens Enzgnost’s immunoassay. Maternal vaccination history was ascertained through the analysis of individual records available at ACES Pinhal Litoral, Portugal.

**Results**

Among participants, 54 mothers had never been vaccinated with MMR, 62 had one dose and 90 had two doses. The concentration of measles antibody increased with the age of the mother (r^2^ = 0.097, p = 0.001) and was the highest in the newborns of mothers who had never been vaccinated against measles (μ = 1906, IC 95 % = 1194-2857 mUI/mL; p < 0.001). According to a hypothetical model, and to the level of measles antibody concentrations found at birth, 95 % of the children at 12 months of age should still have a title of measles antibody above 40 mUI/mL.

**Conclusions**

Giving the first dose of MMR at 12 months of age, would be a good decision if the half-life of measles antibodies in infants would be 44 days or less. Above that value, could be to compromise the effectiveness of the immune response.

**Keywords**

Measles, vaccine, Portuguese population

#### O5 Functional assessment of elderly in primary care

##### Conceição Castro^1^, Alice Mártires^2^, Mª João Monteiro^2^, Conceição Rainho^2^

###### ^1^Agrupamento de Centros de Saúde (ACES) Baixo Mondego, 3080-199 Figueira da Foz, Portugal; ^2^Universidade de Trás-os-Montes e Alto Douro, Vila Real, 5001-801, Portugal

####### **Correspondence:** Conceição Rainho (crainho@utad.pt) – Universidade de Trás-os-Montes e Alto Douro, Vila Real, 5001-801, Portugal

**Background**

The aging process is multifactorial and progressive. A major goal in this process is to be active, in order to maintain independence and continue as long as possible an active life. Objectives: To evaluate the functional independence in activities of daily living of elderly; and analyse the scoring in daily activities in relation to socio-demographic variables.

**Methods**

A correlational study with elderly, registered in a health centre of central Portugal. It was used the Barthel index (10 items) to estimate the functional independence in activities of daily life. The score ranges from zero to hundred. The Spearman correlation was used, with a statistically significant level of α = 0.05.

**Results**

A total of 62 elders were assessed, 34 (54.8 %) of them women, with a mean age of 77.2 ± 7.5 years. The Barthel Index showed high internal consistency (Cronbach's alpha = 0.846). The average Barthel Index was 95.5 ± 10.2, most have a higher level of independence, however the elderly had more dependency for daily activities: bathing and using the toilet. The relationship between age and daily activities of the elderly was significant on the total score of the Barthel Index. The age and the level of independence shown to be statistically associated.

**Conclusions**

The results show the reliability of the Barthel Index. The age has been identified as the key factor and therefore is consistent with the results of other studies. This study may contribute to identify the factors that will promote the degree of independence.

**Keywords**

Elderly, life, activities

#### O6 Smoking and coronary events in a population of Spanish health-care centre: An observational study

##### Francisco P. Caballero^1^, Fatima M. Monago^2^, Jose T. Guerrero^3^, Rocio M. Monago^4^, Africa P. Trigo^4^, Milagros L. Gutierrez^3^, Gemma M. Milanés^3^, Mercedes G. Reina^1^, Ana G. Villanueva^5^, Ana S. Piñero^6^, Isabel R. Aliseda^3^, Francisco B. Ramirez^1^.

###### ^1^Centro de Salud La Paz, Badajoz, 06011, España; ^2^Centro de Salud San Fernando, Badajoz, 06006 Badajoz, España; ^3^Complejo Hospitalario Universitario de Badajoz, Badajoz, 06080 Badajoz, España; ^4^Hospital Don Benito-Villanueva, Don Benito, 06400 Badajoz, España; ^5^Centro de Salud Villanueva Norte, Villanueva de la Serena, 06700 Badajoz, España; ^6^Centro de Salud Gevora, 06180 Gévora, Badajoz, España

####### **Correspondence:** Francisco P. Caballero (franciscoluisper@gmail.com) – Centro de Salud La Paz, Badajoz, 06011, España

**Background**

Smoking is a major risk factor for multiple chronic diseases, such as cardiovascular diseases and cancer, and an established risk factor for premature death. Objective. To analyse the association between smoking and total coronary risk (incidence of lethal and non-lethal coronary events) in a cohort of 35-74 year-old patients followed for 10 years.

**Methods**

Longitudinal, observational study of a retrospective cohort followed for ten years in primary care practices in Badajoz (Spain). A cohort of 968 patients (mean 55.7 year-old; 57.5 % women) without evidence of cardiovascular disease was studied. Smokers were defined as those who consumed any amount of tobacco daily during the year before, or those who declared cessation of smoking within less than one year.

**Results**

A 25.9 % of the patients were smokers (47.4 % of male and 10.2 % of female patients). Smokers were younger (51.5 vs 57.2 years, p < 0.001), with less prevalence of arterial hypertension (45.4 % vs 61.5 %, p < 0.01), and lower HDL-cholesterol (45.5 vs 54.0 mg/dl, p < 0.001). During the follow-up, they presented a higher mortality, myocardial infarction events, and more coronary episodes (14.7 % vs 9.2 %, p < 0.05). The final model of the logistic regression multivariate analysis revealed that only smoking and age are predictor variables of total coronary events, the greater odds ratio corresponding to smoking (OR: 2.33; IC95%; 1.31-4.16; p < 0.01).

**Conclusions**

In patients aged 35-74 years followed during 10 years, smoking doubles the risk of total coronary events. Primary health care and general practitioners must keep their commitment to investigate and search strategies that facilitate smoking cessation.

**Keywords**

Smoking, smoking and gender, cardiovascular risk factors, cardiovascular disease, primary health care

#### O7 Prevalence of musculoskeletal injuries in Portuguese musicians

##### Andrea Ribeiro, Ana Quelhas, Conceição Manso

###### Universidade Fernando Pessoa, Porto, 4249-004, Portugal

####### **Correspondence:** Andrea Ribeiro (andrear@ufp.edu.pt) – Universidade Fernando Pessoa, Porto, 4249-004, Portugal

**Background**

Quantify the prevalence of musculoskeletal disorders (MSDs) in musicians due by posture. The goal was also to measure the intensity of pain in different body parts and the influence of playing a certain musical instrument has on pain.

**Methods**

The sample used in this study consisted of all musicians of the Philharmonic Society of Crestuma and the Musical Band of Avintes, each constituted by fifty (50) musicians. All elements completed an individual questionnaire about individual and work factors and then the Nordic Musculoskeletal Questionnaire (NMQ).

**Results**

Neck, shoulders, wrists/hands, lumbar spine were main areas where the musicians complained about pain. Drummers were the ones who reported more intense pain in shoulders, wrists/hands and lumbar spine. It was also observed that women had higher pain intensities while compared to men. The pain seems to decrease with years of practice, except in what concerns the lower back.

**Conclusions**

This study concluded that there is a high prevalence of musculoskeletal disorders (MSDs) in musicians and the lumbar and cervical spine, shoulders and wrists/hands were the most affected areas.

**Keywords**

Prevalence, musculoskeletal disorders, pain, musicians

#### O8 Hip fractures, psychotropic drug consumption and comorbidity in patients of a primary care practice in Spain

##### Francisco P. Caballero^1^, Jose T. Guerrero^2^, Fatima M. Monago^3^, Rafael B. Santos^4^, Nuria R. Jimenez^1^, Cristina G. Nuñez^1^, Inmaculada R. Gomez^1^, Mª Jose L. Fernandez^1^, Laura A. Marquez^3^, Ana L. Moreno^2^, Mª Jesus Tena Huertas^1^, Francisco B. Ramirez^1^

###### ^1^Centro de Salud La Paz, Badajoz, 06011, España; ^2^Complejo Hospitalario Universitario de Badajoz, Badajoz, 06080 Badajoz, España; ^3^Centro de Salud San Fernando, Badajoz, 06006 Badajoz, España; ^4^Universidad de Extremadura, Badajoz, 06071 Badajoz, España

####### **Correspondence:** Francisco P. Caballero (franciscoluisper@gmail.com) – Centro de Salud La Paz, Badajoz, 06011, España

**Background**

Hip fractures are a major health problem in elderly people, with great impact on their quality of life and life expectancy, beyond increasing dependence and institutionalization. Psychotropic drugs have been associated with multiple health problems, and they increase the risk of falls, which can cause fractures. Objectives: To analyse the risk factors for hip fracture in the population of a primary care practice in Badajoz (Spain), and the possible association between hip fractures and chronic use of psychotropic drugs.

**Methods**

Descriptive, observational study of 73 patients diagnosed with hip fracture between 2007-2013 (75.3 % women; 24.7 % men), with clinical history in the primary care practice.

**Results**

The mean age of the patients was 78.3 ± 12.6 years (79.5 % ≥ 70 years; 16.4 % ≥ 90 years), all women. 67.1 % had high blood pressure, 43.8 % cardiovascular diseases, 38.4 % dyslipidaemia, 30.1 % musculoskeletal diseases and 21.9 % some kind of visual impairment. Medication with psychotropic drugs was registered in 50.7 %, highlighting an important prescription among women (58.2 % vs 27.8 %, p < 0.05), mainly benzodiazepines (47.3 % vs 22.2 %, p = 0.053). However, only 19.2 % had picked up on their history a diagnosis to justify such a prescription.

**Conclusions**

Patients with hip fracture were women, with a mean age of 80.9 years and a register of high consumption of psychotropic drugs (58.2 %), particularly benzodiazepines (47.3 %). These results encourage a judicious prescription of psychotropic drugs in general, and benzodiazepines in particular, with limited duration of treatments, as recommended by the clinical practice guidelines.

**Keywords**

Hip fracture, Psychotropic drugs, Benzodiazepines, Prescription

#### O9 The role of self-criticism and shame in social anxiety in a clinical SAD sample

##### Daniel Seabra, Mª Céu Salvador

###### Faculty of Psychology and Education Sciences, University of Coimbra, Coimbra, 3001-802 Coimbra, Portugal

####### **Correspondence:** Daniel Seabra (daniel_seabra_@hotmail.com) – Faculty of Psychology and Education Sciences, University of Coimbra, Coimbra, 3001-802 Coimbra, Portugal

**Background**

Social Anxiety Disorder (SAD) is characterized by a marked fear or anxiety in situations where the individual might be exposed to the possible scrutiny by others. Post-Event Processing (PEP), which refers to a post-mortem rumination wherein the subject reviews critically and with detail what went wrong in the social event, is considered an important maintenance factor of SAD. Clinical practice seems to indicate that rumination in patients with SAD, is mainly a self-critical process frequently resulting in feelings of shame.

Aims: This study aimed to bridge the gap between cognitive variables (PEP) and evolutionary variables (Self-Criticism and Internal/External Shame) in understanding Social Anxiety (SA), exploring the mediator role of these evolutionary variables in the relationship between PEP and SA in a clinical sample of patients with SAD.

**Methods**

The sample was constituted by 32 subjects with SAD – 25 females (78.10 %) and 7 males (21.90 %) – with an average age of 26.78 (SD = 9.22), that filled several self-report instruments and answered a diagnostic interview.

**Results**

Self-Criticism and Internal Shame fully mediated the relationship between PEP and SA. However, Sobel test only supported the full mediation of Internal Shame.

**Conclusions**

These results suggest that SA does not directly depend on PEP levels but from Internal Shame levels. In other words, Internal Shame is the mechanism through which PEP impacts on SA. Therefore, Internal Shame seems to be an important health indicator to consider in the intervention with this population. Limitations and clinical implications will be discussed.

**Keywords**

Social Anxiety Disorder, Post-Event Processing, Shame, Self-Criticism, Mediation

#### O10 Obstruction and infiltration: a proposal of a quality indicator

##### Luciene Braga^1^, Pedro Parreira^2^, Anabela Salgueiro-Oliveira^2^, Cristina Arreguy-Sena^3^, Bibiana F. Oliveira^4^, Mª Adriana Henriques^5^

###### ^1^Universidade Federal de Viçosa, Viçosa - Minas Gerais, 36570-900, Brasil; ^2^Escola Superior de Enfermagem de Coimbra, 3046-851 Coimbra, Portugal; ^3^Universidade Federal de Juiz de Fora, Juiz de Fora – Minas Gerais, 36036-330, Brasil; ^4^ Centro Hospitalar e Universitário de Coimbra, 3000-075 Coimbra, Portugal; ^5^Escola Superior de Enfermagem de Lisboa, 1700-063 Lisboa, Portugal

####### **Correspondence:** Luciene Braga (lucienemunizbraga@yahoo.com.br) – Universidade Federal de Viçosa, Viçosa - Minas Gerais, 36570-900, Brasil

**Background**

The quest for continuous improvement of the quality of provided care is the objective of nursing care. However, the insertion and permanence of a peripheral venous catheter has been associated to complications, thus making a systematic evaluation of the performance of professionals and the management of health services important. Objective: Analyse complications that caused removal of intravenous catheters.

**Methods**

A prospective study with 64 patients of a health service of Portugal, from July to September/2015. Included patients with age 18 years, with a peripheral venous catheter. Descriptive analysis using SPSS. Ethical requirements were met.

**Results**

Two hundred three (203) intravenous catheters, in 64 patients, most elderly (section 95.3 %), with mean age of 80 years were evaluated. The catheters remained inserted between one and 12 days (mean 2 days), 66 % of the devices were removed because of complications, such as: removal by the patient (17.7 %), obstruction (17.2 %), infiltration (14.8 %), phlebitis (9.4 %) and fluid exiting the insertion site (6.4 %). The prevalence of obstruction and infiltration per patient was respectively 36 % and 39 %.

**Conclusions**

Obstruction and infiltration were the complications of higher prevalence that led to the removal and reinsertion of a new peripheral venous catheter with the possibility of increased pain, infection and hospital costs. Faced with the risk of compromising patient safety and being able to contribute to the improvement of health care, we suggest the inclusion of obstruction and infiltration in the indicators of quality of care, in order to have systematic evaluation of results, (re)plan and implement preventive measures.

**Keywords**

Catheterization Peripheral, Complication, Nursing, Patient Safety, Quality Indicators Health Care

#### O11 Balance and anxiety and depression symptoms in old age people

##### Joana Santos, Sara Lebre, Alda Marques

###### School of Health Sciences, University of Aveiro, 3810-193 Aveiro, Portugal

####### **Correspondence:** Joana Santos (joanacarvalhosantos@ua.pt) – School of Health Sciences, University of Aveiro, 3810-193 Aveiro, Portugal

**Background**

Falls have high incidence in elderly and are major responsible for accidental deaths. People with high depression and anxiety symptoms have impaired balance and this is more problematic in the elderly population. However, it is unknown how anxiety and depression symptoms affect the different systems responsible for balance and balance confidence. Objective: Explore balance systems and balance confidence differences between elderly with presence/absence of anxiety and depression symptoms.

**Methods**

A quantitative cross-sectional study was conducted. Socio-demographic, anthropometric and general clinical data were collected with a structured questionnaire. Balance confidence was evaluated with Activities-specific Balance Confidence (ABC) and balance with Balance Evaluation System Test (BESTest) and Berg Balance Scale (BBS). The level of significance was set at p < .05.

**Results**

One hundred thirty-six (136) elderly (75.9 ± 8.8 years old; n = 96 female), participated in this study. All BESTest sections were significantly affected by the presence of anxiety or depression symptoms (p < 0.001). Similar results were observed in BBS (p < 0.001). The Reactive section presented the larger difference between present/absence of anxiety (49.4 ± 21.1 vs 84.2 ± 14.9; p < 0.001) and depression (46.3 ± 30.3 vs 88.5 ± 15.3; p < 0.001) symptoms. Participants’ balance confidence also decreased significantly in the presence of both symptoms (anxiety: p = 0.010; depression: p = 0.001). The severity of both symptoms influenced significantly the balance (BBS (anxiety: p = 0.013; depression: p = 0.029); BESTest (0.001 < p > 0.046)) but not the balance confidence (anxiety: p = 0.516; depression: p = 0.274).

**Conclusions**

The presence of anxiety and of depression symptoms significantly decrease balance performance and balance confidence in the elderly. The severity of symptoms significantly decreases balance performance but does not seem to significantly impact on balance confidence.

**Keywords**

Balance, old age people, anxiety, depression

#### O12 Prevalence of postural changes and risk factors in school children and adolescents in a northern region (Porto)

##### Clarinda Festas, Sandra Rodrigues, Andrea Ribeiro, José Lumini

###### Universidade Fernando Pessoa, 4249-004 Porto, Portugal

####### **Correspondence:** Clarinda Festas (clarinda@ufp.edu.pt) – Universidade Fernando Pessoa, 4249-004 Porto, Portugal

**Background**

Postural changes acquired during childhood and adolescence are a risk factor for disorders of the spine in adulthood and may become irreversible if not treated in time. The purpose of this study was to evaluate the postural changes among students of basic schools in the district of Porto.

**Methods**

Weight and height were measured. The "body chart" and visual analogue scale for pain and static postural form for evaluation of postural changes, backpack characteristics and additional physical activity questionnaire were used.

**Results**

This study included 285 students, aged 11.46 years ± 1.32 years, and weighing 43.36 kg ± 10.49 kg and with a height of 1.48 m ± 0.95 m. Use of backpack: 97.5 % of the participants, 83.0 % use it in both shoulders and 17.0 % only on one shoulder. In relationship to physical activity 58.0 % do it after regular school time, the most prevalent physical activity was swimming with 21.3 % and 23.1 % for football. Pain lifetime prevalence was 43.0 %. With regard to postural changes, elevation of the shoulders was the most prevalent (78.0 %), changes in iliocostalis angle (52.5 %) and flat feet (47.2 %), calcaneus valgus (37.7 %) and scoliosis (25.5 %), anterior head projection (63.0 %), anterior shoulder (45.2 %), pelvic anteversion (37.7 %), lumbar hyperlordosis (56.9 %) and knee recurvatum (24.2 %).

**Conclusions**

We conclude that postural changes found are according the postural characteristics for this age group and identification of changes allowed earlier clinical referral and propose school postural education prevention programmes.

**Keywords**

Postural changes, school children and adolescents, school health, backpack, pain

#### O13 Ischemic stroke vs. haemorrhagic stroke survival rate

##### Ana G. Figueiredo (ritagfigueiredo@gmail.com)

###### ^1^Escola Superior de Saúde Dr. Lopes Dias, Instituto Politécnico de Castelo Branco, 6000-767 Castelo Branco, Portugal

**Background**

Stroke is the main cause of death in Portugal and it’s also one of the pathologies involved in several physical and psychological comorbidities and which often prevents stroke patients from having an autonomous life. There are still several gaps related to the prognosis of the two different types of stroke (ischemic and haemorrhagic) and is relevant to know which type has the better longevity. Objective: The main objective of the present study is to evaluate the survival rate of both types of stroke, and simultaneously the mortality risk in those patients.

**Methods**

A sample of 1367 individuals who suffered a stroke was collected in Hospital do Espírito Santo de Évora, between 2010 and 2015, after that the sample’s profile, risk factors and associated comorbidities were studied. Then, we created a subgroup from the initial sample with 311 individuals who suffered both types of stroke in the period 2013-2014 and performed a statistical analysis of survival and mortality risk.

**Results**

Individuals who suffered ischemic stroke have a lower mortality rate, as well as an improved initial survival; Three months after the event, the mortality risk becomes independent of the stroke type. We also observed that age has an effect on survival.

**Conclusions**

Haemorrhagic stroke is associated with an increased mortality and worse survival rate compared with ischemic stroke.

**Keywords**

Ischemic stroke, haemorrhagic stroke, survival, mortality

#### O14 Chronobiological factors as responsible for the appearance of locomotor pathology in adolescents

##### Francisco J. Hernandez-Martinez^1^, Liliana Campi^2^, Mª Pino Quintana-Montesdeoca^3^, Juan F. Jimenez-Diaz^3^, Bienvenida C. Rodriguez-De-Vera^3^

###### ^1^Cabildo de Lanzarote, 35500 Arrecife, Lanzarote, Las Palmas, España; ^2^Servicio Canario de la Salud, 35500 – Arrecife, Lanzarote, Las Palmas, España; ^3^Universidad de Las Palmas de Gran Canaria, 35001 Las Palmas de Gran Canaria, Las Palmas, España

####### **Correspondence:** Francisco J. Hernandez-Martinez (fjhernandez@denf.ulpgc.es) – Cabildo de Lanzarote, 35500. Arrecife, Lanzarote, Las Palmas, España

**Background**

Chronobiology is a discipline that studies the biological processes following predictable temporal sequences, focusing on the analysis of circadian rhythms and biological rhythms associated with the geophysical basis. Any physiological function of living things can be analysed from the perspective of chronobiology as a phenomenon with a significant temporal and rhythmic dimension. Objective: To determine whether the existence of chronobiological factors and circadian variations involved in the development of locomotives diseases in adolescence.

**Methods**

Observational, descriptive, retrospective and quantitative study on the adolescent population of Lanzarote (Canary Islands) from 2010 to 2015 Study. Data collection: computer system Drago. Statistical Analysis with SPSS 23.0 software.

**Results**

On a sample of 1,214 adolescents it was found a significant association (p < 0.023) between gender, seasonal time and location of the lesion, being more frequent in males 11-16 years, most of them in autumn and winter and being the lower limbs in men (56 %) and arms in women (42 %), the most affected areas. Highlighting the lack of medical records in medical records regarding the etiology of the lesion (37.2 %) and time of occurrence (89 %).

**Conclusions**

The teenage gender is a trigger factor in musculoskeletal disorders. The prevalence of these diseases is higher in urban areas. The prevalent etiology is trauma. The climate and geographical location of Lanzarote, significantly influence the etiology and location of the lesion. We should prepare educational programs on risk prevention at schools.

**Keywords**

Chronobiology, adolescence, Lanzarote, locomotor pathology

#### O15 Risk of malnutrition in the elderly of Bragança

##### Alexandra Parente, Mª Augusta Mata, Ana Mª Pereira, Adília Fernandes, Manuel Brás

###### Escola Superior de Saúde, Instituto Politécnico de Bragança, 5300-121 Bragança, Portugal

####### **Correspondence:** Manuel Brás (manuel-bras@ipb.pt) – Escola Superior de Saúde, Instituto Politécnico de Bragança, 5300-121 Bragança, Portugal

**Background**

Malnutrition affects a significant number of people, the elderly being the main group affected. Retrospective studies highlight that the elderly are at higher risk and have higher susceptibility of nutritional deficiencies than young adults. Objective: To assess the risk of malnutrition in the elderly of Bragança.

**Methods**

Analytical and cross-sectional study. Based on a sampling error under 5 % and a confidence level of 95 %, a sample of 385 elderly people stratified by gender and age was studied. A questionnaire was used including nutritional risk assessment using the Mini Nutritional Assessment (MNA).

**Results**

56.4 % (217) are women and 43.6 % (168) are men with an average of 76.39 ± 7.18 years of age. The majority (62.9 %; 242) is married/civil partnership and 28.6 % (110) are widowers. 37.8 % (134) relate some degree of loneliness. We also noticed that 96 elderly (24.9 %) show risk of malnutrition. Those who are married/in a civil partnership have higher probability of normal nutritional condition when compared with single or divorced (OR = 2.925); those with schooling have higher probability of normal nutritional condition (OR = 2.287) compared to those without schooling. The ones who mention less loneliness present 1.5 to 4.7 higher probabilities of normal nutritional condition compared with the ones who state any level of loneliness. The ones who are functionally independent have higher probability of a normal nutritional state than those who have any degree of dependency.

**Conclusions**

Results highlight the need for specific protocols in health institutions to identify old people at nutritional risk allowing a timely intervention.

**Keywords**

Elderly, malnutrition, risk

#### O16 A Lifestyle Educational Programme for primary care diabetic patients: the design of a complex nursing intervention

##### Mª Rosário Pinto^1^, Pedro Parreira^2^, Marta L. Basto^3^, Ana C. Rei^4^, Lisete M. Mónico^5^

###### ^1^Escola Superior de Saúde de Santarém, Santarém, 2005-075 Portugal; ^2^Escola Superior de Enfermagem de Coimbra, Coimbra, 3046-851 Portugal; ^3^Escola Superior de Enfermagem de Lisboa, Lisboa, 1700-063 Portugal; ^4^Hospital Distrital de Santarém, EPE, Santarém, 2005-177 Portugal; ^5^Universidade de Coimbra, 3004-504 Coimbra, Portugal

####### **Correspondence:** Mª Rosário Pinto (mrosariopinto.essaude@gmail.com) – Escola Superior de Saúde de Santarém, Santarém, 2005-075 Portugal

**Background**

Therapeutic Education is structural to diabetic people’s self-control. Although these questions have been a research theme of concern over the past years, there is a shortage of tested educational programmes that allow discussion of its effectiveness. Objective: To design a lifestyle educational programme for diabetic patients.

**Methods**

An exploratory and descriptive approach was made, starting by characterizing and understanding the context – a Primary Care Unit, as well as the usual educational intervention developed. Data collection included semi-directive interviews, observational moments and record analysis. Supported by a theoretical framework (Orem’s Self-care Theory and Empowerment perspective), information was analysed and discussed with the nurses, in group and individual interactions, from July until October 2014.

**Results**

The result was a Lifestyle Educational Programme directed to people with type 2 diabetes, focused on lifestyle and self-control behaviours, to be implemented by primary care nurses. With a 24-week timeline, the programme starts with a face-to-face nursing intervention, followed by two educational group sessions, the first one focussed on self-motivation and lifestyle generic behaviours, and the second one centred on foot self-care and monitoring. At 12 weeks, the second face-to-face intervention is done, complemented by a telephone monitoring intervention after four weeks, ending with the last individual moment, 24 weeks after the beginning of the programme.

**Conclusions**

The programme developed is a Complex Intervention, with several components, including the intervention usually carried out by nursing professionals, in addition to which is added group and telephone intervention, merged in an educational protocol that follows specific sequential phases.

**Keywords**

Therapeutic Education, Type 2 Diabetes, Lifestyle Educational Program, Nursing education

#### O17 Medication adherence in elderly people

##### Gilberta Sousa, Clementina Morna, Otília Freitas, Gregório Freitas, Ana Jardim, Rita Vasconcelos

###### Universidade da Madeira, 9000-082 Funchal, Portugal

####### **Correspondence:** Gilberta Sousa (gfranca@uma.pt) – Universidade da Madeira, 9000-082 Funchal, Portugal

**Background**

Adherence to medication regimens is an indicator of health service efficiency (WHO, 2003). It promotes improvements in clinical status, safety and quality of life of individuals and leads to better financial results, reflected by reduced use of health services and decreases the risk of aggravation of disease and acute crises caused by inadequate drug management. Objective: Describe the level and factors associated with the adherence to medication regimens in the elderly.

**Methods**

From a research-action perspective, we developed a descriptive, quantitative and transversal study (2014), with random and accidental sample in a Madeira Island parish (n = 493) using the questionnaire “Measure Adherence to Treatment- MAT” validated for the Portuguese population (α = 0.74).

**Results**

Mean age 70 years (σ ± 7.1); 68 % female; 70 % have 4 years of education; 40 % consume 5 or more different drugs/day; with a mean of adherence of 5.4(6). The main factors that influence adherence are the problems of memory (40 %); In the MAT subscales 23.5 % non-adherence was found; 13.5 % non-adherence by excess; 18.0 %-non-adherence by deficit and 14.1 % other causes. In the subscale of non-adherence, the values observed were: 33.0 % failure to take the medication; 30.5 % the neglect in the time of taking; 16.8 % abandonment because they feel better and 15.8 % because they feel worse.

**Conclusions**

The results corroborate the findings of other studies and express the priorities for action in this context. In partnership, specific objectives were outlined and nursing interventions designed to enhance patient adherence to medication prescriptions.

**Acknowledgements**

Authors wish to acknowledge the helpful collaboration given to this study by the Nursing students.

**Keywords**

Adherence to medication regimen, elderly, nursing intervention

#### O18 Hospitalization for cervical cancer of residents in the metropolitan region of Porto Alegre, Southern Brazil, 2012 to 2014

##### Lina G. Horta, Roger S. Rosa, Luís F. Kranz, Rita C. Nugem, Mariana S. Siqueira, Ronaldo Bordin

###### Federal University of Rio Grande do Sul, Porto Alegre, Rio Grande do Sul, 90050-170, Brasil

####### **Correspondence:** Roger S. Rosa (roger.rosa@ufrgs.br) – Federal University of Rio Grande do Sul, Porto Alegre, Rio Grande do Sul, 90050-170, Brasil

**Background**

Cervical cancer is caused by persistent infection due to certain oncogenic types of human papillomavirus (HPV). These changes of the cells are easily discovered on the Pap test and are curable in almost all cases. Objective: To describe the characteristics of hospitalizations for cervical cancer in the public health system by residents of the Greater Porto Alegre (GPA), in southern Brazil, 2012 to 2014.

**Methods**

Analysis of hospitalizations with a primary diagnosis ICD-10 C53 from the Hospital Information System (HIS)/SUS publicly available. Calculation of indicators by age, stay, mortality and hospitalization for spending.

**Results**

There were 2,143 hospitalizations (714.3/year) in the public health system for cervical cancer of the GPA residents (3.4/10,000 inhabitants/year). Hospitalizations and deaths of patients up to 44 years represented 41.0 % and 27.9 % respectively. The average length of stay was 7.4 days. Thirty-two (1.5 %) patients needed to use Intensive Care Unit (ICU). The mortality rate reached 8.5 % with 183 deceased patients (61/year). The average annual expenditure was $ 657,0 thousand PPP (Purchasing Power Parity) and the average value per hospitalization $ 919,78 PPP.

**Conclusions**

Hospitalization for and death from cervical cancer have occurred in young women in GPA and it could be prevented if more emphasis was given to preventive tests.

**Keywords**

Women, cervical cancer, hospitalization, primary care sensitive conditions, public health system

#### O19 Oncologic assistance of high complexity: evaluation of regulating accesses

##### Rosiane Kniess, Josimari T. Lacerda

###### Universidade Federal de Santa Catarina, Florianópolis, 88040-900 Brasil

####### **Correspondence:** Rosiane Kniess (rosik29@gmail.com) – Universidade Federal de Santa Catarina, Florianópolis, 88040-900 Brasil

In many countries, health systems are guided by the principle of equal access, which becomes a major challenge when it comes to high-magnitude diseases, such as cancer. In Brazil, this is considered as a public health problem due to its high incidence, mortality and difficulty in accessing diagnosis and treatment. This research study aimed at analysing the regulation of access to highly complex cancer care in a Brazilian State, considering the normative criteria established under the Unified Health System, the care model structure, and integrated and coordinated network services. The research involved two stages: design of the evaluation model and case study. The evaluation model consisted in 14 indicators, grouped into two dimensions - working condition and adequacy of regulation - and was agreed by experts in consensus workshops. Data were collected through direct observation, interviews with managers and professionals from the State Regulatory Agency, and the review of information and documents from the regulatory sector data banks. The research findings show that the performance of the Regulatory System in regulating the access to highly complex cancer care in the State under study is being implemented, although its ability to meet the patients’ health needs falls short of its potential. There are weaknesses in the implementation and adoption of Information and Regulation Systems and in the availability of information with the potential to help the State manager’s decision-making in highly complex cancer care.

**Keywords**

Evaluation, Regulation, Access Oncology

#### O20 Perceived barriers for using health care services by the older population as seen by the social sector: findings from the Vila Nova de Gaia Gerontological Plan

##### Joana Guedes, Idalina Machado, Sidalina Almeida, Adriano Zilhão, Helder Alves, Óscar Ribeiro

###### Instituto Superior de Serviço Social do Porto, 44600-362 Sra. da Hora, Portugal

####### **Correspondence:** Joana Guedes (joana.guedes@isssp.pt) – Instituto Superior de Serviço Social do Porto, 44600-362 Sra. da Hora, Portugal

**Background**

Access to health services means the timely use of personal health services to achieve the best health outcomes. Several studies have identified a multiplicity of impediments to health services accessibility by older adults including cost, cultural barriers and physical access barriers. Objectives: This study takes part of a Gerontological Plan currently being conducted in the Vila Nova de Gaia (VNG) Municipality. It focuses on the main difficulties observed in the older population as perceived by professionals who assume a pivotal role in the coordination of social care services at a parish level.

**Methods**

Twenty interviews were conducted and data was analysed based on thematic content.

**Results**

Access to health care services was found to be an important difficulty, namely due to the costs/affordability of health care visits and to the lack/costs of transportation particularly for seniors living in rural areas. Problems related to family care provision and medical care compliance, low incomes and the absence of specialized services (e.g. neurocognitive disorders) were also frequently mentioned constraints.

**Conclusions**

Findings are in line with the last WHO World Health Survey, which highlights that in low and lower-middle income countries the greatest barriers that many older people face in accessing health care appear to arise from the cost of the health-care visits and transportation. These findings raise important questions for defining and implementing policies aimed at meeting the needs of the older population and have to be further complemented with the perspective of the older adults themselves and their experiences as patients.

**Keywords**

Older adults, accessibility to health care, healthy aging, gerontological plan

#### O21 Sleep difficulties and depressive symptoms in college students

##### Ana P. Amaral, Ana Santos, Joana Monteiro, Mª Clara Rocha, Rui Cruz

###### Escola Superior de Tecnologia da Saúde de Coimbra, São Martinho do Bispo, 3046-854 Coimbra, Portugal

####### **Correspondence:** Ana P. Amaral (apamaral.22@gmail.com) – Escola Superior de Tecnologia da Saúde de Coimbra, São Martinho do Bispo, 3046-854 Coimbra, Portugal

**Background**

Usually sleep disturbances often precede depression, being an independent risk factor for a first episode or recurrence of depression [1]. Young adults beginning college are entering a new phase with new social contexts, demanding classes and variable sleep schedules. These significant lifestyle changes are associated with lower sleep quality [2] and with a higher prevalence of psychological distress and depression [3]. Objectives: I) To study the prevalence of sleep difficulties and depressive symptoms in college students and II) To analyse the relationship between these two variables.

**Methods**

Seven hounded seventy-six (776) Portuguese students (64.3 % females) filled in the Beck Depressive Inventory II (BDI II) [4] and an adaptation of a sleep self-response questionnaire [5].

**Results**

In the present sample 49.6 % students reported sleep difficulties, which varied according to gender and age (p < 0.05). The prevalence of difficulty initiating sleep was 25.4 %, of maintaining sleep was 20.5 % and of early morning awakening was 30.2 %. The prevalence of depression was 17.4 %, highlighting the somatic dimension. Strongest correlations were observed between sleep difficulties and depression (r = 0.332; p < 0.001).

**Conclusions**

The results show a high prevalence of sleep difficulties associated with depressive symptoms. It was found that approximately 1/5 of the analysed students had depression. These results underline the importance of developing prevention programs in higher education for healthy sleep in order to promote better mental health.

**References**

1. Espie CA, Bartlett DJ. Chapter 142 Insomnia. International Neurology: A Clinical Approach. Edited by Robert P. Lisak, Daniel D. Truong, William M. Carroll, and Roongroj Bhidayasiri. 2009 Blackwell Publishing, ISBN: 978-1-4051-5738-4.

2. Kloss, Nash, Horsey, & Taylor (2011). The Delivery of Behavioral Sleep Medicine to College Students. Journal of Adolescent Health, 48 (6): 553-561. DOI: http://dx.doi.org/10.1016/j.jadohealth.2010.09.023

3. Christensson et al. Change in depressive symptoms over higher education and professional establishment - a longitudinal investigation in a national cohort of Swedish nursing students. BMC Public Health, 2010: 343 http://www.biomedcentral.com/1471-2458/10/343.

4. Beck, A.T., Steer, R.A., & Brown, G.K. Manual for Beck Depression Inventory-II. San Antonio, TX: Psychological Corporation; 1996.

5. Gomes A A, Tavares J & Azevedo M H. Sleep and Academic Performance in Undergraduates: A Multi-measure, Multipredictor Approach. Chronobiology International: The Journal of Biological and Medical Rhythm Research. 2011; 28: 9. DOI: 10.3109/07420528.2011.606518.

**Keywords**

Mental health, sleep, depression, college students

#### O22 Psychopathological symptoms and medication use in higher education

##### Ana P. Amaral, Marina Lourenço, Mª Clara Rocha, Rui Cruz

###### Escola Superior de Tecnologia da Saúde de Coimbra, São Martinho do Bispo, 3046-854 Coimbra, Portugal

####### **Correspondence:** Ana P. Amaral (apamaral.22@gmail.com) – Escola Superior de Tecnologia da Saúde de Coimbra, São Martinho do Bispo, 3046-854 Coimbra, Portugal

**Background**

Research on mental health in higher education reported a high prevalence of psychological distress among students [1]. The college years represent a developmentally challenging transition to adulthood, and untreated mental illness may have significant implications for academic success, productivity, substance use, and social relationships [2]. Objectives: I) To study the prevalence of psychopathological symptoms and medication use in college students and II) To analyse the relationship between these two variables.

Methods

Seven hundred seventy-six (776) Portuguese students (64.3 % females) filled in the Portuguese version of the Brief Symptom Inventory (BSI) [3, 4] and a self-response questionnaire about medication use.

**Results**

20.1 % of the student’s present psychopathology, more prevalent in females in several dimensions (Somatization, Obsessive-Compulsive, Interpersonal Sensitivity, Depression, Anxiety, and Paranoid ideation), being the Obsessive-Compulsive dimension the most prevalent. 50.1 % of the students had consumed medication in the last four weeks: 14.7 % Non-steroidal anti-inflammatory, 14.0 % Analgesics and antipyretics, 5.3 % Antihistamines and 1.7 % Psychotropic drug. The prevalence was higher in females (75.4 %). Comparing students with and without psychopathological symptoms we found significant differences in relation to use of medication (p < 0.05). Students with psychopathological symptoms use more Analgesics and antipyretics and Psychotropic drugs (p < 0.05).

**Conclusions**

Results indicate a relevant prevalence of psychopathology, higher in females and a high prevalence of medication use. Students with psychopathological symptoms use more medication. These results highlight the importance of developing mental health intervention programs.

**References**

1. Christensson A, Runeson B, Dickman, PW, Vaez M. Change in depressive symptoms over higher education and professional establishment - a longitudinal investigation in a national cohort of Swedish nursing students. BMC Public Health, 2010; 10:343–353.

2. Hunt J, Eisenberg D. Mental Health Problems and Help-Seeking Behavior among College Students. Journal of Adolescent Health, 2010; 46(1): 3-10.

3. Derogatis LR. BSI: Brief Symptom Inventory. Minneapolis: National Computers Systems; 1982.

4. Canavarro MC. Inventário de Sintomas Psicopatológicos—BSI. Testes e Provas psicológicas em Portugal. Simões MR, Gonçalves M, Almeida L. Eds., Braga: Sistemas Humanos e Organizacionais. 1999(II), 95-109.

**Keywords**

Mental health, Medication use, Students

#### O23 Sexually transmitted diseases in higher education institutions

##### Sandra Antunes, Verónica Mendonça, Isabel Andrade, Nádia Osório, Ana Valado, Armando Caseiro, António Gabriel, Anabela C. Martins, Fernando Mendes

###### Escola Superior de Tecnologia da Saúde de Coimbra, São Martinho do Bispo, 3046-854 Coimbra, Portugal

####### **Correspondence:** Fernando Mendes (fjmendes@estescoimbra.pt) – Escola Superior de Tecnologia da Saúde de Coimbra, São Martinho do Bispo, 3046-854 Coimbra, Portugal

**Background**

There is evidence from the literature that an individual with adequate health literacy (HL) has the ability to be responsible for its own health as well as one's family and community health. However, actions such as unprotected sexual intercourse (SI) and multiple partners during adolescence are important risk factors to transmission of sexually transmitted diseases (STD). Objective. To study the relationship between HL, STD and risks behaviour in second and third years of higher education studies, comparing students of health sciences (HSc) vs. non-health sciences (NHSc).

**Methods**

A questionnaire was used to evaluate the HL level and sexual risk behaviours, and a blood sample was collected for ELISA screening of HIV, HCV and Herpes simplex virus type 2 (HSV 2), and Treponema Pallidum (VDRL test).

**Results**

Higher levels of HL were found in HSc students (p < 0.001), and also in those with higher level of confidence in condom use (p < 0.05). In average, NHSc students had a higher number of sexual partners (p = 0.001). The practice of anal intercourse is associated with younger age at first SI, as well as a higher number of sexual partners. STD prevalence (2.5 %), in particular in the case of HSV 2, was higher in those who consume more alcohol to relax before a sexual encounter.

**Conclusions**

Students of NHSc show a lower level of HL, higher number of sexual partners and higher level of practice of anal intercourse when compared with HSc students.

**Keywords**

Health literacy, higher education students, HIV, HCV, HSV2, syphilis, risk behaviours

#### O24 Alcohol consumption and suicide ideation in higher education students

##### Lídia Cabral, Manuela Ferreira, Amadeu Gonçalves

###### Escola Superior de Saúde, Instituto Politécnico de Viseu, 3504-510 Viseu, Portugal

####### **Correspondence:** Lídia Cabral (lcabral@essv.ipv.pt) – Escola Superior de Saúde, Instituto Politécnico de Viseu, 3504-510 Viseu, Portugal

**Background**

Alcohol abuse appears associated with numerous risk behaviours among students, including suicide attempts. One in each four students who attempted suicide admitted to having consumed alcohol or drugs before the act (WHO, 2013) [1]. Objective: To identify the influence of alcohol consumption in suicidal ideation of higher education students.

**Methods**

We resorted to a model of quantitative, cross-sectional, analytical, descriptive and correlation research. Two hundred and sixty (260) students of our institution (Health School of Viseu) participated in the study. The evaluation protocol includes a socio-demographic questionnaire, the Involvement Scale for Adolescents with Alcohol Filstead & Mayer (1979) adapted by Fonte & Alves (1999) [2] and the Suicidal Ideation Questionnaire-QIS-Reynolds (1988) adapted to the Portuguese population by Ferreira e Castela (1999) [3].

**Results**

We found that 69.9 % of students started drinking after the age of 15, and curiosity (61.3 %) was the main given reason. Of the 87.2 % habitual drinkers, only 3.3 % reported drinking problems. It should be noted that 3.1 % of the students admitted having suicidal thoughts. In our study there were no statistically significant associations between alcohol consumption and suicidal ideation (p > 0.005).

**Conclusions**

The results point to the importance of continuing to investigate the association between alcohol consumption and suicide ideation. The early onset of alcohol consumption associated with easy access to such drinks and suicidal ideation are variables to consider in future researches and in actions intending to promote healthier lifestyles among higher education students.

**References**

1. WHO Suicide Prevention (SUPRE). WHO: 2013.

2. Fonte A, Alves A. Uso da Escala de Envolvimento com o Álcool para Adolescentes (AAIS). Avaliação das Características Psicométricas. Alcoologia, Revista da Sociedade Portuguesa de Alcoologia. 1999: VII (4).

3. Ferreira J, Castela M. Questionário de Ideação Suicida (Q.I.S.). In M. R. Simões, M.M. Gonçalves & L. S. Almeida (Eds.). Testes e Provas Psicológicas em Portugal. Braga: APPORT/SHO; 1999; 2: 123-130.

**Keywords**

Drinking, suicidal ideation, students

#### O25 Quality of life in university students

##### Tatiana D. Luz, Leonardo Luz, Raul Martins

###### ^1^Universidade Federal de Alagoas, Maceió, Alagoas, 57072-900, Brasil; ^2^Faculdade de Ciências do Desporto e Educação Física, Universidade de Coimbra, 3040-248 Coimbra, Portugal

####### **Correspondence:** Tatiana D. Luz (tati-ddluz@hotmail.com) – Universidade Federal de Alagoas, Maceió - Alagoas, 57072-900, Brasil

**Background**

The transition from high school to university usually represents changes in the lifestyle of the students. However, changes in the quality of life (QoL) are not well known in this population. Objective: The aim of this study is to analyse the QoL of university students by gender and school year.

**Methods**

Participants are 198 undergraduate students (135 males and 63 females) at *Faculdade de Ciências do Desporto e Educação Física* of the University of Coimbra (FCDEF-UC). Demographic variables and physical activity were collected using a self-reported questionnaire. QoL was assessed using the Medical Outcomes Study 36-Item Short-Form Health Survey - SF-36v2. MANOVA one-way, and MANCOVA, controlling for gender, were used to compare variables of interest (p < 0.05).

**Results**

Males had higher QoL than females, specifically in the domains of vitality (M = 61 ± 17, F = 53 ± 18; p =0.003), social functioning (M = 83 ± 18, F = 77 ± 21; p = 0.039), role-emotional (M = 81 ± 21, F = 74 ± 23; p = 0.053), mental health (M = 73 ± 17; F = 66 ± 19; p = 0.005), mental component score (M = 74 ± 15, F = 67 ± 16; p = 0.005), and overall SF-36 (M = 78 ± 11, F = 73 ± 13; p = 0.006). Students in the third school year had higher role-physical (p = 0.045) and bodily pain (p = 0.034) than in the first school year, controlling for gender.

**Conclusions**

Male students had better QoL than women. Students in the third school year had higher values in the domains of role-physical and bodily pain. Further research in this area with university students is recommended.

**Keywords**

Quality of life, SF-36, university students

#### O26 Male and female adolescent antisocial behaviour: characterizing vulnerabilities in a Portuguese sample

##### Alice Morgado, Maria L. Vale-Dias

###### Faculty of Psychology and Education Sciences, University of Coimbra, Coimbra, 3000-115 Coimbra, Portugal

####### **Correspondence:** Alice Morgado (alicemmorgado@gmail.com) – Faculty of Psychology and Education Sciences, University of Coimbra, Coimbra, 3000-115 Coimbra, Portugal

The adolescent antisocial phenomenon is an important matter for our society due to the increase in frequency and severity of deviant conducts during a developmental stage, when individuals face multiple changes.

We present a study on antisocial manifestations and their relation with gender, age, socioeconomic status, personality, social skills, self-concept, and family environment in a sample of 489 adolescents, that filled self-report measures to assess behaviour, personality, self-concept, family environment and social skills.

Results show important gender differences that may explain why boys have higher antisocial tendencies. While, in boys, psychoticism and family environment contribute to determine which individuals are more likely to have higher antisocial scores; female antisocial tendency appears to be defined by individual dispositions and social skills. Significant correlations between antisocial behaviour, age, personality, social skills, self-concept and family environment in boys and girls reveal the importance of individual dispositions and of early prevention efforts focused on the individual and family.

Conclusions focus on the importance of dimensions for the prevention of adolescent antisocial behaviour and reveal important gender differences and trends on the evolution of antisocial behaviour through adolescence. We demonstrate the importance of differences between boys and girls in adolescent antisocial behaviour and of the necessity to address this issue taking into account gender specificities and vulnerabilities.

**Keywords**

Antisocial behaviour, adolescence, gender differences

#### O27 Risk factors for mental health in higher education students of health sciences

##### Rui Porta-Nova (rnova@esscvp.eu)

###### Escola Superior da Saúde da Cruz Vermelha Portuguesa, Lisboa, 1300-125, Portugal

**Background**

The mental health of students attending higher education in the area of Health Sciences may be conditioned by factors which may affect them as a person, their relationships and their academic performance, compromising their adaptation. Objectives: To identify the main risk factors for mental health, associated with the academic life experiences of students of Health Sciences and to contribute to the implementation of prevention strategies of mental health problems in these students.

**Methods**

The research dealt with a sample of 620 people, medical students, nursing students and applied health students, namely: physiotherapy, cardiopulmonary technology and radiography, whose average age was 20.3 and ranged between 18 and 25 years old, 81 % being female. The instruments used were a Demographic Questionnaire; Academic Life Experiences Questionnaire [1,2] and Mental Health Inventory [3].

**Results**

The results showed that attending the 1st year in the course of physiotherapy, belonging to the female gender, being displaced, having a lower career expectation, not being autonomous, showing a negative perception of their cognitive skills, lower self-confidence, decreased of psychological and/or physical well-being, academic difficulties resulting from deficient knowledge bases and high levels of anxiety in situations of assessment, are associated with poorer mental health.

**Conclusions**

This calls for special attention from the different structures of Higher Education institutions, namely, governing bodies, teachers and academic services, to reduce the negative impact of these risk factors and implement strategies to prevent the incidence of mental health problems in this population.

**References**

1. Almeida LS, Ferreira JA. Questionário de vivências académicas. Braga: Universidade do Minho, Centro de Estudos em Educação e Psicologia; 1997.

2. Almeida LS. Ferreira JA. Adaptação e rendimento académico no ensino superior: fundamentação e validação de uma escala de avaliação de vivências académicas. Psicologia: Teoria, Investigação e Prática. 1999; 1:157-170.

3. Veit C, Ware J. The structure of psychological distress and well-being in general populations. Journal of Consulting and Clinical Psychology. 1983; 51:730-742.

**Keywords**

Mental Health, risk factors, higher education, health sciences students

#### O28 International classification of functioning disability and health as reflexive reasoning in primary attention in health

##### Tânia C. Fleig, Éboni M. Reuter, Miriam B. Froemming, Sabrina L. Guerreiro, Lisiane L. Carvalho

###### Universidade de Santa Cruz do Sul, Santa Cruz do Sul – Rio Grande do Sul, 96815-900, Brasil

####### **Correspondence:** Tânia C. Fleig (tcmfleig@gmail.com) – Universidade de Santa Cruz do Sul, Santa Cruz do Sul, Rio Grande do Sul, 96815-900, Brasil

**Background**

In Primary Health Care (PHC) the impact of health problems on the individual’s health, interaction with the environment and on the quality of life guided by the International Classification of Functioning Disability and Health (ICF), allows an approach to health indicators and attention to the components of functionality, environmental and personal contexts. Objective: To describe the functionality profile of people registered in Family Health Strategies (FHSs).

**Methods**

A transversal study. Ninety-two people were attended in the physiotherapy apprenticeship programme of UNISC, in PHC, starting from the physiotherapeutic diagnosis guided by ICF, at four FHSs in Santa Cruz do Sul (Rio Grande do Sul, Brazil), in 2015. Data presented in relative frequency.

**Results**

The prevalent alterations were sensorial functions and pain (84 %), pain (75 %), cardiovascular, haematological, immunological and respiratory alterations (56 %), neuromusculoskeletal problems and those related to movement (85 %), mobility of joints (73 %) and muscular strength (50 %), structures related to movement (94 %) and lower extremities (56 %). The limitations of activities and restriction in participation was in mobility (66 %). The environmental factors of larger occurrence were products and technology (56 %), support and relationships (91 %), immediate family being a facilitator and barrier (67 % and 14 %), and health professional facilitators (82 %). Services, systems and policies (62 %) are added, especially services, systems and policies of health (52 % facilitator; 4 % barrier).

**Conclusions**

The ICF is considered in the composition of health indicators sustaining, expanding and guiding the actions of health, enlarging access and people's accessibility to provide comprehensive attention and to care.

**Keywords**

International Classification of Functionality, Primary Health Care, health indicators

#### O29 Risk factors and cardiovascular disease in Portalegre

##### Daniel Guedelha, P. Coelho, A. Pereira

###### Escola Superior de Saúde Dr. Lopes Dias, Instituto Politécnico de Castelo Branco, 6000-767 Castelo Branco, Portugal

####### **Correspondence:** Daniel Guedelha (daniel.jsguedelha@gmail.com) – Escola Superior de Saúde Dr. Lopes Dias, Instituto Politécnico de Castelo Branco, 6000-767 Castelo Branco, Portugal

**Background**

Cardiovascular disease is the main cause of disability and premature death worldwide, being associated to these clinical conditions various cardiovascular risk factors leading to a higher prevalence of cardiovascular disease manifested in different ways. Among the various cardiovascular risk factors, it stands out dyslipidaemia, diabetes mellitus, hypertension, smoking and family history of atherosclerotic disease. Objective: The aim is to estimate the prevalence of cardiovascular risk factors and cardiovascular disease, such as acute myocardial infarction and stroke in Portalegre city's population.

**Methods**

The present study is of the analytical, observational and cross-sectional type in a random sample, consisting of 1,000 subjects and collected between July 2014 and January 2015. Data were collected by a questionnaire applied to the population under study.

**Results**

In 1,000 subjects there was a mean age of 52.5 ± 19.1 years and 54.7 % of subjects belonged to the female gender. The overall prevalence of hypertension was 35.0 % followed by hypercholesterolemia with 32.3 %, smoking habits with 14.0 % and 15.8 % of ex-smokers, diabetes present in 12.0 % of respondents and hypertriglyceridemia, that was the less present in the population with 7.2 %. It was also analysed cardiovascular pathology, being obtained a prevalence of acute myocardial infarction of 4.2 % and 2.7 % for stroke.

**Conclusions**

The prevalence of risk factors proved to be high in the population of the city of Portalegre, as well as the occurrence of the cardiovascular events studied.

**Keywords**

Cardiovascular risk factors, Acute Myocardial Infarction, stroke, prevalence, Portalegre

#### O30 Health status of the elderly population living in Portalegre historic city centre: A longitudinal study

##### António Calha, Raul Cordeiro

###### Instituto Politécnico de Portalegre, 7301-901 Portalegre, Portugal

####### **Correspondence:** António Calha (antoniocalha@hotmail.com) – Instituto Politécnico de Portalegre, 7301-901 Portalegre, Portugal

Demographical changes and the increasing proportion of older people will generate new health needs in the coming years as well as huge challenges to health and social systems. The growth in the number of older people living alone in urban areas is particularly problematic because of the potential health risk that may be associated with social isolation and loneliness.

In this paper we present the results of a longitudinal descriptive and correlational study of the elderly population living in Portalegre historic city centre. The purpose of the research is to monitor trends between 2013 and 2015, regarding health status (self-assessment of health status; self-assessment of the existence of changes in health status and autonomy in performing everyday activities), feelings of loneliness and symptoms of depression. The first evaluation took place in January 2013 comprising a sample of 123 elders. The second evaluation took place in October 2015 comprising 44 elderly people (79 cases had been lost between the 1st and the 2nd evaluations).

Results showed a deterioration of health status (although differences were not statistically significant), changing the self-perceived unhealthy elderly from 43.5 % to 58.2 % (p = 0.227 by McNemar's test)- The same tendency was found in the percentage of elderly needing help in performing everyday activities, changing from 5 % in 2013 to 14 % in 2015 (p = 0.289 by McNemar's test).

Concerning feelings of loneliness and symptoms of depression a weakening was also found of elderly people’s status with potential consequences on social and personal wellbeing.

**Keywords**

Aging, health status, loneliness, depression symptoms

#### O31 Student’s sleep in higher education: sleep quality among students of the IPB

##### Ana Gonçalves, Ana Certo, Ana Galvão, Mª Augusta Mata

###### Instituto Politécnico de Bragança, Bragança, 5300-121, Portugal

####### **Correspondence:** Ana Gonçalves (velosogoncalves@gmail.com) – Instituto Politécnico de Bragança, Bragança, 5300-121, Portugal

**Background**

Sleep represents a basic human need, embodying several crucial functions in the young adult phase. Objective: To evaluate the sleep quality of higher education students.

**Methods**

A descriptive-transversal study with a quantitative approach. Non-probabilistic convenience sample of 358 students from Instituto Politécnico de Bragança (IPB). Data collection tools used: Socio-demographic record and the Pittsburgh Sleep Quality Index (PSQI).

**Results**

The majority of the sample (54 %) presents bad sleep quality and the remaining (46 %) good quality. Concerning gender, we can highlight that the majority of males presents good sleep quality and the majority of the females bad quality. Concerning sleep quality, the results match those of Pereira (2013) [1], who obtained a global PSQI score of 7.74, with 76 % of the students considered to have bad sleep quality and 24 % good quality. Data from national and international literature state that university or higher education students present bad sleep quality. Concerning the difference of sleep quality among genders, the results corroborate those of Coelho (2014), [2] in which females were more affected by bad sleep quality.

**Conclusions**

The majority of higher education students consider that they have bad sleep quality and gender influences quality. Given the aforementioned, it becomes imperative to perform interventions that promote sleep quality in this group.

**References**

1. Pereira, A. Hábitos de Sono em Estudantes Universitários [Dissertação]. Porto: Universidade Fernando Pessoa; 2013.

2. Coelho, A. Avaliação da Qualidade do Sono em Estudantes Universitários e a sua relação com Disfunções Temporomandibulares Musculares [Dissertação] Porto: Universidade Fernando Pessoa; 2014.

**Keywords**

Sleep, students, higher education, life quality

#### O32 Trend in mortality from cervical cancer in the metropolitan area of Florianópolis, state of Santa Catarina, Brazil, 2000 to 2013

##### Aline Welter^1^, Elayne Pereira^1^, Sandra Ribeiro^2^, Marcia Kretzer^1^

###### ^1^Universidade do Sul de Santa Catarina, Palhoça, Santa Catarina, 88137-270 Brasil; ^2^Secretaria Municipal de Saúde de Palhoça, Palhoça, Santa Catarina, 88132-149, Brasil

####### **Correspondence:** Aline Welter (ninewelter@gmail.com) – Universidade do Sul de Santa Catarina, Palhoça, Santa Catarina, 88137-270 Brasil

**Background**

Cervical cancer is a public health problem in Brazil and worldwide, due to the high mortality rates observed mainly in less developed regions. Being the most preventable cancer, is of great importance its early diagnosis and treatment. Objective: To analyse time trends in mortality from cervical cancer 2000 to 2013 in the metropolitan area of Florianópolis, state of Santa Catarina - Brazil, by age-specific groups and its regions.

**Methods**

An ecological study of time series was conducted using data from the National Information System on Mortality. Data on deaths from 2000 to 2013. Crude and specific mortality rates were calculated according to age group and region. The existence of correlations was analysed by the Spearman's correlation coefficient t.

**Results**

There was little variability in mortality trends from Cervical Cancer in the metropolitan region, ranging between 3.43 and 2.24/100 thousand, with a declining trend from 40 to 49 years (r = -0.547; p < 0.05). Analysing the cities, in Florianópolis there was a declining trend from 60 to 69 years (r = -0.612; p < 0.05) and from 70 to 79 years (r = -0.701; p < 0.01), while in the São José city there was an increasing trend in the age group from 60 to 69 years (r = 0.539; p < 0.05).

**Conclusions**

The mortality trend of Cervical Cancer in the metropolitan area of Florianópolis is stationary, with a decrease from 40 to 49 years. In the city of Florianopolis there was a decreasing trend from 60 to 79 years, and in the São José city there an increase from 60 to 69 years.

**Keywords**

Cervical cancer, uterine cervical neoplasms, mortality trends

#### O33 Adherence to treatment in the elderly in an urban environment in Spain

##### Juan-Fernando Jiménez-Díaz^1^, Carla Jiménez-Rodríguez^1^, Francisco-José Hernández-Martínez^2^, Bienvenida-Del-Carmen Rodríguez-De-Vera^1^, Alexandre Marques-Rodrigues^3^

###### ^1^Universidad de Las Palmas de Gran Canaria, 35001 Las Palmas de Gran Canaria, Las Palmas, España; ^2^Cabildo de Lanzarote, 35500. Arrecife, Lanzarote, Las Palmas, España; ^3^Universidade de Aveiro, 3810-193 Aveiro, Portugal

####### **Correspondence:** Juan-Fernando Jiménez-Díaz (juanfernando.jimenez@ulpgc.es) – Universidad de Las Palmas de Gran Canaria, 35001 Las Palmas de Gran Canaria, Las Palmas, España

**Background**

Lack of adherence to treatment is a major public health problem. Over 50 % of the Spanish population fails to comply with prescribed treatments. The WHO defines adherence as "the degree to which a person's behaviour corresponds with agreed recommendations from a health care provider." Therefore, in addition to the clinical impact also it has socio-economic implications. Objective: To determine the level of adherence to treatment in a sample of over 65 with chronic polypharmacy and diseases in an urban environment.

**Methods**

A cross-sectional descriptive study with a simple random sampling from a Community Pharmacy Service in an urban environment by applying the Morisky-Green Test.

**Results**

36.8 % of patients do not show good adherence in taking medication. However, the overall adherence to treatment increases with age, especially among those over 71, with no statistical significance between genders and, similarly, better adherence correlates with less education of individuals, but not with their socioeconomic status.

**Conclusions**

The positive predictive value of the Morisky-Green Test can be a good alternative to identifying groups at risk of non-adherence in primary care, especially when our findings show that the older and less educated patients achieved better therapeutic adherence, which is conditioned by a greater dependence on health professionals in determining the criteria.

**Keywords**

Adherence, Therapy, Elderly

#### O34 Beira Baixa Blood Pressure Study (Study PABB)

##### Patrícia Coelho, Tiago Bernardes, Alexandre Pereira

###### Escola Superior de Saúde Dr. Lopes Dias, Castelo Branco, 6000-767, Portugal

####### **Correspondence:** Patrícia Coelho (patriciacoelho@ipcb.pt) – Escola Superior de Saúde Dr. Lopes Dias, Castelo Branco, 6000-767, Portugal

**Background**

Arterial Hypertension (AHT) is a multifactorial systemic disease characterized by high and sustained levels of blood pressure (BP). In Portugal a prevalence of 42 % is estimated among the adult population and it is known to be closely related to cardiovascular disease. Objective: To characterize the population of Castelo Branco district regarding the prevalence, knowledge and AHT control rates, as well as to determine cardiovascular risks (CVR).

**Methods**

Cross-sectional study performed in the district of Castelo Branco between 2010-2014 through survey application, BP measurements via the auscultatory method and anthropometric data information. The sample consisted of 11,139 individuals, 55.6 % female, aged between 18 and 101 years, with a mean of 58.17 ± 17.95 years. Data were analysed with SPSS® 20.0.

**Results**

The prevalence of AHT was 53.2 % (53.8 % in males and 52 % in females). 6.8 % of the subjects had prehypertension; 24.1 % at AHT grade 1; 8.1 % AHT grade 2 and 2.1 % AHT grade 3. Of the 4,199 individuals under antihypertensive therapy, 49.8 % had BP values above normal. Regarding the CVR 51.1 % of subjects had no significant CVR; 10.9 % had low risk; 19.0 % moderate risk and 18.9 % high or very high risk according to the international guidelines.

**Conclusions**

There was prevalence above the national average as well as a high rate of medicated AHT with high BP values. It’s a population with high CVR, fact that should be taken into account in drawing up preventive strategies for the control of cardiovascular risk factors.

**Keywords**

Prevalence, arterial hypertension, risk factors, cardiovascular risk

#### O35 Trends in cervical cancer mortality statistics in Santa Catarina State, Brazil, by age group and macro-region, from 2000 to 2013

##### Patrícia Sousa, João G. Filho, Nazare Nazario, Marcia Kretzer

###### Universidade do Sul de Santa Catarina, Palhoça, Santa Catarina, 88137-270 Brasil

####### **Correspondence:** Patrícia Sousa (patricia.pssantos@hotmail.com) – Universidade do Sul de Santa Catarina, Palhoça, Santa Catarina, 88137-270 Brasil

**Background**

Cervical cancer is a public health issue in Brazil and worldwide, due to the high mortality rates observed mainly in less developed regions. Being the most preventable cancer, its early diagnosis and treatment is of great importance. Objective: To analyse the trends in cervical cancer mortality statistics over time in Santa Catarina State, Brazil.

**Methods**

An ecological time series study was conducted using data from the Brazilian National Information System on Mortality. Trends in the data on deaths from 2000 to 2013 were identified using linear regression. Crude and specific mortality rates were calculated according to age group and macro-region.

**Results**

There was little variability in the cervical cancer mortality rates in this state, ranging from 1.80 to 2.66/100,000, and this variation showed no statistical significance. There was an increasing trend for the age group 0-29 years, with a mean annual increase of 0.01 % (p = 0.02), and a decreasing for the 40-49 years (0.16 %; p = 0.004). On analysing the health administration macro-regions, the only significant increasing trend was observed for the Vale do Itajaí (0.04 %; p = 0.02).

**Conclusions**

For the period of 2000 to 2013 the overall cervical cancer mortality in Santa Catarina State is trend stationary, but the mortality is increasing for the age range of 0 to 29 years and decreasing for the age range of 40 to 49 years. The only health administration macro-region of Santa Catarina State which has registered a significant increasing trend in cervical cancer mortality is the Itajaí Valley.

**Keywords**

Uterine cervical neoplasms, cervical cancer, mortality trends

#### O36 Sleep problems among Portuguese adolescents: a public health issue

##### Odete Amaral^1^, António Garrido^2^, Nélio Veiga^3^, Carla Nunes^4^, Ana R. Pedro^4^, Carlos Pereira^1^

###### ^1^Centro de estudos em educação, tecnologias e saúde, Instituto Politécnico de Viseu, 3504 - 510 Viseu, Portugal; ^2^Casa de Saúde de São Mateus, Viseu, 3500-106, Portugal; ^3^Universidade Católica Portuguesa, Viseu, 3504-505, Portugal; ^4^Escola Nacional de Saúde Pública, Lisboa, 1600-560, Portugal

####### **Correspondence:** Odete Amaral (mopamaral@gmail.com) – Centro de estudos em educação, tecnologias e saúde, Instituto Politécnico de Viseu, 3504 - 510 Viseu, Portugal

**Background**

Recent studies have shown that sleeping problems, particularly during adolescence, are an important public health problem. During adolescence many biological, psychological and social factors interact, resulting in shortening of sleep duration, excessive sleepiness, insomnia and delayed sleep phase syndrome. The aim of this study was to determine the prevalence of sleep problems in adolescents.

**Methods**

In a cross-sectional approach we assessed 7,354 students (3944 females), aged 11 to 20 years from twenty-six schools of the district of Viseu, Portugal. Data was collected using a self-administered questionnaire answered by the students in the classroom. Insomnia was defined based on the Diagnostic and Statistical Manual of Mental Disorders IV criteria. Daytime sleepiness was assessed by the Epworth Sleepiness Scale and we considered as "insufficient" less than 8 hours of night sleep.

**Results**

The prevalence of “insomnia” was 8.3 % and of “insomnia symptoms” 21.4 %. The prevalence of “difficulty initiating sleep”, “difficulty maintaining sleep”, “early morning awakening” and “non-restorative sleep” was 8.9 %; 8.2 %; 6.1 % and 5.6 %, respectively. The prevalence of insomnia and symptoms of insomnia were higher among the female gender (p < 0.001). Each symptom was more prevalent among the female gender (p < 0.001). The prevalence of daytime sleepiness was 33.1 % and of insufficient sleep 29.0 %. Both were higher among the female gender (p < 0.001). Only 6.4 % of the adolescents reported to lie down every night at the same hour.

**Conclusions**

Sleep problems in Portuguese adolescents are common. The results of this study suggest the need for comprehensive programs to prevent sleep problems in Portuguese adolescents.

**Keywords**

Sleep problems, Insomnia, Daytime sleepiness, Insufficient sleep, Adolescents

#### O37 Association between body fat and health-related quality of life in patients with type 2 diabetes

##### António Almeia^1^, Helder M. Fernandes^1^, Carlos Vasconcelos^1,2^, Nelson Sousa^1^, Victor M. Reis^1^, M. João Monteiro^1^, Romeu Mendes^1,3^

###### ^1^Research Centre Sports Sciences, Health Sciences and Human Development, University of Trás-os-Montes e Alto Douro, Vila Real, 5001-801, Portugal; ^2^Polytechnic Institute of Viseu, Viseu, 3504-510, Portugal; ^3^Unidade de Saúde Pública do ACES Douro I—Marão e Douro Norte, Administração Regional de Saúde do Norte, IP, 5000-524 Vila Real, Portugal

####### **Correspondence:** Romeu Mendes (romeuduartemendes@gmail.com) – Research Centre Sports Sciences, Health Sciences and Human Development, University of Trás-os-Montes e Alto Douro, Vila Real, 5001-801, Portugal

**Background**

There is increasing evidence that obesity is associated with a decline in health-related quality of life (HRQOL). Objective: This cross-sectional study aimed to analyse the relation between body fat and HRQOL in patients with type 2 diabetes.

**Methods**

The SF-36v2 questionnaire was administered to 95 individuals with type 2 diabetes (47 women and 48 men; 66.23 ± 6.34 years old; 10.55 ± 7.55 years of diabetes; 37.47 ± 8.19 % of body fat) candidates to *Diabetes em Movimento®*, a community-based lifestyle intervention program developed in Vila Real, Portugal (NCT02631902). Body fat was assessed by bioelectrical impedance analysis technique (Tanita, BC-418 MA). Pearson’s correlation coefficients were used to evaluate the associations between each SF-36v2 scale (physical functioning, PF; role physical, RP; bodily pain, BP; general health, GH; vitality, VT; social functioning, SF; role emotional, RE; mental health, MH) and summary scales (physical component score, PCS; mental component score, MCS), and patient’s body fat.

**Results**

Negative and significant correlations were observed between body fat and PF (r = -0.331; p = 0.001), BP (r = -0.324; p = 0.001), VT (r = -0.336; p = 0.001), SF (r = -0.231; p = 0.025), RE (r = -0.280; p = 0.006), MH (r = -0.310; p = 0.002), PCS (r = -0.234; p = 0.023), and MCS (r = -0.230; p = 0.026), respectively.

**Conclusions**

High levels of body fat are associated with impaired HRQL in patients with type 2 diabetes, possibly compromising the individuals' ability to perform daily activities.

**Keywords**

Health-related quality of Life, obesity, type 2 diabetes

#### O38 Therapy adherence and polypharmacy in non-institutionalized elderly from Amares county, Portugal

##### Isabel C. Pinto^1^, Tânia Pires^2^, João Gama^3^

###### ^1^Núcleo de Investigação e Intervenção no Idoso, Departamento de Tecnologias de Diagnóstico e Terapêutica, Escola Superior de Saúde, Instituto Politécnico de Bragança, Bragança, 5300-253, Portugal; ^2^Centro de Investigação de Montanha, Escola Superior Agrária, Instituto Politécnico de Bragança, 5300-253 Bragança, Portugal; ^3^Escola Superior de Saúde, Instituto Politécnico de Bragança, 5300-253 Bragança, Portugal

####### **Correspondence:** Isabel C. Pinto (isabel.pinto@ipb.pt) – Núcleo de Investigação e Intervenção no Idoso, Departamento de Tecnologias de Diagnóstico e Terapêutica, Escola Superior de Saúde, Instituto Politécnico de Bragança, Bragança, 5300-253, Portugal

**Background**

Polypharmacy is frequent in elderly, and therapy adherence is a crucial component to achieve the effectiveness of treatment, although the complex therapies in elderly can lead to therapy non-adherence, increasing costs and several health risks. Objective: To estimate the prevalence of therapy adherence, polypharmacy and associated factors in elderly.

**Methods**

This cross-sectional study was based on a questionnaire, with the MAT scale (Measure of Adherence to Therapy) validated for the Portuguese population [1], applied to 159 elderly (≥65 years) living in their home in Amares county, in the north of Portugal. To assess therapy adherence, those whose average adherence levels were ≥ 5 were called “adherent”, and considered as polymedicated seniors taking ≥ 5 drugs. It was used descriptive statistics, correlations were accessed using the qui-square test and adjusted residuals (AdR) for variables categories, with a significance level of 5 %.

**Results**

The sample consisted mainly of females (54.7 % vs. 45.3 %), aged between 65 and 96 years old (mean 74.6), while 50.3 % was between 65-74 years old. The participants show good therapy adherence (69.8 %), being highly polymedicated (58.5 %) with an average of 5.5 different drugs administered per day. Non-adherence was associated with having mental disorders (p = 0.002) and respiratory system diseases (AdR = 2.0), and seems to be related with being polymedicated (AdR = 1.7) and bad health perception (AdR = 1.3). Having hypertension, cholesterol, depression, mental disorders (p = 0.001), pain (p = 0.003) and diabetes (p = 0.014) were also related to polypharmacy.

**Conclusions**

This study shows a considerable prevalence of therapy adherence and polypharmacy, being several factors associated with these phenomena.

**References**

1. Delgado A, Lima M. Contributo para validação concorrente de uma medida de adesão aos tratamentos. Psicologia, Saúde & Doenças. 2001; 2(2): 81-100.

**Keywords**

Elderly, non-institutionalized elderly, polypharmacy, therapy adherence, therapy non-adherence

#### O39 Prevalence of surgical site infection in adults at a hospital unit in the North of Portugal

##### Vera Preto^1^, Norberto Silva^2^, Carlos Magalhães^3^, Matilde Martins^4^

###### ^1^Programa de Prevenção e Controlo de Infeções e de Resistência aos Antimicrobianos, Unidade Local de Saúde do Nordeste, Bragança, 5301-852, Portugal; ^2^Departamento de Urgência e Emergência Unidade, Unidade Local de Saúde do Nordeste, Bragança, 5301-852, Portugal; ^3^Departamento de Enfermagem, Escola Superior de Saúde, Instituto Politécnico de Bragança, 5300-121 Bragança, Portugal; ^4^Centro de Investigação em Desporto, Saúde e Desenvolvimento Humano, Departamento de Enfermagem, Escola Superior de Saúde, Instituto Politécnico de Bragança, 5300-121 Bragança, Portugal

####### **Correspondence:** Vera Preto (vera.preto@sapo.pt) – Programa de Prevenção e Controlo de Infeções e de Resistência aos Antimicrobianos, Unidade Local de Saúde do Nordeste, Bragança, 5301-852, Portugal

**Background**

Infection of the surgical site is one of the most frequent infections associated with health care. Objective: To identify the prevalence of surgical site infection in adults at a hospital in the North of Portugal.

**Methods**

A prospective study carried out at a hospital in the north of Portugal in 2015. Inclusion criteria: having been hospitalized in surgical and obstetrics services in the past 24 hours, aged 16 years or more, undergoing colon surgery, gallbladder and caesarean section. A sample of 579 participants was obtained. The characterization of the patient and the surgery was performed using an inquiry application in the first 24 hours after surgery, and the registration of the infection at the time of occurrence within 30 days following the procedure.

**Results**

Among the 579 participants 53.4 % were females, with an average age of 57.1 years (17-97 years), 64.1 % underwent prophylactic antibiotic therapy, in 52.7 % the surgery was urgent, 6.6 % underwent surgery laparoscopically, 70.0 % of the surgeries occurred in the surgery department, of which 33.3 % were cholecystectomy. Predominantly the wounds were clean (62.0 %). The average wait for surgery was 7 days, for admission 12 days and average surgical time 59 minutes. There was a 6.0 % prevalence of surgical site infection and Escherichia coli accounted for 47.8 % of them. The average time of onset was 9 days and the organ/space was the most affected site (48.6 %).

**Conclusions**

Prevalence of infection was 6.0 %. It is suggested to carry out further studies that show factors associated with this type of infection.

**Keywords**

Surgical Wound Infection, Escherichia coli, Prevalence

#### O40 Frailty phenotype in old age: implications to intervention

##### Mafalda Duarte^1^, Constança Paúl^2,3^, Ignácio Martín^4^

###### ^1^Health Superior School of Alto Ave, 4830-345 Póvoa de Lanhoso, Portugal; ^2^Unidade de Investigação e Formação sobre Adultos e Idosos, Instituto de Ciências Biomédicas Abel Salazar, Universidade do Porto, 4050-313 Porto, Portugal; ^3^Center for Health Technology and Services Research, Faculdade de Medicina, Universidade do Porto, 4200-450 Porto, Portugal; ^4^Universidade de Aveiro, 3810-193 Aveiro, Portugal

####### **Correspondence:** Mafalda Duarte (mafalda.duarte@isave.pt) – Health Superior School of Alto Ave, 4830-345 Póvoa de Lanhoso, Portugal

**Background**

The Frailty Phenotype is a syndrome composed of five criteria: weight loss, endurance, physical activity, slowness and weakness. An older person is considered to be frail if being impaired in three of these domains. This study aims to identify predictive factors of the frailty condition that may be considered for intervention.

**Methods**

A representative sample, stratified by age group, of elders living in the community (n = 339) was assessed and logistic regression models conducted.

**Results**

Predictive factors were gender (woman) OR 1.7, 95 % CI 1.0 – 2.8), age (more advanced) (OR 2.8, 95 % CI 1.6 - 4.9) and educational level (no schooling) (OR 2.6, 95 % CI 1.1 – 6.0). The bio behavioural variables and the low respiratory flow predicted the frailty condition (OR 3.3, 95 % CI 1.9 – 6.0). Geriatric indicators as falls (OR 3.3, 95 % CI 1.5 - 5.6), changes in sensorial processes (OR 2.1, 95 % CI 1.2 -3. 8; OR 2.1, 95 % CI 1.1 - 4.0 respectively), comorbidity (OR 1.8, 95 % CI 1.0 - 3.2) were also predictors of frailty. Impairment in ADL increases the risk of frailty (OR 2.1, 95 % IC 1.2 -3.5). The presence of depressive symptomatology (OR 4.2, 95 % IC 1.9-9.2) and cognitive deterioration (OR 2.9, 95 % IC 1.6 -5.3) are equally predictive of this condition.

**Conclusions**

These biopsychosocial predictors were all considered in an intervention program.

**Keywords**

Elders, frailty, predictive factors, intervention

#### O41 Portuguese women: sexual symptoms in perimenopause

##### Arminda A. Pinheiro (aanes@ese.uminho.pt)

###### Escola Superior de Enfermagem, Universidade do Minho, Braga, 4710-057 Braga, Portugal

**Background**

Cultural differences in sexual symptoms exist, and should be measured in perimenopause, including the following symptoms: loss of interest in sex, vaginal dryness, satisfaction and pain during intercourse. The measurement of these symptoms provides a comparison between studies.

**Methods**

A cross-sectional study, correlational; with a non-probabilistic convenience sample (n = 600 Portuguese women perimenopause, 45 - 55 years) was performed. Protocol included: Menopause Rating Scale; attitudes and beliefs before menopause (built and validated by us); Social Support Satisfaction Scale, Scale, levels E2; FSH, sociodemographic; lifestyle and projects, perception of subjective well-being and stressful events.

**Results**

Regarding the influence of different factors included in the final model on the probability of a woman having reported uncomfortable sexual symptoms, logistic regression forward showed the conditions of socio-demographic/socio-economic factors: labour condition (bunemployed = 0.817; p = 0.001; OR = 2.264), household formation (balone, with children = - 0.993; p < 0.001; OR = 0.136); the conditions of psychosocial factors: meaning attributed to menopause (bpositive meaning = -1.038; p < 0.001; OR = 0.354), level of self-esteem (bself-esteem = -0.045; p = 0.029; OR = 0.956), satisfaction with social support (b family support = -0.099; p = 0.039; OR = 0.906), attitudes/beliefs facing menopause (bchanges health aging = -0.148, p = 0.023; OR = 0.863);attitudes/beliefs facing menopause (b physical changes = -0.147; p = 0.011; OR = 0.863).The adjusted Logit model (G2(8) = 173.621; p < 0.001; X2wald (8) = 16.847; p = 0.032; R2CS = 0.251; R2N = 0.349; R2MF = 0.228).

**Conclusions**

Midwives can address the attitudes and self-esteem of perimenopausal women to promote sexual health in family planning consultations.

**Keywords**

health sexual, perimenopause, women

#### O42 Predictive ability of the Perinatal Depression Screening and Prevention Tool – preliminary results of the categorical approach

##### Sandra Xavier^1^, Julieta Azevedo^1^, Elisabete Bento^1^, Cristiana Marques^1^, Mariana Marques^1,2^, António Macedo^1,2^, Ana T. Pereira^1^

###### ^1^Faculty of Medicine, University of Coimbra, 3000-354 Coimbra, Portugal; ^2^Centro de responsabilidade integrado, Serviço Psiquiatria, Centro Hospitalar e Universitário de Coimbra, 3000-354 Coimbra, Portugal

####### **Correspondence:** Sandra Xavier (sandraalvesxavier@gmail.com) – Faculty of Medicine, University of Coimbra, 3000-354 Coimbra, Portugal

**Background**

The 2015 update of the depression screening recommendations reflects the importance of screening during and after pregnancy. The perinatal depression screening and early detection should combine the evaluation of depressive symptoms and risk factors. Objective: To analyse the predictive ability of the Perinatal Depression Screening and Prevention Tool (PDSP Tool) assessing both PD symptoms and risk factors identified by our team (lifetime history of depression/LtHD, prenatal insomnia, increased depressive symptoms and negative affect/NA at pregnancy) to identify postpartum major depression.

**Methods**

Nighty-two (92) pregnant women (mean age: 32.64 ± 4.59 years) in their second trimester (21.38 ± 2.41 weeks of gestation) completed the PDSP Tool. At six (6.34 ± 1.66) weeks postpartum they were interviewed with the Diagnostic Interview for Psychological Distress-Postpartum, to determine if they fulfilled the diagnostic criteria for major depression/DSM-5.

**Results**

4.3 % of the women showed major depression. The global correct classification rate of the PDSP Tool was 53.3 %. False-negatives 1.1 %, false-positives 44.6 %, true-negatives 55.2 % and true-positives 3.3 %. Considering the PDSP Tool components, high NA showed the highest predictive ability: 100 % of women with high NA at pregnancy (X^2^ = 11.21, OR = 2.190, p = .005) had major depression in the postpartum.

**Conclusions**

Considering that false-negatives are worse than false-positives, the finding that the PDS Tool identifies more than half of the women who will develop postpartum major depression encourages us to continue to develop efforts to succeed in this very difficult task of identifying pregnant women who will tend to have major depression in the postpartum.

**Keywords**

Perinatal Depression, early screening, assessment

#### O43 Aging and muscle strength in patients with type 2 diabetes: cross sectional analysis

##### José P. Almeida^1^, António Almeida^1,2^, Josiane Alves^1^, Nelson Sousa^1,2^, Francisco Saavedra^1,2^, Romeu Mendes^1,2,3^

###### ^1^University of Trás-os-Montes e Alto Douro, Vila Real, 5001-801, Portugal; ^2^Research Centre Sports Sciences, Health Sciences and Human Development, University of Trás-os-Montes e Alto Douro, Vila Real, 5001-801, Portugal; ^3^Unidade de Saúde Pública, ACES Douro I, Marão e Douro Norte, Administração Regional de Saúde do Norte, 5000-524 Vila Real, Portugal

####### **Correspondence:** José P. Almeida (romeuduartemendes@gmail.com) – University of Trás-os-Montes e Alto Douro, Vila Real, 5001-801 Vila Real, Portugal

**Background**

The aging process is associated with the decline of muscle mass and strength and this loss is increased by diabetes, leading to the development of physical disability in older adults with diabetes. Objective: This study aimed to analyse the association between age and muscle strength levels in middle-age and older patients with type 2 diabetes (T2D).

**Methods**

Ninety-three individuals with T2D (47 men and 46 women; 66.26 ± 6.32 years of age [55 to 80 years]) candidates for Diabetes em Movimento®, a community-based lifestyle intervention programme developed in Vila Real, Portugal (NCT02631902), participated in a cross sectional analysis. Upper limb muscle strength was assessed through performance in the Seated Medicine Ball Throw Test (SMBT) and lower limb through performance in the 30-Second Chair Stand Test (30-SCS). Pearson’s correlation coefficients were used to evaluate the associations between age and muscle strength levels, in both genders.

**Results**

Negative and significant correlations were observed between age and SMBT in men (r = -0.345, p = 0.019) and women (r = -0.314, p = 0.033), and between age and 30-SCS in women (r = -0.409, p = 0.005), but not in men (r = -0.126, p = 0.403).

**Conclusions**

In general, the results showed a relation between aging and loss of muscle strength in both genders in patients with T2D. However, men seem more protected than women in the loss of lower limb muscle strength.

**Keywords**

Muscle strength, aging, Type 2 Diabetes

#### O44 Accessibility of the elderly in the prevention of hypertension in a family health unit

##### Ana S. Maia^1^, Michelle T. Oliveira^1^, Anderson R. Sousa^1^, Paulo P. Ferreira^2^, Luci S. Lopes^1^, Eujcely C. Santiago^3^

###### ^1^Faculdade Nobre, Feira de Santana - Bahia, 44001-008, Brasil; ^2^Secretaria de Saúde do Estado da Bahia, Salvador - Bahia, 41745-900, Brasil; ^3^Hospital Estadual da Criança, Vila Valqueire, Rio de Janeiro, Brasil

####### **Correspondence:** Ana S. Maia (anamargarette@yahoo.com.br) – Faculdade Nobre, Feira de Santana, Bahia, 44001-008, Brasil

In Brazil, there is a steady increase in life expectancy, due to the decline in mortality and fertility rates, thus increasing the number of elderly. With that index of Chronic Diseases has grown, with arterial hypertension as an example. This research aimed to analyse accessibility of the elderly in the prevention of hypertension in a Family Health Unit. This is a descriptive and exploratory study, with qualitative approach, performed at a Family Health Unit in the municipality of Bahia, Brazil. It was conducted with 16 elderly people, aged over 60 years, through a semi-structured interview. The interviews were submitted to thematic content analysis. The study was divided into the following categories: “*You have to be careful*”, “*have to do your exams every month*”, “*every year to stay healthy*”, respondents show access to knowledge on how to prevent high blood pressure, citing changes in habits beyond just use of prescribed medication. Respondents indicated that access to knowledge and information in the prevention of hypertension happens through media, health professionals, consultations, lectures, television, and conversation groups. The orientation of the unit's health team contributes to changes in the lifestyle of the elderly. Therefore, it is concluded that access to health education and encouraging changes of habits in daily life are required for the acquisition of knowledge, assisting in self-care.

**Keywords**

Access, arterial hypertension, elderly

#### O45 Community Health screenings and self-reported chronic diseases

##### Sílvia Monteiro^1^, Ângelo Jesus^1^, Armanda Colaço^1,2^, António Carvalho^1,2^, Rita P. Silva^1,3^, Agostinho Cruz^1^

###### ^1^Escola Superior de Tecnologia da Saúde, Instituto Politécnico do Porto, 4400-330 Vila Nova de Gaia, Portugal; ^2^Centro Hospitalar de São João, EPE, Porto, 4200-319 Porto, Portugal; ^3^Unidade Local de Saúde de Matosinhos, EPE, 4454 509 Senhora da Hora, Portugal

####### **Correspondence:** Sílvia Monteiro – Escola Superior de Tecnologia da Saúde, Instituto Politécnico do Porto, 4400-330 Vila Nova de Gaia, Portugal; Ângelo Jesus (acj@estsp.ipp.pt) – Escola Superior de Tecnologia da Saúde, Instituto Politécnico do Porto, 4400-330 Vila Nova de Gaia, Portugal

**Background**

Community Health screenings are an important part of the Pharmacy Technician’s role as a health care provider. Objectives: To evaluate the relation between anthropometric, physiological and biochemical parameters and self-reported chronic conditions during community health screenings.

**Methods**

The authors conducted an exploratory study including 60 individuals: 63.3 % had one or more chronic diseases and 36.7 % were healthy individuals. For anthropometric measurements we obtained height, weight, body mass index, waist circumference, body fat percentage and muscle mass; for physiological assessment, blood pressure was measured; biochemical variables evaluated were blood glucose, cholesterol and triglycerides in point-of-care testing.

**Results**

Self-reported chronic diseases consisted of 30 % cardiovascular diseases, 26.7 % metabolic diseases, and 16.7 % of other diseases. Cardiovascular patients had abnormal values of systolic blood pressure, triglycerides and body fat. Patients with metabolic disorders showed considerable differences in systolic blood pressure, blood sugar and central adiposity compared to healthy individuals; individuals with obesity revealed high levels of blood pressure, cholesterol, triglycerides, body mass index, waist circumference and body fat percentage. There were a significant number of patients with abnormal values that were neither diagnosed nor medicated.

**Conclusions**

Community health screening is of major importance for patients’ awareness of chronic diseases, and a fundamental role for Pharmacy Technicians. These results show the need for further action with patients, in order to promote a correct follow-up with other health care providers.

**Keywords**

Chronic disease, Community Pharmacy Services, Pharmacy Technician, Metabolic disease, Cardiovascular disease, Obesity

#### O46 Evaluation of indoor air quality in Kindergartens

##### Ana Ferreira, Catarina Marques, João P. Figueiredo, Susana Paixão

###### College of Health Technology, Polytechnic Institute of Coimbra, São Martinho do Bispo, 3046-854 Coimbra, Portugal

####### **Correspondence:** Ana Ferreira (anaferreira@estescoimbra.pt) – College of Health Technology, Polytechnic Institute of Coimbra, São Martinho do Bispo, 3046-854 Coimbra, Portugal

The indoor air pollutants may cause several effects on human health, although there is a greater severity in risk groups, particularly among children. The aim was to evaluate the Indoor Air Quality in kindergartens of the Coimbra city, its structural and functional conditions, and respiratory health of its occupants.

The study evaluated the air quality of 4 kindergartens, both inside and outside the rooms. Air carbon dioxide (CO_2_), carbon monoxide (CO), particles PM10 and PM2.5 volatile organic compounds (VOCs), formaldehyde (H_2_CO), temperature (T,°C), relative humidity (Hr) and Velocity were evaluated.

The results showed that on average every single parameter of the sampled parameters exceed the limits set by legislation. However, it was found that the maximum value of some parameters was equal or exceeded the reference value, among them VOCs, H_2_CO, T°C, Hr and Velocity. According to the occupants' respiratory health, it appears that there is a relationship between the concentration of pollutants and the frequency of disease/symptoms perceived by employees.

**Keywords**

Indoor Air Quality, kindergartens, health

#### O47 Atmospheric exposure to chemical agents under the occupational activity of pathology technicians

##### Ana Ferreira, Carla Lopes, Fernando Moreira, João P. Figueiredo

###### College of Health Technology, Polytechnic Institute of Coimbra, São Martinho do Bispo, 3046-854 Coimbra, Portugal

####### **Correspondence:** Ana Ferreira (anaferreira@estescoimbra.pt) – College of Health Technology, Polytechnic Institute of Coimbra, São Martinho do Bispo, 3046-854 Coimbra, Portugal

In anatomical pathology laboratories (APLs) the presence of several chemical agents and other pollutants is common. These have repercussions in air quality, representing a risk factor for the health of the workers who handle them daily. In this regard, the occupational exposure of 19 anatomical pathology technicians of 3 APLs in the centre region of Portugal to air pollutants was evaluated: formaldehyde (CH_2_O), volatile organic compounds (VOCs) and particles (PM2.5 and PM10).

The indoor air quality (IAQ) was evaluated by direct reading equipment regarding the referred pollutants, as well as temperature, relative humidity and air velocity. In addition to these measurements, questionnaires were distributed to obtain data regarding personal/professional history of workers, occupational exposure characterization, APTs’ health conditions and tobacco consumption data.

The study was of the observational type, descriptive-correlational and cross-sectional (cohort). The type of sampling was non-probabilistic. To proceed with the analytical collection of the evaluated parameters, portable equipment of real-time reading was used, namely the Q-TrakTM Plus – IAQ Monitor gauge, label TSI, model 8552/8554, the Phocheck + gauge, ion science (to measure total VOCs), the Lightouse gauge, model Handheld 3016 IAQ to collect the quantitative values of PM2,5 e PM10 and the PPM Formaldemeter TM htV – IAQ Monitor gauge to evaluate the concentration of CH_2_O.

The results led to the assessment that in all APLs situations of exposure above the protection threshold were verified. It was concluded it is necessary the implementation of safety and control measures that minimize the risk of exposure to air pollutants.

**Keywords**

Anatomical pathology laboratories, occupational exposure, air pollutants

#### O48 Occupational exposure to air pollutants in night entertainment venues workers

##### Ana Ferreira, Diana Ribeiro, Fernando Moreira, João P. Figueiredo, Susana Paixão

###### College of Health Technology, Polytechnic Institute of Coimbra, São Martinho do Bispo, 3046-854 Coimbra, Portugal

####### **Correspondence:** João P. Figueiredo (jpfigueiredo@estescoimbra.pt) – College of Health Technology, Polytechnic Institute of Coimbra, São Martinho do Bispo, 3046-854 Coimbra, Portugal

Given the significant increase of time spent by the population in nightlife venues and the lack of indoor air quality (IAQ), there is a need to study the possibility that this deprivation might affect the employees’ health through their exposure to this environment.

The main goal of this study consisted in assess workers’ occupational exposure to environmental indoor air pollutants, in order to understand its risk and interaction with people’s health.

Therefore, a data collection was held through the measurements of concentration levels: volatile organic compounds (VOC’s), carbon dioxide (CO_2_), particles PM2.5 and PM10, in three different night-time entertainment venues situated in the North of Portugal. For this study, a sample constituted by 14 employees from those three nightclubs was asked important and necessary questions to the execution of this research.

The pollutants evaluated were the VOC’s with use of the equipment branded as ION SCIENCE, model: Phocheck + 2000 – Fristcheck; CO2 with TSI equipment, model 8558552/8554, Q-TrakTM Plus – IAQ Monitor with electrochemical cell direct reading; formaldehyde using the handheld meter PPM Formaldemeter TM htv, 3 Parameter – IAQ Monitor and finally the PM2,5 e PM10 were evaluated with a Lighthouse Worldwide Solutions equipment, model: Handheld 3016-IAQ.

The results show that they infringe extensively the maximum concentration values set by law; one of the reasons is that the artificial air vents are constantly turned off. Despite the strong evidences found in this research it was not possible to associate the employees’ symptoms caused by pollutants with their occupational exposure.

**Keywords**

Indoor Air Quality, Human health, Occupational exposure

#### O49 Beliefs and attitudes of young people towards breastfeeding

##### Telma Fernandes^1^, Diogo Amado^1^, Jéssica Leal^1^, Marcelo Azevedo^1^, Sónia Ramalho^1,2^

###### ^1^Escola Superior de Saúde de Leiria, Instituto Politécnico de Leiria, 2411-901 Leiria, Portugal; ^2^School of Health Sciences, Polytechnic institute of Leiria, 2411-901 Leiria, Portugal

####### **Correspondence:** Sónia Ramalho (sonia.ramalho@ipleiria.pt) – Escola Superior de Saúde de Leiria, Instituto Politécnico de Leiria, 2411-901 Leiria, Portugal

**Background**

Breastfeeding is a priority of global strategy for women’s, children’s and adolescents’ Health: 2016-2030 (World Health Organization, 2015) [1]. Objectives: To understand beliefs and attitudes and knowledge of young people towards breastfeeding and to assess the relation between the beliefs and attitudes of young people towards breastfeeding and sociodemographic and academic variables.

**Methods**

Quantitative, cross-sectional and correlational study with a sample of 177 first-year students of the degree courses of a Portuguese School of Health Sciences. A questionnaire with sociodemographic and academic data and "Adolescent breastfeeding beliefs and intentions questionnaire” validated for the Portuguese population by Catarino, Henriques and Dixe (2011) [2]. Ethical and legal procedures were followed.

**Results**

These young people have an average age: X = 18.89, s = 1.56, and 80.8 % were female. 55.4 % live in rural areas, 54.2 % obtained information about breastfeeding on television. It was found the level of beliefs and attitudes (X = 82.59, s = 5.75) and perceived social barriers (X = 16.46, s = 4.57) for breastfeeding is low. There are significant differences between the beliefs and attitudes towards breastfeeding and gender (z = 1374.0 and p < 0.001). Beliefs and attitudes are mainly influenced by health professionals and the perception of social barriers is influenced by family and magazines.

**Conclusions**

This study confirms the importance of early intervention, the need for specific training in breastfeeding and developing strategies to promote and support breastfeeding in the community.

**References**

1. World Health Organization. National Breastfeeding Policy and Action Plan: 2015-2020. Malta: Health Promotion and Disease Prevention Directorate; 2015.

2. Catarino H, Henriques C, Dixe M. Escala de crenças e atitudes dos adolescentes face à amamentação: validação para a população portuguesa. International Journal of Developmental and Educational Psychology. 2011; 1(2):179-186.

**Keywords**

Beliefs, attitudes, young, breastfeeding

#### O50 Profiling informal caregivers: surveying needs in the care of the elderly

##### Catarina Mangas^1^, Jaime Ribeiro^2^, Rita Gonçalves^3^

###### ^1^Unidade de Investigação Inclusão e Acessibilidade em Acção "iACT" e Escola Superior Educação e Ciências Sociais, Instituto Politécnico de Leiria; 2411-901 Leiria, Portugal; ^2^Unidade de Investigação em Saúde, Escola Superior de Saúde de Leiria eCADR& Centro de Investigação Didática e Tecnologia na Formação de Formadores, Universidade de Aveiro, 3810-193 Aveiro, Portugal; ^3^Instituto Politécnico de Leiria, 2411-901 Leiria, Portugal

####### **Correspondence:** Jaime Ribeiro (jaime.ribeiro@ipleiria.pt) – Unidade de Investigação em Saúde, Escola Superior de Saúde de Leiria eCADR& Centro de Investigação Didática e Tecnologia na Formação de Formadores, Universidade de Aveiro, 3810-193 Aveiro, Portugal

Currently, in Portugal and in the world, a marked increase has been observed in the number of elderly and their longevity. Due to this phenomenon, more studies on ageing and all associated topics are emerging. Informal caregivers arise due to concern about the welfare and health of the elderly. They devote their time, often full-time, in providing care for their relatives.

This descriptive-exploratory study aimed to understand and describe the difficulties faced by each caregiver, their motivations, concerns, and how to overcome them. For this, through a mixed methods approach, 14 interviews were carried out with 14 primary informal caregivers and questionnaires to 30 secondary and tertiary informal caregivers in the municipality of Leiria.

The quantitative data were analysed using descriptive statistics and qualitative data were subjected to content analysis from the perspective of Bardin with coding in predetermined literature-based categories and also in emerging categories. In this way, we characterized informal caregivers and the elderly in their care and perceived the economic and/or social needs, the implications caused by the provision of care and the level of training of each caregiver. In conclusion, we observed that training is not the biggest need pointed out by informal caregivers, but rather the social, family and economic support that would allow these caregivers less physical and emotional burden, as well as lower personal and professional implications.

**Keywords**

Informal caregivers, elderly people, implications, dependency and training

#### O51 Visual health in teenagers

##### Amélia F Nunes^1^, Ana R. Tuna^1^, Carlos R. Martins^2^, Henriqueta D. Forte^2^

###### ^1^Universidade da Beira Interior, Covilhã, 6201-001, Portugal; ^2^Unidade de Saúde Pública, Agrupamento de Centros de Saúde Cova da Beira, 6200-251 Covilhã, Portugal

####### **Correspondence:** Amélia F Nunes (amnunes@ubi.pt) – Universidade da Beira Interior, Covilhã, 6201-001, Portugal

**Background**

The advance of education levels causes a greater visual effort in school requirements and the teenagers are likely to present signs and symptoms associated with vision disorders. This can be reduced or avoided with the adoption of healthy visual habits. Objective: To estimate the frequency of the more common visual changes in adolescence, and to design strategies that promote prevention of visual health in this age group.

**Methods**

A pilot study implemented in two schools in the centre of the country: one is in the city and the other one is in the suburb. It was applied a screening protocol, designed to identify refractive errors that weren’t corrected and changes in binocular vision (focus and convergence system). The size of the sample was 303 teenagers between 12 and 15 years, 158 were boys and 145 were girls.

**Results**

Around 10 % of teenagers have a refractive error uncorrected, 35 % have binocular vision disorders and 55 % have normal binocular vision. The frequencies between rural areas and the suburbs, are significantly different when we talk about refractive errors, there are higher frequencies in rural areas.

**Conclusions**

It was found that the changes in binocular system, are the most common visual changes and these are closely related with the excess of use of the near vision. Knowing the relationship between these changes and the higher risk of development myopia, it is important to implement preventive strategies to adopt appropriate vision and posture habits to relieve stress on the near vision.

**Keywords**

Binocular Vision, Health Vision, Eye care, Teens' Vision

#### O52 Amenable mortality and the geographic accessibility to healthcare in Portugal

##### Cláudia Costa^1^, José A. Tenedório^2^, Paula Santana^1^

###### ^1^Centre of Studies on Geography and Spatial Planning, University of Coimbra, 3000-043 Coimbra, Portugal; ^2^Research Centre for Geography and Regional Planning, Universidade Nova de Lisboa, 1069-061 Lisboa, Portugal

####### **Correspondence:** Cláudia Costa (claudiampcosta@gmail.com) – Centre of Studies on Geography and Spatial Planning, University of Coimbra, 3000-043 Coimbra, Portugal

**Background**

Amenable mortality (premature deaths that should not have occurred given the availability of healthcare) is an important public health theme in Europe and Portugal is one of the European countries with the highest rates. One of the causes may be the time people need to reach the closest hospital. The aim of this paper is to identify the association between geographic accessibility to the nearest hospital in mainland Portugal and the geography of amenable mortality which occurred between 2009 and 2013.

**Methods**

Three methods were applied: To measure geographic accessibility, the Municipal Time-Weighted Accessibility; to analyse amenable mortality, a hierarchical Bayesian model was used to calculate the smooth Standardized Mortality Ratio. Finally, we applied an ecological regression model to identify the association between both.

**Results**

Three major results came out from this study: 1. The mortality amenable to healthcare presents low values in the north and high values in the south; 2. On average, the population has good accessibility to the closest hospital; and 3. For each additional ten minutes required to travel to the nearest hospital, the risk of dying from an amenable cause of death increases by 0.3 % and the relative risk of those living over 30 minutes away from a hospital, is 13 % greater.

**Conclusions**

Geographic accessibility to healthcare services has an important role in the health of the Portuguese population. As such, it is important to implement policies and interventions that reduce the gap between the place of living and healthcare services.

**Keywords**

Amenable Mortality, geographic accessibility, healthcare services

#### O53 Bacterial contamination of door handles in a São Paulo See Metropolitan Cathedral public restrooms in Brazil

##### J. A. Andrade^1^, J. L. Pinto^2^, C. Campofiorito^1^, S. Nunes^1^, A. Carmo^2^, A. Kaliniczenco^1^, B. Alves^3^, F. Mendes^4^, C. Jesus^4^, F. Fonseca^3^, F. Gehrke^1^

###### ^1^Biomedical Sciences Department, Paulista University, São Paulo, 04026-002, Brazil; ^2^Pharmacy Department, Paulista University, São Paulo, 04026-002, Brazil; ^3^Molecular Laboratory of Diagnosis, ABC Medical School, Santo André, São Paulo, 09080-650, Brazil; ^4^Biomedical Science Department, College of Health Technology of Coimbra, Polytechnic Institute of Coimbra, São Martinho do Bispo, 3046-854 Coimbra, Portugal

####### **Correspondence:** F. Mendes (fjmendes@estescoimbra.pt) – Biomedical Science Department, College of Health Technology of Coimbra, Polytechnic Institute of Coimbra, São Martinho do Bispo, 3046-854 Coimbra, Portugal

**Background**

Increasing incidence of epidemic outbreaks of certain diseases and its rate of spread from one community to the other has become a major public health concern. Door handles are heavily contaminated with microbes of faecal origin. Enterobacteriaceae are a large family of gram-negative, facultative anaerobic, rod-shaped bacteria usually motile. In humans, disease is produced by invasive action and toxin production. Species not normally associated with disease are often opportunistic pathogens. Objective: To determine the composition of Enterobacteriaceae communities associated with door handle restroom surfaces.

**Methods**

35 door handles were analysed using sterile cotton swabs to obtain specimens from door handles, were streaked on MacConkey and CLED agar plates and incubated for a 24 h/48 h period at 37 °C. After incubation, the number of resulting colonies was counted and characterized based on margin, elevation, and colony shape. Gram-stain procedure was used to assist in the separation of bacterial cells. After a macro- and microscopic study was performed, colonies were analysed in regard to their biochemical characteristics.

**Results**

In 35 swab samples cultured, 80 % were positive. Isolation differentiated bacteria arranged according their percentage as *Enterobacter* 32 %, *Escherichia coli* 25 %, *Edwardsiella sp.* 14 %, *Proteus vulgaris* 11 %, *Klebsiella sp.* 7 %, *Klebsiella pneumoniae* 4 %, *Citrobacter* 4 % and *Morganella morgani* 4 %.

**Conclusions**

These results imply that Gram-negative organisms can be transmitted through door handles. Three of these bacterial species are classified as fecal coliform that can cause serious diseases. Preventive and inspection measures should be strengthened so that potential hazards individuals are daily exposed to can be avoided.

**Keywords**

Enterobacteriaceae infections, door handles, public restrooms, public health, education

#### O54 Adherence of patients to rehabilitation programmes

##### Carlos Albuquerque^1^, Rita Batista^2^, Madalena Cunha^1^, António Madureira^1^, Olivério Ribeiro^1^, Rosa Martins^1^

###### ^1^Escola Superior de Saúde, Instituto Politécnico de Viseu, 3504-510 Viseu, Portugal; ^2^Hospital Sousa Martins, 6300-858 Guarda, Portugal

####### **Correspondence:** Carlos Albuquerque (cmalbuquerque@gmail.com) – Escola Superior de Saúde, Instituto Politécnico de Viseu, 3504-510 Viseu, Portugal

**Background**

Sticking with a rehabilitation programme is still a source of concern to all healthcare professionals. However, the default rates associated with participation in rehabilitation programmes are still very high. So, regarding this subject, the main goal of this study was to determine the influence of sociodemographic and labour factors in healthcare professionals’ perception towards patient adhesion to rehabilitation treatments.

**Methods**

Thus, we conducted a cross-sectional, descriptive-correlational study with a non-probabilistic sample of 98 healthcare professionals, 58.16 % females, with a mean age of 39.80 years. They answered questions about socio-demographic profiles in order to measure the scale of patient adhesion to rehabilitation programmes.

**Results**

The score of healthcare professionals towards patient adhesion to rehabilitation programmes is 6.48, a value above average. Study results also reveal that strategies used by specialist nurses are more likely to improve patient adhesion than those used by doctors. When it comes to the methods used to promote patient adhesion there is a higher score in cardio-respiratory pathology when compared to the ones used in trauma and rheumatology. Healthcare professionals who work with neurology patients have a higher score than the ones who work with trauma and cardio-respiratory patients.

**Conclusions**

Study results suggest that there is still a long way to go when it comes to finding strategies to improve patient adhesion to rehabilitation programmes. Study findings also suggest that academic contents should be reviewed, as well as promoting public awareness campaigns to encourage patients to keep up with their rehabilitation programmes.

**Keywords**

Adhesion, rehabilitation programs, healthcare professionals

#### O55 Prevalence of malnutrition among Portuguese elderly living in nursing homes: preliminary results of the PEN-3S project

##### Teresa Madeira^1,2,3^, Catarina Peixoto-Plácido^1,2,3^, Nuno Santos^1,2^, Osvaldo Santos^1,2,3^, Astrid Bergland^4^, Asta Bye^4^, Carla Lopes^1,2^, Violeta Alarcão^1,2,3^, Beatriz Goulão^1,2^, Nuno Mendonça^1,5^, Paulo Nicola^1,2,3^, João G. Clara^1,2,3^

###### ^1^Instituto de Medicina Preventiva e Saúde Pública, Faculdade de Medicina, Universidade de Lisboa, 1649-028 Lisboa, Portugal; ^2^Instituto de Saúde Ambiental, Faculdade de Medicina, Universidade de Lisboa, 1649-028 Lisboa, Portugal; ^3^Faculdade de Medicina, Universidade de Lisboa, 1649-028 Lisboa, Portugal; ^4^Oslo and Akershus University College of Applied Sciences, 0167 Oslo, Norway; ^5^Newcastle University Institute for Ageing, Newcastle upon Tyne, NE4 5PL UK

####### **Correspondence:** Teresa Madeira (anateresamadeira@gmail.com) – Instituto de Medicina Preventiva e Saúde Pública, Faculdade de Medicina, Universidade de Lisboa, 1649-028 Lisboa, Portugal

**Background**

Malnutrition is a modifiable risk factor for several diseases among elderly. To develop appropriate nutrition policies to decrease malnutrition, up-to-date information on the dimension of the problem is warranted. Objective: To estimate the prevalence of malnutrition among Portuguese elderly (65+ years old) resident in nursing homes.

**Methods**

This nationally representative cross-sectional study collects data through computer-assisted face-to-face structured interviews and anthropometric measurements. Elderly nursing homes were randomly selected (national-wide sampling). In each home, all elderly with no severe pathologies and who were not bedridden were interviewed. Nutritional status was assessed with the Mini Nutritional Assessment (MNA®) and body mass index was calculated.

**Results**

Overall, 660 elderlies (mean age: 84.3 years; SD = 6.9) participated in the study (60 % of the target sample size). Undernutrition affects 5.6 % (95 % CI: 3.9-7.4) of them and 38.9 % (95 % CI: 35.2-42.7) are at risk of it. Undernutrition was significantly more prevalent among women (5.9 %) than men (4.8 %) and significantly increased with age (1.2 % for 65-74 years old, 4.6 % for 75-84 years old, and 7.6 % for 85+ years old). On the other hand, 27.4 % (95 % CI: 24.0-30.8) of the elderly were considered obese, with significant differences between genders (33.3 % for women; 24.9 % for men) and age groups (43.4 % for 65-74 years old, 37.7 % for 75-84 years old, and 22 % for 85+ years old).

**Conclusions**

Preliminary results reveal a high prevalence of malnutrition (both under- and over nutrition) among elderly living in nursing homes. This highlights the importance of developing an electronic surveillance system for early detection of malnutrition in the elderly.

**Keywords**

Malnutrition, undernutrition, obesity, elderly, nursing home

#### O56 Relation between emotional intelligence and mental illness in health students

##### João Gomes^1,2^, Ana Querido^2^, Catarina Tomás^2^, Daniel Carvalho^1,2^, Marina Cordeiro^2^

###### ^1^Hospital de Santo André, Centro Hospitalar de Leiria, 2410-197 Leiria, Portugal; ^2^School of Health Sciences, Polytechnic institute of Leiria, 2411-901 Leiria, Portugal

####### **Correspondence:** João Gomes (joao.gomes@ipleiria.pt) – Hospital de Santo André, Centro Hospitalar de Leiria, 2410-197 Leiria, Portugal

**Background**

Emotional ability to perceive, use, understand and manage emotions has been associated with mental health. Objectives: Analyse the differences in emotional intelligence (EI) due to presence of mental illness; Correlate EI and perceived mental health knowledge.

**Methods**

Cross-sectional correlational study, in a non-probabilistic sample of 672 Portuguese health students from Dietetics, Nursing, Physiotherapy, Speech Therapy, Occupational Therapy. We applied a sociodemographic questionnaire and Emotional Intelligence Scale (WLEIS-P). Students were mostly women (85.4 %), mean age of 21.16 (±4.17), 6.0 % with a diagnose of mental illness, 36.9 % reporting family with mental illness.

**Results**

Globally, students revealed good EI. Students with mental illness scored lower in EI on subscale of self-appraisal emotions (p = 0.000) and emotional regulation (p = 0.015). However, students with mental illness revealed higher “emotional appraisal of others” compared to the ones without mental illness (p = 0.000). There are no differences in EI related to mental illness in family. Positive significant correlations were found between perceived knowledge in mental health and EI (p = 0.039) as well as regulation of emotions (p = 0.039).

**Conclusions**

Health students with mental illness have lower emotional self-appraisal and regulation of emotional than students without mental illness. Nevertheless, students with mental illness also have better ability to emotional appraisal of others, suggesting a bigger awareness to emotional suffering of others. Correlation between mental health knowledge and EI points out the need of enhancing mental health education to improve emotional intelligence.

**Keywords**

Emotional Intelligence, mental health, students

#### P1 Fall risk factors in people older than 50 years old – a pilot report

##### Marlene C. Rosa^1^, Alda Marques^2^

###### ^1^Secção Autónoma das Ciências da Saúde, Universidade de Aveiro, 3810-193 Aveiro, Portugal; ^2^School of Health Sciences, University of Aveiro, 3810-193 Aveiro, Portugal

####### **Correspondence:** Marlene C. Rosa (marlenerosa@ua.pt) – Secção Autónoma das Ciências da Saúde, Universidade de Aveiro, 3810-193 Aveiro, Portugal

**Background**

Falls are a major public health problem worldwide that has been mainly studied in people older than 65 years old however, recently it has been demonstrated that this problem might affect people at an earlier age. Objective: To determine fall risk factors in a wider range of age interval.

**Methods**

People older than 50 years were recruited in a primary care centre of Portugal. Age, gender, falls history over the past 2 months, comorbidities, need of ambulatory aid and gait difficulties were collected using Morse scale. Independent t-tests and Chi-square tests were used to examine statistical differences (p < 0.05) in these characteristics between fallers and non-fallers. A logistic regression model was built using as independent variables the statistically different characteristics between the two groups and as dependent variable the fall status (fallers or non-fallers).

**Results**

One hundred and forty participants were included (90 female; 71.33 ± 9.97 years; 31.4 % fallers). Fallers were statistically different from non-fallers in: age (74.0 ± 9.9 years vs 69.9 ± 9.9 years, p = 0.03), gender (77.3 % vs 58.5 % female; p = 0.01); incidence of multiple comorbidities (70.5 % vs 48.0 %, p = 0.01); need of ambulatory aid (55.0 % vs 17.0 %, p = 0.01) and incidence of gait difficulties (79.5 % vs 44.7 %, p = 0.01). The odds of falling increased 2.6 in females (95 % CI 1.1-6.5), 3.5 in people that need ambulatory aid (95 % CI 1.4-8.4) and 3.3 in people with gait difficulties (95 % CI 1.3-8.6). These three factors significantly contributed to explain 27.2 % of falls history variability (p = 0.01).

**Conclusions**

Gait impairments and female gender seem to explain almost 30 % of fall risk in people older than 50 years.

**Keywords**

Falls, risk factors, middle-aged, elderly

#### P2 What about the Portuguese oldest old? A global overview using census data

##### Daniela Brandão^1,2^, Óscar Ribeiro^1,3^, Lia Araújo^1,4^, Constança Paúl^1,5^

###### ^1^Unidade de Investigação e Formação sobre Adultos e Idosos, Instituto de Ciências Biomédicas Abel Salazar, Universidade do Porto, 4050-313 Porto, Portugal; ^2^Faculdade de Medicina da Universidade do Porto, 4200-450 Porto, Portugal; ^3^Universidade de Aveiro, 3810-193 Aveiro, Portugal e Instituto Superior De Serviço Social Do Porto, 44600-362 Sra. da Hora, Portugal; ^4^Escola Superior de Educação de Viseu, 3504-501 Viseu, Portugal; ^5^Center for Health Technology and Services Research, Faculdade de Medicina, Universidade do Porto, 4200-450 Porto, Portugal

####### **Correspondence:** Daniela Brandão (daniela.brandao@unifai.eu) – Unidade de Investigação e Formação sobre Adultos e Idosos, Instituto de Ciências Biomédicas Abel Salazar, Universidade do Porto, 4050-313 Porto, Portugal

**Background**

The older population is itself aging, and achieving an advanced age is becoming more common worldwide. In Portugal, individuals aged 80+ represent 5.6 % of the total population and 26.5 % of the population were aged 65 and over in 2011. Having a national profile on this population will give important information to develop intervention programs and identify the areas requiring most attention. Objective: This study aims to provide a profile of the Portuguese oldest old, as given by the last national census data.

**Methods**

The characteristics of all residents aged 80+ (N = 532,219) were analysed considering socio-demographic information (gender, marital status, education, type of residence, place of birth, income) and the existence of difficulties in functional, sensorial and cognitive activities due to health problems or ageing.

**Results**

The majority of the most aged are females (64.5 %), widowed (53.9 %), and present low educational levels (46.1 % never attended school and 31.6 % do not know how to read/write). Own pensions constitute the main source of income (96.3 %) and the majority live in private households (88.8 %), with 43.2 % currently living in the place where they were born. The majority (73.0 %) reported major difficulties in at least one functional activity – bathing/dressing, walking/climbing stairs, seeing, hearing, memory/concentration, understanding others/being understood.

**Conclusions**

The high percentage of oldest old living in private households and the presence of functional limitations point to the importance of informal care and community care services to support this population. Further studies paying attention to their needs and utilisation of services are required.

**Keywords**

Oldest old, census, Portuguese, sociodemographic profile

#### P3 Prevalence of injuries in senior amateur volleyball athletes in Alentejo and Algarve clubs, Portugal: factors associated

##### Beatriz Minghelli^1,2^, Sylvina Richaud^2^

###### ^1^Research in Education and Community Intervention, Escola Superior de Saúde Jean Piaget, Silves, 8300-025, Portugal; ^2^Escola Superior de Saúde Jean Piaget, Silves, 8300-025, Portugal

####### **Correspondence:** Beatriz Minghelli (beatriz.minghelli@silves.ipiaget.pt) – Research in Education and Community Intervention, Escola Superior de Saúde Jean Piaget, Silves, 8300-025, Portugal

Volleyball practice leads to more precise movements and imposes a higher injury risk on the athletes. The study investigates the prevalence of injuries in volleyball athletes in amateur clubs in the Alentejo and Algarve regions and factors associated.

The sample consisted of 52 athletes aged between 18 and 47 years, where 25 (48.1 %) were male.

A questionnaire was used with questions about aspects related to sports, about injury mechanism and techniques to be used at the time of injury. The athlete answered about the most frequent injury (FI) related with the practice and/or the injury that most limited (LI) and/or prevented them from practicing their sports activity (SA).

Thirty-eight (73.1 %) athletes reported having suffered some type of injury, 29 (55.8 %) referring to FI and 29 (55.8 %) LI. The most prevalent type injury was ligament (27.6 % for FI and 44.8 % LI). The most affected body areas in FI were shoulders, hands and fingers and ankles (27.6 % each) and the LI were the ankle (24.1 %) and knee (20.7 %).

The most common injury mechanism was the impact with the ball and repetitive movements (20.7 % each) for FI and LI. Females had 1.33 times (95 % CI: 0.38-4.58; p = 0.648) more probability of having an injury, and older athletes (>25 years) had 1.88 (95 % CI: 0.51-6.37; p = 0.362). Athletes with less practice of the activity (up to 5 years) had 1.154 (95 % CI: 0.300-4.43; p = 0.835), those who train over 2 hours had 1.289 (85 % CI: 0.324-5.122; p = 0.718), and those who are not in net line positions had 1.174 (95 % CI: 0.329-4.189; p = 0.805).

A high prevalence of injuries was observed.

**Acknowledgements**

Authors wish to acknowledge the financial support of the Portuguese Foundation for Science and Technology (FCT), and of the programme COMPETE - Programa Operacional Factores de Competitividade, QREN – Quadro de Referência Estratégico Nacional.

**Keywords**

Prevalence, injury, volleyball, risk factors

#### P4 Shame feelings and quality of life: the role of acceptance and decentring

##### Ana L. Mendes, Joana Marta-Simões, Inês A. Trindade, Cláudia Ferreira

###### Cognitive and Behavioural Centre for Research and Intervention, Faculty of Psychology and Education Sciences, University of Coimbra, 3000-115 Coimbra, Portugal

####### **Correspondence:** Ana L. Mendes (analauramendes@live.com.pt) – Cognitive and Behavioural Centre for Research and Intervention, Faculty of Psychology and Education Sciences, University of Coimbra, 3000-115 Coimbra, Portugal

**Background**

Shame is highlighted as a pathogenic phenomenon in well-being and mental health. In fact, although shame has been considered as an adaptive emotion, higher levels of this painful affect are strongly associated with several psychological difficulties and different mental health conditions. However, the association between external shame and psychological quality of life does not seem to be linear. Objectives: The present study thus aims to clarify the role of two emotion regulation processes, decentring and acceptance, in the association between external shame and psychological quality of life.

**Methods**

The sample comprised 359 participants (131 males and 228 females), aged between 18 and 30 years old.

**Results**

The tested model explained 40 % of psychological quality of life and showed excellent model fit indices. Results showed that external shame negatively impacted on psychological quality of life with a direct effect, and also with an indirect effect through the mechanisms of acceptance and decentring. These findings suggest that the impact of external shame on psychological quality of life is partially attenuated by the effects of higher levels of acceptance and decentring abilities.

**Conclusions**

These findings seem to offer significant implications, emphasizing the importance of targeting shame in the promotion of young women’s psychological quality of life, through community interventions that comprise the development of acceptance (the willingness to contact with internal experiences) and decentring (the ability to perceive internal experiences as transitory and subjective events). Furthermore, longitudinal research should study the protective role of these adaptive emotion regulation processes on psychological well-being.

**Keywords**

Shame, emotion regulation, acceptance, decentring, quality of life

#### P5 Assessment of social support during deployment in portuguese colonial war veterans

##### Teresa Carvalho^1,2^, Marina Cunha^1,2^, José Pinto-Gouveia^1^

###### ^1^Centro de Investigação do Núcleo de Estudos e Intervenção Cognitivo-Comportamental, Faculdade de Psicologia e Ciências da Educação, Universidade de Coimbra, 3001-802 Coimbra, Portugal; ^2^Instituto Superior Miguel Torga, Coimbra, 3000-132, Portugal

####### **Correspondence:** Teresa Carvalho (teresacarvalho.psi@gmail.com) – Centro de Investigação do Núcleo de Estudos e Intervenção Cognitivo-Comportamental, Faculdade de Psicologia e Ciências da Educação, Universidade de Coimbra, 3001-802 Coimbra, Portugal

**Background**

Support social during deployment has proved to be a protective factor for psychopathology developed by war Veterans. The Deployment Social Support Scale (DSSS) of the Deployment Risk and Resilience Inventory (DRRI) has been used to evaluate the perception of assistance and encouragement received in the theatre of military operations from fellow unit members and unit leaders. Objectives: This study aimed to translate and adapt to Portuguese the DSSS and explore its factor structure.

**Methods**

Translation and adaptation of the scale were carried out by clinical psychologists, army officers and an English native speaker. Items adequacy and comprehensibility were first tested in a sample of 30 war veterans. Subsequently, 306 Portuguese colonial war veterans completed the DSSS. Test-retest reliability was assessed in a subset of 115 participants. This subgroup completed the DSSS for a second time approximately three weeks after the first administration.

**Results**

Exploratory Factor Analysis (EFA) indicated that the DSSS presented a single-component structure composed by 12 items (the same of the original version). Factor loadings ranged between .67 and .83. Adequate values of internal consistency (α = .92), corrected item-total correlations (ranged from r = .59 to r = .79) and temporal reliability (r = .88) were obtained.

**Conclusions**

The Portuguese version of the DSSS shows adequate psychometric properties. The results corroborated the structure of the DSSS found in the original version. The scale is internally consistent and has a good temporal stability.

**Keywords**

Assessment of social support, Deployment Social Support Scale (DSSS), DRRI, Exploratory factor Analysis, Portuguese Colonial War Veterans

#### P6 Hospitalization for acute viral bronchiolitis of residents in the metropolitan region of Porto Alegre, Southern Brazil, 2012 to 2014

##### Morgana C. Fernandes, Roger S. Rosa, Rita C. Nugem, Luís F. Kranz, Mariana S. Siqueira, Ronaldo Bordin

###### Federal University of Rio Grande do Sul, Porto Alegre, Rio Grande do Sul, 90050-170, Brasil

####### **Correspondence:** Roger S. Rosa (roger.rosa@ufrgs.br) – Federal University of Rio Grande do Sul, Porto Alegre, Rio Grande do Sul, 90050-170, Brasil

**Background**

Acute viral bronchiolitis (AVB) is a disease characterized by acute inflammation of the bronchioles and increased production and secretion of mucus that may be associated with bronchospasm. It mainly affects infants being the most common cause of paediatric hospitalization in the first year of life. Objective: To describe the characteristics of hospitalizations for BVA in the public health system (SUS) of residents 0 to 2 years-old of the Greater Porto Alegre (GPA) region, in southern Brazil, 2012-2014.

**Methods**

Analysis of hospitalizations with first-listed diagnosis ICD-10 J21.0 and J21.8 from the Hospital Information System (HIS)/SUS publicly available. Calculation of indicators by sex, age groups, stay, mortality and spending.

**Results**

There were 7,091 admissions (2,364/year) in SUS for BVA of GPA residents (153.6/10,000 inhabitants/year). Males predominated (4,246 or 59.9 % vs. 2,845 or 40.1 % of females). Admissions of patients up to 1 year-old accounted for 99.2 %. Bronchiolitis by respiratory syncytial virus (ICD-10 J21.0) accounted for 2,226 (31.4 %) hospitalizations. The average length of stay was brief (5.3 days) and the mortality rate was low (0.2 %) having 12 deceased patients (4/year) (5 males and 7 females). The average annual expenditure was $955.7 thousand PPP (Purchasing Power Parity) and the average value per hospitalization $404.33 PPP.

**Conclusions**

As hospitalizations for BVA conditions are sensitive to primary health care interventions, these data can serve as a basis to identify weaknesses in the basic local network of health services.

**Keywords**

Acute viral bronchiolitis, hospitalization, public health system, children, primary care sensitive conditions

#### P7 Falls-risk screening – an opportunity for preventing falls in the elderly from Nordeste

##### Anabela C. Martins^1^, Anabela Medeiros^2^, Rafaela Pimentel^2^, Andreia Fernandes^2^, Carlos Mendonça^2^, Isabel Andrade^1^, Susana Andrade^1^, Ruth L. Menezes^3^

###### ^1^Escola Superior de Tecnologia da Saúde de Coimbra, São Martinho do Bispo, 3046-854 Coimbra, Portugal; ^2^Municipality of Nordeste, Azores, 9630 – Nordeste, Azores, Portugal; ^3^University of Brasilia, Brasília – Distrito Federal, 70910-900, Brasil

####### **Correspondence:** Anabela C. Martins (anabelacmartins@estescoimbra.pt) – Escola Superior de Tecnologia da Saúde de Coimbra, São Martinho do Bispo, 3046-854 Coimbra, Portugal

**Background**

The European Innovation Partnership on Active and Healthy Ageing (EIP on AHA) issued several key documents for launching and implementing a collaborative partnership aiming to increase the average healthy lifespan of Europeans by 2 years by 2020. Through cooperation between all EU countries, evidence-based programmes for the prevention, early detection, risk minimization and management of falls must be implemented. Objective. To identify the population at risk of falling (and of fractures) which may benefit from effective individual interventions.

**Methods**

A descriptive, exploratory study was conducted with 213 participants of a home-dwelling population (72.5 % female), with an average age of 75.85 years, SD = 6.94.

**Results**

The population from Nordeste evidenced a probability of limited mobility within the community, with a mean value of 13.45 sec in the walking speed test. A mean value of 12.43 sec in the Timed Up & Go test indicated a moderate risk of falling. Up to 36 % participants reported having fallen in the last 12 months.

**Conclusions**

All together these results are an opportunity for the development of a programme for fall prevention in the community, in a partnership joining local health and social organizations. This will contribute to the implementation of programmes adapted to the functional and psychological demands of these older people from Nordeste, and also to their daily life activities, in order to enhance strength, walking speed, flexibility, balance and resistance, under the framework of the collaborative initiative “*Dar Vida aos Anos*” of the municipality of Nordeste dedicated to those over 65.

**Keywords**

Falls-risk screening, Home-dwelling, Older adults

#### P8 Aging provokes chronodisruption in mature people in temperature circadian rhythm

##### Rafael Bravo^1^, Marta Miranda^2^, Lierni Ugartemendia^2^, José Mª Tena^3^, Francisco L. Pérez-Caballero^4^, Lorena Fuentes-Broto^5^, Ana B. Rodríguez^2^, Barriga Carmen^2^

###### ^1^Universidad de Extremadura, Badajoz, 06071 Badajoz, España; ^2^Chrononutrition Laboratory, Department of Physiology, Faculty of Science, University of Extremadura, Badajoz, 06071 Badajoz, España; ^3^Department of Anestesiology, Complejo Hospitalario Universitario de Badajoz, 06080 Badajoz, España; ^4^Centro de Salud La Paz, Badajoz, 06011 Badajoz, España; ^5^Aragon Institute for Health Research, Zaragoza, 50009 Zaragoza, España

####### **Correspondence:** Rafael Bravo (rbravo@unex.es) – Universidad de Extremadura, Badajoz, 06071 Badajoz, España

Most physiological functions in the body follow a circadian rhythm. There are several pathologies and biological processes like obesity or aging, among others, that provokes chronodisruption in these rhythms. It is well known that elderly people suffer from chronodisruption in most of their circadian rhythms. Our aim was to elucidate if this trend may begin in earlier stages. Thirty-two 32 people (aged between 40 and 65) participated in this assay. Volunteers wore a wrist temperature logger on their non-dominant hand for 3 days. Data were read in a computer and analysed through Circadianware software. Chronobiological parameters MESOR (the average value around which the variable oscillates), amplitude (difference between the peak and the mean value of the wave) and acrophases (the time at which the peak of a rhythm occurs) were calculated and correlated with age. Our results showed that MESOR (p < 0.05; R = 0.4586), amplitude (p < 0.05; R = - 0.5178) changed with age but not acrophase. We conclude from the present assay that chronodisruption symptoms in temperature circadian rhythm in the elderly begin to be expressed in a mature stage in humans.

**Keywords**

Chronobiology, Chronodisruption, Aging, Temperature, Circadian rhythm

#### P9 The influence of climate and pollution factors in dengue cases of great ABC region, São Paulo

##### M. A. Carneiro^1^, J. N. Domingues^2^, S. Paixão^2^, J. Figueiredo^2^, V. B. Nascimento^1^, C. Jesus^3^, F Mendes^3^, F. Gehrke^4^, B. Alves^1^, L. Azzalis^5^, F. Fonseca^1^

###### ^1^ABC Medical School, Santo André, São Paulo, 09210-180, Brazil; ^2^Department of Complementary Sciences, College of Health Technology of Coimbra, Polytechnic Institute of Coimbra, São Martinho do Bispo, 3046-854 Coimbra, Portugal; ^3^Biomedical Science Department, College of Health Technology of Coimbra, Polytechnic Institute of Coimbra, São Martinho do Bispo, 3046-854 Coimbra, Portugal; ^4^Biomedical Sciences Department, Paulista University, São Paulo, 04026-002, Brazil; ^5^Federal University of São Paulo, Diadema campus, Diadema, São Paulo, 09913-030, Brazil

####### **Correspondence:** C. Jesus (gaspar_c94@hotmail.com) – Biomedical Science Department, College of Health Technology of Coimbra, Polytechnic Institute of Coimbra, São Martinho do Bispo, 3046-854 Coimbra, Portugal

**Background**

Dengue is considered a major public health problem in the world. It is estimated that 80 million people are infected annually in 100 countries on all continents. The global changes that have taken place directly interfere with the natural environment; relating them to the climate and tropical diseases, it can be seen that changes in temperature alter the ecosystem, directly influencing the growth in transmission of diseases caused by vectors, which includes dengue. Objectives: To understand the consequences of temporal variability of climate conditions on the occurrence of dengue in the population of the Greater ABC metropolitan region of São Paulo and characterize the temporal trend of dengue in the region in the period 2010-2013.

**Methods**

Analysis of numbers of dengue cases reported in the years under review, complemented by the meteorological data (temperature and humidity) and pollutant concentration data (PM10). Dengue in the region had a higher incidence in 2010 and lower incidence in 2012.

**Results**

It was found that there is a statistical association between moisture and PM10 with dengue cases reported. Though the temperature does not statistically assume an association with reported dengue cases, it was found that the temperature peaks coincided with the epidemic peak of dengue.

**Conclusions**

High pollutant concentration (PM10) is associated with a decrease in the number of dengue cases. It would be interesting to carry out future studies related to environmental pollution and its influence on the mosquito *Aedes aegypti* development in all phases of its life cycle and developing strategies for better monitoring, campaigns and surveillance.

**Keywords**

Reported Dengue Cases, Vectors, Climatic and Environmental Factors, Particulate Pollutants

#### P10 Visual function and impact of visual therapy in children with learning disabilities: a pilot study

##### Ana R. Martins^1^, Amélia Nunes^1^, Arminda Jorge^2^

###### ^1^Universidade da Beira Interior, Covilhã, 6201-001, Portugal; ^2^Centro Hospitalar Cova da Beira, Covilhã, 6200-251, Portugal

####### **Correspondence:** Ana R. Martins (anarita.m91@gmail.com) – Universidade da Beira Interior, Covilhã, 6201-001 Covilhã, Portugal

**Background**

Undetected visual dysfunctions affect academic performance. Thus it's essential the implementation of programs for visual evaluation/intervention in schoolchildren. Given that children with learning disabilities in reading (LDR) can benefit from early intervention with visual therapy (VT), this study aims to estimate the frequency and distribution of visual impairment in children with LDR, assess the impact of visual disturbances and the influence of VT on their quality of life.

**Methods**

Seventeen children with LDR (9 ± 1 years), followed in speech therapy/educational intervention in the paediatric service of "Centro Hospitalar Cova da Beira" (Portugal), participated in this study. The control group included 103 children without learning disabilities (10 ± 1 years). The visual function evaluation included the application of the Inventory of Visual Efficiency (COVD-QoL questionnaire), and optometric measurement of various parameters of visual function. All children with LDR with altered visual function were advised to conduct a VT plan.

**Results**

Children with LDR showed a higher percentage of visual function alterations (vergencial function, accommodation and ocular motility) and reported more symptoms compared to control group. After a VT plan was registered a significant improvement in most visual parameters assessed, and observed a significant reduction of visual symptoms.

**Conclusions**

This study shows that improvement of objective/subjective parameters of visual function reduce visual symptoms, increasing comfort while carrying out schoolwork, improving the quality of life in LDR children. This study suggests the importance of evaluation of visual function in LDR children, as well as the benefits of VT and the importance of a multidisciplinary approach.

**Keywords**

Learning disabilities, visual function, visual symptoms, visual therapy, school performance

#### P11 Edentulism and the need of oral rehabilitation among institutionalized elderly

##### Nélio Veiga^1,2^, Ana Amorim^1^, André Silva^1^, Liliana Martinho^1^, Luís Monteiro^1^, Rafael Silva^1^, Carina Coelho^1^, Odete Amaral^3,5^, Inês Coelho^4^, Carlos Pereira^3,5^, André Correia^1,2^

###### ^1^Departamento de Ciências da Saúde, Universidade Católica Portuguesa, Viseu, 3504-505, Portugal; ^2^Centro de Investigação Interdisciplinar em Saúde, Universidade Católica Portuguesa, 3504-505 Viseu, Portugal; ^3^Escola Superior de Saúde, Instituto Politécnico de Viseu, 3504-510 Viseu, Portugal; ^4^Unidade de Saúde Familiar Grão Vasco, Viseu, 3500-177, Portugal; ^5^Centro de estudos em educação, tecnologias e saúde, Instituto Politécnico de Viseu, 3504-510 Viseu, Portugal

####### **Correspondence:** Nélio Veiga (nelioveiga@gmail.com) – Departamento de Ciências da Saúde, Universidade Católica Portuguesa, Viseu, 3504-505, Portugal

**Background**

An adequate dentition is important for well-being and increase of quality of life. Despite advances in preventive dentistry, edentulism is still a major public health issue in Portugal. Objectives: Determine the prevalence of edentulism and assess the oral rehabilitation and oral/prosthetic hygiene habits in a sample of institutionalized elderly.

**Methods**

We conducted a cross-sectional study in a sample of 68 institutionalized elderly (79.4 % female), in which the average age was 78.3 ± 12.0 years old. Data collection was performed by applying a questionnaire about oral health behaviours and through an intraoral observation in order to determine the oral status and verify the condition of the oral rehabilitation of each participant.

**Results**

In the present study, 58.8 % presented total edentulism with no natural teeth in the oral cavity and 66.7 % had a removable prosthesis. From the elderly that had a removable prosthesis, only 42.9 % presented satisfactory prosthetic retention and stability. Only 44.1 % refer daily oral/prosthetic hygiene at least twice-a-day. From the total sample, 29.0 % refer having toothache, 58.1 % refer dry mouth and 67.7 % refer difficulty in chewing, even in the case of having a removable prosthesis. The educational level of the elderly was associated with dental pain (p = 0.012) and oral/prosthetic hygiene (p = 0.034).

**Conclusions**

A high prevalence of tooth loss was found in the sample studied and also the need of improvement of the removable prosthesis is fundamental. Improvement in oral healthcare and oral hygiene habits is essential to promote better oral health and quality of life among the institutionalized elderly.

**Keywords**

Edentulism, institutionalized elderly, oral rehabilitation, quality of life.

#### P12 Therapy adherence of outpatients in the pharmacy services of a hospital unit

##### Diana Rodrigues^1^, Nídia Marante^1^, Pedro Silva^1^, Sara Carvalho^1^, André Rts Araujo^1,2^, Maximiano Ribeiro^1,2^, Paula Coutinho^1,2^, Sandra Ventura^1,2^, Fátima Roque^1,2^

###### ^1^School of Health Sciences, Polytechnic Institute of Guarda, 6301-559 Guarda, Portugal; ^2^Research Unit for Inland Development, Polytechnic Institute of Guarda, 6301-559 Guarda, Portugal

####### **Correspondence:** Fátima Roque (froque@ipg.pt) – School of Health Sciences, Polytechnic Institute of Guarda, 6301-559 Guarda, Portugal

The low adherence to therapy of chronic patients undergoing long-term treatment has been identified as a major factor responsible for the lack of effectiveness of prescribed treatments.

In the present study, we have conducted interviews to patients over the age of 18 that acquired their medications in a hospital pharmacy of the north region of Portugal. Compliance with the treatment regimen and self-perception of health status were assessed by applying the Morisky Medication Adherence Scale© (MMA-8-Item) and the The Brief Illness Perception Questionnaire© (IPQ-B), respectively.

The association between gender and some types of disease was observed for Psoriatic Arthritis and for the incidence of breast cancer in women and lung cancer in males. Of the 11 patients, 3 reported to have undergo therapeutic changes from the beginning of treatment. These three correspond to patients with Hepatitis B and Psoriatic Arthritis.

The results of therapy adherence show 90.9 % of adherence, in which 5 of the 11 respondents showed a high degree of adherence to therapy and only 1 revealed lower levels of adherence. According to the MMA-8-Item (n = 11) respondents do not associate the non-adherence to oblivion of taking the medicines. However, the limiting factors of adherence most mentioned were adverse effects associated with the medication and depressive states linked with the negative impact that the disease has on society.

Although this is a pilot study, our results have shown to be promising. Patients reported high adherence rates and it was verified a relationship between therapy adherence and the self-perception of health.

**Keywords**

Therapy adherence, health status, effectiveness

#### P13 Universal access and comprehensive care of oral health: an availability study

##### Cristina Calvo, Manoela Reses

###### Centro de Ciências da Saúde, Departamento de Saúde Pública, Universidade Federal de Santa Catarina, Florianópolis, Santa Catarina, 88040-900 Brasil

####### **Correspondence:** Cristina Calvo (cristina.clv@gmail.com) – Centro de Ciências da Saúde, Departamento de Saúde Pública, Universidade Federal de Santa Catarina, Florianópolis, Santa Catarina, 88040-900 Brasil

The constitutional right to health can be considered as one of the greatest social achievements of the post-democracy period in Brazil. After 25 years of the implementation of the Unified Health System, although undeniable advances have been observed, the guidelines for universal access and comprehensive care in oral health still remain as challenges. In this sense, we performed an availability study (AS) of oral health services with a focus on universal access and comprehensive care.

The EA consists of a set of procedures that precede the stage of evaluation for verifying the extent to which the object can be evaluated. In this study the following steps were taken: (a) description of oral health care by identifying goals and activities; (b) design of the theoretical and logical model; (c) development of an evaluation matrix; (d) identification of stakeholders in the evaluation; and (e) achieving consensus on the evaluation procedures.

The evaluative matrix of oral health care with a focus on universal access and comprehensiveness was composed of five dimensions: accessibility; availability; priority of promotion and prevention actions; articulation of promotion, prevention and recovery; and integral approach of individuals and families. For each dimension indicators were discussed related to the level of dental care: management, primary care, specialized care, urgent and emergency services and hospital care.

The EA proved to be appropriate and allowed the identification of priority areas of oral health care for future evaluations.

**Keywords**

Health services evaluation, access, oral health care services, Integrality in Health, oral health

#### P14 Is the respiratory function of children a predictor of air quality? Coimbra as a case study

##### Jorge Conde, Ana Ferreira, João Figueiredo

###### College of Health Technology, Polytechnic Institute of Coimbra, 340-162 Coimbra, Portugal

####### **Correspondence:** Jorge Conde (jconde@estescoimbra.pt) – College of Health Technology, Polytechnic Institute of Coimbra, 340-162 Coimbra, Portugal

**Background**

Respiratory changes are increasingly present, from an early age, either because life is increasingly sedentary, or because city pollution is increasingly present. Knowing that the circuits of the children of the city of Coimbra, are apparently "clean", we assessed two groups of children, using spirometry in the search for respiratory changes denoting disease. Objective: to assess a population of children representing the municipality of Coimbra, in order to understand possible respiratory changes.

**Methods**

We studied children who attend the 1st grade and those who attend the 4th grade, using spirometry. The children were selected among those who had no history of disease, nor evident symptoms and presented adequate collaboration. The results were stratified to be representative of the population of the municipality of Coimbra.

**Results**

The results show a predominantly healthy population, with children of the 1st grade (6-7 years old), presenting alterations to small and medium airways in 7.0 % of the cases, with girls representing 4.3 % of the sample. Only in 0.4 % of the children were we in the presence of respiratory alterations of the obstructive type. In the 4th grade group (9 to 10 years), the results showed 4.0 % of obstructive alterations in peripheral airways and only in 2.0 % was obstructive respiratory alteration observed.

**Conclusions**

We can say that the population between 6 and 10 years old presents a good level of absence of disease, which cannot be unrelated to good air quality of the city and low level of pollution.

**Keywords**

Spirometry, city pollution, children

#### P15 Meaning-in-life of college students

##### David Silva, Luís Seiça, Raquel Soares, Ricardo Mourão, Teresa Kraus

###### School of Health Sciences, Polytechnic institute of Leiria, 2411-901 Leiria, Portugal

####### **Correspondence:** Teresa Kraus (teresa.kraus@ipleiria.pt) – School of Health Sciences, Polytechnic institute of Leiria, 2411-901 Leiria, Portugal

**Background**

Admission to college and academic life both represent a particularly susceptible period of emotional turmoil. This period can be exacerbated by the organization of the student’s identity, a result of developing maturity. In this context, meaning in life presents itself as a variant with promising effect on the attainment of life goals. Objective: To characterize the sample as to its life goals.

**Methods**

A simple descriptive study, performed during 2015 with students from a college in the centre of Portugal. The sample answered to a survey on Google Docs with open and closed-ended questions, with the study’s goal as its basis. Data processing was accomplished using descriptive statistic means.

**Results**

The sample had 242 subjects, mostly female (78.1 %, n = 189). To the question “More than anything, I want to…”, answers were “be happy” (59.5 %, n = 144); “finish college” (4.1 %, n = 10); “start a family” (2.9 %, n = 7); and “help others” (2.5 %, n = 6). We can point out that a few subjects (12 %, n = 29) referred that they haven’t made any progress in the pursuit of their life goals yet, such as “to be desired”, “to be loved”, “to be happy” and “to enjoy life”.

**Conclusions**

Most of the sample’s subjects recognize they are achieving their goals. Only a minority admits their lack of ability in succeeding. Despite only being a few, they need holistic attention to guide them towards a discovery of a real meaning in life.

**Keywords**

Meaning-in-life, college, students

### Education & Training of Health Professionals

#### O57 Training needs for nurses in palliative care

##### Ana C. Abreu^1^, José M. Padilha^2^, Júlia M. Alves^3^

###### ^1^Oporto Portuguese Institute of Oncology FG, EPE, 4200-072 Porto, Portugal; ^2^Porto Nursing School, 4200-072 Porto, Portugal; ^3^Oporto Central Hospital, 4099-001 Porto, Portugal

####### **Correspondence:** Ana C. Abreu (catarina.abreu19@gmail.com) – Oporto Portuguese Institute of Oncology FG, EPE, 4200-072 Porto, Portugal

**Background**

Demographic, social and health changes are at the origin of the change in the place of death. The characterization of nursing care needs to be documented in the Nursing Information System (NIS) in use, and may represent an effective strategy to define training policies for nurses, for the organization of care as well as for the definition of areas of focus for research. Objective: To describe the main needs for nursing care of palliative patients referred to the Hospital-Support Team in Palliative Care of a hospital in central Portugal.

**Methods**

We conducted an exploratory, descriptive and cross-sectional study, from the documentation of nursing care in the NIS in use. Our sample comprises the accessible population between September 1, 2012 and September 1, 2013, making a total of 276 patients.

**Results**

In this study, 53.99 % of patients have an oncological pathology, despite the increase of referral of non-oncologic pathologies. Concerning the needs of nursing care documented in the NIS, 68 % of nursing focus is given to the dimension of function and 32 % to the dimension of the person. The management and control of symptoms is one of the main focuses in the available evidence, also found in the study with an emphasis on function.

**Conclusions**

Despite the relevance of the physical dimension, it represents only a part of total care, exposing the opportunity for improving nursing care quality, regarding the psychological, social, cultural and spiritual dimensions. The need emerges for appropriate training in the other key areas of Palliative Care, particularly fatigue, grief, family, communication, comfort and spirituality.

**Keywords**

Palliative Care, Nursing Care, Total Care

#### O58 Impact of computerized information systems in the global nurses’ workload: nurses’ perceptions and real-time

##### Paulino Sousa^1,2^, Manuel Oliveira^1^, Joana Sousa^3^

###### ^1^Escola Superior Escola Superior de Enfermagem do Porto, 4200-072 Porto, Portugal; ^2^Center for Health Technology and Services Research, Faculdade de Medicina, Universidade do Porto, 4200-450 Porto, Portugal; ^3^Centro Hospitalar do Porto, 4099-001 Porto, Portugal

####### **Correspondence:** Paulino Sousa (paulino@esenf.pt) – Escola Superior Escola Superior de Enfermagem do Porto, 4200-072 Porto, Portugal

**Background**

During the last decade, health institutions have been involved in the process of moving away from paper-based and implementing electronic health information to support patient care. The documentation is in fact an essential part of nursing practice, but will undoubtedly be responsible for a substantial portion of the overall workload of nurses. We are confronted too often with opinions that contain a perception that the use of computerized information systems (CIS) have a major impact on the overall workload of nurses. Objective: To explore nurses’ perceptions and the real-time of CIS impact on the global nurses’ workload.

**Methods**

A cross-sectional survey was applied to collect data from 190 nurses that use CIS in a hospital. For the analysis of real-time spent on the CIS usage we collected the automated registered times of medicine ward nursing users.

**Results**

Only a quarter (Q1) of nurses believes that the time spent with the use of CIS is less or equal to 30 % of their global workload and 75 % of the nurses (Q3) consider that is less or equal to 50 %, existing an equal dispersion to the right and left of the median (40 %). The average of the real-time spent on CIS use was 16.7 % of nurses’ workload (median: 17.1 % and standard deviation: 3.4).

**Conclusions**

Nurses have the perception that the time used in CIS has a high impact on the workload. However, the analysis of “real-time” shows that the time spent is overlapping the lowest times referenced in the literature.

**Keywords**

Computerized Information Systems, electronic health information, nursing workload, time spent

#### O59 The perspective of health care professionals on self-care in hereditary neurodegenerative disease: a qualitative study

##### Sónia Novais^1^, Felismina Mendes^2^

###### ^1^Instituto de Ciências da Saúde, Universidade Católica Portuguesa, 1649-023 Lisboa, Portugal; ^2^Escola Superior de Enfermagem de S. João de Deus, Universidade de Évora, 7004-516 Évora, Portugal

####### **Correspondence:** Sónia Novais (snovais@gmail.com) – Instituto de Ciências da Saúde, Universidade Católica Portuguesa, 1649-023 Lisboa, Portugal

Familial Amyloidotic Polyneuropathy (FAP) is a rare, irreversible and fatal disease resulting from a genetic mutation with autosomal dominant transmission. This neurodegenerative disorder has late age of onset, after 25 years of age and is a progressive autonomic sensory and motor neuropathy. This disease is characterized by deposition of insoluble amyloid fibrils in connective tissue causing high dependency and premature death. Treatment options for patients with FAP are very limited. Symptomatic treatment can control symptoms and improve the quality of life of these patients.

This work aims to: I) Understand the perspective of health professionals on self-care in FAP; II) Understand which behaviours and self-care needs of patients are perceived by health professionals. This is a part of a larger ethnographic study. A semi-structured interview was conducted with eight privileged witnesses. The collected qualitative data was analysed according to the method proposed by Miles, Huberman and Saldaña.

The results obtained by analysis of the interviews allow us to have two categories, namely self-care needs and self-care behaviours. The needs identified by health professionals primarily relate to the increased level of dependence that occurs with the evolution of this illness.

The self-care behaviours are identified with the completion of presymptomatic testing and the use of medically assisted procreation techniques with pre-implantation testing and selection of free mutation embryos. The health care professionals’ perspective of self-care within the inherited genetic disease is expanded, but is mainly derived from self-care needs and not from self-care behaviours.

**Keywords**

FAP, Self-care, hereditary disease, social illness representation, health care professional

#### O60 Contribution for health-related physical fitness reference values in healthy adolescents

##### Joana Pinto, Joana Cruz, Alda Marques

###### School of Health Sciences, University of Aveiro, 3810-193 Aveiro, Portugal

####### **Correspondence:** Alda Marques (amarques@ua.pt) – School of Health Sciences, University of Aveiro, 3810-193 Aveiro, Portugal

**Background**

Physiotherapy intervenes in health-related physical fitness (HRPF). However, normative values are lacking for prescribing exercise in healthy and non-healthy populations, namely in adolescents. Objective: The primary aim was to contribute for establishing normative values of the measures most commonly used in physiotherapy to assess HRPF. Secondary aims were to examine gender-specific differences and the relationship between HRPF and physical activity (PA).

**Methods**

A cross-sectional study was conducted with Portuguese adolescents. Socio-demographic, anthropometric data and vital signs were first collected. Then, HRPF was assessed through: body mass index (BMI), incremental shuttle walk test (ISWT), quadriceps muscle strength using hand-held dynamometry (HHD), modified sit-and-reach test (MSRT) and timed up and go (TUG). Participants’ PA levels were assessed using the "Physical Activity Index" (PAI).

**Results**

One hundred forty-one (141) adolescents participated (14.33 ± 1.34 years, 71 males). The proposed normative values for HRPF were: BMI 58.95 ± 2.44 %; ISWT 1251.19 ± 26.25 m; HHD 21.04 ± 0.69 Kgf; MSRT 38.09 ± 1.75 cm; TUG 4.31 ± 0.13 s. Male adolescents were significantly (p < 0.001) faster performing TUG (males: 3.75 ± 0.43 s; females: 4.88 ± 0.87 s), walked a superior distance in the ISWT (males: 1409.00 ± 265.63 m; females: 1111.94 ± 251.10 m) and had more quadriceps muscle strength (males: 23.77 ± 7.13 kgf; females: 18.70 ± 6.28 kgf) than females. Adolescents had moderate PA levels. Higher PA levels were associated with better results in the MSRT (r = 0.592, p < 0.001), TUG (r = -0.543, p < 0.001) and ISWT (r = 0.432, p < 0.001).

**Conclusions**

Findings are a contribution for HRPF normative values of tests most commonly used in physiotherapy assessment of adolescents. The associations between PA and HRPF suggest that future work should encourage adolescents to adopt more active lifestyles in order to improve their HRPF performance.

**Keywords**

Physical fitness, cardiorespiratory, muscular strength, neuromuscular, flexibility, physical activity index, adolescents

#### O61 Perception of learning, satisfaction and self-efficacy of nursing students about High-Fidelity Simulation

##### Hugo Duarte, Maria Dos Anjos Dixe, Pedro Sousa

###### Health Research Unit & School of Health Sciences, Polytechnic institute of Leiria, 2411-901 Leiria, Portugal

####### **Correspondence:** Hugo Duarte (hugoduarte2009@gmail.com) – Health Research Unit & School of Health Sciences, Polytechnic institute of Leiria, 2411-901 Leiria, Portugal

**Background**

Over the last years the teaching of nursing has been progressively directed to the use of new methodologies, among which we highlight the High-Fidelity Simulation (HFS), which allows nursing students to have their first contact with the clinical practice within the academic environment. Objective: To evaluate and correlate the perception of learning, satisfaction and self-efficacy of nursing students about HFS.

**Methods**

A correlational study with 139 nursing students was developed, using the Portuguese version of the Nursing Students’ Perceptions of Learning and Learner Satisfaction with Simulation Tool.

**Results**

Nursing students reveal a moderated perception of learning about the HFS (3.649 ± 0.513 out of 5 points), a good level of satisfaction (7.317 ± 1.065 out of 10 points) and a self-efficacy for the practice of HFS (31.590 ± 3.974 out of 40 points). All correlations between perception of learning, satisfaction and self-efficacy of the students regarding HFS were positive and highly significant (p ≤ 0.001), with values between 0.991 and 0.385.

**Conclusions**

This study reveals that nursing students are satisfied with the practice of HFS, as well as shows a positive perception of learning and self-efficacy about HFS. The fact that the correlations among these three variables are highly significant and positive, demonstrate the importance of the practice of HFS within the school environment, which allows the development of experiences of high value for the nursing students.

**Keywords**

High Fidelity Simulation, nursing students, personal satisfaction, self-efficacy, learning perception

#### O62 Analysis of statements of diagnosis about health deviation in self-care requisites customized in a Nursing Practice Support System (SAPE®): Management of therapeutic regimen

##### Inês Cruz, Fernanda Bastos, Filipe Pereira

###### Escola Superior de Enfermagem do Porto, Porto, 4200-072, Portugal

####### **Correspondence:** Inês Cruz (inescruz@esenf.pt) – Escola Superior de Enfermagem do Porto, Porto, 4200-072, Portugal

**Background**

Nowadays chronic diseases are the leading cause of death in Portugal and in the world. Since most of these diseases have behavioural determinants in their origin, and their control depends on self-care behaviours, including an effective management of the therapeutic regimen, this is a key area in nursing. Objective: To identify the terms used by nurses to describe the range of diagnosis customized in the Nursing Practice Support System (SAPE®), focused on the management of the medication regimen.

**Methods**

A qualitative study. A content analysis process of nursing documentation in SAPE® centred on the management of medication regimen was conducted. An a priori analysis model was used – the ISO 18104: 2003 standard – Health informatics: integration of a reference terminology model for nursing.

**Results**

There were 598 statements of nursing diagnosis identified centred on “managing the medication regimen”. After the content analysis process, and after excluding all conceptual redundancy, the statements of nursing diagnosis were reduced to 30 clinically useful syntaxes.

Thirty-two different terms for judgments about the focus were found. After consensus, we reduced these to 2 terms: “impaired” and “potential to enhance”. We found 20 “dimensions” for which the principal focus is put into perspective. After analysis, they were reduced to 6: knowledge; ability; awareness; meaninglessness; support, and family support. Eight judgments associated with dimensions were found.

**Conclusions**

The proliferation of different statements of diagnosis reflect the same reality which makes information management and production of indicators more difficult. Studies that make a conceptual analysis of nursing documentation are necessary.

**Keywords**

Management of therapeutic regimen, Nursing Diagnosis, Nursing Informatics Systems

#### O63 Hybrid management and hospital governance: doctors and nurses as managers

##### Francisco L. Carvalho, Teresa T. Oliveira, Vítor R. Raposo

###### Faculdade de Economia, Universidade de Coimbra, 3004-512 Coimbra Portugal

####### **Correspondence:** Francisco L. Carvalho (edinaldo.lira@gmail.com) – Faculdade de Economia, Universidade de Coimbra, 3004-512 Coimbra Portugal

**Background**

Hybrid systems imply multi-tasking as in post Fordist lean production [1-2] and are relevant to complex organisations such as teaching hospitals [3]. Nonetheless, they raise questions concerning levels of leadership and responsibility [4], group working and relational coordination [5-7] across different domains of practice [8]. Moreover, there is a need to better understand how group operational effectiveness is associated with clarity of job roles [9], workloads and motivation [10] and their impact on the climate and quality of care [11].

**Methods**

A case study in a European university hospital based on a grounded theory approach. Longitudinal data (2011 to 2015) from audio-taped and semi-structured interviews (N = 64) with 41 health professionals as managers (28 doctors and 13 nurses). Content analysis on the basis of a newly developed coding system using MAXQDA software. Originality: Locating the concept of hybrid management in terms of the role of doctors as managers in the context of different logics, i.e. distinguishing institutional logic (an NHS) from organizational logic (a hospital) and operational logic (at unit and service levels).

**Results and Conclusions**

Doctors and nurses as managers need relative autonomy at operational levels which should be recognized by hospital administrators

**References**

1. Womack JP, Jones DT. Lean Thinking. New York: Simon and Schuster;1996.

2. Womack JP, Jones DT. Lean Solutions: How Companies and Customers Can Create Wealth Together. New York: Simon Schuster; 2005.

3. Joffe M, MacKenzie‐Davey K. The problem of identity in hybrid managers: who are medical directors?, International Journal of Leadership in Public Services. 2012, 8(3):161 - 174.

4. Guest D, Bos-Nehles A. HRM and Performance: The Role of Effective Implementation. London: Department of Management King´s College London; 2011.

5. Gittell JH. The Southwest Airlines Way - Using the Power of Relationships to Achieve High Performance. M.-H. Companies. New York: McGraw-Hill Companies; 2003.

6. Gittell JH. Relational Coordination: Guidelines for Theory, Measurement and Analysis. USA: Relational Coordination Research Collaborative; 2011.

7. Gittell JH, Weinberg DB, Bennett A, Miller JA. “Is the Doctor In?” A relational approach to job design and the coordination of work. Human Resource Management. 2008; 47(4): 729-755.

8. Mørk BE, Hoholm T, Maaninen-Olsson E, Aanestad M. Changing practice through boundary organizing: A case from medical R&D. Human Relations. 2012; 65(2):263-288.

9. Woodrow C, Guest DE. Public violence, staff harassment and the wellbeing of nursing staff: an analysis of national survey data. Health Services Management Research. 2012; 25(1): 24-30.

10. Gungor P. The Relationship between Reward Management System and Employee Performance with the Mediating Role of Motivation: A quantitative Study on Global Banks 7th International Strategic Management Conference. 2011;1510-1520.

11. Moran C M, Diefendorff JM, Kim TY, & Liu ZQ. A profile approach to self-etermination theory motivations at work. Journal of Vocational Behavior. 2012; 81(3), 354-363.

**Keywords**

Hospital reforms, institutional, organisational and operational logics, hybrid management

#### O64 Time management in health professionals

##### Conceição Rainho^1^, José C. Ribeiro^2^, Isabel Barroso^1^, Vítor Rodrigues^1^

###### ^1^Universidade de Trás-os-Montes e Alto Douro, Vila Real, 5001-801, Portugal; ^2^Centro Hospitalar de Trás-os-Montes e Alto Douro, Vila Real, 5000-508, Portugal

####### **Correspondence:** Conceição Rainho (crainho@utad.pt) – Universidade de Trás-os-Montes e Alto Douro, Vila Real, 5001-801, Portugal

**Background**

Time management affects the productivity and health of workers. At health organizations professionals are exposed to heavy workload and time pressure, labour demand indicators, in a highly competitive and demanding health sector. Therefore, the application of time management techniques is presented as an essential tool, with implications for productivity, well-being of workers and quality of health care. Objective: analyse the dimensions of the Time Management Behaviour (TMB) scale in healthcare professionals.

**Methods**

A descriptive cross-sectional study, the TMB scale was used to evaluate time management dimensions, consisting of 25 items answered on a range varying between 1 and 5, where higher scores correspond to higher levels.

**Results**

Of the 703 health professionals studied, 76.0 % were female with an average of 41 years old. The dimensions of time management showed values close to scale midpoint, with preference for organization presenting the highest average (3.83) and mechanics of time management dimension the lowest average (2.85).

**Conclusions**

The results showed that the time management dimensions: setting goals and priorities, mechanics of time management and preference for organization, have lower average values compared to other studies and other professions, signalling the management of human resources for health organization studied has relevant information to contribute to the implementation of strategies aimed at the effectiveness of time management.

**Keywords**

Time management, Health professionals

#### O65 Financial rewards and wellbeing in primary health care

##### Carmo Neves, Teresa C. Oliveira

###### Faculdade de Economia, Universidade de Coimbra, 3004-512 Coimbra Portugal

####### **Correspondence:** Carmo Neves (carmonevespt@gmail.com) – Faculdade de Economia, Universidade de Coimbra, 3004-512 Coimbra Portugal

**Background**

Contractualisation in the private sector may include performance-related pay [1]. But this was not typical of Human Resource Management (HRM) in the National Health Service (NHS) until the New Public Management (NPM)-style reforms [2]. This paper draws on the perceptions of clinical professionals of financial reward systems through contractualisation in Primary Care to explore the links between clarity of job roles [3], workloads and motivation [4] and their impact on the climate and quality of care [5] and employee wellbeing.

**Methods**

Using a case study approach within grounded theory, content analysis was carried out of six fully transcribed audio-taped semi-structured interviews with 2 doctors, 2 nurses and 2 administrators in two Unit Care Services in north of Portugal which were recently subject to NPM-style reforms.

**Results**

Although health professionals may welcome increases in pay, they are not motivated primarily by financial rewards, but rather are committed to health as a public service. When contractualisation is only partial for some rather than all employees, its outcome may be negative. Reward systems based on quantitative performance criteria may compromise the climate and quality of care through lack of the clarity of job roles and employee wellbeing.

**Conclusions**

Motivating health professionals needs a more holistic HRM approach than performance-related pay and involvement of clinical managers in its design and operational implementation.

**References**

1. Mehmood S, Ramzan M, Akbar MT. Managing Performance through Reward System. Journal of Education & Research for Sustainable Development. 2013; 1(1): 1-8.

2. Oliveira TC, Fontes da Costa J, Lira de Carvalho FE. Hierarchies or Holdings? In C. Machado and J. P. Davim, (Eds). 2014. Effective Human Resource Management in Small and Medium Enterprises: Global Perspectives. IGI Global Associates, 342-376.

3. Woodrow C, Guest DE. Public violence, staff harassment and the wellbeing of nursing staff: an analysis of national survey data. Health Services Management Research. 2012; 25(1): 24-30.

4. Güngör P. The Relationship between Reward Management System and Employee Performance with the Mediating Role of Motivation: A Quantitative Study on Global Banks. The Proceedings of 7th International Strategic Management Conference. Procedia - Social and Behavioral Sciences. 2011; 24: 1510-1520.

5. Moran CM, Diefendorff JM, Kim TY, Liu ZQ. A profile approach to self-determination theory motivations at work. Journal of Vocational Behavior. 2012; 81(3): 354-363.

**Keywords**

Contractualisation, Performance related pay, HRM, Motivation, Reward systems, Work roles, Wellbeing, Primary health care

#### O66 Patient safety promotion in the operating room

##### Bárbara Oliveira^1^, Mª Carminda Morais^2,3^, Pilar Baylina^4^

###### ^1^Centro Hospitalar do Porto, 4099-001 Porto, Portugal; ^2^Escola Superior de Saúde, Instituto Politécnico de Viana do Castelo, 4900-347 Viana do Castelo, Portugal; ^3^Centro de Estudos e Investigação em Saúde, Universidade de Coimbra, 3004-512 Coimbra, Portugal; ^4^Escola Superior de Tecnologia da Saúde do Porto, Instituto Politécnico do Porto, Vila Nova de Gaia, 4400-330, Portugal

####### **Correspondence:** Bárbara Oliveira (barbaroliveira@gmail.com) – Centro Hospitalar do Porto, 4099-001 Porto, Portugal

Over recent years, patient safety has gained more importance in the field of public health. Surgical safety is particularly relevant due to the great number of perioperative complications which could be avoided and can cause damage to the patient. This also implies major costs to the healthcare organization, causing a great impact on its budget. Through the analysis of a safety culture, measuring patient safety in a healthcare institution is possible since they are influenced by each other. Hence, the “Hospital Survey on Patient Safety Culture” (HSPSC) arises due to the need for evaluation of patient safety in healthcare.

The aim of this work is to evaluate and promote patient safety in the Operating Rooms (OR) of the Centro Hospitalar do Porto (CHP).

This descriptive study was conducted in the OR of CHP, where the Portuguese version of the HSPSC was applied to the workers, namely doctors, nurses, technicians, health care assistants and secretaries.

The results obtained show that in CHP teamwork within units has great value, as this dimension has the highest percentage of positive answers (63.4 %). The lowest percentage of positive answers is the dimension of frequency of events reported (24.9 %). The workers’ perception of the degree of patient safety is divided between “very good” (47.5 %) and “acceptable” (39.7 %). The strengths in CHP are teamwork within units, efforts for the improvement of patient safety, manager expectations and actions promoting patient safety. The areas requiring intervention in CHP are those related to the system of events reported, staffing and work between units.

**Keywords**

Safety Culture, Healthcare Quality, Patient Safety

#### O67 Difficulties and needs of pre-graduate nursing students in the area of Geriatrics/Gerontology

##### Rogério Rodrigues^1^, Zaida Azeredo^2^, Corália Vicente^3^

###### ^1^Escola Superior de Enfermagem de Coimbra, Coimbra, 3046-851, Portugal; ^2^Research in Education and Community Intervention, Escola Superior de Saúde Jean Piaget, 4405-678 Gulpilhares, Vila Nova de Gaia, Portugal; ^3^Instituto de Ciências Biomédicas Abel Salazar, Universidade do Porto, 4050-313 Porto, Portugal

####### **Correspondence:** Rogério Rodrigues (rogerio@esenfc.pt) – Escola Superior de Enfermagem de Coimbra, Coimbra, 3046-851, Portugal

**Background**

In an era of global ageing, the increasing proportion of older people has a direct impact on nursing practice, and also on nursing education, presenting unprecedented opportunities and challenges. Objective: To study difficulties and needs of pre-graduate nursing students in the area of Geriatrics/Gerontology.

**Methods**

A quantitative, descriptive study, with a non-probabilistic accidental sample, constituted by 238 undergraduate nursing students of the 4th year of the Nursing School of Coimbra. Application of self-administered questionnaires related to knowledge on ageing issues, difficulties in caring for the elderly person in community and hospital settings, after authorization of the institution and informed consent of students.

**Results**

The study emphasizes the difficulties perceived by students and consequent training needs combined with the reality of the various clinical teaching contexts; notable is the nonexistence of a vision of care that integrates the multidimensional systematic assessment of the elderly, to support and sustain conditions for quality of life of the elderly in community, family and social support.

**Conclusions**

This study presents relevant data about the difficulties experienced by nursing students of the 4th year and their training needs, regarding the care of the elderly, justifying the adequacy of the curricula of the 1st cycle of studies in nursing, specifically the integration in the curricula of the methodology of multidimensional functional assessment of elderly. Their use in graduate nursing training will be an asset for students to have an integrated vision of the elderly in the community.

**Keywords**

Nursing students, Geriatrics, Gerontology

#### O68 Teaching and learning sexuality in nursing education

##### Hélia Dias^1^, Margarida Sim-Sim^2^

###### ^1^Escola Superior de Saúde, Instituto Politécnico de Santarém, Santarém, 2005-075, Portugal; ^2^Escola Superior de Enfermagem S. João de Deus, Universidade de Évora, 7000-811 Évora, Portugal

####### **Correspondence:** Hélia Dias (helia.dias@essaude.ipsantarem.pt) – Escola Superior de Saúde, Instituto Politécnico de Santarém, Santarém, 2005-075, Portugal

Sexuality is recognized as part of holistic nursing care, but its inclusion in clinical practice and nursing training is inconsistent. Based on the question "How students and teachers acknowledge sexuality in teaching and learning?", we developed a study in order to characterize the process of teaching and learning sexuality in a micro perspective of curriculum development.

We used a mixed methods design with a sequential strategy: QUAN → qual of descriptive and explanatory type. 646 students and teachers participated. The quantitative component used questionnaire surveys. Document analysis was used in the additional component.

A curricular dimension of sexuality emerges guided by a behaviourist line and based on a biological vision. The issues considered safe are highlighted and framed in steps of adolescence and adulthood and more attached to female sexuality and the procreative aspect. There is in emergence a hidden curriculum by reference to content from other dimensions of sexuality but less often expressed. Theoretical learning follows a communicational model of reality through abstraction strategies, which infers a deductive method of learning, with a behaviourist approach to assessment. Clinical teaching addresses sexuality in combination with reproductive health nursing. The influencing factors of teaching and learning of sexuality were also explored.

We conclude that the vision of female sexuality taught and learned in relation to women has a projection of care in clinical practice based on the same principles.

**Keywords**

Sexuality, nursing education, teaching and learning process, curricular development, students, teachers

#### O69 Entrepreneurial Motivations Questionnaire: AFC and CFA in academy

##### Pedro Parreira^1^, Anabela Salgueiro-Oliveira^1^, Amélia Castilho^1^, Rosa Melo^1^, João Graveto^1^, José Gomes^1^, Marina Vaquinhas^1^, Carla Carvalho^2^, Lisete Mónico^2^, Nuno Brito^3^

###### ^1^Escola Superior de Enfermagem de Coimbra, 3046-851 Coimbra, Portugal; ^2^Faculdade de Psicologia e Ciências da Educação, Universidade de Coimbra, 3001-802 Coimbra, Portugal; ^3^Instituto Politécnico de Viana do Castelo, 4900-347 Viana do Castelo, Portugal

####### **Correspondence:** Pedro Parreira (parreira@esenfc.pt) – Escola Superior de Enfermagem de Coimbra, 3046-851 Coimbra, Portugal

**Background**

Entrepreneurship has been considered essential to create value, being a powerful driver of economic and social development. Academy must invest in the development of entrepreneurial skills in students (“Entrepreneurial Academy”). Objective: To analyse the psychometric proprieties of the EMQ-Entrepreneurial Motivations Questionnaire.

**Methods**

Sample: 6,532 students from 17 polytechnic Institutes of Portugal. Measure: EMQ, composed by measures of Entrepreneurial motives for the business, Entrepreneurial influences, and Support Services.

**Results**

Exploratory factor analysis (EFA) for Entrepreneurial-Motives-for-the-business (KMO = .921, X^2^ = 54387.94, p < .001) extracted 4 factors, explaining 53.81 % of variance (EV): F1-Family security (19.92 % EV; M = 4.07, SD = 0.67), F2-Prestige (14.60 % EV; M = 3.25, SD = 0.75), F3-Independence (13.13 % EV; M = 3.33, SD = 0.75), and F4-Realization of an opportunity (11.15 % EV; M = 4.08, SD = 0.56). This factorial structure was supported by Confirmatory factor analysis (CFA), with an acceptable fit: RMSEA = .084. EFA of Entrepreneurial-Influences (KMO = .916, X^2^ = 60584.93, p < .001), extracted 4 factors, EV 60.08 %: F1-Resources availability (21.25 % EV; M = 3.86, SD = 0.68), F2-Stable customers and incentives (19.70 % EV; M = 3.82, SD = 0.65), F3-Social and economic instability (11.11 % EV; M = 2.96, SD = 1.06), and F4-Opportunities in the sector and residence area (8.03 % EV; M = 3.29, SD = 0.95). This factorial structure was supported by CFA, with an acceptable fit: RMSEA = .073. EFA of Support-Services (KMO = .995, X^2^ = 57311.43, p < .001) extracted 2 factors, EV 58.43 %: F1-Financial support (32.59 % EV; M = 3.93, SD = 0.69) and F2-Prestige (25.84 % EV; M = 3.95, SD = 0.65). This factorial structure was supported by CFA, with an acceptable fit: RMSEA = .090. The health group showed scores mostly above global average in factors.

**Conclusions**

EMQ showed adequate psychometric properties. The instrument is useful for measuring entrepreneurships motivations in health.

**Keywords**

Entrepreneurship, Entrepreneurial Motivations, Entrepreneurial Motivations Questionnaire, Entrepreneurial Academy

#### O70 Nursing intervention to patient with Permanent Pacemakers and Implantable Cardioverter Defibrillators: a qualitative analysis

##### Cassilda Sarroeira^1,2,3^, José Amendoeira^1,2,3,4^, Fátima Cunha^1,2,3^, Anabela Cândido^1,2,3^, Patrícia Fernandes^5^, Helena R. Silva^5^, Elsa Silva^5^

###### ^1^Escola Superior de Saúde, Instituto Politécnico de Santarém, 2005-075 Santarém, Portugal; ^2^Unidade de Investigação do Instituto Politécnico de Santarém, 2001-904 Santarém, Portugal; ^3^Unidade de Monitorização de Indicadores em Saúde, Instituto Politécnico de Santarém, 2005-075 Santarém, Portugal; ^4^Life Quality Research Centre, Instituto Politécnico de Santarém, 2001–904 Santarém, Portugal; ^5^Hospital Prof. Doutor Fernando Fonseca E.P.E., Amadora, 2720-276, Portugal

####### **Correspondence:** Cassilda Sarroeira (cassilda.sarroeira@essaude.ipsantarem.pt) – Escola Superior de Saúde, Instituto Politécnico de Santarém, 2005-075 Santarém, Portugal

**Background**

The study stems from a project of the Health Indicator Monitoring Unit (HIMU) of the Health School of Santarém (ESSS), developed in partnership with the Cardiology Service of Nurses of an Urban Hospital in nursing consultation (NC) with patients with Permanent Pacemakers (PPMs) and Implantable Cardioverter Defibrillators (ICDs). Nurses, as members of the multidisciplinary team, meet the needs of the patient with PPMs and ICDs, promoting the ability for self-care, whether in developing strategies to prevent health deviations or integrating new self-care behaviour. It was imperative to know how nurses develop the NC to carry out an assessment of the effectiveness of the results in these patients. Objective: To characterize and describe the nurses’ interventions in NC with patients with PPMs and ICD.

**Methods**

Descriptive, qualitative and cross-sectional, exploratory design, developed with nurses (NC) with patients with PPMs and ICDs. Data from nurses’ narratives of action and recording instruments were analysed with Content Analysis.

**Results**

Three dimensions were identified: Initial Assessment, Nurse Intervention and Intervention Evaluation (outcomes). The categories: Initial assessment - Function; Reason for the action; Intervention action of Nurse - Observe, Manage, Attending; Inform. The result of the intervention evaluation - structural and functional integrity of the person and self-care action.

**Conclusions**

The nursing intervention in the NC shows an orientation to patient-centred-care on their physical dimension and capacity for self-care.

Identified different ways of approach, but the concern for the well-being, knowledge and training of the person, are cross-cutting elements for nurses.

**Keywords**

Nursing intervention, nursing consultation, patient, permanent-pacemaker/ICD, self-care, qualitative analysis

#### O71 Alcohol consumption among nursing students: where does education fail?

##### Isabel Barroso, Leila Lapa, Cristina Antunes

###### Universidade de Trás-os-Montes e Alto Douro, Vila Real, 5001-801, Portugal

####### **Correspondence:** Isabel Barroso (imbarroso@utad.pt) – Universidade de Trás-os-Montes e Alto Douro, Vila Real, 5001-801, Portugal

**Background**

The drinking behaviour of university students has become a cause of concern due to the occurrence of excessive consumption situations during academic festivities, when, hypothetically, nursing students should have better health behaviours than their peers. Objectives: (I) to describe the drinking behaviour of nursing students; (II) to determine the prevalence of regular alcohol consumption among this population; and (III) to relate the regular alcohol consumption to gender and grade year.

**Methods**

Data was collected through self-reported questionnaires. This was a descriptive and correlational study.

**Results**

The participants were 226 students, of whom 219 were female and 40 were male. Alcohol consumption was high; 68.9 % of the students reported usually drinking alcoholic beverages. The majority (96.9 %) already consumed alcohol at some time in their lives, and reported consuming mostly in the company of friends (99.1 %). The majority, 67.3 %, had already been drunk at least once. Also, 27.2 % of the students had consumed more than three alcoholic drinks on a single occasion. Among the students who reported having been drunk at some time, 2.2 % reported having had unprotected sexual intercourse when drunk.

**Conclusions**

The results are discussed within the question that these students, although knowledgeable of the harmful effects that the consumption of alcoholic beverages can cause, have a high prevalence of alcohol consumption. Some dangerous behaviours occur along with alcohol abuse, such as driving under the influence of alcohol, or having unprotected sexual intercourse. Implications for the planning of community nursing interventions within health education are discussed.

**Keywords**

Consumption of alcohol, nursing students, nursing interventions

#### O72 Labour stress in nursing

##### Ana Gonçalves^1^, Ana Galvão^1^, Mª José Gomes^1^, Susana R. Escanciano^2^

###### ^1^Instituto Politécnico de Bragança, Bragança, 5300-121 Bragança, Portugal; ^2^Universidad de León, 24004 León, España

####### **Correspondence:** Ana Gonçalves (velosogoncalves@gmail.com) – Instituto Politécnico de Bragança, Bragança, 5300-121 Bragança, Portugal

**Background**

Based on evidence found during the empirical study we can affirm that the nursing profession is affected by work stress. Objective: Evaluate stress and engagement levels among nurses in health units in Portugal and Spain and describe the stress-generating factors among the surveyed nurses.

**Methods**

A comparative study on a transversal level. Sample of 867 nurses (504 Portuguese, 363 Spanish), female 83.6 % (78.6 % in Portugal, 90.6 % in Spain) and average age of 37. 77.2 % of the Portuguese and 39.4 % of the Spanish nurses work on average 40 hours per week. 60.6 % and 57.7 % of the Spanish and Portuguese respectively have exercised their profession for 10 years. Pamela Gray-Toft’s Nursing Stress Scale (1981) [1] and Schaufeli & Bakker’s Utrecht Work Engagement Scale (2003) [2] were used.

**Results**

Globally, Portuguese nurses experience higher stress levels although the difference with Spanish nurses is not statistically significant. There are statistically significant differences between Portugal and Spain in “Lack of help from colleagues” and also in the psychological domain in general. Concerning Engagement, there are statistically significant differences in the three dimensions, the p-value of the Student t-test was under 5 %, highlighting that Spanish nurses are more vigorous, dedicated and absorbed by their work.

**Conclusions**

Portuguese nurses perceive more psychological stress and mention having less help from colleagues. Spanish nurses feel more vigorous, dedicated and absorbed by their work.

**References**

1. Pamela GT, James GA. The Nursing Stress Scale: Development of an Instrument. Journal of Behavioral Assessment. 1981; 3(1): 11-23.

2. Schaufeli B, Bakker A. UWES – Utrecht Work Engagement Scale - Preliminary Manual. Utrecht: Occupational Health Psychology Unit - Utrecht University; 2003.

**Keywords**

Engagement, Nurses, Stress

#### O73 The influence of safe staff nursing in patient satisfaction with nursing care

##### Maria Freitas^1^, Pedro Parreira^2^, João Marôco^3^

###### ^1^Instituto de Ciências da Saúde, Universidade Católica Portuguesa, Lisboa, 1649-023, Portugal; ^2^Escola Superior de Enfermagem de Coimbra, 3046-851 Coimbra, Portugal; ^3^Instituto Universitário de Ciências Psicológicas, Sociais e da Vida, 1149-041 Lisboa, Portugal

####### **Correspondence:** Maria Freitas (mjbsfreitas@gmail.com) – Instituto de Ciências da Saúde, Universidade Católica Portuguesa, Lisboa, 1649-023 Lisboa, Portugal

Safe Staff Nursing emerges as condition for quality nursing care. It integrates a quantitative dimension (number of nurses) and a qualitative dimension (mix of nurses’ competencies). It was intended in this research to analyse the relationship between Safe Staff Nursing and patient satisfaction with nursing care.

A quantitative and correlational study was carried out, with a convenience sample of 1,290 guests, 43 head nurses and 629 nurses. We used the Evaluation of Patient Satisfaction with Nursing Care Scale” (EASCCE18), and to identify patterns of nursing services/staff allocation we used the Cluster Analysis Technique.

Two allocation patterns emerged: the more secure allocation pattern (Pattern A - 55.8 % services) the teams are more developed (nurses with higher age, greater professional experience, more specialist nurses), and the number of nurses observed is equal to or greater than that estimated (by formula in the Normative Circular N.1, MH, 2006); less secure allocation pattern (Pattern B - 44.2 % services) characterized by nursing teams with less developed skills profiles and the number of nurses observed is lower than estimated.

The results showed that patients in Pattern A services have higher overall satisfaction levels (Average AP = 4.53) compared to Pattern B (Average BP = 4.39), and the differences are statistically significant for p ≤ 0.05 (t (41) = 2.502; p = 0.016). We concluded that the safe staff nursing is a factor that positively influences patient satisfaction with nursing care.

**Keywords**

Nursing, patient satisfaction, nursing care, nursing human resources

#### O74 Intention to use eHealth strategies with nursing students

##### Ana R. Fernandes, Cremilde Cabral, Samuel Alves, Pedro Sousa

###### School of Health Sciences, Polytechnic institute of Leiria, 2411-901 Leiria, Portugal

####### **Correspondence:** Pedro Sousa (pedro.sousa@ipleiria.pt) – School of Health Sciences, Polytechnic institute of Leiria, 2411-901 Leiria, Portugal

**Background**

Information and Communication Technologies have come to facilitate access and efficiency of healthcare, and promote a closer relationship between health professionals and patients.

Objectives: This study aims to assess the intention to use of eHealth with nursing students, and to evaluate the influence of sociodemographic variables, internet user profile and eHealth literacy on the intention to use eHealth.

**Methods**

This correlational and cross-sectional study was applied in a sample of 67 nursing students from a Portuguese School of Health Sciences (85.1 % female and 14.9 % male), using the “eHealth Literacy Scale” and the “Intention to use eHealth questionnaire”, drawn up according to the “Unified Theory of Acceptance and Use of Technology”. Statistical analysis was performed using SPSS software and non-parametric tests were used to verify the statistical significance of data, considering statistical significance for p < 0.05.

**Results**

Moderate scores (from 2.88 to 3.97 out of 5 points) were found in all dimensions of intention to use eHealth (behavioural intention, performance expectancy, effort expectancy, social influence, facilitating conditions and expectancy of difficulties). Significant associations were found between the intention to use eHealth and several sociodemographic variables (age, gender, education), internet user profile and eHealth Literacy index.

**Conclusions**

The intention to use eHealth by nursing students was found to be only moderate, several predictors being found. These findings offer implications for enhancing the nursing curriculum and for continuing education opportunities. Increasing eHealth literacy has the potential to change the adoption of eHealth and support improved patient outcomes.

**Keywords**

eHealth, nursing, Intention to use

#### O75 Community Based Mental Health: contributions of an interdisciplinary international program for students in higher health education

##### António Ferreira^1^, Fernanda Príncipe^1^, Ulla-Maija Seppänen^2^, Margarida Ferreira^1^, Maribel Carvalhais^1^, Marilene Silva^3^

###### ^1^Escola Superior de Enfermagem da Cruz Vermelha Portuguesa de Oliveira de Azeméis, 3720-126 Oliveira de Azeméis, Portugal; ^2^Oulu University of Applied Sciences, 90250 Oulu, Finlândia; ^3^Unidade de Saúde Familiar - Espaço Saúde, Porto, 4100-503, Portugal

####### **Correspondence:** António Ferreira (ferreira.esecvpoa@gmail.com) – Escola Superior de Enfermagem da Cruz Vermelha Portuguesa de Oliveira de Azeméis, 3720-126 Oliveira de Azeméis, Portugal

**Background**

Current challenges in mental health have led knowledge societies to promote a review and discussion of policies and intervention strategies with a positive impact in promoting the mental health of communities. Community-Based Mental Health (CBMH) is an international joint program between 8 European countries and 9 institutions of higher education, since 2011. Objective: To promote collaborative learning in community-based mental health as well as develop actions and community intervention strategies focused on the client.

**Methods**

The research aimed to evaluate the efficiency of the international program (CBMH) with the use of post-program assessment and collection of data from individual reports and the online questionnaire.

**Results**

The CBMH program was attended by 180 students of undergraduate studies from different disciplines such as nursing (25 %), physiotherapy (20 %), occupational therapy (15 %), social work (15 %), cardiopneumology (12 %), speech therapy (8 %), art (3 %), psychology and other areas (2 %). Students refer as main factors: the "content of keynotes," "diverse and innovative use of teaching-learning methodologies", work in tutorials with multidisciplinary groups "and" thematic workshops".

**Conclusions**

Students who participated in this program state expressively that it had a very positive impact on the understanding of the phenomena inherent to a mental health community-based approach, as well as allowing the appropriation of knowledge and tools that make them more competent in their field and cooperation with other areas. High levels of students’ satisfaction with program, as well as in the development and application of new teaching methods were some of the strong points mentioned.

**Keywords**

Community Based Mental Health, Professional development, interdisciplinary learning, intercultural learning.

#### O76 Study of satisfaction at work of graduates in nursing: 2002-2014

##### Manuela Ferreira, Joana Silva, Jéssica Neves, Diana Costa, Bruno Santos, Soraia Duarte

###### Escola Superior de Enfermagem da Cruz Vermelha Portuguesa, 3720-126 Oliveira de Azeméis, Portugal

####### **Correspondence:** Manuela Ferreira (ferreiramanuela75@gmail.com) – Escola Superior de Enfermagem da Cruz Vermelha Portuguesa, 3720-126 Oliveira de Azeméis, Portugal

**Background**

The increasing number of graduated nurses, the reduction of employment opportunities in the country and the poor working conditions/salary, causes many graduates to begin their nursing profession finding themselves in a less positive context. This context may negatively interfere with the satisfaction of nurses at work and nursing care given. Objective: Characterize the satisfaction at work of graduates in nursing.

**Methods**

This is a descriptive study in a sample of 141 graduates between 2002 and 2014 in a nursing school of the Northern Region of Portugal. The "Satisfaction Scale with the Professional Status of Nurse" was created and validated based on literature review. It is a Likert scale of 1 to 5 points: 1 – very dissatisfied and 5 – very satisfied. The scale has five dimensions: working conditions, salary, interpersonal relations, and personal and professional fulfilment.

**Results**

It was concluded that nurses feel more satisfied at work in the dimension interpersonal relationships (mean = 3.46, SD = 0.60), followed by degree of personal and professional fulfilment (mean = 3.37; SD = 0.65) and the quality of working conditions (mean = 3.13, SD = 0.51). The dimension that had a lower satisfaction at work was remuneration (mean = 2.85, SD = 0.79).

**Conclusions**

Graduate in Nursing show average values of job satisfaction but dissatisfaction with regard to remuneration. This satisfaction situation at work, for people who are starting their professional career, requires a very special look of the profession and society in general. It also requires conducting more research studies to extend this study, and further study of some aspects relating to minor levels of satisfaction presented and the quality of nursing care provided.

**Keywords**

satisfaction at work, graduates in nursing, quality of nursing care

#### O77 Health professionals’ attitudes towards breastfeeding

##### Sílvia Marques^1^, Sónia Ramalho^2^, Isabel Mendes^3^

###### ^1^Administração Regional de Saúde de Lisboa e Vale do Tejo, 1749-096 Lisboa, Portugal; ^2^Health Research Unit & School of Health Sciences, Polytechnic institute of Leiria, 2411-901 Leiria, Portugal; ^3^Escola Superior de Enfermagem de Coimbra, 3046-851 Coimbra, Portugal

####### **Correspondence:** Sílvia Marques (silvimarques@gmail.com) – Administração Regional de Saúde de Lisboa e Vale do Tejo, 1749-096 Lisboa, Portugal

**Background**

The National Breastfeeding Policy and Action Plan 2015-2020 recognises the right that all mothers have for receiving clear and impartial information so as to be able to make a fully informed choice on how to feed their baby (WHO, 2015) [1]. Objectives: To identify the sociodemographic, professional and contextual variables influencing the attitudes of health professionals towards breastfeeding and to assess the relation between attitudes of professionals and some sociodemographic, professional and contextual variables.

**Methods**

A quantitative, correlational and cross-sectional study of a sample of 122 health professionals of Portugal. A questionnaire was applied on sociodemographic, professional and contextual data and “Scale of Health professional attitudes to breastfeeding” [2].

**Results**

Health professionals with an average age of 42.75 years (s = 10.2), female (77.9 %), married or in union (79.5 %), working in a Family Health Unit (64.8 %). The majority (88.5 %) did not attend training courses in this area. They showed a positive attitude towards the breastfeeding (X = 183.77). There are differences between attitudes: the profession (χ2 = 8.863, p = 0.031), the nurse's area of expertise (U = 24.00, p = 0.026), the duration of professional experience (r = -0.231, p = 0.011) and that health professionals attended training in this area influenced more favourable attitudes.

**Conclusions**

This study confirms the need for specific training in counselling on breastfeeding for health professionals and implementing repaired promotion strategies for breastfeeding support.

**References**

1. World Health Organization. National Breastfeeding Policy and Action Plan: 2015-2020. Malta: Health Promotion and Disease Prevention Directorate; 2015.

2. Marinho C. Os Profissionais de Saúde e o Aleitamento Materno: Um estudo exploratório sobre as atitudes de médicos e enfermeiros [Dissertação]. Lisboa: Instituto Superior de Psicologia Aplicada; 2003.

**Keywords**

Attitudes, health professionals, breastfeeding

#### O78 Continuity of nursing care to person with type 2 diabetes

##### Clarisse Louro^1^, Eva Menino^1^, Maria Dixe^1^, Sara S. Dias^1,2^

###### ^1^Health Research Unit & School of Health Sciences, Polytechnic institute of Leiria, 2411-901 Leiria, Portugal; ^2^Departamento de Saúde Pública & Centro de Estudos de Doenças Crónicas, Faculdade de Ciências Médicas, Universidade Nova de Lisboa, 1169-056 Lisboa, Portugal

####### **Correspondence:** Eva Menino (eva.guilherme@ipleiria.pt) – Health Research Unit & School of Health Sciences, Polytechnic institute of Leiria, 2411-901 Leiria, Portugal

**Background**

Nursing has revealed to provide continuity of care and the necessary structure for the management of chronic disease [1]. Continuity of care in the area diabetes assumes particular importance, once self-care is a cornerstone of an effective treatment and requires a continuous support and supervision by nurses [2]. Objective: To assess the frequency of nursing interventions directed to continuity of care in education to people with diabetes in primary health care.

**Methods**

Cross-sectional study with 104 nurses, in primary health care, using Diabetes Education Process Scale (DEP) [3]. The results were obtained using descriptive statistics. All inherent ethical principles were respected in this study.

**Results**

The intentional sample, included 104 nurses who agreed to participate in the study, and currently provide nursing care to clients with type 2 diabetes in primary health settings. The sample has an average age of 43 years (S.D. = 7.5), with maximum age of 62 years and a minimum of 29 years, the majority (95.9 %) were female. Respondents, on average, develop such interventions in a high frequency, with a mean value of 3.80 (SD = 0.79), (4 corresponds to the maximum frequency and 1 minimum).

**Conclusions**

These nurses, on average, considering the frequency of interventions, have developed therapeutic education respecting the continuity of care. These interventions have major importance in self-care promotion for the proper control of diabetes.

**References**

1. Watts SA, Gee J, O’Day ME, Schaub K, Lawrence R, Aron D, Kirsh S. Nurse practitioner‐led multidisciplinary teams to improve chronic illness care: the unique strengths of nurse practitioners applied to shared medical appointments/group visits. Journal of the American Academy of Nurse Practitioners, 2009: 167-172.

2. Haas L. et al. National standards for diabetes self-management education and support. Diabetes care, 2013, 36.Supplement 1: S100-S108.

3. Menino EG, Dixe MA, Martins MC. Construction and Validation of “Diabetes Education Process (DEP)” Scale. International Journal of Health and Life-Sciences. 2015: 01-12.

**Keywords**

Nursing intervention, community health care, Diabetes

#### O79 Stigma toward mental illness among future health professionals

##### Marina Cordeiro^1^, Catarina Tomás^1^, Ana Querido^1^, Daniel Carvalho^1,2^, João Gomes^1,2^

###### ^1^School of Health Sciences, Polytechnic institute of Leiria, 2411-901 Leiria, Portugal; ^2^Hospital de Santo André, Centro Hospitalar de Leiria, 2410-197 Leiria, Portugal

####### **Correspondence:** Marina Cordeiro (marina.cordeiro@ipleiria.pt) – School of Health Sciences, Polytechnic institute of Leiria, 2411-901 Leiria, Portugal

**Background**

Mental health stigma is still a major problem of our society and represents a big source of anguish for patients. This type of stigma is a relatively common phenomenon among health professionals, and represents a big obstacle to provide qualitative health care. Objectives: To acknowledge mental health stigma level among health students, and to know its correlation with the prevalence of diagnosed mental disorder and contact with a mentally ill relative, in this sample.

**Methods**

A cross-sectional study, descriptive and correlational, was performed. The sample, selected by a non-probabilistic of convenience sampling method, is composed by 672 higher health education students of the Leiria’s district. Data was collected by a self-administered questionnaire, composed with sociodemographic questions and the Attribution Questionnaire.

**Results**

Stigma levels in the sample tested were low to moderate. 33 students have a mental disorder diagnose, most of them depression disorder (n = 23), and 224 students have contact with a mentally ill relative. There are no statistically significant differences in stigma level between students that have a mental disorder diagnose or have contact with a mentally ill relative and those not.

**Conclusions**

Low to moderate stigma level in health students is a similar finding when comparing to other studies in this area. Students who have mental disorder diagnose or contact with a mentally ill relative present the same stigma level when compared to those who haven’t. This result shows that pedagogic intervention aiming to reduce this stigma should be a priority in the higher health education to every student.

**Keywords**

Stigma, mental health, mental disorders

#### O80 Working with fears and anxieties of medical students in search of a humanized care

##### Frederico C. Valim, Joyce O. Costa, Lúcia G. Bernardes

###### Faculdade de Medicina, Universidade José do Rosario Vellano, Divinópolis – Minas Gerais, 35502-634, Brasil

####### **Correspondence:** Frederico C. Valim (fred.valim@gmail.com) – Faculdade de Medicina, Universidade José do Rosario Vellano, Divinópolis – Minas Gerais, 35502-634, Brasil

**Background**

The globalization process has brought with it anxiety and anguish. Greater access and interactivity and increased information exchange replaces the core of the individual’s exposure to fear and discomfort. The new education curriculum policy is in favour of more effective and humane doctor-patient relationships. Humanization can be stimulated with academic approaches. In psycho-pedagogical support services for medical students (PPSS), one of the purposes is to help scholars to manage stress and frustrations and deal with social issues. Objective: To analyse the processes that occurred in PPSS and their possible contributions to the humanization of medical students in the light of a reflexive analysis of the literature.

**Methods**

Qualitative research of interviews, experiments and expressions of medical students that were developed in PPSS activities and discussions, were recorded in a systematic way. A literature review about concepts and processes of humanization guided the analysis.

**Results**

The development of a humanistic individual is a complex process, consisting of experiments, individual psychological factors and social contexts that influence the construction of the "humanized" person. It was observed that the events and challenges have become a rich space of experiences. Experiences, responsibilities, anxieties and successes have been developed between students in context, in the individual or collective mind.

**Conclusions**

Medical education involves a process itself, generating specific fears and anxieties. The sharing of solutions developed by PPSS and students to reduce stress of this stage of life was considered effective by themselves, reinforcing the importance of having a working PPSS approach.

**Keywords**

Humanized care, doctor patient, relationship medical student

#### P16 Surgical paediatrics patients’ psycho prophylaxis at a teaching hospital

##### Helena Prebianchi

###### Pontifícia Universidade Católica de Campinas, Campinas, São Paulo, 13086-900, Brasil

**Background**

Surgical Psychoprophylaxis helps the patient to deal with and to relieve symptoms and other specific problems created by surgical intervention situations. Goal: to get to know the means of hospital psychologists acting with surgical paediatric patients in teaching hospitals, during the preoperative period, as well as the surgery doctors’ perceptions about the surgical psychoprophylaxis.

**Methods**

A qualitative research, with the participations of six psychologists and six surgery doctors who work in teaching hospitals in Brazil. The testimonials content was analysed through three synergic steps: description, inductive analysis and critical analysis, and after that, the convergence points were analysed.

**Results**

Both the description and the inductive analysis indicate that the psychologists’ activities refer to post-surgical appointments requested by the doctors and nurses for the children operated on. Critical analysis stressed that the clarity each professional has about his or her own functions and their ability to communicate with colleagues are determinant for the appropriation of multidisciplinary action. Convergence analysis revealed that psychologists and doctors have similar conceptions about the need of the therapeutics interventions to involve the parents; on the other hand, they differ in the granting of legitimacy to the specialized discourse on surgical prophylaxis for paediatric patients.

**Conclusions**

It is necessary to increase the doctors’ comprehension about how surgical psychoprophylaxis actions at the above mentioned hospital can take into account the needs and wishes of the population served and satisfy the professionals involved.

**Keywords**

Psycho prophylaxis, children, surgery

#### P17 Patient-perceived outcomes in physiotherapy – a pilot study

##### Marlene Cristina Rosa (marlenerosa@ua.pt)

###### Secção Autónoma das Ciências da Saúde, Universidade de Aveiro, Aveiro, 3810-193 Aveiro, Portugal

**Background**

Patient-perceptions are related to several important health outcomes, being determinant to treatment success and a consequent contribution to physiotherapy evidence. However, this information has not been provided by most physiotherapy studies. Objective: To describe patient-perceived outcomes in physiotherapy.

**Methods**

Patients doing ambulatory physiotherapy in private and public health services were included. Sociodemographic data (age, gender) was collected. At the end of the treatment a questionnaire with 10 topics for physiotherapy outcomes was administered: 3 topics for general function (daily activities independence; general mobility; fatigue and tiredness); 2 topics for pain relief (pain intensity; antialgic medication); 5 topics for psychosocial factors (sleep pattern; self-esteem and confidence; participation in recreational activities; interaction with friends and family members; participation in physical activities). Patients were asked to select maximum of 2 topics that have improved. The frequencies of selected topics (%) were reported, i.e. general function (GF), pain relief (PR), psychosocial factors (PS) and the different combinations between them.

**Results**

Seventy patients (53.63 years ± 16.69; 41 female) were included. Sixty-three patients (90.0 %) referred to general function improvement, 48 (68.6 %) referred to pain relief; 15 (21.4 %) referred to positive psychosocial impact. Moreover, patients reported simultaneous improvement in: P and GF (N = 39; 55.71 %), GF and PS (N = 10; 14.28 %) and P and PS (N = 5; 7.14 %).

**Conclusions**

At discharge from physiotherapy, improvement in GF is frequently perceived by patients and most of the time it is combined with perceived pain relief. Patients’ perceptions of psychosocial improvement corroborate the positive impact of physiotherapy in a wide range of life domains.

**Keywords**

Patient-perceptions, physiotherapy, outcomes

#### P18 Building competencies for managers in nursing

##### Narcisa Gonçalves^1,2^, Maria M. Martins^1,2^, Paulina Kurcgant^3^

###### ^1^Instituto de Ciências Biomédicas Abel Salazar, Universidade do Porto, 4050-313 Porto, Portugal; ^2^Escola Superior de Enfermagem do Porto, 4200-072 Porto, Portugal; ^3^Universidade de São Paulo, 05403-000, São Paulo, Brasil

####### **Correspondence:** Narcisa Gonçalves (mnarcisa@esenf.pt) – Instituto de Ciências Biomédicas Abel Salazar, Universidade do Porto, 4050-313 Porto, Portugal

**Background**

The changing profile of nursing professionals have been configured historically as a function of a dynamic labour market in health, which in turn is influenced in the economic, social and cultural context. The growing competitiveness of organizations requires a search for health professionals who are more and more qualified and able professionals. The need to increase assistance, management, research and teaching skills, is gradually being incorporated in the professional competencies. Objectives: To analyse competencies expressed by nursing managers and to identify areas of learning need in management.

**Methods**

A descriptive study of qualitative approach, with 20 interviews with nurse managers analysed. We followed the methodological orientations recognized by Bardin (2009) [1].

**Results**

From the analysis of the interviews, the following main categories emerged: professional practices; management of human resources; skills development in nursing; material and equipment resources; training and development of nursing practice and the improvement of quality. The findings lend support to the body of knowledge that recognizes the wide range of financial, operational, clinical, and human resource responsibilities of the nursing manager.

**Conclusions**

Today it is demanded that the manager in nursing should have a combination of knowledge, attitudes and behaviours that are fluid in when applied to different types of organizations. We need to construct a plan of professional development for nursing managers, which is adjusted in competencies and takes technical, ethical-politics, communicative and the development of citizenship dimensions into consideration.

**References**

1. Bardin L. Análise de Conteúdo. Lisboa: Edições 70; 2009.

**Keywords**

Management in nursing, competencies, training and development

#### P19 Theoretical basis underlying physiotherapy practice in stroke rehabilitation

##### André Vieira (andrevieira@ipcb.pt)

###### Escola Superior de Saúde Dr. Lopes Dias, Castelo Branco, 6000-767, Portugal

**Background**

The fundaments used by Portuguese physiotherapists that support the interventions in upper limb rehabilitation are not known. Joining clinical practice with clinical evidence is a current challenge. The goal of this study is to find the state of the art of physiotherapists’ beliefs in rehabilitation of upper limbs in stroke.

**Methods**

A national web-based survey focused in characterizing the professional profile of physiotherapists from Portugal working in upper limb rehabilitation of patients with stroke was conducted in 237 health institutions (n = 462 physiotherapists). The total time of recruitment was from August to December 2014.

**Results**

A total of 179 physiotherapists from 64 different locations in mainland Portugal answered the survey, giving an approximate rate of response of 38.7 %. For this study, 147 responses were eligible for statistical analysis. The average age of respondents was 30.95 (±7.02) years old and had on average 6.91 (±6.54) years of experience. For this sample, the first objective for rehabilitation in acute and chronic stroke was to normalize tonus. In 13 questions about scientific fundaments in rehabilitation of upper limbs post-stroke, the majority of responses in 4 were in disagreement with evidence. Only 3 questions reached a consensus (more than 90 % agreement).

**Conclusions**

The fundaments for rehabilitation are the mirror of clinical practice in physiotherapy. It is hard to find agreement among these professionals, and some have contradictory elements according to scientific evidence. It seems to be necessary to alert professionals and future professionals to interventions with supported scientific results.

**Keywords**

Upper Limb Rehabilitation, stroke, theoretical basis, physiotherapy

#### P20 When the life-cycle ends: the nurse’s confrontation with death

##### Sandrina Bento, Sérgio Deodato, Isabel Rabiais

###### Universidade Católica Portuguesa, Lisboa, 1649-023, Portugal

####### **Correspondence:** Isabel Rabiais (raby@ics.lisboa.ucp.pt) – Universidade Católica Portuguesa, Lisboa, 1649-023, Portugal

**Background**

With the shift of death and dying from home to hospital, in the hospital death process, nurses are inevitably faced with death in their professional context. Accompanying someone at the time of death can be a privilege and an opportunity to give meaning to life, but it is also a time of great exhaustion and emotional overload. It involves having the ability to deal with the suffering of people and one’s own emotions, which is not easy at all and for which the nurse was neither professionally nor naturally prepared. Based on this, the question is how the nurse can develop this emotional competence and mobilize it in the context of care, ensuring quality care and a dignified death for patients at the end of life? Objective: To identify and better understand the strategies developed by nurses to deal with the emotional impact of death and dying.

**Methods**

An integrative literature review with research in the Institutional Repository database of the Portuguese Catholic University and the Portuguese Open Access Scientific Repository.

**Results**

Rationalization, the situation of denial, forging ahead, avoidance, false security, projective identification, patient relationship severance, focus on routines and technique, team sharing, outside-work activities and family support strategies were identified during this study.

**Conclusions**

The intense emotional experience of end-of-life care can generate a disproportionate workload and stress with negative consequences, so it is important that nurses develop strategies that help them deal with these experiences and keep their emotional and spiritual health.

**Keywords**

Nurse, end of life, care, emotions

#### P21 Nursing students’ opinion about the supervision relationship during their first clinical experience

##### Laura Reis (laurareis@esenf.pt)

###### Escola Superior de Enfermagem do Porto, 4200-072 Porto, Portugal

**Background**

The first contact with a clinical reality always has a strong impact on students, in so far as it is from this moment that practices developed begin to gain significance. In the context of the relationship that is established between the clinical supervisor and the student it is essential that pedagogical interaction be vested, which is based on a relationship of help in an atmosphere of trust and openness, thus compelling their personal and professional growth. Objective: To identify the skills that Nursing Students most value related to the supervisor-student relationship.

**Methods**

We chose an ethnographic study within the framework of the qualitative paradigm, in a longitudinal approach according to the logic of the case study. As a data collection technique, we made use of the participants’ observations and semi-structured interviews. The study was carried out by a 2^nd^-year class of a Nursing Course in a Portuguese Nursing Faculty that was undergoing its first clinical experience in a hospital context: 10 weeks in internal medicine and 10 weeks in general surgery.

**Results**

According to the students’ perspective, the supervisory rapport established diverged in relation to the clinical context. It is considered that in the context of medicine, contrary to the context of surgery, the supervisory rapport favoured a relationship of help, listening, trust, respect, commitment, accessibility and development of different areas of know-how.

**Conclusions**

It was ascertained that the students fundamentally valued the clinical supervisors who had relational and socio-effective skills, and who were able to provide development of positive affect.

**Keywords**

Nursing Student, clinical education, supervision process

#### P22 Nursing Relational Laboratory: Pedagogical, dialogic and critical project

##### Ana Torres^1^, Sérgio Soares^1^, Margarida Ferreira^1^, Pedro Graça^2^

###### ^1^Escola Superior de Enfermagem da Cruz Vermelha Portuguesa de Oliveira de Azeméis, 3720-126 Oliveira de Azeméis, Portugal; ^2^Academia de Teatro, 4465-095 S. Mamede de Infesta, Portugal

####### **Correspondence:** Sérgio Soares (sergiosoares@ua.pt) – Escola Superior de Enfermagem da Cruz Vermelha Portuguesa de Oliveira de Azeméis, 3720-126 Oliveira de Azeméis, Portugal

The development of relational skills is a complex and demanding process. For the introduction of new teaching and learning strategies in the Nursing Degree Course, the Nursing Relational Laboratory was developed with the support of the Calouste Gulbenkian Foundation: this is a pedagogical, dialogic and critical project, using sign language, drama and analysis of emotions to develop relational skills. It aims to encourage the development of relational skills of students through active pedagogical action and interactional communication.

Methodology of action research in which the actors are 42 students in the 1st year. Autoscopy sessions to perceive relational skills. Use of a control group. Descriptive analysis and content of autoscopy and instruments: Interpersonal Relations Inventory; Emotional thermometers, Patient Health Questionnaire, Assertiveness Questionnaire and Self-Efficacy Perceived Scale.

Students signed a "Letter of Commitment" to defend the ethical principles of research. The first autoscopy was analysed under the light of a defined grid. There are differences between autoscopy of students in the control group and those who started the first year of the course. They also effectively implemented a framework of conceptual activities and, looked for other similar experiences with a visit to Manchester Metropolitan University.

For Abreu (2001), one’s skills are not inborn nor static, but have a strong personal touch and they progress through the course of experiences that occur in the formative period, in comparison with others and with himself, with the expectation that students will develop technical and scientific skills and relational skills.

**Keywords**

Relation and communication skills, sign language, facial emotions analysis, acting

#### P23 Job satisfaction of bioscientists at a Lisbon hospital

##### Céu Leitão, Renato Abreu, Fernando Bellém, Ana Almeida, Edna Ribeiro-Varandas, Ana Tavares

###### Escola Superior de Tecnologia da Saúde de Lisboa, 1990-096 Lisboa, Portugal

####### **Correspondence:** Céu Leitão (mcleitao@estesl.ipl.pt) – Escola Superior de Tecnologia da Saúde de Lisboa, 1990-096 Lisboa, Portugal

Professional satisfaction is an important phenomenon due to its influence on the human mental resources and physical health, on their attitudes and on their behaviour, both professional and social. The purpose of this study is to assess the actual level of professional satisfaction of bioscientists working at a Lisbon hospital.

A modified questionnaire which allowed the data collection was used for each subject, intended to characterize the sample, order the eight dimensions of professional satisfaction as well determining their levels by using a satisfaction scale. A statistical analysis was made by descriptive methods, Friedman test, Student’s t-test for independent samples and one-way ANOVA.

In a sample of 30 technicians (25.42 % of the target-population), the most important dimension was Status/Prestige (7.12) and the least important was Remuneration (3.12). Global satisfaction shows that they are neither satisfied nor dissatisfied, although the dimension Professional-Patient Relation had better results, in opposition to Remuneration, which had the worst outcome.

The study concluded that the sample’s level of professional satisfaction is not affected by gender, qualifications, years of technical experience and remuneration. The results reveal a state of accommodation and lack of interest regarding labour, which might be a consequence of the actual stagnation in their career. The replication of this study in other public and private institutions is of the most relevance, in order to obtain representative outcomes concerning the professional satisfaction of bioscientists at a national level.

**Keywords**

Evaluation, Professional satisfaction, Motivation

#### P24 Sociodemographic and professional profile of nurses and its relation with the importance of family in nursing practices

##### João G. Frade, Carolina Henriques, Eva Menino, Clarisse Louro, Célia Jordão

###### Unidade de Investigação em Saúde, Escola Superior de Saúde de Leiria, 2411-901 Leiria, Portugal

####### **Correspondence:** João G. Frade (joao.frade@ipleiria.pt) – Unidade de Investigação em Saúde, Escola Superior de Saúde de Leiria, 2411-901 Leiria, Portugal

**Background**

Family health care nursing has been influenced by many variables that are derived from both historical and current events within society and the profession of nursing. In these variables are included issues related with nursing theory, practice, education and research [1]. Objective: This study aims to evaluate the relation between the importance of family in nursing care and nurse’s sociodemographic and professional characteristics.

**Methods**

Descriptive and correlational study of Portuguese nurses, through the questionnaire about the importance of families in nursing care (*Importância da Família nos Cuidados de Enfermagem - Atitude dos Enfermeiros,* IFCE-AE). The results were obtained using non-parametric statistical tests.

**Results**

Respondents were 71 nurses aged 22 to 58 years old. The importance given to the family by nurses surveyed did not vary with age (r = 0.181; p = 0.789) nor with schooling (p = 0.190) or with the specialty of nurses (p = 0.963). Child health and paediatric nurses have assigned greater importance to the family (s = 18.5), and mental health nurses were those who attributed the minor (s = 9.25). Also, the emphasis on family was not associated with the professional category (p = 0.963), the professional exercise time (r = 0.231; p = 0.870), nor the context of the provision of care (p = 0.520). Although primary health care nurses (s = 30.23) gave greater importance to the family presence that differentiated health care nurses (s = 26.32).

**Conclusions**

The sociodemographic and professional characteristics evaluated were not associated with the importance that nurses assign to the family.

**References**

1. Kaakinen, Joanna Rowe, et al. Family health care nursing: Theory, practice, and research. USA: FA Davis, 2014.

**Keywords**

Family, nursing care, health care

#### P25 Professional satisfaction of rehabilitation nurses

##### Sofia Neco^1^, Carminda Morais^2^, Pedro Ferreira^2^

###### ^1^Hospital Santa Maria Maior de Barcelos, 4754-909 Barcelos, Portugal; ^2^Centro de Estudos e Investigação em Saúde, Faculdade de Economia, Universidade de Coimbra, 3004-512 Coimbra Portugal

####### **Correspondence:** Sofia Neco (sofia3rrr@hotmail.com) – Hospital Santa Maria Maior de Barcelos, 4754-909 Barcelos, Portugal

**Background**

Professional satisfaction in nursing constitutes an index of the quality of care provided to the patients, and of the assessment of the organizations’ performance, being of particular relevance the study of the rehabilitation nurses’ satisfaction in the context of renovations in health services. Objective: To know the conditioning factors (positive and negative) of satisfaction; to study the influence of the area of care provided in the professional satisfaction; and to analyse the influence of socio-demographic and professional characteristics in the perceptions of professional satisfaction.

**Methods**

It is a quantitative study, exploratory, descriptive and correlational. The sample is constituted by 60 nurses, exerting functions in hospitals (47) and in ACES (13). For the data collection, the Instrument of Assessment of Professional Satisfaction (IASP, 5th version) was used.

**Results**

No significant differences were found regarding nurses’ professional satisfaction, according to professional category, between specialist nurses and nurses who, being qualified with the specialty, did not accede to this category. Men were the ones who presented more satisfaction, mainly in the quality of the workplace scale (t = -2.26, p < 0.05), and particularly in the human resources policy (t = 2.24, p < 0.05) and moral (t = -2.23, p < 0.05) subscales. Moreover, CSP nurses were also more satisfied with the improvement on the quality of care (F(2, 46) = 4.99, p < 0.01). The non-recognition of specialized education and the underuse of their competencies emerged as dissatisfaction factors, from the open questions.

**Conclusions**

These findings point to an investment in the profitability/recognition of education, crossed by the deconstruction of gender disparities.

**Keywords**

Rehabilitation nurses, Professional satisfaction, health care

#### P26 The person living with a stoma: the formalization of knowledge in nursing

##### Carla R. Silva^1,2^, Alice Brito^2^, Antónia Silva^2^

###### ^1^Universidade Católica Portuguesa/Porto, 4202-401 Porto, Portugal; ^2^Escola Superior de Enfermagem do Porto, 4200-072 Porto, Portugal

####### **Correspondence:** Carla R. Silva (enf_carlasilva@hotmail.com) – Universidade Católica Portuguesa/Porto, 4202-401 Porto, Portugal

**Background**

Information Systems in Nursing are the support of nursing documentation and constitute data repositories capable of being used as raw material for the formalization of nursing knowledge. The Nursing Practice Support System (SAPE), has an electronic format that integrates the International Classification for Nursing Practice (CIPE) and allows nurses to document their decision-making process at different care levels. Objectives: To identify and specify nursing diagnoses describing the needs and nursing interventions that responds to the needs of people with stoma, as well as specifying a referential integrity relationship between them.

**Methods**

Based on a study of exploratory nature, analysis of content of the active national customizations in SAPE in December 2011 was carried out, subsequently validated by two experts. The final result was submitted to a focus group.

**Results**

Ninety-four (94) diagnoses and 165 nursing interventions were obtained, as well as, 323 links between diagnoses and nursing interventions related to the person living with a stoma. The diagnoses documented by nurses take as their nursing focus knowledge and capacity and, at times, lacked referential integrity between these and the nursing interventions.

**Conclusions**

This analysis contributes to the systematization of the knowledge related to the subject and, therefore, to the formalization of this knowledge, enabling the future construction of a Detail Clinical Model of Nursing related to the person living with a stoma.

**Keywords**

Nursing, Information Systems, Stoma, Formalization of knowledge, Detail Clinical Model

#### P27 Validation of the Portuguese versions of the nursing students’ perceptions of learning and learner satisfaction with simulation tool

##### Hugo Duarte, Maria Dos Anjos Dixe, Pedro Sousa

###### Health Research Unit & School of Health Sciences, Polytechnic institute of Leiria, 2411-901 Leiria, Portugal

####### **Correspondence:** Hugo Duarte (hugoduarte2009@gmail.com) – Health Research Unit & School of Health Sciences, Polytechnic institute of Leiria, 2411-901 Leiria, Portugal

**Background**

One of the new methodologies applied to the teaching of nursing is High-Fidelity Simulation (HFS). This type of simulation allows the nursing students to develop their clinical practice by using technological and sophisticated simulators. Recent investigations have studied the importance of HFS, through the level of perception of learning and of satisfaction of the students. Since there is a lack of instruments in Portuguese, we felt the necessity to translate and validate two evaluation scales of HFS. Objectives: Translate, validate transculturally and analyse the psychometrics features of two instruments: “*Nursing Students’ Perceptions of Learning using a High-Fidelity Human Patient Simulator”* and the “*Learner Satisfaction with Simulation Tool”*.

**Methods**

A linguistic and cultural translation was conducted with 139 Portuguese nursing students. Internal consistency reliability and exploratory factor analysis were performed.

**Results**

The Scale of Perception of Learning of the Nursing Students about HFS (SPLNS-HFS) has a Cronbach's Alpha of 0.942 and the Scale of Satisfaction of the Nursing Students about HFS (SSNS-HFS) of 0.969. Values of correlation of each item with the SPLNS-HFS are over 0.507, and of the SSNS-HFS over 0.633. The SPLNS-HFS shows a satisfactory factorial analysis’, with unifactorial structure, and the SSNS-HFS revealed two factors – Utility of Simulation (α = 0.956) and Functionality of Simulation (α = 0.912).

**Conclusions**

The Portuguese versions of both scales are psychometrically reliable, cross-culturally valid, and useful measurement instruments for assessing the impact of High-Fidelity Simulation in nursing students.

**Keywords**

Validation studies, nursing students, High Fidelity Simulation, personal satisfaction, learning perception

#### P28 Physiotherapists’ perceived knowledge on technologies for electronic health records for physiotherapy

##### Gabriela Postolache^1^, Raul Oliveira^2^, Isabel Moreira^3^, Luísa Pedro^4^, Sónia Vicente^5^, Samuel Domingos^6^, Octavian Postolache^6^

###### ^1^Instituto de Medicina Molecular, Universidade de Lisboa, 1649-028 Lisboa, Portugal; ^2^Faculdade de Motricidade Humana, Universidade de Lisboa, Cruz quebrada, 1499-002 Lisboa, Portugal; ^3^Faculdade de Medicina, Universidade de Porto, 4200-319 Porto, Portugal; ^4^Escola Superior de Tecnologias de Saúde, Instituto Politécnico de Lisboa, 1990-096 Lisboa, Portugal; ^5^Escola Superior de Saúde, Universidade Atlântica, 2745-615 Barcarena, Portugal; ^6^Instituto de Telecomunicações, Lisboa, 1049-001, Portugal

####### **Correspondence:** Gabriela Postolache (pgpostolache@gmail.com) – Instituto de Medicina Molecular, Universidade de Lisboa, 1649-028 Lisboa, Portugal

**Background**

In the last decade the amount of evidence has increased indicating that stakeholders’ training and education are key to the successful adoption of electronic health records (EHRs). Also, it has been shown that the availability of technical and training support during the initial implementation is essential. Objective: We investigate the perceived knowledge of EHRs and the motivation for adoption of EHRs in physiotherapy practice.

**Methods**

A cross-sectional study was conducted using a questionnaire developed with 27 questions. Among studied variables were: perceived knowledge of technologies for electronic health records, number of training sessions for adoption of EHRs, motivation related to adoption of EHRs.

**Results**

A random sample of 180 physiotherapists [mean(SD) age: 34.7(±10.3); 70.0 % female; mean and range of years of practice: 11.5 (1-40)] from Portugal was surveyed. Perceived knowledge of technologies for EHRs was low and very low for the majority of physiotherapists (72.2 %). Also, most physiotherapists (75.0 %) reported not having any training related to technologies for EHRs for physiotherapy. The lack of training related with technologies for EHRs was perceived by the majority of physiotherapists (82.2 %) as an important barrier to the adoption of EHRs for physiotherapy. 75.0 % physiotherapists (74.1 % with age ≤ 40 years) reported high motivation to learn about technologies related to EHRs for physiotherapy.

**Conclusions**

The findings indicate that overall, physiotherapists’ needs related to training on technologies for EHRs for physiotherapy are generally not met. Physiotherapists with expertise in informatics may help design EHR systems that meet the needs of physiotherapy, and rigorously evaluate the extent to which these technologies improve care.

**Keywords**

Training, physiotherapists, electronic health records

#### P29 Quality of life and physical activity of medicine undergraduate students in the University of Southern Santa Catarina, Brazil

##### Darlen Silva^1^, João G. Filho^1^, Nazare Nazario^1^, Marcia Kretzer^1^, Dulcineia Schneider^2^

###### ^1^Universidade do Sul de Santa Catarina, Palhoça, Santa Catarina, 88137-270 Brasil; ^2^Universidade Federal de Santa Catarina, Florianópolis, Santa Catarina, 88040-900 Brasil

####### **Correspondence:** Darlen Silva (darlendj@hotmail.com) – Universidade do Sul de Santa Catarina, Palhoça, Santa Catarina, 88137-270 Brasil

**Background**

The Presence of stressors in medical training interfere with quality of life. Physical Activity improves people's overall quality of life. The learning requirements of a medical degree can interfere with the healthy habits and consequently with the student's quality of life. Objective: To analyse the relationship between standards of Physical Activity and the quality of life of Medicine undergraduate students.

**Methods**

Cross-sectional study, executed in the University of Southern Santa Catarina (UNISUL), Brazil, with students from the 1st and 6th year of medical school. The data was collected from March till July of 2014, using the instruments WHOQOL-bref and of physical activity, the IPAQ. Analysis on SPSS 18.0, compared to averages on T-test, with p ≤ 0.05 and confidence interval 95 %. The project was approved by the Ethics Committee for Human Research from UNISUL.

**Results**

One hundred nineteen (119) medicine undergraduates took part in the study, 72.30 % females between 17 and 34 years of age, 87.40 % single. In terms of physical activity, 36.10 % were active and 34.50 % sporadically active. Better quality of life socially (60.40) and worst (63.76) psychologically. Final year students had better quality of life when it comes to the environment (p = 0.035). The very active had better quality of life scores in all domains, without statistical significance. The sedentary presented the lowest average social quality of life.

**Conclusions**

When we associate the physical activity standard with the various domains quality of life, it stands out that the individuals that are classified as “Very Active” present better average quality of life scores across all domains.

**Keywords**

Quality of Life, physical activity, medicine students

#### P30 The curricular skills for decision making education in a Nursing Degree

##### Fátima M. Marques (fmarques@esel.pt)

###### Escola Superior de Enfermagem de Lisboa, Lisboa, 1700-063, Portugal

**Background**

In order for a nursing student to become a qualified professional he needs a diverse set of skills, where decision-making is the cornerstone of nursing care. Teaching skills should structure the curriculum so it is centred on skills acquisition. Objective: To identify the curricular skills for the teaching of decision-making in a Nursing Degree of a school in Lisbon.

**Methods**

An exploratory study of qualitative nature, done with data collection through interviewing teachers and documental analysis of the curriculum. The inclusion criteria for the 10 participants were: being a member of the conceptor group of the school’s syllabus, having experience as a professor of the school’s Nursing Degree and being a course conductor on the school’s Nursing Degree. To analyse the results, Bardin’s content analysis was used. Anonymity and confidentiality of the findings were guaranteed.

**Results**

The order of the structural competences of the analysed curriculum (curriculum and professor’s discourse) begins with the category ‘considering knowledge as a resource to be mobilized’, ‘working through problems’ and ends in the category of ‘guidance for a smaller disciplinary compartmentalization’.

**Conclusions**

The curriculum seems to be focused on the knowledge and the critical thinking that support effective decision-making. However, the course’s structure can attenuate the development of the decision-making apprenticeship in the care process.

**Keywords**

Curricular skills, decision making, nursing degree

#### P31 Effect of nurses’ mobilization in satisfaction at work and turnover: An empirical study in the hospital setting

##### Pedro Parreira^1^, Carla Carvalho^2^, Lisete M. Mónico^2^, Carlos Pinto^2^, Sara Vicente^1^, São João Breda^2^

###### ^1^Escola Superior de Enfermagem de Coimbra, 3046-851 Coimbra, Portugal; ^2^Faculdade de Psicologia e Ciências da Educação, Universidade de Coimbra, 3001-802 Coimbra, Portugal

####### **Correspondence:** Pedro Parreira (parreira@esenfc.pt) – Escola Superior de Enfermagem de Coimbra, 3046-851 Coimbra, Portugal

**Background**

Health organizations are characterized by a complex work environment, being influenced by the behaviour of those who work there. Management strategies play a key role in human resource management, namely in mobilization behaviours that can promote satisfaction at work and decrease turnover. Objective: To analyse whether nurses’ mobilization at work (collaboration; improvement; organizational loyalty; participation in civic life) influence job satisfaction and turnover.

**Methods**

Sample: 338 nurses from a public hospital (mean = 38.63 years-old; 79.2 % females). Measures: I) Behaviours at work [1]: F1-participation in civic life, F2-Collaboration; F3-Organizational loyalty (37.58 %, 10.10 %, and 8.75 % of explained variance, respectively); II) Work satisfaction [2] – unidimensional, 76.10 % of explained variance; and III) Turnover [3]- unidimensional (67.90 % of explained variance). All scales showed good psychometric properties.

**Results**

The adjusted regression model of behaviour at work explained 20 % of the overall satisfaction and only 3 % of the turnover. The organizational loyalty (β = .29) and participation in civic life (β = .18) were predictors of job satisfaction. Collaboration was not a significant predictor (p > .05). Organizational loyalty negatively predicted the turnover (β = - .12).

**Conclusions**

The results contribute to best practices and improvements in the quality of nursing care. It is important to look at the human resources management taking into account their active role, their involvement at work and their commitment to the organization. This seems to enhance satisfaction at work and decrease of turnover. Nurses’ mobilization at work seems to improve the quality of health care and the safety of the patients.

**References**

1. Tremblay M, Wils T, Guay P. L`engagement organisationel el les comportements discrétionnaires: L’influence des pratiques de gestion des ressources humaines. Actes du 11° Congrès de l’Association Francophone de Gestion des Ressources Humaines. Paris. 2000.

2. Brayfield AH, Rothe HF. An index of job satisfaction. Journal of Applied Psychology. 1951; 35: 307-311.

3. Meyer JP, Allen NJ, Smith CA. Commitment to organization and occupation: extension and test of a three-component conceptualization. Journal of Applied Psychology. 1993; 78(4): 538-551.

**Keywords**

Mobilization behaviours, job satisfaction, turnover, nursing, human resources

#### P32 Entrepreneurial skills of students of polytechnic higher education in Portugal: Business influences

##### José H. Gomes, Rosa Melo, Pedro Parreira, Anabela Salgueiro, João Graveto, Marina Vaquinhas, Amélia Castilho

###### Escola Superior de Enfermagem de Coimbra, Coimbra, 3046-851, Portugal

####### **Correspondence:** José H. Gomes (herminio@esenfc.pt) – Escola Superior de Enfermagem de Coimbra, Coimbra, 3046-851 Coimbra, Portugal

**Background**

Most entrepreneurial ideas do not appear ready or finished. Any business opportunity needs to be developed and improved throughout the enterprise process. Educational institutions may facilitate the ability of the students in undertaking, identifying and building business opportunities, enhancing their knowledge and formative experiences along the learning process. Objective: Evaluate business influences on the entrepreneurial ability of students of the Polytechnic.

**Methods**

Correlational quantitative study, conducted with 1,604 students from 18 institutions of the Polytechnic of Portugal.

Data collection took place between July and November/2015, with a questionnaire to assess the entrepreneurial profile, the Carland Entrepreneurship Index (CEI) and sociodemographic variables of students.

**Results**

We found four business factors that influence entrepreneurship: "availability of funds" (4:13, SD = .67); "Having stable customers and incentives" (3.99, SD = .58); "Social and economic instability" (3:08, SD = 1.17) and "Opportunities in the sector and area of residence" (3:36, SD = 1.05). On a scale ranging between 1 and 5, we obtained an overall score of 3.86 (SD = .55), for the corporate influences on entrepreneurship.

**Conclusions**

For students, entrepreneurial influences are important, with a greater sense of fear with regard to economic instability, reinforcing the need for further training and academic investment in business.

**Keywords**

Entrepreneurship, capacity, students, influences

#### P33 Design and assessment of e-learning modules for Pharmacology

##### Ângelo Jesus^1^, Nuno Duarte^1,2^, José C. Lopes^1,3^, Hélder Nunes^1,4^, Agostinho Cruz^1^

###### ^1^Escola Superior de Tecnologia da Saúde, Instituto Politécnico do Porto, 4400-330 Vila Nova de Gaia, Portugal; ^2^Farmácia Outeiro do Linho, 4440-762 Valongo, Portugal; ^3^Hospital Militar Regional n.°1, Porto, 4150-113, Portugal; ^4^Centro Hospitalar do Porto, 4099-001 Porto, Portugal

####### **Correspondence:** Ângelo Jesus (acj@estsp.ipp.pt) – Escola Superior de Tecnologia da Saúde, Instituto Politécnico do Porto, 4400-330 Vila Nova de Gaia, Portugal

**Background**

Teaching and Learning through web-based learning platforms is a complementary method of conventional teaching and learning approaches which has the potential to produce meaningful learning experiences. The study of Pharmacology in the Pharmacy Degree of the School of Allied Health Technologies is normally taught face-to-face. With this paper we aim to describe an online approach to the teaching of this subject. Objectives: To develop and assess two e-learning modules for the teaching of Pharmacology.

**Methods**

Using the MIPO Model for instructional design, Moodle as an LMS, and commercial software, we developed several learning objects that make part of two different modules of Pharmacology. The modules were offered as mandatory training for the third-year students. Knowledge retention was assessed 10 weeks later in a written exam.

**Results**

Two modules and five lessons were developed. Quality assurance was assessed by the Web-Based Learning Environment Questionnaire. Students enrolled in both modules and engaged thoroughly in learning activities and learning contents. All students had a good performance in online quizzes. After 10 weeks, knowledge retention was analysed via a written test. Students were grouped according to their final achievement in the discipline. Students with high and medium achievement grades showed greater knowledge retention than other groups. There was no difference between genders.

**Conclusions**

An online environment was successfully designed and implemented for complementing the teaching of Pharmacology. Knowledge retention does not seem to be associated with shifting the paradigm to online learning, but is probably related to student characteristics or motivations for learning.

**Keywords**

Distance Education, Educational Technology, Online Courses, Web Based Instruction, Instructional Design, Pharmacology

#### P34 Perspective of nurses involved in an action-research study on the changes observed in care provision: results from a focus group

##### Anabela Salgueiro-Oliveira^1^, Pedro Parreira^1^, Marta L. Basto^1^, Luciene M. Braga^2^

###### ^1^Escola Superior de Enfermagem de Coimbra, 3046-851 Coimbra, Portugal; ^2^Universidade Federal de Viçosa, Viçosa - Minas Gerais, 36570-900, Brasil

####### **Correspondence:** Anabela Salgueiro-Oliveira (anabela@esenfc.pt) – Escola Superior de Enfermagem de Coimbra, 3046-851 Coimbra, Portugal

**Background**

According to the Nursing Role Effectiveness Model, the structural components (nurses, patients, organizational variables) may directly or indirectly influence the care outcomes through the process (actions developed by the nurses). Objectives: To identify the changes that, from the nurses’ perspective, occurred during the provision of care to patients with peripheral venous catheters (PVCs) between the first and the second phase of the Action-Research (AR) study, and the components that influenced these changes.

**Methods**

During the second phase of the AR study (December, 2011), a focus group composed of six nurses was held at a medicine unit of a central hospital. A script was used with six open-ended questions. All ethical procedures were followed.

**Results**

Positive changes in nursing care provision to patients with PVCs were identified related to the type of dressing used, patient monitoring, aseptic care, and infusion rate. The nurses believed that some variables of the organizational component influenced those changes, such as the centralization of the material used for catheterization or the availability of materials, such as transparent dressings. The nurses also valued the following aspects: knowledge of the research findings of the first phase; training sessions on the topic; and, above all, the nurses’ engagement throughout the process of change in care provision.

**Conclusions**

Considering the model of analysis used, we found that the changes identified in nursing care resulted from several factors, with the engagement of the professionals themselves in the change process being considered a key aspect.

**Keywords**

Nursing Role Effectiveness Model, nursing care, peripheral venous catheters

#### P35 Use of peer feedback by nursing students during clinical training: teacher’s perception

##### António Ferreira^1^, Beatriz Araújo^2^, José M. Alves^2^, Margarida Ferreira^1^, Maribel Carvalhais^1^, Marilene Silva^3^, Sónia Novais^4^

###### ^1^Escola Superior de Enfermagem da Cruz Vermelha Portuguesa de Oliveira de Azeméis, 3720-126 Oliveira de Azeméis, Portugal; ^2^Universidade Católica Portuguesa, Porto, 4202-401, Portugal; ^3^Unidade de Saúde Familiar - Espaço Saúde, Porto, 4100-503, Portugal; ^4^Administração Regional de Saúde do Norte, Porto, 4000-447, Portugal

####### **Correspondence:** António Ferreira (ferreira.esecvpoa@gmail.com) – Escola Superior de Enfermagem da Cruz Vermelha Portuguesa de Oliveira de Azeméis, 3720-126 Oliveira de Azeméis, Portugal

Peer feedback in the field of higher education, has been defined as an advantageous process in the development of soft skills, improving the learning outcomes of students, reflected in the professional skills to be acquired. The implementation of peer feedback, generates by itself a set of individual and group dynamics, actively involving students and teachers in this methodology, which simultaneously achieves the evaluative and learning purpose.

Through a qualitative approach using the method of focus groups, this study aims to present the perception of teachers on the concept of peer feedback, its applicability in clinical training context in nursing, its influence on skills acquisition by students, and the strength and threats during implementation.

The perception of peer feedback is described by teachers (n = 8) involved in a focus group, which set guidelines for carrying out a participatory action research project in nursing clinical training.

We conclude that peer feedback is a complex process which contributes to increased responsibility for the students but also for teachers in a collaborative and integrative approach to learning and its evaluation. It is evident that preparation and planning of implementation are fundamental, suggesting that peer feedback will positively contribute to students’ development, such as self-directed and self-regulated learning, critical and reflective thinking, self-assessment ability, decision-making, involvement, responsibility and autonomy.

**Keywords**

peer feedback, learning, competences, perception of teachers, nursing

#### P36 What’s new on endotracheal suctioning recommendations

##### Ana S. Sousa^1,2^, Cândida Ferrito^3^

###### ^1^Universidade Católica Portuguesa, Porto, 4202-401, Portugal; ^2^Centro Hospitalar De São João, E.P.E., Porto, 4200–319, Portugal; ^3^Escola Superior de Saúde, Instituto Politécnico de Setúbal, 2910-761 Setúbal, Portugal

####### **Correspondence:** Ana S. Sousa (sabrinasousa72@hotmail.com) – Universidade Católica Portuguesa, Porto, 4202-401, Portugal

**Background**

Critically ill intubated patients inevitably need endotracheal suctioning. There are innumerable complications associated with this procedure, including hypoxemia, atelectasis, tissue trauma, hypertension, microbial colonization and bronchospasm. Best practices can reduce endotracheal-suctioning-related adverse events. Objective: The aim of this study is to describe endotracheal suctioning recommendations in adult mechanically ventilated patients.

**Methods**

An integrative review. Research was conducted in B-on, PUBMED, and RCAAP between 28 and 30 December 2015, including guidelines and original articles from the last 5 years. We found 534 documents and after analysing their abstract and methodological quality, five documents were selected. Data were compiled in a chart in terms of grade of evidence, acceptance and applicability.

**Results**

Strong evidence was found in the following recommendations: suction only when necessary, pre-oxygen before suction event, use of a suction catheter half the endotracheal tube lumen size, use of the lowest suction pressure possible, shallow suction, avoiding saline instillation, use of sterile technique, avoiding disconnection from the ventilator, use of closed suction when there is high FiO2 or PEEP or risk of lung derecruitment, suction duration less than 15 s and monitoring.

**Conclusions**

Endotracheal-suctioning-related adverse events are frequent and can be reduced by the implementation of practice guidelines. Nurses need to have to knowledge of current best evidence in order to make informed decisions. This review intends to be a step towards safer practice.

**Keywords**

ICU, Endotracheal suction, Best practice recommendations, Saline instillation, Open suction, Closed suction

#### P37 Assessment of the nurses satisfaction on the Central Region of Portugal

##### Pedro L. Ferreira^1^, Alexandre Rodrigues^2^, Margarida Ferreira^3^, Isabel Oliveira^4^

###### ^1^Faculdade de Economia, Universidade de Coimbra, 3004-512 Coimbra Portugal; ^2^Universidade de Aveiro, 3810-193 Aveiro, Portugal; ^3^Escola Superior de Enfermagem da Cruz Vermelha Portuguesa de Oliveira de Azeméis, 3720-126 Oliveira de Azeméis, Portugal; ^4^Ordem dos Enfermeiros da zona Centro, Coimbra, 3000-076, Portugal

####### **Correspondence:** Isabel Oliveira (ijoliveira@sapo.pt) – Faculdade de Economia, Universidade de Coimbra, 3004-512 Coimbra Portugal

**Background**

The changes occurred in labour in the last decades are consuming workers’ physical and mental energy. Nurses’ job satisfaction is of most importance since they have responsibilities within the multidisciplinary team and is far from being a secondary concern because it is directly linked to the quality of care, productivity and personal fulfilment. It is a challenge for safety and health at work, representing a significant impact on health, requiring an active and dynamic involvement of the employer, workers and their representatives in the management of job satisfaction. Objective: Evaluate the level of satisfaction of nurses in the central region of Portugal.

**Methods**

This was a descriptive and transversal study. Nurses from the Central Region of Portugal were asked to fill a staff satisfaction internet questionnaire based on the Professional Satisfaction Assessment Tool (IASP).

**Results**

A sample of 1,126 nurses answered the questionnaire, 71.8 % of them female. On average they were 39.7 ± 9.8 years old, ranging from a minimum of 22 to a maximum of 65. The majority (69.0 %) was married or living together with a partner and working in the public sector (hospital: 59.1 %; primary care: 26.5 %). In general, about half of the nurses were “satisfied” or “very satisfied” with the organization where they work and 28.0 % were “unsatisfied” or “very unsatisfied”. At last, almost 5.0 % of the nurses had already emigrated and returned to Portugal and 15.0 % were planning to emigrate.

**Conclusions**

We evidenced a significant low satisfaction among the nurses and some recommendations were drawn from this study.

**Keywords**

Work, satisfaction, nurses

#### P38 Study of graduate’s satisfaction with the school of nursing

##### Manuela Ferreira, Jéssica Neves, Diana Costa, Soraia Duarte, Joana Silva, Bruno Santos

###### Escola Superior de Enfermagem da Cruz Vermelha Portuguesa, 3720-126 Oliveira de Azeméis, Portugal

####### **Correspondence:** Manuela Ferreira (ferreiramanuela75@gmail.com) – Escola Superior de Enfermagem da Cruz Vermelha Portuguesa, 3720-126 Oliveira de Azeméis, Portugal

**Background**

The increasing number of graduated nurses, the reduction of employment opportunities in the country and the poor working conditions/salary cause many graduates to begin their nursing profession finding themselves in a less positive context. Schools are a factor that can influence this initiation context in the profession. Objectives: Characterize satisfaction with the nursing school.

**Methods**

This is a descriptive study in a sample of 141 graduates between 2002 and 2014 in a nursing school of the Northern Region of Portugal. The "Satisfaction Scale with the Professional Status of Nurse" was created and validated based on a literature review. It is a Likert scale of 1 to 5 points: 1 – very dissatisfied and 5 – very satisfied. The factor analysis with orthogonal rotation Varimax type scale – composed of 25 items – reveals good value for Kaiser-Meyer-Olkin (0.901) and high internal consistency (α = 0.851). Three dimensions were differentiated: career, professional self-fulfilment and social representation of nursing.

**Results**

All dimensions have a degree of satisfaction with the school: skills development (mean = 4.05, SD =0.46); linking (mean = 4.01, SD = 0.52); centrality of the student (mean = 3.97, SD = 0.94); interpersonal relationships (mean = 4.10, SD = 0.59); and the reference in nursing education (mean = 3.90, SD = 0.60).

**Conclusions**

Graduates Nurses reveal themselves satisfied with the school, which is a promoting factor to mitigate the difficulties in the job market. Schools have to adapt ever more to the current contexts of the learning/education process and work of nurses.

**Keywords**

Scale validation, Satisfaction with the graduated School, Nursing graduates

#### P39 Partnership between the school of nursing and the hospital: Supervisors´ perspectives

##### Cristina Martins, Ana P. Macedo, Odete Araújo, Cláudia Augusto, Fátima Braga, Lisa Gomes, Maria A. Silva, Rafaela Rosário

###### Escola Superior de Enfermagem, Universidade do Minho, Braga, 4710-057, Portugal

####### **Correspondence:** Cristina Martins (cmartins@ese.uminho.pt) – Escola Superior de Enfermagem, Universidade do Minho, Braga, 4710-057, Portugal

**Background**

Partnership is commonplace in life and is characterized by a relationship and shared goals as well as common purpose. The research into partnership as a phenomenon among organizations is scarce and nowadays this partnership is more urgent than before to work more effectively and with a greater sense of efficacy. Objective: To explore, via a focus group, the supervisors’ representations about the partnership between the school of nursing and the hospital.

**Methods**

Qualitative study. Five sessions following a focus group approach were developed focusing on a discussion about the partnership between the school of nursing and the hospital. Eighteen supervisors (eleven from the local hospital and seven from the academy) participated in this focus group (nine supervisors allocated equally in two groups). Data collection also included interviews and field notes analysed according to qualitative content analysis.

**Results**

Three categories emerged as cornerstones of a framework. These categories are expectations related to clinical supervision, understanding the partnership between the school of nursing and hospital and a collaborative approach as a way to enhance the development of quality references.

**Conclusions**

This study provides further support for the improvement of the partnership between the school of nursing and the hospital. It identifies the need for a partnership between the school and hospital, which includes preparation/joint planning and meetings.

**Keywords**

Partnership, supervision, collaborative supervision, focus group

#### P40 Coping strategies of college students

##### Luís Pimenta, Diana Carreira, Patrícia Teles, Teresa Barros

###### School of Health Sciences, Polytechnic institute of Leiria, 2411-901 Leiria, Portugal

####### **Correspondence:** Teresa Barros (teresa.kraus@ipleiria.pt) – School of Health Sciences, Polytechnic institute of Leiria, 2411-901 Leiria, Portugal

**Background**

In college, students are faced with potentially disturbing life events, and to adapt to the new context, they need to use coping strategies [1]. Objectives: I) To understand more about the coping strategies that college students use; II) to evaluate the relationship between coping strategies used by students with their age and gender.

**Methods**

Correlational study performed in 2015, with 242 students from a college in the centre of Portugal, who have replied to a survey in Google Docs consisting of the Toulousaine Scale of Coping [2] and a sociodemographic and academic questionnaire. Data was analysed using descriptive statistics and non-parametric inference.

**Results**

The study sample was composed by 242 students with a mean age of 21.78 years old (SD = 4.78), being mostly female (n = 189/78.1 %). The coping strategy used by both genders, but particularly by males, is the “control”, having verified a positive and statistically meaningful relation between the use of this strategy and age (p = 0.004). On the other hand, the female gender uses social support, with statistical significance (p = 0.001).

**Conclusions**

Coping strategies that students most commonly use are “control” and “social support”, where male participants rely more on “control”, and female on “social support”. We could also conclude that older students use “control” more often and it’s verified a positive relation between “control” and age.

**References**

1. Costa E, Leal I. Estratégias de Coping em Estudantes do Ensino Superior. Análise Psicológica. 2006; 2(24): 189-199.

2. Tap P, Costa E, Alves M. Escala Toulousiana de Coping (ETC): Estudo de Adaptação à População Portuguesa. Psicologia, Saúde & Doenças, 2005; 6(1): 47-56.

**Keywords**

Psychological adaptation, students, college

#### P41 Emotional intelligence and mental health stigma in health students

##### Catarina Tomás^1^, Ana Querido^1^, Daniel Carvalho^1,2^, João Gomes^1,2^, Marina Cordeiro^1^

###### ^1^School of Health Sciences, Polytechnic institute of Leiria, 2411-901 Leiria, Portugal; ^2^Hospital de Santo André, Centro Hospitalar de Leiria, 2410-197 Leiria, Portugal

####### **Correspondence:** Catarina Tomás (catarina.tomas@ipleiria.pt) – School of Health Sciences, Polytechnic institute of Leiria, 2411-901 Leiria, Portugal

**Background**

Health professionals need good emotional intelligence to deal with patients, and to have effective communication, attending patients’ physical, mental and emotional and needs. Having good emotional intelligence leads to a positive attitude towards mental health patients. Objectives: The aim of this study is to find the relation between mental health stigma and emotional intelligence in students of a Portuguese health college.

**Methods**

This is a quantitative, descriptive and correlational study, with a non-probabilistic sample of 672 students of a Portuguese health college in a central region, which comprises students of Nursing, Dietetics, Physiotherapy, Speech Therapy and Occupational Therapy. To collect data, a questionnaire was used composed of sociodemographic questions, the Portuguese version of the Attribution Questionnaire (AQ-27) and the Emotional Intelligence Scale (WLEIS-P).

**Results**

The sample is mainly composed by female students (82.7 %) with ages between 17 and 56 years old. The mental health stigma was moderate (4.19; SD = 0.732), and emotional intelligence is good (3.62; SD = 0.422). A negative correlation was found (R = -0.081; p = 0.037) between help and emotional intelligence. Help (R = -0.152; p = 0.000) and avoidance (R = -0.121; p = 0.002) have also a negative correlation with the emotional assessment of the other person, and pity is correlated with self-emotional appraisal (R = -0.088; p = 0.022). Dangerousness and use of emotions are positively correlated (R = 0.094; p = 0.015).

**Conclusions**

Emotional intelligence can be negatively related to mental health stigma, although dangerousness seems to engage with the use of emotions. It is important to enhance emotional intelligence in health students, in order to reduce mental health stigma.

**Keywords**

Mental health, stigmatization, emotional Intelligence

#### P42 Stigma of mental health assessment: Comparison between health courses

##### Daniel Carvalho^1,2^, Ana Querido^2^, Catarina Tomás^2^, João Gomes^1,2^, Marina Cordeiro^2^

###### ^1^Hospital de Santo André, Centro Hospitalar de Leiria, 2410-197 Leiria, Portugal; ^2^School of Health Sciences, Polytechnic institute of Leiria, 2411-901 Leiria, Portugal

####### **Correspondence:** Daniel Carvalho (drscarvalho@gmail.com) – Hospital de Santo André, Centro Hospitalar de Leiria, 2410-197 Leiria, Portugal

**Background**

Health workers are among the stigmatizing agents as regards mental health. It is important to look at this issue in future professionals as they may play a role in changes in the future. Some studies on health professionals show slightly higher levels of stigma in women, while the fear factor decreases with age. Objective: This study aimed to evaluate the level of stigma in mental health with health students of a Portuguese health college and the relation to gender, age and course.

**Methods**

A quantitative, descriptive and correlational transversal study was conducted on a non-probabilistic convenience sample of 672 students from dietetic, nursing, physical, speech and occupational therapy courses. For data collection a questionnaire was used consisting of sociodemographic questions and the Portuguese version of the Attribution Questionnaire (AQ27).

**Results**

The study sample consists mostly of female students (82.7 %) with a mean age of 21.16 years (SD = 4.18). The level of mental health stigma of the sample is considered moderate (4.19; SD = 0.73), and equal within gender. With age, there is an increase in the factors responsibility, aid and avoidance (p < 0.05), with a reduction in compassion, dangerousness and fear (p < 0.05). Stigma levels are lower in students of occupational therapy, both in total score and in aid and avoidance. Physiotherapy students had the lowest values of coercion and segregation. All dimensions of stigma decrease during the course (p = < 0.05) with the exception of aid and responsibility, whose value increased (p < 0.05).

**Conclusions**

Factors were found in the sample that may be considered stigmatizing, which raises the need to rethink pedagogical strategies for the reduction of stigma.

**Keywords**

Social Stigma, mental health, students

### Effectiveness of Health Intervention programmes

#### O81 Short- and long-term effects of pulmonary rehabilitation in mild COPD

##### Cristina Jácome^1^, Alda Marques^2^

###### ^1^Research Centre in Physical Activity, Health and Leisure, Faculty of Sports, University of Porto, 4200-450 Porto, Portugal; ^2^Lab 3R – Respiratory Research and Rehabilitation Laboratory, School of Health Sciences, University of Aveiro, 3810-193 Aveiro, Portugal

####### **Correspondence:** Cristina Jácome (cristinajacome@ua.pt) – Research Centre in Physical Activity, Health and Leisure, Faculty of Sports, University of Porto, 4200-450 Porto, Portugal

**Background**

It is well established that pulmonary rehabilitation (PR) is effective in improving dyspnea and health-related quality of life of patients with moderate-to-very-severe Chronic Obstructive Pulmonary Disease (COPD). However, the effects of PR in patients with mild COPD have been little explored. Thus, this study investigated the short- and long-term effects of PR on patients with mild COPD.

**Methods**

This was a single-arm longitudinal study. A 12-week PR program with exercise training and psychoeducation was conducted in the community. Outcome measures at baseline, post-PR, 3 and 6 months were the 6-minute walk test (6 MWT) for exercise tolerance; the Modified British Medical Research Council questionnaire for dyspnea; 1-repetition maximum on the chest press and leg extension exercises for peripheral muscle strength; the Brief physical activity assessment for self-reported physical activity and the St. George’s Respiratory Questionnaire (SGRQ) for health-related quality of life.

**Results**

Thirty-two patients (65.94 ± 8.95 years, FEV1 86.7 ± 5.17 % predicted) completed the program. After PR, significant improvements were observed in all measures (all p < .004), with the exception of SGRQ symptoms (p = .013) and impact (p = .104) scores. Compared to baseline, significant improvements in the 6 MWT, leg extension exercise, self-reported physical activity and SGRQ total and symptoms scores were still observed 6 months after PR (all p < .002).

**Conclusions**

PR improves exercise tolerance, dyspnea, muscle strength, physical activity and health-related quality of life in patients with mild COPD, and most of these benefits last for at least 6 months. These data suggest that PR should be part of the first-line management of patients with mild COPD. Further work is warranted prior to broader implementation.

**Keywords**

COPD, mild COPD, GOLD 1, pulmonary rehabilitation, exercise training

#### O82 Phonological awareness programme for preschool children

##### Sylvie Capelas^1^, Andreia Hall^2^, Dina Alves^3^, Marisa Lousada^4^

###### ^1^Unidade de Surdos, Escola de Referência para a Educação Bilingue, 3830-195 Ílhavo, Portugal; ^2^Center for Research and Development in Mathematics and Applications & Department of Mathematics, University of Aveiro, 3810-193 Aveiro, Portugal; ^3^School of Health Care, Polytechnic Institute of Setúbal, 2910-761 Setúbal, Portugal & Centro de Linguística da Universidade de Lisboa, Faculdade de Letras, Universidade de Lisboa, 1600-214 Lisboa, Portugal; ^4^Center for Health Technology and Services Research, School of Health Sciences, University of Aveiro, Aveiro, 3810-193, Portugal

####### **Correspondence:** Marisa Lousada (marisalousada@ua.pt) – Center for Health Technology and Services Research, School of Health Sciences, University of Aveiro, Aveiro, 3810-193, Portugal

**Background**

In the phonological disorder domain, it is common to observe poor phonological awareness skills and, consequently, a later or deficit literacy learning. As so, it is important that speech and language therapists stimulate early phonological awareness to prevent reading and spelling difficulties in children with phonological disorder. Objective: This study investigated the effectiveness of a phonological awareness programme for preschool children with phonological disorder. Sixty-one Portuguese children, aged 4.0 - 6.2 years participated in this study. Twenty-one children with phonological disorder and 40 typically developing children.

**Methods**

Children received 10 sessions focused on phonological awareness abilities, specifically on syllabic and phonemic awareness (blending, segmentation and manipulation). For ethical reasons, children were not assigned to an intervention control group. Rather, all children from a kindergarten were invited to participate in the programme. Speech-language assessment and diagnostic were performed by a speech and language therapist. Outcome measures of phonological awareness ability (related to syllabic and phonemic units) were taken before and after the intervention.

**Results**

Results revealed that both groups improved significantly after intervention (p = 0.041 for syllabic awareness; p = 0.011 for phonemic awareness). Despite significant differences between groups at pre-treatment assessment, children with phonological disorder showed a more significant improvement for syllabic awareness (p = 0.004).

**Conclusions**

The findings suggest that the phonological awareness programme was effective in the improvement of syllabic and phonemic awareness.

**Keywords**

Syllabic awareness, phonemic awareness, intervention, preschool children, phonological disorder

#### O83 REforma ATIVA: An efficient health promotion program to be implemented during retirement

##### Mª Helena Loureiro^1^, Ana Camarneiro^1^, Margarida Silva^1^, Aida Mendes^1^, Ana Pedreiro^2^

###### ^1^Escola Superior de Enfermagem de Coimbra, 3046-851 Coimbra, Portugal; ^2^Unidade de Investigação em Ciências da Saúde: Enfermagem, Escola Superior de Enfermagem de Coimbra, 3046-851 Coimbra, Portugal

####### **Correspondence:** Mª Helena Loureiro (hloureiro@ua.pt) – Escola Superior de Enfermagem de Coimbra, 3046-851 Coimbra, Portugal

**Background**

Retirement is one of the major adult life transitions that can originate different vulnerabilities. Implementation of a health promotion program in this life stage can avoid vulnerabilities and contribute to promoting health benefits. REATIVA, a Portuguese project funded by Fundação para a Ciência e Tecnologia (FCT), has as its goal to create a health promotion program to increase self-efficacy and adaptation perception during the transition to retirement. In this work we present the efficiency of this program, called REforma ATIVA_Program.

**Methods**

Quasi-experimental design study, involving 3 groups: 2 experimental (EG1/EG2) and 1 control group (CG). The target population comprised recent retirees enrolled in the Health Administration Region – Centre/Portugal. The effects of the program implementation have been evaluated by applying an instrument that measured self-efficacy (General Self-Efficacy Scale, GSE) and perception of adaptation to retirement transition (Escala de Posicionamento Face à Adaptação à Reforma, EPFAR). All participants have signed informed consent. Data was processed using SPSS Program.

**Results**

There was a positive change in GSE and EPFAR on average, in all participants who have undergone the REforma ATIVA Program (EG1/EG2). The most notable evidence of its effect has been noted in EPFAR, in which was found, using MANOVA (Greenhouse-Gerssen test), that there was a significant effect of the program in adaptation to retirement over time (F = 17.405, p = 0.001; ƞ2 = 0.554; PO = 0.982).

**Conclusions**

The efficiency of REforma ATIVA Program denoted the added value that an intervention of this nature can have in the promotion of individual and family health during transition to retirement, conducting to an active and healthy aging process.

**Keywords**

Health Promotion Program, retirement, active aging

#### O84 Intervention for men who batter women, a case report

##### Anne G.Silva, Elza S. Coelho

###### Universidade Federal de Santa Catarina, Florianópolis, Santa Catarina, 88040-900, Brasil

####### **Correspondence:** Anne G.Silva (anne_clg@hotmail.com) – Universidade Federal de Santa Catarina, Florianópolis, Santa Catarina, 88040-900, Brasil

Intimate partner violence is one of the most common forms of violence. Although men are the main authors of violence, it is essential to include actions to help them in order to produce a more effective result, because it is believed that they are able to recognize and take responsibility for the violence, using different forms of self-expression. However, it is difficult to assess the results of these actions.

This research aims to analyse the relapse of violence after participation in a batterer intervention programme. It was conducted a case study in a batterer intervention programme, where 86 men agreed to participate. The Centres for Disease Control and Prevention - Follow up questionnaire was used, adapted to be used in Brazil.

Most participants (61 %) were aged between 30 and 40, had studied 11 years (26 %), were employed (89 %), 60 % were separated, 56 % had a new relationship and 19 % had no contact with the ex-wife. About violence perpetration in the last 3 months, one reported committing physical abuse, 9 % psychological and 21 % reported one situation when they felt like hitting their partner.

The majority of participants (84 %) said that nothing would lead them to assault their partner, 31 % described behavioural changes after participation in the programme and one reported no change.

The study concludes that batterer intervention programmes have positive influence on participants; however, despite lower occurrence of physical violence, there were critical situations and psychological violence. Longer follow-ups including women are necessary, but this intervention can be a way to decrease violence against women.

**Keywords**

Violence against women, batterer intervention, men

#### O85 Immediate effects of Bowen Therapy on muscle tone and flexibility

##### Flávio Melo^1,2^, Fernando Ribeiro^1,3^, Rui Torres^4^, Rui Costa^1,5^

###### ^1^School of Health Sciences, University of Aveiro, Aveiro, 3810-193, Portugal; ^2^Peroneo Centro Terapêutico Lda, Amieiro, Montemor-o-velho, 3140-021 Arazede, Portugal; ^3^Institute for Research in Biomedicine, University of Aveiro, Aveiro, 3810-292, Portugal; ^4^Department of Physiotherapy, North Polytechnic Institute of Health, Gandra, 4585-116, Portugal; ^5^Center for Health Technology and Services Research, University of Aveiro, Aveiro, 3810-193, Portugal

####### **Correspondence:** Flávio Melo (flavio.melo.2010@gmail.com) – School of Health Sciences, University of Aveiro, Aveiro, 3810-193, Portugal

**Background**

Bowen Therapy is a noninvasive technique that uses a series of gentle hand movements over muscles, tendons, ligaments, joints, nerves and fascia to promote relief from musculoskeletal and related neurological complaints. Although little research-based effectiveness data are available, some studies showed positive effects on pain perception, range of motion, functionality and lymphatic circulation. Objective: To investigate the immediate effects of Bowen Therapy on the mechanical properties – tone, elasticity and stiffness – of the erector spinae and biceps femoris muscles and hamstring flexibility.

**Methods**

Twenty-one healthy individuals (mean age: 21.05 ± 3.79 years old), 9 male and 13 female, participated in a crossover study. They were randomly allocated to a placebo intervention or a Bowen therapy session with 8-day interval period. Before and after the intervention, hamstring flexibility was assessed by the "Sit and Reach test”; and the mechanical properties – tone, elasticity and stiffness – of the erector spinae and biceps femoris muscles were measured bilaterally using a handheld mechanical impulse-based myotonometric device.

**Results**

No significant changes were observed in muscle tone, elasticity or stiffness with the application of the Bowen Therapy session, but a significant improvement was observed in hamstring flexibility (34 to 42 cm, p < 0.001) in comparison with the placebo session.

**Conclusions**

A single session of Bowen Therapy immediately increases hamstring flexibility, but do not change muscle tone, elasticity and stiffness in healthy individuals.

**Keywords**

Bowen therapy, muscle tonus, elasticity, MyotonPRO, fascia

#### O86 Predictive equation for incremental shuttle walk test in adolescents

##### Tânia Pinho^1^, Cristina Jácome^2^, Alda Marques^1^

###### ^1^School of Health Sciences, University of Aveiro, 3810-193 Aveiro, Portugal; ^2^Research Centre in Physical Activity, Health and Leisure, Faculty of Sports, University of Porto, 4200-450 Porto, Portugal

####### **Correspondence:** Alda Marques (amarques@ua.pt) – School of Health Sciences, University of Aveiro, 3810-193 Aveiro, Portugal

**Background**

The incremental shuttle walk test (ISWT) is one of the most used measures to assess cardiorespiratory fitness in clinical and research settings. Reference equations to predict ISWT distance (ISWD) in different adult populations have been established. However, equations for adolescents have been less explored. Objective: This study developed a reference equation to predict the ISWD for adolescents.

**Methods**

Healthy Portuguese adolescents aged 12–17 years were included. Socio-demographic and anthropometric (body mass index [BMI]) data were first collected. Lung function was assessed with spirometry and quadriceps muscular strength (QMS) with hand-held dynamometry. Physical activity level was evaluated through the Physical Activity Index (PAI). ISWT was performed twice and the best ISWD was used for analysis.

**Results**

A total of 125 participants (56 male; 14.6 ± 1.3 years) with normal lung function (forced expiratory volume in one second 104.8 ± 14.9 % predicted) completed the assessment. According to PAI, 54.4 % (n = 63) of the participants were moderately active and mean QMS was 20.7 ± 6.8 kgf. Participants walked on average 1254.0 ± 280.9 m [760–2250] in the ISWT. A multiple regression model showed that sex, BMI, QMS and PAI were independent contributors to the ISWD, explaining 54 % of the variability (p < 0.05). The reference equation was: ISWD = 814.49 + (286.80 × sex) - (3.05 × BMI) + (8.83 × QMS) + (22.30 × PAI), sex: female = 0, male = 1.

**Conclusions**

Sex, BMI, QMS and PAI were predictors of the ISWD in adolescents, providing a simple reference to assess their cardiorespiratory fitness. This equation is a valuable tool to interpret ISWD obtained from Portuguese adolescents, with or without a health condition.

**Keywords**

Cardiorespiratory fitness, Incremental Shuttle Walk Test, predictive equation, adolescents

#### O87 Life satisfaction and psychopathology in institutionalized elderly people: The results of an adapted Mindfulness-Based Stress Reduction program

##### Bárbara Cruz^1^, Daniel Seabra^2^, Diogo Carreiras^3^, Maria Ventura^1^

###### ^1^Irmandade da Misericórdia de Albergaria-a-Velha, 3850-069 Albergaria-a-Velha, Portugal; ^2^Faculdade de Psicologia e Ciências da Educação, Universidade de Coimbra, 3001-802 Coimbra, Portugal; ^3^Gabinete de Apoio Psicológico, Instituto Superior Miguel Torga, Coimbra, 3000-132, Portugal

####### **Correspondence:** Daniel Seabra (daniel_seabra_@hotmail.com) – Faculdade de Psicologia e Ciências da Educação, Universidade de Coimbra, 3001-802, Portugal

**Background**

Elderly people tend to experience higher levels of depression, anxiety, subjective pain and experiential avoidance when compared with younger people. Recently, several studies have been giving empirical support to mindfulness practice as a way to improve well-being and life satisfaction. Objective: The present study aimed to test the benefits of a Mindfulness-Based Stress Reduction (MBSR) program in Portuguese institutionalized elderly people, as a way to improve life satisfaction and reduce psychopathology.

**Methods**

The present sample included 12 institutionalized elderly people, 9 females (75 %) and 3 males (25 %), with ages between 65 and 91 (M = 82.58 years; SD = 7.87), that filled several self-report questionnaires and were assessed with Mini Mental State Examination. Our exclusion criteria were the presence of cognitive or sensorial impairment, illiteracy, and age under 65 years. They attended to an adapted Mindfulness-Based Stress Reduction program, with 48 sessions (16 weeks) and they were evaluated before and after the intervention.

**Results**

The comparative analysis (Wilcoxon rank-sum test) revealed that depression, anxiety, experiential avoidance and subjective pain decreased significantly after the program. On the contrary, life satisfaction increased significantly.

**Conclusions**

The results suggest that the MBSR program is a useful intervention to reduce psychopathology and increase life satisfaction in institutionalized elderly people. The application of these interventions may be very useful in elderly care institutions. Future research should enlarge the sample, include a control group and assess follow-ups.

ClinicalTrials.gov Identifier: NCT02774018.

**Keywords**

Institutionalized elderly, mindfulness, life satisfaction, depression, anxiety, subjective pain

#### O88 Outcome changes in COPD rehabilitation: exploring the relationship between physical activity and health-related outcomes

##### Joana Cruz^1^, Dina Brooks^2^, Alda Marques^1^

###### ^1^School of Health Sciences, University of Aveiro, 3810-193 Aveiro, Portugal; ^2^Rehabilitation Science Institute and Department of Physical Therapy, University of Toronto, Toronto, Ontario M5S 1A1 Canada

####### **Correspondence:** Joana Cruz (joana.cruz@ua.pt) – School of Health Sciences, University of Aveiro, 3810-193 Aveiro, Portugal

**Background**

Reduced physical activity (PA) levels are associated with poor health-related outcomes in patients with Chronic Obstructive Pulmonary Disease (COPD). PA-focused interventions complementary to pulmonary rehabilitation (PR) have been developed to increase patients’ PA. However, it is unknown whether PA changes are related to health-related outcomes improvement. This study explored the relationship between changes in PA and health-related outcomes in patients with COPD.

**Methods**

Thirteen patients with COPD (65.6 ± 10.6 yrs) participated in a 12-week PR programme plus a PA-focused intervention. Daily PA was measured using accelerometers on weeks (W) 1, 7 and 12 and feedback was given to participants in the following weeks regarding: daily steps; time spent in sedentary, light and moderate-to-vigorous (MVPA) intensity activities. Exercise capacity (6-minute walk test), functional balance (Timed Up-and-Go (TUG) test) and health-related quality of life (St George’s Respiratory Questionnaire (SGRQ) – Symptoms, Activities, Impact) were assessed at W1/W12. Correlations between PA data and health-related outcomes were performed at W1 and using the change scores (W12-W1).

**Results**

At W1, time spent in MVPA was correlated with exercise capacity (r = 0.817, p = 0.001) and TUG (r = -0.692, p = 0.009). Changes in MVPA time were correlated with changes in TUG (r = -0.653, p = 0.016) and SGRQ Symptoms (r = -0.588, p = 0.035). The latter was also correlated with changes in sedentary time (r = 0.760, p = 0.003). No other significant correlations were found.

**Conclusions**

Patients with better exercise capacity and functional balance were also more physically active at W1. Nevertheless, findings suggest that intervention-related improvements in symptoms and functional balance may contribute to PA changes in a greater extent than exercise capacity. More research is needed.

**Keywords**

Active lifestyle, activity monitoring, chronic respiratory disease, COPD

#### O89 Assessing the effectiveness of a Complex Nursing Intervention

##### M Rosário Pinto^1^, Pedro Parreira^2^, Marta Lima-Basto^3^, Miguel Neves^4^, Lisete M. Mónico^5^

###### ^1^Escola Superior de Saúde de Santarém, Santarém, 2005-075, Portugal; ^2^Escola Superior de Enfermagem de Coimbra, Coimbra, 3046-851, Portugal; ^3^Unidade de Investigação & Desenvolvimento em Enfermagem, Escola Superior de Enfermagem de Lisboa, Lisboa, 1700-063, Portugal; ^4^Hospital Distrital de Santarém, EPE, Santarém, 2005-177, Portugal; ^5^Universidade de Coimbra, 3004-504 Coimbra, Portugal

####### **Correspondence:** M Rosário Pinto (mrosariopinto.essaude@gmail.com) – Escola Superior de Saúde de Santarém, Santarém, 2005-075, Portugal

**Background**

Nurses are engaged in patient education as an empowering patient-centred approach increases self-management and self-control, making this activity one of the most significant in nursing practice. Objective: To evaluate the effect of a nurses’ lifestyle education programme on type 2 diabetic patients’ metabolic control and self-care behaviours.

**Methods**

A controlled before and after experimental study was conducted to assess the effectiveness of the programme consisting of a sequence of individual, group and telephone educational interventions, applied over 24 weeks. 64 subjects completed the study in the experimental group (EG) and 58 in the comparison group (CG) (CES RLVT process number 039/CES/INV/2014). As it aimed to change self-care behaviours and increase metabolic control, Glycosylated haemoglobin, Body Mass Index (BMC) and self-care activities (Summary of Diabetes Self-Care Activities Scale) were assessed before and after the intervention programme.

**Results**

Mean percentage change in glycosylated haemoglobin was more significant in EG (–0.79 %, SD = 1.3) than in CG (-0.05 %, SD = .65), while BMCs decreased in EC (-0.50 kg/m^2^, SD = 1.5) and increased in CG (+0.04 kg/m^2^, SD = 1.2). Self-care activities showed positive changes, more significant in the experimental group. Expected behaviours concerning food increased by a mean of 2 days/week and physical activity 1 day/week in EG while in CG changes were lower (+0.6 days/week and +0.28 days/week, respectively). Daily foot examination increased 1.8 days/week in EG and only 0.14 days/week in CG.

**Conclusions**

The nursing educational programmed designed was effective in people with type 2 diabetes, resulting in higher metabolic control and more frequent self-care behaviours after the intervention was concluded.

**Keywords**

Complex Intervention effectiveness, nursing, *Diabetes mellitus*, Lifestyle Educational Program

#### O90 Psychotherapeutic intervention in addiction disorders: Change in psychopathological symptoms and emotional states

##### Carla Bizarro^1^, Marina Cunha^1^, Ana Galhardo^1^, Couto Margarida^1^, Ana P. Amorim^1^, Eduardo Silva^2^

###### ^1^Instituto Superior Miguel Torga, Coimbra, 3000-132, Portugal; ^2^Centro de Tratamento Internacional VillaRamadas, 2460-355 Cela, Alcobaça, Portugal

####### **Correspondence:** Carla Bizarro (bizarro.carla@gmail.com) – Instituto Superior Miguel Torga, Coimbra, 3000-132, Portugal

**Background**

Addiction is considered a complex phenomenon, set within a framework of equal complexity, given the amount of variables which interfere with it and, at the same time, are influenced by it. Objective: This study aims to explore the existence of changes in psychopathological symptoms and in emotional states, in patients undergoing treatment in a therapeutic community (applying the Change & Grow Therapeutic Model), specialized in treating addiction disorders.

**Methods**

The sample consists of 32 patients with ages between 15 and 55 years old of both genders. Patients filled in a sociodemographic questionnaire and the following self-report questionnaires: Depression, Anxiety and Stress Scale, Positive and Negative Affect Schedule, Internalized Shame Scale, Other as Shamer Brief, Aggressive Questionnaire and the Barratt Impulsiveness Scale. These instruments were administered at two distinct moments, with a five-month interval.

**Results**

The results revealed significant differences between in the variables studied when comparing the baseline and the second assessment. After five months of treatment there was a significant improvement in terms of psychopathological symptoms, including fewer depressive anxiety and stress symptoms. There was a decrease in negative affect, internal and external shame, as well as in aggressive and impulsive behaviours. An increase in positive affect was also found.

**Conclusions**

This is a novel study as it addresses and raises awareness of intervention with the Change & Grow Therapeutic Model. This programme shows promising results regarding psychopathological symptoms and in emotional states.

**Keywords**

Addiction disorders, change & grow, therapeutic community

#### O91 Economic impact of a nursing intervention program to promote self-management in COPD

##### Susana Cruz^1^, José M. Padilha^2^, Jorge Valente^3^

###### ^1^Administração Regional de Saúde do Norte, Porto, 4000-447, Portugal; ^2^Escola Superior de Enfermagem do Porto, Porto, 4200-072, Portugal; ^3^Faculdade de Economia, Universidade do Porto, 4200-464 Porto, Portugal

####### **Correspondence:** Susana Cruz (susanacruz.ermesinde@gmail.com) – Administração Regional de Saúde do Norte, Porto, 4000-447, Portugal

**Background**

Chronic obstructive pulmonary disease (COPD) is one of the main causes for morbidity and death worldwide. Maintaining life quality, autonomy and control over the disease involves the development of self-management skills. Objective: To determine the cost benefit of a nursing intervention program to promote self-management in COPD compared to a conventional treatment.

**Methods**

A quantitative and retrospective study was developed based on a post-factual documental analysis. A descriptive and exploratory analysis and a simulation to assess the robustness of results was performed. Between 2010-2013, 2,469 cases of COPD patients attending a Portuguese central hospital were analysed.

**Results**

This study evidenced that patients with more self-management skills have lower costs and fewer inpatient and emergency episodes. Although an increase in costs and number of nursing consultations is observed, these have minor impact in total care costs. Overall, the group under the intervention program registered a 67 % reduction in hospital costs between 2010-2013. The program is cost-beneficial for the health institution and has positive impact on the opportunity cost related to time saving while searching for health care provision services.

**Conclusions**

The nursing intervention programmes aimed at developing self-management skills show positive impact on patients’ health condition and on health care costs. An increase in advantages deriving from this long-term type of program is to be expected in the forthcoming years.

**Keywords**

COPD, self-management, costs, cost-effectiveness, cost-benefit, economic analysis

#### O92 Multimodal acute pain management during uterine artery embolization in treatment of uterine myomas

##### José T. Guerrero^1^, Francisco P. Caballero^2^, Rafael B. Santos^3^, Estefania P. Gonzalez^1^, Fátima M. Monago^4^, Lierni U. Ugalde^3^, Marta M. Vélez^1^, Maria J. Tena^2^

###### ^1^Complejo Hospitalario Universitario de Badajoz, Badajoz, 06080, España; ^2^Centro de Salud La Paz, Badajoz, 06011, España; ^3^Universidad de Extremadura, Badajoz, 06071, España; ^4^Centro de Salud San Fernando, Badajoz, 06006, España

####### **Correspondence:** José T. Guerrero (tenaguerrero@hotmail.com) – Complejo Hospitalario Universitario de Badajoz, Badajoz, 06080, España

**Background**

Selective uterine arterial embolization can be followed by pain, nausea, vomiting and stress-induced catabolism. Development of improved pain relief with multimodal analgesia and stress reduction by regional anaesthetic techniques has provided important possibilities for enhanced recovery. Objective: To evaluate the effectiveness of a multimodal analgesia protocol for the management of uterine artery embolization in women for the relief of acute pain.

**Methods**

Observational, prospective study of 40 patients who underwent selective uterine arterial embolization, with epidural catheter implanted and intravenous administration of analgesia for postoperative pain management. Pain intensity was measured by visual analogue scale (VAS) during the first 24-48 hours after procedure.

**Results**

The mean age of the patients was 39.1 ± 6.79 years; we used a regional anaesthetic technique with epidural anaesthesia or combined spinal and epidural anaesthesia for perioperative period in combination with Nonsteroidal anti-inflammatory drugs (NSAIDs) and paracetamol. 75 % of patients reported a VAS pain score below 3 point 24 hours after the procedure. 15 % of patients needed bolus administration of morphine and 10 % of patients needed a local anaesthetic bolus of lidocaine 2 % for an adequate pain control. The average length of stay in resuscitation area was 1.07 ± 0.26, with a VAS < 3 to discharge. Degree of patient’s satisfaction after selective uterine artery embolization was 7.8 ± 1.6.

**Conclusions**

Pain is a complex sensation that requires an interdisciplinary approach. Multimodal analgesia includes the association of analgesics and local anaesthetic administered by different routes to achieve effective relief. This approach increases patient satisfaction.

**Keywords**

Pain, analgesia, multimodal

#### O93 Fluid administration strategies in major surgery: Goal-directed therapy

##### José T. Guerrero^1^, Rafael Bravo^2^, Francisco L. Pérez-Caballero^3^, Isabel A. Becerra^1^, Mª Elizabeth Agudelo^1^, Guadalupe Acedo^1^, Roberto Bajo^1^

###### ^1^Department of Anestesiology, Complejo Hospitalario Universitario de Badajoz, 06080 Badajoz, España; ^2^Universidad de Extremadura, Badajoz, 06071, España; ^3^Centro de Salud La Paz, Badajoz, 06011, España

####### **Correspondence:** Rafael Bravo (rbravo@unex.es) – Universidad de Extremadura, Badajoz, 06071, España

**Background**

Inappropriate fluid management during major surgery may result in fluid overload or systemic hypoperfusion. Perioperative goal-directed therapy based on hemodynamic parameters in comparison with “liberal” fluid administration appears to have different effects on perioperative outcomes. Objective: We investigated whether individualized goal-directed therapy would decrease fluid administration and improve postoperative outcomes.

**Methods**

Patients undergoing elective intraabdominal surgery were randomly assigned to a control group (CG) with routine intraoperative care and goal-directed therapy fluid administration group (GDT group), where fluid management was guided by hemodynamic measures. The local ethics committee approved the study. A total of 60 patients were included in the study.

**Results**

Groups were similar with respect to demographics and baseline hemodynamic variables. The median duration of resuscitation area stay was significantly reduced in the GDT group (p < 0.05) and fewer patients developed complications than in the control group (p > 0.05). The median amount of intravenous fluids was significantly lower in the GDT group (p < 0.01). The total number of complications was reduced in the GDT group (p > 0.05). There were no significant differences in mortality between the groups.

**Conclusions**

In patients undergoing major abdominal surgery, implementation of intraoperative goal-directed hemodynamic strategies was associated with a reduced length of resuscitation area stay, with a reduced amount of intravenous fluids allowing us to avoid fluid overload and lower the incidence of complications compared to “liberal” fluid administration.

**Keywords**

Goal-directed therapy, fluid therapy, major surgery

#### O94 Development and implementation of a self-management educational programme using lay-led’s in adolescents Spina Bifida: A pilot study

##### Isabel Malheiro^1^, Filomena Gaspar^1^, Luísa Barros^2^

###### ^1^Escola Superior de Enfermagem de Lisboa, 1700-063 Lisboa, Portugal; ^2^Faculdade de Psicologia, Universidade de Lisboa, 1649-013 Lisboa, Portugal

####### **Correspondence:** Isabel Malheiro (mmalheiro@esel.pt) – Escola Superior de Enfermagem de Lisboa, 1700-063 Lisboa, Portugal

**Background**

Development challenges of an adolescent are based on the search for identity and parental independence. For adolescents with Spina Bifida (SB) these challenges become particularly hard to overcome. The development of functional skills in performing daily life activities and self-management are crucial to facilitate their transition to adulthood. The main purpose of this study was to develop an educational programme for self-management for adolescents with SB and evaluate its effect.

**Methods**

Fifty-six (56) adolescents with SB, aged 10 to 18 performed the programme in a Summer Camp environment, with a before and after programme evaluation and 6 months later. The Functional Independence Measure and Self-evaluation functionality scale were used as data collection instruments (quantitative approach) and 12 focus group interviews, 8 with the adolescents on the last day of the programme and 4 with their parents 6 months later (qualitative approach).

**Results**

The results revealed that the programme induced statistically significant differences in self-care, elimination, transfers and social cognition dimensions that persisted 6 months after the programme. Of the psychoeducational strategies used in the programme, they highlighted the problem solving technique, roleplaying, mentoring and modelling by lay-led programmes as those that most contributed to changes in the self-management behaviour of these adolescents.

**Conclusions**

The results suggest clear benefits in improving the functionality and the development of self-management skills and, suggest that the programme was highly effective. These results reinforce the importance of using psychoeducational strategies within the nursing intervention to promote mastery and the self-management competences in young people with SB.

**Keywords**

Adolescents, Spina Bifida, Nursing Intervention, Self-management Educational Program, Psychoeducational Strategies, Lay-Leds

#### O95 Influence of chair-based yoga exercises on salivary anti-microbial proteins in institutionalized frail-elderly women: a preliminary study

##### Guilherme Furtado^1,2^, Mateus Uba-Chupel^1,2^, Mariana Marques^1^, Luís Rama^1^, Margarida Braga^1,3^, José P. Ferreira^1^, Ana Mª Teixeira^1^

###### ^1^Centro de Investigação do Desporto e da Atividade Física, Faculdade de Ciências do Desporto e Educação Física, 3040-156 Coimbra, Portugal; ^2^Fundação Capes, Ministério da Educação, 70.040-020, Brasília, Distrito Federal, Brasil; ^3^Faculty of Medicine, University of Porto, Porto, 4200-450, Portugal

####### **Correspondence:** Guilherme Furtado (furts2001@yahoo.com.br) – Centro de Investigação do Desporto e da Atividade Física, Faculdade de Ciências do Desporto e Educação Física, 3040-156 Coimbra, Portugal

**Background**

Exercise has been reported as a way of attenuating the effects of immunosenescence. Reductions in salivary anti-microbial proteins (AMP) levels reflect reduced airway mucosal immune protection leading to upper respiratory tract infections. Yoga practice could be used as therapy, as it doesn’t require strenuous effort and it could be easily adaptable to the necessities of the frail elderly. Objective: With this study we propose to analyse whether chair-based yoga practice can be used to attenuate the effects of immunosenescence in pre-frail elderly women.

**Methods**

The final sample consisted of thirty-four elderly individuals (mean age 84.21 ± 6.9 years) that participated in the study and were divided into two groups: an exercise group (EG, n = 22) and a control group (CG, n = 14). All subjects were evaluated before and after the exercise program. The EG practised exercise 2-3 times per week for 14 weeks, and the CG was not involved in any exercise programmes. Saliva samples were collected before and after 28 weeks. Differences between moments were analysed using the Student t-test.

**Results**

According to the results there were no statistical significant differences in the EG individuals after the 14-week exercise program in the AMP concentrations, however the GC participants showed a decrement of IgA and lysozyme levels (p ≥ 0.05).

**Conclusions**

Our results revealed that chair-based yoga practice was able to maintain mucosal immune parameters in frail elderly women, possibly preventing a decrease in quality of life, and that it can be used as a therapy to attenuate the effects of immunosenescence.

**Keywords**

Elderly, Yoga, Immunology-system

#### O96 High intensity interval training vs moderate intensity continuous training impact on diabetes 2

##### João Cruz^1,2^, Tiago Barbosa^1^, Ângela Simões^1^, Luís Coelho^1,2^

###### ^1^School of Education and Social Sciences, Polytechnic Institute of Leiria, Leiria, 2411-901 Leiria, Portugal; ^2^Life Quality Research Centre – IPLeiria Branch, Polytechnic Institute of Leiria, Leiria, 2411-901 Leiria, Portugal

####### **Correspondence:** João Cruz (joaocruz@ipleiria.pt) – School of Education and Social Sciences, Polytechnic Institute of Leiria, Leiria, 2411-901 Leiria, Portugal

**Background**

Diabetes is a metabolic disease emerging as a threat to human health. Physical Activity is presented as a strategy for the treatment and control of this disease. Objectives: To compare the benefits of High Intensity Interval Training (HIIT) versus Moderate Intensity Continuous Training (MICT) on several diabetes markers, namely HbA1c.

**Methods**

The sample comprised 10 diabetic individuals, aged 61.7 ± 9.37 years old; with a body mass of 88.39 ± 2.54 kg; 163.7 ± 9.13 cm in height; a body fat mass of 29.84 ± 12.74 kg; lean mass of 55.63 ± 14.47 kg, visceral fat rating 14.4 ± 6.6 and a waist circumference of 109.8 ± 16.92 cm. Group 1 (HIIT, n = 5) performed a walk of 30 minutes at an intensity of HRR60-65 %, and Group 2 (MICT, n = 5) performed a 20-minute walk/run alternating 1 minute run at HRR70-75 % with 1 minute active recovery at HRR50-60 % for seven weeks, two times a week. Fibrinogen, HbA1c, total cholesterol, HDL, LDL, triglycerides, C-reactive protein, homocysteine, waist circumference, and VO_2_ max were determined for the two groups.

**Results**

No differences were found between the type of exercise performed between groups. HbA1c in MICT was significantly reduced (p = 0.043) in post versus pre-testing; Homocysteine and Body Fat Mass was also significantly reduced (p = 0.043) at the end of the HIIT protocol.

**Conclusions**

The type of exercise (HIIT vs. MICT) does not appear to induce different outcomes on markers of diabetes, namely on HbA1c.

**Keywords**

Quality of life, diabetes, metabolic disease, HbA1c, physical exercise

#### O97 Family caregiver of people with pressure ulcer: Nursing intervention plan

##### Alexandre Rodrigues^1^, Juan-Fernando Jiménez-Díaz^2^, Francisco Martinez-Hernandez^2^, Bienvenida Rodriguez-De-Vera^2^, Pedro Ferreira^3^, Alexandrina Rodrigues^4^

###### ^1^Universidade de Aveiro, 3810-193 Aveiro, Portugal; ^2^Universidad de Las Palmas de Gran Canaria, 35001 Las Palmas de Gran Canaria, España; ^3^Centro de Estudos e Investigação em Saúde, Faculdade de Economia, Universidade de Coimbra, 3004-512 Coimbra Portugal; ^4^Unidade de Cuidados na Comunidade da Ponte da Barca, 4980-620 Ponte da Barca, Portugal

####### **Correspondence:** Alexandre Rodrigues (rodriguesalexandr@gmail.com) – Universidade de Aveiro, 3810-193 Aveiro, Portugal

**Background**

Daily routines and the specific pressure ulcer (PU) care can cause different needs for the caregiver. This follows results of a study (2015) where the main needs were identified of family caregivers of people with pressure ulcers. In this work the nursing interventions for each need are defined. Objective: To define the nursing interventions associated with the needs of caregivers of people with pressure ulcers.

**Methods**

A qualitative study. Questionnaire with open questions to twelve domiciliary care nurses. Speech analyses with WEBQDA program. All ethical aspects checked.

**Results**

For difficulties in positioning, the nursing interventions are: teach, instruct, demonstrate, train, assist and supervise positioning, provide materials and literary support. When they refuse to contact with PU, it is important to know the reasons, to explain the importance of the treatment, to positively strengthen their performance and ascertain if a secondary caregiver exists with competences to give treatment. The low level of quality of life and the high level of burden require health and social support, the use of scales for assessment, providing a secondary caregiver and defining strategies to rest. When caregivers need to be recognised, nurses may strengthen their importance and encourage them to have time to themselves.

**Conclusions**

Main interventions are integrated into the autonomous nursing area. To obtain health gains for the caregiver, nursing interventions may be direct not only to the caregiver, but also with best care for the patient and to find health and social resources for both.

**Keywords**

Caregiver, Pressure ulcer, Nursing care

#### O98 Chronic effects of exercise on motor memory consolidation in elderly people

##### André Ramalho^1^, João Petrica^1,2^, Pedro Mendes^1^, João Serrano^1,2^, Inês Santo^3^, António Rosado^4^

###### ^1^Escola Superior de Educação, Instituto Politécnico de Castelo Branco, 6000-767 Castelo Branco, Portugal; ^2^Centro de Estudos em Educação, Tecnologias e Saúde, Escola Superior de Saúde, Instituto Politécnico de Viseu, 3504-510 Viseu, Portugal; ^3^Studio Osteopatico Franchi, 6512 Giubiasco, Switzerland; ^4^Faculty of Human Kinetics, University of Lisbon, 1649-028 Lisboa, Portugal

####### **Correspondence:** André Ramalho (andreramalho361@gmail.com) – Escola Superior de Educação, Instituto Politécnico de Castelo Branco, 6000-767 Castelo Branco, Portugal

Recent studies have shown that a single bout of aerobic exercise improves motor memory consolidation. However, few studies have explored the chronic effects of exercise on motor memory consolidation in elderly people. The main purpose of this study was to investigate if a six-month period of physical exercise could improve motor memory consolidation in elderly people.

Thirty-eight (38) subjects of both genders, with a mean age of 71 years old participated in the study. Subjects were divided into two groups: a control group and an experimental group. Before the intervention of a physical exercise program, subjects performed a Finger Tapping Sequence to measure baseline performance. After the intervention, the assessment of the impact of exercise on motor memory consolidation was held in three stages: Training, 1 hour after training and 24 hours after training. For the analysis of the results, descriptive and inferential statistics were used.

The Shapiro-Wilk test was applied to the normality distribution of data and one-way ANOVA test for parametric statistics. When compared with the control group, results showed a better performance in motor memory consolidation 1 hour and 24 hours after training in the experimental group. Nevertheless, these differences were not significant (p ≥ 0.05). It seems therefore that regular physical exercise does not significantly improve motor memory consolidation capacity in elderly people.

ClinicalTrials.gov Identifier: NCT02731261.

**Keywords**

Physical Exercise, Motor Memory Consolidation, Finger Tapping Sequence, Learning, Aging

#### O99 Impression cytology of the ocular surface: Collection technique and sample processing

##### Paula Mendonça, Kátia Freitas

###### Escola Superior de Tecnologia da Saúde de Lisboa, Instituto Politécnico de Lisboa, 1549-020 Lisboa, Portugal

####### **Correspondence:** Paula Mendonça (paula.mendonca@estesl.ipl.pt) – Escola Superior de Tecnologia da Saúde de Lisboa, Instituto Politécnico de Lisboa, 1549-020 Lisboa, Portugal

**Background**

The impression cytology of the ocular surface is a minimally invasive technique that allows the analysis of conjunctival and corneal cells, as an alternative to smears, biopsies and punches. Objective: The aim of this study was to present a technique of collecting, fixing and staining for impression cytology of ocular specimens.

**Methods**

Fifty (50) samples of bulbar conjunctiva taken from 50 ESTeSL volunteers were analysed. The material was collected on a strip of cellulose acetate from Millipore, then fixed and stained with Papanicolaou stain. The slides were analysed by three independent evaluators, using an evaluation grid with the following parameters: cell size, detail and nuclear membrane, detail and cytoplasmic membrane, ratio N/C and tinctorial affinity. The final score per slide was obtained through the sum of all considered parameters (scale 0 to 41).

**Results**

The filter paper with apex helped to correctly position the paper in the eye and the procedure that was applied allowed an effective collection of cells with 50-70 % of the filter surface being filled without the need for topical anaesthesia. The SureThin fixative presented quality in cell preservation, in addition to being more economical. The Papanicolaou staining technique proved to be ideal in the colouring of ocular epithelial cells. This developed methodology presented a maximum score of 41 values in a 74 % response.

**Conclusions**

The presented method proved to be very effective in evaluating of ocular cell samples, while simultaneously proving to be a very cheap and comfortable technique for the patient.

**Keywords**

Impression cytology, Collection method, Fixation, SureThin, Papanicolaou staining

#### O100 Does sport practice affect the reaction time in neuromuscular activity?

##### Dora Ferreira, António Brito, Renato Fernandes

###### Escola Superior Desporto De Rio Maior, Instituto Politécnico de Santarém, 2040-413 Rio Maior, Portugal

####### **Correspondence:** Dora Ferreira (dferreira@esdrm.ipsantarem.pt) – Escola Superior Desporto De Rio Maior, Instituto Politécnico de Santarém, 2040-413 Rio Maior, Portugal

Nowadays the regular practice of sport is an important way to preserve the population’s health. One of the benefits of practising sport is related to the maintenance and improvement of neuromuscular functions, especially the capacity to react to external stimuli and to promote movement. The present study aimed to identify the effects of training on Neuromuscular Reaction Time (NRT) in karatekas and non-karate athletes with various ages and level of expertise.

NRT was considered as the time between the application of the auditory stimulus and the onset of electrical activation of the first muscle activated in the inferior limb to perform a front kick against a fixed target (Mae-geri). The data were collected with surface electromyography. The NRT was measured, analysed and compared in n = 46 participants divided into four groups.

The results revealed that the first muscle to be activated in all groups was the Tibialis Anterior (TA). However, there were no significant differences between groups respecting NRT of this muscle. Nevertheless, significant differences were found between groups in NRT in the Biceps Femoris (BF) and Gastrocnemius (GA) muscles. The results show a differentiated neuromuscular time of recruitment in response to the stimulus in some muscles, which could be associated with the benefits of the sport practice on neuromuscular activity. We may assume that the individuals that keep active in daily routine life skills probably don’t decline at the cognitive process level. They change and adapt due to the natural aging process.

**Keywords**

Reaction Time, Neuromuscular, Karate

#### O101 Efficiency of the enteral administration of fibbers in the treatment of chronic obstipation

##### Sofia Gomes^1^, Fernando Moreira^1,2^, Cláudia Pinho^1,3^, Rita Oliveira^1,3,4^, Ana I. Oliveira^1,3^

###### ^1^Escola Superior de Tecnologia da Saúde do Porto, Instituto Politécnico do Porto, Vila Nova de Gaia, 4400-330, Portugal; ^2^Department of Legal Medicine and Forensic Sciences, Faculty of Medicine, University of Porto, 4200-319 Porto, Portugal & Pharmaceutical Services, Centro Hospitalar de Vila Nova de Gaia/Espinho, EPE, Vila Nova de Gaia, 4400-129, Portugal; ^3^Núcleo de Investigação e Intervenção em Farmácia, Centro de Investigação em Saúde e Ambiente, Instituto Politécnico do Porto, Vila Nova de Gaia, 4400-330, Portugal; ^4^Secção Autónoma de Ciências da Saúde, Universidade de Aveiro, 3810-193 Aveiro, Portugal

####### **Correspondence:** Sofia Gomes (sofiasilvagomes93@gmail.com) – Escola Superior de Tecnologia da Saúde do Porto, Instituto Politécnico do Porto, Vila Nova de Gaia, 4400-330, Portugal

It is estimated that constipation affects 20 % of the population in western countries, leading to a significant impact on people’s quality of life. The administration of some types of fibres has significantly improved the symptoms of constipation over 4-week periods of administration, apparently increasing the frequency of defecation and having a protective effect on the intestinal flora. In addition to their effect on this pathology, fibres have been linked to beneficial outcomes in cardiovascular diseases, diabetes, obesity, colorectal cancer and haemorrhoids. This study focuses on the short-term evaluation of the benefits of fibres on intestinal motility in constipated patients.

A quasi-experimental pilot study was performed, consisting of the administration of 6.3 g of a blend supplement of six soluble and insoluble fibres (containing soy polysaccharide, cellulose, resistant starch, gum arabic, oligofructose and inulin) both at lunch and dinner, for 15 days. The sample included 5 patients diagnosed with chronic constipation who frequently use laxatives, aged between 50 and 83, and who did not require a restricted-fibre diet.

Participants obtained stools of type 3 and 4, according to the Bristol stool form scale. The average daily bowel evacuations decreased and all participants ended up taking laxatives after a short initial stop. The supplementation with the fibre-enriched product did not cause any adverse symptoms.

According to this study, the supplementation of chronically constipated patients with fibres is ineffective in the reduction of obstipation, over a short-term period of 15 days. Besides, the dependence generated by the use of laxatives is clearly demonstrated.

**Keywords**

Supplement, inulin, oligosaccharides, bowel, prebiotics

#### O102 Fast decalcifier in compact bone and spongy bone

##### Paula Mendonça^1^, Ana P. Casimiro^2^, Patrícia Martins^1^, Iryna Silva^3^

###### ^1^Escola Superior de Tecnologia da Saúde de Lisboa, Instituto Politécnico de Lisboa, 1549-020 Lisboa, Portugal; ^2^Hospital SAMS – Serviços de Assistência Médico-social do Sindicato dos Bancários, 1849-017 Lisboa, Portugal; ^3^North Tyneside General Hospital, Tyne and Wear NE29 8NH, Newcastle, UK

####### **Correspondence:** Paula Mendonça (paula.mendonca@estesl.ipl.pt) – Escola Superior de Tecnologia da Saúde de Lisboa, Instituto Politécnico de Lisboa, 1549-020 Lisboa, Portugal

**Background**

This study evaluates the effectiveness of the decalcification process, comparing water decalcifiers – Nitric Acid 10 %, for compact bone, and Surgipath® Decalcifier® II (SD® II), for spongy bone – and the new commercial formula, Fast Decalcifier, for both types. Objective: To compare the effectiveness of decalcifiers through the study of morphology, preservation and quality of bone biological samples.

**Methods**

One hundred and forty (140) blocks of histological samples of compact and spongy bone of swine, previously fixed in 10 % formalin and impregnated in paraffin were used. The decalcification process was evaluated for compact bone by weighing after 24 hours, cutting with a scalpel and by sound, while the spongy bone was evaluated every hour within 24 hours through the evaluation by palpation with forceps. The morphology preservation was evaluated by Hematoxylin-Eosin staining and presence of calcium was checked by Von Kossa and Alizarin Red S staining.

**Results**

The one-way ANOVA test indicates that were no statistically significant differences compared with Fast Decalcifier (p-value > α = 0.05) with SD® II for spongy bones. The Fast Decalcifier bone did not reveal good results in compact bone which means that the decalcifier is aggressive and doesn’t preserve the tissue architecture in the cases with a high calcium level.

**Conclusions**

The new commercial formula, Fast Decalcifier showed a better performance in the removal of calcium, in the preservation of morphology and an adequate time for performance, proving to be the best decalcifier for spongy bone compared with SD® II.

**Keywords**

Nitric acid 10 %, Fast decalcifier, Compact bone, Spongy bone

#### O103 Prevention of musculoskeletal disorders through an exercise protocol held in labour context

##### Catarina Leitão^1^, Fábia Velosa^2^, Nélio Carecho^3^, Luís Coelho^1,2^

###### ^1^Life Quality Research Centre, Instituto Politécnico de Castelo Branco, 6000-767 Castelo Branco, Portugal; ^2^School of Education and Social Sciences, Polytechnic Institute of Leiria, 2411-901 Leiria, Portugal; ^3^Universidade Federal de Santa Catarina, Florianópolis, Santa Catarina, 88040-900, Brasil

####### **Correspondence:** Catarina Leitão (catarinaleitao.pt@gmail.com) – Life Quality Research Centre, Instituto Politécnico de Castelo Branco, 6000-767 Castelo Branco, Portugal

Musculoskeletal disorders (LME) are well recognized as work related diseases (MSDs). Physical Activity can play an important role in preventing MSDs and improving pain symptoms related to labour activity.

This research aimed to analyse the influence of a 10 week (bi-weekly) exercise program (gymnastics and fitness) held in labour context, on the reduction of musculoskeletal symptoms and well-being of factory workers.

An exploratory longitudinal study design was conducted using a sample of 20 factory workers. The Nordic Questionnaire for musculoskeletal symptoms and physical tests of strength and flexibility were used to collect data in two different moments, T0 (before the intervention) and T1 (after the intervention). There was a decrease of pain complaints (25 % in the cervical region, 20.8 % in the shoulders, elbows 16.7 %, 8.4 % wrists, 12.5 % in the lumbar region, 20.8 % in the knees and 8.3 % over the ankles/feet) and an increased sense of well-being (feeling optimistic = 58.3 % and feel active = 66.7 %). There were also statistically significant improvements in flexibility, respectively in the trunk flexion (p = 0.000) and in the Reaching the Left Upper Limb test (p = 0.000).

Exercise has become an essential element in promoting health and improving quality of life, reducing the risk of developing chronic diseases.

**Keywords**

Exercise protocol, musmusculoskeletal disorders, musmusculoskeletal disorders prevention

#### O104 Knowledge of teachers and other education agents on diabetes type 1: Effectiveness of an intervention program

##### Eva Menino^1^, Anjos Dixe^1^, Helena Catarino^1^, Fátima Soares^2^, Ester Gama^3^, Clementina Gordo^1^

###### ^1^Health Research Unit & School of Health Sciences, Polytechnic institute of Leiria, 2411-901 Leiria, Portugal; ^2^Unidade de Saúde Pública, ACES Pinhal Litoral, ARS Centro, 3100-462 Pombal, Portugal; ^3^Hospital de Santo André, Centro Hospitalar de Leiria, 2410-197 Leiria, Portugal

####### **Correspondence:** Eva Menino (eva.guilherme@ipleiria.pt) – Health Research Unit & School of Health Sciences, Polytechnic institute of Leiria, 2411-901 Leiria, Portugal

**Background**

Type 1 diabetes is one of the most prevalent chronic diseases in school age, needing therefore knowledgeable staff in order to assure a safe school environment. It is recommended that the school health nurse should be the professional to plan, prepare and ensure (coordinate) training to school staff (ADA, 2014) [1]. Objectives: To evaluate the effectiveness of an intervention program focused on the development of knowledge of education staff on type 1 diabetes.

**Methods**

This is a quasi-experimental study, with a pre-post intervention design with no control group, which included 81 educators. It was implemented an intervention program by a school nurse with theoretical and practical components. Before and after intervention, it was applied a questionnaire with socio-economic data and the Portuguese version of the “Diabetes knowledge Questionnaire” scale [2] about type 1 diabetes.

**Results**

The knowledge improved with the intervention. Total score (ranging from 0 -17) obtained before intervention was 11.10 ± 2.69 and after this average rose to 13.84 ± 1.87, having a statistically significant difference (p < 0.001). Less knowledge was identified in healthy eating and monitoring blood glucose, before intervention. After intervention, worst scores were identified in the detection of hypoglycemia signals and in intervention during hypoglycemia.

**Conclusions**

Knowledge is a basic condition for monitoring and for a correct intervention in type 1 diabetes complications, such as severe hypoglycemia. It is, therefore, necessary to develop effective intervention programs towards the acquisition of knowledge by the school staff that supervise students with diabetes.

**References**

1. American Diabetes Association. Diabetes care in the school and day care setting. Diabetes care, 2012; 35. Supplement 1: S76-S80.

2. Menino E, Roque S, Dixe M. Cross-cultural adaptation of the "Test of Diabetes Knowledge for Teachers". 2015.

**Keywords**

Type 1 diabetes mellitus, nursing, knowledge, health school

#### O105 Treatment of diabetic peripheral neuropathic pain: a systematic review of clinical trials of phase II and III

##### Eliana Moreira, Cristiana Midões, Marlene Santos

###### Escola Superior de Tecnologia da Saúde, Instituto Politécnico do Porto, 4400-330 Vila Nova de Gaia, Portugal

####### **Correspondence:** Marlene Santos (mes@estsp.ipp.pt) – Escola Superior de Tecnologia da Saúde, Instituto Politécnico do Porto, 4400-330 Vila Nova de Gaia, Portugal

Diabetic Peripheral Neuropathic Pain (DPNP) is a common complication of diabetes, which affects a wide fraction of patients, and it’s likely to increase due to the prevalence of Diabetes. Diabetic neuropathy is a complication associated with patient age, illness course and hyperglycaemia severity. DPNP pharmacologic treatment includes antidepressants, anticonvulsants, and opioid drugs, among others. However, and given the different pharmacologic options, pain relief is currently unsatisfactory in most of the cases.

The aim of this study was to systematically review randomized controlled trials of oral and topical pharmacotherapy for DPNP, including studies published in peer-reviewed journals in PUBMED and unpublished trials retrieved from ClinicalTrials.gov, reporting predefined efficacy and safety outcomes, published from 2010 on. Participants in these trials included people with diabetes mellitus and diabetic peripheral neuropathy who were given any treatment for diabetic peripheral neuropathy. Data from the trials were reviewed by two authors independently, and extracted using standardized data extraction sheets.

From the 29 selected trials, 11 were elected after exclusion criteria were applied; these trials included drugs with different pharmacotherapeutic profiles. Significant improvement of pain was only reported for two trials: one with a serotonin and norepinephrine reuptake inhibitor and other with a cholinergic agonist of the nicotinic receptors. Both these drugs present statistically significant differences in the intensity of the pain when compared to placebo.

Further investigation in required to fully explain the mechanisms of the nerve injury in in order to develop a target therapy for DPNP, with more efficient pain control.

**Keywords**

neuropathic pain, diabetic peripheral neuropathic pain, diabetes mellitus, drug, treatment

#### O106 New drugs for osteoporosis treatment: Systematic review of clinical trials of phase II and III

##### Sara Machado, Vânia P. Oliveira, Marlene Santos

###### Escola Superior de Tecnologia da Saúde, Instituto Politécnico do Porto, 4400-330 Vila Nova de Gaia, Portugal

####### **Correspondence:** Marlene Santos (mes@estsp.ipp.pt) – Escola Superior de Tecnologia da Saúde, Instituto Politécnico do Porto, 4400-330 Vila Nova de Gaia, Portugal

Osteoporosis is a disease characterized by decreased bone mass and deterioration of bone microarchitecture. It is a highly prevalent condition in western countries, and it is estimated to affect 5 % of the Portuguese population. Advances in the research in the pathophysiology of the disease have been used for the development of new molecules. In addition, several clinical trials have been developed in order to determine the effectiveness of new drugs to treat osteoporosis.

The aim of this study was to systematically review randomized controlled trials of oral drugs for osteoporosis treatment. The systematic review was performed in electronic databases, particularly at ClinicalTrials (www.clinicaltrials.gov) and PubMed (http://www.ncbi.nlm.nih.gov/pubmed), of which were selected only clinical trials of phase II and III, in patients of female gender, published in the last five years, which addressed any drug for the treatment of osteoporosis.

After inclusion and exclusion criteria, of the 132 studies, 34 were selected to be included in the systematic review. These trials included drugs of different pharmacologic profiles. Regarding drugs with anabolic effects, molecules which stimulate bone formation, clinical trials show efficacy for Romosozumab and BA 058, while for MK-5442 the results were unsatisfactory. Regarding bone antiresorptive drugs, Denosumab was reported in several studies to have high efficacy. Other further promising drugs included ONO-5334 and Odanacatib, which showed positive results in several clinical trials.

Despite the wide diversity of drugs under clinical trials, new pharmacological options should be developed to revert bone loss in this highly prevalent chronic disease.

**Keywords**

Osteoporosis, drugs, clinical trials, bone mineral density

#### O107 Promoting hope at the end of life: Effectiveness of an Intervention Programme

##### Ana Querido^1^, Anjos Dixe^1^, Rita Marques^1,2^, Zaida Charepe^3^

###### ^1^Health Research Unit, School of Health Sciences, Polytechnic institute of Leiria, 2411-901 Leiria, Portugal; ^2^Hospital de Santa Maria, Centro Hospitalar Lisboa Norte, 1649-035 Lisboa, Portugal; ^3^Universidade Católica Portuguesa, Lisboa, 1649-023, Portugal

####### **Correspondence:** Ana Querido (ana.querido@ipleiria.pt) – Health Research Unit, School of Health Sciences, Polytechnic institute of Leiria, 2411-901 Leiria, Portugal

**Background**

Hope is essential to life and a coping strategy for people with advanced chronic illness facing the end of life. Objectives: Test the effect of an intervention Programme to Promote Hope (PPH) on hope, quality of life (QOL) and comfort of people in palliative situation.

**Methods**

Fifty-six (56) palliative patients were randomly assigned to an intervention group (n = 28) and a control group (n = 28). The intervention group was provided with 3 PPE sessions (1 hour) for one week, while the control group were submitted to the regular treatment. All patients were assessed with the Portuguese versions of the Herth Hope Index, McGill Quality of Life Questionnaire and Holistic Comfort Questionnaire at pre-test/post-test, at one month follow up. The dropouts resulted in n = 11 in intervention group and 12 in control group. Results were analysed using non-parametric statistics.

**Results**

Patients experienced good levels of hope, QOL and comfort. There was no significant difference at the pre-test of outcome measures (p > 0.05). After the intervention significant differences existed between the 2 groups in Hope and QOL, but no differences in comfort levels. Follow-up hope levels decreased with significant differences between the groups (p < 0.05). Differences in QOL were not found. Comfort levels were significantly different in follow up, with higher levels of comfort experienced by the intervention group.

**Conclusions**

A structural hope intervention programme in palliative patients in a community setting had a positive effect on improving hope, quality of life and comfort. More studies need to be developed to test the intervention on a bigger sample.

**Keywords**

Hope, quality of life, comfort, end of life, intervention program

#### P43 Psychomotor therapy effects on adaptive behaviour and motor proficiency of adults with intellectual disability

##### Ana Antunes^1^, Sofia Santos^2^

###### ^1^Associação Portuguesa de Pais e Amigos do Cidadão Deficiente Mental, 6200-050 Covilhã, Portugal; ^2^Faculdade de Motricidade Humana, Universidade de Lisboa, Cruz quebrada, 1499-002 Lisboa, Portugal

####### **Correspondence:** Sofia Santos (sofiasantos@fmh.ulisboa.pt) – Associação Portuguesa de Pais e Amigos do Cidadão Deficiente Mental, 6200-050 Covilhã, Portugal

Persons with intellectual disabilities (ID) usually present limitations in adaptive behaviour and motor skills, which constitute a barrier to their daily independent functioning. Our goal was to analyse the efficacy of a Psychomotor Intervention (PMT) programme on adults with ID, in terms of adaptive behaviour and motor proficiency skills.

The sample comprised 15 participants divided into 3 groups of 5 individuals each (A = ID, B = Down Syndrome and C = typical development). Ages ranged from 22 to 31 years old (26.5 ± 3.76), 6 were females and 9 males. All adults with ID were institutionalized and adults without ID were selected by convenience. Portuguese versions of the Adaptive Behaviour Scale [1] and of the Bruininks-Oseretsky Motor Proficiency Test were applied [2]. After establishing a baseline, a 3-month PMT Programme was designed for participants with ID, focused on balance, global/fine motor skills, economic/vocational activities and academic/verbal skills. A Kruskall-Wallis test comparing pre-, post- and retention test scores of all groups was computed and to compare intra-groups differences a Wilcoxon test was performed.

Results showed statistically significant differences between groups with and without ID, with the latter presenting higher scores after the PMT program. Assessments showed great improvements in motor and adaptive skills, by the participants with ID.

Findings may indicate that PMT could strengthen and improve the areas assessed, although these findings should be interpreted with caution, due to the reduced sample.

**References**

1. Santos S, Morato, P. Comportamento Adaptativo – 10 anos depois. Lisboa: Edições FMH; 2012.

2. Bruininks R, Bruininks B. PMBO 2 Bruininks-Oseretsky Test of Motor Proficiency 2nd edition. San Antonio, USA: Pearson Assessments; 2005.

**Keywords**

Adaptive behaviour, motor proficiency, intellectual and developmental disabilities, psychomotor Intervention

#### P44 The effect of exercise therapy in multiple sclerosis – a single study case

##### Marlene C. Rosa (marlenerosa@ua.pt)

###### Secção Autónoma das Ciências da Saúde, Universidade de Aveiro, Aveiro, 3810-193, Portugal

**Background**

Multiple sclerosis (MS) is a chronic, often disabling disease of the central nervous system. Disability in MS is related with decline in general functioning (e.g., mobility, gait, balance). The exercise therapy is known to reduce the rate of functional decline in other diseases; however, its effect in the treatment of MS remains relatively unexplored. Objective**:** To determine the effect of exercise therapy in MS.

**Methods**

One patient with MS was included in a 6-week exercise program (2 sessions/week: progressive muscle strength, balance and aerobic exercises) conducted by a physiotherapist. Anthropometric (height; weight) and sociodemographic (age; gender) data, stage of disease (Abbreviated Expanded Disability status scale - AED) and functional tests (5-meter walking test at comfortable speed– 5mWT; Functional Reach test - FRT; 5 times sit-to-stand test – 5STS) were collected at the beginning (T0) and at the end (T1) of the exercise program. Averages of two trials for each functional test were compared between T0 and T1 and the remaining functional impairment was calculated as a percentage of normative aged adjusted values (%).

**Results**

A female (52 years; 48 kg; 1.59 m) in a stage 4 of AED scale (ambulatory without aid 12 h) participated in this study. From T0 to T1 the participant increased the performance in: 5mWT (0.51 m/s ± 0.08 vs 0.64 m/s ± 0.01; 36.69 % vs 46.04 %); FRT (20.5 cm ± 2.12 vs 25 cm ± 0.71; 47.99 % vs 58.53 %); 5STS (19.45 s ±1.62 vs 16.55 s ± 2.47; 36.5 % vs 42.9 %).

**Conclusions**

The exercise therapy improved general functioning in one patient with MS, getting functional scores close to/above 50 % of the expected value.

**Keywords**

Exercise, therapy, multiple sclerosis

#### P45 Physical condition and self-efficacy in people with fall risk – a preliminary study

##### Marlene C. Rosa^1^, Silvana F. Marques^2^

###### ^1^Secção Autónoma das Ciências da Saúde, Universidade de Aveiro, 3810-193 Aveiro, Portugal; ^2^Unidade de Cuidados na Comunidade, Centro de Saúde de Anadia, 3780-780 Anadia, Portugal

####### **Correspondence:** Marlene C. Rosa (marlenerosa@ua.pt) – Secção Autónoma das Ciências da Saúde, Universidade de Aveiro, 3810-193 Aveiro, Portugal

**Background**

In people with fall risk, the perceived inefficiency in dealing with falls contributes greatly to restrictions in movement and therefore might interfere with physical condition. However, this relationship has been relatively unexplored. Objective: To explore the association between physical condition and self-efficacy in people with fall risk.

**Methods**

People older than 50 years old with moderate fall risk (Morse scale) were included in a fall prevention exercise programme. Age, gender and Body Mass Index (BMI) were collected. At the end of the programme, self-efficacy (SE) was assessed, the physical condition was characterized (% of normative aged adjusted values - % N) and correlations between them were explored (Spearman test, p < 0.05). Four questions (scoring: 0-not confident – 2-very confident) were used to assess self-efficacy in: moving indoors; moving outside; walking on uneven surfaces; and participating in physical activities. Physical condition was assessed considering: (I) mobility (5 metres walking test at comfortable speed – 5mWT), (II) balance (Functional Reach Test – FRT) and (III) endurance (Sit-to-stand test – STS) tests.

**Results**

Six females and one male (66.43 ± 11.70 years; 42.56 ± 4.24 kg/m^2^) were included. At the end of the programme patients presented: better self-efficacy in moving indoors (1.33 ± 0.81) and in participation in physical activities (1.17 ± 0.75) items; walking speed at 0.66 ± 0.17 m/s (51.10%N); 23.78 ± 4.65 cm in FRT (66.07%N); 14.70 ± 2.04 sec in STS (58.97%N). Correlations were only significant between the STS results and the total SE score (r = 0.87; p = 0.01).

**Conclusions**

High endurance capacity seems to be determinant for the improvement of self-efficacy in people with fall risk.

**Trial registration**

NCT02746835.

**Keywords**

Physical condition, self-efficacy, fall risk

#### P46 Shock waves: their effectiveness in improving the symptoms of calcifying tendinitis of the shoulder

##### Beatriz Minghelli^1^, Eulália Caro^1,2^

###### ^1^Research in Education and Community Intervention, Escola Superior de Saúde Jean Piaget, Silves, 8300-025, Portugal; ^2^Fisiatris - Recuperação Física, Lda, Sacavém, 2685-010, Portugal

####### **Correspondence:** Beatriz Minghelli (beatriz.minghelli@silves.ipiaget.pt) – Research in Education and Community Intervention, Escola Superior de Saúde Jean Piaget, Silves, 8300-025, Portugal

**Background**

This paper intends to assess the effectiveness of shock waves in improving shoulder calcifying tendinitis symptoms.

**Methods**

The sample consisted of 6 patients diagnosed with calcific tendinitis of the shoulder, aged between 38 and 70 years of age (55.00 ± 12.93 years), 4 (66.7 %) females. The shoulder’s range of motion (with goniometer), muscle strength (Muscle Testing), pain (Numerical Pain Scale) and shoulder functionality (Disabilities of the Arm, Shoulder and Hand (DASH) scale) were evaluated. Five shock wave sessions were conducted.

**Results**

The start and end values of the motion range were: flexion – 156.67 ± 8.16 and 169.17 ± 5.84 (p = 0.020); extension – 31.67 ± 2.58 and 57.50 ± 42.98 (p = 0.026); abduction – 141.67 ± 28.57 and 160.00 ± 20.00 (p = 0.012); internal rotation – 44.17 ± 10.21 and 65.00 ± 5.48 (p = 0.001); external rotation – 73.33 ± 12.11 and 82.50 ± 12.55 (p = 0.020); horizontal adduction – 65.83 ± 13.57 and 80.00 ± 10.95 (p = 0.03) and horizontal abduction – 29.17 ± 8.01 and 37.50 ± 4.18 (p = 0.105). As regards the Muscle Testing, the initial value was 3.00 ± 0.00 and the final value was 3.83 ± 0.41 (p = 0.025) for all movements. The initial and final values of pain evaluations were, respectively: 6.67 ± 0.82 and 3.17 ± 1.17 (p < 0.001). The initial and final values of DASH were 49.31 ± 15.24 and 20.00 ± 10.13 (p = 0.005).

**Conclusions**

It was found that shock wave therapy improved symptoms in calcific tendonitis patients.

**Acknowledgements**

Authors wish to acknowledge the financial support of the Portuguese Foundation for Science and Technology (FCT), and of the programme COMPETE - Programa Operacional Factores de Competitividade, QREN – Quadro de Referência Estratégico Nacional.

**Keywords**

Shock Waves, calcifying tendinitis, shoulder

#### P47 Pacifier – construction and pilot application of a parenting intervention for parents of babies until six months in primary health care

##### Mª José Luís, Teresa Brandão

###### Faculdade de Motricidade Humana, Universidade de Lisboa, Cruz quebrada, 1499-002 Lisboa, Portugal

####### **Correspondence:** Mª José Luís (mariaa.pluis@gmail.com) – Faculdade de Motricidade Humana, Universidade de Lisboa, Cruz quebrada, 1499-002 Lisboa, Portugal

This work had as its main objective the construction and pilot testing of a preventive and universal parenting programme with ten sessions designated as - Pacifier - for implementation in primary health care, with parents of children aged 0 to 6 months based on a home-visiting format.

Conceptual foundation and design was based on literature review and evidence-based practices related to positive parenting and parental education programmes. In order to explore its applicability and impact we used a qualitative approach based on multiple case studies. Four mothers and two fathers – two couples and two mothers – living in the Castelo de Vide region agreed to participate.

These four families showed great satisfaction with the programme and would recommend it to other families. Parents’ sense of competence also shows improvements for most parents. This intervention seems to have good potential for increasing parents’ level of information and awareness about child development. It needs further analysis and could be an interesting instrument for improving parents’ knowledge and competence in primary health care.

**Keywords**

Parenting Intervention, Primary Health Care, Pacifier

#### P48 The influence of Motor Imagery in fine motor skills of individuals with disabilities

##### Pedro Mendes^1^, Daniel Marinho^2^, João Petrica^1,3^, Diogo Monteiro^4^, Rui Paulo^1,5^, João Serrano^1,3^, Inês Santo^6^

###### ^1^Escola Superior de Educação, Instituto Politécnico de Castelo Branco, 6000-767 Castelo Branco, Portugal; ^2^Research Centre of Sports Sciences, Health Sciences and Human Development, Universidade da Beira Interior, Covilhã, 6201-001 Covilhã, Portugal; ^3^Centro de Estudos em Educação, Tecnologias e Saúde, Escola Superior de Saúde, Instituto Politécnico de Viseu, 3504-510 Viseu, Portugal; ^4^Escola Superior de Desporto de Rio Maior, Instituto Politécnico de Santarém, 2040-413 Rio Maior, Portugal; ^5^Research, Education and Community Intervention, Instituto Piaget, 3515-776 Galifonge, Viseu, Portugal; ^6^Studio Osteopatico Franchi, 6512 Giubiasco, Switzerland

####### **Correspondence:** Pedro Mendes (pedromendes@ipcb.pt) – Escola Superior de Educação, Instituto Politécnico de Castelo Branco, 6000-767 Castelo Branco, Portugal

Imagery is a cognitive process that can play an important role on planning and executing different movements or actions. The main purpose of this study was to ascertain whether the application of Motor Imagery together with normal practice improved fine motor skills in disabled individuals.

Forty-two (42) subjects with disabilities participated in this study, with a mean age of 37.0 (SD = 12.0), and of both genders. Subjects were randomly divided in two groups: a control group and an experimental one. The study procedures were applied on five different tasks of the Psychomotor Battery of fine motor skills (BPM). This instrument was applied on two stages, at the beginning of the study (pre-test) and at the end of 4 weeks (post-test). Both groups performed the tasks twice a week for a month.

Motor imagery sessions were added on in the experimental group. Participants on the experimental group were asked to mentally imagine themselves recreating tasks they had performed earlier on the initial assessment. For the analysis of the results, descriptive and inferential statistics were used. The T-test for independent samples, and the T-test for paired samples were applied.

The results show a significant difference in all 5 tasks performed by the experimental group (p ≤ 0.05) thereby, the subjects in this group improved their fine motor skills. The control group however, just had statistical differences in two tasks. These results, shows that motor imagery, when used together with physical repetition of the movement imagined, can improve fine motor skills in disabled individuals.

ClinicalTrials.gov Identifier: NCT02725450.

**Keywords**

Imagery, motor imagery, fine motor skills, disabled

#### P49 Evaluation of the effects of a walking programme on the fall risk factors in older people – a longitudinal pilot study

##### Lina Monteiro^1^, Fátima Ramalho^1,2^, Rita Santos-Rocha^1,2^, Sónia Morgado^1,3^, Teresa Bento^1,4^

###### ^1^Escola Superior de Desporto de Rio Maior, Instituto Politécnico de Santarém, 2040-413 Rio Maior, Portugal; ^2^Centro Interdisciplinar de Estudo da Performance Humana, Faculdade de Motricidade Humana, Universidade Nova de Lisboa, 1499-002 Cruz Quebrada, Portugal; ^3^Centro de Investigação e Qualidade de Vida, Instituto Politécnico de Santarém, 2040-413 Rio Maior, Portugal; ^4^Research Centre in Sports Sciences, Health and Human Development, University of Trás-os-Montes and Alto Douro, Vila Real, 5001-801, Portugal

####### **Correspondence:** Teresa Bento (teresabento@esdrm.ipsantarem.pt) – Escola Superior de Desporto de Rio Maior, Instituto Politécnico de Santarém, 2040-413 Rio Maior, Portugal

**Background**

Falls are the main reason for the high mortality and morbidity rate among older people, and are the main cause of immobility and autonomy loss. Walking programmes are a positive way of preventing falls on apparently healthy elders. Objective: The objective of this study was to analyse the effects of a walking programme on the fall risk factors of older people, namely, the strength, balance and agility.

**Methods**

Five elders (63 and 83 years old) integrated an experimental group that participated in a weekly walking activity supervised by an exercise specialist for a sixty minutes’ period, throughout a nine months’ period. The evaluations took place in three moments, consisting of functional fitness and physical activity tests.

**Results**

Results showed an increase in the vigorous activity score, walking score, movement score and standing position score between the first and the second moment of evaluation. There was as also a significant decrease in the sitting position score, which confirmed the decrease in the sedentary behaviour of the participants. The pedometer showed us a significant increase of steps, between the two moments, and the variable related to walking, indicated positive average results in the longitudinal evaluation (0.026 ≤ 0.05).

**Conclusions**

The walking programme induced positive results in some of the fall risk factors in the older people, such as physical activity and cardiorespiratory fitness, therefore we believe that the quality of life of the participants was improved. Finally, the observed decreased in sedentary behaviour can be an important message for interventions with walking activity.

**Keywords**

Walking programme, health, falls, quality of life

#### P50 Nursing intervention programme in lifestyles of adolescents

##### Gilberta Sousa, Otília Freitas, Isabel Silva, Gregório Freitas, Clementina Morna, Rita Vasconcelos

###### Universidade da Madeira, 9000-082 Funchal, Portugal

####### **Correspondence:** Gilberta Sousa (gfranca@uma.pt) – Universidade da Madeira, 9000-082 Funchal, Portugal

**Background**

The National Health Plan refers to health promotion throughout the life cycle and during adolescence, the school environment is a reference point for the acquisition and consolidation of healthy behaviours. Interventions related to health education enable the accomplishment and better control of wellness. Objective: Diagnose the lifestyles and implement nursing interventions promoting healthy behaviours.

**Methods**

An action-research perspective developed a descriptive study (2012), in a middle school of Madeira Island (n = 270), utilizing a questionnaire adapted from the “Health behaviour in school-aged children" from WHO (α = 0.72). After prioritization of areas, the nursing intervention programme integrated education sessions; a fair and website with educational and interactive contents about physical exercise; nutrition; oral hygiene habits and knowledge of psychoactive substances. In 2014 we evaluated the effectiveness of intervention.

**Results**

There was a positive variation in percentage changes for healthier behaviours specifically: realization of 5 or more daily meals (22.7 %); water intake several times/day (75.4 %); brushing teeth after a meal (1.6 %); use of dental floss (23.0 %); playing sport outside school hours, 4 to 6 x week (6.5 %) and 2 to 3 x week (3.6 %) and a decrease of 4.6 % in respect of viewing TV 5 or more hours/day. There was also a significant percentage increase in knowledge about psychoactive substances (56.0 %).

**Conclusions**

The results express the importance and contribution of the project to significant gains in knowledge and some healthy behaviours being necessary to prolong the intervention period to increase other behaviour modifications and to extend to similar populations.

**Acknowledgements**

Authors wish to acknowledge the helpful collaboration given to this study by the Nursing students.

**Keywords**

Health behaviors, adolescents, health promotion, nursing intervention

#### P51 The person submitted to hip replacement rehabilitation, at home

##### Tatiana Azevedo^1^, Salete Soares^2^, Jacinta Pisco^3^

###### ^1^Lar Cantinho dos Avós - Santa Casa da Misericórdia de Melgaço, 4960-570 Melgaço, Portugal; ^2^Escola Superior de Saúde, Instituto Politécnico de Viana do Castelo, 4900-347 Viana do Castelo, Portugal; ^3^Unidade Local de Saúde do Alto Minho, EPE, 4904-858 Viana do Castelo, Portugal

####### **Correspondence:** Tatiana Azevedo (tatiana.azevedo@scmmelgaco.pt) – Lar Cantinho dos Avós - Santa Casa da Misericórdia de Melgaço, 4960-570 Melgaço, Portugal

**Background**

A person submitted to hip replacement, following hospital discharge to his home, faces some difficulties related to the ability to do daily activities, mobility and balance. Objectives: Evaluate the dependency level in daily activities; mobility and balance grade; quality of life perception and the effect of the rehabilitation programme.

**Methods**

A quasi-experimental study, with evaluations before/after programme implementation, during one month. Thirty participants. Data collection tools: sociodemographic/clinical questionnaire; Barthel Index; Tinetti Test; WHOQOL-bref.

**Results**

The participants have a similar distribution regarding the sex variable; the average age was 69.67 years and the BMI was above the normal level on most cases. The dependency level was higher for the following activities: having a shower; getting dressed; using the stairs and moving to a bed/chair; lower mobility and quality of life perception levels before the rehabilitation programme implementation. After the implementation, there was significant evidence that the functional independency level for all the daily activities, mobility and balance and quality of life perception increased.

**Conclusions**

After the programme’s implementation most of the participants were independent in their daily activities; they had a positive evolution concerning balance and mobility and they had a positive perception of their quality of life evolution. The only variable that had interference in the functional dependency level and quality of life social relations domain was the pain level. Implementing a rehabilitation programme with people with hip replacement, at their home, increases their capability to do daily activities, improves their mobility and balance and their quality of life perception.

**Keywords**

Hip replacement, hip arthroplasty

#### P52 Effects of Melatonin use in the treatment of neurovegetative diseases

##### Paulo P. Ferreira^1^, Efrain O. Olszewer^2^, Michelle T. Oliveira^3^, Anderson R. Sousa^4^, Ana S. Maia^4^, Sebastião T. Oliveira^4^

###### ^1^Secretaria de Saúde do Estado da Bahia, Salvador, Bahia, 41745-900, Brasil; ^2^Fundação de amparo à pesquisa e inovação do Espírito Santo, Vitória, Esprito Santo, 29066-380, Brasil; ^3^Universidade Estadual de Feira de Santana, Feira de Santana, Bahia, 44036-900, Brasil; ^4^Faculdade Nobre, Feira de Santana, Bahia, 44001-008, Brasil

####### **Correspondence:** Paulo P. Ferreira (paulopanelli@gmail.com) – Secretaria de Saúde do Estado da Bahia, Salvador, Bahia, 41745-900, Brasil

Population aging is one of the greatest contemporary public health challenges. With the change of demographic and population profile, effective therapies should be evaluated to provide better monitoring and modulation of therapeutic approaches aiming to minimize the effects of degenerative diseases.

This article studies the effects of melatonin in neurodegenerative disease and new possibilities for professional performance. It is a descriptive study, based on a literature review, using a qualitative approach and of an exploratory character. The literature review was based on the inclusion criteria: articles related to the subject, published between 2004 and 2014, containing the descriptors: melatonin, neurodegenerative diseases, Alzheimer's and Parkinson's. We opted for the content analysis technique for data analysis.

The study showed the effectiveness of the use of melatonin in the treatment of neurodegenerative diseases and its capacity to minimize the neurological effects of Alzheimer's and Parkinson’s. Melatonin is able to interact with nuclear receptors, exerting direct genomic action, by altering the expression of apoptotic genes and thereby inhibiting cell death. It has further potential neuroprotective activity, with findings suggesting the effectiveness of this therapy.

The cellular and molecular mechanisms for this activity remain poorly understood. Melatonin administration within this new professional approach seems a viable practice to minimize the aggressiveness of neurodegenerative diseases. In conclusion, it was noted there are few professionals who have knowledge of the diversity of these existing therapeutic methods, and there is a lack of studies to establish this therapy practice.

**Keywords**

Melatonin, neurodegenerative diseases, Alzheimer, Parkinson

#### P53 Review of Phytotherapy and other natural substances in alcohol abuse and alcoholism

##### Erica Santos^1^, Ana I. Oliveira^1,2^, Carla Maia^1,3^, Fernando Moreira^1,4^, Joana Santos^1,5^, Maria F. Mendes^1,3^, Rita F. Oliveira^1,2^, Cláudia Pinho^1,2^

###### ^1^Escola Superior de Tecnologia da Saúde do Porto, Instituto Politécnico do Porto, Vila Nova de Gaia, 4400-330, Portugal; ^2^Centro de Investigação em Saúde e Ambiente, Instituto Politécnico do Porto, Vila Nova de Gaia, 4400-330, Portugal; ^3^Hospital de Santo Antonio, Centro Hospitalar do Porto, EPE, 4099-001 Porto, Portugal; ^4^Department of Legal Medicine and Forensic Sciences, Faculty of Medicine, University of Porto, 4200-319 Porto, Portugal & Pharmaceutical Services, Centro Hospitalar de Vila Nova de Gaia/Espinho, EPE, Vila Nova de Gaia, 4400-129, Portugal; ^5^Centro Hospitalar de São João, EPE, Porto, 4200-319, Portugal

####### **Correspondence:** Cláudia Pinho (clp@estsp.ipp.pt) – Escola Superior de Tecnologia da Saúde do Porto, Instituto Politécnico do Porto, Vila Nova de Gaia, 4400-330, Portugal

**Background**

Alcoholism and alcohol abuse represent a worldwide problem, associated with considerable morbidity and mortality. A number of medicinal plants are reported to have preventive and therapeutic effects on alcoholism and alcohol abuse. The present review summarizes the most common natural substances used in alcohol use disorders.

**Methods**

All relevant literature databases were searched up to March 2015. The search terms were 'alcohol abuse', 'alcoholism', 'herbal medicine', 'natural products', 'phytomedicine'. Human, animal studies and reviews were included.

**Results**

Recently, some plants drew the attention of researchers, namely, danshen (*Salvia miltiorrhiza* Bge.), ginseng (*Panax ginseng* C.A. Mey.), ibogaine (*Tabernanthe iboga* Baill.), kudzu [*Pueraria lobata* (Willd.) Ohwi], and St. John’s wort (*Hypericum perforatum* L.). Reduction of alcohol absorption appears to be a common feature among most of the cited plants. A standardized formulation of kudzu produced minimal side effects, and resulted in a modest reduction in alcohol consumption in young non-treatment-seeking heavy drinkers. Hypericum extract also reduced voluntary alcohol intake to a significant degree in rat models. Danshen has no efficacy data, but can reduce alcohol intake in animal models. Other plants like Banisteriopsis caapi (Spruce ex Griseb.) Morton, Lophophora williamsii (Lem. ex Salm-Dyck) J.M. Coult., and Thymus vulgaris L. are also suggested to have beneficial effects in alcohol use disorders. Carnosine, a natural dipeptide, seems to be a promising compound for the therapy of alcoholism.

**Conclusions**

Data suggest that some plants and other natural substances may constitute novel and effective approaches for treatment of alcohol use disorders.

**Keywords**

Alcohol abuse, alcoholism, herbal medicine, medicinal plants, natural products, phytomedicine

#### P54 Dietary programme impact on biochemical markers in diabetics: systematic review

##### Eduarda Barreira, Ana Pereira, Josiana A. Vaz, André Novo

###### Escola Superior de Saúde, Instituto Politécnico de Bragança, Bragança, 5300-253, Portugal

####### **Correspondence:** Eduarda Barreira (edubarreira4@hotmail.com) – Escola Superior de Saúde, Instituto Politécnico de Bragança, Bragança, 5300-253, Portugal

**Background**

Given the high prevalence of diabetes in the population and in the elderly, it’s crucial to raise awareness of the need for people to change their eating habits and adopt healthier lifestyles. Nutrition interventions emphasizing the promotion of healthy eating have been shown to be an important point in Diabetes Mellitus treatment since it fosters a better glycaemic control. Objectives: To determine the effectiveness of the implementation of a dietary programme in the amounts of glucose and the lipid profile in patients with *Diabetes mellitus*.

**Methods**

A systematic review of the literature published in 2015 in PubMed/Medline database. It is intended to answer the research question: "What is the effectiveness of implementing a dietary programme on the blood glucose values and on the lipid profile of Diabetes Mellitus elderly subjects?" After applying the inclusion criteria 6 articles were selected from a total of 622.

**Results**

The implementation of a programme based on a higher dietary intake of polyunsaturated fatty acids, caloric restriction and intake of probiotics significantly improves glycaemic levels as well as the lipid profile in patients with Diabetes Mellitus.

**Conclusions**

It was found that the implementation of dietary programmes that aimed for a healthy and balanced diet are fundamental pillars in the treatment of diabetes mellitus, and its implementation should be fostered.

**Keywords**

Nutrition, diabetes mellitus, dyslipidaemia

#### P55 Biological approaches to knee osteoarthritis: platelet-rich plasma and hyaluronic acid

##### Luís D. Silva^1,2^, Bruno Maia^1^, Eduardo Ferreira^1^, Filipa Pires^1^, Renato Andrade^2,3^, Luís Camarinha^1^

###### ^1^Unidade Local de Saúde da Guarda, E.P.E, 6300-858 Guarda, Portugal; ^2^Clínica Espregueira-Mendes Sports Centre, FIFA Medical Centre of Excellence; 4350-415 Porto, Portugal; ^3^Faculdade de Desporto, Universidade do Porto, 4200-450 Porto, Portugal

####### **Correspondence:** Luís D. Silva (luisduartesilva1985@gmail.com) – Unidade Local de Saúde da Guarda, E.P.E, 6300-858 Guarda, Portugal

**Background**

The increasing prevalence of knee osteoarthritis has become a worldwide concern. Furthermore, the articular cartilage lesions, due to lack of self-repair, represent a clinical challenge yet to overcome. In this sense, several biological approaches have emerged as an alternative treatment. Objective: Summarize the current scientific literature in which concerns the autologous platelet-rich plasma (PRP) and hyaluronic acid (HA) effectiveness in the clinical, radiological and functional outcomes in patients with knee osteoarthritis.

**Methods**

A comprehensive literature review was made to search for level I and II human studies that assessed that assessed the clinical, radiological or functional effectiveness of PRP or HA intra-articular injections on the knee osteoarthritis. The search was made from 2005 to 2015 and the databases used were: PubMed, Cochrane Library, Scopus, CINAHL and SPORTDiscus.

**Results**

Overall, the most of the studies reported that the autologous PRP intra-articular injections provided better results when compared to the HA intra-articular injections. Both biological approaches showed superior results when compared to placebo. However, when compared to each other, the autologous PRP showed better results concerning the reduction of pain and nonsteroidal anti-inflammatory medication intake, improving the quality of life and the knee functionality. Both biological approaches showed to be safe, without significant side effects.

**Conclusions**

These presented biological approaches showed to be safe and effective in reducing the symptomatology and increasing the quality of life in patients with knee osteoarthritis. Hence, it is suggested the implementation of intra-articular injections of autologous PRP and HA in the current clinical practice.

**Keywords**

Biological approaches, Knee, Osteoarthritis, Hyaluronic Acid, Platelet-Rich Plasma, Complementary Medicine

#### P56 Platelet-rich plasma and hyaluronic acid intra-articular injections for the treatment of ankle osteoarthritis

##### Luís D. Silva^1,2^, Bruno Maia^1^, Eduardo Ferreira^1^, Filipa Pires^1^, Renato Andrade^2,3^, Luís Camarinha^1^

###### ^1^Unidade Local de Saúde da Guarda, E.P.E, 6300-858 Guarda, Portugal; ^2^Clínica Espregueira-Mendes Sports Centre, FIFA Medical Centre of Excellence; 4350-415 Porto, Portugal; ^3^Faculdade de Desporto, Universidade do Porto, 4200-450 Porto, Portugal

####### **Correspondence:** Luís D. Silva (luisduartesilva1985@gmail.com) – Unidade Local de Saúde da Guarda, E.P.E, 6300-858 Guarda, Portugal

**Background**

The burden associated with ankle osteoarthritis has been increasing in the elderly community. Several biological therapies have been suggested as an effective treatment to this pathological condition. Objectives: This study’s aim was to assess the effectiveness of autologous platelet-rich plasma (PRP) and hyaluronic acid (HA) intra-articular injections in patients with ankle osteoarthritis.

**Methods**

A computerized database search from 2005 to 2015 was carried out on PubMed, Cochrane Library, Scopus, CINAHL and SPORTDiscus databases. It included human studies that assessed the effectiveness of autologous PRP or HA intra-articular injections in patients with ankle osteoarthritis. The exclusion criteria were as follows: reviews and meta-analyses; clinical commentaries or technical notes; single study cases; animal studies. The reference lists of the most relevant papers were screened for additional studies.

**Results**

Both therapies were shown to be safe and effective in reducing pain and improving clinical and functional outcomes when compared to control or placebo groups. There was a slight superiority of the autologous PRP intra-articular injections over HA, regarding pain reduction and improving ankle functionality. Several methodological limitations across the literature were pointed out, such as: different timing, concertation and preparation modes of the injections; different outcome scores used; different follow-up timings; lack of control groups or randomized allocation.

**Conclusions**

Autologous PRP and HA injections showed promising results in patients with ankle osteoarthritis. High evidence level studies (randomized controlled trials) are needed to assess clinical superiority between PRP and HA intra-articular injections to treat ankle osteoarthritis.

**Keywords**

Ankle, Osteoarthritis, Hyaluronic Acid, Platelet-Rich Plasma, Complementary biological medicines

#### P57 The impact of preventive measures in the incidence of diabetic foot ulcers: a systematic review

##### Ana F. César^1^, Mariana Poço^2^, David Ventura^3^, Raquel Loura^4^, Pedro Gomes^5^, Catarina Gomes^6^, Cláudia Silva^7^, Elsa Melo^8^, João Lindo^8^

###### ^1^Centro de Ação Social do Concelho de Ílhavo, 3830-201 Ílhavo, Portugal; ^2^Zelar, Serviços de Apoio Domiciliário, 3810-232 Aveiro, Portugal; ^3^Walk'In Clinics, Esgueira, 3800-042 Aveiro, Portugal; ^4^São João de Deus Clinic, 1749-098, Lisboa, Portugal; ^5^Arrabida Hospital, 4400-346 Vila Nova de Gaia, Portugal; ^6^ Yasmin Residence^,^ 2500 – 064 Caldas da Rainha; ^7^Faro unit, Algarve Hospital, 8000-386 Faro, Portugal; ^8^School of Health Sciences, University of Aveiro, Aveiro, 3810-193, Portugal

####### **Correspondence:** Ana F. César (afilipacesar@gmail.com) – Centro de Ação Social do Concelho de Ílhavo, 3830-201 Ílhavo, Portugal

**Background**

Diabetes Mellitus is a chronic disease with repercussions at a global level. The diabetic foot ulcer is one of the major complications of the diabetic foot, and that with bigger repercussions on a social-economic level. The action of nursing along with the multidisciplinary team is essential to obtain health gains. Objective: Analyse the impact of primary prevention measures in diabetic foot ulcer incidence in patients with Diabetes Mellitus.

**Methods**

This study is a systematic literature review, which follows the Cochrane method, reviewing articles published 2004-2014.

**Results**

There were three randomized studies, one non randomized study and three cross quantitative studies included. They highlighted foot self-care and health education as a practice of primary prevention of the diabetic foot ulcer. The method of temperature evaluation is a simple procedure with extreme importance. Although nurses guide their patients for prevention, there are still non-studied subjects. It is fundamental to evaluate all strategies of health education, as well as the nursing records. The patient and family should be trained to self-care and for the surveillance of the lower limbs.

**Conclusions**

There are biopsychosocial factors affecting the incidence of diabetic foot ulcers in patients. The knowledge acquired by the patients does not always translate into the practice of self-care. Nursing records are key to ensuring the continuity of care.

**Keywords**

Diabetes Mellitus, Foot ulcer, Diabetic foot, Primary prevention.

#### P58 Dating violence among young adolescents

##### Joana Domingos^1^, Zaida Mendes^1^, Susana Poeta^1^, Tiago Carvalho^1^, Catarina Tomás^1,2^, Helena Catarino^1,2^, Mª Anjos Dixe^1,2^

###### ^1^School of Health Sciences, Polytechnic institute of Leiria, Leiria, 2411-901, Portugal; ^2^Health Research Unit, Polytechnic institute of Leiria, Leiria, 2411-901, Portugal

####### **Correspondence:** Mª Anjos Dixe (maria.dixe@ipleiria.pt) – School of Health Sciences, Polytechnic institute of Leiria, Leiria, 2411-901, Portugal

Violence is a complex phenomenon and the adoption of acceptable behaviours or not is influenced by culture and society, therefore, an early intervention is extremely important. This study is a quantitative, longitudinal and quasi-experimental, pre-test and post-test type, without control group. We aimed at: I) evaluating the knowledge that students have about dating violence; II) evaluating existing conflicts in dating relationships between young adolescents; and III) evaluating the effectiveness of intervention by peers using the theatre of the oppressed in knowledge about dating violence.

In the first moment (M1), a research protocol was applied composed by: Group I - Conflict in Adolescent Dating Relationships Inventory (CADRI) [1] and Group II - Knowledge about violence in dating relationships. Group II was evaluated in the second moment (M2). A non-probabilistic convenience sample consisted of 49 students, 17 females and 32 males, aged between 14 and 21 years old. Generally, students do not have affective-sexual relations ruled by violence, either as victim or as a perpetrator.

The level of knowledge about dating violence increased after the completion of the intervention by the oppressed theatre (M1 = 32.8, SD = 6.1 and M2 = 37.1, SD = 6.1) and there are statistically significant differences, proving the effectiveness of it (t = 4.265, p < 0.005). The study results highlight the relevance of this intervention and the importance of becoming aware of the phenomenon of dating violence.

**References**

1. Saavedra, RMM Prevenir antes de remediar: prevenção da violência nos relacionamentos íntimos juvenis. Tese de Doutoramento em Psicologia. 2010; Braga: Universidade do Minho.

**Keywords**

Violence, dating, teenagers

#### P59 Physical activity and motor memory in pedal dexterity

##### André Ramalho^1^, António Rosado^2^, Pedro Mendes^1^, Rui Paulo^1,3^, Inês Garcia^4^, João Petrica^1,5^

###### ^1^Escola Superior de Educação, Instituto Politécnico de Castelo Branco, 6000-767 Castelo Branco, Portugal; ^2^Faculty of Human Kinetics, University of Lisbon, 1649-028 Lisboa, Portugal; ^3^Research, Education and Community Intervention, Instituto Piaget, 3515-776 Galifonge, Viseu, Portugal; ^4^Studio Osteopatico Franchi, 6512 Giubiasco, Switzerland; ^5^Centro de Estudos em Educação, Tecnologias e Saúde, Escola Superior de Saúde, Instituto Politécnico de Viseu, 3504-510 Viseu, Portugal

####### **Correspondence:** André Ramalho (andreramalho361@gmail.com) – Escola Superior de Educação, Instituto Politécnico de Castelo Branco, 6000-767 Castelo Branco, Portugal

Regular physical activity has a positive impact on cognition and brain function. Recent studies have shown that even a single bout of aerobic exercise can improve memory and declarative learning. The main purpose of this study was to investigate if a six-month period of physical exercise could improve motor memory consolidation in elderly people in pedal dexterity. 34 subjects of both genders, with a mean age of 71 years old participated in the study. Subjects were divided in two groups: a control group and an experimental one. Before the intervention of the physical exercise programme, subjects performed a Tapping Pedal Test to measure baseline performance. After the intervention, the assessment of the impact of exercise on motor memory consolidation was held in three stages: Training, 1 hour after training and 24 hours after training. For the analysis of the results, descriptive and inferential statistics were used. The Shapiro-Wilk test was applied to the normality distribution of data and one-way ANOVA test for parametric statistics. When compared with the control group, results showed better performance in motor memory consolidation 1 hour and 24 hours after training in the experimental group. Nevertheless, these differences were not significant (p ≥ 0.05). It seems therefore that the regular physical exercise does not significantly improve motor memory consolidation capacity in pedal dexterity of elderly people.

ISRCTN Study record 31900.

ClinicalTrials.gov Identifier: NCT02731274.

**Keywords**

Physical Exercise, Motor Memory Consolidation, Tapping Pedal Test, Learning, Aging

#### P60 The effects of whole body vibration on the electromyographic activity of thigh muscles

##### Sandra Rodrigues, Rui Meneses, Carlos Afonso, Luís Faria, Adérito Seixas

###### Universidade Fernando Pessoa, Porto, 4249-004, Portugal

####### **Correspondence:** Sandra Rodrigues (sandrar@ufp.edu.pt) – Universidade Fernando Pessoa, Porto, 4249-004, Portugal

**Background**

Whole body vibration exercises consist in performing exercise on a platform which transmits mechanical stimuli (vibration) to the anatomic area in contact with the platform and which propagate throughout the body. Objective: The aim of the present study is to analyse the electromyographic response of the thigh muscles exposed to different vibrational frequencies, when compared to a non-vibration condition.

**Methods**

Eight male participants (mean age 24.13 ± 1.55) were randomly exposed to two synchronous vibration frequencies (25 Hz and 50 Hz) and a non-vibration control procedure during squatting, while monitored with surface EMG.

**Results**

For the dominant and non-dominant semitendinosus muscles there is significant increase in muscle activity at 50 Hz exposure. For the dominant biceps femoris muscle, the myoelectric activity significantly increased during both 25 and 50 Hz conditions, when compared to control. There was no statistically significant influence of vibration on vastus medialis oblique and vastus lateralis muscle activity, both for dominant and non-dominant sites and for the non-dominant biceps femoris muscle.

**Conclusions**

Exposure to vibration has no influence on quadriceps muscles (both vastus medialis and lateralis) and the non-dominant biceps femoris muscles. However, increase in hamstring activity was found for the dominant side and for the semitendinosus muscle of the non-dominant side. The primary finding in this investigation is that isometric squatting in a vibrational condition did not change agonist muscle activity, but had some degree of change in antagonist or synergist muscle activity.

**Trial registration**

Australian New Zeland Clinical Trials Registry: 370389.

ANZCTR.org.au Identifier: ACTRN12616000580471.

**Keywords**

Whole Body Vibration, Electromyography, Muscle activity

#### P61 Mental health promotion in the workplace

##### Marina Cordeiro^1^, Paulo Granjo^2^, José C. Gomes^1^

###### ^1^School of Health Sciences, Polytechnic institute of Leiria, 2411-901 Leiria, Portugal; ^2^Instituto de Ciências Sociais da Universidade de Lisboa, Universidade de Lisboa, 1600-189 Lisboa, Portugal

####### **Correspondence:** Marina Cordeiro (marina.cordeiro@ipleiria.pt) – School of Health Sciences, Polytechnic institute of Leiria, 2411-901 Leiria, Portugal

**Background**

Work can contribute to positive mental health, providing financial support, a life goal and a feeling of usefulness, or for its deterioration, due to poor work conditions. This means that work, which mostly involves stressful tasks, can cause mental health disturbance or worsen pre-existent mental health diseases. There are various policies and entities that argue the need for mental health promotion programmes, which empower people to deal with work distress and improve workplace conditions. Objective: To identify effective workplace mental health promotion interventions.

**Methods**

This systematic review was performed according to the Joanna Briggs Institute orientations, using the main descriptors “mental health”, “mental health promotion”, “occupational health”, “employee health” and “workplace”, at the MEDLINE, CINAHL and Cochrane databases. Only publications between February of 2009 and February of 2014 were considered. After meeting inclusion and exclusion criteria, six articles were selected and a narrative synthesis of the data was made.

**Results**

All the effective interventions performed were developed in a group setting, using a range of psychotherapeutic and psychoeducational techniques (mainly cognitive-behaviour therapy, stress management techniques, coping strategies and problem resolution techniques). None of the interventions were developed considering the employees’ exposure of their own needs, and some studies emphasized possible constraints when considering different settings.

**Conclusions**

Evidence shows that there are effective workplace interventions to promote mental health; however, there’s a gap in considering the real employee needs in their development. It’s necessary to develop personalized interventions to promote mental health based on employees’ opinions and their own needs.

**Keywords**

Mental Health Promotion, workplace, occupational health

#### P62 Influence of physical exercise on the self-perception of body image in elderly women: A systematic review of qualitative studies

##### Nelba R. Souza^1^, Guilherme E. Furtado^1^, Saulo V. Rocha^2^, Paula Silva^3^, Joana Carvalho^3^

###### ^1^Centro de Investigação do Desporto e da Atividade Física, Faculdade de Ciências do Desporto e Educação Física, 3040-156 Coimbra, Portugal; ^2^Núcleo de Estudos em Saúde da População, Universidade Estadual do Sudoeste da Bahia, Itapetinga, Bahia, 45700-000, Brasil; ^3^Faculdade de Desporto, Universidade do Porto, 4200-450 Porto, Portugal

####### **Correspondence:** Nelba R. Souza (nelbaef@yahoo.com.br) – Centro de Investigação do Desporto e da Atividade Física, Faculdade de Ciências do Desporto e Educação Física, 3040-156 Coimbra, Portugal

**Background**

The perception of body image in older people seems to be carried in a special way. However, the aspects linked to 'physical and functional' health and the psychological well-being associated with mental health seem to be more preponderant. Objective: This systematic review aims at reporting the effectiveness of participation in exercise programmes on the self-perception of body image in elderly women.

**Methods**

A systematic literature search of qualitative research (QR) studies was conducted using main electronic database and specialized sources such PubMed, SciELO, SPORTDiscus and PsycoINFO, articles published between 2006 and 2016. The following were used as descriptors in the English language: ‘Elderly OR Older adults’, ‘Physical activity or Motor activity’ or Physical Exercise, ‘Body Image’, ‘Qualitative research OR Qualitative study OR Qualitative’. PRISMA and TREND statement positioning were used to obtain and assess the quality of information. From the 58 articles found, 6 were included because they fulfilled the inclusion criteria. An inductive content analysis was performed on the extracted findings to identify emerging elements on the self-perception of body image in elderly women.

**Results**

Three themes related to the self-perception of body image in elderly women emerged: empowerment, social contacts, self-esteem.

**Conclusions**

Adherence to exercise programmes seem to be effective for a positive self-perception of body image assessment in the elderly. Findings showed that qualitative research can provide in-depth information about the elements that influence the assessment of body image of in older women. Multidisciplinary mixed-methods studies are recommended to establish quantitative relationships complemented with in-depth qualitative information.

**Keywords**

Older adults, physical exercise, body image

### Health in & out: Taking Care Within Diversity

#### O109 **Psychometric properties of the Portuguese version of the Éxamen Geronto-Psychomoteur (P-EGP)**

##### Marina Ana Morais^1^, Sofia Santos^1^, Paula Lebre^1^, Ana Antunes^2^

###### ^1^Faculdade de Motricidade Humana, Universidade de Lisboa, Cruz quebrada, 1499-002 Lisboa, Portugal; ^2^Casa de Saúde da Idanha, Instituto das Irmãs Hospitaleiras, 2605-077 Belas, Portugal

####### **Correspondence:** Marina Ana Morais (arodriguesmorais@gmail.com) – Faculdade de Motricidade Humana, Universidade de Lisboa, Cruz quebrada, 1499-002 Lisboa, Portugal

**Background**

Aging involves changes in psychomotor performance such as balance and fine/gross motor skills. Few studies are focused on psychomotor skills among the elderly population due to the absence of valid psychomotor instruments. Objective: This paper aims to describe the translation and validation procedures of the Portuguese version of Éxamen Geronto-Psychomoteur (P-EGP), [1].

**Methods**

The P-EGP was applied to 497 participants, with and without dementia, according with original guidelines: all applicants were psychomotor therapists specifically trained for P-EGP administration. Socio-demographic data were collected between January 2013 and September 2014.

**Results**

Results demonstrated excellent internal consistency (α > .90) and total P-EGP score (α = .92) was superior to the original (α = .83). Stability coefficients (test-retest), were above .60, showing strong stability. Factor analysis revealed three main component structures (physical constraints, motor and cognitive prevalence) accounting for 48 % of the overall variance. All domains were strongly correlated. P-EGP differentiated between people with and without dementia across the different domains, except on Joint Mobilization of Upper and Lower Limbs. Participants’ psychomotor profiles are presented, focusing on the skills most influenced by the dementia process and which may be the goal of psychomotor therapy.

**Conclusions**

The P-EGP demonstrated adequate scores of reliability and validity. It was necessary to make specific changes in some items to clarify instructions and adapt it to the Portuguese context. P-EGP domains are highly correlated, which reinforces the “psychomotor construct” and sensitivity to the presence of dementia. Further research should be conducted on this area. Practical recommendations will be given.

**References**

1. Michel S, Soppelsa R, Albaret, J. Examen Géronto Psychomoteur-Manuel D'Aplication. Paris: Hogrefe, 2011.

**Keywords**

Aging, geriatric evaluation, psychomotor performance, dementia

#### O110 Symptoms of depression in the elderly population of Portugal, Spain and Italy

##### António Calha

###### Instituto Politécnico de Portalegre, 7301-901 Portalegre, Portugal

Depression is one of the most common mental disorders affecting the quality of life of individuals. Due to their specificity, the elderly population is particularly vulnerable. The aim of this communication is to assess the prevalence of depressive symptoms in the elderly population of three southern European countries: Portugal (PT) Spain (ES) and Italy (IT).

In the present study we use a shorter version of the test from the Centre for Epidemiologic Studies - Depression Scale (CES-D8), using data from the elderly Portuguese, Spanish and Italian samples from the sixth round of the European Social Survey: PT (N. = 647); ES (N. = 371); IT (N. = 188).

The CES-D8 is a scale aimed at evaluating the frequency of depressive symptoms experienced during the week preceding interview and consists of eight items measured on a Likert scale with ranging from 0 to 3 points.

The analysis focused on two indicators drawn from the scale: I) number of “high depressive symptoms” accounted for in situations where 5 or more symptoms on the survey manifested “all or almost all of the time” during the week before the interview; II) scale score, determined by the sum of the results in each of the responses, varying between 0 and 24.

Results reveal a relatively high percentage of the elderly population with “high depressive symptoms” (PT = 2.2 %; ES = 3.4 %; IT = 4.4 %). The average values of the scores obtained in CES-D8 were the following: PT (M = 7.66; SD = 4.69) ES (M = 7.28; SD = 5.07) IT (M = 7.37; SD = 5.16). The observed differences are not statistically significant F (2, 2374) = 0.509; p = 0.601).

**Keywords**

Depressive symptoms, aging, quality of life

#### O111 Emotion regulation strategies and psychopathology symptoms: A comparison between adolescents with and without deliberate self-harm

##### Ana Xavier^1^, Marina Cunha^2^, José Pinto-Gouveia^1^

###### ^1^Cognitive-Behavioural Research Centre, Faculty of Psychology and Education Sciences, University of Coimbra, 3001-802 Coimbra, Portugal; ^2^Instituto Superior Miguel Torga, Coimbra, 3000-132, Portugal

####### **Correspondence:** Ana Xavier (ana.mj.xavier@gmail.com) – Cognitive-Behavioural Research Centre, Faculty of Psychology and Education Sciences, University of Coimbra, 3001-802 Coimbra, Portugal

**Background**

Deliberate self-harm (DSH) is a serious public health problem due to its high prevalence during adolescence and its link to psychological impairments. Despite the theoretical emphasis on the role of emotion dysregulation in DSH, few studies have analysed this relationship among a community sample of adolescents. Objective: The current study aims to compare adolescents with and without DSH in emotion regulation processes and psychopathological symptoms, and to examine the contribution of maladaptive emotion regulation processes to predict DSH.

**Methods**

The sample consists of 180 adolescents with DSH and 180 adolescents without a history of DSH. The total sample is aged between 12 and 18 years old (M = 14.98, SD = 1.77) and was recruited in middle and secondary schools (mean of years of education = 9.54, SD = 1.63).

**Results**

A one-way ANOVA showed that adolescents with DSH reported higher levels of maladaptive emotion regulation processes, such as dissociation, rumination, experiential avoidance and self-criticism than adolescents without DSH. Adolescents with DSH also experience higher levels of depression, anxiety and stress symptoms than adolescents without DSH. Results from logistic regression analyses showed that the full model accounted for between 26 % and 35 % of DSH. The best predictors of DSH are depressive symptoms, being female and the most severe form of self-criticism (self-hate). When this form of self-criticism was not included in the equation analysis, other risk emotional factors for DSH emerged, namely dissociation and lower levels of self-compassion.

**Conclusions**

These findings unveil the emotional risk factors for DSH in adolescence.

**Keywords**

Adolescence, Deliberate self-harm, emotion regulation processes

#### O112 Prevalence of physical disability in people with leprosy

##### Liana Alencar^1,2^, Madalena Cunha^1,2^, António Madureira^2^

###### ^1^Centro de estudos em educação, tecnologias e saúde, Escola Superior de Saúde, Instituto Politécnico de Viseu, 3504-510 Viseu, Portugal; ^2^Escola Superior de Saúde, Instituto Politécnico de Viseu, 3504-510 Viseu, Portugal

####### **Correspondence:** Madalena Cunha (madalenacunhanunes@gmail.com) – Centro de estudos em educação, tecnologias e saúde, Escola Superior de Saúde, Instituto Politécnico de Viseu, 3504-510 Viseu, Portugal

**Background**

Hansen’s disease is a curable nosologic entity caused by Mycobacterium Leprae with a predilection for the peripheral nervous system which is associated with incapacities, with higher prevalence among men. Given that it is an infectious-contagious disease, the following are useful strategies for diminishing its sequels: early diagnosis, monitoring patients and household contacts, promoting educational campaigns. Objective: To assess the degree of disability in people with Hansen’s disease.

**Methods**

An observational study conducted in 104 Brazilians diagnosed with Hansen’s disease. Most were male (60.6 %) with an average age of 37 years, dark-skinned (49.0 %) and had completed basic education (34.6 %). Data collection was authorized and supported in the Notifiable Diseases Information System.

**Results**

The operational and clinical classification of Hansen’s disease revealed that 47.1 % were paucibacillary and 52.9 % were multibacillary. 32.0 % were diagnosed with the borderline type and 26.0 % with the tuberculoid type. At the time of the diagnosis 25.0 % had a physical disability. The level of physical disability (LPD) was the following: Level 0 - 73 (70.19 %); level I - 17 (16.35 %) and 9 (8.65 %) had level II deformities. LPD was not registered in 5 cases.

**Conclusions**

Physical disability associated with Hansen’s disease is significant and a public health problem in Brazil. Thus, it is urgent to implement measures for the eradication of this endemic disease and for the control of its sequels.

**Keywords**

Physical, Disability, Leprosy

#### O113 Quality of life and self-esteem in type 1 and type 2 diabetes mellitus patients

##### Ilda Cardoso^1,2^, Ana Galhardo^1,3^, Fernanda Daniel^1,4^, Vítor Rodrigues^1,2^

###### ^1^Instituto Superior Miguel Torga, Coimbra, 3000-132, Portugal; ^2^Faculty of Medicine, University of Coimbra, 3000-548 Coimbra, Portugal; ^3^Centro de Investigação do Núcleo de Estudos e Intervenção Cognitivo Comportamental, Universidade de Coimbra, 3001-802 Coimbra, Portugal; ^4^Centre for Health Studies and Research, University of Coimbra, 3004-512 Coimbra, Portugal

####### **Correspondence:** Ilda Cardoso (ildamassano@ismt.pt) – Instituto Superior Miguel Torga, Coimbra, 3000-132, Portugal

**Background**

Diabetes mellitus (DM) is one of the most prevalent chronic diseases, being responsible for high rates of morbidity and mortality. In Portugal its prevalence is about 12.7 %. Quality of life (Qol) can be influenced by the individual physical and mental health, social environment and personal beliefs. Self-esteem is related to the sense of personal value or self-worth and may be a relevant aspect of coping with chronic disease. Objectives: To explore the existence of differences between type 1 and type 2 diabetes patients regarding Qol and self-esteem. Gender differences in these two groups of patients were also investigated.

**Methods**

Participants were 333 with a DM diagnosis for over a year who completed World Health Organization Quality of Life (WHOQOL-Bref) and the Rosenberg Self-Esteem (RSE]. The study had a cross-sectional design.

**Results**

Patients with type 1 DM show significant higher scores in all Qol dimensions as well as in self-esteem (p < 0.05) when compared to type 2 DM patients. Concerning gender differences type 1 male patients and type 1 female patients do not differ. When considering type 2 DM, men score significantly higher than women in self-esteem (p = 0.002) and in general Qol (p = 0.036), physical (p = 0.008), psychological (p < 0.001) and environment (p = 0.001) dimensions.

**Conclusions**

Independently of being male or female type 1 DM patients show better Qol and higher self-esteem. Regarding type 2 DM it seems important to integrate Qol and self-esteem in the overall treatment approach, particularly with women.

**Keywords**

Diabetes mellitus, self-esteem, quality of life, gender differences

#### O114 Cross-cultural comparison of gross motor coordination in children from Brazil and Portugal

##### Leonardo Luz^1^, Tatiana Luz^1^, Maurício R. Ramos^2^, Dayse C. Medeiros^3^, Bruno M. Carmo^1^, André Seabra^4^, Cristina Padez^5^, Manuel C. Silva^5^

###### ^1^Universidade Federal de Alagoas, Maceió, 57072-900, Alagoas, Brasil; ^2^Instituto Federal de Alagoas, Maceió, 57035-660, Alagoas, Brasil; ^3^Centro Universitário Cesmac, Maceió, 57051-160, Alagoas, Brasil; ^4^Universidade do Porto, Porto, 4099-002, Portugal; ^5^Universidade de Coimbra, Coimbra, 3004-504, Portugal

####### **Correspondence:** Leonardo Luz (ltluz@ig.com.br) – Universidade Federal de Alagoas, Maceió, 57072-900, Alagoas, Brasil

**Background**

Motor coordination is an important predictor of physical activity and health in childhood. However, childhood competence levels in many countries are lower than desired. Due to the many different motor skill instruments in use, children’s motor coordination across countries is rarely compared. Objective: This study aimed to examine the gross motor coordination of children from Brazil and Portugal.

**Methods**

The cross-sectional sample included 121 boys (61 from Brazil) aged 8 years old from schools in Brazil and Portugal. The subjects completed morphological measurements. Motor coordination was evaluated using a 4-item battery (*Körperkoordinationstest für Kinder-KTK*): walking backward on balance beams (WB), moving sideways on boxes (MS), jumping sideways across a wooden slat (JS), and hopping for height on one leg (HH). The percentage of predicted mature stature was used to assess the somatic maturation. MANOVA and MANCOVA (with waist circumference as covariate) were used to examine the independent effect of country on morphological variables, somatic maturation and KTK items. Significance level was set at 5 % for all inferential statistics.

**Results**

Our results showed a significant country effect (Wilks’λ = 0.547; F = 23.993; p < 0.05) and suggested that Portuguese boys attained better performance compared to Brazilian boys in JS and HH. The current results suggested that they are country-related and not substantially explained by biological maturation.

**Conclusions**

The low levels reported by Brazilian boys in JS and HH may be the result of maturity status and cultural differences in physical activity contexts such as physical education and sports participation.

**Keywords**

Motor coordination, KTK, children, cross-cultural comparison

#### O115 Electrocardiographic differences between African and Caucasian people

##### António Rodrigues, Patrícia Coelho, Alexandre Coelho

###### Escola Superior de Saúde Dr. Lopes Dias, Instituto Politécnico de Castelo Branco, Castelo Branco 6000-767, Portugal

####### **Correspondence:** António Rodrigues (afrodrigues6@gmail.com) – Escola Superior de Saúde Dr. Lopes Dias, Instituto Politécnico de Castelo Branco, Castelo Branco 6000-767, Portugal

**Background**

In the second half of the 20th century, life expectancy of African people was considerably lower than Caucasians, identifying the need to study the differences between racial and ethnic groups so that health measures can be taken. Objective: To verify if there are electrocardiographic differences between African and Caucasian individuals.

**Methods**

The sample includes a total of 122 individuals, collected between October 2011 and January 2012. All the individuals answered a personal questionnaire and did a conventional 12-lead electrocardiogram. The study included individuals from both races and genders, with a sedentary life style, without alcoholic habits, non-smokers, non-obese, with similar sociodemographic conditions, with no kind of disease associated, with ages between 19 and 35 years, and who had agreed to take part in the study by signing an informed agreement.

**Results**

With this study, we were able to confirm that individuals of African race have more prevalence of negative T waves and/or biphasic in V1 (p < 0.001), V2 (p = 0.014) and V3 (p = 0.043), higher prevalence of ST-segment elevation in V1 (p < 0.001), V2 (p = 0.001), V3 (p = 0.010) and V4 (p = 0.027), a higher dispersion of QT interval (p = 0.001) and higher prevalence of left ventricular hypertrophy (Sokolow-Lyon index – p < 0.001).

**Conclusions**

Although we found statistical differences in the average values and in the prevalence, we did not actually verify electrocardiographic differences between the racial groups. In spite of all the study results, we conclude that they have no pathological value, and are therefore considered as normal variations.

**Keywords**

Eletrocardiography, African, Caucasian

#### O116 Factors associated with domestic, sexual and other types of violence in the city of Palhoça - Brazil

##### Madson Caminha^1^, Filipe Matheus^1^, Elenice Mendes^2^, Jony Correia^2^, Marcia Kretzer^1^

###### ^1^Universidade do Sul de Santa Catarina, Palhoça, Santa Catarina, 88137-270 Brasil; ^2^Secretaria Municipal de Saúde de Palhoça, Palhoça, Santa Catarina, 88132-149, Brasil

####### **Correspondence:** Madson Caminha (madsoncaminha@hotmail.com) – Universidade do Sul de Santa Catarina, Palhoça, Santa Catarina, 88137-270 Brasil

**Background**

Violence is intentional use of force or power in the form of a threat or effectively, against oneself, another person, group or community, which leads to or is most likely to result in injury, death, psychological harm or developmental disorders. Brazil displays an increased tendency of the notifications in all kinds of violence. Objective: Analyse the factors related to types of violence notified in the city of Palhoça, Brazil, during the period 2010-2015.

**Methods**

Cross-sectional study, with analysis of 890 records of notification/personal investigation of domestic violence, sexual and/or other types of violence. Data obtained from Notifiable Diseases Information System. Analyses in SPSS 20.0, Chi-square test with p ≤ 0.05. Approved by the Research Ethics Committee of University of Southern Santa Catarina No 1.370.313.

**Results**

Prevalent in females (53.1 %), older than 18 years (99.7 %), white (66.2 %), low educational level (38.7 %), single (40.5 %), urban zone (98.2 %) occurrence in the home (54.0 %), have suffered violence other times (32.2 %), have disabilities/disorders (13.7 %), pregnant women (2.7 %). Male aggressor (75.7 %), partner/boyfriend (20.3 %), alcohol use (24.5 %). Higher incidence of physical violence (83.1 %), followed by sexual (10.8 %) and psychological (9.4 %) violence. Physical force as a means of aggression (58.7 %), cut injury/blunt trauma (58.2 %), self-harm by 19.7 %. Death in 1.4 %. Significant correlation (p ≤ 0.05) between physical violence with physical/mental disability and mental illness, suffering violence other times, male sex and alcohol abuse.

**Conclusions**

There was a higher report of physical violence on female adults performed by family members, associated with male aggressors and alcohol abuse.

**Keywords**

Violence, Domestic violence, Social violence

#### O117 Tinnitus prevalence study of users of a hospital of public management - Spain

##### Francisco J. Hernandez-Martinez^1^, Juan F. Jimenez-Diaz^2^, Bienvendida C. Rodriguez-De-Vera^2^, Carla Jimenez-Rodriguez^2^, Yadira Armas-Gonzalez^3^

###### ^1^Cabildo de Lanzarote, 35500, Arrecife, Lanzarote, Las Palmas, España; ^2^Universidad de Las Palmas de Gran Canaria, 35001 Las Palmas de Gran Canaria, Las Palmas, España; ^3^Servicio Canario de la Salud, 35500, Arrecife, Lanzarote, Las Palmas, España

####### **Correspondence:** Francisco J. Hernandez-Martinez (fjhernandez@denf.ulpgc.es) – Cabildo de Lanzarote, 35500, Arrecife, Lanzarote, Las Palmas, España

**Background**

The tinnitus or Tinnitus is the subjective perception of a continuous noise in the ears or head in the absence of noise to justify it. It affects 25 % of the general population and in most cases is associated with hearing loss. Objective: To determine the prevalence of tinnitus in users attending otolaryngology consultations and examine the sociodemographic characteristics of the study population.

**Methods**

A descriptive observational, cross-sectional and quantitative study among users of the Canary Health Service (Spain), who are undergoing a "total audiometry or pure tone" after referral to ENT Hospital Jose Molina Orosa. Data collection period was from January to April 2015, respecting the right to confidentiality, privacy and freedom, as well as the voluntary nature of participation.

**Results**

Of 412 patients who attended the consultation of Otolaryngology, 84 patients with tinnitus were included in the sample. The study determined that the prevalence is 20.3 %, with a predominance of women among the group aged 41-60 years. 77.3 % of patients had hearing loss associated with tinnitus, and, on average, adaptation to symptoms in patients was associated with hypertension, depression and insomnia.

**Conclusions**

The degree of adaptation of the patient with tinnitus needs an acceptance of living with it. Health education programmes for the prevention and improved quality of life for people who suffer from this symptom are recommended.

**Keywords**

Tinnitus, prevalence, public hospital, Spain

#### O118 Difficulties experienced by parents of children with diabetes mellitus of preschool age in therapeutic and nutritional management

##### Cátia Rodrigues, Rosa Pedroso

###### Escola Superior de Enfermagem de Coimbra, Coimbra, 3046-851 Coimbra, Portugal

####### **Correspondence:** Cátia Rodrigues (catialexrodrig@gmail.com) – Escola Superior de Enfermagem de Coimbra, Coimbra, 3046-851 Coimbra, Portugal

**Background**

The national and international literature has shown that the incidence of type 1 Diabetes Mellitus (DM1) in children has increased significantly, becoming the chronic disease with the highest incidence in children and adolescents in Portugal. Objective: To describe the difficulties parents experience in the management of therapy and nutrition, evaluate the difficulties in the management of the disease of their children, describe the strategies used by parents in the management of therapy and feeding their children.

**Methods**

A descriptive exploratory study of qualitative nature was developed, conducted through semi-structured interviews. An intentional sample consisting of ten parents was used, followed in endocrinology consultation of the Paediatric Hospital of Coimbra from July to August 2014. Data were analysed using the content analysis technique, according to Bardin (2009) [1].

**Results**

Through the analysis made of the parents’ discourse, five main categories emerged corresponding to the difficulties experienced in the therapeutic and nutrition management of their children: nutrition control and calculation of units of insulin in the day nursery, negative feelings that the children associated with the illness, negative feelings experienced by parents, complexity of the calculations and, finally, parents’ opinions and suggestions.

**Conclusions**

The difficulty of parents in the calculations inherent in this process was evident. Parents rarely delegate functions associated with therapeutic and nutrition management to childcare professionals, claiming insecurity and lack of knowledge. It was also remarkable to experience negative feelings both by children and by parents associated with the disease.

**References**

1. Bardin L. Análise de Conteúdo. Lisboa: Edições 70; 2009.

**Keywords**

Diabetes mellitus type 1, preschool children, difficulties, nutrition, therapeutic

#### O119 E-mental health - “nice to have” or “must have”? Exploring the attitudes towards e-mental health in the general population

##### Jennifer Apolinário-Hagen, Viktor Vehreschild

###### Department for Health Psychology, FernUniversität in Hagen, 58084 Hagen, Germany

####### **Correspondence:** Jennifer Apolinário-Hagen (jennifer.apolinario-hagen@fernuni-hagen.de) – Department for Health Psychology, FernUniversität in Hagen, 58084 Hagen, Germany

**Background**

In the present research literature, e-mental-health is associated with benefits such as improved access to mental health care including online self-help services and psychotherapy [1]. However, the evidence base on attitudes towards e-therapies from the users´ perspective is very limited [2]. Objective: The aim of the study was to explore attitudes towards e-mental health in the general population. A further goal was to identify the utilisation of publicly available online health services.

**Methods**

To measure attitudes towards standardised guided e-therapy, 436 German-speaking persons (M = 29 years, SD = 10.13; 78 % female) completed a 17-item-questionnaire that contains items on different aspects of e-therapies, including statements that reflect commonly proposed benefits and risks. Factor analysis (extraction method: Promax) resulted in a two-dimension-structure of the measure, i.e. “usefulness (of e-therapies)” and “comparability (to face-to-face- therapies)”. The feasibility of the measure was confirmed by a recent pilot study (n = 1.546). Besides, online habits regarding health issues were examined.

**Results**

Attitudes towards guided e-therapies were overall neutral (“usefulness”) or tended to be rather pessimistic (“comparability”). The majority of participants reported not having heard of e-therapies before (57 %), whereas e-counselling was attended by 11 %. Additionally, 57 % reported searching online for health information only rarely or occasionally. Websites of physicians or psychologists were viewed as the most trustworthy resources.

**Conclusions**

Research with the scope on attitudes towards e-mental health is at an early stage. The results could be biased due to the selective sample. The further development and validation of measures is strongly recommended.

**References**

1. Musiat P, Goldstone P, Tarrier N. Understanding the acceptability of e-mental health - attitudes and expectations towards computerised self-help treatments for mental health problems. BMC Psychiatry, 2014; 14:109. doi: 10.1186/1471-244X-14-109.

2. Casey LM, Joy A, Clough BA. The Impact of Information on Attitudes Toward E-Mental Health Services. Cyberpsychology, Behavior, and Social Networking. 2013; 16(8): 593-598.

**Keywords**

Attitudes, e-therapy, online self-help, e-mental health, health literacy, mental health care, access to care, online psychotherapy, e-counselling, self-report measure

#### O120 Violence against children and adolescents and the role of health professionals: Knowing how to identify and care

##### Milene Veloso, Celina Magalhães, Isabel Cabral, Maira Ferraz

###### Universidade Federal do Pará, Belém, Pará, 66075-110, Brasil

####### **Correspondence:** Milene Veloso (mveloso@ufpa.br) –Universidade Federal do Pará, Belém, Pará, 66075-110, Brasil

Violence against children and adolescents is a serious public health problem worldwide and health professionals have an important role in the identification and treatment of cases. This work investigated the perception of health professionals about violence against children and adolescents, to propose possibilities for action and the drafting of a pamphlet to be used in the training of these professionals.

The study was developed in Belém-Pará-Brazil in the period 2011 to 2014, from the application of two structured questionnaires with 174 health professionals. The data were analysed via the BioEstat 5.3 programme.

The results pointed out that about 30.00 % of health professionals never identified child or adolescent victims of violence. Neglect was perceived by 60.74 % of professionals; however, only 19.05 % of these reported having it notified. Sexual violence was the least referred to (24.14 %), but exhibited a higher rate of notification (50.00 %). Physical violence was notified by 39.47 % of professionals who identified it, and psychological violence by 34.88 %. Among the upper level professionals, 50.00 % said they did not know the means of notification and 86.11 % never used it. The main doubts described by health professionals were: how to identify signs and symptoms of violence (48.61 %), the process of notification of cases (27.77 %) and how to deal with the victims and the families (25 %).

The results demonstrated the urgent need for a policy of continued education focused on health professionals, thus ensuring full protection for children and adolescents. The guide is in the wrap-up phase and application.

**Keywords**

Violence, children and adolescents, Health professionals

#### O121 Marital violence. A study in the Algarve population

##### Filipe Nave, Emília Costa, Filomena Matos, José Pacheco

###### Escola Superior de Saúde, Universidade do Algarve, Faro, 8000-510, Portugal

####### **Correspondence:** Filipe Nave (fnave@ualg.pt) – Escola Superior de Saúde, Universidade do Algarve, Faro, 8000-510, Portugal

Marital violence may be included in domestic violence (DV), since this is a type of violence that occurs in an intimate relationship between two partners or spouses and which, because it happened in an intimate space, is not easily identifiable. It affects multiple dimensions of health in all members of a family.

This study aims to evaluate and characterize marital violence in the population of the Algarve. It is an exploratory, descriptive, correlational and transversal study using a quantitative methodology. Apart from a sociodemographic questionnaire, we used the Inventory of Marital Violence (IVC).

Among the 504 men and women respondents in the Algarve region, statistically significant differences were not found in any of the types of violence suffered. In terms of incidence of domestic violence in this sample we found that 49.4 % had experienced psychological violence, 10.1 % reported having experienced some episode of major physical violence, 30.8 % reported having experienced minor physical violence, 42.9 % said they had been abused in the socio-economic context and 42.9 % had never been a victim of sexual violence.

About 60 % of the participants stated that they had already been victims of some form of violence. Psychological violence and socio-economic violence are those with higher values, exceeding 40 % of respondents. Minor physical violence and sexual violence also had poor values, having both been reported by more than 30 % of respondents. Major physical violence is the least reported with an approximate value of 10 % of the participants.

**Keywords**

Domestic violence, marital violence, Inventory of Marital Violence (IVC).

#### O122 Clinical factors and adherence to treatment in ischemic heart disease

##### António Dias, Carlos Pereira, João Duarte, Madalena Cunha, Daniel Silva

###### Centro de Estudos em Educação, Tecnologias e Saúde, Escola Superior de Saúde, Instituto Politécnico de Viseu, 3504-510 Viseu, Portugal

####### **Correspondence:** António Dias (madureiradias@gmail.com) – Centro de Estudos em Educação, Tecnologias e Saúde, Escola Superior de Saúde, Instituto Politécnico de Viseu, 3504-510 Viseu, Portugal

**Background**

Non-adherence to treatment is a motive of concern by the scientific community, since it is considered as an important problem of public health. Approximately 50 % of patients with ischemic heart disease (IHD) do not get a clinical benefit, due to low adherence to treatment (AT). Objectives: Determine the prevalence of AT and relate the influence of several clinical factors in AT.

**Methods**

A cross-sectional study, involving 254 patients with IHD, attending follow-up consultation at the hospital. Patients had a mean age of 66.9 ± 11.6 years; 74.0 % were male; 73.2 % were “married”; 69.3 % had education up to the “4^th^ grade”. We used socio-demographic and clinic questionnaire and Measure Treatment Adherence.

**Results**

The prevalence of non-adherence of patients with IHD was 50.4 %. Patients who adhered more to treatment were those who: I) were diagnosed with “acute myocardial infarction with ST elevation” compared to those who had “unstable angina”; II)had the disease “1 year ago” compared to those who had “4 years ago”; III) had the first episode of acute coronary syndrome compared to those who relapsed; IV) had “1 risk factor” compared to those who had “4 and 5 risk factors”; V) were treated with “primary angioplasty” compared to those that enjoyed “conservative” treatment; VI) were asymptomatic compared to those that reported “limitation or severe limitation to activities”.

**Conclusions**

The results are consistent with previous studies, confirming the low prevalence to AT regarding the cardioprotective medication. Some clinical factors in AT proved to be predictors of adherence.

**Keywords**

Adherence to treatment, ischemic heart disease, clinical factors

#### O123 Can religiosity improve optimism in participants in states of illness, when controlling for life satisfaction?

##### Lisete M. Mónico^1^, Valentim R. Alferes^1^, Mª São João Brêda^1^, Carla Carvalho^1^, Pedro M. Parreira^2^

###### ^1^Faculdade de Psicologia e Ciências da Educação, Universidade de Coimbra, 3001-802 Coimbra, Portugal; ^2^Escola Superior de Enfermagem de Coimbra, Coimbra, 3046-851, Portugal

####### **Correspondence:** Lisete M. Mónico (lisete_monico@fpce.uc.pt) – Faculdade de Psicologia e Ciências da Educação, Universidade de Coimbra, 3001-802 Coimbra, Portugal

**Background**

Considering the classic literature on the effects of religiosity on health, well-being, and risk behaviours, the issue of the influence of religiosity on optimism has been less studied, especially when controlling for life satisfaction. In states of illness, variables such as optimism and life satisfaction assume an important role. Objectives: To analyse the role of religiosity in optimism factors (externality vs. internality) in groups characterized by states of illness or health, controlling for life satisfaction.

**Methods**

The sample consists of 264 patients with chronic diseases or hospitalized due to a given pathology (M = 52.49; SD = 17.69 years old; 64.4 % female), and a comparison group composed of 315 healthy individuals (M = 40.63; SD = 16.63 years old; 78.8 % female). All participants answered the CRSV Questionnaire (2011) composed of several measures of religiosity, optimism and life satisfaction (with good psychometric properties).

**Results**

For ill participants, we didn’t find an association between religiosity and optimism measures when controlling for life satisfaction, both for externality (β = .02) and internality optimism (β = .00); in these patients, life satisfaction predicted internality (β = .65) more than externality optimism (β = .25). For healthy participants, religiosity predicted life satisfaction (β = .19) and optimism (β = .16 for externality optimism; β = .14 for internality optimism); both internality (β = .86) and externality optimism (β = .88) were associated with life satisfaction.

**Conclusions**

Religiosity only influences optimism in healthy individuals. For ill participants, optimism was only anchored in life satisfaction, and internality optimism was promoted more by life satisfaction than optimism based on external causes. For healthy individuals, life satisfaction equally promotes internality and externality optimism.

**Keywords**

Religiosity, optimism, life satisfaction, internality, externality

#### O124 Empowerment, knowledge and quality of life of people with diabetes type 2 in the Alto Minho Health Local Unit

##### Mª Carminda Morais^1,2^, Pedro Ferreira^1^, Rui Pimenta^1^, José Boavida^3^

###### ^1^Centro de Estudos e Investigação em Saúde, Faculdade de Economia, Universidade de Coimbra, 3004-512 Coimbra Portugal; ^2^Escola Superior de Saúde, Instituto Politécnico de Viana do Castelo, 4900-347 Viana do Castelo, Portugal; ^3^Direção geral de Saúde (DGS), 1049-005 Lisboa, Portugal

####### **Correspondence:** Mª Carminda Morais (carmindamorais@ess.ipvc.pt) – Centro de Estudos e Investigação em Saúde, Faculdade de Economia, Universidade de Coimbra, 3004-512 Coimbra Portugal

**Background**

Diabetes mellitus type 2 is one of the non-communicable diseases with increasing prevalence, associated with strong physical, emotional and socio-economic repercussions. Therefore, it requires new integrated approaches to prevent and combat the disease, based on the evidence already being produced. Objective: This study aimed at assessing knowledge, empowerment and quality of life of people with type 2 diabetes, being followed in Alto Minho Region of Portugal.

**Methods**

This correlational descriptive study was conducted on a random sample of 537 individuals, 277 of them in primary care (clusters of health care centres, ACES). The questionnaire encompassed a sociodemographic and clinical characterization part, together with the DES-SF (empowerment), DKT (knowledge) and EQ-5D (quality of life) scales.

**Results**

Individuals’ mean age was 64.7 ± 11.7 (ACES) and 62.3 ± 12.9 (hospital). Concerning empowerment and quality of life we found similar mean scores in both settings 3.7 ± 0.9 and 0.6 ± 0.3 in ACES, and 3.7 ± 0.7 and 0.6 ± 0.3 in hospital. On the other hand, knowledge was higher in the hospital (66.7 ± 12.4) vs. primary care (59.7 ± 16.3), especially in non-insulin treated individuals. Men followed in hospital also showed higher empowerment than women although having similar knowledge. The highest knowledge deficits were related to hypoglycaemia handling, meaning of haemoglobin HbA1C, ketoacidosis signs among the insulin treated, and the care of the feet. Younger and less educated individuals showed higher knowledge of quality of life and empowerment.

**Conclusions**

There is lack of knowledge in fundamental aspects of the disease’s management, followed by an unrealistic feeling of empowerment, taking into account the evidence produced. Local intervention strategies should consider these findings.

**Keywords**

Type 2 diabetes, empowerment, knowledge, quality of life

#### O125 Antihypertensive therapy adherence among hypertensive patients from Bragança county, Portugal

##### Isabel C. Pinto^1^, Tânia Pires^1,2^, Catarina Silva^1^

###### ^1^Instituto Politécnico de Bragança, Bragança, 5300-253, Portugal; ^2^Centro de Investigação de Montanha, Escola Superior Agrária, 5300-253 Bragança, Portugal

####### **Correspondence:** Isabel C. Pinto (isabel.pinto@ipb.pt) – Instituto Politécnico de Bragança, Bragança, 5300-253, Portugal

**Background**

Hypertension increases the risk of cardiovascular diseases and is highly prevalent worldwide, reaching more than a quarter of the Portuguese population. Poor antihypertensive therapy adherence has been identified as the main cause of failure to control hypertension. Objective: To estimate the prevalence of antihypertensive therapy adherence and related factors.

**Methods**

This cross-sectional study was based on a questionnaire, with MAT scale (measure of adherence to therapy) validated for the Portuguese population [1], applied to 122 hypertensive patients from Bragança county, in northern Portugal. To assess therapy adherence, those whose average adherence levels were ≥ 5 were called adherent. Descriptive statistics were used, correlations were accessed using qui-square test, with a significance level of 5 %.

**Results**

The sample consisted mainly of females (59.1 % vs. 40.9 %), aged between 31 and 92 years old (mean 69.8). The participants show high antihypertensive therapy adherence level (82.8 %). Only marital status is related to therapy adherence, with married or widowed people being those who least adhered to antihypertensive treatment (p = 0.04).

**Conclusions**

This study shows that a high prevalence of hypertensive patients adhered to the antihypertensive therapy prescribed, the married or widowed being those who least adhered to treatment.

**References**

1. Delgado AB, Lima ML. Contributo para validação concorrente de uma medida de adesão aos tratamentos. Psicologia, Saúde & Doenças. 2001; 2(2): 81-100.

**Keywords**

Antihypertensive therapy adherence, hypertensive patients, therapy adherence, therapy non-adherence

#### O126 Subjective perception of sexual achievement - An exploratory study on people with overweight

##### Maria Ribeiro^1,2^, Maria Viega-Branco^3^, Filomena Pereira^4^, Ana Mª Pereira^3^

###### ^1^Escola Superior Agrária, Instituto Politécnico de Bragança, 5300-253 Bragança, Portugal; ^2^Centro de Estudos Transdisciplinares para o Desenvolvimento, Universidade de Trás-os-Montes e Alto Douro, 5000-801 Vila Real, Portugal; ^3^Escola Superior de Saúde, Instituto Politécnico de Bragança, 5300-121 Bragança, Portugal; ^4^Faculdade de Medicina, Universidade de Lisboa, 1649-028 Lisboa, Portugal

####### **Correspondence:** Maria Ribeiro (xilote@ipb.pt) – Escola Superior Agrária, Instituto Politécnico de Bragança, 5300-253 Bragança, Portugal

The literature reports that Body Mass Index (BMI) changes are directly related to sexual interest. Individuals with overweight and obesity self-report fewer sexual partners and are less likely to have a romantic relationship compared to their non-obese counterparts.

The objectives of this work are to know the level of perceived sexual achievement, in overweight people with the Binge Eating Scale (BES) and to study the association between age and the perception of quality of sex in people with overweight are the objectives of this work.

An exploratory and quantitative study was developed, based on a sample of 218 patients of both genders, aged between 18 and 65 years. Data was collected in various hospitals of northern and central Portugal with the Sexual Satisfaction Index (SSI) being used for this purpose.

Of all participants, 64.5 % expressed overweight. On a scale of 0 to 100, the SSI was, on average, 26.7 (SD = 14.6). On average, Individuals with severe poor scores on the BES registered the highest level of sexual dissatisfaction. There were, statistically, significant differences in the SSI, among individuals with and without overweight. Dissatisfaction is greater the more advanced the age of participants. Contrary to what is reported in the literature, there was no difference between individuals with and without overweight according to the number of sexual partners.

The results obtained with this research are consistent with the literature, except for those concerning the number of sexual partners.

**Keywords**

Perception, sexual achievement, overweight, Portugal

#### O127 Physical activity level and associated factors in hypertensive individuals registered in the family health strategy of a basic health unit from the city of Palhoça, Santa Catarina, Brazil

##### Fabrícia M. Almeida, Gustavo L. Estevez, Sandra Ribeiro, Marcia R. Kretzer

###### Universidade do Sul de Santa Catarina, Palhoça, Santa Catarina, 88137-270 Brasil

####### **Correspondence:** Marcia R. Kretzer (marcia.kretzer1@gmail.com) – Universidade do Sul de Santa Catarina, Palhoça, Santa Catarina, 88137-270 Brasil

**Background**

Hypertension is a major risk factor for cardiovascular disease, while physical activity is associated with decreased risk of developing hypertension. Objective: To assess the level of physical activity and associated factors in hypertensive individuals registered in the Family Health Strategy in a Basic Health Unit (BHU) in the city of Palhoça, Santa Catarina, Brazil.

**Methods**

A transversal study with 134 hypertensive patients in a BHU in the period of June to November, 2015. The International Physical Activity Questionnaire (IPAQ) was applied. Analyses were carried out in SPSS 18.0, the averages were compared with the t-test (p ≤ 0.05) and a 95 % confidence interval was established. It was approved by the Unisul Research Ethics Committee.

**Results**

Women (59.8 %), medium age of 64.94 years old (SD 12.7), not working (78.4 %), smoking (16.4 %), of which 68.2 % smoke more than 20 cigarettes a day. Active in physical activity (42.6 %), irregularly active (41.5 %) and 6.2 % sedentary. Hypertension lower than 159/99 mmHg (78.8 %), diabetics (38.8 %), obesity (32.8 %), kidney disease (20.9 %), heart disease (17.9 %). 76.8 % use up to 2 antihypertensives. In smokers, the highest level of physical activity is associated with a consumption of less than 20 cigarettes a day (p = 0.008).

**Conclusions**

The hypertensives accompanied by the Family Health Strategy in the City of Palhoça, Brazil, perform regular physical activity, maintain low blood pressure and use up to two antihypertensive drugs. Smokers with a consumption of more than 20 cigarettes a day are more sedentary.

**Keywords**

Physical activity level, hypertension, associated factors

#### O128 Perception of functional fitness and health in non-institutionalised elderly from rural areas

##### Paulo V. João^1^, Paulo Nogueira^2^, Sandra Novais^2^, Ana Pereira^1,3^, Lara Carneiro^2^, Maria Mota^1^

###### ^1^Research Centre for Sports Sciences, Health Sciences and Human Development, University of Trás-os-Montes e Alto Douro, Vila Real, 5001-801, Portugal; ^2^Department of Sport Sciences, Exercise and Health, University of Trás-os-Montes e Alto Douro, Vila Real, 5001-801, Portugal; ^3^Polytechnic Institute of Setúbal, 2910-761, Portugal

####### **Correspondence:** Paulo V. João (pvicente@utad.pt) – Research Centre for Sports Sciences, Health Sciences and Human Development, University of Trás-os-Montes e Alto Douro, Vila Real, 5001-801, Portugal

Exercise is an important way of promoting health and quality of life of the elderly, retarding functional decline associated with aging which tends to be related to a more positive health perception. This study aimed to examine how some functionality indicators are associated with perception of health of the elderly living in rural areas involved in a municipal programme.

The sample was composed by two hundred and seventy-nine (279) subjects (237 females and 42 males, 72.0 ± 7.6 years; 28.8 ± 4.3 kg/m^2^), participating in the "Celorico a Mexer" municipal programme. Waist-hip ratio (WHR), blood pressure (BP), functional fitness and health status perception ("Current health" and "Health compared to others of the same age") was measured.

WHR was 0.96 ± 0.06, with women presenting the highest values (t = 10.902; p = 0.000). BP average was 136.7 ± 9.7 mmHg for SBP and 76.5 ± 10.4 mmHg in DBP. Considering Health Perception, 13.6 % of seniors feel that they have bad health, 29.0 % have poor health, 45.5 % reasonable health, 11.1 % good health and 0.7 % think it is very good.

Men consider their health significantly more positive than women (U = 3577.5, p = 0.002). Compared to other individuals of the same age, 17.6 % do not know, 17.6 % consider their health to be poorer, 48.2 % think their health identical and 16.5 % say they have better health. Men perceive their health better than women (U = 3821.5, p = 0.010). In functional capacity, women showed a better physical performance than men. It is necessary to develop campaigns for me for the maintenance of regular medical consulting, as well as the adoption of a better lifestyle.

**Keywords**

Autarchy, Functional capacity, Exercise, Elderly, Men, Women

#### O129 Medication adherence in patients with type 2 diabetes mellitus treated at primary health care in Coimbra

##### Rui Cruz^1^, Luiz Santiago^2^, Carlos Fontes-Ribeiro^3^

###### ^1^College of Health Technology of Coimbra, Polytechnic Institute of Coimbra, São Martinho do Bispo, 3046-854 Coimbra, Portugal; ^2^Universidade da Beira Interior, Covilhã, 6201-001, Portugal; ^3^Departamento de Farmacologia e Terapêutica, Faculdade de Medicina, Universidade de Coimbra, Coimbra, 3000-548, Portugal

####### **Correspondence:** Rui Cruz (ruic@estescoimbra.pt) – College of Health Technology of Coimbra, Polytechnic Institute of Coimbra, São Martinho do Bispo, 3046-854 Coimbra, Portugal

**Background**

The prevalence of diabetes mellitus is increasing globally and has become a major public health problem. In Portugal, the latest prevalence indicates more than 1 million people living with diabetes with aged between 20 and 79 years. Type 2 diabetes mellitus is more prevalent in 80 % to 90 % of cases. To prevent the complications associated with type 2 diabetes, therapy frequently also includes medications for control of blood pressure, dyslipidaemia and other disorders and it is essential that there is good adherence to the treatment regimen. Objective: To characterize the adherence to medication of patients with type 2 diabetes.

**Methods**

We developed a cross-sectional study in a sample of 357 patients from primary care centres in Coimbra. Data collection was conducted through the Morisky Green adapted questionnaire.

**Results**

In total, 160 men (44.8 %) and 197 women (55.2 %) participated in the study. The mean age was 67.48 (SD ± 9.5). We obtained good results of participation, with 88 % of people having high adherence, 10 % intermediate adherence and only 2 % poor adherence.

**Conclusions**

Our sample of diabetics has a high adherence to therapy. Despite these results we found some factors that interfere with adherence to therapy, including age, employment status and gender.

**Keywords**

Type 2 diabetes mellitus, adherence to medication, primary care

#### O130 Multivariate association between body mass index and multi-comorbidities in elderly people living in low socio-economic status context

##### Guilherme Furtado^1,2^, Saulo V. Rocha^3^, André P. Coutinho^4^, João S. Neto^4^, Lélia R. Vasconcelos^3^, Nelba R. Souza^2^, Estélio Dantas^5^

###### ^1^Fundação Capes, Ministério da Educação, 70.040-020, Brasília, Distrito Federal, Brasil; ^2^Faculdade de Ciências do Desporto e Educação Física, Universidade de Coimbra, 3040-248 Coimbra, Portugal; ^3^Universidade Estadual do Sudoeste da Bahia, Itapetinga, Bahia, 45700-000, Brasil; ^4^Universidade Federal de Santa Catarina, Florianópolis, Santa Catarina, 88040-900 Brasil; ^5^Universidade Tiradentes, Aracaju, Sergipe & Laboratório de Biociências da Motricidade Humana, 49030-620 Aracaju, Brazil

####### **Correspondence:** Guilherme Furtado (furts2001@yahoo.com.br) – Fundação Capes, Ministério da Educação, 70.040-020, Brasília, Distrito Federal, Brasil

**Background**

The increase in life expectancy has been accompanied by increased incidence of chronic diseases. The multi-comorbidity (MC) appears as a frequent condition in the population, especially in the elderly people. The epidemic of obesity seems to be the most significant aggravating factor of this population. Objective: To investigate the association between body mass index (BMI) and multi-comorbidities (MC) in elderly people living in rural and urban areas.

**Methods**

This is a cross-sectional study conducted in the city of Ibicuí-Bahia, Brazil. The sample consisted of n = 310 elderly participants (≥60 years), registered by the Family Health Strategy Program, living in the urban and rural contexts of the city, randomly selected, 56.5 % female and 43.5 % male (71.62 SD = 8.15 years). BMI was calculated through the standard formula. MC were identified by the individual's self-report and by consulting the medical file. In addition, we applied a biosocial questionnaire to obtain information about gender, age, marital status, family conditions and use of medication.

**Results**

Multiple linear regression analysis by gender revealed that only statistically significant associations emerged between “BMI”, “hypertension”, “coronary heart disease” and “hypercholesterolemia” in women (p ≤ 0.001). For the male gender, only “hypertension” is associated significantly with BMI (p ≤ 0.005).

**Conclusions**

Evidence suggests that the “BMI” has statistically significant associations with “cardio-metabolic diseases”, reinforcing its role as a predictor of cardiovascular risk and its association with MC’s, especially in elderly women of low socioeconomic status.

**Keywords**

Elderly, multi-comorbidities, body fat mass, BMI

#### O131 Metacognition, rumination and experiential avoidance in Borderline Personality Disorder

##### Alexandra Dinis, Sérgio Carvalho, Paula Castilho, José Pinto-Gouveia

###### Cognitive-Behavioural Research Centre, Faculty of Psychology and Education Sciences, University of Coimbra, 3001-802 Coimbra, Portugal

####### **Correspondence:** Alexandra Dinis (alexandra.m.b.dinis@gmail.com) – Cognitive-Behavioural Research Centre, Faculty of Psychology and Education Sciences, University of Coimbra, 3001-802 Coimbra, Portugal

**Background**

Borderline Personality Disorder (BPD) is essentially conceptualized as an emotion dysregulation disorder characterized by a persistent pattern of volatile interpersonal relationships, unstable self-identity, intense and labile affect, impulsivity and self-damaging behaviours. Many of the pervasive and severe behaviours present in BPD have been recently conceptualized as forms of experiential avoidance. Objectives: I) Explore if individuals with BPD are significantly different from individuals with Generalized Social Phobia (GSP) and individuals from the general population in several psychological processes (metacognition and rumination) and in negative affect. II) Test whether the impact of these psychological processes on negative affect occurs through experiential avoidance in the three samples under study.

**Methods**

The BPD sample consisted of 73 psychiatric outpatients with a mean age of 28.99 (SD = 7.61), GSP comprised 69 psychiatric outpatients with a mean age of 27.51 (SD = 7.79) and non-clinical sample included 49 individuals with a mean age of 31.31 (SD = 10.61).

**Results**

Multivariate analysis of variance showed that there is at least one significant difference between two or more samples in all studied variables. Path Analysis showed: a) an indirect effect of rumination on negative affect through experiential avoidance, both in BPD and GSP; b) an indirect effect of metacognition on negative affect through experiential avoidance in the three samples under study. Multi-group analysis indicated the invariance of the structural models analysed.

**Conclusions**

Rumination, metacognition and experiential avoidance are present in a higher degree in BPD. It is possible that experiential avoidance represents an emotional regulation function that might incorporate different pervasive cognitive processes, such as rumination and metacognition.

**Keywords**

Rumination, Metacognition, Experiential Avoidance, Borderline Personality Disorder, Generalized Social Phobia, Multivariate analysis of variance, Path Analysis

#### O132 Health issues in a vulnerable population: nursing consultation in a public bathhouse in Lisbon

##### Alexandra Sarreira-Santos^1,2^, Amélia Figueiredo^1,2^, Lurdes Medeiros-Garcia^1,2^, Paulo Seabra^1,2^

###### ^1^Escola Superior de Enfermagem de Lisboa, Lisboa, 1700-063, Portugal; ^2^Instituto de Ciências da Saúde, Universidade Católica Portuguesa, Lisboa, 1649-023, Portugal

####### **Correspondence:** Alexandra Sarreira-Santos (alexantos@ics.lisboa.ucp.pt) – Escola Superior de Enfermagem de Lisboa, Lisboa, 1700-063, Portugal

**Background**

The vulnerability, social exclusion, and mental disorder status identified in an exploratory study conducted in 2014, which characterized the public bathhouse users in the city of Lisbon, as well as their health status, led to the need for the creation, in March 2015, of a communitarian mental health nursing consultation. Objective: To analyse the needs for nursing care in the population that uses public bathhouse.

**Methods**

This is an exploratory, retrospective, descriptive and documental study. Documental analysis of clinical records from patients who were consulted from March 2015 to January 2016 (starting from one of the researcher’s analysis model).

**Results**

Twenty-seven patients are in clinical records and 47 consultations were carried out. We are dealing with a population with an average age of 52 years [26-84]; Gender – 16 (59.0 %) male and 11 (41.0 %) female; Marital status – 13 (48.0 %) single, 6 divorced, 4 married/consensual union, 2 widowers and 2 unknown; Employment status – 16 (59.0 %) unemployed, 6 retired, 3 (11.1 %) employees and 2 unknown; 12 (44.4 %) are homeless. In this nursing consultation the nursing diagnoses of greatest frequency in descending order were the following: Decreased psychological well-being, 17 (63.0 %); Hypertension, 14 (52.0 %); Reduced self-esteem and inefficient health behaviour, 11 (41.0 %); Use of substances, 8 (29.6 %); Anxiety, 8 (29.6 %).

**Conclusions**

The overview of nursing diagnoses confirms, as a result of vulnerability and social exclusion, evidence of mental suffering and difficulty to develop adaptive strategies.

**Keywords**

Vulnerability, nursing diagnosis, nursing consultation, mental suffering

#### O133 The perception of quality of life in people with multiple sclerosis accompanied in External Consultation of the Local Health Unit of Alto Minho

##### Rosa Rodrigues^1^, Mª Carminda Morais^1^, Paula O. Fernandes^2^

###### ^1^Escola Superior de Saúde, Instituto Politécnico de Viana do Castelo, 4900-347 Viana do Castelo, Portugal; ^2^Escola Superior de Tecnologia e Gestão, Instituto Politécnico de Bragança, 5300-253 Bragança, Portugal

####### **Correspondence:** Rosa Rodrigues (rmilir@hotmail.com) – Escola Superior de Saúde, Instituto Politécnico de Viana do Castelo, 4900-347 Viana do Castelo, Portugal

**Background**

Multiple Sclerosis (MS) is a chronic demyelinating, autoimmune, inflammatory and degenerative disease with an unpredictable development. Objectives: This study aims to assess the Quality of Life (QoL) of people with MS, analyse their sociodemographic and clinical profile and the relationship between these variables and QoL.

**Methods**

This is a cross-sectional study of correlational type, using quantitative methods. It presents perceived QoL as dependent variable and as independent variables sociodemographic and clinical profiles were considered. Data collection was done using a sociodemographic form and a QoL assessment tool related to health – MOS SF-36v2.

**Results**

Sixt-seven persons with MS are involved In this work, of both genders (82 % females) with a mean age ± standard deviation (SD) of 42.0 ± 11.7 years (between 20 and 71). The results show that the participants obtained higher scores of QoL is the Mental Health dimension (M = 58.96) and lowest in Vitality dimension (M = 42.07) of the SF-36v2. Statistically significant, moderate and negative differences between age and QoL were found in Physical Function (r = - .568, p ≤ .05) and Physical Performance (r = - .438, p ≤ .05).

**Conclusions**

The results show that older people have lower QoL scores in Physical Function and Physical Performance dimensions and that people with feelings of fatigue and loss of balance present low QoL, significantly lower in all the dimensions of the SF-36v2. Taken as a whole, the results of this study seem promising in terms of understanding the perception of QoL in people with MS.

**Keywords**

Aromatic and medicinal plants, Herbal drugs, Herbal products, Use

#### O134 Representation of interaction established between immigrant women and nurse during pregnancy to postpartum, from the perspective of immigrant women

##### Conceição Santiago^1^, Mª Henriqueta Figueiredo^2^, Marta L. Basto^3^

###### ^1^Escola Superior de Saúde de Santarém, Santarém, 2005-075, Portugal; ^2^Escola Superior de Enfermagem do Porto, Porto, 4200-072, Portugal; ^3^Escola Superior de Enfermagem de Lisboa, Lisboa, 1700-063, Portugal

####### **Correspondence:** Conceição Santiago (cfs65@gmail.com) – Escola Superior de Saúde de Santarém, Santarém, 2005-075, Portugal

**Background**

Health care in pregnancy/maternity in the host country tends not meet the expectations and health needs of immigrant women, justifying the need for more adjusted nursing care practices for the needs of this target group. Objective: To identify elements which characterize the interaction between immigrant women and nurses during prenatal care to postpartum, from the perspective of migrant women.

**Methods**

In an interpretative paradigm, using Grounded Theory, 30 semi-structured interviews were conducted with pregnant immigrant women (from several countries) to 6 months postpartum in Health Units, from February to March 2015, after approval by the Ethics Commission for Health ARSLVT.

**Results and conclusions**

From the results of the focused coding five categories emerged: the established communication between nurse-immigrant woman; the meaning attributed to nursing information/orientation about pregnancy/maternity; the emotional response to the behaviour and nursing interventions; the trust built between immigrant women and nurses; putting the nursing guidelines into practice. From a comprehensive analysis of the interrelation of these categories arose the recognition and safety of women in attitudes, and nursing interventions in relation to maternal and foetal well-being, information and adherence to health behaviours. But the host also needs acceptance, to be respected and understood during the interaction with the nurse. Regarding that this interaction is influenced by the situation of the pregnant woman/mother, with cultural characteristics and personality changes, adjustments and difficulties, these results will allow the continuation of this study, in order to build a comprehensive theory on the interaction between the nurses and migrant women.

**Keywords**

Migrant women, nursing care in pregnancy and postpartum, interaction

#### O135 Illness perceptions and medication adherence in hypertension

##### Teresa Guimarães, André Coelho, Anabela Graça, Ana M. Silva, Ana R. Fonseca

###### Escola Superior de Tecnologia da Saúde de Lisboa, Instituto Politécnico de Lisboa, 1549-020 Lisboa, Portugal

####### **Correspondence:** Teresa Guimarães (tguimaraes@estesl.ipl.pt) – Escola Superior de Tecnologia da Saúde de Lisboa, Instituto Politécnico de Lisboa, 1549-020 Lisboa, Portugal

**Background**

Arterial hypertension constitutes a major risk factor for the development of cardiovascular disease and cognitive impairment, which antihypertensive medication can prevent or minimize. Patients’ beliefs about their illness play an important role in blood pressure control, as they can determine behaviours that patients adopt to cope with their illness, namely adherence to antihypertensive medication. Objective: To identify patients’ perceptions of hypertension and assess associations between those beliefs and medication adherence.

**Methods**

63 hypertensive patients, 69.8 % females, aged 54-95 years (M = 69.02; SD = 10.07), 96.8 % of whom were diagnosed over a year previously and prescribed with antihypertensive medication completed the Revised Illness Perception Questionnaire (IPQ-R) and a 7-item medication adherence measure (*Medida de Adesão aos Tratamentos* – MAT).

**Results**

Most patients perceived their hypertension as a chronic and cyclical condition that can be controlled by their own behaviour and medication intake, and that elicits negative affective responses. Patients reported a high level of medication adherence (M = 5.41; SD = 0.55, with 7 as highest possible score) and a low frequency of non-adherent behaviours, with 20.6 % stating that they did not take medication because they forgot it. We found significant negative correlations between adherence and hypertension timeline (cyclical) (rs(63) = -0.27; p < 0.05), hypertension consequences (rs(63) = -0.50; p < 0.01) and emotional representations (rs(62) = -0.37; p < 0.01).

**Conclusions**

These findings, suggesting an association between illness perceptions and non-adherence behaviours in hypertension, strengthen the importance of patient-centred interventions starting from patients’ beliefs, preferences and needs, could lead to a better understanding of illness and enhancing patients’ active engagement in blood pressure control.

**Keywords**

Illness perceptions, medication adherence, hypertension, ageing

#### O136 A Portuguese study on adults’ intimate partner violence, interpersonal trust and hope

##### Luz Vale-Dias, Bárbara Minas, Graciete Franco-Borges

###### Faculdade de Psicologia e Ciências da Educação, Universidade de Coimbra, 3001-802 Coimbra, Portugal

####### **Correspondence:**Luz Vale-Dias (valedias@fpce.uc.pt) – Faculdade de Psicologia e Ciências da Educação, Universidade de Coimbra, 3001-802 Coimbra, Portugal

**Background**

According to results from the World Health Organization (2002), violence in intimate relationships is a phenomenon whose incidence covers the most diverse populations on a universal scale, with severe health consequences, including death. Being a complex problem, there is an increased need for research and prevention/intervention. Objectives: Considering previous research, this exploratory study aims to examine the relationship between intimate partner violence, interpersonal trust in the intimate partner and hope for the future. Also, the prevalence of violence in intimate relationships will be addressed.

**Methods**

The sample includes 302 subjects (202 women and 100 men), aged 18 to 63 years (M = 29, SD = 10.78). Data collection was performed through a sociodemographic questionnaire, the Portuguese adaptation of the Rotenberg´s Specific Trust Scale-Adults, Scale of Beliefs about Marital Violence, the Inventory of Marital Violence, and the Scale of the Future.

**Results**

Results show the existence of weak negative associations between interpersonal trust and violence. Although modest, some significant positive relationships between certain factors of hope and interpersonal trust were found. Age, gender and socioeconomic status play an interesting role. Results also reveal a worrying prevalence of violence in intimate relationships, whether in present or in past relationships.

**Conclusions**

In sum, the study characterizes the quality of intimate relationships, showing some negative associations of interpersonal trust with violence and positive associations of interpersonal trust with hope. Interpretation of these findings and limitations of the results as well as suggestions of its possible use in future research are also presented and discussed.

**Keywords**

Interpersonal trust, Hope, Intimate partner, Violence

#### P63 QOL’ predictors of people with intellectual disability and general population

##### Cristina Simões, Sofia Santos

###### Universidade de Lisboa, 1649-003 Lisboa, Portugal

####### **Correspondence:** Cristina Simões (cristina-ferreira@iol.pt) – Universidade de Lisboa, 1649-003 Lisboa, Portugal

**Background**

Quality of life (QOL) is a key outcome measure and is progressively being used in the intellectual disability (ID) field. There is an emerging body of evidence concerning personal-environmental characteristics influencing QOL. Personal factors with impact on QOL were identified as demographic (e.g., gender, age) and human functioning (e.g., IQ, adaptive behaviour) variables. Environmental factors involve perceived social supports, residential settings, inclusion, employment status, community interactions, geographical location, and organization culture. Identify predictors of QOL is crucial to know how these determinants can be used by practitioners to enhance personal outcomes and focus on personal-environmental interactions that influence the QOL of each individual. Our goal was to examine factors that influence QOL on people with and without ID. This information is important to meet challenges and overcome barriers that people with ID face. Two groups of characteristics were analysed, namely: personal (gender, diagnosis, age, health status) and environmental determinants (living arrangement, employment status, education level).

**Methods**

Data were collected from 1,929 Portuguese adults: 1,264 individuals with ID and 665 participants without ID. QOL was assessed by the “*Escala Pessoal de Resultados* (EPR”), the Portuguese version of the Personal Outcomes Scale (POS).

**Results**

Results supported that QOL is influenced by individual and environmental factors and the health status was the highest QOL’ predictor among all participants.

**Conclusions**

Findings encourage a strong work in Portugal, highlighting changes in policies and services based on the QOL framework. Furthermore, supports should enable the participation of adults with ID in community-based settings, recognising their personal talents.

**Keywords**

Quality of life, intellectual disability, general population, predictors

#### P64 Content validation of the Communication Disability Profile (CDP) - Portuguese Version

##### Ana Serra, Maria Matos, Luís Jesus

###### Universidade de Aveiro, Aveiro, 3810-193, Portugal

####### **Correspondence:** Ana Serra (acserra@ua.pt) – Universidade de Aveiro, Aveiro, 3810-193, Portugal

**Background**

The assessment of people with aphasia (PWA) should include the evaluation of specific disorders of language and the aphasia impact on their activities and their participation in the society. The Communication Disability Profile (CDP) has thus been translated and adapted to European Portuguese. Objective: CDP – Portuguese Version content validation.

**Methods**

An expert panel of eleven PWA was consulted. The adopted methodology (participatory workshops) fostered panel group integration, so that critical spirit and discussion could be enhanced. Data have been processed by a qualitative analysis programme.

**Results**

Concerning the Form, the expert panel was unanimous to consider that CDP is not suitable for Portuguese PWA and so part of the suggestions refer to adapting it to the Portuguese society and culture. In terms of Content, the panel considered the CDP’s Ambiguity and Relevance categories as valid, constituted by important items that reflect PWA’s reality, covering their needs. In the category of Clarity, CDP was considered clear but incomplete in the areas of Activities and Participation.

**Conclusions**

The CDP – Portuguese Version is incomplete in the areas mentioned above. However, the consequences of aphasia in terms of Activity, Participation, Context Factors and Emotions can be measured with it. The suggestions proposed by the expert panel have complemented the instrument gaps, having been integrated in a new version of the CDP.

**Keywords**

Communication Disability Profile, CDP, aphasia, aphasia assessment, people with aphasia

#### P65 Study of biochemical and haematological changes in football players

##### Ana S. Tavares, Ana Almeida, Céu Leitão, Edna Varandas, Renato Abreu, Fernando Bellém

###### Escola superior de tecnologia da saúde de Lisboa, 1990-096 Lisboa, Portugal

####### **Correspondence:** Ana Almeida (ana.almeida@estesl.ipl.pt) – Escola superior de tecnologia da saúde de Lisboa, 1990-096 Lisboa, Portugal

**Background**

Football players develop throughout their practice, an endurance-type exercise. This type of practice is known to cause changes both at the cellular level as well as on muscle enzymes. Modifications are also seen in terms of lipid profile, caused by the faster rate of metabolism of these molecules. The aim of this paper was to study the variation of biochemical and haematological parameters on football players.

**Methods**

Blood samples were drawn from 18 football players undergoing an 8 hour-a-week training program. The following parameters were tested: lipid profile, creatine kinase (CK), lactate dehydrogenase (LDH), aspartate aminotransferase (AST); red blood cells (RBC) and their indices, haemoglobin and haematocrit.

**Results**

The lipid profile showed that the HDL-Cholesterol mean (=53.33 mg/dL) was above the value determined as target (37.00 mg/dL). The LDL-Cholesterol mean was below the target-value, as it was for the triglycerides. There were no significant differences between the real mean and the target value for total cholesterol. All creatine kinase (CK) values were above the target-value of 77 UI/L. The values obtained for lactate dehydrogenase (LDH) and aspartate aminotransferase (AST) tests did not show any significant difference from target-values. No major differences from the target values were seen in terms of the haematological parameters.

**Conclusions**

The results of this study have shown that the practice of physical exercise has caused a modification in the subject's lipid profile, especially on the HDL-C values. Concerning muscle enzymes, the only altered value found was for creatine kinase (CK). The so called sports anaemia was not confirmed.

**Keywords**

Lipid profile, muscle damage, sports anaemia, athletes, football

#### P66 Body image dissatisfaction in inflammatory bowel disease: exploring the role of chronic illness-related shame

##### Inês A. Trindade, Cláudia Ferreira, José Pinto-Gouveia, Joana Marta-Simões

###### Cognitive and Behavioural Centre for Research and Intervention, Faculty of Psychology and Education Sciences, University of Coimbra, 3000-115 Coimbra, Portugal

####### **Correspondence:** Joana Marta-Simões (joana_simoes_@hotmail.com) – Cognitive and Behavioural Centre for Research and Intervention, Faculty of Psychology and Education Sciences, University of Coimbra, 3000-115 Coimbra, Portugal

**Background**

Inflammatory bowel disease (IBD) is characterized by a chronic, relapsing inflammation of the intestinal system which causes symptoms such as abdominal pain, bloating, diarrhoea, and weight loss. Although the majority of IBD patients experience body image dissatisfaction, which presents substantial implications for patients’ quality of life, the mechanisms associated with this link are not clearly understood. Objectives: The aim of this study was thus to explore the role of chronic illness-related shame in the association between IBD symptomatology and body image dissatisfaction, while controlling for age, BMI and surgery.

**Methods**

Participants included 161 adult IBD patients (52 males and 109 females), that reported demographic and medical data and completed self-report measures.

**Results**

Results from path analyses revealed that age and IBD symptomatology significantly predicted illness-related shame with effects of -.23 (p < .01) and .43 (p < .001), respectively. Further, IBD symptomatology presented a total effect of .43 on body image dissatisfaction: a direct effect of .23 (p < .001) and an indirect effect through the mechanisms of illness-related shame of .20 (C.I. from .13 to .29). The model explained 22 % of chronic illness-related shame and 38 % of body image dissatisfaction, and revealed an excellent model fit.

**Conclusions**

These findings suggest that higher IBD symptomatology leads to higher levels of body image dissatisfaction in IBD patients. Further, part of this effect seems to be mediated by the experience of chronic illness-related shame. Therefore, it seems that, to diminish IBD patients’ body image dissatisfaction, treatment programmes should address shame feelings and promote self-compassion abilities.

**Keywords**

Inflammatory Bowel Disease, chronic illness, body image dissatisfaction, chronic illness-related shame

#### P67 Obesity and sleep in the adult population - a systematic review

##### Odete Amaral^1^, Cristiana Miranda^1^, Pedro Guimarães^1^, Rodrigo Gonçalves^1^, Nélio Veiga^2^, Carlos Pereira^1^

###### ^1^Escola Superior de Saúde, Instituto Politécnico de Viseu, 3504-510 Viseu, Portugal; ^2^Universidade Católica Portuguesa, Viseu, 3504-505, Portugal

####### **Correspondence:** Odete Amaral (mopamaral@gmail.com) – Escola Superior de Saúde, Instituto Politécnico de Viseu, 3504-510 Viseu, Portugal

**Background**

Current scientific evidence has reported associations between inadequate sleep patterns and overweight/obesity. Epidemiological studies show a curvilinear relationship in "U-shape" between inadequate sleep and obesity, resulting in consequences on individual and public health levels. Objectives: To identify the association between overweight/obesity and inadequate sleep patterns among adults.

**Methods**

We conducted a systematic review of the literature, using a search in PubMed, Cochrane Library, SciELO and Google Scholar. We identified studies published between January 2008 and November 2015. Inclusion criteria were previously defined, then the selected studies were assessed for their quality and later analysed. By applying the "Scale to critically assess a paper describing a prospective, randomized and controlled clinical trial" in the included studies, it was found that of the four studies that formed the textual corpus, three were considered of quality (final grade ≥ 75 %).

**Results**

Inadequate sleep patterns increase the risk of weight gain and consequently of overweight and obesity in adults. This was a result supported by the data presented in the studies analysed (systematic literature review, longitudinal studies of clinical intervention, randomized and controlled trials, cross-sectional analytic studies). Overweight/obesity are associated with inadequate sleep-wake patterns, namely the "short" duration of sleep in adults.

**Conclusions**

An association is suggested between decreased sleep duration and increase in weight. Longitudinal and experimental studies, using objective and repeated measures of sleep, are important to define a causal relationship between sleep deprivation and obesity.

**Keywords**

Sleep, sleep deprivation, obesity, overweight, adults

#### P68 Frequency of daytime sleepiness and obstructive sleep apnea risk in COPD patients

##### Tânia C. Fleig, Elisabete A. San-Martin, Cássia L. Goulart, Paloma B. Schneiders, Natacha F. Miranda, Lisiane L. Carvalho, Andrea G. Silva

###### Universidade de Santa Cruz do Sul, Santa Cruz do Sul, Rio Grande do Sul, 96815-900, Brasil

####### **Correspondence:** Tânia C. Fleig (tcmfleig@gmail.com) – Universidade de Santa Cruz do Sul, Santa Cruz do Sul, Rio Grande do Sul, 96815-900, Brasil

**Background**

Chronic Obstructive Pulmonary Disease (COPD) is characterized by persistent and progressive airflow limitation associated with multisystem manifestations and comorbidities resulting in poor sleep quality or Obstructive Sleep Apnoea (OSA) with a prevalence of around 10 %. Objective: To identify the frequency of disorders sleep in COPD patients.

**Methods**

Cross-sectional study that evaluated 23 patients with COPD in the Programme of Santa Cruz Hospital. All were submitted to the Berlin questionnaire (BQ) to assess the probability of OSA and the Epworth Sleepiness Scale (ESS) that evaluates the possibility of dozing in eight everyday situations and establishes diagnosis of excessive daytime sleepiness (EDS). The results were expressed as mean ± standard deviation and analysed with Student’s t-test.

**Results**

Average age was 63.22 ± 8.4 years, body mass index (BMI) 26.3 ± 5.7 Kg/m^2^, 57 % male, expiratory volume forced in the 1st second 36.46 ± 16.12 % predicted and the classification of COPD as very severe (43.5 %). Frequency of EDS was 8.7 % (n = 02) and 22 % presented high probability of OSA (n = 05). The analysis of clinical variables in COPD stratified by high probability of OSA and absence of OSA showed no significant difference between groups, but there was an overweight trend in COPD with high probability of OSA (BMI 30.16 ± 6.31 Kg/m^2^ vs. 25.24 ± 5.24 Kg/m^2^, p = 0.08).

**Conclusions**

COPD patients presented a high probability of OSA compared to the literature, where the prevalence of this sleep disorder is around 10 %.

**Keywords**

COPD, Obstructive Sleep Apnea, Excessive daytime sleepiness

#### P69 Working with immigrant-origin clients: discourses and practices of health professionals

##### Joana Topa^1,2^, Conceição Nogueira^3^, Sofia Neves^1,2^

###### ^1^Instituto Superior da Maia, 4475-690 Avioso São Pedro, Portugal; ^2^Centro Interdisciplinar de Estudos de Género, Instituto Superior de Ciências Sociais e Políticas, 1300-663 Lisboa, Portugal; ^3^Faculdade de Psicologia e Ciências da Educação, Universidade de Coimbra, 3001-802 Coimbra, Portugal

####### **Correspondence:** Joana Topa (joana.topas.07@hotmail.com) – Instituto Superior da Maia, 4475-690 Avioso São Pedro, Portugal

**Background**

In Portugal there is a lack of scientific reflection on the maternal health care advocated for immigrant women [1]. Knowing that in 2009 were born 1,0350 children of resident foreign women in our country [2], emerges the need to understand not only the difficulties of immigrant populations on the access and use of services, but also the speeches, practices and challenges that health professionals face on provision of maternity care to these women.

**Methods**

The present study adopted a qualitative methodology for collecting (semi-structured interviews) and analysing the data (thematic analysis). This study was based on privileged information obtained from fourteen health professionals that provide care to pregnant immigrant women.

**Results and conclusions**

The data shows that health care professionals had misinformation about the immigrant legal rights and free access to maternal health services, they face many dilemmas in providing care to women who follow their cultural values, beliefs and perceptions especially if the behaviour portrayed does not conform to the norms of the health care system. When facing this situation, they know that they need to respond using a culturally sensitive approach by incorporating cultural competency into their practice but many times they don’t know how to do it. They also face communication difficulties specially when the immigrant women don’t speak or understand Portuguese language. Some of the interviewed showed discourses of stigmatization and regulation of knowledge in favour of biomedical knowledge. These speeches, practices and challenges can enhance the vulnerability of these women.

**References**

1. Dias S, Gama A, Silva A, Cargaleiro H, Martins M. Barriers in access and utilization of health services among immigrants: the perspective of health professionals. Acta Med Port. 2011; 24(4): 511-516.

2. Instituto Nacional de Estatística. Estatísticas demográficas 2010. Lisboa: INE; 2011.

**Keywords**

Immigration, Maternal Health Services, Health Professionals

#### P70 Systemic Lupus Erythematosus – what are audiovestibular changes?

##### Rita Ventura, Cristina Nazaré

###### College of Health Technology, Polytechnic Institute of Coimbra, São Martinho do Bispo, 3046-854 Coimbra, Portugal

####### **Correspondence:** Rita Ventura (ritaventura_91@hotmail.com) – College of Health Technology, Polytechnic Institute of Coimbra, São Martinho do Bispo, 3046-854 Coimbra, Portugal

**Background**

Systemic Lupus Erythematosus (SLE) is an autoimmune disease that can develop auditory and vestibular disorders, explained by a change associated with the autoimmune disease and justified by vasculitis in the cochlea which may involve the striavascularis, the spiral ligament, or internal auditory artery, or immune complexes, which instead of vasculitis, are the cause of minor stroke of the capillaries or arterioles in the temporal bone. Objective: To study auditory and vestibular disorders in individuals with SLE and identify the variables in SLE disease that could justify possible audiovestibular changes.

**Methods**

A literature review of articles since 2007 was performed with a search made in scientific databases as Elsevier, B-on, Medline, PubMed and Google Scholar using the keywords “autoimmune disease, lupus, systemic lupus erythematous, auditory system, vestibular system, hearing loss, vertigo, dizziness” in English and in Portuguese. After the search strategies, five articles in accordance with pre-established inclusion criteria were selected.

**Results**

Audiovestibular tests performed in patients with SLE indicated bilateral sensorineural hearing loss with the highest incidence in the high frequencies and unilateral vestibular disorders. It should be noted that vertigo and tinnitus were the most commonly reported symptoms and duration of the disease appears to influence the integrity of the auditory and vestibular system.

**Conclusions**

By the analysis of articles was possible to conclude that the involvement of the auditory and vestibular system should be considered as one of the elements of the clinical picture of SLE, and the matter should be explored by the scientific community.

**Keywords**

Systemic Lupus Erythematosus (SLE), Autoimmune disease, Auditory system, Vestibular system, hearing loss, vertigo

#### P71 Mental disorders in the oldest old: findings from the Portuguese national hospitalization database

##### Daniela Brandão^1,2^, Alberto Freitas^2,3^, Óscar Ribeiro^1,4^, Constança Paúl^1,3^

###### ^1^Unidade de Investigação e Formação sobre Adultos e Idosos, Instituto de Ciências Biomédicas Abel Salazar, Universidade do Porto, 4050-313 Porto, Portugal; ^2^Faculdade de Medicina da Universidade do Porto, 4200-450 Porto, Portugal; ^3^Center for Health Technology and Services Research, Faculdade de Medicina, Universidade do Porto, 4200-450 Porto, Portugal; ^4^Universidade de Aveiro, 3810-193 Aveiro, Portugal e Instituto Superior De Serviço Social Do Porto, 44600-362 Sra. da Hora, Portugal

####### **Correspondence:** Daniela Brandão (daniela.brandao@unifai.eu) – Unidade de Investigação e Formação sobre Adultos e Idosos, Instituto de Ciências Biomédicas Abel Salazar, Universidade do Porto, 4050-313 Porto, Portugal

**Background**

Mental health problems have been reported as one of the principal causes of incapacity and morbidity. According to the World Health Organization, approximately 15 % of adults aged 60 and over at a global level suffer from a mental disorder, and dementia and depression are the most common neuropsychiatric disorders in this age group. In the oldest old population, a higher deterioration is expected, which is thought to increase the risk of incidence of mental problems. Objectives: The aim of this study is to examine inpatient episodes with a mental disorder as primary diagnosis between 2000 and 2014 in very old patients in mainland Portugal.

**Methods**

All inpatient episodes of hospital admissions by patients aged 80 years and older due to mental disorders during the study period were considered. Exploratory descriptive analyses of data regarding the number and kind of mental disorder diagnosis on admission were performed.

**Results**

A total of 16,430 inpatient episodes were analysed. Delirium, dementia and amnestic and other cognitive disorders (60.1 %), alcohol-related disorders (17.7 %) and mood disorders (8.6 %) were the most common diagnoses. An analysis by age group revealed that in octogenarians and nonagenarians, delirium, dementia and amnestic and other cognitive disorders were the most common diagnoses, but in centenarians these were outweighed by alcohol-related disorders.

**Conclusions**

Findings from this study document the high rate of dementia as a primary reason for hospitalization in the oldest old and corroborate previous data regarding mental health in the older population in general. Further studies should examine the prevalence of medical comorbidities in patients with mental disorders.

**Keywords**

oldest old, mental disorders, inpatient episodes

#### P72 Recurrence analysis in postural control in children with cerebral palsy

##### Cristiana Mercê, Marco Branco, Pedro Almeida, Daniela Nascimento, Juliana Pereira, David Catela

###### Escola Superior de Desporto de Rio Maior, Instituto Politécnico de Santarém, 2040-413 Rio Maior, Portugal

####### **Correspondence:** Cristiana Mercê (cristianamerce@esdrm.ipsantarem.pt) – Escola Superior de Desporto de Rio Maior, Instituto Politécnico de Santarém, 2040-413 Rio Maior, Portugal

**Background**

Cerebral palsy (CP) is the most common motor disorder among children [1]. The aim of the study consists of analysing and comparing the postural control in children with CP during sitting position in the following tasks: I) simply sitting (baseline); II) observing the modelling of a plasticine ball; III) molding a plasticine ball; and IV) idem III) with closed eyes.

**Methods**

Five children of both sexes were analysed, with 12 ± 1 years old, and CP levels I and II [2]. Data were obtained by filming at 240 Hz in vertex and C7 points, for kinematic (Kinovea) and recurrence analysis [3].

**Results and conclusions**

Compared to the condition I), in condition II) children were more deterministic, periodic and complex but less stable. The focus on movements implied a reorganization of postural control. In conditions III) and IV) children were less deterministic, periodic, stable and complete at C7, but more periodic, stable and complex at vertex, probably due to the need to stabilize the head. When performing a functional motor task (conditions III and IV), children changed their pattern of postural control, with likely increase in degrees of freedom, anchored in stabilizing the head.

The introduction of a visual focus (condition II) caused a postural adjustment probably resulting from the activity of mirror neurons. When performing a functional task (conditions III and IV) the postural adjustment was anchored in fixing the head and trunk release.

**References**

1. Herskind A, Greisen G, Nielsen JB. Early identification and intervention in cerebral palsy. Dev Med Child Neurol. 2015; 57(1): 29-36.

2. Eliasson A, Krumlinde-Sundholm L, Rosblad B, Beckung E, Arner M, Ohrvall A, Rosenbaum P. The Manual Ability Classification System (MACS) for children with cerebral palsy: scale development and evidence of validity and reliability. Dev Med Child Neurol. 2006; 48(7): 549-554.

3. Webber C, Zbilut J. Recurrence Quantification Analysis of Nonlinear Dynamical Systems. In: Tutorials in Contemporary Nonlinear Methods for the Behavioral Sciences. Arlington: National Science Foundation. 2005. p. 26-94.

**Keywords**

Recurrence analysis, Postural control, Children, Cerebral Palsy

#### P73 The experience of self-care in the elderly with COPD: contributions to reflect proximity care

##### Helga Rafael (hrafael@esel.pt)

###### Escola Superior de Enfermagem de Lisboa, Lisboa, 1700-063, Portugal

**Background**

Chronic Obstructive Pulmonary Disease (COPD) leads to changes in different areas of life and poses several challenges in self-care. It presents a resource for elderly people to remain in their own homes, while containing an important source of knowledge of personal identity. We aim to understand the importance of the home in self-care management in elderly patients living with COPD and to put proximity care in perspective.

**Methods**

It follows a phenomenological-hermeneutic approach, based on 16 interviews conducted with persons between 65 and 100 years old. The narratives were interpreted in accordance with Paul Ricoeur’s Interpretation Theory, following the stages of naive understanding, structural analysis and comprehensive understanding.

**Results and conclusions**

One of the themes that emerged was "Limiting the living space to home". Through this experience, elderly people lived in discontinuity and continuity. Home is linked to the experience of discontinuity of self-care when it is felt like a limited space, of closure. Home is linked to the experience of continuity when it means (re)construction of personal identity, ability to preserve, acquire or adapt self-care habits, maintaining integrity. The elderly interact dynamically with their houses, in a movement between opposing feelings.

For these participants home is a very important place of care. Policy guidelines should include proximity care, patient-centred care or care in their homes, for as long as possible, giving priority to the preservation of identity elements of each. Self-care experience must be lived with congruence with the individual health design and continuity with integrity, personal identity and human dignity.

**Keywords**

Self-care, Elderly, Patient-Centred Care, Chronic Obstructive Pulmonary Disease

#### P74 Culturally competent nurses: managing unpredictability in clinical practice with immigrants

##### Alcinda C. Reis (alcinda.reis22@gmail.com)

###### Escola Superior de Saúde, Instituto Politécnico de Santarém, 2005-075 Santarém, Portugal

**Background**

In this presentation we will feature the process of building cultural competence in nursing, from the study of the clinical practice with immigrants in primary health care contexts, in Portugal.

**Methods**

We developed an inductive study of ethnographic orientation, with participant observation; analysis was developed of discursive material of ethnobiographical interviews and narratives of 52 nurses and immigrants, and two focus groups with these different types of participants – at different moments.

**Results**

We identified difficulties in cultural contextualization of people cared for by nurses, more visible at the level of knowledge of particularities in the communication process, in the use of family members as interpreters, diversity of religious beliefs and about health and illness, roles and socio-familial control.

**Conclusions**

Regardless of the individual motivation of nurses to care for immigrants, they have the capacity for adequate management of unpredictability in cultural encounters. This management emerges as a central element of the professional development and of the construction of cultural skills – in a gradual distancing of nurses from their own ethnocentrism. However, the management of unpredictability is made especially with an "advance” for initial assessment and care planning, in accordance with the professional standards and dominant organizational culture, and sometimes there is a cultural misfit. The management of unpredictability in successive moments promotes adjustments in clinical practice with immigrants; it occurs together with a process of awareness by professionals, exploring how to experience the health-illness transitions in different ways.

**Keywords**

Cultural competency, Nurses, Immigrants, Clinical practice

### Health Instruments & Indicators

#### O137 **Paediatric speech and language screening: An instrument for health professionals**

##### Ana Mendes^1,2^, Ana R. Valente^2,3^, Marisa Lousada^4^

###### ^1^School of Health Sciences, Polytechnic Institute of Setúbal, 2910-761 Setúbal, Portugal; ^2^Institute of Electronics and Informatics Engineering of Aveiro, 3810-193 Aveiro, Portugal; ^3^Department of Education, University of Aveiro, 3810-193 Aveiro, Portugal; ^4^Center for Health Technology and Services Research & School of Health Sciences, University of Aveiro, 3810-193 Aveiro, Portugal

####### **Correspondence:** Marisa Lousada (marisalousada@ua.pt) – Center for Health Technology and Services Research & School of Health Sciences, University of Aveiro, 3810-193 Aveiro, Portugal

**Background**

To develop and validate a speech and language screening instrument (*Rastreio de Linguagem e Fala*, RALF), which aims to quickly identify children who may be at risk of a speech-language disorder and need to be referred to a detailed speech and language assessment.

**Methods**

A sample of 203 Portuguese children (n = 109 males, 94 females) aged 3.0 to 5.11 were recruited. 133 presented a typical language development and 70 a primary language disorder. Speech-language assessments and diagnostics were performed by a licensed and certified speech and language therapist. Subject selection criteria for disordered children included: I) language score below -1.5 SD, the mean of the Language Test-ALPE (*Teste de Linguagem* – TL-ALPE), a standardized language instrument; II) speech sound disorder identified through a spontaneous speech sample; III) absence of another condition such as hearing impairment, emotional or behavioural difficulties, autism, neurological impairment or general developmental difficulties. RALF and TL-ALPE’s scores were crossover. Sensibility as well as specificity were calculated for three age groups (3, 4 and 5 years).

**Results**

Sensibility values were 95 %, 96 %, 83 % and specificity values were 85 %, 84 % 71 % (for 3, 4 and 5 age groups, respectively). Results revealed that RALF was able to discriminate typically developing children from disordered speech-language developing children.

**Conclusions**

RALF is a valid instrument that can be used by health professionals to assist on the screening and identification of children who may need a speech and language detailed assessment.

**Keywords**

Screening, speech and language development, validity, children

#### O138 Anthropometric and nutritional assessment in bodybuilders

##### Diana Sousa, Ana L. Baltazar, Mª Helena Loureiro

###### Escola Superior de Tecnologia da Saúde de Coimbra, São Martinho do Bispo, 3046-854 Coimbra, Portugal

####### **Correspondence:** Diana Sousa (dianasousa18@hotmail.com) – Escola Superior de Tecnologia da Saúde de Coimbra, São Martinho do Bispo, 3046-854 Coimbra, Portugal

**Background**

In sport, nutrition must ensure a supply of nutritional requirements, to provide sufficient calories to supply the high energy costs associated with daily sports, either in training or in competition. They must deal with the high nutritional requirements of the athletes, promoting and maintaining a high level of physical and mental welfare, so that the athlete can engage in his sports discipline.

The aim of this study is to characterize the nutritional habits and body composition of bodybuilding practitioners in order to obtain muscle hypertrophy.

**Methods**

These individuals were selected from the gyms of São Martinho do Bispo, in Coimbra. The sample was composed by 17 male athletes, aged between 19 and 39 years. Nutritional assessment was performed using the food frequency questionnaire (FFQ), supplied by the Department of Hygiene and Epidemiology, Faculty of Medicine of Porto, and also by the 24-hour questionnaire. Body composition was determined through skin folds, weight, waist circumference and height of the individual.

Statistical analyses were performed using the SPSS statistical programme (Statistical Package for the Sciences), version 19.0.

**Results and conclusions**

Data analysis concluded that athletes have a high protein intake with a low consumption of carbohydrates. However, they have an energy and fat intake within the range recommended by the literature.

**Keywords**

Nutrition, sports, bodybuilding, hypertrophy

#### O139 Computerized adventitious respiratory sounds in children with lower respiratory tract infections

##### Ana Oliveira^1^, José Aparício^2^, Alda Marques^1^

###### ^1^School of Health Sciences, University of Aveiro, 3810-193 Aveiro, Portugal; ^2^Pediatrics Emergency Department, Hospital Lusíadas, Porto, 4050 113 Porto, Portugal

####### **Correspondence:** Alda Marques (amarques@ua.pt) – School of Health Sciences, University of Aveiro, 3810-193 Aveiro, Portugal

**Background**

The objective assessment of lower respiratory tract infections (LRTI) in children is challenging. Computerized adventitious respiratory sounds (ARS) are valuable to assess adult respiratory diseases; however, its application in children with LRTI is unknown. Objective: This cross-sectional study explored ARS potential to identify a LRTI and monitor its severity in children aged less than years.

**Methods**

Twenty-two (22) children aged ≤ 2 years (G1: 11 healthy; G2: 11 LRTI) and 18 aged 3-5 years (G3: 9 healthy; G4: 9 LRTI) were recruited from three healthcare institutions. Respiratory distress was assessed with the modified Wang Score. Computerized respiratory sounds were recorded following the international guidelines. Wheeze (occupation rate and frequency) and crackle (mean number and two cycle duration -2CD) parameters were analysed per respiratory phase using developed algorithms. Comparisons were established using Mann-Whitney and correlations with Spearman’s tests.

**Results**

Children with LRTI presented a higher expiratory wheeze occupation rate [G1: 2.15 (1.67-3.11) vs. G2: 4.73 (2.59-9.31) p = 0.001; G3: 2.80 (1.89-5.16) vs. G4: 5.17 (2.64-18.63) p = 0.07] and more inspiratory crackles [G1: 0.25 (0.14-0.44) vs. G2: 0.52 (0.22-0.92); p < 0.001; G3: 0.50 (0.25-0.72) vs. G4: 0.70 (0.33-1.55) p = 0.03] than healthy children. No differences were found for other ARS parameters. Moderate to strong correlations were found between ARS and children’s respiratory distress (0.35 < r < 0.51; p < 0.05).

**Conclusions**

Wheeze occupation rate and mean number of crackles were the parameters that most differed between healthy children and children with LRTI and that most correlated with the respiratory distress score. Those parameters may be useful to objectively diagnose and monitor children with LRTI.

**Keywords**

Lower respiratory tract infections, computerized respiratory sounds, paediatrics

#### O140 Role of computerized respiratory sounds as a marker in LRTI

##### Alda Marques^1^, Ana Oliveira^1^, Joana Neves^2^, Rodrigo Ayoub^2^

###### ^1^School of Health Sciences, University of Aveiro, 3810-193 Aveiro, Portugal; ^2^Centro Hospitalar do Baixo Vouga, Aveiro, 3814-501, Portugal

####### **Correspondence:** Alda Marques (amarques@ua.pt) – School of Health Sciences, University of Aveiro, 3810-193 Aveiro, Portugal

**Background**

Computerized respiratory sounds, especially adventitious respiratory sounds (ARS), have been increasingly used to monitor lower respiratory tract infections (LRTI). Although this measure has shown to be reliable in patients with stable chronic diseases, its variability and reliability in acute conditions is unknown. This cross-sectional study assessed the variability and reliability of computerized respiratory sounds in patients with LRTI.

**Methods**

Ninety-seven patients with LRTI (57 females; 54.82 ± 17.18 years) were recruited from one central hospital. Three repeated respiratory sound recordings were taken simultaneously from seven anatomical locations. Normal respiratory sound (NRS) intensity, mean number of crackles and wheeze occupation rate (Wh%) were analysed with validated algorithms. Intra-subject reliability was assessed with the intraclass correlation coefficient (ICC 1.3) and Bland-Altman plots. Inter-subject variability was assessed with the coefficient of variation (CV).

**Results**

Relative reliability was moderate to excellent for NRS intensity and mean number of crackles (0.42 < ICC < 0.80), except at trachea (0.29 < ICC < 0.74), and poor to excellent for Wh% (0.18 < ICC < 0.86). Absolute reliability demonstrated no systematic bias between measures. Inter-subject variability was acceptable for NRS intensity (14 % < CV < 25 %) and high for crackle and wheeze parameters (49 % < CV < 211 %).

**Conclusions**

NRS intensity seems to be the most reliable marker to monitor patients with LRTI. More research is needed with controlled flows and volumes to confirm these results since NRS are always produced during breathing whilst ARS are superimposed events on NRS hence, timing may not be perfectly repeatable from breath to breath.

**Keywords**

Computerized respiratory sounds, lower respiratory tract infections, variability, reliability

#### O141 Confirmatory factor analysis of the Personal Wellbeing Index in people with chronic kidney disease

##### Luís Sousa^1^, Cristina Marques-Vieira^2^, Sandy Severino^1^, Helena José^3^

###### ^1^Hospital Curry Cabral, Centro Hospitalar Lisboa Central, 1069-166 Lisboa, Portugal; ^2^Instituto de Ciências da Saúde, Universidade Católica Portuguesa, Lisboa, 1649-023, Portugal; ^3^Centro de Formação Multiperfil, Luanda, PR - 56Q, Angola

####### **Correspondence:** Luís Sousa (luismmsousa@gmail.com) – Hospital Curry Cabral, Centro Hospitalar Lisboa Central, 1069-166 Lisboa, Portugal

**Background**

The Personal Wellness Index/Satisfaction with life in general (PWI/SLG) is a questionnaire designed to measure subjective well-being (SWB). The PWI was based on the Comprehensive Quality of Life Scale [1] and is validated for the Portuguese population [2]. Objective: To confirm the unifactorial structure of the "Personal Wellbeing Index" in people with chronic kidney disease (CKD) on hemodialysis.

**Methods**

The random sample included 159 people with CKD on hemodialysis in a Nephrology Service and two clinics in the region of Lisbon, Portugal. Data collection was carried out between March and June 2015. The AMOS software was used for a confirmatory factor analysis (CFA), with the maximum likelihood method. The adjustment ratios were used: ratio Chi Square and the degrees of freedom (X^2^/gl); goodness-of-fit index (GFI); comparative fit index (CFI), Tucker-Lewis index (TLI) and root mean square error of approximation (RMSEA) [3-4].

**Results**

The results of this study [X^2^/df = 1.871; GFI = 0.96; CFI = 0.97; TLI = 0.95; RMSEA = 0.07] show a good fit to the hypothesis of the solution of a factor, which confirms the solution proposed in the original version [1] and the Portuguese version [2].

**Conclusions**

The Portuguese version of "Personal Wellbeing Index in people with CKD has a single factor. The personal well-being Index or satisfaction with life in general is suitable for measuring the impact of interventions in nursing in people with CKD.

**References**

1. Cummins RA, McCabe MP, Romeo Y, Gullone E. The Comprehensive Quality of Life Scale: Instrument development and psychometric evaluation on tertiary staff and students. Educ psychol measur. 1994; 54: 372-382.

2. Ribeiro JP, Cummins R. O bem-estar pessoal: estudo de validação da versão portuguesa da escala. In: Leal I, Pais-Ribeiro J, Silva I, Marques S, Editors. Actas do 7° Congresso Nacional de Psicologia da Saúde. Lisboa: ISPA; 2008. 505-508.

3. Marôco J. Análise de equações estruturais: fundamentos teóricos, software e aplicações. Pero Pinheiro: ReportNumber. 2010

4. Sousa LMM, Marques-Vieira CMA, Carvalho ML, Veludo F, José, HMG. Fidelidade e validade na construção e adequação de instrumentos de medida. Enformação. 2015; 5:25-32.

**Keywords**

Renal Insufficiency Chronic, Renal Dialysis, Quality of life, Validation studies, Psychometry

#### O142 Phonological awareness skills in school aged children

##### Inês Cadorio^1^, Marisa Lousada^1,2^

###### ^1^Center for Health Technology and Services Research, University of Aveiro, Aveiro, 3810-193 Aveiro, Portugal^; 2^School of Health Sciences, University of Aveiro, 3810-193 Aveiro, Portugal

####### **Correspondence:** Inês Cadorio (inescadorio@ua.pt) – Center for Health Technology and Services Research, University of Aveiro, Aveiro, 3810-193, Portugal

**Background**

Phonological awareness development is crucial in the beginning of the schooling process, as it is a key precursor of reading and writing skills. However, data on phonological awareness development of European Portuguese (EP) school aged children are scarce. Objective: The current study aimed to determine which phonological skills are acquired in school aged children (6.00 - 6.11 years) and whether there are differences between phonological awareness levels or not.

**Methods**

A total of 80 EP typically developing children aged between 6.00 and 6.11 years participated in this study. We used a valid and standardized instrument – Language Test: Pre-school Language Assessment (TL-ALPE) to collect data on phonological awareness skills. Descriptive statistics were used to determine which skills were acquired in the age range considered. Furthermore, Wilcoxon test for paired samples was performed to detect differences between syllabic awareness and phonemic awareness performance, as the sample data did not follow a normal distribution.

**Results**

Syllable identity is considered an acquired and established skill, as children showed 86.88 % accuracy in this task. Syllabic segmentation as well as phoneme identity are acquired but not established, with 68.75 % and 71.25 % accuracy, respectively. The lowest scores were achieved in phonemic segmentation (15.25 % accuracy) and this skill was considered non-acquired by the age of 6 years old. Significant differences were found between syllabic awareness and phonemic awareness levels (p = 0.000), the first presenting advantage over the second.

**Conclusions**

The present study is a valuable contribution, as it expands the literature on school aged children’s phonological awareness development.

**Keywords**

Syllabic awareness, phonemic awareness, typical development, children

#### O143 Assessment of early memories of warmth and safeness in interaction with peers: its relationship with psychopathology in adolescence

##### Marina Cunha, Diogo Andrade, Ana Galhardo, Margarida Couto

###### Instituto Superior Miguel Torga, Coimbra, 3000-132, Portugal

####### **Correspondence:** Marina Cunha (marina_cunha@ismt.pt) – Instituto Superior Miguel Torga, Coimbra, 3000-132, Portugal

**Background**

Given that the growing autonomy towards parents and the interaction with peers are particularly important aspects concerning the development of self-identity, it is crucial to assess early memories associated with feelings of safeness or threat in the interaction with peers. Objectives: I) To examine the psychometric characteristics of the Early Memories of Warmth and Safeness Scale in interaction with peers (EMWSS- Peers); II) to explore the relationship between EMWSS-Peers and the global measure of early positive memories with attachment figures (EMWSS); III) to investigate the impact of positive emotional memories in depressive and anxiety symptoms.

**Methods**

Three hundred fifty-five (355) adolescents (152 boys), aged between 12 and 18 years old, completed the Early Memories of Warmth and Safeness Scale (EMWSS – global measure), EMWSS in interactions with peers, and the Depression, Anxiety and Stress Scales (DASS-21).

**Results**

The EMWSS-Peers showed a single-factor structure, excellent internal consistency (α = .93), and test-retest reliability (r = .86). Memories of warmth and safeness regarding interaction with peers showed moderate negative correlations with anxiety and depressive symptoms, and a moderate positive association with warmth and safeness memories concerning the interaction with attachment figures. Additionally, early positive memories revealed a significant and independent contribution for the prediction of anxiety and depressive symptoms.

**Conclusions**

The EMWSS-Peers proved to be a reliable and valid instrument for the assessment of warmth and safeness feelings in the interaction with peers’ context. These results suggest that positive emotional memories can act as protective factors regarding psychopathological symptoms.

**Keywords**

Adolescence, self-report instrument, early emotional memories within peers

#### O144 The molecular effects induced by single shot irradiation on a diffuse large B cell lymphoma cell line

##### Fernando Mendes^1^, Cátia Domingues^2^, Susann Schukg^3^, Ana M. Abrantes^4^, Ana C. Gonçalves^5^, Tiago Sales^4^, Ricardo Teixo^4^, Rita Silva^4^, Jéssica Estrela^1^, Mafalda Laranjo^4^, João Casalta-Lopes^6^, Clara Rocha^1^, Paulo C. Simões^6^, Ana B. Sarmento-Ribeiro^7^, Mª Filomena Botelho^4^, Manuel S. Rosa^8^

###### ^1^Escola Superior de Tecnologia da Saúde de Coimbra, São Martinho do Bispo, 3046-854 Coimbra, Portugal; ^2^Centre of Investigation in Environment, Genetics and Oncobiology, Faculty of Medicine, University of Coimbra, 3000-548 Coimbra, Portugal; ^3^Biomedical Laboratory Sciences Department, University of Gothenburg, S-405 30 Gothenburg, Sweden; ^4^Biophysics and Biomathematics Institute, Institute for Biomedical Imaging and Life Sciences, Faculty of Medicine, University of Coimbra, 3000-354 Coimbra, Portugal; ^5^Oncobiology and Haematology Laboratory of Applied Molecular Biology & Clinical University of Hematology, Faculty of Medicine, University of Coimbra, 3000-354 Coimbra, Portugal; ^6^Radiation Oncology Department, Coimbra Hospital and Universitary Centre, 3000-075 Coimbra, Portugal; ^7^Clinical University of Hematology, Coimbra Hospital and Universitary Centre, 3000-075 Coimbra, Portugal; ^8^Imunology Institute, Faculty of Medicine, University of Coimbra, 3000-354 Coimbra, Portugal

####### **Correspondence:** Fernando Mendes (fjmendes@estescoimbra.pt) – Escola Superior de Tecnologia da Saúde de Coimbra, São Martinho do Bispo, 3046-854 Coimbra, Portugal

**Background**

An estimate of 386,000 cases of non-Hodgkin Lymphoma (NHL) occurred in 2012 (2.7 % of all cancers). Diffuse large B cell lymphoma (DLBCL) is a heterogeneous group of haematological malignancies and forms the most common type of aggressive non-Hodgkin lymphoma. Objective: To evaluate cellular and molecular effects of high doses X radiation in DLBCL cells.

**Methods**

Farage cells were exposed to 0.5-60 Gy of Ionizing Radiation (IR). Cell viability and proliferation were assessed by trypan blue assay and cell survival by clonogenic assay. Cell death was assessed by flow cytometry (FC) and optical microscopy. Cell cycle, mitochondrial membrane potential, reactive oxygen species, GSH and BAX/BCL-2 were measured by FC. DNA damage was evaluated using comet assay. Total and phosphorylated P53 was assessed by western blot.

**Results**

IR induced cytotoxic and cytostatic effects in Farage cells in a dose and time dependent manner with an LD50 of 1.73 Gy. Cell death occurs mainly by apoptosis with an increase in BAX/BCL-2 ratio and significant increase in Reactive Oxygen Species (ROS) production. We observed cell cycle arrest at G2/M phase and significant increase in DNA damage and in P53 total and phosphorylated expression levels.

**Conclusions**

High doses of IR induce a time dose dependent response which leads to increased ROS production, DNA damage with increased P53 expression and activation expressed by elevated pP53 levels, resulting in G2/M cell cycle arrest and increased later apoptosis/necrosis cell death. Our results showed that single shot IR induces effects in different cell components and its comprehension is essential to choose treatment planning.

**Keywords**

Large diffuse B cell lymphoma, Radiotherapy, Genotoxicity, Oxidative stress, Cell death, P53

#### O145 Morpho-functional characterization of cardiac chambers by Transthoracic Echocardiography, in young athletes of gymnastics competition

##### Virgínia Fonseca, Diogo Colaço, Vanessa Neves

###### Escola Superior de Tecnologia da Saúde de Lisboa, Instituto Politécnico de Lisboa, Lisboa, 1549-020 Lisboa, Portugal

####### **Correspondence:** Virgínia Fonseca (virginia.fonseca@estesl.ipl.pt) – Escola Superior de Tecnologia da Saúde de Lisboa, Instituto Politécnico de Lisboa, Lisboa, 1549-020 Lisboa, Portugal

**Background**

Prolonged intensive physical training causes structural and functional cardiovascular adaptations, however these modifications can be misinterpreted as pathological. The aim of this study was to describe the cardiovascular structure and functionality, by transthoracic echocardiography, in young competitive artistic gymnastics athletes.

**Methods**

This study was conducted in a population of feminine artistic gymnastics athletes, with ages between 12 and 15 years old. All individuals underwent transthoracic echocardiography, following the defined study protocol. Statistical analysis of the study variables was made using descriptive statistics, Shapiro-Wilk test, T-test, Wilcoxon's test and Pearson's correlation. The results were considered statistically significant when p value < 0.05.

**Results and conclusions**

With regard to left cardiac structure and functionality, there were no statistically significant changes, however the athletes had larger left atrial area (15.35 cm^2^), which is common due to pressure increase during exercise practice, especially in a combined isometric and isotonic training.

In the diastolic function, the mitral E wave velocity (1.08 m/s) and the mitral E/A ratio (2.31) were significantly higher. There was a correlation between years of practice and both mitral A wave velocity and mitral E/A ratio. These findings suggest a supernormal diastolic function as a consequence of the left ventricular relaxation and compliance enhancement.

With this study it was possible to verify, that all variables used to analyse structural and cardiovascular functional adaptations, measured by transthoracic echocardiogram, were within normal values, taking in consideration the paediatric and adult Guidelines. There were found statistically significant differences for the left atrial area and left diastolic function.

**Keywords**

Transthoracic echocardiography, athlete's heart, isometric training, isotonic training

#### O146 Prevalence of the antibodies of the new histo-blood system – FORS system

##### Carlos Jesus^1^, Camilla Hesse^2^, Clara Rocha^3^, Nádia Osório^1^, Ana Valado^1^, Armando Caseiro^1^, António Gabriel^1^, Lola Svensson^2^, Fernando Mendes^1^, Wafa A. Siba^4^, Cristina Pereira^5^, Jorge Tomaz^5^

###### ^1^Biomedical Science Department, College of Health Technology of Coimbra, Polytechnic Institute of Coimbra, São Martinho do Bispo, 3046-854 Coimbra, Portugal; ^2^Department of Clinical Chemistry and Transfusion Medicine, Sahlgrenska University Hospital, Göteborg, 413 45 Göteborg, Switzerland; ^3^Complementary Sciences Department, College of Health Technology of Coimbra, Polytechnic Institute of Coimbra, São Martinho do Bispo, 3046-854 Coimbra, Portugal; ^4^Medical Laboratory Sciences Department, Al-Quds University, Abu Dis, P.O Box 89 Abu Dis, Palestinian National Authority; ^5^Blood Bank Service, Coimbra Hospital and Universitary Center, 3000-075 Coimbra, Portugal

####### **Correspondence:** Fernando Mendes (fjmendes@estescoimbra.pt) – Biomedical Science Department, College of Health Technology of Coimbra, Polytechnic Institute of Coimbra, São Martinho do Bispo, 3046-854 Coimbra, Portugal

**Background**

In 1987 three unrelated English families were reported with an alleged subgroup called Apae. The Apae positive family members showed a divergent antibody and lectin reaction pattern of erythrocytes. The lectin Helix Pomatia had strong reaction, polyclonal antibodies (Ab) anti-A had weak response and monoclonal Ab anti-A had no reaction, suggesting a controversy. Swedish researchers found evidences leading to abolish the subgroup Apae and establishing instead the FORS blood group system (accepted by ISBT 2012 as blood group system 31). It’s important to know the prevalence of the Ab to make the right decisions in transfusion medicine. For that reason, are needed cells containing the Fs saccharide, such as sheep erythrocytes, to search the Ab. Objective: Study the anti-Forssman (anti-Fs) antibody frequency.

**Methods**

Plasma from a total of 800 blood donors was included in the study. Sheep erythrocytes were crossed with blood donor plasma using the tube technique. Plasma from Apae individual was used as negative control and anti-Fs (M1/22.25.8HL cell line supernatant) was used as positive control.

**Results**

In 800 blood donors, one was negative for the presence of anti-Fs Ab.

**Conclusions**

Sheep erythrocytes are suitable to search the human anti-Fs. In this blood donor population (n = 811) we found one individual lacking the anti-Fs. Those results contribute to the data of being a rare blood group. It would be interesting to study the cells of the individual lacking the anti-Fs to understand if it is a FORS+ individual and if can have impact in transfusion medicine.

**Keywords**

Blood Group, New Histoblood System, FORS System, Forssman Antigen, Anti-Forssman

#### O147 Assessment of the war-related perceived threat in Portuguese Colonial War Veterans

##### Teresa Carvalho^1,2^, José Pinto-Gouveia^1^, Marina Cunha^1,2^

###### ^1^Centro de Investigação do Núcleo de Estudos e Intervenção Cognitivo-Comportamental, Faculdade de Psicologia e Ciências da Educação, Universidade de Coimbra, 3001-802 Coimbra, Portugal; ^2^Instituto Superior Miguel Torga, Coimbra, 3000-132, Portugal

####### **Correspondence:** Teresa Carvalho (teresacarvalho.psi@gmail.com) – Centro de Investigação do Núcleo de Estudos e Intervenção Cognitivo-Comportamental, Faculdade de Psicologia e Ciências da Educação, Universidade de Coimbra, 3001-802 Coimbra, Portugal

**Background**

The perception of threat (the fear for one’s safety and well-being) during exposure to war proved to be a major risk factor for development of psychopathology developed by war Veterans. The Perceived Threat Scale (PTS) of the Deployment Risk and Resilience Inventory (DRRI) is one the most widely used self-report instrument to assess this construct. Objective: This study aimed to test the two-factorial latent structure (Threats Combat and Non-Threats Combat) of the PTS adapted to Portuguese Colonial War Veterans (modified version), previously found through Exploratory Factor Analysis (EFA). The internal consistency and discriminant validity of this scale were also analysed.

**Methods**

Three hundred and ten (310) males from the general population of Portuguese Colonial War Veterans completed the PTS-MV (PTS-Modified Version), PTSD Checklist-Military Version (PCL-M), Beck Depression Inventory (BDI) and the Anxiety and Stress Scales of DASS-21.

**Results**

Confirmatory Factor Analysis (CFA) showed that the re-specified first–order two factor model has a good fit to the data (GFI = .92; TLI = .93; CFI = .94; RMSEA = .06; PCFI = .79) and adequate factorial validity. Also an adequate internal consistency was obtained (Treats Combat: α = .82; Non-Treats Combat: α = .83) and a good discriminant capacity regarding PTSD, depression, anxiety and stress symptoms.

**Conclusions**

CFA corroborates the two-factorial structure of the Portuguese Version of PTS previously found by the authors. The scale is internally consistent and has a good discriminant capacity.

**Keywords**

Assessment of perceived threat, Perceived Threat Scale (PTS), DRRI, Confirmatory factor Analysis, Portuguese Colonial War Veterans

#### O148 Pulse transit time estimation for continuous blood pressure measurement: A comparative study

##### Diana Duarte^1^, Nuno V. Lopes^1,2^, Rui Fonseca-Pinto^1,3^

###### ^1^Escola Superior de Tecnologia e Gestão, Instituto Politécnico de Leiria, 2411-901 Leiria, Portugal; ^2^Centre for the Research and Technology of Agro-Environmental and Biological Sciences, University of Trás-os-Montes and Alto Douro, 5001-801 Vila Real, Portugal; ^3^Instituto de Telecomunicações, Instituto Politécnico de Leiria, 2411-901 Leiria, Portugal

####### **Correspondence:** Diana Duarte (rui.pinto@ipleiria.pt) – Escola Superior de Tecnologia e Gestão, Instituto Politécnico de Leiria, 2411-901 Leiria, Portugal

**Background**

Continuous blood pressure (BP) monitoring provides important information about the cardiovascular system condition. Invasive methods are accurate but denote increased risk. Non-invasive methods are safe but less reliable and don´t provide continuous information. An alternative approach for a continuous, non-invasive measurement of BP is based on changes in pulse transit time (PTT). PTT is defined as the time delay between the R-wave of the electrocardiogram (ECG) and the peak value of the photoplethysmogram (PPG) signal acquired in the patient finger on the same cardiac cycle.

The main goal of this work is to estimate the PTT using different methodologies found in the literature, such as derivative based approaches and Discrete Wavelet (DWT) and Hilbert-Huang (HHT) transforms, and compare the results with ground truth values obtained manually.

**Methods**

Several ECG and PPG signals were obtained from a public physiologic signals database and from real data using in vivo patients. A derivative based approach to find the R waves and the PPG peak values was implemented to estimate the PTT. Then, a previous signal processing using DWT and HHT was applied. Results provided from each methodology were compared with ground truth values.

**Results and conclusions**

Globally all methods had similar results when using the database signals. However, using real data, the derivative based approach performed poorly. Due to noise, non-stationary and non-linearity nature of physiologic signals, methodologies that incorporate DWT or HHT provide accurate results.

**Keywords**

Blood Pressure, Pulse Transit Time, Wavelet Transform, Hilbert-Huang Transform

#### O149 Blood pressure assessment during standard clinical manoeuvres: A non-invasive PPT based approach

##### Diana Duarte^1^, Nuno V. Lopes^1,2^, Rui Fonseca-Pinto^1,3^

###### ^1^Escola Superior de Tecnologia e Gestão, Instituto Politécnico de Leiria, 2411-901 Leiria, Portugal; ^2^Centre for the Research and Technology of Agro-Environmental and Biological Sciences, University of Trás-os-Montes and Alto Douro, 5001-801 Vila Real, Portugal; ^3^Instituto de Telecomunicações, Instituto Politécnico de Leiria, 2411-901 Leiria, Portugal

####### **Correspondence:** Rui Fonseca-Pinto (rui.pinto@ipleiria.pt) – Instituto de Telecomunicações, Instituto Politécnico de Leiria, 2411-901 Leiria, Portugal

**Background**

Continuous and reliable blood pressure (BP) monitoring during standard clinical manoeuvres provides important information about the cardiovascular system condition. Common invasive methods are accurate but denote increased risk. An alternative approach is based on changes in pulse transit time (PTT) defined as the time delay between the R-wave of the electrocardiogram (ECG) and the peak value of the photoplethysmogram (PPG) signal at the same cardiac cycle.

The main goal of this work is to estimate BP variations derived from PTT changes during typical clinical autonomic manoeuvers associated with BP variations (e.g. lower limb elevation, orthostatic changes, and Valsalva). Furthermore, the validation of these blood pressure variations was also addressed.

**Methods**

Using the commercial equipment BIOPAC MP35, a set of ECG and PPG signals were acquired from healthy individuals during the manoeuvers, and PTT is calculated. Afterwards, the variations of BP were computed using a mathematical relationship and a blood pressure variation curve was obtained.

To validate the accuracy of this method, BP curves derived from PTT were compared with well-known pressure variation changes associated with the autonomic manoeuvres, from which the pressure profile is well documented in physiological studies. BP variations behave accordingly to responses from feedback mechanisms to maintain homeostasis.

**Conclusions**

This work establishes a diverse approach to assess BP changes, which proved to be reliable and accurate. The use of this methodology is an alternative to invasive and uncomfortable methods.

**Keywords**

Blood Pressure, Pulse Transit Time, Clinical autonomic manoeuvres

#### O150 Development and initial validation of the Activities and Participation Profile related to Mobility (APPM)

##### Anabela C. Martins (anabelacmartins@estescoimbra.pt)

###### Escola Superior de Tecnologia da Saúde de Coimbra, São Martinho do Bispo, 3046-854 Coimbra, Portugal

**Background**

Social participation has been extensively used in research and policy reports in health and social care as an outcome, since the World Health Organization published the International Classification of Functioning, Disability and Health (ICF) in 2001. Objective: To describe the development and validation of a questionnaire to measure participation of adults (Activities and Participation Profile related to Mobility; APPM) in their everyday activities.

**Methods**

The instrument was constructed within the frames of the ICF; the items are supported by results from previous studies and focus groups. The instrument’s internal consistency was assessed using Cronbach’s alpha coefficient. The content validity of the questionnaire was evaluated by linking the 18 items to the ICF. For construct validity, it was conjectured that the need for assistive technologies (AT) help of the arms to stand up from a chair, being sedentary and presence of falls were a mirror for more severely impaired performance, leading to a major limitation of activities and restrictions on participation relating to mobility. Criterion validity was investigated by correlation with FES.

**Results**

One hundred and fifty community dwelling adults (mean age 68.71 +/- 9.09 years old) participated in the study. The APPM demonstrated good internal reliability (alpha 0.90), indicating good homogeneity. All predefined hypotheses involving expected differences between groups and significant correlations between FES and APPM were confirmed.

**Conclusions**

The APPM demonstrated good psychometric properties and can be used as a reliable and valid measure to assess community dwelling adults' social participation over the age of 55 years.

**Keywords**

Activities and Participation, community dwelling adults, outcome measures, validation study

#### O151 MEASYCare-2010 Standard–A geriatric evaluation system in primary health care: Reliability and validity of the latest version in Portugal

##### Piedade Brandão^1,2^, Laura Martins^1^, Margarida Cardoso^3,4^

###### ^1^Escola Superior de Saúde, Universidade de Aveiro, 3810-193 Aveiro, Portugal; ^2^Center for Health Technology and Services Research, Universidade do Porto, 4200-450 Porto, Portugal; ^3^Instituto de Ciências Biomédicas Abel Salazar, Universidade do Porto, Porto, 4050-313 Porto, Portugal; ^4^Interdisciplinary Centre of Marine and Environmental Research, Universidade do Porto, 4050-123 Porto, Portugal

####### **Correspondence:** Laura Martins (laura.martins@ua.pt) – Escola Superior de Saúde, Universidade de Aveiro, 3810-193 Aveiro, Portugal

**Background**

EASYCare is a multidimensional instrument for assessing self-perception of the health care needs of the elderly population in primary health care. The latest version of this tool is the EASYCare-2010 Standard. Objective: This study aimed to validate the EASYCare-2010 Standard in older Portuguese people.

**Methods**

The sample comprises 244 participants with 65 years old or over recruited at Primary Health Care Centres from the Portuguese National Health Service. The World Health Organization Quality of Life Assessment Instrument-Short and the Tinetti test were considered for construct validity.

**Results**

Results from this study revealed a two-factor model through Categorical Data Principal Component Analysis. The internal consistency of this instrument had highly acceptable levels. The results also showed that in the polytomous items of EASYCare-2010 Standard some of the categories were not considered at all or only by a small number of participants.

**Results**

The EASYCare-2010 Standard factors were named *“mobility and activities of daily life*” and “*general well-being and safety*” and were associated with measures of quality of life with a moderate correlation with balance and gait. The instrument could be simplified, replacing polytomous with dichotomous items.

**Conclusions**

This study showed that the EASYCare-2010 Standard in Portugal is a valid and reliable comprehensive geriatric assessment tool for older community-dwelling people. However, the reduction and simplification of items should be equated in a new revised version. More studies are needed in other countries using EASYCare-2010 Standard to certify the results obtained.

**Keywords**

Geriatric assessment, Primary health care, Questionnaires, Reliability and validity

#### O152 Interrater and intrarater reliability and agreement of the range of shoulder flexion in the standing upright position through photographic assessment

##### Nuno Morais^1^, Joana Cruz^2^

###### ^1^School of Health Sciences, Polytechnic Institute of Leiria, 2411-901 Leiria, Portugal; ^2^School of Health Sciences, University of Aveiro, 3810-193 Aveiro, Portugal

####### **Correspondence:** Nuno Morais (nuno.morais@ipleiria.pt) – School of Health Sciences, Polytechnic Institute of Leiria, 2411-901 Leiria, Portugal

**Background**

The assessment of range of shoulder flexion in clinical settings is usually performed in supine position. However, this assessment position may not accurately reflect the impairments in shoulder mobility observed in several real-life situations that involve sitting or standing upright positions. This study aimed to assess the interrater and intrarater reliability and agreement of range of shoulder flexion in the upright position through photographic assessment.

**Methods**

Ten asymptomatic volunteers (4 males, 70.50 ± 10.29 years) participated in the study. They were photographed in the sagittal plane while performing maximal shoulder flexion. Shoulder flexion angle was then determined with computer-assisted digitising software. Raters 1 and 2 independently performed the measurements once and twice (respectively) using the standard goniometry landmarks: lateral epicondyle of the humerus, lateral midline of the thorax, and the centroid of the virtual glenohumeral joint region. Interrater and intrarater reliability were calculated using Intraclass Correlation Coefficients (ICC2,1) and agreement parameters using the 95 % limits of agreement (LoA), standard error of the measurement (SEM) and minimal detectable change (MDC95).

**Results**

Excellent interrater (ICC2,1 = 0.98 95 % confidence intervals [95%CI] = 0.93–0.99, SEM = 1.54°, MDC95 = 4.28°) and intrarater (ICC2,1 = 0.99, 95%CI = 0.98–0.99, SEM = 0.85°, MDC95 = 2.36°) reliability and agreement results were found. The LoA followed the same trend (intrarater LoA: -2.80°–1.91°; interrater LoA: -2.04°–6.51°).

**Conclusions**

Assessment of range of shoulder flexion in the upright position using photographic analysis is highly reliable and provides acceptable measurement error to be used in clinical practice. Further research is necessary to validate this measurement against ’gold standard’ measures (e.g., 3D motion tracking device).

**Keywords**

angular displacement, measurement properties, mobility, upper limb

#### O153 Three-dimensional biofabrication techniques for tissue regeneration

##### Nuno Alves, Paula Faria, Artur Mateus, Pedro Morouço

###### Centre for Rapid and Sustainable Product Development, Polytechnic Institute of Leiria, 2430-028 – Marinha Grande, Portugal

####### **Correspondence:** Nuno Alves (nuno.alves@ipleiria.pt) – Centre for Rapid and Sustainable Product Development, Polytechnic Institute of Leiria, 2430-028 – Marinha Grande, Portugal

Human tissue loss or end-stage organ failure resulting from an injury or a disease is a major health care problem in the world as the transplantation of tissues or organs in these patients is severely limited by availability of compatible donors. In this context, three-dimensional biofabrication techniques have emerged as powerful tools to produce functional homogeneous/heterogeneous substitutes (scaffolds), which have a huge potential to fulfil biological and mechanical requirements in order to achieve the full tissue regeneration.

Accordingly, several 3D biofabrication techniques have been developed and tested to produce anatomically correct biological substitutes through the use of computer-controlled 3D devices to accurately deposit biomaterials, drugs/growth factors/cells into precise geometries.

Given the multitude of 3D scaffolds applications, ranging from in vitro studies using a combination of 3D biocompatible and biodegradable structures with drugs/growth factors/living cells to in vivo implantation in order to pursue the full tissue regeneration, 3D biofabrication techniques can have a significant impact on the future of health care and well-being.

Advantages, drawbacks, results and capabilities of these kind of 3D scaffolds, techniques, ex vivo and in vivo studies are detailed. New directions in research on biofabrication for full tissue regeneration are also pointed out.

**Keywords**

Biological substitutes, 3D Biofabrication techniques, Tissue regeneration, In vitro studies

#### O154 A new computer tool for biofabrication applied to tissue engineering

##### Nuno Alves, Nelson Ferreira, Artur Mateus, Paula Faria, Pedro Morouço

###### Centre for Rapid and Sustainable Product Development, Polytechnic Institute of Leiria, 2430-028 – Marinha Grande, Portugal

####### **Correspondence:** Nuno Alves (nuno.alves@ipleiria.pt) – Centre for Rapid and Sustainable Product Development, Polytechnic Institute of Leiria, 2430-028 – Marinha Grande, Portugal

Traditionally, additive manufacturing (AM) uses digital information from a computer-aided design (CAD) file that is later converted to a stereo-lithography (STL) file format in order to produce a physical part. The surfaces of a 3D computer model are approximated by a set of triangular facets and then sliced to generate a series of layers for fabrication. The STL file format has become the standard for the data input of AM systems.

Digital information contained in the STL standard format is related to the vertex co-ordinates, which define each triangle and its outer normal. Despite of some STL file improvements focused on solving overlapping surfaces, gaps, and intersections between facets, this is not suitable for multimaterial additive biomanufacturing. Despite others approaches have also been developed, mainly focused in biofabrication processes, they are not able to deal with multimaterial scaffolds.

To overcome the above mentioned drawbacks, a novel multidimensional computer approach for biofabrication is presented, allowing the production of biological multimaterial substitutes with tailored properties improving mechanical and biological performance in tissue engineering applications.

**Keywords**

Biofabrication, computational tools, multimaterial scaffolds, tissue engineering

#### O155 Development and psychometric qualities of a scale to measure the functional independence of adolescents with motor impairment

##### Isabel Malheiro^1^, Filomena Gaspar^1^, Luísa Barros^2^

###### ^1^Escola Superior de Enfermagem de Lisboa, 1700-063 Lisboa, Portugal; ^2^Faculdade de Psicologia, Universidade de Lisboa, 1649-013 Lisboa, Portugal

####### **Correspondence:** Isabel Malheiro (mmalheiro@esel.pt) – Escola Superior de Enfermagem de Lisboa, 1700-063 Lisboa, Portugal

**Background**

There is a shortage of a validated instruments to evaluate the effectiveness of rehabilitation interventions in adolescents with motor impairment. The purpose of this study was to develop an instrument to measure functional independence perceived by adolescents with motor impairment, and evaluate its psychometric qualities.

**Methods**

Based on the Functional Independence Measure scale (FIM) we developed a self-report multidimensional instrument, to assess functional independence in adolescents. Ratings are made on an oriented format with five points, 1 (I can’t), 2 (I can with much help), to 5 (I can without help). The final version of the instrument (16 items), were tested on a convenience sample of 101 adolescents with motor impairment. The pilot test served not only to test their understandability, but also to evaluate their psychometric qualities (sensitivity, reliability and validity).

**Results**

The final version of SSFIA 16 items (Motor Domain) psychometric qualities was assessed by Internal consistency (Cronbach’s α = 0.938, split-half using Spearman & Brown correction α = 0.869). Construct validity was evaluated using principal factor analysis method with varimax orthogonal rotations, and revealed a tridimensional structure and explained 74.6 % of the variance (elimination, self-care and transfers). We found good support for convergent (FIM scale rp = 0.83 and rp > 0.5 between items) and discriminant validity (average variance extracted analysis).

**Conclusions**

The good psychometric qualities showed a clear evidence that the newly developed instrument provides a sensitive, reliable and valid tool to assess the functionality (motor domain) in adolescents with motor impairment.

**Keywords**

Adolescents, Self-rating multidimensional Instrument, Functional Independence, Psychometric qualities, Validity

#### O156 Organizational Trust in Health services: Exploratory and Confirmatory factor analysis of the Organizational Trust Inventory- Short Form (OTI-SF)

##### Pedro Parreira^1^, Andreia Cardoso^2^, Lisete Mónico^3^, Carla Carvalho^3^, Albino Lopes^4^, Anabela Salgueiro-Oliveira^1^

###### ^1^Escola Superior de Enfermagem de Coimbra, 3046-851 Coimbra, Portugal; ^2^Birmingham Hospitals, Birmingham B15 2GW, UK; ^3^Faculdade de Psicologia e Ciências da Educação, Universidade de Coimbra, 3001-802 Coimbra, Portugal; ^4^Instituto Superior de Ciências Sociais e Políticas, 1300-663 Lisboa, Portugal

####### **Correspondence:** Pedro Parreira (parreira@esenfc.pt) – Escola Superior de Enfermagem de Coimbra, 3046-851 Coimbra, Portugal

**Background**

Organizational Trust is a core concept for the effectiveness of Health services organizations. The concept has a great importance for the dynamic of professionals when working in groups, influencing the quality of health care. Instruments with god validity and reliability are useful for Portuguese health care services. Objective: To analyse the psychometric proprieties of the Organizational Trust Inventory-Short Form (OTI-SF) in Portuguese health services.

**Methods**

Quantitative study with a sample of N = 687 (89.2 % nurses and 10.8 % physicians of 50 units of medical and surgery services from Portuguese hospitals, age > 35 years, 75.8 % female). Measure: Organizational Trust Inventory- Short Form (OTI-SF), 7-point Likert scale.

**Results**

According to eigenvalue >1, two factors were extracted with Exploratory factor analysis (EFA) for Organizational Trust (KMO = .891, X^2^(66) = 2424.11, p < .001) explaining 60.87 % of variance (EV): F1-Honesty (33.70 % EV; M = 4.62, SD = 0.90), F2-Avoid opportunism (27.17 % EV; M = 3.52, SD = 1.10). Reliability was good (Cronbach’s α = .88 and α = .85 for F1 and F2, respectively). The item-factor correlations showed values greater than 0.74, consistent with construct validity. This factorial structure was supported by Confirmatory factor analysis (CFA), with a good fit: CMIN/50 = 1.92, RMSEA = .047 (LO90 = .033, HI90 = .061), NFI = .955, CFI = .978. Both “Honesty” (F1) and “Avoid opportunism” (F2) were equal across gender, F(1, 397) = 0.39 and 0.52, respectively, p > .40, and also across nurses and physicians, F(1, 322) = 1.72 and 0.25, p > .25. “Honesty” and “Avoid opportunism” shared 28 % of the variance and were not influenced by years of work.

**Conclusions**

The Organizational Trust Inventory-Short Form (OTI-SF) in health services showed adequate psychometric properties. The instrument is useful to measure organizational trust in health services.

**Keywords**

Organizational trust, health services, organizational trust inventory, health professionals

#### O157 Thermal symmetry: An indicator of occupational task asymmetries in physiotherapy

##### Adérito Seixas^1^, Valter Soares^1^, Tiago Dias^1^, Ricardo Vardasca^2^, Joaquim Gabriel^2^, Sandra Rodrigues^1^

###### ^1^Universidade Fernando Pessoa, Porto, 4249-004 Porto, Portugal; ^2^Faculdade de Engenharia, Universidade do Porto, 4200-465 Porto, Portugal

####### **Correspondence:** Adérito Seixas (aderito@ufp.edu.pt) – Universidade Fernando Pessoa, Porto, 4249-004 Porto, Portugal

**Background**

The burden associated to work-related musculoskeletal disorders is increasing and the need for research about technologies that allow a better understanding of this problem is well identified. The use of thermal imaging in occupational health research has been increasing but not to assess physiotherapy related tasks. Thermal symmetry reflects normal physiology and is defined as the degree of similarity between the skin temperature of two regions of interest mirrored across the human body longitudinal axis.

**Methods**

Fifteen (15) physiotherapy final year graduation students (age: 25.5 ± 5.4 years; BMI: 22.9 ± 2.8 Kg.m^-2^) underwent thermographic evaluation before, immediately after and 5 minutes after a passive hip hyper-extension mobilization task performed at a rate of 20 mobilizations per minute, during 4 minutes. The region of interest selected for analysis was located in the area of both upper trapezius muscles. Thermal images were obtained using a calibrated FLIR A325 camera with 320 x 240 resolution, 70 mK sensitivity and ± 2 % accuracy. Previously published recommendations were followed to capture thermal images.

**Results**

The passive mobilization task induced statistically significant changes in skin temperature. Thermal symmetry values increased from 0.1 ± 0.1 °C before the mobilization task to 0.4 ± 0.2 °C immediately after the task and 0.5 °C 5 minutes after. Statistically significant differences were found between thermal symmetry values before and 5 minutes after the task (p < 0.01).

**Conclusions**

The findings of this study provided interesting data regarding task asymmetry. Thermal symmetry values reflected the increase in skin temperature observed in the dominant side as a result of the mobilization task.

**Keywords**

Thermal imaging, thermal symmetry, passive mobilization, physiotherapy

#### O158 A study of ICT active monitoring adoption in stroke rehabilitation

##### Hugo Paredes^1,2^, Arsénio Reis^1^, Sara Marinho^1^, Jorge Lains^3^, Vítor Filipe^1,2^, João Barroso^1,2^

###### ^1^Universidade de Trás-os-Montes e Alto Douro, Vila Real, 5001-801 Vila Real, Portugal; ^2^Instituto de Engenharia de Sistemas e Computadores, Tecnologia e Ciência, 4200 - 465 Porto, Portugal; ^3^Centro de Medicina de Reabilitação da Região Centro - Rovisco Pais, 3064-908 Tocha, Portugal

####### **Correspondence:** Hugo Paredes (hparedes@utad.pt) – Universidade de Trás-os-Montes e Alto Douro, Vila Real, 5001-801 Vila Real, Portugal

**Background**

Every year 15 million people suffer an episode of stroke, worldwide. During the recovery therapy, the evaluation of patients' progress is carried out by doctors and specialized therapists. The application of technology in health care has attracted the attention of engineering for the support of recovery therapy practices. Nowadays, advances in information and communication technologies allow the creation of systems to support and evaluate the physical recovery of people that help health professionals evaluating the recovery of their patients. Objectives: The main objective of this work is to gather the requirements to implement a system focused on monitoring the upper limb rehabilitation of post stroke patients, establishing a link between main hospitals and patients when they are recovering at home or in proximity centres.

**Methods**

We used ethnographic methodology to examine the daily routines of patients in the recovery process. The information gathered was combined with interviews with therapists and medical doctors about the monitoring of the recovery process and therapy practices.

**Results**

The characterization of patients’ daily routines in the rehabilitation process, the exercises performed and the major physiological data required for monitoring rehabilitation recovery instruments was carried out. A combined perspective and analysis of the rehabilitation process is in an engineering-ready format for prototype implementation and pilot deployment.

**Conclusions**

The results of this study allow the specification of an innovative digital platform for stroke rehabilitation and patient monitoring, using pervasive technologies embedded with patients’ daily life without requiring explicit interaction of the patients or changes in daily routines.

**Keywords**

Stroke rehabilitation, ICT, active monitoring, technology in health care

#### O159 Paranoia Checklist (Portuguese Version): Preliminary studies in a mixed sample of patients and healthy controls

##### Carolina Da Motta^1,2^, Célia B. Carvalho^1,2^, José Pinto-Gouveia^1^, Ermelindo Peixoto^2^

###### ^1^Cognitive-Behavioural Research Centre, Faculty of Psychology and Education Sciences, University of Coimbra, 3001-802 Coimbra, Portugal; ^2^Azores University, São Miguel, 9501-855 Ponta Delgada, Portugal

####### **Correspondence:** Carolina Da Motta (carolina.d.motta@uac.pt) – Azores University, São Miguel, 9501-855 Ponta Delgada, Portugal

**Background**

Paranoid ideation is consistently found to be a thought process that is present across the population continuum, and a theme that most people find in their everyday thoughts. However, it is important to be able to distinguish subclinical or less severe forms of this phenomena from more clinical manifestations of paranoid beliefs. Objective: To provide preliminary data on the psychometric properties of the Portuguese version of the Paranoia Checklist in a mixed sample (47 patients and 157 healthy controls).

**Methods**

Self-report questionnaires were completed by 202 participants, with the aid of a psychologist when necessary.

**Results**

The PC has shown excellent internal consistency (frequency, conviction and distress subscales α > .96) and is a brief and simple measure capable of distinguishing between a clinical and non-clinical group of participants regarding the dimensions of frequency and conviction of paranoid thoughts. Participants from the clinical and non-clinical groups did not present statistically significant differences regarding the distress resulting from the paranoid thoughts.

**Conclusions**

Overall, the clinical population presented increased scores in all dimensions of paranoia in comparison to the healthy controls, similarly to the original studies with the English version of the checklist. The assessment of paranoia has been shown to be a continuum process common to clinical and non-clinical groups. The PC is a psychometrically sound measure to assess different paranoid thoughts on a multidimensional perspective and with sensitivity to distinguish groups of patients and healthy individuals, being suitable for use both in clinical and research settings.

**Keywords**

Paranoia, schizophrenia, assessment

#### O160 Reliability and validity of the Composite Scale on Morningness: European Portuguese version, in adolescents and young adults

##### Ana A. Gomes^1,2^, Vanessa Costa^3,4^, Diana Couto^1,4^, Daniel R. Marques^1,5^, José A. Leitão^3,6^, José Tavares^1^, Maria H. Azevedo^5^, Carlos F. Silva^1,2^

###### ^1^Department of Education and Psychology, University of Aveiro, Aveiro, 3810-193 Aveiro, Portugal; ^2^Center for Health Technology and Services Research, Faculty of Medicine, University of Porto, Porto, 4200-450 Porto, Portugal; ^3^Faculty of Psychology and Education Sciences, University of Coimbra, Coimbra, 3000-115 Coimbra, Portugal; ^4^Centro de investigação em educação e ciências do comportamento, Universidade de Aveiro, Aveiro, 3810-193 Aveiro, Portugal; ^5^Instituto de Imagem Biomédica e Ciências da Vida, Faculdade de Medicina, Universidade de Coimbra, Coimbra, 3000-548 Coimbra, Portugal; ^6^Centro de Investigação do Núcleo de Estudos e Intervenção Cognitivo Comportamental, Universidade de Coimbra, Coimbra, 3001 - 802 Coimbra, Portugal

####### **Correspondence:** Ana A. Gomes (ana.allen@ua.pt) – Center for Health Technology and Services Research, Faculty of Medicine, University of Porto, Porto, 4200-450 Porto, Portugal

**Background**

Morningness-eveningness, also known as chronotype, reflects demonstrable inter-individual differences in the peak timings (but not in the amount) of several circadian rhythms. The Composite Scale of Morningness (CSM) by Smith et al. (1989) [1] is one of the most widely used tools to access it. It has been long used in research in Portugal, but very few detailed reports exist about its psychometric properties in younger ages and student samples in our country. Objective: to report reliability and validity data about the Portuguese version (Pt) of the CSM in high school and university students.

**Methods**

Three hundred eighty-seven (387) high school students (7th to 12th grades, 51.5 %F) and 1654 undergraduates (1st to 3rd grades, 55.0%F) completed the CMS-Pt version [2], plus a set of self-report questions on sleep patterns in order to examine the questionnaire validity.

**Results**

As to internal consistency, Cronbach alpha coefficients were 0.81 in each sample (high school and undergraduate students). Corrected item-total correlations ranged from .27 to .55 (high schoolers) and from .31 to .59 (undergraduates). As to validity, lower morningness scores were associated, as expected, with later sleep-wake schedules and mid-points of sleep. Correlations between morningness-eveningness scores and sleep patterns were generally larger for sleep schedules variables than for time in bed or sleep durations, suggesting convergent and discriminant validity, respectively. Scale structure agreed with previous literature reports.

**Conclusions**

The CSM-Pt version is a reliable and valid tool to measure morningness-eveningness in adolescents and young adults’ students with ages ranging from 12 to 25 years old.

**References**

1. Smith CS, Reilly C, Midkiff K. Evaluation of three circadian rhythm questionnaires with suggestions for an improved measure of morningness. J Appl Psychol. 1989; 74: 728-738.

2. Silva CF, Azevedo MHP, Dias MRVC. Estudo padronizado do trabalho por turnos - versão portuguesa do SSI. [Standard Shiftwork Index - Portuguese version of the SSI]. Psychologica. 1995; 13: 27-36.

**Keywords**

Morningness-eveningness, chronotype, composite scale, CSM, adolescents, young adults, interindividual differences

#### O161 Evaluation scale of patient satisfaction with nursing care: Psychometric properties evaluation

##### João Freitas^1^, Pedro Parreira^2^, João Marôco^3^

###### ^1^Instituto de Ciências da Saúde, Universidade Católica Portuguesa, Lisboa, 1649-023 Lisboa, Portugal; ^2^Escola Superior de Enfermagem de Coimbra, 3046-851 Coimbra, Portugal; ^3^Instituto Universitário de Ciências Psicológicas, Sociais e da Vida, 1149-041 Lisboa, Portugal

####### **Correspondence:** João Freitas (mjbsfreitas@gmail.com) – Instituto de Ciências da Saúde, Universidade Católica Portuguesa, Lisboa, 1649-023 Lisboa, Portugal

**Background**

Patient satisfaction with nursing care appears as an important indicator for the evaluation of Structure factors – allocation of nurses, and Process factors – providing nursing care. The research aimed to evaluate the psychometric properties of the Patient Satisfaction with Nursing Care Scale (SUCEH21) in a Hospital, and also the reconstruction and validation of the scale’s new version.

**Methods**

We carried out a quantitative study, cross-sectional, sample of 1,290 patients admitted in 43 services, from 8 Portuguese hospitals. Confirmatory factor analysis (CFA) did not allow the confirmation of factorial structure.

The original model was composed by 6 factors and 21 items and revealed an unacceptable quality of adjustment (χ2(176) = 5050.132; p = 0.000; χ2/gl = 28.694; GFI = 0.765; PGFI = 0.583; RMSEA = 0.147). The model is valid only with 3 factors, ending up with a total of 13 items (χ2/gl = 6.017; p = 0.000; GFI = 0.958; PGFI = 0.600; RMSEA = 0.062).

**Results**

The exploratory factor analysis (EFA) studies confirm the number of factors found (Quality of Care, Quality of Information, Quality of Nursing Care) and 18 items. We found that the 3 factors are individually more extensive, covering the aspects that included the 6 initial factors. The final version of the Evaluation of Patient Satisfaction with Nursing Care Scale (EASCCE18) has an index of reliability (α = 0.875) and validity (total variance explained of 71.5 %) higher than those presented by SUCEH21.

**Conclusions**

Psychometric studies demonstrate this is a potential tool for the research and monitoring of patient satisfaction with nursing care.

**Keywords**

Nursing, nursing care, evaluation patient satisfaction

#### O162 Impact of fibromyalgia on quality of life: Comparing results from generic instruments and FIQR

##### Miguel A. Garcia-Gordillo^1^, Daniel Collado-Mateo^1^, Gang Chen^2^, Angelo Iezzi^3^, José A. Sala^1^, José A. Parraça^4^, Narcis Gusi^1^

###### ^1^University of Extremadura, Badajoz, 06071 Badajoz, España; ^2^Flinders Health Economics Group, Flinders University, Adelaide 5001, Australia; ^3^Centre for Health Economics, Monash University, Victoria, 3800, Australia; ^4^Universidade de Évora, 7004-516 Évora, Portugal

####### **Correspondence:** Miguel A. Garcia-Gordillo (miguelgarciagordillo@gmail.com) – University of Extremadura, Badajoz, 06071 Badajoz, España

**Background**

Cost-utility analysis (CUA) cannot be developed when the condition-specific measures do not allow calculation of quality-adjusted life-years (QALYs), as they do not provide utility scores. The revised version of the fibromyalgia impact questionnaire (FIQR) is the most common fibromyalgia specific questionnaire. The main aim of the current study was to develop a mapping algorithm which enables FIQR scores to be transformed into utility scores that can be used in economic analyses.

**Methods**

Data was obtained from 192 Spanish women with fibromyalgia aged between 23 and 83 years old. Several multi-attribute utility (MAU) instruments (EQ-5D-5 L, AQoL-8D, 15D and SF-6D) were selected to develop a mapping algorithm from FIQR. The “transfer to utility regression approach” was the technique selected for mapping analysis. Two models were calculated: I) using the total score of FIQR as independent variable and II) using the score in the three domains of FIQR. Age was included in both models.

**Results**

There were relevant differences between utility scores of the four analysed MAU instruments. All FIQR sub-scales were significantly correlated with the utility score of all MAU questionnaires (p < 0.0001). The predicted mean utilities using ordinary least squares (OLS) or generalized linear model (GLM) were always identical (up to three decimals) to the observed means. Both OLS and GLM showed good performance, with relatively low error values. The best model for estimation of FIQR utilities from MAU instruments differs depending on the MAU questionnaire.

**Conclusions**

The current study enables CUA using data from the most used fibromyalgia specific disease questionnaire.

**Keywords**

HRQoL, Multi attribute utility, Health, Mapping algorithm

#### O163 Preliminary study of the adaptation and validation of the Rating Scale of Resilient Self: Resilience, self-harm and suicidal ideation in adolescents

##### Jani Sousa^1^, Mariana Marques^1,2^, Jacinto Jardim^3^, Anabela Pereira^4^, Sónia Simões^1^, Marina Cunha^1^

###### ^1^Instituto Superior Miguel Torga, Coimbra, 3000-132 Coimbra, Portugal; ^2^Centro Hospitalar e Universitário de Coimbra, 3000-075 Coimbra, Portugal; ^3^Centro de Literaturas e Culturas Lusófonas e Europeias, Universidade de Lisboa, 1600-214 Lisboa, Portugal; ^4^Universidade de Aveiro, 3810-193 Aveiro, Portugal

####### **Correspondence:** Jani Sousa (janims@live.com.pt) – Instituto Superior Miguel Torga, Coimbra, 3000-132 Coimbra, Portugal

**Background**

In Portugal, there are few validated instruments to assess resilience in the adolescent population. Our main objective was a preliminary adaptation and validation of the Rating Scale of Resilient Self (RSRS) to Portuguese adolescents. Additionally, in the same sample, we explored the associations between resilience, self-harm and suicidal ideation in adolescence.

**Methods**

226 adolescents (male, n = 139, 61.5 %; age range = 12-18 years) filled in a protocol consisting of a sociodemographic questionnaire, the Rating Scale of Resilient Self (RSRS), the Impulse, Self-harm and Suicide Ideation Questionnaire for Adolescents and the Self-concept Scale.

**Results**

The RSRS showed good internal consistency (α = 0.857) and good temporal stability (r = 0.720). A principal component analysis showed that RSRS has three factors: external support; internal personal strengths; coping strategies. Resilience was negatively correlated with self-harm and suicidal ideation and positively correlated with self-concept, confirming the divergent and convergent validity of RSRS. There were high levels of resilience in this sample (M = 58.69, SD = 6.67). In the total sample, 61.5 % (n = 139) presented suicidal ideation and 26.5 % (n = 60) self-harm behaviours.

**Conclusions**

The RSRS presented good psychometric properties. It can be considered a valid and useful instrument, and can be safely used in the evaluation of resilience in Portuguese adolescents. With this study we extended the range of valid instruments for the measurement of resilience in adolescents and contributed to the advance of research in the area of adolescence in Portugal.

**Keywords**

Resilience, RSRS, self-harm, suicidal ideation, adolescence, assessment

#### O164 Development of the first pressure ulcer in inpatient setting: Focus on length of stay

##### Pedro Sardo^1^, Jenifer Guedes^2^, João Lindo^1^, Paulo Machado^3^, Elsa Melo^1^

###### ^1^School of Health Sciences, University of Aveiro, Aveiro, 3810-193 Aveiro, Portugal; ^2^Centro Hospitalar do Baixo Vouga, Aveiro, 3814-501 Aveiro, Portugal; ^3^Escola Superior de Enfermagem do Porto, Porto, 4200-072 Porto, Portugal

####### **Correspondence:** Pedro Sardo (pedro.sardo@ua.pt) – School of Health Sciences, University of Aveiro, Aveiro, 3810-193 Aveiro, Portugal

**Background**

Pressure ulcer incidence represents an indicator of healthcare quality. Several instruments are used in clinical practice to assess and identify patients at risk of developing pressure ulcers, however the preventive interventions were not always fully implemented and the incidence of pressure ulcer in inpatient setting is still high. Some studies reported a correlation between pressure ulcer development and the length of stay. Objectives: To identify the day of the first pressure ulcer development in inpatient setting.

**Methods**

Retrospective cohort analysis of electronic health record database from adult patients, admitted to medical and surgical areas in a Portuguese hospital during 2012, without any pressure ulcer at the admission. The development of the first pressure ulcer was associated with the length of inpatient stay.

**Results**

From a sample of 6,572 participants, 153 (2.3 %) developed their first pressure ulcer during the length of inpatient stay. The median length of stay was 6 days (Q25 = 3 days; Q75 = 10 days), being the maximum 134 days. During the first week, 80 participants (52.3 %) developed their first pressure ulcer. The highest frequency of participants that developed their first pressure ulcer occurred on the 5th day (minimum 2nd day; maximum 82nd day) and the median was on the 7th day.

**Conclusions**

Our study showed that the first week was particularly critical for pressure ulcer development and should be a period of highest nursing surveillance and preventive interventions. However, more studies are needed to better understand the correlation between time and pressure ulcer development.

**Keywords**

Length of stay, nursing assessment, pressure Ulcer, risk assessment

#### O165 Forms of Self-Criticizing and Self-Reassuring Scale: Adaptation and early findings in a sample of Portuguese children

##### Célia B. Carvalho^1,2^, Joana Benevides^1^, Marina Sousa^1^, Joana Cabral^1^, Carolina Da Motta^1,2^

###### ^1^Cognitive-Behavioural Research Centre, Faculty of Psychology and Education Sciences, University of Coimbra, 3001-802 Coimbra, Portugal; ^2^Azores University, São Miguel, 9501-855 Ponta Delgada, Portugal

####### **Correspondence:** Célia B. Carvalho (celia.mo.carvalho@uac.pt) – Cognitive-Behavioural Research Centre, Faculty of Psychology and Education Sciences, University of Coimbra, 3001-802 Coimbra, Portugal

**Background**

Self-criticism is characterized by self-blame and negative self-judgements, often associated with the onset of psychopathology and interpersonal difficulties. Two forms of self-criticism were conceptualized: the inadequate self (feelings of inadequacy or deficiency), and the hated self (more intense feelings of self-disgust and aggressiveness directed towards the self). On the other hand, the reassured self was conceptualized as the acceptance of past failure and mistakes, which is believed to be a protective factor against psychological problems. Objective: This study is aimed at adapting and presenting preliminary findings on the Forms of Self-Criticizing and Self-Reassuring Scale for Portuguese children (FSCSR-C).

**Methods**

A sample of 127 children participated in this study and were administered a research protocol including the FSCSR-C. Results: after deleting items that presented reliability and saturation problems, exploratory factor analysis with orthogonal rotation yielded a 3-factor solution that explained 49.33 % of the total FSCRS-C scores. The measure revealed a good internal consistency of the 3 factors found in the exploratory analysis: Inadequate self (α = 0.66), hated self (α = 0.73), reassured self (α = 0.69). Convergent and divergent validity were established with measures of shame and emotional intelligence.

**Conclusions**

Self-criticism can make individuals more vulnerable to psychopathology in adult life, and that such internal relationship models may arise early in childhood. Findings indicate that the FSCRS-C is and adequate measure to assess self-criticizing and self-reassuring in children with 8 years old or above. The FSCRS-C may constitute an important contribution for future research and in the development of preventive and intervention strategies for children.

**Keywords**

Self-criticism, psychometric properties, children

#### O166 Predictive ability of the Perinatal Depression Screening and Prevention Tool – Preliminary results of the dimensional approach

##### Ana T. Pereira^1^, Sandra Xavier^1^, Julieta Azevedo^1^, Elisabete Bento^1^, Cristiana Marques^1^, Rosa Carvalho^2^, Mariana Marques^3^, António Macedo^1^

###### ^1^Department of Psychological Medicine, Faculty of Medicine, University of Coimbra, 3000-354 Coimbra, Portugal; ^2^Unidade de Saúde Familiar – Topázio, Eiras, 3020-171 Coimbra, Portugal; ^3^Instituto Superior Miguel Torga, Coimbra, 3000 Coimbra, Portugal

####### **Correspondence:** Ana T. Pereira (bemestarperinatal.fmuc@gmail.com) – Department of Psychological Medicine, Faculty of Medicine, University of Coimbra, 3000-354 Coimbra, Portugal

**Background**

Experts recommend that the perinatal depression screening should combine the evaluation of depressive symptoms and of psychosocial risk factors. Objective: To analyse the predictive ability of the Perinatal Depression Screening and Prevention Tool (PDSP Tool) assessing both PD symptoms and risk factors previously identified by our team (lifetime history of depression/LtHD, prenatal insomnia, increased depressive symptoms and negative affect/NA at pregnancy) to identify clinical relevant postpartum depression.

**Methods**

Ninety-two (92) pregnant women (Mean age: 32.64 ± 4.59 years) in their second trimester of pregnancy (21.38 ± 2.41 weeks of gestation) completed the PDSP Tool; at six (6.34 ± 1.66) weeks postpartum they completed the Postpartum Depression Screening Scale (PDSS-21), in order to determine if they scored above or below the PDSS-21 cut off point for clinical depression (>40).

**Results**

Eighteen women (19.6 %) presented PDSS-21 scores > 40. The global correct classification rate of the PDSP Tool was 65.2 %. The false-negative rate was 4.3 %, the false-positive rate was 35.9 %, the true-negative rate was 44.6 % and the true-positive rate was 15.2 %. Considering the PDSP Tool components, 61.0 % of the women scoring PDSS-24 > 43 at pregnancy (X2 = 14.24, OR = 7.37); 38.9 % of women with LtHD (X2 = 14.24, OR = 7.37) and 66.7 % of women with high NA at pregnancy (X2 = 17.64, OR = 9.38) (all p < .001) presented PDSS-21 > 40 in the postpartum.

**Conclusions**

Clinically it is a very difficult task to identify the pregnant women who will have significant depressive symptoms in the postpartum. Our preliminary results are encouraging, by showing that using the PDS Tool can help we identify approximately two-thirds of these women.

**Keywords**

Perinatal depression, health assessment, screening

#### O167 Psychometric properties of the BaSIQS-Basic Scale on insomnia symptoms and quality of sleep, in adults and in the elderly

##### Ana M. Silva^1^, Juliana Alves^1^, Ana A. Gomes^1,2^, Daniel R. Marques^1,3^, Mª Helena Azevedo^3^, Carlos Silva^1,2^

###### ^1^University of Aveiro, Aveiro, 3810-193 Aveiro, Portugal; ^2^Center for Health Technology and Services Research, Faculty of Medicine, University of Porto, Porto, 4200-450 Porto, Portugal; ^3^Instituto de Imagem Biomédica e Ciências da Vida, Faculdade de Medicina, Universidade de Coimbra, 3000-548 Coimbra, Portugal

####### **Correspondence:** Ana A. Gomes (ana.allen@ua.pt) – Center for Health Technology and Services Research, Faculty of Medicine, University of Porto, Porto, 4200-450 Porto, Portugal

**Background**

Poor sleep and insomnia symptoms (e.g., difficulties in initiating or maintaining sleep, early morning awakenings, light sleep) are amongst the most common sleep complaints in adult’s populations, and especially in older ages. The Basic Scale on Insomnia complaints and Quality of Sleep (BaSIQS) [1] was developed as a brief tool to access sleep quality and insomnia complaints, in alternative either to longer questionnaires (such as the PSQI, the Pittsburgh Sleep Quality Index) or to single item assessments, and has already shown appropriate psychometric properties in higher-education samples. Objective: To assess the reliability and validity of the BaSIQS in non-student adults with varying age ranges and in the elderly.

**Methods**

Sample 1 comprised exclusively 60 elderly people (65 % women) from 66 to 89 years old, 30 institutionalized and 30 non-institutionalized. Sample 2 comprised 227 participants (52.2 % men) from 20 to 79 years old. Participants completed the BaSIQS and two questionnaires relevant for insomnia in order to examine scale validity (GSES-Glasgow Sleep Effort Scale and GCTI-Glasgow Contents of Thought Inventory).

**Results**

As to internal consistency, Cronbach alpha coefficients were 0.73 (sample 1) and 0.84 (sample 2). Corrected item-total correlations ranged from .28 to .61 (sample 1) and from .52 to .69 (sample 2). Moderate to large associations were found between the BaSIQS, GSES and GCTI, supporting construct validity. As expectable, women and older individuals had higher sleep difficulties in all scales.

**Conclusions**

BaSIQS shows suitable reliability and validity in young, middle-aged and elderly adults, so that it may constitute a practical tool to briefly assess perceived sleep quality and insomnia complaints.

**References**

1. Allen Gomes A, Ruivo Marques D, Meia-Via AM, Meia-Via M, Tavares J, Fernandes da Silva C, Pinto de Azevedo MH. Basic Scale on Insomnia complaints and Quality of Sleep (BaSIQS): reliability, initial validity and normative scores in higher education students. Chronobiol Int. 2015; 32(3):428-440.

**Keywords**

Sleep quality, insomnia symptoms, questionnaire, adult, elderly, BaSIQS, scale reliability and validity

#### O168 Enlightening the human decision in health: The skin melanocytic classification challenge

##### Ana Mendes^1^, Huei D. Lee^2,3^, Newton Spolaôr^2^, Jefferson T. Oliva^2^, Wu F. Chung^2,3^, Rui Fonseca-Pinto^1,4^

###### ^1^Escola Superior de Tecnologia e Gestão, Instituto Politécnico de Leiria, 2411-901 Leiria, Portugal; ^2^Laboratory of Bioinformatics, Graduate Program in Engineering and Computing, West Paraná State University, Cascavel, Paraná, 85819-110, Brasil; ^3^Universidade Estadual de Campinas, Campinas – São Paulo, 13083-970, Brasil; ^4^Instituto de Telecomunicações, Instituto Politécnico de Leiria, 2411-901 Leiria, Portugal

####### **Correspondence:** Ana Mendes (aimendes@ipleiria.pt) – Escola Superior de Tecnologia e Gestão, Instituto Politécnico de Leiria, 2411-901 Leiria, Portugal

**Background**

Skin cancer is becoming increasingly relevant due to its high rates of incidence and mortality, being early detection of skin markers the key for successful treatment. Digital image processing (DIP) techniques can quantitatively describe dermoscopic images, reducing the effects of inter and intra individual differences in classification, resulting from the subjectivity of human eye assessments. Moreover, quantitative markers enable the use of automatic classifiers, which should be regarded as an additional tool in diagnosis. Objectives: This work employs image descriptors and classification algorithms, aimed to describe and differentiate cancerous from non-cancerous skin images.

**Methods**

Texture and shape characteristics from 104 images were extracted, representing 166 features. Subsequently, four groups were defined: Texture, Shape and Local binary patterns (TSL); Texture and Shape (TS); Texture and Local binary patterns (TL); and Texture (T). Next, the ReliefF feature selection algorithm was employed to rank features. For each group, the 10 %, 20 %, 40 % and 80 % of the best features were selected. All subsets were used to construct models using four machine learning (ML) methods implemented in the Weka framework: J48 Decision Tree, Support Vector Machines, Nearest Neighbour and Random Forest.

**Results and conclusions**

Among the ML used algorithms, J48 classifiers were considered promising after reaching the higher score in a sensitivity derived metric (14.01 in 16.00) when compared with the others.

Once the obtained ML classification reveals to be competitive, when comparing with other related works, the results strengthen the idea that DIP can be useful to provide a second opinion regarding skin cancer diagnosis.

**Keywords**

Skin Cancer, Dermoscopy, Digital Image Processing, Machine Learning Algorithms

#### O169 Test-retest reliability household life study and health questionnaire Pomerode (SHIP-BRAZIL)

##### Keila Bairros, Cláudia D. Silva, Clóvis A. Souza, Silvana S. Schroeder

###### Fundação Universidade Regional de Blumenau, Blumenau - Santa Catarina, 89012-900, Brasil

####### **Correspondence:** Keila Bairros (ship.keila@gmail.com) – Fundação Universidade Regional de Blumenau, Blumenau - Santa Catarina, 89012-900, Brasil

**Background**

Population health research primarily aims to find answers to various health issues and the integrity of the results is largely determined by the quality of the information produced. Objectives: To assess the test-retest reliability of the data collected during home interviews conducted by field interviewers in the Study of Health In Pomerode (SHIP-BRAZIL) in partnership with the University Ernst-Moritz-Arndt University of Greifswald in Germany.

**Methods**

Eight questions in the household questionnaire SHIP - BRAZIL study were administered twice with an interval of 15 to 20 days, from May to August 2015, with 85 participants. Test-retest reliability of the responses was estimated by the kappa statistic.

**Results**

Kappa coefficients ranged from 0.59 to 0.90. The Kappa values for stratification by gender ranged from 0.71 to 0.92 in females and 0.64 to 0.94 for men. For ages they ranged between 0.54 and 0.90 in participants aged 40 years or more and 0.77 to 1.0 in participants of 39 years old or less. By level of education they ranged from 0.48 to 0.88 in participants with primary education and 0.82 to 0.92 in participants with high school and college. There was no significant variation in agreement among sociodemographic subgroups.

**Conclusions**

The high Kappa values indicate good reliability and reproducibility of the data collected with the household questionnaire SHIP - BRAZIL study between May-August 2015 ranging from moderate to perfect agreement, ensuring the applicability of these data in future actions of promotion and prevention of health.

**Keywords**

Reliability, Reproducibility of results, Questionnaire, Epidemiology

#### O170 Characterization of sun exposure behaviours among medical students from Nova Medical School

##### Elsa Araújo^1^, Helena Monteiro^1^, Ricardo Costa^1^, Sara S. Dias^1,2,3^, Jorge Torgal^1^

###### ^1^Departamento de Saúde Pública, Faculdade de Ciências Médicas, Universidade Nova de Lisboa, 1169-056 Lisboa, Portugal; ^2^Centro de Estudos de Doenças Crónicas, Universidade Nova de Lisboa 1150-082 Lisboa, Portugal; ^3^Unidade de Investigação em Saúde, Escola Superior de Saúde de Leiria, 2411-901 Leiria, Portugal

####### **Correspondence:** Sara S. Dias (sara.dias@fcm.unl.pt) – Unidade de Investigação em Saúde, Escola Superior de Saúde de Leiria, 2411-901 Leiria, Portugal

**Background**

The exposure to sun ultraviolet radiation has important implications on public health. The intensive and intermittent solar exposure is associated with well reported complications and the younger population is a target due to the increase of malignant pathology of skin. The aim of this study was to characterize sun protection and sun exposure behaviour among medical students from Nova Medical School (Lisbon).

**Methods**

We performed an exploratory, descriptive and cross-sectional study. This study included 186 students from the Faculty of Medical Sciences of the Nova Medical School, Universidade Nova de Lisboa, between the ages of 17 and 39. The information was acquired by self-administered questionnaires that collected information about sociodemographic and behaviour variables including sun exposition and the used methods to protect from sun exposition.

**Results**

High prevalence of solar exposure has been verified among the students. The association between sociodemographic variables and the use of sunscreen has not been proven, except when it comes to “age”. There has been evidence of association between lower phototype and the anuse of Sun Protection Factor (SPF) 6-10. The practice more used was “to find a shadow” and the less used was “use of hat, cap or scarf on the head”.

**Conclusions**

This study revealed a high prevalence of solar exposure in the summer, sunburns and low adherence to protective measures, except for the use of sunscreen. However, the pattern of use is unsatisfactory when compared to the recommended guidelines, foreshadowing a potential risk of development of associated complications. Hereupon, the necessity of change in the behaviour of solar exposure and protection of students stands out.

**Keywords**

Sun exposure, sun protection, sunscreen, students

#### O171 Spirituality in pregnant women

##### Carolina G. Henriques^1,2^, Luísa Santos^2^, Elisa F. Caceiro^2^, Sónia A. Ramalho^1,2^

###### ^1^Health Research Unit, Polytechnic institute of Leiria, 2411-901 Leiria, Portugal; ^2^School of Health Sciences, Polytechnic institute of Leiria, 2411-901 Leiria, Portugal

####### **Correspondence:** Carolina G. Henriques (carolina.henriques@ipleiria.pt) – School of Health Sciences, Polytechnic institute of Leiria, 2411-901 Leiria, Portugal

**Background**

Spirituality is used by pregnant women as a coping strategy and includes: the need for respect; the individual’s quest for individual fulfilment; and the meaning of life and the experiences to which they are subject. Objectives: To identify the sociodemographic characteristics of pregnant women; determine the level of spirituality of pregnant women and to assess the relation between the level of spirituality and some sociodemographic, obstetric and clinical variables.

**Methods**

A quantitative, correlational and cross-sectional study, with a sample of 143 pregnant women in prenatal consultation in the central region of Portugal. A questionnaire was applied on sociodemographic, obstetric and clinical data and the "Scale for the assessment of spirituality in the health settings" Pinto and Pais-Ribeiro (2007) was used.

**Results**

Participants/pregnant women had an average age of 32.13 years (s = 4.93), are married/have partners (74.1 %), Portuguese (95.8 %), catholic (86.0 %), with secondary education (42.7 %) and live with the father of the unborn child (93.0 %). The sample included women in their first pregnancy (31.5 %), who desired the pregnancy (98.6 %), with a diagnosed disease (18.2 %) and previous abortion (16.1 %). Their spirituality level is 14.94 (s = 3.0), above the average range value (X_med_ = 12.5). It was verified that there are significant differences between the level of spirituality and: nationality (U = 203.0; p = 0.035) and education (X^2^ = 7.89; p = 0.019).

**Conclusions**

This study showed the need for spirituality as the focus of nursing care, the importance of this in the transition process, justifying the need to include assessment tools of spiritual well-being in clinical settings.

**References**

1. Pinto C, Pais-Ribeiro J. Construção de uma escala de avaliação da espiritualidade em contextos de saúde. ArquiMed. 2007; 21 (2): 47-53.

**Keywords**

Pregnancy, Spirituality, Nursing

#### O172 Polypharmacy in older patients with cancer

##### Rita Oliveira^1,2^, Vera Afreixo^2^, João Santos^1^, Priscilla Mota^1^, Agostinho Cruz^1^, Francisco Pimentel^2^

###### ^1^Escola Superior de Tecnologia da Saúde, Instituto Politécnico do Porto, 4400-330 Vila Nova de Gaia, Portugal; ^2^Universidade de Aveiro, 3810-193 Aveiro, Portugal

####### **Correspondence:** Rita Oliveira (rfo@estsp.ipp.pt) – Universidade de Aveiro, 3810-193 Aveiro, Portugal

**Background**

Polypharmacy is associated with drug–drug interactions, adverse drug events, hospitalization, increased mortality and rising costs. Older cancer patients are potentially vulnerable to polypharmacy because cancer treatment often involves exposure to chemotherapy and other adjunctive or supportive medication. Furthermore, the majority of elderly patients with cancer have pre-existing medical conditions requiring pharmacotherapy. This study allowed us to characterize the drugs administered to elderly patients undergoing chemotherapy treatments and to investigate the prevalence of polypharmacy.

**Method**

This was an observational and transversal study of older cancer patients admitted to three different medical centres of both genders aged > 65 years old undergoing chemotherapy treatments. The medication review included patient self-report and medical records. This study included 559 elderly patients. Mean age was 71.9 years (SD = 0.2). 44 % were women. Mean number of medications reported was 5.

The total number of medications used for the 559 patients was 2699, 58 % of them corresponding to drugs administered out of cancer treatment contexts.

**Results**

When analysed individually, the most prescribed drugs were Proton Pump Inhibitors (261/559; 47 %); HMG CoA reductase inhibitors (161/559; 29 %); Anxiolytics, Benzodiazepine derivatives (149; 27 %); Drugs for Functional Gastrointestinal Disorders, Propulsives (106/559; 19 %) and Analgesics and Antipyretics, Anilides (96/559; 17 %). The prevalence of polypharmacy (> = 5 medications) was 48 %.

**Conclusions**

Polypharmacy is prevalent in older people with cancer. A careful assessment of the medication used in older patients needs to be part of the routine evaluation to minimize risks associated with polypharmacy. However, more research is needed to identify strategies to simplify patients’ medication regimens.

**Keywords**

Elderly patients, older patients, polypharmacy, cancer, chemotherapy

#### O173 Quality of life of caregivers of people with advanced chronic disease: Translation and validation of the quality of life in life threatening illness - family carer version (QOLLTI-C-PT)

##### Rita Marques^1^, Mª Anjos Dixe^2^, Ana Querido^2^, Patrícia Sousa^3^

###### ^1^Hospital de Santa Maria, Centro Hospitalar Lisboa Norte, 1649-035 Lisboa, Portugal; ^2^Health Research Unit & School of Health Sciences, Polytechnic institute of Leiria, 2411-901 Leiria, Portugal; ^3^Universidade Católica Portuguesa, Lisboa, 1649-023 Lisboa, Portugal

####### **Correspondence:** Rita Marques (ritamdmarques@gmail.com) – Hospital de Santa Maria, Centro Hospitalar Lisboa Norte, 1649-035 Lisboa, Portugal

**Background**

Caregivers are often subject of huge physical, emotional, psychological, spiritual, social and financial demands, which can seriously compromise their quality of life (QOL). Objective: To translate, adapt and validate the Quality of life in life threatening illness-family carer version (QOLLTI-C) of Cohen et al (2006) [1], to the Portuguese culture, for caregivers of people with chronic and advanced disease.

**Methods**

We applied a questionnaire consisting of demographic data and the QOLLTI-C to 314 caregivers of patients with palliative needs. The sample is predominantly female (84.1 %; 264), with an average age of 63 years old (±11). Through the process of validation and cultural adaptation, we followed the methodological steps recommended by international guidelines. The study of the items and respective reliability was done according to two criteria: determining the Pearson correlation coefficient and the Cronbach's alpha (α). The construct validation was done by factorial analysis. Throughout the study, Helsinki Declaration Principles were followed.

**Results**

The QOLLTI-C-PT has an internal consistency with 13 items, α = 0.780, a 0.736 KMO, and a Bartlett's sphericity test of 1189.967 (p < 0.001). Using the Kaiser criterion, we obtained four factors that explain 61.89 % of the total variance: Factor 1 – social domain (items 1, 12, 13, 14, 15); Factor 2 – Spiritual domain (items 9, 10, 11); Factor 3 – Psychological domain (items 3, 4, 5) and Factor 4 – Physical and emotional domain (items 7, 8).

**Conclusions**

The instrument showed an adequate factorial validity and reliability, in the sample under study, and can be used to access QOL in Portuguese caregivers.

**References**

1. Cohen R, Leis A, Kuhl D, Charbonneau C, Ritvo P, Ashbury F. QOLLTI-F: Measuring Family Carer Quality of Life. Palliative Medicine. 2006; 20:755-767.

**Keywords**

Quality of life, caregivers, people with advanced chronic disease, validation, scale

#### O174 The psychometric properties of the brief Other as Shamer Scale for Children (OAS-C): preliminary validation studies in a sample of Portuguese children

##### Joana Benevides^1^, Carolina Da Motta^1,2^, Marina Sousa^1^, Suzana N. Caldeira^1,3^, Célia B. Carvalho^1,2^

###### ^1^Azores University, São Miguel, 9501-855 Ponta Delgada, Portugal; ^2^Cognitive-Behavioural Research Centre, Faculty of Psychology and Education Sciences, University of Coimbra, 3001-802 Coimbra, Portugal; ^3^Centro Interdisciplinar de Ciências Sociais, Universidade dos Açores, São Miguel, 9501-855 Ponta Delgada, Região Autónoma dos Açores, Portugal

####### **Correspondence:** Joana Benevides (joana.c.benevides@uac.pt) – Azores University, São Miguel, 9501-855 Ponta Delgada, Portugal

**Background**

Shame is a social emotion with specific and adaptive functions, involved in complex human behaviour and social interactions. The study of shame gained increased interest in several research fields, particularly in mental health, as an involuntary response associated with self-consciousness, loss of status and self-devaluation. Early shame experiences have been consistently reported as an important factor in the development of psychopathology, emphasizing the importance of evaluating shame and the negative representations of the self from an early age. Aims: This study’s goal is to adapt and present preliminary psychometric data on the Other as Shamer Scale to Portuguese children (8 years or over), a scale devised to assess external shame (how one believes one appears in the eyes of others).

**Methods**

A sample of 127 children participated in this study and a research protocol was administered including the OAS adapted for children (OAS-C).

**Results**

Exploratory Factor Analysis (EFA) was computed and the one-factor solution explained 48.85 % of the total variance of the scale. The measure also showed good internal consistency (α = .84). Convergent and divergent validity was found with measures of self-criticism and self-reassurance, and emotional intelligence.

**Conclusions**

The OAS-C is a brief and adequate measure of external shame in children, with the potential to be used by professionals in clinical and research settings. Moreover, the availability of a widely used measure of external shame will also facilitate transnational and cross-cultural studies by warranting score comparability across several countries and also across the lifespan.

**Keywords**

external shame, psychometric properties, children

#### O175 Measuring emotional intelligence in health care students – Revalidation of WLEIS-P

##### Ana Querido^1^, Catarina Tomás^1^, Daniel Carvalho^1,2^, João Gomes^1,2^, Marina Cordeiro^1^

###### ^1^School of Health Sciences, Polytechnic institute of Leiria, 2411-901 Leiria, Portugal; ^2^Hospital de Santo André, Centro Hospitalar de Leiria, 2410-197 Leiria, Portugal

####### **Correspondence:** Ana Querido (ana.querido@ipleiria.pt) – School of Health Sciences, Polytechnic institute of Leiria, 2411-901 Leiria, Portugal

**Background**

Emotional intelligence (EI) has been linked to effective communication in education environments, better study skills, stress and conflict management, and academic and workplace success. Measuring EI could provide information to access and monitoring emotional skills. Objective: To test psychometric properties of the Portuguese version of Wong and Low Emotional Intelligence Scale (WLEIS-P) in a sample of Health care students.

**Methods**

We applied a questionnaire consisting of demographic data and WLEIS-P to a sample of 672 health care students in Dietetics, Nursing, Physiotherapy, Speech Therapy and Occupational Therapy, mostly females (85.4 %), M = 21.6 years old (±4.17). The process of validation followed the methodological steps recommended by international guidelines. The study of the items and respective reliability was done by determination of Pearson correlation coefficient and the Cronbach's alpha(α). Construct validation was done by factorial analysis. Helsinki Declaration Principles were attended.

**Results**

The WLEIS-P applies to students has a good internal consistency with 16 items, α = 0.825, a 0.825 KMO, and a Bartlett's sphericity test of 3691.523 (p < 0.001). Using the Kaiser criterion, we obtained four factors that explain 61.52 % of the total variance: Factor 1- Self-emotional appraisal (items 1,2,3,4); Factor 2- Emotional appraisal of others (items 5,6, 7, 8); Factor 3 – Use of emotion (items 9,10,11,12) and Factor 4 – Regulation of emotion (items 13, 14, 15, 16).

**Conclusions**

WLEIS-P revealed an adequate factorial validity and reliability in the sample of health students, better than previous Portuguese validation, therefore it can be used to access Emotional Intelligence in Portuguese students.

**Keywords**

Emotional intelligence, students, validation, scale

#### O176 Health indicators in prenatal assistance: The impact of computerization and of under-production in basic health centres

##### Joyce O. Costa, Frederico C. Valim, Lígia C. Ribeiro

###### Faculdade de Medicina, Universidade José do Rosario Vellano, Divinópolis – Minas Gerais, 35502-634, Brasil

####### **Correspondence:** Joyce O. Costa (joyceoliveiramed@gmail.com) – Faculdade de Medicina, Universidade José do Rosario Vellano, Divinópolis – Minas Gerais, 35502-634, Brasil

**Background**

The innovative and transformative use of information technology improves professional practice, helps in the management and establishment of public policy and in knowledge creation. The platform SISREDE, created in 2002, stores the medical card of citizens of the public system, and was considered the most advanced platform in the country. Besides the monitoring and reporting of prevalent diseases, like hypertension, it even allows access to SISPRENATAL, part of the system that receives the data of pregnant women. Objectives: To compare the assistance indicators of prenatal from SISREDE/SISPRENATAL data and the whole volume of attendance registered on computers managed by professionals for the use of such resources in Etelvina Carneiro Health Centre, located in the North of Belo Horizonte.

**Methods**

Technicians from Belo Horizonte Council and researchers have created specific training for doctors and nurses involved in the unit’s prenatal assistance. After such preparation, the productivity of a quarter year was compared from national and state prenatal assistance.

**Results**

The minority of professionals received previous training to use the system and/or were unaware of what was needed within the protocol for prenatal assistance and generating trusted indicators. The numbers after the intervention demonstrated an exponential improvement of prenatal assistance indicators.

**Conclusions**

The making of health indicators contributes to public policy and resource management.

There are several limitations for such data effectively reflecting Health Care Centre assistance. The professional specialization for the system’s management and the intrinsic delays for such this relate to a lower index than the actual number of assistances.

**Keywords**

Computerization, health indicators, prenatal care

#### O177 Hope genogram: Assessment of resources and interaction patterns in the family of the child with cerebral palsy

##### Zaida Charepe^1^, Ana Querido^2^, Mª Henriqueta Figueiredo^3^

###### ^1^Universidade Católica Portuguesa, Lisboa, 1649-023 Lisboa, Portugal; ^2^Health Research Unit, School of Health Sciences, Polytechnic institute of Leiria, 2411-901 Leiria, Portugal; ^3^Escola Superior de Enfermagem do Porto, Porto, 4200-072 Porto, Portugal

####### **Correspondence:** Zaida Charepe (zaidacharepe@ics.lisboa.ucp.pt) – Universidade Católica Portuguesa, Lisboa, 1649-023 Lisboa, Portugal

**Background**

Hope has been defined as a dynamic life force, an important phenomenon in the promotion, maintenance and sustenance of life. At this level there are several strategies that promote hope that have been described in the literature, including the involvement of the extended family, especially in its relationship with the anguish of experiences in parents of children with special health care needs. Objective: To identify the resources and hope interactions in an inter-generational perspective on child family with cerebral palsy.

**Methods**

This qualitative study was conducted with a sample of parents of children with cerebral palsy in the area of influence of *Administração Regional de Saúde* (ARS) of Lisboa and Vale do Tejo. For data collection and treatment, semi-structured interviews and content analysis were used, respectively.

**Results**

From the parents' perspective the Hope genogram was co-constructed. They identified the family as a network of support and encouragement. Optimism, self-efficacy of mothers in caring for their children and the acceptance of their health condition were valued as positive psychological factors. From the analysis of indicators emerged personal attributes, relationships and bond established with other family members. Hope was valued in the coping process, playing a significant role in the adjustment of mothers to children's health.

**Conclusions**

Hope genogram is an instrument that facilitates the assessment of the internal and external structure of the family of children with cerebral palsy. It allowed the identification of stories of hope as well as personal attributes and patterns of hope promoters as interactions established among members.

**Keywords**

Cerebral Palsy, child, family relations, hope

#### O178 The influence of childbirth type in postpartum quality of life

##### Priscila S. Aquino^1^, Samila G. Ribeiro^2^, Ana B. Pinheiro^1^, Paula A. Lessa^3^, Mirna F. Oliveira^4^, Luísa S. Brito^2^, Ítalo N. Pinto^2^, Alessandra S. Furtado^2^, Régia B. Castro^1^, Caroline Q. Aquino^1^, Eveliny S. Martins^1^

###### ^1^Federal University of Ceará, Fortaleza - Ceará, 60020-181, Brazil; ^2^Federal University of Piauí, Teresina - Piauí, 64049-550, Brazil; ^3^Federal Institute of Education, Science and Technology of Ceará, Fortaleza – Ceará, 60040-531, Brazil; ^4^Federal University of Cariri, Juazeiro do Norte - Ceará, 63048-080, Brazil

####### **Correspondence:** Priscila S. Aquino (priscilapetenf@gmail.com) – Federal University of Ceará, Fortaleza - Ceará, 60020-181, Brazil

**Background**

The quality of life related to postpartum health is influenced by several factors. Purpose: The aim is to analyse the quality of life related to the health of women immediately postpartum.

**Methods**

It is a cross-sectional, correlational study, performed in a public hospital of reference in Piauí, Brazil. Two hundred and seventy-two (272) women in immediate postpartum were interviewed between February and June 2015. A questionnaire with clinical data and the scale for the evaluation quality of life - Short Form Health Survey (SF-36) were used.

**Results**

The sample was composed by young mothers, living with a partner (76.8 %); with an average of 10 years of study; 74.3 % didn't follow gainful occupation. In assessing the quality of life related to health from the SF-36, the average total score was 69.94. The association between type of delivery, attending professionals and the mean scores of the Short Form Health Survey (SF-36) showed that the domains of physical limitations (41.37), pain (67.53), vitality (62.75), social issues (74.78), limitations due to emotional problems (58.05) and mental health (75.86) had higher mean scores for vaginal delivery performed by a nurse, in which the statistically significant domains were pain, vitality and mental health. There was a significant association with age and the domains of functional capacity and general health, as well as education and the "functioning" domain.

**Conclusions**

The results indicated that vaginal delivery performed by the nurse promotes better scores in quality of life related to health of mothers.

**Keywords**

Postpartum Period, nursing, quality of life

#### O179 Women’s beliefs about pap smear test and cervical cancer: influence of social determinants

##### Ana B Pinheiro^1^, Priscila S. Aquino^1^, Lara L. Oliveira^1^, Patrícia C. Pinheiro^1^, Caroline R. Sousa^1^, Vívien A. Freitas^1^, Tatiane M. Silva^1^, Adman S. Lima^1^, Caroline Q. Aquino^1^, Karizia V. Andrade^1^, Camila A. Oliveira^1^, Eglidia F. Vidal^2^

###### ^1^Federal University of Ceará, Fortaleza - Ceará, 60020-181, Brazil; ^2^Federal University of Cariri, Juazeiro do Norte - Ceará, 63048-080, Brazil

####### **Correspondence:** Priscila S. Aquino (priscilapetenf@gmail.com) – Federal University of Ceará, Fortaleza - Ceará, 60020-181, Brazil

**Background**

Cervical cancer is considered a serious public health problem. Objective: To analyse the influence between social determinants and women’s beliefs regarding Pap smear and cervical cancer by using the scale Health Belief Model Scale for Cervical Cancer and Pap Smear Test.

**Methods**

Cross-sectional study conducted in a primary care unit in Fortaleza, Brazil, with 175 women. For data collection, it was used the socio-demographic and gynaecological characterization form based on the Model of Social Determinants of Health involving individual, proximal, intermediate determinants and influence of social networks, beyond the scale. Data was collected in May 2015.

**Results**

Women over 30 years old had better perception regarding cervical cancer seriousness (p = 0.024); women with menarche over 11 years old had high health motivation (p = 0.052); women with less education had many barriers to realize the preventive exam (p = 0.000) and better perception of cervical cancer seriousness (p = 0.000). Concerning the perception of cervical cancer seriousness, stood out women with a partner (p = 0.030), pregnant women (p = 0.010), those who have sex currently (p =0.029) and those who currently owned partner (p = 0.020). Those with income less than one salary had lot of barriers to realize the preventive exam (p = 0.000).

**Conclusions**

The social determinants of health influenced health beliefs for cervical cancer. Therefore, to intervene in such determinants may contribute to decrease the morbidity and mortality caused by this disease.

**Keywords**

Colonic Neoplasms, Health Promotion, Papanicolaou Test

#### O180 Validity of the Portuguese version of the ASI-3: Is anxiety sensitivity a unidimensional or multidimensional construct?

##### Ana Ganho-Ávila^1^, Mariana Moura-Ramos^2^, Óscar Gonçalves^1^, Jorge Almeida^2,3^

###### ^1^Centro de Investigação em Psicologia, Escola de Psicologia, Universidade do Minho, 4710-057 Braga, Portugal; ^2^Cognitive and Behavioural Centre for Research and Intervention, Faculty of Psychology and Educational Sciences, University of Coimbra, 3000-115 Coimbra, Portugal; ^3^PROACTION Laboratory, 3001-802 Coimbra, Portugal

####### **Correspondence:** Ana Ganho-Ávila (anaganho@gmail.com) – Centro de Investigação em Psicologia, Escola de Psicologia, Universidade do Minho, 4710-057 Braga, Portugal

**Background**

We aimed to test the Portuguese version of the Anxiety Sensitivity Scale 3 – ASI-3-PT factor structure, by comparing the fit of the data for a unidimensional and a multidimensional hierarchical approach.

**Methods**

We recruited a sample of 322 college students (mean age = 22.26; SD = 5.75) of which 76.3 % were women. We tested the goodness of fit of two models, conducting a confirmatory factor analysis (CFA). In Model 1, we proposed that Anxiety Sensitivity should be understood as a unidimensional construct, with the items directly contributing to a global score. In Model 2 we proposed that Anxiety Sensitivity is organized into different domains (social concerns, cognitive concerns and physical concerns), with the items contributing to the global construct but also, and to some extent, operating as independent.

**Results**

Results showed Model 2 to be the best fitting, where items loaded onto a first-order factor which in turn loaded onto a second-order factor which represents anxiety sensitivity. Model 1 had a poor fit to the data (χ2 135 = 787.08, p < 0.001, CFI = 0.79; RMSEA = .12 (90 %, CI = .11; .13), while the fit indices of Model 2 were satisfactory (χ2 132 = 402.26, p < 0.001) CFI = .91, RMSEA = .08; (90 % CI = .07; .09).

**Conclusions**

In sum, the ASI-3-PT is an instrument aiming to measure the Anxiety Sensitivity construct. Factorial analysis suggests it can be used as a screening tool, assessing an Anxiety Sensitivity Global Index as well as the Anxiety Sensitivity subscales of Physical, Social and Cognitive Concerns.

**Keywords**

Anxiety Sensitivity Index-3-PT, Confirmatory Factor Analysis, Assessment

#### O181 **Lifestyles of higher education students: the influence of self-esteem and psychological well-being**

##### Armando Silva^1^, Irma Brito^1^, João Amado^2^

###### ^1^Escola Superior de Enfermagem de Coimbra, 3046-851 Coimbra, Portugal; ^2^Universidade Católica Portuguesa – Porto, 4202-401 Porto, Portugal

####### **Correspondence:** Armando Silva (armandos@esenfc.pt) – Escola Superior de Enfermagem de Coimbra, 3046-851 Coimbra, Portugal

**Background**

The transition to higher education can affect lifestyle-related factors. Objectives: To identify lifestyles of higher education students and analyse the influence of self-esteem and psychological well-being.

**Methods**

Correlational cross-sectional study. A total of 4,314 students participated in the study. Online questionnaires were used: Estilo de Vida Fantástico (Fantastic Lifestyle Assessment) [1]; Questionário de Bem-estar Psicológico (Psychological General Well-Being Questionnaire) [2], and Escala de auto-estima de Rosenberg (Rosenberg Self-Esteem Scale [3].

**Results**

Most students (85.3 %) have a healthy lifestyle. Lifestyle is strongly correlated with self-esteem and psychological well-being (p < 0.001). While analysing the association between self-esteem and psychological well-being and the various lifestyle domains according to gender, a positive and significant correlation (p < 0.001) was found among female students, except for the Smoking domain (p = 0.393); in relation to psychological well-being, positive correlations were found in all domains. Among male students, positive and significant correlations (p < 0.001) were found in most lifestyle domains and self-esteem, except for the Smoking (p = 0.992), Alcohol and other drugs (p = 0.181) and Other behaviours (p = 0.442) domains; in relation to psychological well-being, positive and significant correlations (p < 0.001) were found in most lifestyle domains, except for the Smoking (p = 0.458) and Other behaviours (p = 0.128) domains.

**Conclusions**

Based on the results, higher education institutions should support intervention projects to maintain high levels of psychological well-being and self-esteem, promoting healthy lifestyles.

**References**

1. Silva A, Brito I, Amado J. Tradução, adaptação e validação do questionário Fantas¬tic Lifestyle Assessment em estudantes do ensino superior. Ciência & Saúde Coletiva. 2014; 19(6): 1901-1909.

2. Rainho M, Antunes M, Carvalho A, Barroso I, Monteiro M, Mateus S. Bem--estar psicológico e perceção de saúde geral em estudantes do ensino superior. Revista de Enfermagem Referência. 2012; 3(Supl.): 344.

3. Santos PJ, Maia J. Análise factorial confirmatória e validação preliminar de uma versão portuguesa da escala de auto-estima de Rosenberg. Psicologia: Teoria, Investigação e Prática. 2003; 2: 253-28.

**Keywords:** lifestyle, self-esteem, students

#### P75 Assessing the quality of life of persons with significant intellectual disability: Portuguese version of Escala de San Martín

##### António Rodrigo, Sofia Santos, Fernando Gomes

###### Faculdade de Motricidade Humana, Universidade de Lisboa, Cruz quebrada, 1499-002 Lisboa, Portugal

####### **Correspondence:** Sofia Santos (sofiasantos@fmh.ulisboa.pt) – Faculdade de Motricidade Humana, Universidade de Lisboa, Cruz quebrada, 1499-002 Lisboa, Portugal

**Background**

Recognized as an important measure of outcome of health intervention, the assessment of quality of life (QOL) is only now being used in Portugal in the field of intellectual disability (ID) and has never been addressed to persons with permanent support needs/significant disabilities. Therefore, and with the author’s permission, the Escala of San Martin [1] was translated into Portuguese and a psychometric study was initiated.

**Method**

Measuring the content validity of the instruments was one of the first steps to accomplish, which was studied using descriptive (literature review) and empirical evidence (experts’ quantitative analysis). After the processes of translation and back-translation, products were compared and discussed by 10 experts who agreed (after doubts and language clarification) on a pre-final version, which was pre-tested with a sample of 50 institutionalized adults (over 18 years in age) with severe/profound intellectual disability/permanent support needs. 2-3 weeks after, the scale was re-administered to the same participants. Data obtained from the expert committee were analysed.

The Content Validity Index (CVI) was determined for each item, as well as for the entire scale and its average. A minimum of 0.75 was used.

**Results and conclusions**

Results pointed out the relevance of all items, with a moderate/strong agreement between experts. The study’s results are also discussed in terms of reliability and validity. Further research on psychometric properties is being carried out.

**References**

1. Verdugo M, Gomez L, Arias B, Santamaría M, Navallas E, Fernandez S, Hierro, I. Escala San Martín Evaluación de la Calidad de Vida de Personas con Discapacidades Significativas. Santander, España: Fundación Obra San Martin/INICO; 2014.

**Keywords**

Quality of life, severe intellectual disability, cross-cultural adaptation, psychometric properties

#### P76 Childhood obesity and breastfeeding - A systematic review

##### Marlene C. Rosa^1^, Silvana F. Marques^2^

###### ^1^Secção Autónoma das Ciências da Saúde, Universidade de Aveiro, 3810-193 Aveiro, Portugal; ^2^Unidade de Cuidados na Comunidade, Centro de Saúde de Anadia, 3780-780 Anadia, Portugal

####### **Correspondence:** Marlene C. Rosa (marlenerosa@ua.pt) – Secção Autónoma das Ciências da Saúde, Universidade de Aveiro, 3810-193 Aveiro, Portugal

**Background**

Obesity is considered by the World Health Organization as the global epidemic of 21th century and is very common among children. Obesity affects the quality of life of children and related specific interventions are very costly and ineffective. Therefore, there is an urgent need to further understand the determinants of obesity in childhood. The hypothesis that breastfeeding has a protective effect against obesity has been discussed and needs to be further clarified. Objective: To systematize and discuss the most recent research on childhood obesity and breastfeeding.

**Methods**

The following keywords “breastfeeding” AND “early weaning” AND “obesity” were searched on Web of Knowledge, B-On, CINAHL, Nursing Centre, EBSCO-HOST, PubMed, Web of Science, Medline and Lilacs databases. Only literature published between 2004 and 2014 was considered.

**Results**

A total of 158 studies were screened but only six correlated breastfeeding with childhood obesity (<18 years) and therefore were included. These six studies demonstrated that there is an association between breastfeeding and childhood obesity (i.e., early weaning increases the child chances of being obese), despite the high importance of other multifactorial risk factors (e.g., genetic, physiological, environmental, behavioural, cultural and psychological).

**Conclusions**

This systematic review demonstrated the importance of breastfeeding for childhood obesity prevention, but also highlights the relevance of exploring multifactorial obesity etiologic models in future related research.

**Keywords**

Breastfeeding, early weaning, obesity

#### P77 Cross-cultural adaptation of the Foot and Ankle Ability Measure (FAAM) for the Portuguese population

##### Sara Luís^1,2^, Luís Cavalheiro^2,4^, Pedro Ferreira^3,4^, Rui Gonçalves^2,4^

###### ^1^Cáritas Diocesana de Coimbra - Centro Rainha Santa Isabel, 3030-382 Coimbra, Portugal; ^2^Escola Superior de Tecnologia da Saúde de Coimbra, São Martinho do Bispo, 3046-854 Coimbra, Portugal; ^3^Faculdade de Economia, Universidade de Coimbra, 3004-512 Coimbra, Portugal; ^4^Centro de Estudos e Investigação em Saúde, Faculdade de Economia, Universidade de Coimbra, 3004-512 Coimbra, Portugal

####### **Correspondence:** Sara Luís (sararluis@gmail.com) – Escola Superior de Tecnologia da Saúde de Coimbra, São Martinho do Bispo, 3046-854 Coimbra, Portugal

**Background**

Patient-reported outcome measures are unique instruments to assess the impact of a condition on a patient, as well as the efficacy of a treatment. The Foot and Ankle Ability Measure (FAAM) is a specific self-reported measure of the foot/ankle region that measures physical function in musculoskeletal conditions. To our knowledge, there is only one other self-reported measure of this region adapted to Portugal. Objective: To culturally and linguistically adapt the FAAM to the Portuguese population.

**Methods**

The cultural and linguistic adaptation of the FAAM followed a sequential methodology. After gaining authorization of the original author, a sequence of translations and back translations were made and consensus versions were obtained. The last consensus version, similar to the original version with respect to semantics, was delivered to a committee of two experts in the area, who analysed the target version for semantic, idiomatic, experiential and conceptual equivalences, producing a pre-final version. This pre-final version was used on a 10-patient committee with foot/ankle problems, to assess the contents’ equivalence in an applied situation.

**Results**

Semantic equivalence was obtained by the translations, back translation and clinical review. Content equivalence was obtained by the patient committee, with 10 subjects (37.5 years [27-52], 60 % men, with different conditions, occupations and academic qualifications), who described the instrument as “appropriate” and “easy to understand”. The average completion time was 5 minutes.

**Conclusions**

The Portuguese version of FAAM was obtained, with semantic equivalence and content validity with the original version. The remaining psychometric properties are being analysed.

**Keywords**

FAAM, cross-cultural adaptation, foot, ankle, patient-reported outcomes

#### P78 Cross-cultural adaptation of the Patient-Rated Wrist Evaluation score (PRWE) for the Portuguese population

##### Rui S. Lopes^1,2^, Luís Cavalheiro^2,3^, Pedro Ferreira^3^, Rui Gonçalves^2,3^

###### ^1^Cáritas Diocesana de Coimbra - Centro Rainha Santa Isabel, 3030-382 Coimbra, Portugal & Fundação Mário da Cunha Brito, 3360-908 S. Pedro de Alva, Portugal; ^2^Escola Superior de Tecnologia da Saúde de Coimbra, Instituto Politécnico de Coimbra, São Martinho do Bispo, 3046-854 Coimbra, Portugal; ^3^Centro de Estudos e Investigação em Saúde da Universidade Coimbra, Faculdade de Economia, Universidade de Coimbra, 3004-512 Coimbra, Portugal

####### **Correspondence:** Rui S. Lopes (ftruisousa@gmail.com) – Escola Superior de Tecnologia da Saúde de Coimbra, Instituto Politécnico de Coimbra, São Martinho do Bispo, 3046-854 Coimbra, Portugal

**Background**

The importance of patient-reported outcomes (PRO) in assessing the health status of individuals is recognized. The Patient-Rated Wrist Evaluation Score (PRWE) is a specific PRO to assess the impact of pain and functionality relating to problems in the wrist/hand joint complex. Objective: To translate and linguistically validate the PRWE for the Portuguese population.

**Methods**

The cultural adaptation of PRWE followed the sequential methodology. With the authorization of the author of the original measure, we proceeded with translations, back translation and reached consensus of the different versions produced in order to obtain an identical measure to the original from the semantic point of view. The final consensus version, created in this process, was subjected to a clinical review panel (2 experts in the field) to verify the technical and semi-technical terms of the questionnaire. Subsequently, the final version was analysed in a cognitive debriefing that included 9 patients with problems in the wrist/hand joints.

**Results**

Semantic equivalence was obtained by the consensus obtained in translations, back translations and clinical review. The content validity resulted from analysis and consensus of the panel of patients (43.8 ± 22.3 years; 55.5 % male, with several clinical conditions of the wrist/hand, and different professions and qualifications) who described the measure as "accessible", "adequate", "easy to fill out and understand", taking on average 3 minutes to complete.

**Conclusions**

The Portuguese version of PRWE was obtained, with semantic equivalence and content validity to the original measure. The remaining psychometric characteristics must be analysed.

**Keywords**

PRWE, hand, wrist, assessment, cross-cultural adaptation

#### P79 Cross-cultural adaptation of the Myocardial Infraction Dimensional Assessment Scale (MIDAS) for Brazilian Portuguese language

##### Bruno H. Fiorin^1,2,3^, Marina S. Santos^3^, Edmar S. Oliveira^3^, Rita L. Moreira^1^, Elizabete A. Oliveira^3^, Braulio L. Filho^1^

###### ^1^Universidade Federal de São Paulo, São Paulo, 04021-001, Brasil; ^2^Escola superior de Ciências da Santa Casa de Misericórdia de Vitória, Vitória- Esprito Santo, 29045-402 Brasil; ^3^Faculdade Católica Salesiana do Espírito Santo, Vitória- Esprito Santo, 29017-950, Brasil

####### **Correspondence:** Bruno H. Fiorin (bfiorin@salesiano.br) – Faculdade Católica Salesiana do Espírito Santo, Vitória- Esprito Santo, 29017-950, Brasil

**Background**

Quality of life is an important issue for public health, especially because it involves aspects inherent to the individual. MIDAS consists of 35 questions, which feature seven distinct domains associated with patients with AMI. Objectives: To conduct the cross-cultural adaptation of the instrument "Myocardial Infarction Dimensional Assessment Scale" (MIDAS) for the Brazilian Portuguese language.

**Methods**

This is a methodological study, according to Guillemin methodology, concerning the translation and cultural adaptation of the MIDAS instrument. Steps: translation, reverse translation, unification evaluation of authors, a panel of judges and pre-test were used to test the technique for face validity in the pre-test. This study was approved by the CEP UFES (191425/2012).

**Results**

In the translation two translations were obtained, and in comparing them there was agreement in over 90 % of the terms, eleven terms were discussed and evaluated again. The back-translation was sent to the authors, after this assessment, the latest version was referred to a panel of judges to check the semantic, conceptual, cultural and idiomatic features. Seven items were discussed by the judges. The pre-test involved 20 patients, none of whom questioned any of the issues. In addition to the equivalence stated by the judges, it had face validity.

**Conclusions**

It can be said that the MIDAS is culturally adapted to be applied to the population of Brazilian Portuguese language, being an instrument of easy application, useful for clinical and good understanding by the population, with coverage of several fields that permeate the quality of life of the patient after infarction.

**Keywords**

Quality of life, validation studies, questionnaire

#### P80 The revised Portuguese version of the Three-Factor Eating Questionnaire: A confirmatory factor analysis

##### Lara Palmeira^1^, Teresa Garcia^1^, José Pinto-Gouveia^1^, Marina Cunha^2^

###### ^1^Cognitive-Behavioural Research Centre, Faculty of Psychology and Education Sciences, University of Coimbra, Coimbra, 3001-802 Coimbra, Portugal; ^2^Instituto Superior Miguel Torga, Coimbra, 3000-132 Coimbra, Portugal

####### **Correspondence:** Lara Palmeira (larapalmeira@gmail.com) – Cognitive-Behavioural Research Centre, Faculty of Psychology and Education Sciences, University of Coimbra, Coimbra, 3001-802 Coimbra, Portugal

**Background**

The Three-Factor Eating Questionnaire (TFEQ) is one of the most widely used instruments to assess unhealthy eating behaviours. Although the TFEQ original version comprised 51-items, the exploratory factor analysis of the Portuguese version revealed that several items were problematic. In 2000 Karlsoon [1] and collaborators developed a shorter version comprising 18 items (TFEQ-18) that significantly improved the instrument’s psychometric properties. The TFEQ-18 comprises three subscales: cognitive restraint –tendency to restrict food intake; uncontrolled eating – tendency to overeat as a result of a loss of control over the intake and subjective feelings of hunger; emotional eating - tendency to overeat in response to negative emotional states. Objective: The current study aims at performing a confirmatory factor analysis (CFA) of the Portuguese version of the TFEQ-R18 and analyse its psychometric properties.

**Methods**

The sample included 282 women with overweight and obesity seeking nutritional treatment with a mean age of 44 years old (SD = 11.30) and 11.54 (SD = 3.92) years of education. Mean Body Mass Index (BMI) was 31.40 (SD = 4.53).

**Results**

The CFA confirmed the three-factor structure of TFEQ. Three items from the cognitive restraint dimension presented poorer local adjustment and were excluded. The final model revealed an acceptable model fit to the data (GFI = .90, CFI = .93, TLI = .91, RMSEA = .075). Furthermore, all three dimensions presented good psychometric properties.

**Conclusions**

The Portuguese version of the TFEQ comprises 15 items and is a useful, reliable and robust instrument to assess distinct patterns of unhealthy eating behaviours in individuals seeking nutritional treatment.

**References**

1. Karlsson J, Persson LO, Sjöström L, Sullivan M. Psychometric properties and factor structure of the Three-Factor Eating Questionnaire (TFEQ) in obese men and women. Results from the Swedish Obese Subjects (SOS) study. International Journal of Obesity and Related Metabolic Disorders: Journal of the International Association for the Study of Obesity. 2000; 24(12), 1715–1725.

**Keywords**

TFEQ-R18, emotional eating, uncontrolled eating, cognitive restraint, Confirmatory Factor Analysis (CFA)

#### P81 Assessing weight-related psychological inflexibility: An exploratory factor analysis of the AAQW’s Portuguese version

##### Sara Cardoso^1^, Lara Palmeira^1^, Marina Cunha^2^, José Pinto-Gouveia^1^

###### ^1^Cognitive-Behavioural Research Centre, Faculty of Psychology and Education Sciences, University of Coimbra, Coimbra, 3001-802 Coimbra, Portugal; ^2^Instituto Superior Miguel Torga, Coimbra, 3000-132 Coimbra, Portugal

####### **Correspondence:** Lara Palmeira (larapalmeira@gmail.com) – Cognitive-Behavioural Research Centre, Faculty of Psychology and Education Sciences, University of Coimbra, Coimbra, 3001-802 Coimbra, Portugal

**Background**

Experiential avoidance is widely associated with mental and health problems and diminished quality-of-life. Nevertheless, weight-related experiential avoidance patterns are yet little explored. In addition, research suggests the importance of developing content-specific measures to capture relevant contents for specific populations. The Acceptance and Action Questionnaire for Weight-Related Difficulties (AAQW) was developed to target experiential avoidance patterns related to weight thoughts, feelings and actions. Preliminary data suggested a five-factor structure and adequate psychometric properties. Nevertheless, AAQW factor structure still requires further examination. Objective: This study aims to explore the factor structure of the Portuguese version of the AAQW and analyse its psychometric properties.

**Methods**

The sample comprised 249 overweight and obese adult women seeking nutritional treatment, with a mean Body Mass Index (BMI) of 30.88 (SD = 4.22). Sample mean age was 44.45 (SD = 11.03), with a mean of 11.87 years of education (SD = 3.89).

**Results**

The original five-factor structure was not replicated. In fact, a three-factor structure was the better solution explaining 50.94 % of AAQW’s total variance. The Portuguese version of AAQW comprised 15 items distributed in three factors (food as control, emotional avoidance and self-stigma), identical to the ones found in the original study. Furthermore, AAQW showed good internal consistency (α = .81) and convergent validity. In fact, AAQW was related to general psychopathology and psychological inflexibility, eating psychopathological symptoms, including binge eating and decreased obesity-related quality-of-life.

**Conclusions**

Taken together, our findings suggest that the Portuguese version of AAQW seems to be useful and reliable instrument to assess weight-related experiential avoidance.

**Keywords**

Weight-related experiential avoidance, overweight and obesity, exploratory factor analysis, psychometric properties

#### P82 Validation of the Body Appreciation Scale-2 for Portuguese women

##### Joana Marta-Simões, Ana L. Mendes, Inês A. Trindade, Sara Oliveira, Cláudia Ferreira

###### Cognitive and Behavioural Centre for Research and Intervention, Faculty of Psychology and Education Sciences, University of Coimbra, 3000-115 Coimbra, Portugal

####### **Correspondence:** Joana Marta-Simões (joana_simoes_@hotmail.com) – Cognitive and Behavioural Centre for Research and Intervention, Faculty of Psychology and Education Sciences, University of Coimbra, 3000-115 Coimbra, Portugal

**Background**

The concept of positive body image marks a shift on the study of body image. Based on holistic love and respect for the body, this concept involves accepting one’s body’s unique features, attending to body needs in a health-promoting manner, and resisting to the internalization of societally-prescribed beauty ideals. Objectives: Given the lack of Portuguese validated instruments of positive body image, this study aimed to validate the Portuguese version of the Body Appreciation Scale-2 (BAS-2).

**Methods**

The present study used a large female sample, with ages between 18 and 50 years old. An Exploratory Factor Analysis (EFA) and a Confirmatory Factor Analysis (CFA) were conducted to analyse BAS-2’s structure.

**Results**

Results from the EFA demonstrated the BAS-2 to be a unifactorial 10-item scale, which explained approximately 70 % of the variance. Also, all items revealed good factor loadings and the scale showed excellent internal consistency. The BAS-2 also presented temporal reliability, and good convergent (with self-compassion) and divergent validities (with BMI, self-judgment, body image-related experiential avoidance, eating psychopathology, and depression). Moreover, the CFA revealed good local and global adjustments.

**Conclusions**

The BAS-2 was revealed as a short and reliable measure of positive body image, that is, the ability to love and accept one's own body (including the aspects inconsistent with societally-prescribed ideals) and to appreciate its uniqueness. This measure seems to provide an important contribution for research and the development of interventions focused on the cultivation of a balanced, comfortable, confident and happy relationship with one’s own body.

**Keywords**

Body Appreciation Scale, positive body image, scale validation, confirmatory factor analysis, CFA

#### P83 The Portuguese validation of the Dietary Intent Scale

##### Ana L. Mendes, Joana Marta-Simões, Inês A. Trindade, Cláudia Ferreira

###### Cognitive and Behavioural Centre for Research and Intervention, Faculty of Psychology and Education Sciences, University of Coimbra, Coimbra, 3000-115 Coimbra, Portugal

####### **Correspondence:** Ana L. Mendes (analauramendes@live.com.pt) – Cognitive and Behavioural Centre for Research and Intervention, Faculty of Psychology and Education Sciences, University of Coimbra, Coimbra, 3000-115 Coimbra, Portugal

**Background**

Dietary restraint, the intentional restriction of caloric intake for the purpose of weight loss, is assumed of particular concern due to its association with eating disorders, and due to its detrimental effect on health and psychological well-being, even at subclinical levels. Objectives: This study aimed to validate for the Portuguese population the Dietary Intent Scale (DIS), a measure of dietary restraint.

**Methods**

The sample comprised 1,077 participants (415 males and 662 females), aged between 14 and 34 years old.

**Results**

Results revealed that DIS presented a Cronbach’s alpha of .92 and that three items did not contribute for the scale’s internal consistency. Therefore, and since their content was similar to other items, these items were excluded. A confirmatory factor analysis revealed the adequacy of the final 6-item DIS, showing good local (SRWs between .64 and .94) and global adjustments (χ2(8) = 12.07, p = .148; CFI = 1.00; TLI = 1.00; RMSEA = .03, p = .876). Furthermore, the model showed invariance between genders. Finally, DIS revealed and temporal validity (r = .82) and good convergent validity (eating psychopathology, eating restraint, inflexible eating, body image-related experiential avoidance and cognitive fusion, depression, anxiety, and stress)

**Conclusions**

DIS seems to be a valid measure of eating restraint, highly correlated (r = .73) with the instrument that is considered the “golden” measure for eating psychopathology screening and diagnosis (EDE-Q). Moreover, since the DIS only presents 6 items, enabling fast administrations, it may represent an advantage in relation to other existing measures for community research in the field of eating difficulties.

**Keywords**

Dietary Intent Scale, dietary restraint, scale validation, confirmatory factor analysis

#### P84 Construction and validation of the Inventory of Marital Violence (IVC)

##### Filipe Nave (fnave@ualg.pt)

###### Centro de Estudos em Saúde, Escola Superior de Saúde, Universidade do Algarve, 8000-510 Faro, Portugal

**Background**

Marital violence is probably one of the most frequent specific forms of domestic violence. Its impact on the functionality, relationship and affectionateness of families is unquestionable, as well as their repercussions. The main objective of this study is to construct and validate an instrument to assess the type and intensity of marital violence, through brief measures, with objective and easy answers for the general population.

**Methods**

The marital violence inventory (MVI) is the result of a triangulation of methods, in the first qualitative stage in the collection of significant units through interviewing people with and without a history of violence and in the second stage with the use of quantitative methods and transforming these units into 39 items distributed on a Likert-type scale of frequency, on six response levels spread over four scales (domains): psychological, physical, socio-economic and sexual.

**Results**

From the factorial analysis of the results of 504 participants (183 men and 321 women) from the general population, 32 items were kept for study and obtained a five-factor structure with the emergence of the separation of the physical domain into two factors we named Physical Major and Minor; these showed good psychometric capabilities with a KMO between 0.759 and 0.921. In the analysis of reliability (internal consistency, Cronbach's alpha), we obtained values between 0.82 and 9.25.

**Results**

The results of the development and validation test inventory indicate this is an easy response instrument, with evaluative diagnostic capabilities in the health context and which can be applied to Primary and/or Differentiated Health care, or even by social and law enforcement agencies.

**Keywords**

Marital violence, construction and validation, inventory

#### P85 Portable continuous blood pressure monitor system

##### Mariana Campos^1^, Iris Gaudêncio^1^, Fernando Martins^1^, Lino Ferreira^1,2^, Nuno Lopes^1,3^, Rui Fonseca-Pinto^1,2^

###### ^1^Escola Superior de Tecnologia e Gestão, Instituto Politécnico de Leiria, 2411-901 Leiria, Portugal; ^2^Instituto de Telecomunicações, Instituto Politécnico de Leiria, 2411-901 Leiria, Portugal; ^3^Centre for the Research and Technology of Agro-Environmental and Biological Sciences, University of Trás-os-Montes and Alto Douro, 5001-801 Vila Real, Portugal

####### **Correspondence:** Rui Fonseca-Pinto (rui.pinto@ipleiria.pt) – Instituto de Telecomunicações, Instituto Politécnico de Leiria, 2411-901 Leiria, Portugal

**Background**

Non-invasive assessments of continuous blood pressure variations, imposed by homeostatic mechanisms, establish a major challenge in medical instrumentation. The use of portable and low cost devices, joined with the ability to integrate physiological signals enables continuous monitoring during daily tasks. Objective: This work aims to present a device to measure blood pressure variations by means of electrocardiogram (ECG) and photoplethysmogram (PPG) signals. Moreover, the validation of the derived blood pressure values, were also addressed.

**Methods**

The prototype, based on a low cost development platform (Arduino), acquires the patient ECG and PPG signals to automatically calculate the PTT (pulse transit time). Blood pressure signal is influenced by thickness, diameter and elasticity of the vessel wall and blood density, whose variations are sensed by PTT. The instantaneous heart beat and pressure values can be shown on the prototype display or sent to a remote mobile device. All this information is stored in a standard memory card, in a format that allows subsequent reading and analysis by several software applications.

**Results**

To validate the acquisition system, the device was tested in a set of healthy individuals during daily tasks. The robustness of the system was compared with a commercial equipment BIOPAC MP35, and both results match.

**Conclusions**

According to the results, this system could be a low cost and portable alternative to assess continuous blood pressure variations to be used in several clinical evaluations related with blood pressure changes.

**Keywords**

Blood pressure monitor, PTT, non-invasive assessments, Low Cost Device, Portable Medical Device

#### P86 Construction and validation of the Scale of Perception of the Difficulties in Caring for the Elderly (SPDCE)

##### Rogério Rodrigues^1^, Zaida Azeredo^2^, Corália Vicente^3^

###### ^1^Escola Superior de Enfermagem de Coimbra, Coimbra, 3046-851 Coimbra, Portugal; ^2^Research in Education and Community Intervention, Escola Superior de Saúde Jean Piaget, 4405-678 Gulpilhares, Vila Nova de Gaia, Portugal; ^3^Instituto de Ciências Biomédicas Abel Salazar, Universidade do Porto, 4050-313 Porto, Portugal

####### **Correspondence:** Rogério Rodrigues (rogerio@esenfc.pt) – Escola Superior de Enfermagem de Coimbra, Coimbra, 3046-851 Coimbra, Portugal

**Background**

Clinical practice influences the perceptions of nursing students about the needs of the elderly and also difficulties in the care of elderly persons. Objective: To develop a self-administered questionnaire exploring nursing students’ perceptions of the difficulties in caring for the elderly (SPDCE).

**Methods**

The development of the scale proceeded through three phases: the first phase was a literature review and exploratory interview with students after clinical training. The second phase was related to the definition of theoretical dimensions and design of the tool; the review of instruments by a panel of specialists and the pre-test were part of the third phase. The study was designed to select items, identify dimensions, measure reliability, content and construct validity.

**Results**

The SPDCE consisted of 34 items. Factorial analysis grouped the items into three categories that define areas in for the elderly: a) physical health, mental health and activities of daily living subscale, b) social and economic resources subscale c) services use subscale. Cronbach’s alpha was 0.98 for the entire questionnaire. The factor solution explained 75.8 % of the variance.

**Conclusions**

The SPDCE appears to measure with adequate reliability and validity the attributes of exploring nursing students’ perception of the difficulties in caring for the elderly within the clinical setting. The instrument is easy to complete, making it a suitable instrument for nursing teachers to evaluate nursing students’ self-perceived difficulties in nursing care for the elderly as a means to support and sustain conditions for quality of the care provided to the elderly.

**Keywords**

Nursing students, Geriatrics, Gerontology

#### P87 Development and validation of a comfort rating scale for the elderly hospitalized with chronic illness

##### Joana Silva^1^, Patrícia Sousa^1^, Rita Marques^1,2^

###### ^1^Universidade Católica Portuguesa, Lisboa, 1649-023 Lisboa, Portugal; ^2^Hospital de Santa Maria, Centro Hospitalar Lisboa Norte, 1649-035 Lisboa, Portugal

####### **Correspondence:** Joana Silva (jo.cunhaesilva@gmail.com) – Universidade Católica Portuguesa, Lisboa, 1649-023 Lisboa, Portugal

**Background**

The growing demand of older people to health services and the increasing number of elderly people living with chronic illness makes us reflect on the importance of providing nursing care that responds to the real needs of the elderly in hospital context. The Comfort is one of the needs of the elderly hospitalized with chronic disease [1] and the lack of an instrument to measure comfort level, was the main motivation for the development of this study. Objectives: To develop and validate an instrument able to measure the comfort of elderly hospitalized patients with chronic illness.

**Methods**

There has been conducted a methodological research study. The sample has 70 hospitalized patients with at least one chronic disease. Data collection was performed by using a questionnaire outlining demographic and social health issues and the application of the comfort rating scale built by us.

**Results**

Patients were aged between 65 and 99 years; 54.3 % were women and 45.7 % were men. The majority of the patients (65 %) had heart disease and 37.1 % felt that their health situation is bad. The fear of remaining sick and becoming a burden for the family decreased the comfort level of the patients. On the other hand, the hope of getting better and the opportunity to talk with other patients were factors that had a positive effect on comfort level.

**Conclusions**

The results highlight that elderly hospitalized patients with chronic disease have several comfort needs, and that it is crucial to measure them with the appropriate scale.

**References**

1. Sousa P. O Conforto da Pessoa Idosa. Lisboa. Lisboa: Universidade Católica Editora; 2014.

**Keywords**

Comfort, Chronic Disease, Validation Scale, Elderly, Hospitalized

#### P88 Construction and validation of the Postpartum Paternal Quality of Life Questionnaire (PP-QOL)

##### Isabel Mendes^1^, Rogério Rodrigues^1^, Zaida Azeredo^2^, Corália Vicente^3^

###### ^1^Escola Superior de Enfermagem de Coimbra, Coimbra, 3046-851 Coimbra, Portugal; ^2^Research in Education and Community Intervention, Escola Superior de Saúde Jean Piaget, 4405-678 Gulpilhares, Vila Nova de Gaia, Portugal; ^3^Instituto de Ciências Biomédicas Abel Salazar, Universidade do Porto, Porto, 4050-313 Porto, Portugal

####### **Correspondence:** Isabel Mendes (isabelmendes@esenfc.pt) – Escola Superior de Enfermagem de Coimbra, Coimbra, 3046-851 Coimbra, Portugal

**Background**

Paternal quality of life in postpartum is at present a priority in studies of the family cycle, with the purpose of defining and facilitating integrative interventions for their adjustment to the parental role. Objective: to develop a self-administered questionnaire to assess the quality of paternal life postpartum.

**Methods**

The development of the scale proceeded in three phases: I) literature review and exploratory interview to students after clinical training; II) definition of theoretical dimensions and design of the tool; II) review of instruments by a panel of specialists and the pre-test made the third phase. The study was designed to select items, identify dimensions, measure reliability, content and construct validity.

**Results**

The Postpartum Paternal Quality of Life Questionnaire (PP-QOL) consisted of 35 items. A factorial analysis grouped the items into five categories of paternal quality of life: a) psychological/baby; b) socioeconomic; c) relational/partner; d) family/friends relational; e) health/functional status. Cronbach’s alpha was 0.74 for the entire questionnaire. The factor solution explained 51.8 % of the variance.

**Conclusions**

Results emerging from the preliminary study validate the instrument of postpartum paternal quality of life questionnaire (PP-QOL) and allow its use in the context of clinical practice, specifically in the context of primary health care, in order to identify the real needs of fathers and the allocation of services to this particular group of care in the area of reproductive health, with implications for their quality of life after the birth of their offspring.

**Keywords**

Quality of life, postpartum, paternal status, validation

#### P89 Infrared thermal imaging: A tool for assessing diabetic foot ulcers

##### Ricardo Vardasca^1^, Ana R. Marques^1^, Adérito Seixas^2^, Rui Carvalho^3^, Joaquim Gabriel^1^

###### ^1^Faculdade de Engenharia, Universidade do Porto, 4200-465 Porto, Portugal; ^2^Universidade Fernando Pessoa, Porto, 4249-004 Porto, Portugal; ^3^Centro Hospitalar do Porto, Porto, 4099-001 Porto, Portugal

####### **Correspondence:** Ricardo Vardasca (ricardo.vardasca@fe.up.pt) – Faculdade de Engenharia, Universidade do Porto, 4200-465 Porto, Portugal

**Background**

Diabetes Mellitus is a condition that affects 14 % of the Portuguese population. One in every 4 patients develops diabetic foot ulcers (DFU) due to poor blood flow, which can lead to cell death and consequent amputations. This severely affects the quality of life of patients and contributes to significant costs to health systems. Objective monitoring methods are needed to predict their occurrence and assess treatments.

**Methods**

Infrared thermal imaging is a modality for capturing and recording skin surface temperature (SST) in a non-contact, non-invasive, non-ionizing, inexpensive and fast manner. SST is associated with peripheral blood flow, which is controlled by the autonomous nervous system. Planar foot incidences were taken from 40 healthy controls and 80 patients with different types of diabetic foot ulcers: ischemic, neuropathic and neuroischemic. Regions of interest (ROI) were measured and thermal symmetries calculated against the non-affected limb.

**Results**

Results demonstrated that in patients developing and with DFU there is a higher value of thermal symmetry than in healthy controls at same ROIs location (p < 0.05).

**Conclusions**

The findings of this study provide valuable indicative information to use this method for screening patients at risk of developing diabetic foot ulcers, since there is an inflammatory process that increases SST before the occurrence of the wound and for treatment assessment, since the SST decreases significantly when there is no neuropathic and vascular activity in a necrotic situation. This method can be used to reduce the occurrence of diabetic foot ulcers and consequent decrease in the collateral costs to health care systems.

**Keywords**

Diabetes, Foot Ulcers, Infrared Thermal Imaging, Skin Temperature

#### P90 Pressure ulcers in an intensive care unit: An experience report

##### Paulo P. Ferreira^1^, Michelle T. Oliveira^2^, Anderson R. Sousa^3^, Ana S. Maia^3^, Sebastião T. Oliveira^3^, Pablo O. Costa^3^, Maiza M. Silva^3^

###### ^1^Secretaria de Saúde do Estado da Bahia, Salvador - Bahia, 41745-900, Brasil; ^2^Universidade Estadual de Feira de Santana, Feira de Santana - Bahia, 44036-900, Brasil; ^3^Faculdade Nobre, Feira de Santana - Bahia, 44001-008, Brasil

####### **Correspondence:** Paulo P. Ferreira (paulopanelli@gmail.com) – Secretaria de Saúde do Estado da Bahia, Salvador - Bahia, 41745-900, Brasil

**Background**

Pressure ulcers (PU) are developed due to pathological changes in blood perfusion of the skin and underlying tissues. The risk of developing ulcers becomes higher in patients in the ICU, because besides the mobility limitations imposed on patients for their clinical condition, the need for more stringent controls, associated with more complex therapies, there are other risk factors in addition, such as sedatives, level of consciousness changes, use of vasoactive drugs and hemodynamic instability.

The aim of this study is to report the experience in handling PU in an ICU of a public hospital in the interior of Bahia.

**Methods**

This is a descriptive study-type experience report, about the professional experience of intensive care in an ICU of the General Hospital, Feira de Santana, Bahia. The report relates the experiences of the year 2015.

**Results**

It observed a high rate of incidence and prevalence of PU in the ICU, with the regions most affected being the sacral and trochanter. Also it has been identified as occurring in sites with low prevalence in population studies, such as the occipital region and calves.

**Conclusions**

These alarming data refer to a sense of duty that the provider has to be aware of the consequences that the PU can trigger for the patient, his family and the institution. It prolongs hospital stays, make recovery difficult, increases the risk of development of complications and represents an increase in the physical and emotional suffering of patients by reducing their independence in carrying out daily activities.

**Keywords**

Pressure ulcers, intensive care unit, prevalence

#### P91 Validation of figures used in evocations: instrument to capture representations

##### Cristina Arreguy-Sena^1^, Nathália Alvarenga-Martins^1^, Paulo F. Pinto^1^, Denize C. Oliveira^2^, Pedro D. Parreira^3^, Antônio T. Gomes^2^, Luciene M. Braga^4^

###### ^1^Universidade Federal de Juiz de Fora, Juiz de Fora, Minas Gerais, 36036-330, Brasil; ^2^Universidade do Estado do Rio de Janeiro, Rio de Janeiro, 20550-900, Brasil; ^3^Escola Superior de Enfermagem de Coimbra, Coimbra, 3046-851, Portugal; ^4^Universidade Federal de Viçosa, Viçosa, Minas Gerais, 36570-900, Brasil

####### **Correspondence:** Cristina Arreguy-Sena (cristina.arreguy@ufjf.edu.br) – Universidade Federal de Juiz de Fora, Juiz de Fora, Minas Gerais, 36036-330, Brasil

**Background**

The difficulty of implementation of the association technique, using the inductive term (word or phrase) activated by speech, can derail a deal, when there is impoverishment or disagreement on the vocabulary/understanding of the term used, or temporary or permanent cognitive restriction, from the one evoking. Objectives: To create and culturally adapt images to support the preparation of the free word association technique, validating them for use among low-educated elderly people from the perspective of urinary incontinence in the aging process.

**Methods**

Cultural validation of images for contextual reality, operationalized in: I) creation of images to portray the chosen everyday situations; II) obtaining consensus for these images by a committee of experts and people from the community, using the Delphi technique and III) Technical application of the free word association with and without image support (Technique of Free Word Association Initiated by Images- “TALPDI”).

**Results**

Five slides were designed with images covering thematic diversity after a ≥ 90 % consensus to ensure full agreement among evaluators. Fifty (50) elderly people participated. The comparison of the non-use/use of images between participants improved evocation of 93.2 %.

**Conclusions**

The creation and cultural validation of images favoured the capture of representational content using the TALPDI.

**Keywords**

Aged, methods, nursing, urinary incontinence, social Representation, validation

#### P92 Telephone assistance to decrease burden in informal caregivers of stroke older people: Monitoring and diagnostic evaluation

##### Odete Araújo^1^, Isabel Lage^1^, José Cabrita^2^, Laetitia Teixeira^3^

###### ^1^Escola Superior de Enfermagem, Universidade do Minho, Braga, 4710-057 Braga, Portugal; ^2^Faculdade de Farmácia, Universidade de Lisboa, 1649-003 Lisboa, Portugal; ^3^Instituto de Ciências Biomédicas Abel Salazar, Universidade do Porto, 4050-313 Porto, Portugal

####### **Correspondence:** Odete Araújo (odete.araujo@ese.uminho.pt) – Escola Superior de Enfermagem, Universidade do Minho, Braga, 4710-057 Braga, Portugal

**Background**

After hospital discharge many stroke survivors return home and need support with at least one activity of daily living. Most of them require help from informal caregivers who are often unprepared for the demands of providing care. Evidence has shown that combining enhanced informal caregivers’ practical skills, face-to-face interventions and telephone support can reduce negative consequences for caregivers, such as burden or depression. Objective: To evaluate the impact of an intervention to reduce burden by using telephone support delivered to informal caregivers, who take care of older people after a stroke, at home.

**Methods**

A quasi-experimental method, which included 3 months of follow-up, was conducted with 174 patients. The Control group (n = 89) received usual care available provided by healthcare units and the InCARE programme was implemented in the experimental group (n = 85), 1 week, 1 and 3 months, telephone support, counselling caregivers on the 3rd, 6th, 8th and 10th weeks at home. It aimed to facilitate the caregiver’s adjustment to stroke demands, increasing knowledge and practical skills to support their decision-making. Data collection took place between February 2014 and December 2014.

**Results**

The experimental group had a significant lower level of burden in comparison with the control group 1 and 3 months after InCARE intervention.

**Conclusions**

Telephone support does not replace face-to-face interventions implemented based on structured programs, however, they are an aid for diagnosis and an important complement to all types of interventions that promote health gains of this group of informal caregivers and stroke survivors.

**Trial registration**

Current Controlled Trials NCT02074501

**Keywords**

Stroke survivors, elderly, informal caregivers, burden, pilot study

#### P93 Hope of informal caregivers of people with chronic and advanced disease

##### Rita Marques^1^, Mª Anjos Dixe^2^, Ana Querido^2^, Patrícia Sousa^3^

###### ^1^Hospital de Santa Maria, Centro Hospitalar Lisboa Norte, 1649-035 Lisboa, Portugal; ^2^Health Research Unit & School of Health Sciences, Polytechnic institute of Leiria, 2411-901 Leiria, Portugal; ^3^Universidade Católica Portuguesa, Lisboa, 1649-023, Portugal

####### **Correspondence:** Rita Marques (ritamdmarques@gmail.com) – Hospital de Santa Maria, Centro Hospitalar Lisboa Norte, 1649-035 Lisboa, Portugal

**Background**

Hope is a crucial factor in the lives of those who care for people with chronic and advanced disease. Therefore, the main objective of this study was to identify the predictors of hope of informal caregivers of people with chronic and advanced disease.

**Methods**

This is a correlational study conducted with 314 caregivers of people with advanced and chronic disease who were admitted in medical services or on an outpatient regime in Portuguese hospitals. We conducted a structured interview to collect demographic data, caring experience and health of patient and caregiver, hope (Hope Herth Index, HHI-C) comfort (Holistic Comfort Questionnaire, HCQ-C) and quality of life (Quality of Life in Life Threatening Illness, QOLLTI-C). Throughout the study, Helsinki Declaration Principles were followed.

**Results**

The 314 caregivers, mostly female (84.1 %) with an average of 63 years old (±11) had good levels of hope (3.06 ± 0.49), comfort (4.23 ± 0.83) and quality of life (6.15 ± 1.12). The caregivers interviewed took care of patients mainly with neoplasms (85.0 %) and severe dependence, for about 17.2 months (±16.25) and they spent about 6.2 (±3.79) hours a day taking care of them. They considered their health as poor (7.83 ± 1.8) and felt quite tired (7.75 ± 1.79). After performing a multiple linear regression, we verified that the explanatory variables of caregivers’ hope were: comfort, quality of life, time spent on caring, health status, fatigue and self-perception of their own health.

**Conclusion**

Nursing interventions should be focused on caregivers' comfort, quality of life and health status in order to maintain their hope while caring for their relatives.

**Keywords**

Hope, informal caregivers, person with advanced and chronic disease, influencing variables

#### P94 Functionality and quality information from the Portuguese National Epidemiological Surveillance System

##### Sara Silva, Eugénio Cordeiro, João Pimentel

###### Unidade de Saúde Pública, Centro Hospitalar do Baixo Vouga, Aveiro, 3814-501, Portugal

####### **Correspondence:** Sara Silva (sararebelo.silva@gmail.com) – Unidade de Saúde Pública, Centro Hospitalar do Baixo Vouga, Aveiro, 3814-501, Portugal

**Background**

In Portugal, the National System of Communicable Diseases Surveillance is a health information system for the monitoring of a number of communicable diseases and other risks to public health. The existence of a list of communicable diseases, now updated, dates back to 1949. With the introduction of a new communicable diseases information system in June 2014, it would be appropriate to evaluate its functionality and quality. Objective: The aim of this study was identify the quality of the information recorded into the National System of Communicable Diseases Surveillance.

**Methods**

The evaluation of filling data was made based on attributes adapted from the rules used for evaluating surveillance systems in Public Health of the Centres for Disease Control and Prevention.

**Results**

Based on the attributes "Simplicity/Acceptability" and "Completeness" it was found that full information was filled in 77.4 % of the cases; in relation to the "Data Quality” it was found that in 65.0 % of the cases the information was properly filled. In 90.5 % of the cases was observed "Information Consistency" and in 51.8 % of the cases "Information Conformity". As to "Transmission Timely" information, it has been found that, depending on the user input, it should be faster.

**Conclusions**

It can be concluded that it is an innovative system with great potential for improving and very useful for the rapid transmission of information, communication between the different levels and quality validation and upcoming reviews and system upgrades are expected.

**Keywords**

Attributes, communicable diseases, health information system

#### P95 Resting metabolic rate objectively measured vs. Harris and Benedict formula

##### Vera Ferro-Lebres, Juliana A. Souza, Mariline Tavares

###### Escola Superior de Saúde, Instituto Politécnico de Bragança, Bragança, 5300-253, Portugal

####### **Correspondence:** Vera Ferro-Lebres (vferrolebres@gmail.com) – Escola Superior de Saúde, Instituto Politécnico de Bragança, Bragança, 5300-253, Portugal

**Background**

Resting Metabolic Rate (RMR) is the energy spent in activities necessary to maintain normal homeostasis and body functions. In the XX century, Harris and Benedict conducted a study using calorimetry to the measurement of basal metabolic rate. This study analysed physical variables (age, weight, height) and physiological data expressing the results in two formulas, one for males and another for females. Studies indicate that the data obtained in the equations are the same as those obtained by indirect calorimetry, however other studies question these results. Objective: Study the correlation between the RMR using indirect calorimetry and Harris-Benedict formula in a group of workers of granite industry.

**Methods**

A quantitative cross-sectional study was developed. Data collection was performed using a sample of 30 men from two companies of granite industry. Data collection was done using anthropometric measurements and the RMR assessed using the Fitmate, Cosmed®. Statistical analysis of the data was performed using the statistical software Statistical Package for Social Sciences version 22.

**Results**

The sample comprised 30 men, with a mean age of 39.8 (SD = 13.9) years. The RMR measured was in average of 1,741.0 (SD = 281.7) Kcal, and the Harris and Benedict Formula resulted in an average of 1,785.0 (SD = 229.2) Kcal. RMRs objectively measured and calculated varied on average 4.1 (SD = 14.6) % and were significantly correlated (=0.539; p-value = 0.01).

**Conclusions**

The Harris-Benedict formula overestimates the RMR value at around 4.1 %, as previously mentioned.

**Keywords**

Resting metabolic rate, Fitmate, Harris and Benedict formula

### Sustainable integrated user-centred care

#### O182 Characteristics of non-urgent patients: Cross-sectional study of an emergency department

##### Mª Anjos Dixe^1^, Pedro Sousa^1^, Rui Passadouro^2^, Teresa Peralta^2^, Carlos Ferreira^2^, Georgina Lourenço^2^

###### ^1^School of Health Sciences, Polytechnic institute of Leiria, 2411-901 Leiria, Portugal; ^2^Hospital de Santo André, Centro Hospitalar de Leiria, 2410-197 Leiria, Portugal

####### **Correspondence:** Pedro Sousa (pedro.sousa@ipleiria.pt) – School of Health Sciences, Polytechnic institute of Leiria, 2411-901 Leiria, Portugal

**Background**

The use of Emergency Departments (ED) by non-urgent patients has shown a significant increase, creating major challenges for the health system, as well as political and financial constraints. Objectives: a) to characterize the profile of non-urgent ED users; b) to identify the determinants of non-urgent ED use.

**Methods**

This cross-sectional study included 357 non-urgent users of a Portuguese ED. Data collection was conducted through questionnaire and electronic medical records. Results were analysed using descriptive statistics and parametric tests. This study has been approved by the ethics committee of the hospital.

**Results**

Data analysis showed that non-urgent users are mostly female, retired, with lower education levels and with an average age of 54.51 years. Most users visited the ED by their own initiative, and only 18.3 % sought previously the primary healthcare. Most of them had access to a family doctor and the clinical motivations for the ED visit lasted for 18.8 days. Patients who considered the ED as the most appropriate service to treat their situation, had a higher number of previous ED admissions (p < 0.05). Abusive ED use seems to be associated with a high consumption pattern of global healthcare. Several reasons for the non-urgent ED use were identified.

**Conclusions**

A public discussion involving clinical, political and community stakeholders is imperative. Non-urgent users’ characteristics should be taken into account when designing a program against the misuse of ED, namely regarding the timely identification of hyperusers and the adequacy of the healthcare accessibility.

**Keywords**

Non-urgent patients, Emergency Department, Healthcare

#### O183 Physical fitness and health in children of the 1st Cycle of Education

##### João Serrano, João Petrica, Rui Paulo, Samuel Honório, Pedro Mendes

###### Escola Superior de Educação, Instituto Politécnico de Castelo Branco, 6000-767 Castelo Branco, Portugal

####### **Correspondence:** João Serrano (j.serrano@ipcb.pt) – Escola Superior de Educação, Instituto Politécnico de Castelo Branco, 6000-767 Castelo Branco, Portugal

**Background**

The interest of researchers on the assessment of physical fitness of children is crucial in a society where the activity/motion constraints are higher and may affect their health. Objectives: I) to study the impact of an oriented programme of physical activity through “Curricular Enrichment Activities” on the physical fitness of children and II) to observe if the results are within healthy regular parameters.

**Methods**

We used as measuring instrument the battery of tests of Fitnessgram©, with methodological procedures according to The Cooper Institute's Application Manual for Aerobics Research [1]. The sample consisted of seventy children attending the 1st Cycle of Education, aged between 6 and 9 years old, with data collected in 2 separate moments with a time interval of three months. Statistical procedures used in the comparison of variables were performed through the chi-square and McNemar tests.

**Results**

In both moments of evaluation most children obtained test results that may be considered appropriate, in terms of healthy and good organic functions. The test results evidenced a statistically significant improvement between the two moments of evaluation.

**Conclusions**

Children improved their physical fitness levels from the 1st to the 2nd moment of evaluation. Thus, this leads us to conclude that the intervention developed during the programme of “Curricular Enrichment Activities” brought benefits and a positive impact on their health levels.

**References**

1. The Cooper Institute for Aerobics Research. Fitnessgram. Manual de aplicação de testes. Lisboa: Faculdade de Motricidade Humana Ed. 2002.

**Keywords**

Physical Fitness, motricity child, physical activity and health, studies of the child

#### O184 The impact of physical activity on sleep quality, in children

##### Alexandra Simões, Lucinda Carvalho, Alexandre Pereira

###### Escola Superior de Saúde Dr. Lopes Dias, Instituto Politécnico de Castelo Branco, 6000-767 Castelo Branco, Portugal

####### **Correspondence:** Alexandra Simões (alexandraalsimoes@hotmail.com) – Escola Superior de Saúde Dr. Lopes Dias, Instituto Politécnico de Castelo Branco, 6000-767 Castelo Branco, Portugal

**Background**

Several factors contribute, positively or negatively, for the quality of sleep. One of the positive factors is regular physical activity. Physical activity should be perceived as having multiple benefits and not be reduced to the evident and proved results for the body and heart. Objective: To estimate the impact of physical activity in the quality of sleep in school-age children from 7 to 9 years old.

**Methods**

The sample was collected in the several existing learning centres of the city of Almeirim, consisting of 128 individuals (55.5 % feminine and 44.5 % masculine) in 2014. The data collection for the study consisted in the use of two questionnaires: Children Sleep Habits Questionnaire and Sleep Self Report.

**Results**

90.6 % of the sample practices weekly physical activity and 60.9 % shows a sleep disorder, being more prevalent among the female gender. It was possible to infer that the older the children were, the less probability they had for having a sleep disorder.

**Conclusions**

There isn’t any positive relationship between the weekly practice of physical activity and a sleep disorder. Children who practice light, moderate or vigorous physical activity can show a reduction in the quality of their sleeping patterns. Nevertheless, the results highlight a considerable percentage of children with some kind of disorder, which may signal a possible underreporting of the lack of quality sleep of this population.

**Keywords**

Quality of sleep, physical activity, school-aged children, learning centres, questionnaire

#### O185 What is the potential for using Information and Communication Technologies in Arterial Hypertension self-management?

##### Sara Silva^1^, Paulino Sousa^2,3^, José M. Padilha^2^

###### ^1^Centro Hospitalar do Porto, Porto, 4099-001, Portugal; ^2^Escola Superior de Enfermagem do Porto, Porto, 4200-072, Portugal; ^3^Center for Health Technology and Services Research, Faculty of Medicine, University of Porto, Porto, 4099-002, Portugal

####### **Correspondence:** Sara Silva (sara_pereira_s@hotmail.com) – Centro Hospitalar do Porto, Porto, 4099-001, Portugal

**Background**

Arterial Hypertension (HTA) is a chronic disease with high morbidity, mortality and a socio-economic impact. Information and Communication Technologies (ICT) facilitate the access to the necessary information for HTA self-management. Nowadays, there is a lack of knowledge of the utility, ease and interest expressed by people with HTA for the use of ICTs in accessing the information for the disease’s self-management. Objective: to characterize both ICT’s potential and HTA patients’ global needs for information.

**Methods**

Quantitative, exploratory, descriptive and cross-sectional study using phone interviews and a stratified random probability sample of 391 patients on the National Program of Cardiovascular Disease Prevention list within a Local Portuguese Healthcare Unit.

**Results**

People with HTA manifest a higher information need not only to integrate self-management in their everyday life, but also to motivate the significant people to help them in their daily life and to increase their knowledge on the available resources within the community. People with a lower education level ultimately display a lower technological literacy, lower access, lower use and more difficulty in the use of information resources, preferring the use of voice and image devices. People with a higher education level and technological literacy are younger and admit to use and value more Web-Based technologies.

**Conclusions**

Younger and higher educated people present a higher potential use of ICTs. Although displaying a higher intent towards the use of voice devices, older and less educated people mention having significant people that may help them in accessing the health information through ICTs.

**Keywords**

Arterial Hypertension, eHealth, technological literacy, health literacy, Information and Communication Technologies, Self-management

#### O186 Exploring psychosocial factors associated with risk of falling in older patients undergoing haemodialysis

##### Daniela Figueiredo, Carolina Valente, Alda Marques

###### School of Health Sciences, University of Aveiro, 3810-193 Aveiro, Portugal

####### **Correspondence:** Daniela Figueiredo (daniela.figueiredo@ua.pt) – School of Health Sciences, University of Aveiro, 3810-193 Aveiro, Portugal

**Background**

Fall rates have been found to be higher in haemodialysis (HD) patients than in the general older population. Post-dialysis fatigue, polypharmacy, dialysis related hypotension, chronic kidney disease-mineral and bone disorder, have been recognised as risk factors for falls peculiar to HD. However, little attention has been paid to the psychosocial factors related to falls risk in this population. Objectives: This study aimed to analyse the association between falls risk and psychosocial variables (anxiety, depression and social isolation) among older adults undergoing HD.

**Methods**

A cross-sectional study was conducted. Sociodemographic and health-related data were collected through a structured questionnaire. Risk of falling was assessed with the Five Times Sit to Stand (FTSS) test and isometric muscle force (IMF). Anxiety and depression were assessed with The Hospital Anxiety and Depression Scale. Social isolation was measured with The Lubben Social Network Scale-6 (LSNS-6). Descriptive and inferential analyses were performed.

**Results**

Seventy-two HD patients (mean age: 62.29 ± 14.5; 69.4 % male) have participated. Falls risk varied according to age, education, self-rated physical and mental health, visual and hearing impairment, and history of falls. Significant statistical differences were found between anxiety and IMF (p = 0.011) and between depression and FTSS (p < 0.01) and IMF (p = 0.033) scores (i.e., falls risk increase with anxiety and depressive symptoms). A significant correlation was observed between IMF and LSNS-6 (rs = 0.368; p < 0.01).

**Conclusions**

The findings suggested that psychosocial factors are related with increased risk of falling among HD older patients. Fall preventive strategies should also include psychological and social support to patients undergoing HD.

**Keywords**

Risk of falling, haemodialysis, older adults, anxiety, depression, social isolation

#### O187 Development of pressure ulcers on the face in patients undergoing non-invasive ventilation

##### Patrícia Ribas^1^, Joana Sousa^1^, Frederico Brandão^2^, Cesar Sousa^2^, Matilde Martins^1,3^

###### ^1^Escola Superior de Saúde, Instituto Politécnico de Bragança, 5300-253 Bragança, Portugal; ^2^Centro Hospitalar Tâmega e Sousa, Penafiel, 4564-007 Penafiel, Portugal; ^3^Centro de Investigação em Desporto, Saúde e Desenvolvimento Humano, Escola Superior de Saúde, Instituto Politécnico de Bragança, Bragança, 5300-253 Bragança, Portugal

####### **Correspondence:** Patrícia Ribas (patriciasofiaribas@gmail.com) – Escola Superior de Saúde, Instituto Politécnico de Bragança, 5300-253 Bragança, Portugal

**Background**

The use of Non-Invasive Ventilation (NIV) has been increasing in clinical practice, however, the evidence has shown that its implementation may lead to development of ulcers of the face. Objective: to determine the prevalence of face ulcers on the face of patients admitted in an Intermediate Care Unit (ICU) submitted to NIV and to identify the factors associated to its development.

**Methods**

A prospective study conducted in a ICU between September and December 2015. Inclusion criteria of: age ≥ 18 years, patients admitted to the ICU, submitted to NIV and without ulcers of the face at the time of admission, lead us to a sample of 30 participants. Data were collected through a questionnaire, the Braden and Glasgow scale.

**Results**

The prevalence of ulcers on the face was of 26.7 % with a mean onset time of 3.3 ± 1.1 days. Participants were mostly males (70 %), with a mean age of 74.2 ± 10.3 years. Those who have developed an ulcer showed an older average age of 76.5 years, 16.7 % were changing sensitivity, 16.7 % had the facial skin intact and dry, 26.7 % used reused masks and 16.7 % had an ulcer Grade II. There was a statistically significant positive correlation between the development of an ulcer with the number of hours of daily NIV, the number of days of NIV, days of hospitalization, and a negative correlation with the level of consciousness.

**Conclusions**

We observed a high prevalence of ulcers. Thus, this emphasizes the need for further research to increase knowledge to subsidize ulcer prevention interventions in patients with NIV.

**Keywords**

Non-invasive ventilation, nose ulcers, facial ulcers, pressure ulcers

#### O188 The elder hospitalized: Limiting factors of comfort

##### Patrícia Sousa^1^, Rita Marques^2^

###### ^1^Universidade Católica Portuguesa, Lisboa, 1649-023 Lisboa, Portugal; ^2^Hospital de Santa Maria, Centro Hospitalar Lisboa Norte, 1649-035 Lisboa, Portugal

####### **Correspondence:** Patrícia Sousa (patriciapontificesousa@gmail.com) – Universidade Católica Portuguesa, Lisboa, 1649-023 Lisboa, Portugal

**Background**

Hospitalization causes significant changes in the lives of the eldery, due to factors such as the environment, changes in routines, loss of functional capacity, among others. Therefore, it is crucial to identify the contextual factors that emerge as discomforting. Objectives: To identify the limiting factors for comfort on hospitalized elderly.

**Methods**

It is a descriptive study using qualitative methods of data gathering, guided by the ethnographic method. Semi-structured interviews were conducted with 20 elderly patients, audio-recorded and submitted for content analysis [1]. The patients were selected from admissions to the medical services of a Central Hospital, in Lisbon. There was participant observation in order to understand the situational experiences, based on previously structured scripts [2].

**Results**

Regarding the action context, the factors that emerged, that cause discomfort included environmental conditions (light, noise, equipment, colour, temperature, natural or artificial elements of the environment); quality of food; the absence of activities as well as the lack of human resources and time to care.

**Conclusions**

Comfort nursing care is challenged by the unpredictability of circumstances surrounding the satisfaction of multiple health needs and resources for caring. The action context, related to all its elements, can be limitative of the humanization and completeness in geriatric comfort care, which has a negative impact on the comforting experience of the elderly in hospital.

**References**

1. Huberman M, Miles, M. Analyse des donnés qualitative. Recueil de nouvelles méthodes. Bruxeles: De Beock; 1991.

2. Spradley JP. The participant observation. New York: Holt Rinehart and Winston; 1980.

**Keywords**

Elderly, hospitalization, limiting factors, comfort

#### O189 Physical activity and health state self-perception by Portuguese adults

##### Francisco Mendes^1^, Rosina Fernandes^1^, Emília Martins^1^, Cátia Magalhães^1^, Patrícia Araújo^2^

###### ^1^Centro de Estudos em Educação, Tecnologias e Saúde, Escola Superior de Educação de Viseu, 3504-501 Viseu, Portugal; ^2^Universidade Católica Portuguesa – Porto, 4202-401 Porto, Portugal

####### **Correspondence:** Rosina Fernandes (rosina@esev.ipv.pt) – Centro de Estudos em Educação, Tecnologias e Saúde, Escola Superior de Educação de Viseu, 3504-501 Viseu, Portugal

**Background**

According to the World Health Organization (2015) [1], physical activity translates into significant health benefits and the lack of its practice constitutes a fundamental risk factor in non-transmitted diseases. Objectives: to determine physical activity levels in adults that go (N = 150) or do not go (N = 206) to a gym and relate them with health self-perception (from mediocre to very good).

**Methods**

Data was collected through the International physical activity questionnaire (IPAQ) and analysed using SPSS 23 for Windows.

**Results**

In accordance with the minimal weekly referential of vigorous (75`) and moderate (150`) practice recommended by WHO, we found that 98.6 % of practitioners achieved the recommendations, against the 54.8 % of non-practitioners. Only 27.6 % of non-practitioners against 63.3 % of practitioners achieves or surpasses the weekly moderate activity referential recommended by WHO for additional health benefits (300`).

In contrast with different health states of participants, some significant differences manifest (p < .05) in frequency and duration of diverse physical activity types, but not in the sitting time. Also, in the same analysis, divided in practitioners and non-practitioner’s subgroups, no significant differences where observed (p < .05)

Health states Good and Very Good revealed themselves statistically in contrast (p > .05) of frequency and duration of moderate and vigorous activities, between practitioners and non-practitioners, with the first having advantage, the same not happening in light activities and in sitting time.

**Conclusions**

Results confirm recent studies [2, 3] and highlight the urgent necessity of promoting physical activity as a health and well-being promoting factor in populations.

**References**

1. World Health Organization. Health topics physical activity. WHO: 2015.

2. Carvalho ED, Valadares AL, da Costa-Paiva LH, Pedro AO, Morais SS, Pinto-Neto AM. Physical activity and quality of life in women aged 60 or older: associated factors. Rev Bras Ginecol Obstet. 2010; 32(9): 433-440.

3. Ogwumike OO, Kaka B, Adegbemigun O, Abiona T. Health-related and socio-demographic correlates of physical activity level amongst urban menopausal women in Nigeria. Maturitas. 2012; 73(4): 349-353.

**Keywords**

Health state, physical activity, IPAQ

#### O190 **Satisfaction with social support in the elderly of the district of Bragança**

##### Carla Grande^1^, Mª Augusta Mata^2^, Juan G. Vieitez^3^

###### ^1^Unidade Local de Saúde do Nordeste, Bragança, 5301-852 Bragança, Portugal; ^2^Escola Superior de Saúde, Instituto Politécnico de Bragança, 5300-121 Bragança, Portugal; ^3^Universidad de León, 24004 León, España

####### **Correspondence:** Carla Grande (carlagrande1@sapo.pt) – Unidade Local de Saúde do Nordeste, Bragança, 5301-852 Bragança, Portugal

**Background**

This is a demographic transition scenario with implications in all areas of social life: it is unacceptable to face aging without reflecting on the problems around opportunities for older people, including satisfaction with social support. Social support is particularly important to facilitate an independent and fulfilling life in the community for the elderly. Objectives: To assess the satisfaction of elderly people with social support (individuals aged over 65 years) living in the district of Bragança, by applying a sociodemographic questionnaire and the Satisfaction Scale with Social Support (ESSS) of Ribeiro (2011) [1].

**Methods**

Starting from the elderly population living in Bragança district, we developed an observational, analytical and cross-sectional study with a quantitative approach, with a sample of 517 subjects, mostly females (54.9 %; n = 284); aged between 65 and 74 years old (45.3 %; n = 234); married or living in consensual union (61.9 %; n = 320); residents in rural areas (69.8 %; n = 361); with sons and daughters (89.0 %; n = 460); and illiterate (50.7 %; n = 262).

**Results and conclusions**

The sample under study has average social support, which allows us to infer that the elderly is, in general, satisfied with the social support which they have, and the support received from friends is that which gives them more satisfaction, expressing less satisfaction with family support. It is observed that most of the variables are, in the case of the elderly, determinants of their satisfaction with social support.

**Keywords**

Elderly, Social Support, Satisfaction

**References**

1. Ribeiro JLP. Escala de Satisfação com o Suporte Social. 2011; Lisboa: Placebo Editora.

#### O191 Prevalence of death by traumatic brain injury and associated factors in intensive care unit of a general hospital, Brazil

##### Bruna Bianchini, Nazare Nazario, João G. Filho, Marcia Kretzer

###### Universidade do Sul de Santa Catarina, Palhoça, Santa Catarina, 88137-270 Brasil

####### **Correspondence:** Nazare Nazario (nazare.nazario@unisul.br) – Universidade do Sul de Santa Catarina, Palhoça, Santa Catarina, 88137-270 Brasil

**Background**

Traumatic brain injury is a major cause of acute brain injury and the leading cause of death among persons aged less than 50 years. Brain Injury is a public health concern that demands ongoing an epidemiological study, increased efforts to prevent occurring injuries, and research to advance medical options and therapeutic interventions. Objective: To assess the factors associated with death by Traumatic Brain Injury in a General Hospital.

**Methods**

Cross-sectional study, conducted at the Regional Hospital of St. Joseph, Brazil, with 75 patients diagnosed with traumatic brain injury admitted to the Intensive Care Unit from January/2010 to December/2011. Analysis by SPSS 20.0, Chi-square test with p ≤ 0.05 and a confidence interval of 95 %. Approved by the Research Ethics Committee of University of Southern Santa Catarina, No 855.750.

**Results**

Prevalence in men (81.3 %), average age of 36.09 years (SD 15.35). The major cause of accidents were traffic accidents (54.1 %). The length of stay in the intensive care unit was 6-15 days (35.8 %), with severe classification in the Glasgow Coma Scale (77.1 %), pneumonia complications (16.0 %), and death in 37.1 %. Significant associations were found between death and severe classification (p = 0.022), period of time in the intensive care unit of 1 to 15 days (p = 0.051), use of a catheter to evaluate intracranial pressure (p = 0.002) and tracheostomy (p = 0.044).

**Conclusions**

Death by traumatic brain injury is associated with aspects related to the severity of the condition, use of catheter to evaluate intracranial pressure and tracheostomy.

**Keywords**

Traumatic brain injury, death, craniocerebral trauma

#### O192 Relation between family caregivers burden and health status of elderly dependents

##### Tânia Costa^1,2^, Armando Almeida^1,2^, Gabriel Baffour^1,2^

###### ^1^Instituto de Ciências da Saúde, Universidade Católica Portuguesa, Porto, 4202-401 Porto, Portugal; ^2^Escola Superior de Enfermagem do Porto, 4200-072 Porto, Portugal

####### **Correspondence:** Tânia Costa (tcosta@porto.ucp.pt) – Instituto de Ciências da Saúde, Universidade Católica Portuguesa, Porto, 4202-401 Porto, Portugal

**Background**

The inability of the National Health Service and sociodemographic changes have contributed to increase the number of elderly who are beneficiaries of informal care at home. However, the literature reveals association between exercising the role of caregiver and burden, which in turn is influenced by the health status of receptor. Objective: To assess the burden of family caregivers of dependent elderly in self-care and correlate the burden of family caregivers with health status of the receptor.

**Methods**

Cross-sectional analytical study. Were eligible 30 dyads of care composed by family caregivers and the dependent elderly in self-care. Collection of data was held by structured interviews and analysis by SPSS.

**Results**

Most of caregivers are female (77 %), in average with 74 years, married (73 %), with education below 4th year (76 %). Predominated the lower middle class (73 %). With regard to burden, 63 % had moderate, 30 % severe and 7 % extreme high. The Pearson coefficient revealed a statistically significant correlation between the burden caregivers and many variables of receptors like number of wounds (r = -0.499; p = 0.009); number of health complication (r = -0.645; p =0.003); drug complex (r = 0.326; p = 0.035); dependence in AVDS (r = 0.355; p = 0.021); AIVDs (r = -0.349; p = 0.025); pressure ulcer risk (r = 0.292; p = 0.041) and cognitive impairment (r = 0.578; p = 0.011).

**Conclusions**

Family caregivers with higher levels of burden care for receptors with less number of wounds, less number of health complications, lower risk of pressure ulcers, lower dependence on Activities of Daily Living/Instrumental Activities of Daily Living (ADLs/IADLs) more complex drug regimens and cognitive impairment. These data revealed the need to elaborate assessment tools and customized/efficient intervention programs.

**Keywords**

Family Caregiver, Burden, Elderly, Health Status

#### O193 Phenomena sensitive to nursing care in day centre

##### Armando Almeida^1,2^, Tânia Costa^1,2^, Gabriel Baffour^1,2^

###### ^1^Instituto de Ciências da Saúde, Universidade Católica Portuguesa, Porto, 4202-401 Porto, Portugal; ^2^Escola Superior de Enfermagem do Porto, 4200-072 Porto, Portugal

####### **Correspondence:** Armando Almeida (enfarmandoalmeida@gmail.com) – Instituto de Ciências da Saúde, Universidade Católica Portuguesa, Porto, 4202-401 Porto, Portugal

**Background**

Day centres receive the elderly with self-care problems but usually do not have nursing care. For this reason, it is unknown what are the needs/problems potentially sensitive to nursing care. Objective: To conduct a multidimensional health diagnosis, centred in the elderly that attend day centres.

**Methods**

Quantitative study, observational, cross-sectional which focused on 68 users, with more than 65 years, from three day centres of Oporto.

**Results**

It is a very old population (mean 80 years), predominantly female (86.8 %), low educated and socially isolated. Dominated by situations of suspected cognitive impairment (51.5 %) dependence in instrumental activities of daily living (91.2 %), basic activities (41.2 %) and mobility (17.6 %). However, the phenomena considered most relevant for nursing practice were: the presence of pain (66.9 %); the risk of falling associated with loss of balance (64.7 %), direct relationship with the number of falls in six months (n = 59); the fear of falling associated with physical inactivity (70.6 %); polypharmacy (75.0 %) and the inability to manage the drug regimen associated with loss of cognitive ability and mobility, as well as vulnerability of social and financial character.

**Conclusions**

We observed a set of phenomena sensitive to nursing care that should be seen as opportunities to develop intervention projects and contractually new health indicators targeted to these social facilities.

**Keywords**

Elderly, day centre, nursing indicators

#### O194 Frailty: what do the elderly think?

##### Zaida Azeredo, Carlos Laranjeira, Magda Guerra, Ana P. Barbeiro

###### Research in Education and Community Intervention, Escola Superior de Saúde Jean Piaget, 4405-678 Gulpilhares, Vila Nova de Gaia, Portugal

####### **Correspondence:** Zaida Azeredo (zaida.azeredo@gmail.com) – Research in Education and Community Intervention, Escola Superior de Saúde Jean Piaget, 4405-678 Gulpilhares, Vila Nova de Gaia, Portugal

**Background**

With the demographic ageing and the increase of very old persons, a new concept appears with greater importance in elderly health and family care: frailty. Among old persons (with or without pathology) ageing may decrease their biologic and psychological reserves to cope with stress, or some events. The elderly frail need more social and health support because the danger of institutionalization or death is high. Objective: To study how the elderly understand frailty.

**Methods**

This is an exploratory study in a sample of 113 persons of 65 years or over of both sexes who want to and were able to participate. We interviewed to data saturation.

**Results**

The majority (93.8 %) are 80 years old or over. 65.5 % were female, 54.0 % considered themselves a frail person. Age, mobility constrains, health problems, loss of abilities, loneliness, weakness, less ability to cope with stress, grief and losses are mentioned as situations involved in the definition of frailty.

**Conclusions**

Fifty-four (54 %) of the elderly felt they were frail. The reasons why they think a person is frail are in agreement with the literature concept of frailty and expressed in some scales to measure frailty. It will be interesting to go on with this study and survey the elderly living in other situations (e.g. at home).

**Keywords**

Elderly, frailty, prevention

#### O195 The therapeutic self-care as a nursing-sensitive outcome: A correlational study

##### Regina Ferreira

###### Escola Superior de Saúde, Instituto Politécnico de Santarém, Santarém, 2005-075 Santarém, Portugal

**Background**

The theory of self-care is a reference to the practice of nursing care. A change in the person's health status compromises their ability to care for themselves. Self-care is a nursing-sensitive outcome. Objective: To assess therapeutic self-care as a nursing-sensitive outcome.

**Methods**

Correlational study; simple random sample with application of a therapeutic self-care scale.

**Results**

Two hundred and eighty-eight (288) clients participated in the study, 53.8 % females, aged between 19 and 95 years old and only 18.4 % having higher education. By factor analysis we obtained two factors (managing medications and symptoms and performing daily life activities) with 52.8 % of the total variance explained and with good internal consistency (α = .87). The younger (F = 5.27; sp ≤ .006) and more highly educated (F = 4.91; p ≤ .000) evaluate "managing medications and symptoms" more positively. Those who have the 2nd cycle or higher education have higher average values (F = 3.88; p ≤ .002) for "activities of daily living". The lower number of hours required for client care corresponds to highest average value in both dimensions obtained (F = 2.41; p ≤ .02 and F = 3.32; p ≤ .001). Using multiple linear regression, it was found that the centrality of care explains 19.0 % of the variance of therapeutic self-care (R2 = .19; F = 58.20; gl = 248; p = .000).

**Conclusions**

Therapeutic self-care is a nursing-sensitive outcome. The results of the socio-demographic variables seem to be related to quality of care, from which the clients focus emerges and which, we believe, not only depends on the hours of nursing care. Centred care predicts therapeutic self-care.

**Keywords**

Therapeutic self-care, nursing-sensitive outcomes, centred care

#### O196 Phonetic-phonological acquisition for the European Portuguese from 18 months to 6 years and 12 months

##### Sara Lopes, Liliana Nunes, Ana Mendes

###### School of Health Sciences, Polytechnic Institute of Setúbal, 2910-761 Setúbal, Portugal

####### **Correspondence:** Sara Lopes (sara.rf.lopes@gmail.com) – School of Health Sciences, Polytechnic Institute of Setúbal, 2910-761 Setúbal, Portugal

**Background**

Phonetic-phonological acquisition varies according to the spoken language. For the European Portuguese (EP), the phonemes’ majority is acquired before the age of 3 years (Y) and the phonological processes decrease progressively throughout the development until 5-6 years. Objectives: The present study defined the phoneme acquisition, the phonological processes used and determined if there were differences between genders and among ages, from 18 months (M) to 6Y 12 M, divided into 11 age groups, of 6 M each.

**Methods**

Nine hundred three (963) children participated, 469 males and 494 females, EP speakers, ages between 18 M and 6Y 12 M, with kindergarten attendance and Portugal’s mainland or islands residency. They named 67 images of Phonetic-Phonological Test (Teste Fonético-Fonológico, TFF-ALPE). Productions were transcribed using the International Phonetic Alphabet. Frequency of occurrence of phonemes and phonological processes were calculated with Microsoft Office Excel 2013.

**Results and conclusions**

Acquisition order of the phonemes was: oral vowels < nasal vowels < oral occlusive < nasal occlusive < fricative < liquid and the frequency of occurrence of phonemes increased with age growth. The frequency of phonological processes decreased with age growth. Syllabic structure processes were more frequent than substitution processes. Inferential statistical results will be presented at the Convention of Health.

**Keywords**

Phonetic-phonological acquisition, European Portuguese, 18 months to 6 years and 12 months

#### O197 Quality of life of patients undergoing liver transplant surgery

##### Julian Martins^1^, Dulcineia Schneider^2^, Marcia Kretzer^1^, Flávio Magajewski^1^

###### ^1^Universidade do Sul de Santa Catarina, Palhoça, Santa Catarina, 88137-270 Brasil; ^2^Universidade Federal de Santa Catarina, Florianópolis – Santa Catarina, 88040-900 Brasil

####### **Correspondence:** Julian Martins (julianrodrigues1@hotmail.com) – Universidade do Sul de Santa Catarina, Palhoça, Santa Catarina, 88137-270 Brasil

**Background**

Liver transplantation is indicated worldwide for the treatment of liver diseases in advanced stages and most successful solid organ transplantation with one-year survival 83-91 %. After transplantation, an improvement in the patient's perception regarding their health and quality of life (QOL) occurs. Objective: To evaluate the quality of life of patients undergoing liver transplant surgery at a specialized hospital in Santa Catarina in 2009, compared with patients who are on the waiting list for the same type of transplant.

**Methods**

Case-control study with 38 cases and 54 controls. Data collected on Santa Catarina SC transplants, Brazil, in December 2013. Excluded patients who underwent liver transplantation intervivo; Double liver-kidney transplant; transplanted or queued with positive HIV test. Specific instrument Liver Disease Quality of Life (LDQOL). Analyses in SPSS 20.0, Chi-square test and Student's t-test with p ≤ 0.05, Odds ratios and Confidence Interval 95 %. Approved by the Research Ethics Committee of University of Southern Santa Catarina No 384.389.

**Results**

Frequency in male cases (89.5 %), age above 50 years (65.8 %) and retired (72.4 %). Statistical significance (p ≤ 0.05) in almost all domains of quality of life in transplant: liver symptoms, sleep, memory, sexual function, hope, social interaction, concentration, concern about the disease, effects of the disease and hepatic stigmata. **Conclusions**

The quality of life in patients undergoing liver transplantation was high in all aspects compared to patients on the waiting list for transplantation.

**Keywords**

Liver Transplantation, Quality of Life, Transplantation

#### O198 Professional competences in health: views of older people from different European Countries

##### Célia Soares, António Marques

###### School of Health Sciences, Polytechnic Institute of Setúbal, 2910-761 Setúbal, Portugal

####### **Correspondence:** Célia Soares (celia.soares@ess.ips.pt) – School of Health Sciences, Polytechnic Institute of Setúbal, 2910-761 Setúbal, Portugal

**Background**

Innovative expertise related to the promotion of positive ageing is crucial to deal with the rapid growth of the ageing population in modern societies. Higher education plays an essential role in the development of new models of training in health professions. Nevertheless, the perspectives and the diversity of needs of the recipients of care can enrich the (re)conceptualization of education on ageing and health. Objectives: The main aim of this study was to explore older people’s views on the competences of health professionals working with elderly populations in different European countries.

**Methods**

A qualitative study was conducted in Austria, Finland, Lithuania, Portugal, Turkey, and the U.K. A convenience sample of 16 participants was selected in each country (N = 96). Semi-structured interviews were used for data collection according to a common interview script. Thematic analysis was performed throughout the 96 interviews.

**Results**

Older people’s views are structured around four major themes. These emphasize individuality and dignity of service users, as well as their personal and social background; effective communication and relational orientation of professionals are also highlighted. Technical expertise, training in gerontology and ageing, vocation, commitment and ethical recommendations are also part of participant’s discourses.

**Conclusions**

Despite the cultural differences that can be found in European countries, interpersonal sensitivity, and person-centred care represent a relevant aspect for older people when they focus on the competences of health professionals. Hence, some changes may in fact be necessary to improve in European health education and training within the near future.

**Keywords**

Ageing, Professional Competences, Europe

#### O199 Life satisfaction of working adults due to the number of hours of weekly exercise

##### Marco Batista^1,2^, Ruth J. Castuera^3^, Helena Mesquita^1^, António Faustino^1^, Jorge Santos^1^, Samuel Honório^1,2^

###### ^1^Instituto Politécnico de Castelo Branco, 6000-767 Castelo Branco, Portugal; ^2^Research in Education and Community Intervention, Instituto Piaget, 3515-776 Galifonge, Viseu, Portugal; ^3^Universidad de Extremadura, 10003 Cáceres, España

####### **Correspondence:** Marco Batista (marco.batista@ipcb.pt) – Instituto Politécnico de Castelo Branco, 6000-767 Castelo Branco, Portugal

**Background**

The wellness construct, as measured by satisfaction with life is understood as a trial process in which individuals generally estimate the quality of their respective lives based on their own criteria. For the assessment of satisfaction with life, it is inseparable from the evaluation of positive and negative feelings of each individual. Objective: The objective was to analyse the differences and correlations depending on the number of hours of exercise and practice context, satisfaction with life and feelings of active Portuguese adults.

**Methods**

The study sample consisted of 560 Portuguese adults of both genders, aged between 30 and 64 years. The Satisfaction with Life Scale (SWLS) and the Scale of Positive and Negative Affect were used. The parametric tests one-way ANOVA and the coefficient of linear Pearson correlation were used.

**Results**

The results revealed a growing trend in the levels of life satisfaction and positive feelings with the increased volume of hours of weekly exercise, yielding an inverse relationship in the face of negative feelings. There were also significant differences when compared between groups in levels of life satisfaction and positive and negative feelings.

**Conclusions**

Apparently the increase in the volume of hours of weekly exercise promotes an increase in life satisfaction and positive feelings, with benefits for individuals who practise exercise in both group and individual contexts.

**Keywords**

Satisfaction with life, Exercise, Wellbeing

#### O200 Therapeutic itinerary of women with breast cancer in Santa Maria City/RS

##### Betina P. Vizzotto^1^, Leticia Frigo^1^, Hedioneia F. Pivetta^2^

###### ^1^Centro Universitário Franciscano, Santa Maria, Rio Grande do Sul, 97010-032 Brasil; ^2^Universidade Federal de Santa Maria, Santa Maria, Rio Grande do Sul, 97105-900, Brasil

####### **Correspondence:** Betina P. Vizzotto (be_vizzotto@hotmail.com) – Centro Universitário Franciscano, Santa Maria, Rio Grande do Sul, 97010-032 Brasil

The incidence of new cases of breast cancer increases every year in Brazil, as well as mortality rates that are higher possibly because this disease is diagnosed in advanced stages. The objective of this study was to investigate the therapeutic itinerary of women diagnosed with breast cancer in the city of Santa Maria- RS.

This is a retrospective cross-sectional documentary study in which medical records of women diagnosed with breast cancer were analysed.

Data collection was conducted from August 2013 to January 2015 in two public referral centres in the diagnosis and treatment for municipal breast cancer. Four hundred seventy-five (475) records were analysed, and of these 91 contained all the necessary information.

It was observed that the predominant therapeutic itinerary diagnosis was followed by surgery, chemotherapy and radiotherapy. The time between the procedures mostly followed the deadline established by the Ministry of Health and histological types of higher prevalence in the current study was invasive ductal and invasive lobular.

It was observed that patients’ therapeutic itinerary confirms the findings in the literature of better disease prognosis and the time elapsed between diagnosis and treatment initiation is in accordance with the public policy recommendations.

**Keywords**

Breast neoplasms, therapeutic itinerary, public health

#### O201 The breastfeeding prevalence at 4 months: Maternal experience as a determining factor

##### Dolores Sardo

###### Escola Superior de Enfermagem do Porto, 4200-072 Porto, Portugal

**Background**

The protection, promotion and support of breastfeeding (BF) is a public health priority, taking into account the benefits for the child, mother and society. BF prevalence depends on psychological, cultural and social factors of the mother [1]. Knowledge and the mother's previous experience is critical for the BF success. Objectives: To identify the prevalence of BF at 4 months and to evaluate the effect of the prior maternal experience in BF prevalence.

**Methods**

We performed a quantitative, descriptive and cross-sectional study in a Portuguese population. The sample was non-probabilistic, intentional (n = 286) of mothers who were married or with a partner (86.7 %) and average age was 31 years old. Data was collected with a self-report questionnaire at 4 months after delivery.

**Results**

The BF prevalence at 4 months is 72.7 %, but only 51.0 % do so exclusively. 20.3 % of mothers have previous experience of BF. Women with previous breastfeeding experience tend to breastfeed longer now, with statistically significant differences (U = 19.500, p = 0.000). However, the variables studied only previous experiences and BF duration was statistically significant (U = 19.500, p = 0.000).

**Conclusions**

Previous maternal experience positively influences breastfeeding prevalence at 4 months, although this is still low according to WHO guidelines [2]. As health professionals, we should be able to identify difficulties early and support lactating mothers, whatever their decision.

**References**

1. Pak-Gorstein S, Haq A, Graham E. Cultural influences on infant feeding practices. Pediatrics Revue. 2009; 30:11-21.

2. World Health Organization. Global Strategy on infant and young child feeding. WHO: 2002.

**Keywords**

Breastfeeding, prevalence, maternal experience

#### O202 The impact of the transition to parenthood in health and well-being

##### Cristina Martins^1^, Wilson Abreu^2^, Mª Céu Figueiredo^2^

###### ^1^Escola Superior de Enfermagem, Universidade do Minho, Braga, 4710-057 Braga, Portugal; ^2^Escola Superior de Enfermagem do Porto, 4200-072 Porto, Portugal

####### **Correspondence:** Cristina Martins (cmartins@ese.uminho.pt) – Escola Superior de Enfermagem, Universidade do Minho, Braga, 4710-057 Braga, Portugal

**Background**

Even though it is common, normative, predictable and usually desired, parenthood is one of the most dramatic developmental transitions in the family life cycle, likely to cause imbalance and vulnerability. Objective: This study aimed to explore the influences on the health and well-being of parents during the first 6 months of transition to the exercise of the parental role.

**Methods**

Grounded Theory. Data collection from semi-structured interviews (total of 60 interviews). Use of constant comparative method and theoretical sampling in the process of data collection and data analysis, undertaken in a simultaneous and recursive manner. Participation of five fathers and five mothers (couples).

**Results**

They describe the category of living on the edge of one’s capacities, which is composed by the subcategories of feeling exhaustion and weariness, perceiving exhaustion and weariness in the wife, releasing emotions and feeling less exhaustion and weariness, that explain the consequences of the discovery of the impact that the birth of the child has on the parents' lives, and upon realizing all the tasks and responsibilities of parenthood.

**Conclusions**

The adaptation to parenthood is not easy, linear and fast. It involves numerous situations that generate stress and emotional disturbance, tied to tiredness, sleep disturbance, work overload and readjustments of the dynamics of life, which affect the mother especially. The antenatal and postnatal preparation for this impact should, therefore, be encouraged and effective, as a focus of nursing intervention.

#### P96 Self-determined motivation and well-being in Portuguese active adults of both genders

##### Marco Batista^1^, Ruth Jimenez-Castuera^2^, João Petrica^1^, João Serrano^1^, Samuel Honório^1^, Rui Paulo^1^, Pedro Mendes^1^

###### ^1^Instituto Politécnico de Castelo Branco, 6000-767 Castelo Branco, Portugal; ^2^Universidad de Extremadura, 10003 Cáceres, España

####### **Correspondence:** Marco Batista (marco.batista@ipcb.pt) – Instituto Politécnico de Castelo Branco, 6000-767 Castelo Branco, Portugal

**Background**

The self-determination theory suggests that humans have several basic psychological needs that are innate, universal and essential to health and well-being, namely autonomy, competence and relation perception. The wellness construct, measured by satisfaction with life is understood as a judgment process in which individuals generally estimate the quality of their lives based on their own criteria. Objective: The objective was to analyse the differences by gender, of the self-determined and wellbeing levels of Portuguese active adults’ motivation.

**Methods**

The study sample consisted of 560 Portuguese active adults of both genders, aged between 30 and 64 years. The instruments used were the Behavioural Regulation in Sport Questionnaire (BRSQ), the Basic Psychological Needs Scale Exercise (BPNES) and also the Satisfaction with Life Scale (SWLS).

**Results**

The descriptive analysis revealed that female gender individuals had higher average levels of external regulation, identified regulation and integrated regulation, as well as autonomy, competence, relation perception and satisfaction with life. Men had higher average values of demotivation, introjected regulation and intrinsic motivation.

**Conclusions**

We did not identify significant gender differences in motivation dimensions. However, when comparing the dimensions of basic psychological needs and life satisfaction between genders, women showed significantly higher levels of autonomy perception, competence and relation perception, as well as well-being, when compared to men.

**Keywords**

Self-determination, exercise, well-being

#### P97 The geriatric care: ways and means of comforting

##### Patrícia Sousa^1^, Rita Marques^2^

###### ^1^Universidade Católica Portuguesa, Lisboa, 1649-023 Lisboa, Portugal; ^2^Hospital de Santa Maria, Centro Hospitalar Lisboa Norte, 1649-035 Lisboa, Portugal

####### **Correspondence:** Patrícia Sousa (patriciapontificesousa@gmail.com) – Universidade Católica Portuguesa, Lisboa, 1649-023 Lisboa, Portugal

**Background**

Thought needs to be given to ways that lead to understanding and practising geriatric care as well as identifying the actions of health professionals, requiring an understanding of the meaning/significance of care. Objective: to know the ways and means of giving comfort as perceived by the hospitalized elderly.

**Methods**

A descriptive study with a qualitative approach, guided by the ethnographic method. Semi-structured interviews were conducted with 20 elderly patients, audio-recorded and submitted to content analysis. The patients were deliberately chosen, having been admitted to a medical service of a hospital in Lisbon. There was participant observation in order to understand the situational experiences, based on previously structured scripts [1].

**Results**

The ways and means of giving comfort are contextualized within the person’s resilience and help to maintain and restore capabilities, allowing better support in moments of discomfort. Among the ways and means of giving comfort used by nurses, emerged aspects related to communication (touch, smiles, humour, silence/unconditional presence), information/clarification; relationship of empathy/complicity; respecting the decision/will of the individual; relief of discomfort through massage. This study also highlights particular moments of comfort such as family visits, the initial contact and hygiene and personal arrangements.

**Conclusions**

Geriatric care is based on the meeting/interaction between the actors under the influence of the context in which it arises. The purpose of the different ways and forms of seeking comfort is to facilitate/enhance comfort, relieve discomfort and/or invest in potential comfort.

**References**

1. Spradley JP. The participant observation. New York: Holt Rinehart and Winston; 1980.

**Keywords**

Geriatric Care, elderly patient, hospitalization, ways and forms of comforting

#### P98 The influence of relative age, subcutaneous adiposity and physical growth on Castelo Branco under-15 soccer players 2015

##### António Faustino, Paulo Silveira, João Serrano, Rui Paulo, Pedro Mendes, Samuel Honório

###### Escola Superior de Educação, Instituto Politécnico de Castelo Branco, 6000-767 Castelo Branco, Portugal

####### **Correspondence:** António Faustino (a.faustino@ipcb.pt) – Escola Superior de Educação, Instituto Politécnico de Castelo Branco, 6000-767 Castelo Branco, Portugal

The aim of our study was to analyse the influence of Relative Age (RA), Subcutaneous Adiposity and Physical Growth of Castelo Branco soccer players. To characterize the sample of 49 soccer players, born in 2000 and 2001 [ARC Bairro do Valongo (11), CD Alcains (16) CD Castelo Branco (11) and SB Castelo Branco (11)], dates of birth were framed in quarters.

Data collected were: (i) Relative Age; (ii) Morphological variables - Anthropometric [H- Height, W-Weight, WS- Wingspan, Skinfolds (TRISK- triceps, SBSSK- subscapularis, SILSK- suprailiac, CSK- crural and GMLSK- geminal), Muscular Perimeters (forearm, brachial with contraction, crural and geminal) Bone Diameters (BCHD- bi-condyle humeral, BCFD- bi-condyle femoral, BAD- biacromial, BCD- bicristal, WD- Wrist and AD- Ankle)] and Somatic (BMI).

For the study we used descriptive statistics and the Pearson correlation coefficient.

Results show: (I) Correlation for H, W, WS and BMI is negative, the higher the year/quarter of birth, the lower these measures are; (II) Correlations between RA and Skinfolds are weak and not significant for TRISK, SBSSK, SISKL and CSK. But the GMLSK is superior to the previous one and though not strong, is statistically significant; (III) Correlation between RA and Perimeters are moderate and all statistically significant. The younger the player, the smaller is every one of the perimeters; (IV) Correlations between RA and Bone Diameters are weak and not significant for BCUD, BCFD, BAD and BCD, but with the WD and AD it is greater than the previous ones and, although not strong, it is statistically significant.

**Keywords**

Relative age, Subcutaneous Adiposity, Physical Growth, Under-15 soccer players

#### P99 Data for the diagnostic process focused on self-care – managing medication regime: An integrative literature review

##### Catarina Oliveira^1^, Fernanda Bastos^2^, Inês Cruz^2^

###### ^1^Unidade de Transplante Hepático e Pancreático, Centro Hospitalar do Porto, 4099-001 Porto, Portugal; ^2^Escola Superior de Enfermagem do Porto, 4200-072 Porto, Portugal

####### **Correspondence:** Catarina Oliveira (olive.enf@gmail.com) – Unidade de Transplante Hepático e Pancreático, Centro Hospitalar do Porto, 4099-001 Porto, Portugal

**Background**

Nursing diagnostic activity involves obtaining a set of data that is necessary to interpret, arrange, systematize, and assign meaning to constitute useful information. This approach is important in the beginning of the clinical decision-making process. It is important for nurses to better understand the complexity of the phenomenon “managing therapeutic regimen”, particularly, the medication regime. Objective: Identify the necessary data for description of Nursing Diagnoses about "Self -Care: managing the medication regime”.

**Methods**

Integrative literature review using the EBSCOhost databases with the following keywords: “Medication” and “Therapeutic” from a previous research universe. Inclusion criteria: language; full text; publication date from 2007-01-01 to 2012-12-31; descriptors in at least one of the parties (TI), (AB), (MM), (MH), (SU); peer reviewed articles.

**Results**

Of the 408 articles analysed it was possible to identify data that are essential to the diagnostic process. After a content analysis process, this data assumed different statuses: data that are manifestations, i.e. data which arise as a premise and are indispensable to diagnosis identification (e.g.: doesn´t take medication) and data that are competing factors for the diagnosis. These data are those which often have a causal relationship with the diagnosis (e.g. person, illness and medication regime characteristics).

We also find that many of the dates are more related with adherence than with managing therapeutic regime.

**Conclusions**

Since data collection is the first step of the nursing diagnosis process, nurses must improve their knowledge based on scientific evidence, to better identify the needs in nursing care in this area.

**Keywords**

Diagnostic process, self-care – managing medication regime, integrative literature review

#### P100 Art therapy as mental health promotion for children

##### Cláudia K. Rodriguez^1^, Márcia R. Kretzer^2^, Nazaré O. Nazário^2^

###### ^1^Universidade Estadual Paulista 'Júlio de Mesquita Filho", 01049-010, São Paulo, Brazil; ^2^Universidade do Sul de Santa Catarina, Palhoça, Santa Catarina, 88137-270 Brazil

####### **Correspondence:** Nazaré O. Nazário (nazare.nazario@unisul.br) – Universidade do Sul de Santa Catarina, Palhoça, Santa Catarina, 88137-270 Brazil

**Background**

The contemporary world instigated by new technologies is resulting in changes in our perceptions and thus in society. This fact predisposes rising fears and mental conflicts, especially in children. Carl G. and Maurice Merleau-Ponty’s grounded work reflects this today, with its concerns about being in its totality. The psychological, cultural and social make art a therapeutic experience, where the child can deal with their social and personal skills. Objective: To allow therapeutic experiences in art therapy that promotes mental health of children and can be used as a tool for social rehabilitation.

**Methods**

A phenomenological qualitative study, applied to a group of 27 children 09-10 years in the State public school from São Paulo, Brazil, in 2013. Registration and understanding through camera recordings of interviews and reports. The artistic techniques and materials were varied in accordance with the engagement established with each child. Ethical aspects were respected.

**Results**

Relations with perception and space were key concepts to art therapeutic activities collaborating in their cognitive and affective connection. The expansion of the concept of expressive therapies using techniques and materials collaborated with the externalization of being symbolic. The process developed led to union and group trust, helping the children to share their fears, anxieties and stored feelings and position themselves positively in regard to their conflicts.

**Conclusions**

Therapeutic art activities experienced by the children enabled the significance of the symbols preventing and promoting mental health.

**Keywords**

Art therapy, mental health, children, cultural

#### P101 Chemical characterization of fungal chitosan for industrial applications

##### Pedro Cruz^1^, Daniela C. Vaz^1,2^, Rui B. Ruben^3^, Francisco Avelelas^4^, Susana Silva^4^, Mª Jorge Campos^4^

###### ^1^Coimbra Chemistry Centre, Chemistry Department, University of Coimbra, 3004-535 Coimbra, Portugal; ^2^Health Research Unit & School of Health Sciences, Polytechnic institute of Leiria, 2411-901 Leiria, Portugal; ^3^Escola Superior de Tecnologia e Gestão, Instituto Politécnico de Leiria, 2411-901 Leiria, Portugal; ^4^MARE, Instituto Politécnico de Leiria, Peniche, 2520–641 Peniche, Portugal

####### **Correspondence:** Daniela C. Vaz (dvaz@ci.uc.pt) – Coimbra Chemistry Centre, Chemistry Department, University of Coimbra, 3004-535 Coimbra, Portugal

Chitosan biofilms have been object of study and of application to several industrial and research fields, such as to winemaking, food science, tissue engineering, drug delivery and clinical rehabilitation, among others. Chitosan can be obtained by deacetylation of chitin, naturally present in the exoskeletons of crustaceans, invertebrates, insects and cell walls of some fungi. The use of chitosan from different sources presents not only important advantages in terms of extraction and purification of this polysaccharide, but also in terms of the different chemical and biomechanical properties exhibited by chitosan biofilms.

In order to evaluate the properties of fungi-extracted chitosan and to compare them with the features presented by chitosan obtained from crustacean sources, we have analysed the chemical properties presented by chitosan extracted from two fungi, namely, from *Absidia coerulea* and *Cunninghamella* sp., grown in different culture media (YM and PDB). Chitosan solutions were analysed by refractometry, circular dichroism (CD), UV-visible spectroscopy and solution ^1^H NMR, while chitosan biofilms were analysed by differential scanning calorimetry (DSC) and optical microscopy.

Fungal chitosan presents exclusive chemical and physical properties, in what concerns the degree of deacetylation of the polymeric chains, viscosity, thermal resistance and structural conformation in solution. Features that are also influenced by the composition of the growth media used for cell culture, and that can be used industrially as advantages.

**Keywords**

Chitosan, fungi, biofilms, chemical characterization

#### P102 The impact of caring older people at home

##### Maria Almeida^1^, Liliana Gonçalves^2^, Lígia Antunes^3^

###### ^1^Escola Superior de Enfermagem de Coimbra, 3046-851 Coimbra, Portugal; ^2^Hospital Arcebispo João Crisóstomo, Cantanhede, 3060 Cantanhede, Portugal; ^3^Hospital José Luciano de Castro, Anadia, 3780-226 Anadia, Portugal

####### **Correspondence:** Maria Almeida (mlurdes@esenfc.pt) – Escola Superior de Enfermagem de Coimbra, 3046-851 Coimbra, Portugal

**Background**

The progressive aging of the population and the families’ increasing difficulty in being available to provide care are creating a new reality: older people as caregivers of dependent older people. The increase in the number of these caregivers, the consequences for their own health and the importance of their role have turned informal care into a highly relevant area for nursing, particularly regarding family education in home care provision. Objective: This study aims at identifying the reasons why informal caregivers undertake the provision of care to dependent older people at home, as well as the impact of care provision on informal caregivers.

**Methods**

A qualitative design was used, combined with phenomenographic research processes. The study was conducted in the district of Coimbra with nine informal elderly caregivers of dependent older people. Data were collected through semi-structured interviews and observation. Ethical aspects were followed and formal authorizations were obtained.

**Results**

In most cases, home care is provided by women in the 71-80 age group. The following dimensions emerged from interview analysis: motivation to care for the family member and caring task: perceived needs. Caregivers highlighted the physical, social, economic, and emotional burden. However, they also pointed out positive aspects, such as personal satisfaction, sense of duty, and family and social recognition.

**Conclusions**

This study reveals that women are the ones who usually provide care to the dependent elderly. The caring task has a negative impact on the life of the caregivers due to its overburden.

**Keywords**

Elderly, dependent elderly, informal caregiver, caring task

#### P103 Development of the first pressure ulcer in an inpatient setting: Focus on patients’ characteristics

##### Pedro Sardo^1^, Jenifer Guedes^2^, João Simões^1^, Paulo Machado^3^, Elsa Melo^1^

###### ^1^School of Health Sciences, University of Aveiro, Aveiro, 3810-193 Aveiro, Portugal; ^2^Hospital de Aveiro, Centro Hospitalar do Baixo Vouga E.P.E., Aveiro, 3814-501 Aveiro, Portugal; ^3^Escola Superior de Enfermagem do Porto, Porto, 4200-072 Porto, Portugal

####### **Correspondence:** Pedro Sardo (pedro.sardo@ua.pt) – School of Health Sciences, University of Aveiro, Aveiro, 3810-193 Aveiro, Portugal

**Background**

Pressure ulcers continue to be a challenge and represent an indicator of healthcare quality. Objectives: To analyse the incidence of the participants who developed their first pressure ulcer during the length of stay in association with their demographic and clinical characteristics.

**Methods**

A retrospective cohort analysis of electronic health record databases of adult patients, admitted to medical and surgical areas in a Portuguese hospital during 2012, without any pressure ulcer on admission. The development of the first pressure ulcer was associated with age, gender, type of admission, specialty units, first Braden Scale score, length of inpatient stay and ICD-9 diagnosis.

**Results**

From a sample of 6,572 participants, 153 (2.3 %) developed their first pressure ulcer and the odds were significantly higher for those admitted through the emergency service [OR = 3.64 (95 % CI, 2.20-6.05)], with Braden Scale scores ≤ 16 [OR = 4.82 (95 % CI, 3.45-6.73)] and/or with length of inpatient stay longer than 20 days [OR = 8.35 (95 % CI, 5.92-11.78)]. Older participants, in the age group 70-79 [OR = 9.87 (95 % CI, 1.35-71.84)] and ≥ 80 [OR = 17.75 (95 % CI, 2.46-128.19)] also had higher odds. Participants with “traumatisms and fractures” and “infectious diseases” had a higher incidence (6.6 % and 5.3 % respectively).

**Conclusions**

The participants that developed the first pressure ulcer during the length of stay differ from those that remain without pressure ulcers in the variables “admission”, “Braden Scale score”, “length of inpatient stay” and “age”. There were no differences between “gender” and “specialty units”. The ICD-9 diagnosis could be an important risk factor for pressure ulcer development.

**Keywords**

Incidence, International Classification of Diseases, Nursing, Nursing Assessment, Pressure Ulcer, Risk Assessment

#### P104 Association between General Self-efficacy and Physical Activity among Adolescents

##### Susana Cardoso^1^, Osvaldo Santos^2^, Carla Nunes^1^, Isabel Loureiro^1^

###### ^1^Escola Nacional de Saúde Pública, Lisboa, 1600-560 Lisboa, Portugal; ^2^Instituto de Medicina Preventiva, Faculdade de Medicina de Lisboa, Universidade de Lisboa, 1649-028 Lisboa, Portugal

####### **Correspondence:** Susana Cardoso (suscardoso@yahoo.com.br) – Escola Nacional de Saúde Pública, Lisboa, 1600-560 Lisboa, Portugal

**Background**

Technological society often carries an increased sedentarism among adolescents. These can integrate exercise into their free time, with clear health benefits. Self-efficacy, first described by Bandura (1982) can be described as feelings of adequacy, efficiency and competence to tackle the problems and underlies the choices and effort spent in activities or accomplishments. Objective: to investigate associations between self-efficacy and physical activity in adolescents.

**Methods**

This is a cross-sectional survey, with data collected through self-administered IPAQ (International Physical Activity Questionnaire) and General Self-Efficacy Questionnaire (GSQ - General Self Efficacy Questionnaire, adapted by Ribeiro (2011)) [1]. Two schools participated in the survey (convenience sample). It was possible to distribute the students (n = 358, aged 14 to 18) into three groups: with sedentary behaviours, with a moderate physical activity and presenting intense physical activity. The scores obtained from the General Self-efficacy scale, allowed to evaluate this parameter, specifically in such areas as resistance to adversity, initiative and persistence and social effectiveness.

**Results**

A statistically significant difference in self-efficacy was found between the sedentary adolescents (GSQ: 38-101, mean 73.7) and adolescents with intense physical activity (GSQ: 33-105, mean 80.4). Adolescents with intense physical activity have a higher self-efficacy (ANOVA with Bonferroni correction = .02).

**Conclusions**

Higher self-efficacy may be associated with more physical activity. Health promoting strategies which promotes self-efficacy may be appropriate to boost up healthy habits.

**References**

1. Ribeiro JP. Adaptação de uma Escala de Avaliação da Auto-eficácia Geral. Porto, Universidade do Porto; 2011.

**Keywords**

physical activity, adolescents, self-efficacy

### The health safety culture

#### O203 Characterization of the habits of online acquisition of medicinal products in Portugal

##### Flávia Santos^1^, Gilberto Alves^1,2,3^

###### ^1^Faculty of Health Sciences, University of Beira Interior, Covilhã, 6200-506 Covilhã, Portugal; ^2^Health Sciences Research Centre, University of Beira Interior, Covilhã, 6200-506 Covilhã, Portugal; ^3^Centre for Neuroscience and Cell Biology, University of Coimbra, 3004-504 Coimbra, Portugal

####### **Correspondence:** Flávia Santos (flaviapasantos@sapo.pt) – Faculty of Health Sciences, University of Beira Interior, Covilhã, 6200-506 Covilhã, Portugal

**Background**

Technology has progressively changed many conventional processes from patient registration and monitoring to the acquisition of medicines. Indeed, the purchase of medical products online is now an emerging reality, including prescription medicines without restrictions. Although it may bring advantages (convenience, saving time and money), the acquisition of medical products online has also important risks (e.g. counterfeit medicines). Objective: This work intends to characterize the habits of online customers of medicines/medical supplements in Portugal.

**Methods**

A structured inquiry was applied to 872 people with questions regarding the knowledge and the practices of online medicinal products acquisition.

**Results**

The majority of respondents (75.5 %) knew about this possibility, but only 8.4 % had online purchasing activities. The respondents who never bought medications online pointed out reasons like preference for the community pharmacy and lack of trust in virtual pharmacies. Among those who have resorted to the online acquisition of medicinal products, the better prices and convenience were the main reasons cited. The slimming preparations and products for enhancement of physical abilities were the most frequently acquired. A particular concern is the fact that many respondents did not take any precaution when choosing a website (43.8 %).

**Conclusions**

According to these findings the Portuguese population has great confidence in healthcare professionals and still has a small adherence to the acquisition of medicinal products online. However, people need to be educated for this new paradigm since the Internet and social networking sites have an increasing impact in the marketing of medicinal products.

**Keywords**

Medicinal products, online purchase, public health, safety concerns

#### O204 Waiting room – A space for health education

##### Cláudia Soar, Teresa O. Marsi

###### Universidade do Vale do Paraíba, São José dos Campos, São Paulo, 12244-000, Brasil

####### **Correspondence:** Cláudia Soar (claudiasoar@hotmail.com) – Universidade do Vale do Paraíba, São José dos Campos, São Paulo, 12244-000, Brasil

In order to promote actions that encourage healthy eating practices, autonomy and self-care, nutrition/cooking workshops were developed in the outpatient waiting room of a college hospital. They included an oral presentation by the Nutrition students, using a flip chart as a visual aid. The benefits of various ingredients used in the recipes were discussed, as well as preparation methods and storage. The oral presentation was followed by a tasting of the food made that day. Fifty-four (54) workshops took place between 2014 and 2015, totalling 1,775 participants.

Several topics were approached, such as homemade energy supplements, how to use leguminous plants, healthy and flavoured olive oils, and organic produce.

The audience comprised patients and their family members, professors, students and employees. Participants indicated that the tasting was their preferred portion of the workshop (40 %), followed by the lectures (32 %) and the brochures (28 %). An average of 99 % of the participants considered that the information was relevant and stimulating for adopting healthier habits. All participants of all workshops, would recommend the workshops to other people.

Finally, 96 % of the participants declared they were highly satisfied with the activity. Students got to exchange experiences with the participants, and broaden their skills as educators. For the participants, it was a time for building healthier habits in a casual environment that favoured closer integration between all those involved.

**Keywords**

Health education, nutrition, waiting room

#### O205 Safey culture evaluation in hospitalized children

##### Ernestina Silva^1^, Dora Pedrosa^2^, Andrea Leça^3^, Daniel Silva^1^

###### ^1^Centro de Estudos em Educação, Tecnologias e Saúde, Escola Superior de Saúde, Instituto Politécnico de Viseu, 3504-510 Viseu, Portugal; ^2^Maternidade Daniel de Matos, Centro Hospitalar e Universitário de Coimbra, 3000-075 Coimbra, Portugal; ^3^ Hospital Dr. Nélio Mendonça, Região Autónoma da Madeira, Funchal, 9004-514 Funchal, Portugal

####### **Correspondence:** Ernestina Silva (ernestinabatoca@sapo.pt) – Centro de Estudos em Educação, Tecnologias e Saúde, Escola Superior de Saúde, Instituto Politécnico de Viseu, 3504-510 Viseu, Portugal

**Background**

Safety culture of an organization holds special meaning for patients, payers, managers and caregivers, being a phenomenon likely to be assessed in the various dimensions that integrates. Objective: To assess the safety culture of the paediatric patient perceived by health professionals.

**Methods**

Quantitative study of descriptive nature. A non-probabilistic sample was made up of 258 health professionals in functions in paediatric and neonatal services of Centro Hospitalar e Universitário de Coimbra and Hospital Dr. Nélio Mendonça, Região Autónoma da Madeira (RAM). The instrument was based on the Hospital Survey on Patient Safety Culture.

**Results**

Most professionals consider the patient safety “good” or “very good”. The dimension "teamwork" stood out positively, being the "patient safety support by management" and "response to the non-punitive error" considered problematic. More than 79 % of the professionals did not notify events/occurrences in the last 12 months.

**Conclusions**

These data suggest that it is necessary to invest in a safety culture that promotes voluntary reporting and non-punitive error and adverse events.

**Keywords**

Patient safety, Quality of health care, Medical errors, Child health services

#### O206 Sexual Self-awareness and Body Image

##### Ana Galvão^1^, Maria Gomes^1^, Paula Fernandes^2^, Ana Noné^1^

###### ^1^Escola Superior de Saúde, Instituto Politécnico de Bragança, 5300-253 Bragança, Portugal; ^2^Escola Superior de Tecnologia e Gestão, Instituto Politécnico de Bragança, 5300-253 Bragança, Portugal

####### **Correspondence:** Maria Gomes (mgomes16mgomes@gmail.com) – Escola Superior de Saúde, Instituto Politécnico de Bragança, 5300-253 Bragança, Portugal; Ana Galvão – Escola Superior de Saúde, Instituto Politécnico de Bragança, 5300-253 Bragança, Portugal

**Background**

Body image is understood as a multidimensional construction that broadly describes the internal representations of the body structure and the physical appearance of the individual in regards him/herself and others. Sexual self-awareness (ACS) can be understood as the evaluation that each of us makes of his/her feelings and actions related to his/her sexuality and sexual behaviour, describing what each of us thinks about sex and what we feel about behaviours. Objective: Identify dimensions of sexual self-awareness and body image in sexual satisfaction of the young.

**Methods**

Correlational descriptive study, a convenience sample of 84 students of a health school (29.8 % male, 20.2 % female), with ages between 19 and 34 years. As data collection instrument a poll through questionnaire, incorporating a Body Image Satisfaction and a Multidimensional Sexual Self-awareness scale, was used.

**Results**

The majority of the sample subjects indicate having a partner (59.5 %), perceive themselves as having the ideal weight (75.0 %), the ideal height (65.5 %) and a normal appearance (76.2 %). Globally a high and statistically significant ACS was observed (t-Student = 12.520; GL = 83; p-value < 0.001) and significant statistical differences exist between having/not having a partner and the ACS (Student t = 2,965; GL = 82; p-value = 0.004) showing that those who mention having a partner have a higher average ACS (average = 3.812; SD = 0.412) compared to those without (average = 3.496; SD = 0.563). No statistically significant correlations were observed between ACS and Body Image.

**Conclusions**

The Body Image Satisfaction and the Sexual Self-awareness prove positive.

**Keywords**

Sexual self-awareness, body image, young

#### O207 Perception of a Portuguese population regarding the acquisition and consumption of functional foods

##### Jaime Combadão, Cátia Ramalhete, Paulo Figueiredo, Patrícia Caeiro

###### Universidade New Atlântica, 2745-615 Barcarena, Portugal

####### **Correspondence:** Jaime Combadão (jcombadao@uatlantica.pt) – Universidade New Atlântica, 2745-615 Barcarena, Portugal

**Background**

Foods are no longer only recognized for their role in providing essential nutrients for normal body activity and function. Since from the late 1980s, emphasis has been credited to their content in substances capable to promote health and wellbeing. Those functional foods (FF) can benefit body functions beyond nutritional effects, in a way that improves the state of health and wellbeing. To improve interventions, it is advantageous to understand the consumer’s perception, namely at regional level, towards FF. Objectives: To assess consumers' attitudes regarding FF, as well as to characterise social, demographic and economic aspects associated with the consumption of FF, in a Portuguese population.

**Methods**

A survey was carried out, among a Portuguese population (N = 120), in order to collect information on their socio-economic profile and on the constructs knowledge, motivation and attitude related to FF. The constructs were modelled as latent variables, divided in its factors, with the statistical assessment done by factorial analysis (FA).

**Results**

The FA validated two different constructs, the attitudinal and the motivational. Those constructs were factorized as follows: one focused in foods, a second with the degree of knowledge in health benefits and the third concerning the fortification and supplementation of food.

**Conclusions**

These results can be used as a basis to improve communication efforts regarding consumption of FF as a means to improve health and wellbeing.

**Keywords**

Functional food, consumer knowledge, wellbeing, factorial analysis

#### O208 The work process in primary health care: evaluation in municipalities of southern Brazil

##### Karine C. Fontana, Josimari T. Lacerda, Patrícia O. Machado

###### Universidade Federal de Santa Catarina, Florianópolis, 88040-900 Brasil

####### **Correspondence:** Josimari T. Lacerda (jtelino@gmail.com) – Universidade Federal de Santa Catarina, Florianópolis, 88040-900 Brasil

The work process of primary care in Brazil must be based on completeness, longitude and coordination of care in health care networks. In Brazil, the municipal management guarantees structural and procedural conditions for the implementation of coordinated actions, based on these principles.

This research evaluated the work process of primary health care of 271 municipalities and 1,399 health care teams of a state in southern Brazil. A model consisting of 22 indicators was developed, aggregated into two dimensions. The Structural Conditions dimension covers Infrastructure, Personnel and Technological and Logistical Support so that the teams can carry out their activities. The Procedural Conditions analyses: Link and Territorialisation, organization of work and Coordination of Care. The model validated and agreed on by experts in the area allowed the identification of strengths and weaknesses subject to intervention. Data was used from the External Evaluation of the National Programme for Improving Access and Quality of Primary Health Care (known as PMAQ-AB), phase 2.

The evaluated universe represented 91.9 % of the municipalities of the Brazilian state. The study identified management advances in structural conditions, especially in permanent education and in the technological and specialized supply. About the procedural conditions, barriers were observed on the continuity of care and work organization with a highlight on: popular participation, intersectorial and shared actions, monitoring and accountability of the user. Efforts must be made on awareness and training of professionals in the exercise of their duties guided by the principles of Primary Health Care.

**Keywords**

Primary Health Care, Health Evaluation, Process Assessment (Health Care)

#### O209 Exploration and evaluation of potential probiotic lactic acid bacteria isolated from Amazon buffalo milk

##### Raphaelle Borges^1^, Flávio Barbosa^2^, Dayse Sá^1^

###### ^1^Universidade Federal do Amapá, Macapá - Amapá, 68903-419, Brasil; ^2^Universidade Federal do Sergipe, São Cristóvão - Sergipe, 49100-000, Brasil

####### **Correspondence:** Raphaelle Borges (raphaellebio@yahoo.com.br) – Universidade Federal do Amapá, Macapá - Amapá, 68903-419, Brasil

**Background**

Human intestinal tract presents important non-pathogenic microorganisms relevant for health. Dysregulation of this microbiota can cause diseases and poor nutritional status. Probiotics are used for intestinal regulation and disease prevention, either as supplements or as food components. Objectives: The purposes of this study were to isolate, select and characterize the lactobacillus present in the milk of Amazonian buffalo. This aimed at the production and evaluation of the probiotic potential of this product, in order to produce a Probiotic food in the future.

**Methods**

Samples of milk from two female buffalos were used in this study, in Macapá-AP, Brazil. Lactobacillus were isolated in Merck Research laboratories, and were characterized by breathing tests, Gram and Catalase tests, and identified by Polymerase chain reaction (PCR). They were submitted to stockpiling simulation, to viability, curves of growth, sensibility to antimicrobials, cellular surface adhesion tests and antagonism in vitro.

**Results**

Based on the samples of buffalo milk, 21 microorganisms of six species of lactobacillus were isolated: *Lactobacillus acidophilus* (33.33 %), *Lactobacillus ruminis* (23.80 %), *Lactobacillus reuteri* (14.28 %), *Lactobacillus johnsonii* (9.53 %), *Lactobacillus mucosae* (9.53 %) and *Lactobacillus. salivarius* (9.53 %). All species showed satisfactory results in terms of growth curves and sensibility tests. In the adhesion tests, stockpiling simulation and antagonism, the presence of *L. acidophilus* was more pronounced when compared to other species.

**Conclusions**

*L. acidophilus* is considered a probiotic microorganism and is widely used in dairy products industry. This fact supports the possibility of producing a Probiotic food, made from the milk of Amazonian buffalo, in the near future.

**Keywords**

Lactobacillus, probiotic foods, buffaloes

#### O210 Road safety for children: Using children’s observation, as a passenger

##### Germana Brunhoso^1^, Graça Aparício^2^, Amâncio Carvalho^3^

###### ^1^Centro Hospitalar de Trás –os-Montes e Alto Douro, Vila Real, 5000-508 Vila Real, Portugal; ^2^Centro de estudos em educação, tecnologias e saúde, Escola Superior de Saúde, Instituto Politécnico de Viseu, 3504-510 Viseu, Portugal; ^3^Escola Superior de Enfermagem de Vila Real, Universidade de Trás –os-Montes e Alto Douro, Vila Real, 5000-232 Lordelo, Vila Real, Portugal

####### **Correspondence:** Graça Aparício (gaparicio5@hotmail.com) – Centro de estudos em educação, tecnologias e saúde, Escola Superior de Saúde, Instituto Politécnico de Viseu, 3504-510 Viseu, Portugal

**Background**

Road accidents are a leading cause of death of Portuguese children. The correct use of restraint systems for car transportation can contribute to a reduction of up to 90 % of injuries in the case of traffic accidents. Objective: To analyse whether sociodemographic characteristics and the driver behaviour influences the protection of the child, as a passenger.

**Methods**

Observational and cross-sectional study achieved in a sample of 119 drivers and 152 school children. We made up a STOP operation in partnership with police officers, resorting to a record of socio demographic characterization and a check-list to observe the children's carriage conditions to, and from school in a surrounding area of a school centre in a Portuguese city.

**Results**

The transport was done in 92.8 % of the cases by mothers who mostly assumed to have safety road knowledge (66.4 %). The observation of the children's transport conditions revealed that 70.4 % used restraint system for children, 27.6 % seat belt and that 2.0 % were traveling loosened in the car. With adequate protection were traveling 51.3 % of children. The inferential analysis showed that the mother, drivers aged < = 40 years, with intellectual and scientific occupations and higher education, influences significantly the proper protection of children in road transport.

**Conclusions**

The use of restraint road systems for children is high, but the intention of protection proved to be higher than the proper protection, highlighting the need to keep the nursing counselling in this priority area of child safety promotion, especially among the most disadvantaged Portuguese families.

**Keywords**

Road safety, children as passengers, children restraint systems

#### O211 Perception and application of quality-by-design by the Pharmaceutical industry in Portugal

##### Ana P. Garcia^1^, Paula O. Fernandes^2^, Adriana Santos^1^

###### ^1^Health Sciences Research Centre, Faculty of Health Sciences, University of Beira Interior, Covilhã, 6201-001, Portugal; ^2^Unidade de Investigação Aplicada em Gestão, Instituto Politécnico de Bragança, Bragança, 5300-253 Bragança, Portugal & Research Unit in Business Sciences, University of Beira Interior, Covilhã, 6201-001 Covilhã, Portugal

####### **Correspondence:** Ana P. Garcia (acatarinagarcia@gmail.com) – Health Sciences Research Centre, Faculty of Health Sciences, University of Beira Interior, Covilhã, 6201-001 Covilhã, Portugal

**Background**

A Quality-by-Design (QbD) approach is mandatory in generics development for the USA and encouraged elsewhere. How pharmaceutical companies in Portugal are integrating it is unknown. Objective: The aim of this study was to assess the Portuguese pharmaceutical industries’ perception and experience with QbD.

**Methods**

A structured survey was validated by three specialists and sent to 27 pharmaceutical companies producing active substances or medicines (exception of medicinal gases) in Portugal between 16/12/2013 and 1/06/2014.

**Results**

Twelve companies answered the survey. Eleven have implemented QbD, which is considered of greater importance among the industries having R&D activities (p-value = 0.024). Implementation has increased in recent years, and 36 % used it already in a systematic and continuous way. The most used QbD tool was “Quality Risk Assessment and Quality Risk Management” (27 %), and the most frequent scopes of implementation were “*development and improvement of analytical methods*” and “*process improvements*” (28 %). 32 % of the companies indicated “*greater robustness in the final product quality and reduction of the risks of non-compliance and costs*” as benefit of QbD and the main obstacle towards QbD implementation was “*inability/difficulty of allocating time and qualified staff*” (43 %).

**Conclusions**

In Portugal there are companies with long experience in QbD and its implementation is increasing. An important obstacle to QbD implementation is the requirement of skilled staff, therefore it is essential that the Portuguese academy keeps pace with the professional demands and provides students the skills needed for working with the concept and tools of QbD.

**Keywords**

Quality-by-design, survey, pharmaceutical industry

#### O212 Oral health among Portuguese children and adolescents: a public health issue

##### Nélio Veiga^1,2^, Carina Brás^1^, Inês Carvalho^1^, Joana Batalha^1^, Margarida Glória^1^, Filipa Bexiga^1,2^, Inês Coelho^3^, Odete Amaral^4^, Carlos Pereira^4^

###### ^1^Departamento de Ciências da Saúde, Universidade Católica Portuguesa, Viseu, 3504-505 Viseu, Portugal; ^2^Centro de Investigação Interdisciplinar em Saúde, Universidade Católica Portuguesa, 3504-505 Viseu, Portugal; ^3^Unidade de Saúde Familiar Grão Vasco, Viseu, 3500-177 Viseu Portugal; ^4^Centro de estudos em educação, tecnologias e saúde, Instituto Politécnico de Viseu, 3504 - 510 Viseu, Portugal

####### **Correspondence:** Nélio Veiga (nelioveiga@gmail.com) – Departamento de Ciências da Saúde, Universidade Católica Portuguesa, Viseu, 3504-505 Viseu, Portugal

**Background**

Oral hygiene habits such as tooth brushing, dental flossing and dental appointments are important factors in determining good oral health. Objectives: To determine the prevalence of dental caries and assess oral health behaviours among a sample of Portuguese children and adolescents.

**Methods**

A cross-sectional study was conducted with a sample of 1,575 children and adolescents (50.3 % female) aged 2 to 18 years old from Viseu, Portugal. Data collection was performed by applying a questionnaire about oral health behaviours (answered by parents and adolescents) and through an intraoral observation for determination of the decayed, missing and filled permanent teeth index (DMFT) and deciduous teeth index (dmft).

**Results**

In this study, we obtained a DMFT index of 1.80 ± 2.47 with a decayed component of 1.26 ± 1.94 and a dmft index of 2.46 ± 3.23, with a decayed component of 2.01 ± 2.88. About 79.2 % of the sample revealed tooth brushing at least twice a day. With regard to dental appointments, 60.9 % referred to visiting the dentist in the last 12 months and 48.6 % to the consumption of sugary snacks between meals. A statistically significant association was found between the prevalence of dental caries and the amount of daily tooth brushing (p = 0.033) and consumption of sugary snacks between meals (p = 0.037).

**Conclusions**

The presence of oral diseases and inadequate oral health behaviours among children and adolescents is still an important public health issue in Portugal. Improvement has been verified in the last years; however, it continues to be essential to carry out primary preventive measures in oral healthcare among children and adolescents.

**Keywords**

Dental caries, oral health, children, adolescents, primary prevention, DMFT index

#### O213 Plant species as a medicinal resource in Igatu-Chapada Diamantina (Bahia, Brazil)

##### Cláudia Pinho^1†,3^, Nilson Paraíso^2†^, Ana I. Oliveira^1^, Cristóvão F. Lima^1^, Alberto P. Dias^1^

###### ^1^Escola Superior de Tecnologia da Saúde do Porto, Instituto Politécnico do Porto, Vila Nova de Gaia, 4400-330 Vila Nova de Gaia, Portugal; ^2^Universidade Federal da Bahia, Salvador da Bahia, 0170-115, Brazil; ^3^Centro de Investigação e de Tecnologias Agro-Ambientais e Biológicas, Universidade de Trás-os-Montes e Alto Douro, 5001-801 Vila Real, Portugal & Universidade do Minho, 4704-553 Braga, Portugal

####### **Correspondence:** Cláudia Pinho (clp@estsp.ipp.pt) – Escola Superior de Tecnologia da Saúde do Porto, Instituto Politécnico do Porto, Vila Nova de Gaia, 4400-330 Vila Nova de Gaia, Portugal

**Background**

Thnobotanical and ethnopharmacological studies are important for documenting traditional knowledge and have contributed to the discovery of many plant-derived drugs. Objectives: This study aims to document the medicinal plant use in the community of Igatu (Bahia, Brazil), as well as determining species with bioprospecting potential.

**Methods**

The ethnobotanical data were collected using semi-structured interviews. Recorded plants are listed along with their popular name, traditional use, part used, method of preparation and use value (UV). The informant consensus factor (ICF) and fidelity level (FL) of the plants were also determined.

**Results**

A total of 80 species belonging to 73 genera and 36 families were cited. Lamiaceae (27.8 %), Fabaceae (25.0 %), and Asteraceae (19.4 %) families are of greater importance. Leaves were found to be the most frequently used plant part, while infusion was the major form of preparation. The largest number of medicinal species was indicated for diseases of the digestive system. According to UV the most important species were Lantana camara L. (2.88) and Polygala multiceps Mart. ex A.W. Benn. (2.75). ICF values obtained reflect the agreement on the use of plants for the treatment of diseases of eye and adnexa, neoplasm-tumours, and endocrine, nutritional and metabolic diseases.

**Conclusions**

Plants with high UV, ICF and FL values are good candidates for further pharmacological and phytochemical investigations in the search for new drugs. A comparison of the results in plants with high UV with other studies showed that some of the traditional indications are supported by available data from scientific literature.

**Keywords**

Traditional knowledge, Ethnobotany, Ethnopharmacology, Chapada Diamantina

#### O214 Characterization of cognitive and functional performance in everyday tasks: Implications for health in institutionalised older adults

##### Pedro Silva^1,2^, Mário Espada^3,4^, Mário Marques^1,5^, Ana Pereira^1,5^

###### ^1^Universidade da Beira Interior, Covilhã, 6201-001 Covilhã, Portugal; ^2^Associação de Melhoramentos Pró Outeiro, Oliveira de Azeméis, 3720-514 Santiago de Riba-Ul, Portugal; ^3^School of Education, Polytechnic Institute of Setúbal, 2910-761 Setúbal, Portugal; ^4^Centro Interdisciplinar da Performance Humana, Faculdade de Motricidade Humana, Universidade de Lisboa, 1499-002 Cruz Quebrada, Portugal; ^5^Research Centre Sports Sciences, Health Sciences and Human Development, University of Trás-os-Montes e Alto Douro, Vila Real, 5001-801 Vila Real, Portugal

####### **Correspondence:** Ana Pereira (anapereiraphd@gmail.com) – Research Centre Sports Sciences, Health Sciences and Human Development, University of Trás-os-Montes e Alto Douro, Vila Real, 5001-801 Vila Real, Portugal

Functional and cognitive difficulties have been associated with early cognitive decline in older adults and increased risk for conversion to dementia in cognitive impairment, but our understanding of this decline has been limited by a dearth of objective methods. The primary purpose of this study was to investigate functional and cognitive performance in institutionalized older adults.

Ten subjects [(78.1 years (9.5), 158.4 cm (9.6), 68.2 kg (13.2), 27 BMI (3.9)] performed a battery of functional ability tests. These tests included functional measures such as the chair stand test (CTS), 6-minute walking test (6MWT) and get-up and go test (GUG). Cognitive impairment was evaluated by mini-mental state examination test (MMSE), activities of daily living using Barthel’s index (BI) and balance assessment with Tineti Test (TT). Results showed lower performance in all tests (p ≤ 0.05): the chair stand test (9.8 ± 3.0 repetitions), 6MWT (226.4 ± 114 meters), GUG (14.0 ± 10.9 seconds), MMSE (21.4 ± 5.9 total score) BI (95 ± 5.5 total score) and TT (21.9 ± 5.0 total score) at baseline of the study. Results suggest that the effectiveness of dual-task health intervention programmes seems to be the new strategic way to improve functional capacity and cognitive performance and can be useful in decreasing functional decline in institutionalized older adults. Activities targeting episodic memory may be most effective in addressing early functional impairment in older age.

**Keywords**

Cognition, dual-task, gait, muscular performance, institution, health promotion

#### O215 BMI and the perception of the importance given to sexuality in obese and overweight people

##### Ana Mª Pereira^1^, Mª Veiga-Branco^1^, Filomena Pereira^2^, Maria Ribeiro^1^

###### ^1^Escola Superior de Saúde de Bragança, Instituto Politécnico de Bragança, 5300-121 Bragança, Portugal; ^2^Faculdade de Medicina, Universidade de Lisboa, 1649-028 Lisboa, Portugal

####### **Correspondence:** Ana Mª Pereira (amgpereira@ipb.pt) – Escola Superior de Saúde de Bragança, Instituto Politécnico de Bragança, 5300-121 Bragança, Portugal

**Background**

Literature has been pointing towards obesity as the moderating variable of a depressive vicious cycle of self-esteem and self-image, with social isolation, anxiety and depression. This in turn drives people to channel sexual pleasure into the pleasure of food, thus aggravating their condition of obesity even more and consequently causing a major negative impact on the individual’s sexual life. Ojective: To assess the importance given to sexuality in obese and overweight individuals as well as assessing the existence of a correlation between these variables.

**Methods**

A quantitative exploratory study was conducted on 218 patients of both genders (68.3 % female and 31.7 % male) aged between 18 and 65. Data collection was carried out in several hospitals in the centre and north of the country. The data was collected by using the Index of Sexual Satisfaction (ISS).

**Results**

Among participants, 82.2 % were obese or overweight. Among the obese, 38.1 % registered a type I obesity; 16.4 % had type II obesity (severe); and 8.7 % had type III obesity (morbid). The obese revealed to be the ones who gave the most importance to sexuality, despite also being the ones who present the highest sexual dissatisfaction. Finally, the results show that there is a positive correlation, though weak, between sexual dissatisfaction and BMI.

**Conclusions**

Obese individuals are the ones who revealed the highest sexual dissatisfaction. Therefore, the cause of such dissatisfaction must be sought and valued as an issue related to obesity.

**Keywords**

Sexuality, obese, body mass index

#### O216 **Analysis and comparison of microbiological contaminations of two different composition pacifiers**

##### Vera Lima^1^, Ana I. Oliveira^1,2^, Cláudia Pinho^1,2^, Graça Cruz^1^, Rita F. Oliveira^1,2,3^, Luísa Barreiros^4^, Fernando Moreira^1,5^

###### ^1^Escola Superior de Tecnologia da Saúde do Porto, Instituto Politécnico do Porto, Vila Nova de Gaia, 4400-330 Vila Nova de Gaia, Portugal; ^2^Centro de Investigação em Saúde e Ambiente, Instituto Politécnico do Porto, Vila Nova de Gaia, 4400-330 Vila Nova de Gaia, Portugal; ^3^Secção Autónoma das Ciências da Saúde, Universidade de Aveiro, Aveiro, 3810-193 Aveiro, Portugal; ^4^UCBIO-REQUIMTE Rede de Química e Tecnologia^,^ 4051-401 Porto, Portugal & Departamento de Ciências Químicas, Faculdade de Farmácia, Universidade do Porto, Porto, 4050-313 Porto, Portugal; ^5^Department of Legal Medicine and Forensic Sciences, Faculty of Medicine, University of Porto, Porto, 4200-319 Porto, Portugal & Pharmaceutical Services, Centro Hospitalar de Vila Nova de Gaia/Espinho, EPE, Vila Nova de Gaia, 4400-129 Vila Nova de Gaia, Portugal

####### **Correspondence:** Fernando Moreira (ffm@estsp.ipp.pt) – Department of Legal Medicine and Forensic Sciences, Faculty of Medicine, University of Porto, Porto, 4200-319 Porto, Portugal & Pharmaceutical Services, Centro Hospitalar de Vila Nova de Gaia/Espinho, EPE, Vila Nova de Gaia, 4400-129 Vila Nova de Gaia, Portugal

Pacifiers are important devices during the development and growth of babies and young children, mainly owing to possible prevention of sudden instant death syndrome and provision of a comfort feeling towards stress and anxiety. However, permanent contact between pacifier and oral microflora leads to the creation of a biofilm in the pacifier’s surface. Besides, the contamination of pacifier’s outsides toddlers’ mouth cannot be disregarded by being dropped and immediately used by infants, enabling the entrance of pathogenic bacteria that might generate relevant and eventual systemic infections.

The main objectives of this study were to develop a method to quantitatively analyse the contamination of pacifiers used by infants and to compare the contamination susceptibility of two different materials: natural rubber and silicon. Ten samples were collected in a nursery in North of Portugal and properly kept in sterile bags during its transportation to the laboratory. Subsequently, a microbiological collection was performed from the pacifiers into Petri dishes previously filled with nutrient agar. Following incubation for 48 h, bacterial colonies were counted.

It was possible to confirm the presence of several colonies in the studied samples and, according to the obtained results, there was a tendency to a greater contamination in silicon than in rubber pacifiers.

The present study demonstrates that it’s important to define strategies to ensure the convenient cleaning and sterilization of pacifiers, owing to their massive contamination. Further studies, with larger number of samples, would be important to conclude about the most suitable composition of pacifiers, regarding the contamination prevention.

**Keywords**

Silicon, rubber, childcare, biofilm, measurement

#### O217 Experiences of couple relationships in the transition to retirement

##### Ana Camarneiro^1^, Mª Helena Loureiro^2^, Margarida Silva^1^

###### ^1^Escola Superior de Enfermagem de Coimbra, 3046-851 Coimbra, Portugal; ^2^University of Aveiro, Aveiro, 3810-193 Aveiro, Portugal

####### **Correspondence:** Ana Camarneiro (pcamarneiro@esenfc.pt) – Escola Superior de Enfermagem de Coimbra, 3046-851 Coimbra, Portugal

**Background**

Transition to retirement requires an effective adaptation to the new roles and functions in individuals and their partners. The couple lives special moments in this transition, maybe with a new marital dynamic. Objectives: To analyse the experiences of couple relationships in the process of adaptation to retirement and the strategies adopted to address it.

**Methods**

This is a qualitative and descriptive study. The sample consisted of 32 couples in which at least one spouse was retired for less than five years. The subjects were registered in health units of a Regional Health Administration of the central region of Portugal. Semi-structured interviews and thematic analysis using the NVivo10® program were used.

**Results**

The experiences of couple relationships in transition to retirement showed the following topics: Resources; Vulnerabilities; Expectations and future idealizations. Resources of conjugality and marital idealization in the future proved to be strategies of adaptation to retirement. Positive couple relationships are assumed as an important condition in the transition to retirement which is reflected in health and well-being. Couples have different vulnerabilities and marital resources. The spouse seems to be a key resource for the retired individual. Couples idealize happy times but also expect future difficulties.

**Conclusions**

The transition to retirement is a moment which, due to its marital and social importance, needs more investment in terms of psychological and physical health care.

**Keywords**

Couple, retirement, transition

#### O218 Preventive and corrective treatment of drug-induced calcium deficiency: an analysis in a community pharmacy setting

##### Catarina Duarte, Ângelo Jesus, Agostinho Cruz

###### Escola Superior de Tecnologia da Saúde do Porto, Instituto Politécnico do Porto, Vila Nova de Gaia, 4400-330 Vila Nova de Gaia, Portugal

####### **Correspondence:** Ângelo Jesus (acj@estsp.ipp.pt) – Escola Superior de Tecnologia da Saúde do Porto, Instituto Politécnico do Porto, Vila Nova de Gaia, 4400-330 Vila Nova de Gaia, Portugal

**Background**

Drug-nutrient interactions are not always valued in clinical practice. These interactions may result in drug-induced nutrient deficiency and therefore preventive and corrective treatments may be necessary. Pharmacy Technicians play an important role in identifying patients at risk. Objectives: To determine and characterise the values of the bone mineral density of patients using quantitative ultrasonic measurements of the calcaneus; to analyse the use of preventive or corrective treatment of drug-induced nutritional deficiencies and their consequences.

**Methods**

Observational analytic cross-sectional study performed in a Community Pharmacy in Porto with 103 individuals (82.5 % female) over the age of 40. Data collection was obtained through a structured questionnaire. Bone mineral density of the participants was quantitatively assessed by ultrasound of the calcaneus. Data were analysed using SPSS.

**Results**

Bone mineral density values were significantly lower in women (0.405 ± 1.07 g/cm^3^ vs. 0.510 ± 0.142 g/cm^3^). There was also a more pronounced decrease with age. Sixty-six per cent of the patients were being medicated with at least one drug that could induce calcium deficiency. Of the patients at risk, only 24.5 % take any sort of oral supplementation. About 30%of patients at risk were never screened or treated for osteoporosis.

**Conclusions**

The quantitative assessment of bone mineral density by ultrasound of the calcaneus in the pharmacy, allows the tracking of high-risk individuals. Pharmacy Technicians and other health professionals must provide a greater awareness of drug-induced nutritional deficiencies and adopt measures to prevent and avoid negative effects on patient health.

**Keywords**

Food-Drug Interactions, osteoporosis, calcium, community pharmacy services

#### O219 Profile of mood states in physically active elderly subjects: Is there a relation with health perception?

##### Maria Mota^1^, Sandra Novais^2^, Paulo Nogueira^2^, Ana Pereira^1,3^, Lara Carneiro^2^, Paulo V. João^1^

###### ^1^Research Centre for Sports Sciences, Health Sciences and Human Development, University of Trás-os-Montes e Alto Douro, Vila Real, 5001-801 Vila Real, Portugal; ^2^Department of Sport Sciences, Exercise and Health, University of Trás-os-Montes e Alto Douro, Vila Real, 5001-801 Vila Real, Portugal; ^3^Polytechnic Institute of Setúbal, 2910-761 Setúbal, Portugal

####### **Correspondence:** Maria Mota (mpmota@utad.pt) – Research Centre for Sports Sciences, Health Sciences and Human Development, University of Trás-os-Montes e Alto Douro, Vila Real, 5001-801 Vila Real, Portugal

Age-related psychological changes frequently express an increase in depressive symptoms and loss of vigour. A wide literature describes the benefits of regular physical exercise in mental health. However, concerning the elderly, the results are scarce. This study aimed to characterize the Profile of Mood States (POMS) in regular physically active elderly people. Moreover, we also intended to verify how the perception of health status was associated with POMS and whether there are differences between the sexes.

The survey sample was composed by 279 elderly people (237 females and 42 males), aged between 65 and 92 years, practitioners of physical exercise. The POMS questionnaire was used to measure positive (vigour) and negative (tension, depression, hostility, fatigue and confusion) variables. A case history questionnaire was used to measure the perception of their state of health. The t-test and Mann-Whitney test were used to compare variables by sex. A Spearman test was used to test association of variables. The significance level was set at 0.05.

According to POMS, the results revealed that the typical positive iceberg profile exists in this sample, with higher values obtained in vigour and lower values in negative variables. This profile was even better in men compared to women (p < 0.05). The perception of health was higher in men and was positively associated with the vigour scale of POMS and negatively associated with negative variables of POMS.

These results suggest that regular physical activity may contribute to maintaining elderly psychological well-being which proved to be related with health perception.

**Keywords**

Elderly, physical exercise, POMS, health

#### O220 (Un)Safety behaviour at work: the role of education towards a health and safety culture

##### Teresa Maneca Lima (tmaneca@gmail.com)

###### Centro de Estudos Sociais, Universidade de Coimbra, 3000-995 Coimbra, Portugal

Work accidents are a constant and fatal reality in all countries. The main causes are related to precarious working conditions and to workers’ inadequate behaviour. There is an obvious connection between behaviour and risk prevention at work. However, behaviour is not always the most important element in preventing labour accidents. Therefore, to promote a culture of health and safety among workers, it is fundamental to understand not only the role of education in fostering a conscientious behaviour prone to following safety procedures, but also the individual characteristics of each employee. In Portugal, work related accidents occur frequently among workers with low levels of education and training.

The objective of this paper is to explore the correlation between education and training programmes and the prevention strategy regarding labour risks in Portugal. Since the early 1990s, several national plans have been designed and implemented in order to assure the effective integration into school curricula of contents related to labour risks and health and safety at work. The National Programme for Education on Safety and Health at Work (PNESST) implemented in 2000 is the main example of the national strategy for education and training towards a culture of health and safety.

By examining the implementation of the PNESST and analysing the statistics of occupational accidents in Portugal, it is possible to determine that safety behaviour at work is related to the workers’ level of information and training in safety and health. Undoubtedly, this approach can contribute to reduce the number of labour accidents.

**Keywords**

work accidents, workers’ behaviours, education and training, health and safety culture

#### O221 Analysis of the entrepreneurial profile of students attending higher education in Portugal: the Carland Entrepreneurship Index application

##### Anabela Salgueiro-Oliveira, Marina Vaquinhas, Pedro Parreira, Rosa Melo, João Graveto, Amélia Castilho, José H. Gomes

###### Escola Superior de Enfermagem de Coimbra, 3046-851 Coimbra, Portugal

####### **Correspondence:** Anabela Salgueiro-Oliveira (anabela@esenfc.pt) – Escola Superior de Enfermagem de Coimbra, 3046-851 Coimbra, Portugal

**Background**

The societal changes have created new necessities in terms of health care and of professionals with diverse skills. The institutions of higher education should promote the development of an entrepreneurial profile in students which may boost the exploration of new opportunities. Objectives: To identify the entrepreneurial profile in the students in higher education and its relationship to some personal characteristics and training.

**Methods**

Correlational quantitative study, accomplished with 1,604 students from 18 institutions of polytechnic Institutes of Portugal. The collection of data occurred between July and November/2015. The Carland Entrepreneurship Index (CEI) entrepreneurial skills questionnaire was applied, along with the acquisition of socio-demographic variables of the students. Data was analysed with the SPSS 23.0. The study followed the ethical requirements.

**Results**

The (CEI) application, allowed us to note that 75.7 % of the students presented an entrepreneurial profile, 20.8 % a Micro entrepreneurial profile and 3.4 % a Macro entrepreneurial. Additionally, we verified that older students (r = 0.193, p < = 0.000), of the male gender (Female = 0.55; Male, M = 0.580 p < 0.000), that had already worked or would like to work for others, showed the greatest entrepreneurial potential (Yes M = 0.60; No 0.54; p < 0.000), along with those who participated in entrepreneurial contents during their training (Did not participate M = 0.53; Participated 0.58; p < 0.000).

**Conclusions**

The entrepreneurial profile is related with some social demographic characteristics of the students; however, the educational institutions may have a preponderant role in the development of that profile, which may contribute to a greater contribution in the wellbeing of the populations.

**Keywords**

entrepreneurial profile, students, higher education

#### O222 Evaluation of welfare and quality of life of pregnant working women regarding the age of the pregnant

##### María S. Medina^1^, Valeriana G. Blanco^2^

###### ^1^GyM Prevención, Burgos, 09007 Burgos, España; ^2^Universidad de Burgos, 09001 Burgos, España

####### **Correspondence:** María S. Medina (mariasierra3@hotmail.com) – GyM Prevención, Burgos, 09007 Burgos, España

The working situation of pregnant women has special connotations, both in the physical and psychological situation and this may influence their welfare. Aware of this, the present work has as objective to evaluate the perceived quality of life for pregnant working women at different times of pregnancy.

This work had a convenience sample of 165 pregnant women living in Burgos (Spain) and workers from different weeks of gestation and a wide range of ages.

The study has a cross-cutting nature and the data collection was carried out with the WHOQOL-BREF questionnaire and an ad hoc questionnaire to collect identification data and the weeks of gestation.

The criteria variables were: perceived quality of life, satisfaction with the health and quality of life in physical dimensions, health, psychological health, social relationships and environment. The predictor variables were weeks of pregnancy and age.

The results show that in general women are quite satisfied with their quality of life and health. The relationship between the variables that measure the quality of life and stage of pregnancy does not become significant although a trend to higher scores is observed in the overall assessment and the different dimensions in women who are in the category between 31 and 35 years.

**Keywords**

Pregnant, Quality of life, Working women

#### O223 Psychological wellbeing protection among unemployed and temporary workers: Uncovering effective community-based interventions with a Delphi panel

##### Osvaldo Santos^1,2^, Elisa Lopes^1,2^, Ana Virgolino^1,2^, Alexandra Dinis^3^, Sara Ambrósio^3^, Inês Almeida^3^, Tatiana Marques^3^, Mª João Heitor^3,4^

###### ^1^Instituto de Medicina Preventiva e Saúde Pública, Faculdade de Medicina, Universidade de Lisboa, 1649-028 Lisboa, Portugal; ^2^Instituto de Saúde Ambiental, Faculdade de Medicina, Universidade de Lisboa, 1649-028 Lisboa, Portugal; ^3^Faculdade de Medicina da Universidade de Lisboa, Lisboa, 1649-028 Lisboa, Portugal; ^4^Hospital Beatriz Ângelo, Loures, 2674-514 Loures, Portugal

####### **Correspondence:** Osvaldo Santos (osvaldorsantos@gmail.com) – Instituto de Medicina Preventiva e Saúde Pública, Faculdade de Medicina, Universidade de Lisboa, 1649-028 Lisboa, Portugal

**Background**

Economic crises have a deleterious impact on mental health. Unemployment and precarious work conditions pose an increased risk to psychological wellbeing. Effective community-based interventions need to be developed within the context of mental health equity-oriented programmes. Objectives: To identify effective short-term community-based interventions for psychological wellbeing protection among unemployed and temporary agency workers. More specifically, we aimed to identify which psychosocial competences, therapeutic reference-models and intervention formats should be used in these interventions.

**Methods**

We did a scoping review to map conceptually-driven interventions (through PubMed and SciELO). Results of this review were submitted to the opinion of a Delphi panel of experts, selected by intentional sampling, in the areas of: mental health promotion, public health, psychological intervention and human-resources management. Consensus criteria were set as 90 % of the scores per-item higher than 3 (5-point Likert scale) and average higher than 3.5 (variation coefficient lower than 0.35).

**Results**

The Delphi panel involved 46 experts (participation-rate at the end of 2 rounds: 90 %). Consensus was achieved for 5 intervention sessions with medium-size groups (15-20 people). Experts proposed different therapeutic-driven models (e.g., psychodynamic-inspired, systemic, cognitive-behavioural) as adequate for the indicated purposes. Cognitive-behavioural and psycho-educational strategies for mental health literacy promotion, psychosocial competences and emotional regulation were considered as maximizing the possibility of positive effects.

**Conclusions**

Irrespective of the conceptual framework, increased knowledge about mental health and basic psychosocial (empathy, active listening, assertiveness) and emotional-regulation competences emerged as necessary for wellbeing protection and to enhance resilience among vulnerable populations.

**Keywords**

Psychological wellbeing, Mental health promotion, Community intervention, Unemployment health, Delphi study

#### O224 Chilean population norms derived from the Health-related quality of life SF-6D

##### Miguel A. Garcia-Gordillo^1^, Daniel Collado-Mateo^1^, Pedro R. Olivares^2^, José A. Parraça^3^, José A. Sala^1^

###### ^1^University of Extremadura, Badajoz, 06071 Badajoz, España; ^2^Universidad Autonoma de Chile, Talca, 1670 Talca, Chile; ^3^Universidade de Évora, 7004-516 Évora, Portugal

####### **Correspondence:** Miguel A. Garcia-Gordillo (miguelgarciagordillo@gmail.com) – University of Extremadura, Badajoz, 06071 Badajoz, España

**Background**

The SF-6D classification provides utility values for health status. Utilities generated have a number of potentially valuable applications in economic evaluations and not only to ensure comparability between studies. Reference values can be useful to estimate the effect of interventions on patients’ HRQoL in the absence of control groups. Thus, the purpose would be to provide the SF-6D normative values in the Chilean population.

**Methods**

A cross-sectional study was conducted. A total of 5,293 people agreed to participate in the study. SF-6D utilities were derived from SF-12 questions.

**Results**

Mean SF-6D utility index for the whole sample was 0.74. It was better for men (0.78) than for women (0.71). The ceiling effect was much higher for men (11.16 %) than for women (5.31 %). Women were more likely to show problems in any dimension than men.

**Conclusions**

Chilean population norms for the SF-6D are shown in this paper to help in decision-making in health policies. Men reported a higher state of health than women in all sub-categories analysed. Likewise, men also reported higher scores than women in all dimensions of SF-6D in overall.

**Keywords**

Reference values, HRQoL, Utility, Health

#### O225 Motivation of college students toward Entrepreneurship: The influence of social and economic instability

##### Amélia Castilho, João Graveto, Pedro Parreira, Anabela Oliveira, José H. Gomes, Rosa Melo, Marina Vaquinhas

###### Escola Superior de Enfermagem de Coimbra, 3046-851 Coimbra, Portugal

####### **Correspondence:** Amélia Castilho (afilomena@esenfc.pt) – Escola Superior de Enfermagem de Coimbra, 3046-851 Coimbra, Portugal

**Background**

The concept of entrepreneurship is subdivided into entrepreneurship of opportunity and entrepreneurship of necessity, evidencing that labour-market instability has an important role in the decision to pursue it (Global Entrepreneurship Monitor, 2013). The recent world financial crisis led to social and employment instability in Portugal, with potential influence on the motivation to partake in business ventures. Objectives: To analyse the relationship between the Perception of Social and Economic Instability (PSEI) and select contextual and socio-demographic variables in polytechnic college students.

**Methods**

A correlational quantitative study made with 1,604 students from 18 different Portuguese Superior-Polytechnic institutions (mainland Portugal). A survey on business motivation from Parreira, Pereira and Brito (2011) was applied. The sample consists of female students (65.2 %), married students (11.1 %) and worker-students (19.7 %). The data were analysed through SPSS.

**Results**

Students with entrepreneur aspirations felt able to start a business and contrast with worker-students, who have less PSEI, respectively (Midea = 2.97, SD = 1.20; Mwithout_idea s = 3.21, SD = 1.12, t(1601) = 4.10, p < .000); (Mable_create = 3.01, SD = 1.16; Mnot_able_create = 3.19, SD = 1.17, t(1601) = 2.97, p < .003); (Mstudent = 3.12, SD = 1.17; Mstudent_worker = 2.92, SD = 1.17, t(1599) = 2.74 p < .006), relation between aspiration to the international market and PSEI (Minternacional = 2.95, SD = 1.24; Mnot_international = 3.11, SD = 1.15, t(1601) = 2.31, p < .021); women demonstrate more PSEI (Mmale = 2.93, SD = 1,13; Mfemale = 3.16, SD = 1.18, t(1601) = 3.17 p < .000); married students show less PSEI (Mmarried/joint = 2.91, SD = 1.17; Mother = 3.10, SD = 1.17, t(1601) = 2.11 p < .035).

**Conclusions**

Some sociodemographic variables are revealed to have influence on this process. Socioeconomic reality and the perception of instability in different contexts conditions the student’s perception as an entrepreneur.

**Keywords**

Entrepreneurship profile, Motivation of college students, Social and economic instability

#### O226 Use of aromatic and medicinal plants, drugs and herbal products in Bragança city

##### Mónica Cheio^1^, Agostinho Cruz^1^, Olívia R. Pereira^2^

###### ^1^Escola Superior de Tecnologia da Saúde, Instituto Politécnico do Porto, 4400-330 Vila Nova de Gaia, Portugal; ^2^Escola Superior de Saúde, Instituto Politécnico de Bragança, Bragança, 5300-121 Bragança, Portugal

####### **Correspondence:** Olívia R. Pereira (oliviapereira@ipb.pt) – Escola Superior de Saúde, Instituto Politécnico de Bragança, Bragança, 5300-121 Bragança, Portugal

Herbal therapy is characterized by the use of aromatic and medicinal plants (AMP) in different pharmaceutical forms for therapeutic purposes. The present study aims to characterize the use of AMP, drugs and herbal products in Bragança city.

A cross-sectional study was conducted through application of a questionnaire to 404 subjects of both gender and aged between 18 and 89 years.

AMP were therapeutically used by 53.7 % mainly due “*to be natural*” (43.9 %) while 33.8 % use drugs and/or herbal products mainly “*because it is good for health*” (53.5 %). The AMP most used were Cidreira (n = 149) and Camomila (n = 117) and concerning drugs and/or herbal products Valdispert® (n = 48) and Daflon® 500 (n = 41) were the most reported.

Overall, the reported uses of AMP, drugs and herbal products were correct, according to the reported in literature. The use of AMP is motivated by self-knowledge (55.4 %) while drugs and/or herbal products are used mostly by medical prescription (44.1 %). AMP were obtained by own cultivation (44.1 %) and drug and/or herbal products in pharmacies (89.0 %). Of all users, about 90 % did not combined these products with conventional drugs and it was identified just one potential occurrence of drug interactions related with the use of Hipericão. The occurrence of adverse effects was noted after the use of AMP Sene (11.8 %), Hipericão (9.1 %) and Ginkgo Biloba (8.3 %). The use of these products is a common practice among the residents of Bragança city, which use a wide diversity of AMP and plant-based products.

**Keywords**

Aromatic and medicinal plants, herbal drugs, herbal products

#### O227 Edible flowers as new novel foods concept for health promotion

##### Sara Pinto^1^, Adriana Oliveira^1^, M. Conceição Manso^1,2^, Carla Sousa^1^, Ana F. Vinha^1,2^

###### ^1^Unidade de Investigação UFP em Energia, Ambiente e Saúde & Centro de Estudos em Biomedicina, Fundação Fernando Pessoa, Porto, 4249-004 Porto, Portugal; ^2^REQUIMTE/LAQV, Departamento de Ciências Químicas, Faculdade de Farmácia, Universidade do Porto, 4050-313 Porto, Portugal

####### **Correspondence:** Ana F. Vinha (acvinha@ufp.edu.pt) – REQUIMTE/LAQV, Departamento de Ciências Químicas, Faculdade de Farmácia, Universidade do Porto, 4050-313 Porto, Portugal

Edible flowers are commonly used in human nutrition and their consumption has increased in the last years. In Europe, the most common application of flower petals in human nutrition is in the preparation of hot beverages (tisane or infusion), providing wellness due to the medicinal properties already recognized. Thus, it is paramount to know their nutritional composition as well as other functional and beneficial properties often related to their bioactive compounds and antioxidant properties.

Rose (*Rosa canina* L.), marigold (*Calendula officinalis* L.) and camellia (*Camellia* L.) were compared for their contents in total phenolics, flavonoids and carotenoids. Moreover, their antioxidant capacity was assessed.

In what concerns bioactive compounds and antioxidant activity (DPPH•), promising levels were obtained, showing significant differences among samples (p < 0.001). *C. officinalis* presented the highest levels of total phenolics and carotenoids contents (35.4 mg GAE/g, 15.6 mg/g, respectively). Herein, the best results for flavonoids content were obtained in rose petals (~95 mg ECE/g) potentially indicating the presence of high percentage of glycosylated polyphenolics which are readily soluble in water. Since the antioxidant activity is often correlated with the contents in total bioactive compounds the correlation coefficients among bioactive compounds and antioxidant activity were also studied.

The antioxidant activity was found to be positively and significantly highly correlated with total phenolics (rs = 0.917, p = 0.001) and carotenoids (rs = 0.900, p = 0.001). These findings might have practical applications regarding the enhancement of edible flowers, either for prompt consumption as well as to develop food supplements or pharmaceutics related products.

**Keywords**

Rose (Rosa canina L.), Marigold (Calendula officinalis L.), Camellia (Camellia L.), bioactive compounds, antioxidant activity (DPPH), health promotion

#### O228 The influence of leisure activities on the health and welfare of older people living in nursing homes

##### Mª Manuela Machado^1^, Margarida Vieira^2^

###### ^1^Escola Superior de Enfermagem, Universidade do Minho, Braga, 4710-057 Braga, Portugal; ^2^Universidade Católica Portuguesa – Porto, 4202-401 Porto, Portugal

####### **Correspondence:** Mª Manuela Machado (mmachado@ese.uminho.pt) – Escola Superior de Enfermagem, Universidade do Minho, Braga, 4710-057 Braga, Portugal

**Background**

Leisure activities, often left in the background throughout life, play an important role for older persons. They are activities that they like to accomplish, according to individual preferences that make it easier to maintain an active life, on a physical, mental and social level, improving their health. Objectives: I) Identify the leisure habits of older people living in nursing homes; II) Describe the nursing homes’ offer of leisure activities; III) Identify relationships between leisure activities and the seniors’ health condition.

**Methods**

A cross-sectional descriptive correlational study, in 12 nursing homes in the north of Portugal with a sample of 1,131 seniors. We performed a descriptive and inferential statistical data analysis using SPSS/PC for Windows, version 22.

**Results**

Participants were mostly female, widows, with an average age of 84 years. The most common leisure activity is watching TV. Impaired sight and illiteracy are the most frequent causes of elderly people’s non-adherence to leisure activities. Leisure activities: card/board games, crafts and reading are associated with better cognitive performance; sightseeing and reading are associated with greater independence in self-care; watching television is associated with greater compromise of bodily processes and greater dependence on self-care; performing manual work is associated with less depression; walking is associated with a lower compromise of bodily processes and lower risk of falling.

**Conclusions**

Leisure habits are important in maintaining the health of older people living in nursing homes.

**Keywords**

Elderly, Leisure habits, Nursing homes, Health status

#### O229 Risk of falling, fear of falling and functionality in community-dwelling older adults

##### Beatriz Fernandes, Teresa Tomás, Diogo Quirino

###### Escola Superior de Tecnologia da Saúde de Lisboa, Instituto Politécnico de Lisboa, 1549-020 Lisboa, Portugal

####### **Correspondence:** Beatriz Fernandes (beatriz.fernandes@estesl.ipl.pt) – Escola Superior de Tecnologia da Saúde de Lisboa, Instituto Politécnico de Lisboa, 1549-020 Lisboa, Portugal

**Background**

Ageing among Portuguese population is leading to an increase in the proportion of elderly people. Age-related changes are responsible for high levels of disability, balance problems and high risk of falls, Physiotherapy can identify elderly in risk of falling and provide strategies to prevent falls in this population contributing to maintain functionality. The purpose of this study was to characterise the risk of falling in a sample of community-dwelling older adults and investigate the associations between functionality and balance. Objective: To identify the risk of failing in community-dwelling older adults and ifs relations with fear of falling and functional capacity.

**Methods**

Cross-sectional study. Sixty-one (61) subjects participated in the study, 40 (65.6 %) females and 21 (34.4 %) males, aged 74 ± 7.5 years. Outcome measures were balance assessed with Berg Balance Scale (BBS); fear of falling with Falls Efficacy Scale (FES); functionality with Composite Physical Function (CPF).

**Results**

The results of our study showed that for BBS the median was 54, for FES was 96 and for CPF was 20 points. The risk of falling for this sample was 11 %. Positive associations were found between BBS and FES (R = 0.589; p = 0.00), CPF and BBS (R = 0.723; p = 0.00) and CPF and FES (R = 0.613; p = 0.00).

**Conclusions**

Risk of falling is present among the participants in our study. The positive associations between balance, confidence and functionality indicate that balance and confidence in performing activities of daily living are important for having high levels of function, suggesting that physiotherapy focused on balance training can contribute to enhance independence.

**Keywords**

Ageing, balance, risk of falling, function, physiotherapy

#### O230 Musculoskeletal pain and postural habits in children and teenage students

##### Gustavo Desouzart^1^, Rui Matos^2^, Magali Bordini^1^, Pedro Mouroço^3^

###### ^1^School of Health Sciences, Polytechnic institute of Leiria, 2411-901 Leiria, Portugal; ^2^Life Quality Research Centre, Polytechnic Institute of Santarém, 2001–904 Santarém, Portugal & Polytechnic Institute of Leiria, 2411-901 Leiria, Portugal; ^3^Centre for Rapid and Sustainable Product Development, Polytechnic Institute of Leiria, 2430-028 – Marinha Grande, Portugal

####### **Correspondence:** Gustavo Desouzart (gustavodesouzart@gmail.com) – School of Health Sciences, Polytechnic institute of Leiria, 2411-901 Leiria, Portugal

The study of the relationship between the musculoskeletal pain and postural habits in children and teenagers of the 2nd and 3rd cycles of basic education (107 male and 97 female) can establish criteria in health care in individuals during growth. A body part discomfort scale was adopted to check the references of pain level and localization. The results indicated ranged from 0 (no pain) and 5 (maximum pain), with an average of 2.40 ± 0.96 (moderate to severe pain), and longer time of the presence of pain (39.2 % of chronic pain) the greater the average intensity of pain (2.59 ± 0.87). 67.3 % (148) of the participants indicated musculoskeletal pain, 53.4 % reported pain in at least two different locations and 27 % referred to pain in three or more places. The 5th grade participants indicated chronic pain with 27.3 % as being 25 % (subacute) and 47.7 % (acute). In contrast, the amount of 9th grade participants who referred to pain in the body decreased (58.8 %), but the average pain (2.50 ± 0.89) and its duration increased (56.7 % of chronic pain). The average value of greater pain among women (2.57 ± 0.93) in both years of schooling was greater compared to males who had a mean value of pain 2.18 ± 0.97. This study aimed to present the 1st phase of a study developed in the school environment, in order to contribute to the deepening of the discussion on postural education. These results showed a prevalence of moderate/severe pain, the largest level of pain in females and in higher grade students.

**Trial registration**

Current Controlled Trials ANZCTR370295

**Keywords**

Musculoskeletal pain, Postural habits in students, Postural education

#### O231 What's different in Southern Europe? The question of citizens’ participation in health systems

##### Ana R. Matos, Mauro Serapioni

###### Centro de Estudos Sociais, Universidade de Coimbra, 3000-995 Coimbra, Portugal

####### **Correspondence:** Ana R. Matos (amatos@ces.uc.pt) – Centro de Estudos Sociais, Universidade de Coimbra, 3000-995 Coimbra, Portugal

Contemporary governance demands mutual interdependence of actors through participatory devices in order to competently address public problems and improve the quality of decisions. This debate is actively present within the health system where several international organizations have been exhorting national governments to develop public spaces within civil society as the proper locations for the improvement of their health systems. To date, several initiatives have been put forward and substantial resources have been invested in the design and implementation of citizens’ participation exercises in the health domain.

The main focus of this paper is civil society's involvement with health systems. It analyses the characteristics of the Greek, Italian, Portuguese, and Spanish health systems and the main steps in their reform processes. The most relevant initiatives of citizen participation are identified, highlighting their key features and potential, as well as the main critical issues raised.

The evolution of the national health services in the countries analysed shares a common history, as well as similar models for the provision of health services in the case of Italy, Portugal, and Spain.

With the exception of Greece, where participation is in an early stage, the participatory activities implemented in the other three countries revealed common features: I) a gap between the discourse on the importance of citizens' participation that permeated the reform processes and the practices implemented within health services; II) most of the experiments only take place at a local or regional level; III) the changes of governments have been negatively affecting the consolidation of participatory experiences.

**Keywords**

National Health Service, Citizen participation, Southern European countries

#### O232 Occupational stress in Portuguese police officers

##### Teresa Guimarães, Virgínia Fonseca, André Costa, João Ribeiro, João Lobato

###### Escola superior de tecnologia da saúde de Lisboa, 1990-096 Lisboa, Portugal

####### **Correspondence:** Teresa Guimarães (tguimaraes@estesl.ipl.pt) – Escola superior de tecnologia da saúde de Lisboa, 1990-096 Lisboa, Portugal

**Background**

Occupational stress occurs when individuals perceive work demands as exceeding their resources and abilities to cope with them, inducing a set of physical and emotional responses and affecting job satisfaction. Policing is often associated with high levels of occupational stress, resulting from stressors found in the work environment and in the individual’s family life. Objectives: To assess perceived occupational stress levels in police officers and to identify the most relevant stressors.

**Methods**

Fifteen (15) police officers (from a police station with 45 professionals), 93.3 % males, 24 - 46 years (M = 33.27; SD = 6.24), with an average of 9.07 years of experience (SD = 6.72), 66.7 % working more than 40 hours/week and 86.7 % doing shift work, completed the “Operational Police Stress Questionnaire” (PSQ-Op) and the “Organizational Police Stress Questionnaire” (PSQ-Org).

**Results**

Participants presented moderate stress levels for PSQ-Org (M = 3.45; SD = 1.18) and PSQ-Op (M = 3.62; SD = 1.01), with no significant differences between scores. No significant differences were found in stress levels regarding demographic or job variables. Main sources of stress are related to lack of resources, bureaucracy, working hours, shift work, the risk of being injured or witnessing traumatic events.

**Conclusions**

Our findings suggest a perception of moderate level of occupational stress in police officers, both at organizational and operational level. The fact that even moderate-intensity stressors affect individuals in a chronic way and may elicit physical and emotional responses, affecting health and well-being, strengthens the importance of interventions that enhance police officers’ ability to cope with stressors, helping to preserve job satisfaction and quality of life.

**Keywords**

Occupational stress, Police stress, Occupational health

#### O233 Is occupational therapy culturally relevant to promote mental health in Burkina Faso?

##### Inmaculada Z. Martin, Anita Björklund

###### Jönköping University Foundation, 551 11 Jönköping, Sweden

####### **Correspondence:** Inmaculada Z. Martin (zainma@ju.se) – Jönköping University Foundation, 551 11 Jönköping, Sweden

**Background**

The processes of globalization have a clear impact on occupational therapy. Consequently, there is a growing interest in aspects relating to health and culture in many societies. The development of an occupational therapy centre to promote mental health in Houndé, Burkina Faso (West Africa), must to go beyond the usual terminological questions, and to deepen and refine new guidelines in relation to the construction and consolidation of knowledge and also to the practical aspects in the country. Objectives: The main objective of this qualitative study was to analyse the occupational therapy professional culture and its impact on practice having the development of the occupational therapy centre to promote mental health in Burkina Faso as reference.

**Methods**

A qualitative design was used and it was based on a doubly reflexive ethnography, a heuristic three-dimensional model consisting of syntactic, pragmatic and semantic dimensions. Accordingly, the methodology is based on ten semi-structured interviews with professionals and local leaders who were involved in the pilot phase of this project.

**Results**

The results show that it’s necessary and urgent to develop consciousness and responsibility for the power relations that are implicit but also explicit in the theory and practice of occupational therapy.

**Conclusions**

This presentation will highlight the need of co-reflexion among the different actors involved in occupational therapy, because it is conclusive for the development of an intercultural perspective of occupational therapy that may be therapeutically effective. The ethnoccupation approach may be proposed as an alternative to the ethnocentric occupation intervention.

**Keywords**

Ethnoccupation approach, Reflexivity, Mental Health, Culture, Ethnography

#### P105 Pay-for-performance satisfaction and quality in primary care

##### Aida I. Tavares^1^, Pedro Ferreira^1,2^, Rui Passadouro^3^

###### ^1^Centro de Estudos e Investigação em Saúde, Faculdade de Economia, Universidade de Coimbra, 3004-512 Coimbra Portugal; ^2^Faculdade de Economia, Universidade de Coimbra, 3004-512 Coimbra Portugal; ^3^ACES Pinhal Litoral, ARS Centro, Coimbra, 3001-553 Coimbra, Portugal

####### **Correspondence:** Aida I. Tavares (aitavar@gmail.com) – Centro de Estudos e Investigação em Saúde, Faculdade de Economia, Universidade de Coimbra, 3004-512 Coimbra Portugal

**Background**

Pay-for-performance has been increasingly used in health systems. The reform of primary care in Portugal introduced health professionals to the possibility of pay-for-performance. Thus, two types of payment co-exist: the fixed salary, and pay-for-performance. This work sets out to test if the type of payment scheme determines payment satisfaction, to identify factors explaining the payment satisfaction and also to test if the type of payment scheme influences the self-perceived quality of health care provided in primary health care units.

**Methods**

A quantitative approach is adopted. Data from a survey is analysed by estimating tobit regressions.

**Results**

Payment satisfaction is explained by the type of incentives paid; being nurse decreases the payment satisfaction; the quality of health care unit has a positive relation with the type of incentives paid.

**Conclusions**

This is the first work of this kind for the Portuguese primary health care and it contributes to the ongoing discussion about pay-for-performance in the health sector.

**Keywords**

Incentives, pay-for-performance, primary care, tobit

#### P106 Economic development through life expectancy lenses

##### Sónia Morgado (soniamorgado2006@gmail.com)

###### Escola Superior de Desporto de Rio Maior, Instituto Politécnico de Santarém, 2040-413 Rio Maior, Portugal & Instituto Superior de Ciências Policiais e Segurança Interna, 1349-040 Lisboa, Portugal

**Background**

The paper aims to define the association between health development, measured by life expectancy, and economic development, focusing on Portugal, measured by Gross Domestic Product (GDP). We wish to test if causality between growth and health measured by life expectancy is possible, thus attesting to the adequacy of health policies, concerning the increase in quality and years of life of the population.

**Methods**

To perform the test a Vector Autoregressive Model Analysis – VAR model is used, and Granjer’s [1] causality test, applied to Portuguese time-series data from 6 decades. Alongside this, a univariate and bivariate analysis is applied.

**Results**

The positive evolution of GDP, over the years until 2010, before the beginning of economic crisis, at a positive rate of 14.0 %, has been followed by an increase in life expectancy. Even though the linear regression evidences a significant direct relation between variables (p-value = 0.002), the causality is not sustained.

**Conclusions**

Even though the results in the period show no interdependence of variables, health conditions are supported by growth [2], and health contributes to economic development [3-7]. Therefore, policies concerning productive aging should be considered.

**References**

1. Granjer, CWJ. Some Recent Developments in a Concept of Casuality. Journal of econometrics. 1988; 39:199-211.

2. Egger, G. Health “Iilth”, and Economic Growth: Medicine, Environment and Economics at the Crossroads. American Journal of Preventive Medicine. 2009; 37(1):78-83.

3. Ashraf, QH, Weil, DN. When Does Improving Health Raise GDP?. National Bureau of Economic Research. 2012; 14449:1-46.

4. Bloom, DE, Canning, D, Sevilla, J. The Effect of Health on Economic Growth: A production Function Approach. World Development. 2004; 32(1):1-13.

5. Deaton, AS, Paxson. CH. Aging and Inequality in Income and Health. The American Economic Review. 2005; 88:248-253.

6. Morgado, SMA. Does health promote economic growth? Portuguese case study: from dictatorship to full democracy. The European Journal of Health Economics. 2014; 15(6):591-598.

7. Morgado, SMA. Is Infant Mortality an Evidence of Economic Development. Revista de Saúde Pública. 2014; 48(n.esp):239.

**Keywords**

Life expectancy, economic growth, VAR model, Gross Domestic Product, Portugal

#### P107 What is the effectiveness of exercise on smoking cessation to prevent clinical complications of smoking?

##### Nuno Tavares, João Valente, Anabela C. Martins

###### Escola Superior de Tecnologia da Saúde de Coimbra, Instituto Politécnico de Coimbra, São Martinho do Bispo, 3046-854 Coimbra, Portugal

####### **Correspondence:** Nuno Tavares (ftnunotavares@gmail.com) – Escola Superior de Tecnologia da Saúde de Coimbra, Instituto Politécnico de Coimbra, São Martinho do Bispo, 3046-854 Coimbra, Portugal

**Background**

Cigarette smoking is one of the main risk factors for the occurrence of clinical complications such as cardiovascular diseases, cancer or hypertension. Currently the most common methods to quit smoking are behavioural counselling and pharmacological therapy, however their success rate remains quite low. Objectives: To analyse what is the effectiveness of exercise on smoking cessation and to discover what is the type of exercise and its characteristics that lead to better outcomes.

**Methods**

Pubmed, Cochrane Library, PEDro and ScienceDirect were consulted during the months of March and April 2015. The research was limited to guidelines, meta-analyses, systematic reviews and randomized controlled trials, written in English and published in scientific journals over the past 5 years. To be valid, the article should have as population smokers that performed an exercise program as intervention in comparison to its non-application. The wanted outcome was the percentage of subjects who stopped smoking on a long-term basis.

**Results**

It was considered valid to analysis eight randomized controlled trials that tested the effectiveness of aerobic exercise programs, muscular resistance and yoga.

**Conclusions**

Exercise tends to improve the percentage of long-term smoking cessation. The application of an aerobic exercise program of moderate intensity, 100 to 150 minutes per week, over 12 weeks of smoking abstinence is recommended.

**Keywords**

Exercise, Smoking cessation, Cigarrete smoking, Primary prevention

#### P108 A systematic review of the effects of yoga on mental health

##### Patrícia Araújo^1^, Rosina Fernandes^2^, Francisco Mendes^2^, Cátia Magalhães^2^, Emília Martins^2^

###### ^1^Universidade Católica Portuguesa – Porto, 4202-401 Porto, Portugal; ^2^Centro de Estudos em Educação, Tecnologias e Saúde, Escola Superior de Educação de Viseu, 3504-501 Viseu, Portugal

####### **Correspondence:** Rosina Fernandes (rosina@esev.ipv.pt) – Centro de Estudos em Educação, Tecnologias e Saúde, Escola Superior de Educação de Viseu, 3504-501 Viseu, Portugal

**Background**

Positive psychology is changing the way we research mental health in western society, bringing a focus to practices more than 5,000 years old in eastern culture, specifically with roots in India, such as yoga. Objectives: The aim of this paper is to explore documented effects of yoga on mental health, through a systematic review of literature (independently of year of publication, country or language).

**Methods**

The Scopus database was used and 29 publications were retrieved, 10 were excluded (such as theoretical articles, books and research that didn’t report mental health effects).

**Results**

Forty-eight (48) effects of yoga in mental health were found in the 19 publications analysed, which were systematized in three major categories. Personality and life style impacts (11), the first category, included decreases in neuroticism and psychoticism traits, changes in life style and increased quality of life, among others. Increased spatial memory and attention span, as well as decreased rumination negative affect are examples of the 15 effects included in the second category, named cognitive-perceptual impacts. Finally, 22 emotional effects (third category) were found, such as decreased anger levels, depression and anxiety. Overall, positive effects were predominant and only one effect can be interpreted as somehow negative (obsessive passion for yoga).

**Conclusions**

Since there are several types of yoga and diverse yoga techniques, further studies should analyse its specific impacts on mental health. Nevertheless, we hope this study allows a better comprehension of the benefits of yoga and, possibly, integration of yoga strategies in mental health interventions.

**Keywords**

Yoga, mental health, systematic review

#### P109 Healthy lifestyle: comparison between higher education students that lived until adult age in rural and urban environment

##### Pedro Mendes^1^, Rui Paulo^1,2^, António Faustino^1^, Helena Mesquita^1^, Samuel Honório^1,2^, Marco Batista^1,2^

###### ^1^Escola Superior de Educação, Instituto Politécnico de Castelo Branco, 6000-767 Castelo Branco, Portugal; ^2^Research, Education and Community Intervention, Instituto Piaget, 3515-776 Galifonge, Viseu, Portugal

####### **Correspondence:** Pedro Mendes (pedromendes@ipcb.pt) – Escola Superior de Educação, Instituto Politécnico de Castelo Branco, 6000-767 Castelo Branco, Portugal

Environment and lifestyle have been recognized as important determinants of health problems of populations of economically developed countries. In particular, these two factors seem to play a role on chronic degenerative diseases, sudden illness and accidents. The aim of our study is to compare lifestyles among higher education students that lived in rural and urban environments until adult age.

One hundred and thirty-two (132) students of both genders (85 males; 47 females) participated in the study. The mean age was of 21.81 years old (SD = 3.78). Subjects were divided in two groups (group 1 - those that lived until adult age in a rural environment and group 2 - the students that lived in an urban environment until adult age). To characterize the sample an adaptation of the Telama et al.’s [1] questionnaire was applied. The Fantastic Lifestyle Assessment Questionnaire - Portuguese version was applied to evaluate the lifestyle. For the study analysis, descriptive and inferential statistics were used. A Kolmogorov-Smirnov test was applied for normal distribution of data and a Mann-Whitney test for non-parametric statistics.

Results show a significant difference in only one dimension (insight) of the questionnaire (p ≤ 0.05). The remaining dimensions and the total scores of the questionnaire show no statistically significant differences. These results show that the type of environment (rural or urban) lived in until adult age doesn’t seem to reveal significant differences in a healthy lifestyle.

Overall the evaluated subjects presented results of “Good” and "Very Good" in the total score of the questionnaire revealing healthy lifestyles.

**References**

1. Telama R, Yang X, Laakso L,Viikari J. Physical activity in childhood and adolescence as predictor of physical activity in young adulthood. American Journal of Preventive Medicine.1997; 13(4): 317-323

**Keywords**

Lifestyle, environment, Fantastic Lifestyle Assessment

#### P110 Evaluation of the Mobile Emergency Care Service (SAMU) in Brazil

##### Josimari T. Lacerda^1^, Angela B. Ortiga^2^, Mª Cristina Calvo^1^, Sônia Natal^1^

###### ^1^Universidade Federal de Santa Catarina, Florianópolis, 88040-900 Brasil; ^2^Secretaria de estado da saúde de Santa Catarina, Florianópolis – Santa Catarina, 88015-130, Brasil

####### **Correspondence:** Josimari T. Lacerda (jtelino@gmail.com) – Universidade Federal de Santa Catarina, Florianópolis, 88040-900 Brasil

The Mobile Emergency Care Service (SAMU) in Brazil carries out pre-hospital care in seeking harm reduction, deaths and increased survival. It acts as an originator of network attention to the urgency and link between different services.

This is an evaluative research of mobile emergency care service with analysis of 80 central regulations and 87 municipalities of a Brazilian state that host units of basic and advanced support. It used a theoretical and logical model and evaluative matrix, validated through workshops with experts. The model included two dimensions. The emergency management dimension analyses infrastructure, financing, regulatory process and joins SAMU with the entire system. Attention is paid to urgent infrastructure analyses, diagnostic and therapeutic support and appropriateness of care. We used different collection strategies: document and literature review; interviews with key informants and direct observation.

The analysis allowed the identification of strengths, and also weaknesses that can be prevented. No region performed well, all dimensions were classified as regular and nearly half of the emergency management units require urgent attention. The SAMU partially organises the network which must evolve towards greater internal integration of its units and regulation of so-called fire and rescue services. It is recognized as a fast and quality service, but there was resistance to the acceptance once the patients had been referred.

Coordination mechanisms and internal and external communication need to be implemented. The quality of the units is a matter of concern. The model was considered feasible to contemplate municipal, regional and state assignments can be replicated in other contexts.

**Keywords**

Health Services Evaluation, Emergency Relief, Emergency Medical Services, Pre-hospital care.

#### P111 Bioactive compounds - antioxidant activity of tropical fruits

##### Marta Pereira (msofpereira@gmail.com)

###### Instituto Politécnico de Leiria; 2411-901 Leiria, Portugal

The consumption of fruits has become a way to improve our health. Bioactive compounds have an important role in antioxidant activity, since they are responsible for the protection of the cells by stabilizing or deactivating free-radicals. The evaluation and quantification of the bioactive compounds of dozens of tropical fruits allowed to stand out three fruits with a significant antioxidant activity: camu camu, acerola and açai. However, by-products also showed stunning amounts of bioactive compounds, which may be useful for developing of new products.

Further, the correlation identified phenolic compounds as major contributors for the antioxidant activity, although it was inconclusive about which specific phenolic compound had more influence.

**Keywords**

bioactive compounds, antioxidants, antioxidant activity, tropical fruits

#### P112 Use of non-pharmacological methods to relieve pain in labour

##### Manuela Ferreira^1^, Ana R. Prata^2^, Paula Nelas^1^, João Duarte^1^

###### ^1^Escola Superior de Saúde, Instituto Politécnico de Viseu, 3504-510 Viseu, Portugal; ^2^Hospital Pêro da Covilhã, Centro Hospitalar Cova da Beira EPE, 6200-251 Covilhã, Portugal

####### **Correspondence:** Manuela Ferreira (mmcferreira@gmail.com) – Escola Superior de Saúde, Instituto Politécnico de Viseu, 3504-510 Viseu, Portugal

**Background**

Labour pain is the result of complex interactions, similar to acute pain, but with specific characteristics of neurophysiological, obstetric, psychological and sociological nature involved in its threshold. In this sense, non-pharmacological methods can help the mother in relieving pain, reduce stress and anxiety levels and thus foster greater satisfaction. Objectives: Determining contextual variables of pregnancy and childbirth that interfere with the use of non-pharmacological methods to relieve pain.

**Methods**

A systematic literature review followed by a quantitative and simple descriptive, cross-sectional study, according to a non-probability analysis of 382 clinic records of post-partum mothers.

**Results**

The non-pharmacological methods used for pain relief during labour are more effective compared to not using any method, placebo or any other method. In a sample of 382 women with an average age of 30.95 years (±5.45 years), in 34.6 % of cases non-pharmacological methods for pain relief were applied in labour, highlighting breathing and relaxation (86.3 %). In some cases, although to a lesser extent, hydrotherapy was applied, isolated (6.9 %) or associated with breathing and relaxation (5.3 %) and hypnosis (0.8 %) and the association between breathing and massage (0.8 %).

**Conclusions**

According to the results obtained and on the basis of scientific evidence available, it should be noted that it is essential that non-pharmacological care relief in childbirth pain is exploited because it is safer and tends to carry fewer interventions and arguably a greater humanization of childbirth and safer motherhood.

**Keywords**

Labour, pain, complementary therapies,

#### P113 Mechanical safety of pacifiers sold in Portuguese pharmacies and childcare stores

##### Juliana Carneiro^1^, Ana I. Oliveira^1,2^, Cláudia Pinho^1,2^, Cristina Couto^1,3^, Rita F. Oliveira^1,2^, Fernando Moreira^1,4^

###### ^1^Escola Superior de Tecnologia da Saúde do Porto, Instituto Politécnico do Porto, Vila Nova de Gaia, 4400-330 Vila Nova de Gaia, Portugal; ^2^Centro de Investigação em Saúde e Ambiente, Instituto Politécnico do Porto, Vila Nova de Gaia, 4400-330 Vila Nova de Gaia, Portugal; ^3^Serviços farmacêuticos, Hospital Pedro Hispano, Unidade Local de Saúde de Matosinhos, EPE, 4454 509 Senhora da Hora, Portugal; ^4^Department of Legal Medicine and Forensic Sciences, Faculty of Medicine, University of Porto, 4200-319 Porto, Portugal & Pharmaceutical Services, Centro Hospitalar de Vila Nova de Gaia/Espinho, EPE, Vila Nova de Gaia, 4400-129 Vila Nova de Gaia, Portugal

####### **Correspondence:** Fernando Moreira (ffm@estsp.ipp.pt) – Department of Legal Medicine and Forensic Sciences, Faculty of Medicine, University of Porto, 4200-319 Porto, Portugal & Pharmaceutical Services, Centro Hospitalar de Vila Nova de Gaia/Espinho, EPE, Vila Nova de Gaia, 4400-129 Vila Nova de Gaia, Portugal

Potential dangers that arise from the use of pacifiers by children have been reported for the past few decades. The repeated cases of injuries and even fatalities possibly due to poorly designed pacifiers justified the elaboration of guidelines that ensured the mechanical safety of those devices in United States of America, Brazil and European Union.

This experimental study is the first one to describe the submission of a group of pacifiers sold in a European country to a group of tests and determinations that enable the evaluation of its mechanical safety, after EN1400 came into force. Adopted methods included determinations of nipple resistance, total size of the device, presence and size of ventilation holes, performance of a swallowing risk test and label analysis.

All 13 samples were bought in pharmacies and childcare stores in the north of Portugal and belonged to five different brands. The obtained results revealed that all analysed samples presented the minimum required ventilation holes with adequate sizes and correct placement. Also all samples proved to have nipples with adequate resistance to stretch and never exceeded the maximum permitted size. None of the samples went by the polytetrafluoroethylene (PTFE) device that mimicked the mouth of a child. It is quite clear that pacifiers sold in Portuguese pharmacies and childcare stores present high standards of mechanical security, probably owing to the implementation of EN1400. However, it would be important to perform similar tests on pacifiers sold in internet websites.

**Keywords**

Toddlers, EN1400, asphyxiation prevention

#### P114 The importance of prenatal consultation: Information to pregnant women given on a unit of primary care

##### Ana S. Maia^1^, Michelle T. Oliveira^1^, Anderson R. Sousa^1^, Paulo P. Ferreira^2^, Géssica M. Souza^1^, Lívia F. Almada^1^, Milena A. Conceição^1^, Eujcely C. Santiago^3^

###### ^1^Faculdade Nobre, Feira de Santana - Bahia, 44001-008, Brasil; ^2^Secretaria de Saúde do Estado da Bahia, Salvador - Bahia, 41745-900, Brasil; ^3^Hospital Estadual da Criança, Vila Valqueire, Rio de Janeiro, Brasil

####### **Correspondence:** Ana S. Maia (anamargarette@yahoo.com.br) – Faculdade Nobre, Feira de Santana - Bahia, 44001-008, Brasil

**Background**

The development of prenatal care requires multi-disciplinary actions focused on women's health. Humanization and quality care are crucial for the health of the mother-infant pair. The study aimed to analyse the knowledge of pregnant women about the importance of prenatal consultation

**Methods**

It is a field study, descriptive with a qualitative approach. It was held at the Family Health Unit of Jussara neighbourhood in the city of Feira de Santana, Bahia, Brazil. The research subjects were 18 pregnant women undergoing prenatal follow up in the unit. Semi-structured interviews were used for this study. Interviews were subjected to thematic content analysis.

**Results**

Most women reported that the importance of completing the prenatal follow up was mainly based on the detection of some diseases and also to care for the health of the child. The first prenatal consultation should take place in the first twelve weeks of pregnancy, as recommended by the Ministry of Health, and this period was respected by most of the pregnant women.

**Conclusions**

Prenatal influences directly the gestational process, and such assistance should be carried out properly, by professionals who have the knowledge that must be transmitted to pregnant women. The positive role of prenatal care during pregnancy was recognized by all respondents. However, it is important to stress out the need to carry out a quality prenatal care, because when conducted improperly, it can bring numerous health losses to both mother and child.

**Keywords**

Prenatal, pregnant women, query

#### P115 Influence of different backpack loading conditions on neck and lumbar muscles activity of elementary school children

##### Sandra Rodrigues, Gabriela Domingues, Irina Ferreira, Luís Faria, Adérito Seixas

###### Universidade Fernando Pessoa, Porto, 4249-004 Porto, Portugal

####### **Correspondence:** Sandra Rodrigues (sandrar@ufp.edu.pt) – Universidade Fernando Pessoa, Porto, 4249-004 Porto, Portugal

**Background**

The purpose of the present study is to analyse the influence of four backpack loading conditions (0, 10, 15 and 20 % of body weight) on the electromyographic activity of the neck and lumbar muscles of elementary school children.

**Methods**

Fourteen (14) school children participated in the present study, 5 males and 9 females, between the ages of 8 and 10. Bilateral sternocleidomastoid, rectus abdominis and erector spinae muscles were recorded using surface electromyography (SEMG), while tested for the effect of four randomized backpack loading conditions.

**Results**

The results showed an increase in the activity of the cervical erector spinae muscle, while erector lumbar spinae decreased in the presence of heavy loading. Rectus abdominis activity also increased and esternocleidomastoid muscle did not change its activity in the presence of loading. There seems to be an asymmetrical activation of the axial muscles, with preference towards the right side of the body, in a loading condition. A 20 % body weight backpack causes the most significant muscular changes; however, statistically significant differences were also found at 15 % body weight conditions.

**Conclusions**

Analysis of the present findings suggests the choice of 10 % body weight as the upper limit recommended for backpack load for schoolchildren.

ClinicalTrials.gov Identifier: NCT02725645.

**Keywords**

Surface electromyography, children, backpack

#### P116 Efficacy and safety of dry extract Hedera helix in the treatment of productive cough

##### Ana R. Costa^1^, Ângelo Jesus^1^, Américo Cardoso^1,2^, Alexandra Meireles^1,3^, Armanda Colaço^1,4^, Agostinho Cruz^1^

###### ^1^Escola Superior de Tecnologia da Saúde, Instituto Politécnico do Porto, Vila Nova de Gaia, 4400-330 Vila Nova de Gaia, Portugal; ^2^Instituto Português de Oncologia do Porto FG, EPE, Porto, 4200-072 Porto, Portugal; ^3^Centro Hospitalar do Porto, Porto, 4099-001 Porto, Portugal; ^4^Centro Hospitalar São João, Porto, 4200–319 Porto, Portugal

####### **Correspondence:** Ângelo Jesus (acj@estsp.ipp.pt) – Escola Superior de Tecnologia da Saúde, Instituto Politécnico do Porto, Vila Nova de Gaia, 4400-330 Vila Nova de Gaia, Portugal

**Background**

One of the most frequent symptoms in medical practice is cough. Consequently, there is a wide range of products available for its treatment, not only drugs but also some herbal products. This work focuses on the dry extract of Hedera helix, which increasingly gains popularity. Objective: Systematize the scientific evidence on the efficacy and safety of Hedera helix extract in the treatment of productive cough.

**Methods**

Systematic review, using databases Pubmed, EBSCO and Science Direct. Articles were selected in English, Spanish and Portuguese and with no more than twenty years since publication.

**Results**

Ten studies were selected, consisting of 69310 individuals with varying ages. One study was placebo controlled. Four studies compared different formulations with extract. One study analysed differences between the treatment with the dry extract of Hedera helix compared with the conventional therapy. Three open non-comparative multi-centre trials were also included as well as a retrospective study.

**Results**

All studies show evidence of the efficacy and tolerability of Hedera helix extract in the treatment of productive cough. Due to the minimal incidence of adverse effects, the authors were able to admit a good tolerability of ivy extract.

**Conclusions**

The studies included in this review indicate that Hedera helix dry extract preparations have positive effects in productive cough, showing improvement in terms of respiratory function in patients with diseases of the respiratory tract. However, more robust studies are required for a structured evaluation of the safety and tolerability of this extract in productive cough.

**Keywords**

Hedera helix, cough, ivy, alpha-hederin

#### P117 A portrait of the evaluation processes of education groups in primary health care

##### Viviane L. Vieira, Kellem R. Vincha, Ana Mª Cervato-Mancuso

###### Universidade de São Paulo, São Paulo, 05403-000, Brasil

####### **Correspondence:** Viviane L. Vieira (vivianevieira26@gmail.com) – Universidade de São Paulo, São Paulo, 05403-000, Brasil

**Background**

The development of educational actions in groups has been recommended by public health policies due the construction of knowledge and for the transformation of health care. However, the evaluation of the educational process in order to obtain a critical analysis of the group and its effects is a challenge for professionals. Objective: To analyse the perception of professionals – workers in Primary Health Care – about the evaluation processes of the groups linked with food and nutrition.

**Methods**

A qualitative research with 48 professionals was conducted in Sao Paulo, Brazil, (2012-2014) through semi-structured interviews. For the evaluation processes the Discourse of the Collective Subject was used and eight Central Ideas were identified.

**Results**

The presence of characteristics that promote the educational process was found in the groups, such as participation, links, empowerment and health autonomy. On the other hand, obstacles were found in the realization and systematization of the evaluation of the educational process of the groups, due to naturalization of quantitative variables (changes in weight and in biochemistry parameters), related to acquisition of knowledge and of individual approaches.

**Conclusions**

There is the need for further reflection on analysis criteria of coherent groups with health education assumptions in order to encourage professional development and qualification of care.

**Keywords**

education groups, primary health care, evaluation, food and nutrition education

#### P118 Benefits of vitamins C and E in sensorineural hearing loss: a review

##### Melissa Faria, Cláudia Reis

###### Escola Superior de Tecnologia da Saúde de Coimbra, São Martinho do Bispo, 3046-854 Coimbra, Portugal

####### **Correspondence:** Cláudia Reis (creis@estescoimbra.pt) – Escola Superior de Tecnologia da Saúde de Coimbra, São Martinho do Bispo, 3046-854 Coimbra, Portugal

Sensorineural hearing loss is a tendentiously growing problem and has been associated with toxic effects of free radicals such as reactive oxygen species (ROS) and nitric oxide (NO) in the auditory system. With this in mind, studies have shown that the administration of vitamins has positive effects and may decrease the sensorineural hearing loss.

In this sense, through a systematic review we intend to examine the scientific literature available about the effectiveness of the use of vitamins C and E in the improvement of the auditory system performance in individuals with sensorineural hearing loss.

Keywords were defined and a research was conducted of scientific articles in electronic databases and four studies were selected according to defined criteria and centred on the issue under study. The comparison of the results from the articles chosen reveals concrete evidence of a decrease in hearing thresholds provided by taking vitamins C and E, reaching a hearing gain of 30 dB.

**Keywords**

Vitamin C, vitamin E, hearing loss treatment, sensorineural hearing loss

#### P119 BODY SNAPSHOT – a web-integrated anthropometric evaluation system

##### Marco P. Cova^1^, Rita T. Ascenso^2,3^, Henrique A. Almeida^3^, Eunice G. Oliveira^3,4^

###### ^1^VOID – Software Development, 2415-767 Leiria, Portugal; ^2^Centre for Research in Informatics and Communications, Polytechnic Institute of Leiria, 2411-901 Leiria, Portugal; ^3^Escola Superior de Tecnologia e Gestão, Instituto Politécnico de Leiria, 2411-901 Leiria, Portugal; ^4^Instituto de Engenharia de Sistemas e Computadores de Coimbra, 3000 - 033 Coimbra, Portugal

####### **Correspondence:** Marco P. Cova (mc@void.pt) – VOID – Software Development, 2415-767 Leiria, Portugal

**Background**

Anthropometry refers to the characterisation of the human individual which involves the systematic measurement of the physical properties of the human body for the description of the individual’s body composition and morphology. Today, anthropometry plays an important role in industrial design, clothing design, ergonomics and architecture where statistical data about the distribution of body dimensions in the population are used to optimize products. Changes in lifestyles, nutrition, and ethnic composition of populations lead to changes in the distribution of body dimensions (e.g. the obesity epidemic), and require regular updating of anthropometric data collections. Objectives: This project aims to develop a web application with the intent of aiding and organizing the anthropometric study and follow up of patients regarding the evaluation of their body composition and morphology by professionals and researchers in several areas such as health, sports and body aesthetics.

**Methods**

This evaluation is performed using the calculation of various indexes: Body Mass Index, Conicity Index, Waist-to-Hip Ratio, Body Density, Fat Mass Percentage, Muscle Mass, Bone Mass, and also the calculation of the Somatotype, which is the most complete method to characterize individuals regarding their physical appearance, morphology and body composition.

**Results and conclusions**

The developed application is a fully anthropometric integrated system available on the web, allowing a global utilization worldwide. As a result, the continuous usage of the application will reflect on the amount of data available on the platform, increasing a valuable impact on future studies and research worldwide (http://bodysnapshot.dei.estg.ipleiria.pt).

**Keywords**

Anthropometrics, body morphology and composition, integrated evaluation system, user-friendly, Web application

#### P120 Anthropometric evaluation and variation during pregnancy

##### Miguel Santana^1^, Rafael Pereira^1^, Eunice G. Oliveira^1,2^, Henrique A. Almeida^1^, Rita T. Ascenso^1,3^

###### ^1^Escola Superior de Tecnologia e Gestão, Instituto Politécnico de Leiria, 2411-901 Leiria, Portugal; ^2^Instituto de Engenharia de Sistemas e Computadores de Coimbra, 3000 - 033 Coimbra, Portugal; ^3^Centre for Research in Informatics and Communications, Polytechnic Institute of Leiria, 2411-901 Leiria, Portugal

####### **Correspondence:** Rita T. Ascenso (rita.ascenso@ipleiria.pt) – Centre for Research in Informatics and Communications, Polytechnic Institute of Leiria, 2411-901 Leiria, Portugal

**Background**

Although pregnancy is one of the most common life events, surprisingly little scientific information is available in the literature when considering anthropometrics. Studies regarding the pregnant woman’s body size variation, namely for the design of clothes during gestation are quite common. But anthropometrics is a lot more than this: it is also a science that aids in characterizing body morphology and composition. Objectives: Currently, there are standard data of anthropometric parameters for specific groups such as children, seniors and athletes. However, specific values or data for pregnant women don’t seem to exist, nor any indication to whether or not it is possible to use the values from other data given. Therefore, the main objective of this study is to evaluate if the default data and the current anthropometric tools are appropriate to be used on pregnant women.

**Methods**

In this study, we analysed and evaluated the morphologic characterization and the body composition of seven pregnant women using existing anthropometric restricted profile methods. The tools considered in this study were: Body Mass Index, Somatotype, Conicity Index, Abdominal-Hip Ratio and Waist-Height Ratio.

**Results and conclusions**

Some tools do not demonstrate efficiency in the anthropometric evaluation such as Body Mass Index by placing the pregnant women within risk levels that do not correspond to reality. However, other methods are plausible to be used, such as Conicity, Index Abdominal-Hip and Waist-Height Ratios where the values obtained are within the expected range. In conclusion, the existing anthropometric tools need further study when characterizing pregnant women.

**Keywords**

Anthropometry, Pregnant, Somatotype, Anthropometric restricted profile, Body morphology and composition

#### P121 Knowledge of college students on the amendments of their eating habits and physical activity index in the transition to higher education

##### Rita Jesus^1^, Rodrigo Tapadas^1^, Carolina Tim-Tim^1^, Catarina Cezanne^1^, Matilde Lagoa^1^, Sara S. Dias^2^, Jorge Torgal^1^

###### ^1^Nova Medical School, Universidade Nova de Lisboa, 1169-056 Lisboa, Portugal; ^2^Centro de Estudos de Doenças Crónicas, Universidade Nova de Lisboa 1150-082 Lisboa, Portugal e Unidade de Investigação em Saúde, Escola Superior de Saúde de Leiria, 2411-901 Leiria, Portugal

####### **Correspondence:** Sara S. Dias (sara.dias@ipleiria.pt) – Centro de Estudos de Doenças Crónicas, Universidade Nova de Lisboa 1150-082 Lisboa, Portugal e Unidade de Investigação em Saúde, Escola Superior de Saúde de Leiria, 2411-901 Leiria, Portugal

**Background**

The transition period from high school to university is critical as the students suffer big changes in their life patterns, frequently adopting risk behaviour attitudes like unbalanced diets and physical inactivity that may have repercussions in their long term health. This research aims to study the perception of first year undergraduate students regarding their eating habits and physical activity indices in comparison with their habits at high school.

**Methods**

A cross sectional descriptive study of first year undergraduates (first enrolment) of Lisbon universities was performed. The information was gathered through on-line questionnaires. The data collected was analysed considering a 5.0 % significant level.

**Results**

From the final sample of 223 students from the Lisbon faculties 65.0 % (n = 145) were female and 35.0 % (n = 78) were male with ages between 18 and 21. The whole sample admits having decreased their indices of physical activity and 50.7 % has worsened their eating habits. 61.0 % of the sample is apparently satisfied with their current weight but wants to improve their eating habits and physical activity.

**Conclusions**

Living away from family is strictly related to stricter and harder to manage schedules, leading to worse food and exercise habits. Male students have more risk behaviours although they are the ones who most easily adopt behaviours considered healthy when it comes to physical activity. Health students are more conscious about their own health and are the most penalized by the lack of time. Prevention strategies undergo increasing awareness of these issues and the providing means to implement changes.

**Keywords**

University students, physical activity, food habits, perception, health

#### P122 Muscular activity of a rally race car driver

##### João Lopes^1^, Henrique Almeida^1^, Sandra Amado^2^, Luís Carrão^2^

###### ^1^Escola Superior de Tecnologia e Gestão, Instituto Politécnico de Leiria, 2411-901 Leiria, Portugal; ^2^School of Health Sciences, Polytechnic institute of Leiria, 2411-901 Leiria, Portugal

####### **Correspondence:** Luís Carrão (luis.carrao@ipleiria.pt) – School of Health Sciences, Polytechnic institute of Leiria, 2411-901 Leiria, Portugal

**Background**

Rally is a form of motorsport that takes place on public or private roads with specially built cars. It is distinguished by racing not on a circuit, but instead in a point-to-point format in which participants and their co-drivers drive between a set of control points (special stages), leaving at regular intervals from one or more start points. This type of sporting events has a huge physical load on the drivers where the levels of fatigue are very high. Objectives: The present project main’s purpose is to measure the muscular activation of a rally driver during a race. The study consists of evaluating the hand-force before and after a driving exercise and evaluating the muscular activity during driving.

**Methods**

Two methods were used to in order obtain the data. The first step consisted of obtaining the hand-force data with the use of a dynamometer, after which electromyography was used for evaluating the muscular activity during driving. The study was composed of 5 tasks, firstly the hand-force test, then a vertical vibration, lateral oscillation, a driving test and finally another hand-force test.

**Results and conclusions**

From the results, it is possible to conclude that the key muscles during driving activity are the anterior deltoid since this activity requires many rotations and movements of the upper limbs. This activity is most obvious during turns while driving. By undergoing this type of evaluation, it is possible to have a better understanding of the demands to which rally drivers are subjected.

**Keywords**

Electromyography, muscular activity, biomechanics, rally

### Therapeutic Education/Health literacy

#### O234 **Literacy and results in health**

##### Madalena Cunha^1^, Luís Saboga-Nunes^2^, Carlos Albuquerque^1^, Olivério Ribeiro^3^

###### ^1^Centro de estudos em educação, tecnologias e saúde, Escola Superior de Saúde, Instituto Politécnico de Viseu, 3504-510 Viseu, Portugal; ^2^Escola Nacional de Saúde Publica, Lisboa, 1600-560 Lisboa, Portugal; ^3^Escola Superior de Saúde, Instituto Politécnico de Viseu, 3504-510 Viseu, Portugal

####### **Correspondence:** Madalena Cunha (madalenacunhanunes@gmail.com) – Centro de estudos em educação, tecnologias e saúde, Escola Superior de Saúde, Instituto Politécnico de Viseu, 3504-510 Viseu, Portugal

**Background**

Health Literacy (HL), considered as a mediator of clinical results which can be associated with health promotion campaigns focusing on education, is a current focus for research on behavioural management of individual health. Differences in the levels of health literacy are associated with both a lesser capacity to interpret medication labels and health messages and also with worse health indicators. Objectives: To determine the correlation between HL and the Abdominal Perimeter (AP), the Circumferential Perimeter of the Neck (CPN), the blood glucose level and the Body Mass Index (BMI) in healthy individuals.

**Methods**

A study with observational focus on a sample of 508 people, with an average age of 44.48 years. Health literacy was assessed through the European Health Literacy Survey [1] and the AP, the CPN, the blood glucose level and the BMI were determined.

**Results**

Most participants (73.62 %) revealed a health literacy deficit. The risk of cardiovascular disease proved to be correlated with health literacy. Patients with inadequate literacy had a larger abdominal perimeter (AP χ2 = 101.65; p = 0.000), a larger neck perimeter (CPN χ2 = 10.34; p = 0.016), worse nutritional status/higher BMI (χ2 = 78.09; p = 0.000) and higher blood glucose levels. Contrarily, excellent literacy was linked to lower glucose levels (χ2 = 112.038; p = 0,000).

**Conclusions**

The priority is to consistently monitor the health variables previously mentioned and the significance of the evidence examined, determining the cost-effectiveness and the effects of policies and educational practices on clinical outcomes.

**References**

1. Saboga-Nunes L. Hermenêutica da Literacia em Saúde e sua avaliação em Portugal (HLS-EU-PT). in 40 anos de democracia(s): progressos, contradições e prospetivas. Atas do VIII Congresso Português de Sociologia. Lisboa: Associação Portuguesa de Sociologia; 2014.

**Keywords**

Literacy, results, health

#### O235 Literacy promotion and empowerment of type 2 diabetics elderly in four family health units of the group of health centers of Dão Lafões

##### Suzete Oliveira^1^, Mª Carminda Morais^2^

###### ^1^Escola Superior de Tecnologia e Saúde, Instituto Politécnico do Porto, 4400-330 Vila Nova de Gaia, Portugal; ^2^Escola Superior de Saúde, Instituto Politécnico de Viana do Castelo, 4900-347 Viana do Castelo, Portugal

####### **Correspondence:** Suzete Oliveira (suzt.oliveira@gmail.com) – Escola Superior de Tecnologia e Saúde, Instituto Politécnico do Porto, 4400-330 Vila Nova de Gaia, Portugal

**Background**

Type 2 Diabetes Mellitus (DM) is a serious public health problem that affects mostly elderly people. This disease has a huge human, social and economic cost, some of which could be prevented. Objective: The aim of this study was to analyse the capacity of self-control, the knowledge and the Quality of Life (QoL), of people with type 2 diabetes, aged 65 or older, enrolled in four Family Health Units, belonging to the Group of Health Centers of Dão Lafões.

**Methods**

An exploratory and descriptive-correlational study was applied to 137 sample subjects, that responded a sociodemographic and clinical characterization questionnaire and to the Portuguese version of the Diabetes Empowerment Scale - Short Form (DES-SF), Diabetes Knowledge Test (DKT) and of the EQ-5D.

**Results**

Results point to: high perception of capacity for self-management of DM; poor knowledge about the disease, with no statistical differences among diabetes non-insulin-treated individuals and insulin-treated; with mean ± SD scores of 3.52 ± 0.69 of DES-SF, 54.34 ± 17.72 of DKT and 0.63 ± 0.30 of EQ-5D. We detected, a positive and significant correlation between the ability to control DM, knowledge and the QoL. In addition, we also found some statistically significant differences in DES-SF, DKT and EQ-5D before educational attainment (t = -3.688, p < .001; t = -5.835, p < .001; t = -2.302, p < .05, respectively) and DM complications (t = -2.355, p < .05; t = -2.578, p < .05; t = -2.261, p < .05).

**Conclusions**

The evidence provided is crucial for a better adaption of the processes that promote health literacy and empowerment of people with diabetes.

**Keywords**

Empowerment, Knowledge, Quality of life, Individuals with type 2 diabetes

#### O236 Mediterranean diet, health and life quality among Portuguese children

##### Emília Martins^1^, Francisco Mendes^1^, Rosina Fernandes^1^, Cátia Magalhães^1^, Patrícia Araújo^2^

###### ^1^Centro de Estudos em Educação, Tecnologias e Saúde, Escola Superior de Educação de Viseu, 3504-501 Viseu, Portugal; ^2^Universidade Católica Portuguesa – Porto, 4202-401 Porto, Portugal

####### **Correspondence:** Emília Martins (emiliamartins@esev.ipv.pt) – Centro de Estudos em Educação, Tecnologias e Saúde, Escola Superior de Educação de Viseu, 3504-501 Viseu, Portugal

**Background**

The Chronic Diseases Global Action Plan 2013-2020, from WHO (2013) [1], denotes the relevance of urgent implementation of strategies promoting a healthy diet. The Mediterranean Diet (MD), recognized in 2013 by UNESCO as Humanity Immaterial Cultural Heritage, is increasingly considered in the literature as a fundamental eating pattern in the prevention of non-transmittable diseases and with a positive impact on life quality. Objectives: To understand the adhesion levels to MD in 230 Portuguese children with ages averaging 10.09 ± 1.32, and also analyse their relation to the perception of health state (very bad to very good) and life quality (including physical and psychological health dimensions, self-concept, and relationships with peers and family).

**Methods**

General characterization questionnaires, KIDMED and KINDL were used as instruments for data collection, with later SPSS analysis (IBM 23).

**Results**

Only 1.7 % revealed low adhesion levels to MD, in contrast to 34.8 % at an average level, and 62.6 % at a high level. We found statistically significant positive correlations (p < .05) between the adhesion to MD and the perception of health state (r = .29), physical health (r = .48), psychological health (r = .35), self-concept (r = .36), peer relationships (r = .33) and general life quality (r = .54).

**Conclusions**

Results reinforce the relevance of this eating pattern in childhood life quality, as well in health indicators. The implementation of promoting eating education strategies based on this heritage, also characteristic of Portuguese culture, is revealed as fundamental.

**References**

1. World Health Organization. Global action plan for the prevention and control of non communicable diseases. WHO: 2013.

**Keywords**

Mediterranean Diet, Health, Life quality, Children

#### O237 Health literacy, from data to action - translation, validation and application of the European Health Literacy Survey in Portugal (HLS-EU-PT)

##### Ana R. Pedro^1^, Odete Amaral^2^, Ana Escoval^1^

###### ^1^Escola Nacional de Saúde Pública, Lisboa, 1600-560 Lisboa, Portugal; ^2^Escola Superior de Saúde, Instituto Politécnico de Viseu, 3504-510 Viseu, Portugal

####### **Correspondence:** Ana R. Pedro (rita.pedro@ensp.unl.pt) – Escola Nacional de Saúde Pública, Lisboa, 1600-560 Lisboa, Portugal

In the last few years, several studies have shown that inadequate health literacy levels can have significant implications in health outcomes, in the use of health care services and consequently, in health costs. The concept of health literacy has changed from a purely cognitive definition to a definition that includes the personal and social components of the individual, assuming the ability to make informed decisions in their everyday life.

This analytical cross-sectional study aims to validate and use the European Health Literacy Survey (HLS-EU) for the Portuguese population (HLS-EU-PT).

The HLS-EU-PT has been applied throughout the country, including autonomous regions, by an academic network. Data collection was conducted by personal interview. The final sample was composed of 1,004 individuals aged ≥ 16 years.

The HLS-EU-PT is an adequate instrument to measure the health literacy levels of the Portuguese population and shows comparable psychometric properties to versions used in the other countries.

In Portugal, 61.0 % of the surveyed population has problematic or inadequate general health literacy level, considering that the average of the nine countries tested with HLS-EU is 49.2 %. Concerning the Healthcare dimension, only 44.2 % have a sufficient or excellent level of health literacy. In terms of disease prevention, about 45.0 % of respondents reveal a sufficient or excellent level of health literacy, and the average stands at 54.5 %.

In the Health Promotion dimension about 60.2 % of the auscultated population has problematic or inappropriate health literacy level, and the average stands at 52.1 %. Therefore, it is fundamental to implement a National Strategy for Health Literacy.

**Keywords**

Health Literacy, health education, validation studies, surveys

#### O238 Oral health literacy evaluation in a Portuguese military population

##### Victor Assunção^1^, Henrique Luís^2^, Luís Luís^3^

###### ^1^Escola Superior de Saúde, Instituto Politécnico de Portalegre, Portalegre, 7301-901 Portalegre, Portugal; ^2^Faculdade de Medicina Dentária, Universidade de Lisboa, Lisboa, 1649-003 Lisboa, Portugal; ^3^Escola Superior de Saúde, Instituto Politécnico de Leiria; 2411-901 Leiria, Portugal

####### **Correspondence:** Victor Assunção (victorassuncao@essp.pt) – Escola Superior de Saúde, Instituto Politécnico de Portalegre, Portalegre, 7301-901 Portalegre, Portugal

**Background**

Oral health literacy is crucial for the empowerment of patients. This literacy is fundamental in order for people to understand the procedures and the instructions provided by health professionals. It is also important to provide the individuals with skills to participate in the choice of treatments. In a community setting it is crucial to evaluate oral health literacy levels before the implementation of a health promotion programme, in order for it to become effective in the transmission of educational themes. Objectives: The main goal of this study is to evaluate the oral health literacy and oral health knowledge of students enlisted at a military police force school and military officials.

**Methods**

To achieve this goal, the Portuguese version of the Oral Health Literacy Instrument (OHLI-POR) was used with 274 enlisted students and 12 military officials.

**Results**

The percentage of students with adequate oral health literacy is 54 %; 38 % have marginal literacy and 7.3 % have inadequate literacy. In relation to the military officials, 58.3 % have adequate literacy, 33.3 % marginal and 8.4 % inadequate literacy. When analysing oral health knowledge on a scale from 0 to 100, students and military officials have a positive average of 63.81 and 75.98 respectively.

**Conclusions**

Oral health literacy belongs to a field of science in oral health that needs urgent intervention. The results from this study show that there is a need to develop educational strategies and work in order to increase the oral health literacy of this population.

**Keywords**

Oral health, oral health literacy, health literacy, military, health education

#### O239 Preferences to Internet-based cognitive behavioural therapy – do attachment orientations matter?

##### Jennifer Apolinário-Hagen, Viktor Vehreschild

###### Department for Health Psychology, FernUniversität in Hagen, 58084 Hagen, Germany

####### **Correspondence:** Jennifer Apolinário-Hagen (jennifer.apolinario-hagen@fernuni-hagen.de) – Department for Health Psychology, FernUniversität in Hagen, 58084 Hagen, Germany

**Background**

Attachment representations were found to be relatively stable predictors for health behaviour and related attitudes, e.g. adherence to psychotherapy, and the relationship to be mediated by perceived stress [1-2]. Nonetheless, the role of attachment in e-therapies is not well understood. Objective: The study aims to identify predictors for preferences to guided Internet-based CBT (ICBT) and to assess associations between preferences on ICBT, attachment orientations and stress.

**Methods**

A total of 436 healthy participants were provided with descriptions of three most common types of e-therapies to indicate their preference. The types varied in the involved guidance by a therapist (i.e. unguided versus guided ICBT versus videoconference setting). Attitudes towards ICBT were determined via a 17-item self-report measure. Furthermore, stress within the past four weeks was measured with the Perceived Stress Questionnaire (PSQ-20) [3]. To assess attachment orientations, a 9-item global version of the ECR–Relationship Structures (ECR-RS) [4] was used.

**Results**

Most respondents (39 %) preferred guided ICBT to unguided ICBT (20 %), videoconference psychotherapy (23 %) or none of them (18 %). Half of the respondents had experience with conventional psychotherapy, whereas 12 % were currently undergoing treatment and 7 % were seeking for help. Preferences corresponded to overall attitudes on ICBT. Regression analyses did not indicate that either attachment or stress was significantly predicting attitudes towards ICBT. Both, attachment anxiety and avoidance were positively correlated with perceived stress (medium effect sizes).

**Conclusions**

Most respondents preferred guided ICBT. Defining specific predictors for the utilization and acceptance of ICBT remains a challenge.

**References**

1. Vogel DL, Wei M. Adult attachment and help-seeking intent: the mediating roles of psychological distress and perceived social support. Journal of Counseling Psychology, 2005; 52(3):347-357.

2. Petronzi GJ, Masciale JN. Using personality traits and attachment styles to predict people’s preference of psychotherapeutic orientation. Counselling and Psychotherapy Research, 2015; 15(4):298–308.

3. Fliege H, Rose M, Arck P, Levenstein S, Klapp BF. Validierung des "Perceived Stress Questionnaire" (PSQ) an einer deutschen Stichprobe. Diagnostica, 2011; 47(3):142-152.

4. Fraley RC, Heffernan ME, Vicary AM, Brumbaugh CC. The Experiences in Close Relationships-Relationship Structures questionnaire: A method for assessing attachment orientations across relationships. Psychological Assessment, 2011; 23:615-625.

**Keywords**

Preferences, attitudes, e-therapy, e-mental health, ICBT, attachment orientations, perceived stress, mental health care, psychotherapy

#### O240 A comparative transnational study in health literacy between Austria and Portugal

##### Ulrike Fotschl^1^, Gerald Lirk^2^, Anabela C. Martins^3^, Isabel Andrade^3^, Fernando Mendes^3^

###### ^1^Department of Biomedical Sciences, University of Applied Sciences Salzburg, A-5412 Puch/Salzburg, Austria; ^2^Department of Medical and Bioinformatics, University of Applied Sciences Upper Austria, Hagenberg A-4232 Hagenberg, Austria; ^3^Escola Superior de Tecnologia da Saúde de Coimbra, São Martinho do Bispo, 3046-854 Coimbra, Portugal

####### **Correspondence:** Fernando Mendes (fjmendes@estescoimbra.pt) – Department of Biomedical Sciences, University of Applied Sciences Salzburg, A-5412 Puch/Salzburg, Austria

**Background**

Limited health literacy (HL) is associated with lower health outcomes, higher healthcare costs and lead to impaired student success. Objective. The study aimed at gaining knowledge about the English HL level of non-native students from Health Science studies, to assess their comprehension of medical related information.

**Methods**

An ERASMUS joint research project was conducted to compare the HL level of students from two non-native English speaking countries (N = 185): Austria, University of Applied Sciences, Salzburg (FHS) and Portugal, ESTeSC-Coimbra Health School (CHS). A validated HL assessment tool (Pfizer, The Newest Vital Sign) was used along with a questionnaire to find out if there are any correlations between lifestyle factors and HL.

**Results**

A total of 140 students completed the questionnaire. Reliability was acceptable with Cronbach’s alpha equal to 0.656. FHS and CHS students showed an HL average score of 5.18 and 3.45 (out of 6), respectively (T = 6.68; df = 111; p < 0.001). Better lifestyle habits were related with higher HL scores. There is an intermediate positive correlation between HL score and English education (ρ = 0.37, p < 0.001). Male students have a significant higher level of HL (5.09) (T = 2.1; df = 135; p = 0.04). Up to 86 % of students were capable of understanding/interpreting an information regarding health issues correctly, but only up to 50 % could resolve right all mathematic and analytical-logical tasks.

**Conclusions**

The questionnaire can be used as a reliable and additional as-assessment tool in application procedures for international student’s exchange programs (e.g. Erasmus) or European Master Degree programme in Biomedical Sciences.

**Keywords**

Health literacy, health science students, transnational

#### O241 Health literacy and social behaviours: relationship with sexually transmitted diseases?

##### Verónica Mendonça, Sandra Antunes, Isabel Andrade, Nádia Osório, Ana Valado, Armando Caseiro, António Gabriel, Anabela C. Martins, Fernando Mendes

###### Escola Superior de Tecnologia da Saúde de Coimbra, São Martinho do Bispo, 3046-854 Coimbra, Portugal

####### **Correspondence:** Fernando Mendes (fjmendes@estescoimbra.pt) – Escola Superior de Tecnologia da Saúde de Coimbra, São Martinho do Bispo, 3046-854 Coimbra, Portugal

**Background**

Health Literacy (HL) encodes the capacity to obtain, process and understand basic health information and act appropriately. An adequate level of HL can empower higher education students in the process of decision-making regarding their own health. Furthermore, social behaviours influence the increasing rate of Sexually Transmitted Diseases (STD) in this population. Objective. The aim of this study was to evaluate the relationship between HL, social behaviours and STD in higher education students attending the first year of health sciences (HSc) and non-health sciences (NHSc) courses.

**Methods**

Participants (N = 50 HSc; N = 33 NHSc) filled out a questionnaire to assess sexual history, satisfaction, self-esteem/efficacy; attitude/intention of condom use; control over sexual events; parental relationship; HIV heuristics; alcohol consumption; plus, the level of HL (Newest Vital Sign-PT). Additionally, a blood sample was collected for STD screening.

**Results**

HSc students tend to have a higher level of HL, use more condoms and consume less alcohol before a sexual encounter in comparison to NHSc students. The use of condom is reduced in the case of pill contraception and when the sexual partnership is stable. A prevalence of 2.4 % for HSV 2 was found in this population, along with an association between positive cases and lower HL level, less use of condoms and higher alcohol intake before a sexual encounter.

**Conclusions**

A trend was observed in this population of higher education students showing an association between higher level of HL, fewer social risk behaviours and a lower prevalence of STD.

**Keywords**

Health literacy, Sexually transmitted diseases, Social behaviours, Higher education students

#### O242 Parenting styles and attachment to parents: what relationships?

##### Paula A. Silva^1^, Lisete M. Mónico^2^, Pedro M. Parreira^3^, Carla Carvalho^2^

###### ^1^Universidad de Extremadura, 10003 Cáceres, España; ^2^Faculdade de Psicologia e Ciências da Educação, Universidade de Coimbra, 3001-802 Coimbra, Portugal; ^3^Escola Superior de Enfermagem de Coimbra, Coimbra, 3046-851 Coimbra, Portugal

####### **Correspondence:** Paula A. Silva (paula.almeida.silva0209@gmail.com) – Universidad de Extremadura, 10003 Cáceres, España

**Background**

Parenting and Attachment styles have implications for interpersonal relationships and adult psychopathology, being determinant for internal working models. Objectives: To explore the associations between parenting styles and attachment to parents.

**Methods**

Sample: 600 participants (M = 27.0; SD = 10.6 years-old, 68.7 % female). Measures: I) ECR-RS – The Experiences in Close Relationships – Relationships Structures Questionnaire to evaluate dysfunctional attachment (F1-Avoidance in attachment to mother and father; F2-Anxiety in attachment to mother and father); II) EMBU – the Egna Minnen av Barndoms Uppfostran self-report questionnaire for measuring one's memory of parental rearing experiences (F1-Emotional mother and father support, F2-mother and father Rejection, F3-mother and father overprotection). Both measures showed good psychometric properties.

**Results**

Regarding the mother, attachment avoidance (M = 2.69, SD = 1.14) was negatively correlated with emotional support (M = 2.98, SD = 0.63; r = -.55), rejection (M = 1.40, SD = 0.44; r = .41) and overprotection (M = 1.95, SD = 0.54; r = .14) parenting styles. Considering the father, attachment avoidance (M = 3.33, SD = 1.42) was negatively correlated with emotional support (M = 2.65, SD = 0.73), rejection (M = 1.31; SD = 0.46), and overprotection (M = 1.78; SD = 0.51) parenting styles. Anxiety in the attachment to the mother (M = 2.41, SD = 1.65) was negatively correlated with emotional support (r = -.15) and positively correlated with rejection (r = .26) and overprotection (r = .16). Anxiety in the attachment to the father (M = 2.46, SD = 1.67) was negatively correlated with emotional support (r = -.16) and positively correlated with rejection (r = .18) and overprotection (r = .20).

**Conclusions**

Negative parenting styles (overprotection and rejection) were positively associated with the attachment anxiety and avoidance types. Emotional support of the mother and father was negatively associated with the attachment anxiety and avoidance types.

**Keywords**

Parenting styles, attachment, attachment to parents, attachment styles, ECR-RS, EMBU

#### O243 Work-life balance in health professionals and professors: comparative study of workers with shift work and fixed schedule

##### Carla Carvalho^1^, Pedro M. Parreira^2^, Lisete M. Mónico^1^, Joana Ruivo^1^

###### ^1^Faculdade de Psicologia e Ciências da Educação, Universidade de Coimbra, 3001-802 Coimbra, Portugal; ^2^Escola Superior de Enfermagem de Coimbra, Coimbra, 3046-851 Coimbra, Portugal

####### **Correspondence:** Carla Carvalho (ccarvalho@fpce.uc.pt) – Faculdade de Psicologia e Ciências da Educação, Universidade de Coimbra, 3001-802 Coimbra, Portugal

**Background**

Shift work is usually mentioned in the literature as a factor of weight when it comes to the perception of conflict or facilitation in family and work management, especially in health professionals. Objective: To evaluate the extent to which working hours (fixed/shift) interfere with work-life balance.

**Methods**

We used T-F scale to evaluate the conflict and facilitation in work-family and family-work. The instrument was translated and adapted by Carvalho and Peralta (2009). It was applied to 96 health professionals (time shift) and 594 University Teachers (fixed schedule). The following were tested: the construct validity and reliability (subscales conflict, facilitation and consequences); if the three subscales have equivalent representations in the two samples; the effect of the schedule (fixed/variable) in the three dimensions.

**Results**

The subscales showed adequate psychometric properties (explained variance > 50 %; s > .40; .77 < α < .91), being invariant in the two samples (congruence coefficients from .936 to .976). Contrary to the hypothesis that shift work interferes negatively in work-life balance, schedule (fixed/shift) does not seem to influence the perception of interference (positive and/or negative) between family and work (Wilk's lambda = .99, p > .05; univariate F(6, 683) < 1:50, p > .05).

**Conclusions**

The results highlight the importance of continuing research, given the complexity of intervening variables in work-life balance and considering the samples in analysis. Given that there is life beyond work and because satisfied/happy people produce more, with higher quality, well-being and health, it is important to promote work-life balance.

**Keywords**

Work-life balance, Work-family conciliation, Work-family conflict, Work-family facilitation, Shift work

#### O244 Technology literacy in self-management of diabetes

##### Vânia Silva^1^, Paulino Sousa^1,2^, José M. Padilha^1^

###### ^1^Escola Superior de Enfermagem do Porto, 4200-072 Porto, Portugal; ^2^Center for Health Technology and Services Research, Faculty of Medicine, University of Porto, 4099-002 Porto, Portugal

####### **Correspondence:** José M. Padilha (miguelpadilha@esenf.pt) – Escola Superior de Enfermagem do Porto, 4200-072 Porto, Portugal

**Background**

Diabetes is a serious global public health problem with high mortality and morbidity. Living and controlling the disease implies developing self-management skills. However, little is known about the potential of use of ICT in this population. Objective: Describe and analyse the potential use of Information and communications technology (ICT) in the development of self-management skills.

**Methods**

A quantitative, exploratory, descriptive and correlational study, using a probabilistic stratified random sample of people enrolled in the National Diabetes Control Programme in Health Centres of a Local Health Unit. Data were collected with a form based on Davis and Venkatesh’s theoretical models, applied through telephone contact.

**Results**

The sample consists of 388 users, 53.4 % female, with a mean age of 65.7 years; 6.2 % of participants have no schooling, more than half of the participants (52.3 %) completed only the first cycle of basic education, and only 9.8 % completed higher education. People of higher age and less education are those with less technological literacy, limited access to information resources, as well as less use and a higher difficulty of use. They also present a lower intention of using ICT and make fewer demands of health information on the Internet. At the same time these participants showed more information needs and less health literacy. Already, younger participants and the more educated preferred the use of electronic resources as a source of health information.

**Conclusions**

The evaluation the potential user proves to be a highly useful tool for the development of informational resources available in line with technological literacy and appropriate to the informational needs of people.

**Keywords**

Information and Communication Technologies, Technology Literacy, Health Literacy, Diabetes

#### O245 Satisfaction with therapeutic education and its relationship with clinical variables in children with type 1 diabetes

##### Vera Ferraz^1^, Graça Aparício^2^, João Duarte^2^

###### ^1^Centro Hospitalar de Trás–os-Montes e Alto Douro, Vila Real, 5000-508 Vila Real, Portugal; ^2^Centro de estudos em educação, tecnologias e saúde, Escola Superior de Saúde, Instituto Politécnico de Viseu, 3504-510 Viseu, Portugal

####### **Correspondence:** Graça Aparício (gaparicio5@hotmail.com) – Centro de estudos em educação, tecnologias e saúde, Escola Superior de Saúde, Instituto Politécnico de Viseu, 3504-510 Viseu, Portugal

**Background**

Type 1 diabetes is a chronic illness that requires therapeutic education for empowering children/adolescents in managing their health-disease process. The relationship established and the level of satisfaction with the care provided sustains adherence to the treatment, improving metabolic control. Objectives: To analyse the level of satisfaction of children/youths with therapeutic education appointments and their relationship to the clinical variables.

**Methods**

Cross-sectional and descriptive-correlational study with a non-probabilistic sample of 135 children/adolescents between 8-18 years old (Mean: 13:45; SD = 2.83), with Type 1 diabetes. A survey comprising socio-demographic and clinical characterization was used, as well an adaptation of the “Survey on Patient Satisfaction with therapeutic education in Diabetes”.

**Results**

In the sample 54.1 % were boys, 36.3 % aged less than 12 years, the average of diagnosis was 4.15 years (SD = 46.19 months), ranging from 1 month to 15 years, and 83.0 % reported the mother as the main caregiver. For each subscale, higher rates of satisfaction were found in the "guidance" dimension (mean = 93.55 %; SD = 9.47), and less satisfaction in the "relationship/communication" dimension (Mean = 79.55 %; SD = 19.17). Relative to clinical variables, children/youths monitored in a central hospital, with fewer episodes of hypoglycaemia and less diagnostic time have higher satisfaction with therapeutic education (p < 0.05).

**Conclusions**

Identifying the level of satisfaction of children/youths with therapeutic education and the clinical situations that affect their satisfaction, can contribute to a better understanding of their expectations and needs, allowing adjustment of therapeutic education.

**Keywords**

Children/adolescents, Type 1 Diabetes, Therapeutic education, Satisfaction

#### O246 Nutrition-related knowledge in middle-age and older patients with type 2 diabetes

##### Carlos Vasconcelos^1,2^, António Almeida^2,3^, Joel Neves^2^, Telma Correia^2^, Helena Amorim^2^, Romeu Mendes^2,3,4^

###### ^1^Polytechnic Institute of Viseu, Viseu, 3504-510 Viseu, Portugal; ^2^University of Trás-os-Montes e Alto Douro, Vila Real, 5001-801 Vila Real, Portugal; ^3^Research Centre Sports Sciences, Health Sciences and Human Development, University of Trás-os-Montes e Alto Douro, Vila Real, 5001-801 Vila Real, Portugal; ^4^Unidade de Saúde Pública, ACES Douro I, Marão e Douro Norte, Administração Regional de Saúde do Norte, 5000-524 Vila Real, Portugal

####### **Correspondence:** Carlos Vasconcelos (romeuduartemendes@gmail.com) – University of Trás-os-Montes e Alto Douro, Vila Real, 5001-801 Vila Real, Portugal

**Background**

Understanding the impact of food choices on our health is a basic condition to have a healthy eating pattern – a cornerstone of type 2 diabetes treatment and control. Objectives: This study aimed to assess nutrition-related knowledge in middle-age and older patients with type 2 diabetes (T2D) and to analyse its determinants.

**Methods**

Nutrition-related knowledge was assessed using the Portuguese reduced version of the Nutritional Knowledge Questionnaire [1] in 46 individuals with T2D (29 women; 62.96 ± 7.15 years of age; literate [47.8 % to 4th grade; 28.3 % between the 5th-9th grade; and 23.9 % above the 9th grade]) candidates to *Diabetes em Movimento*®, a community-based lifestyle intervention programme developed in Vila Real, Portugal (NCT02631902). This version of the questionnaire consists of three sections (dietary recommendations [DR, 0-6 points]; sources of nutrients [SN, 0-34 points]; diet-disease relationship [DDR, 0-16 points]), totalling a maximum score of 56 points.

**Results**

Total score was 25.89 ± 7.21 points (58.7 % with a score lower than 28 points); DR 4.59 ± 1.11 points; SN 15.30 ± 5.37 points; and DDR 6.04 ± 2.22 points. No significant differences between genders were observed in the total score, nor any association with age. Significant differences were found between the less and the more educated individuals.

**Conclusions**

These patients present deficits on nutrition-related knowledge, particularly in SN and DDR fields. Gender and age does not appear to determine this knowledge, contrary to education level.

**References**

1. Parmenter K, Wardle J. Development of a general nutrition knowledge questionnaire for adults. Eur J Clin Nutr. 1999; 53:198-308.

**Keywords**

Nutrition-Related Knowledge, Health Literacy, Type 2 Diabetes

#### O247 Validating the HLS-EU-(PT) questionnaire to measure health literacy in adolescents (CrAdLiSa project: HLS-EU-PT)

##### Luís Saboga-Nunes^1^, Madalena Cunha^2^, Carlos Albuquerque^2^

###### ^1^Centro de Investigação em Saúde Pública, Escola Nacional de Saúde Publica, 1600-560 Lisboa, Portugal; ^2^Centro de estudos em educação, tecnologias e saúde, Escola Superior de Saúde, Instituto Politécnico de Viseu, 3504-510 Viseu, Portugal

####### **Correspondence:** Luís Saboga-Nunes (saboga@hotmail.com) – Centro de Investigação em Saúde Pública, Escola Nacional de Saúde Publica, 1600-560 Lisboa, Portugal

**Background**

Educators and the education settings can play a major role in promoting Health literacy (HL) as a direct outcome. New perspectives are being opened, such as the question of whether HL can be considered as a buffer variable at early ages (e.g. adolescence). The HLS-EU questionnaire has emphasized the dimensions of health care, health prevention and health promotion covering a wide range of perspectives in an integrated approach. Objective: The purpose of this research is to evaluate how health literacy can be measured in the age group of adolescents.

**Methods**

A quantitative and qualitative explanatory cross-correlated study based on a sample of 215 adolescents from the VFX region of Portugal was collected in a school setting. Measurement of adolescents’ HL (CrAdLiSa project) was implemented with the HLS-EU-PT survey, the Portuguese version of the European Health Literacy Survey instrument (www.literacia-saude.info).

**Results**

Reliability analysis of HLS-EU-PT dimensions show an internal consistence (Cronbach's alpha coefficient) of 0.946 (Health Care), 0.947 (Disease Prevention) and 0.958 (Health Promotion), while the global instrument presents a value of 0.98. Inadequate HL (4.2 %) and problematic HL (25.6 %) show that about 30.0 % of respondents have limited HL.

**Conclusions**

The results enhance the reliability, validity, internal validity, statistical validity longitudinal and linguistic validity, as land marks of the translation and validation process to Portuguese of the HLS-EU survey and applied to evaluate adolescents HL. Further research must investigate HL potential at this age range and how it should be developed in school curricula.

**Keywords**

health literacy, adolescents, HLS-EU-PT

#### O248 Health education in people with coronary heart disease: Experience of the cardiology department of a hospital on the outskirts of Lisbon

##### Elsa S. Pereira, Leonino S. Santos, Ana S. Reis, Helena R. Silva, João Rombo, Jorge C. Fernandes, Patrícia Fernandes

###### Hospital Prof. Doutor Fernando Fonseca E.P.E., Amadora, 2720-276 Amadora, Portugal

####### **Correspondence:** Elsa S. Pereira (elsa.m.silva@hff.min-saude.pt) – Hospital Prof. Doutor Fernando Fonseca E.P.E., Amadora, 2720-276 Amadora, Portugal

**Background**

This study takes place via a partnership between a Nursing School and the cardiology department of a hospital on the outskirts of Lisbon. Nursing interventions in the acute phase of Acute Coronary Syndrome (ACS) should be started early through a process of recognition of the need for behavioural change, health education and planning the treatment regimen. For the early development of a therapeutic intervention, the nurse acts by focusing the attention of the person on the need for management of a therapeutic regimen (self-care). The department has implemented an education programme. Knowledge of the impact of this intervention is needed for its continued development. Objectives: To characterize and evaluate the education strategy for health undertaken and to understand this intervention through the eyes of those to whom it is addressed.

**Methods**

An exploratory, analytical cross-sectional study of qualitative dimension, developed with the persons’ subject to the education sessions. Retrospective analysis of hospital processes with biopsychosocial characterization of individuals and identification of risk factors. Semi-structured telephone interviews were made one year after discharge.

**Results**

Modification occurred in identified risk factors, an evaluation of sessions in changing these factors was performed, and other educational support was identified.

**Conclusions**

Data was obtained on the impact of health education, given the knowledge of the disease, control of health risk factors and adherence to lifestyle and therapy. Contributions were collected for the reorganization of the sessions, and also for the development of a protocol to standardize the practice of education favouring improvement and guaranteed health gains.

**Keywords**

health education, coronary heart disease, nursing

#### O249 Information and training needs of informal caregivers of individuals with stroke sequelae: a qualitative survey

##### Jaime Ribeiro^1^, Catarina Mangas^2^, Ana Freire^3^

###### ^1^Escola Superior de Saúde de Leiria eCADR& Centro de Investigação Didática e Tecnologia na Formação de Formadores, Universidade de Aveiro, 3810-193 Aveiro, Portugal; ^2^Unidade de Investigação Inclusão e Acessibilidade em Acção “iACT” e Escola Superior Educação e Ciências Sociais, Instituto Politécnico de Leiria; 2411-901 Leiria, Portugal; ^3^Instituto Politécnico de Leiria, 2411-901 Leiria, Portugal

####### **Correspondence:** Jaime Ribeiro (jaime.ribeiro@ipleiria.pt) – Escola Superior de Saúde de Leiria eCADR& Centro de Investigação Didática e Tecnologia na Formação de Formadores, Universidade de Aveiro, 3810-193 Aveiro, Portugal

An adequate offer of information and training is undoubtedly a decisive step to endow the informal caregiver with the competences to safeguard the wellbeing of both the caregiver and the person to whom he/she provides care. There is a known insufficiency of information and training making it necessary to fill these lacks on those who take care of elderly people with stroke sequelae. The difficulty of finding an adjusted training is owed to the little knowledge of their real needs as well as to the fact that there isn’t a systematic plan addressed to these needs.

Focusing an area still to be explored in Portugal, this descriptive-exploratory study attempted to identify the information and training needs of informal caregivers of people with stroke sequelae, envisaging the design of a training programme. We endeavoured to conceptualize a training program starting from that which should be the starting point of any educative action - the identification of needs. Using a qualitative approach, we conducted an enquiry through a semi-structured interview with the informal caregivers, as well with key informants such as the formal caregivers and individuals with stroke sequelae, with subsequent content analysis according to Bardin.

It was observed in different dimensions the perceived importance of information and the need for a formal training structure. The results suggest the training must be geared towards supporting the process of rehabilitation and mediation, as well to the basic learning for the act of caring for a person with stroke sequelae, requiring a practical component.

**Keywords**

Informal caregivers, information and training needs, training programme

#### O250 Prevention of psychoactive substances consumption in students from 6th grade of Albergaria-a-Velha´s School Group

##### Sara Silva, Irene Francisco, Ana Oliveira

###### Unidade de Saúde Pública, Centro Hospitalar do Baixo Vouga, Aveiro, 3814-501 Aveiro, Portugal

####### **Correspondence:** Sara Silva (sararebelo.silva@gmail.com) – Unidade de Saúde Pública, Centro Hospitalar do Baixo Vouga, Aveiro, 3814-501 Aveiro, Portugal

**Background**

Teenagers are considered a vulnerable group for several risk behaviours, which include the use of psychoactive substances. The better you know the risk factors and protection, as well as the risks associated with consumption of these substances, the better you can define and identify intervention strategies.

Objectives: The overall aim of this intervention study was to empower students from the 6th grade, from the Albergaria-a-Velha School Group, in 2014, with personal and social skills for prevention of psychoactive substance consumption.

**Methods**

This study consisted of two groups, one subjected to intervention and one control group not submitted to intervention, each encompassing four classes. Five sessions were held, addressed to students on personal and social skills, in particular, about taking part in the decision and resolution of problems, of resistance to social influence and peer pressure and information. The evaluation is based on self-administered questionnaires before and after the intervention.

**Results**

In the intervention group there was an increase in the proportion of components of decision-making and problem solving and also in information, whereas in the resistance component of the social influence and peer pressure, there was a decrease. In the control group there was a rate of decrease for the three components.

**Conclusions**

After the completion of this work it is considered appropriate to continue this kind of intervention, not only to make use of the available material already applied/tested but also because more interventions on this issue are invaluable.

**Keywords**

Intervention, personal and social skills, psychoactive substances

#### O251 Promoting healthy sexuality: shared responsibility for family, youth and educators

##### Helena Catarino, Mª Anjos Dixe, Mª Clarisse Louro

###### Health Research Unit & School of Health Sciences, Polytechnic institute of Leiria, 2411-901 Leiria, Portugal

####### **Correspondence:** Helena Catarino (helena.catarino@ipleiria.pt) – Health Research Unit & School of Health Sciences, Polytechnic institute of Leiria, 2411-901 Leiria, Portugal

**Background**

The study aimed to identify the knowledge and information of young people and parents concerning sexuality and to identify the needs and difficulties in sexual education of youths.

**Methods**

This quantitative study was conducted on a non-probabilistic convenience sample which was composed by 285 young people aged between 14 and 20 years and their parents (257). A questionnaire comprising questions of open, closed and mixed responses was used.

**Results**

It was found that young people feel they have good information (57.4 %) on sexuality, have doubts about topics such as pleasure and orgasm (16.9 %) and sexually transmitted diseases (12.1 %) and that the issue continues to be a taboo on a family, school and social level. The information is acquired at school (24.9 %) and Internet (20.4 %). They talk with friends (39.1 %) about the subject, but do not talk to health professionals, or resort to family planning, and have risk behaviours. Most parents (73.3 %) had already talked to the child about the issue of sexuality, giving it much importance and considering that the issue should be addressed by 12 years of age. They feel less difficulty in talking to their children about boyfriends (57.6 %) and most difficulties in talking about their sexual behaviour (7.4 %). To overcome the difficulties, most parents resort to books (36.2 %) and Internet (29.6 %), considering the implementation a sexual education programme relevant (67.3 %).

**Conclusions**

The results show the importance of intervention for the prevention of risk behaviours and to promote healthy attitudes and sexual behaviour in young people.

**Keywords**

Sexual education, shared responsibility, youth, parents, educators

#### O252 Sexual risk behaviour in adolescents and young people

##### Saudade Lopes, Anjos Dixe

###### Health Research Unit & School of Health Sciences, Polytechnic institute of Leiria, 2411-901 Leiria, Portugal

####### **Correspondence:** Saudade Lopes (saudade.lopes@ipleiria.pt) – Health Research Unit & School of Health Sciences, Polytechnic institute of Leiria, 2411-901 Leiria, Portugal

**Background**

Young people have risky sexual behaviour for disease transmission. These increase with the early onset of sexual activity. According to the 3rd EU Health Programme 2014-2020 [1], it is necessary to identify and combat this. Objective: This study aimed to describe the sexual risk behaviour of young people by gender and age and its relationship with the age of onset of sexual activity.

**Methods**

This correlational study resulted from the application of a range of risky sexual behaviour, with four items in a sample of 128 adolescents and young people (12-24 years of age). They were 72 boys (M = 18.23 ± 2.99 years) and 56 girls (M = 17.93 ± 2.45 years). The scale was Likert-type with values from 1 to 5. The lowest values correspond to the worst behaviours.

**Results**

The results were grouped into four age groups (12-13; 14-16; 17-19 and 20 and over). The boys had worse behaviours (M = 3.82 ± 0.77) than girls (M = 4.38 ± 0.59) in the set of items and the total of age groups. This difference was statistically significant (p ≤ 0.01). The coefficient of Spearman showed no relationship between these behaviours and the age of onset of sexual activity. The boys report first sexual intercourse at 12 years old (M = 14.77 ± 1.49 years) and girls at 13 years old (M = 15.56 ± 1.36).

**Conclusions**

Young people are at risk from behaviours that differ by gender and age, but are unrelated to the age of onset of sexual activity.

**References**

1. Third health programme (2014-2020). Funding Health Initiatives. Brussels, Belgium: European Commission; 2013.

**Keywords**

Adolescents, young people, sexual behaviour

#### O253 Knowledge of school staff on type 1 diabetes

##### Mª Anjos Dixe^1^, Eva Menino^1^, Helena Catarino^1^, Fátima Soares^2^, Ana P. Oliveira^3^, Sara Gordo^1^, Teresa Kraus^1^

###### ^1^Health Research Unit & School of Health Sciences, Polytechnic institute of Leiria, 2411-901 Leiria, Portugal; ^2^Unidade de Saúde Pública - ACES Pinhal Litoral, ARS Centro, 3100-462 Pombal, Portugal; ^3^Consulta Externa de Pediatria, Centro Hospitalar de Leiria, 2410-197 Leiria, Portugal

####### **Correspondence:** Mª Anjos Dixe (maria.dixe@ipleiria.pt) – Health Research Unit & School of Health Sciences, Polytechnic institute of Leiria, 2411-901 Leiria, Portugal

**Background**

Considering the increasing incidence of type 1 diabetes and the fact most of these children attend school, school staff needs to be knowledgeable about providing a safe environment. Objectives: To evaluate the knowledge of school staff on type 1 diabetes and to relate it to the profession, years of professional practice in the institution and the perception about the level of preparation about type 1 diabetes.

**Methods**

A correlational study was conducted on 81 school staff elements, to whom a questionnaire was applied composed of socio-economic data, scale of knowledge about type 1 diabetes and level of perception about their preparation about it.

**Results**

Participants had an average knowledge of 11.1 ± 2.69 (maximum 17 points). Most of participants (63 %) were wrong on the issue "Cori has vomited. Your best initial response would be: call his parents", but most of them (95.1 %) hit the question "Today, Cory is requesting trips to the bathroom and fountain. You would: allow him to go to the bathroom and fountain”. Teachers hold a higher level of knowledge in relation to other professional groups (p < 0.01) and perceive a greater need for training and preparation (p < 0.01) about type 1 diabetes. The level of knowledge is not related to years of professional practice in the institution (p > 0.05).

**Conclusions**

The results show the importance of having prepared and knowledgeable school staff to follow these students. Thus, it is important to assess the level of knowledge and associated variables, in order to develop adjusted intervention programmes.

**Keywords**

Diabetes, school staff, nursing, knowledge

#### O254 Sexual health in adolescents: the impact of information search in literacy

##### Catarina Tomás^1^, Paulo Queirós^2^, Teresa Rodrigues^3^

###### ^1^Health Research Unit & School of Health Sciences, Polytechnic institute of Leiria, 2411-901 Leiria, Portugal; ^2^Escola Superior de Enfermagem de Coimbra, 3046-851 Coimbra, Portugal; ^3^Escola Superior Escola Superior de Enfermagem do Porto, 4200-072 Porto, Portugal

####### **Correspondence:** Catarina Tomás (catarina.tomas@ipleiria.pt) – Health Research Unit & School of Health Sciences, Polytechnic institute of Leiria, 2411-901 Leiria, Portugal

**Background**

Adolescents face numerous challenges searching for health information, and this search is essential to develop an adequate health literacy, that can enhance healthy choices. Objectives: The aim of this study is to analyse the impact of health information sources use and the search for sexual health information in sexual health literacy in a group of Portuguese adolescents.

**Methods**

To reach the aim it was developed a quantitative descriptive and correlational study, with a non-probabilistic sample of 1,215 adolescents that attend two secondary schools of the central region of the country. Data were collected with a questionnaire with sociodemographic questions, the Health Literacy Assessment Tool and the Portuguese version of the Health Literacy Questionnaire for Children.

**Results**

The sample was composed by female (47.08 %) and male (52.92 %) adolescents, aged between 14 and 20 years old (16.32; SD = 1.11), attending the three years of the secondary school (from 10th to 12th grade). Sexual health literacy was found to be good (0.65; SD = 0.18), being lower in males (p = 0.000) and in adolescents attending the 10th grade (p = 0.005). Sexual health literacy was found to correlate negatively with age (R = -0.105; p = 0.000). Results have shown that sexual health literacy is influenced by the use of health information sources, namely “doctor”, “television”, “pharmaceutics” and “leaflets” (R2 = 0.032; p = 0.000; Z = 9.087) and through receiving sexual health information (R2 = 0.045; p = 0.000; Z = 52.387).

**Conclusions**

There is an influence of the use of some health information sources and the increase of sexual health literacy. To increase this type of literacy it is important to increase the amount of sexual health information available, in order to improve its use.

**Keywords**

Sexuality, mental health, information resources

#### P123 Improving basic life support skills in adolescents through a training programme

##### Pedro Sousa, João G. Frade, Catarina Lobão

###### Health Research Unit & School of Health Sciences, Polytechnic institute of Leiria, 2411-901 Leiria, Portugal

####### **Correspondence:** Pedro Sousa (pedro.sousa@ipleiria.pt) – Health Research Unit & School of Health Sciences, Polytechnic institute of Leiria, 2411-901 Leiria, Portugal

**Background**

Basic life support (BLS) is the basis for saving lives following cardiac arrest. Previous studies support the need to train bystanders in BLS to reduce mortality and morbidity rates in situations of accident and sudden illness in extra-hospital settings. Objective: This study aimed to evaluate the impact of a training programme directed towards the BLS skills of adolescents.

**Methods**

This quasi-experimental study surveyed 176 adolescents from a Public Portuguese School aged between 11 and 17 years old, 55.1 % female and 44.9 % male. The intervention comprised a training programme directed towards the BLS skills of adolescents. The assessment instrument was applied before and after the intervention, including a 20-item questionnaire on BLS skills. The results were obtained using descriptive statistics and nonparametric tests. All ethical and formal procedures were respected.

**Results**

The BLS skills index before the intervention was 15.70 (SD = 2.02) (95 % CI: 15.35 to 16.06), out of a maximum of 20 points. At the end of the intervention the BLS skills index increased (p < 0.001) to 17.31 (SD = 4.58) (95 % CI: 16.93 to 17.68). Regarding the BLS skills determinants, we found a significant influence of parents’ qualifications (father: p = 0.037; χ2 = 8.456; mother: p = 0.001; χ2 = 16.131). Teens whose parents had higher qualifications presented increased skills in airway permeabilization.

**Conclusions**

The BLS training programme improved adolescents’ skills and knowledge. Special attention should be given to the determinants identified as influencing BLS skills in the design of future interventions and policies.

**Keywords**

Basic Life Support, skills, adolescents

#### P124 Difficulties in sexual education reported by basic education teachers in the city of Foz do Iguaçu - Brazil

##### Cynthia B. Moura, Laysa C. Dreyer, Vanize Meneghetti, Priscila P. Cabral

###### Universidade do Oeste do Paraná, Foz do Iguaçu, Paraná, 85851-100, Brasil

####### **Correspondence:** Cynthia B. Moura (cynthia-moura@hotmail.com) – Universidade do Oeste do Paraná, Foz do Iguaçu, Paraná, 85851-100, Brasil

Sexual education is a curricular component present in Brazilian schools, set by the National Curriculum Parameters (PCNs) drawn up by the Ministry of Education and Culture (MEC). However, unprepared teachers talking about this issue can lead to a simplified and just biological approach of sexuality.

The aim of this study was to conduct a survey about the 5 th grade teachers’ difficulties of public schools in Foz do Iguaçu - Paraná - Brazil, regarding sexual education. We chose to study this school grade since sexuality and human reproduction appear for the first time as a subject.

Participants were 82 teachers of 5th grade of fundamental education from 48 municipal schools of Foz do Iguaçu, Paraná, Brazil, representing 94 % of the total number schools in town and 60 % of the total of teachers of the grade. The data collection instrument was a form with questions about teachers’ difficulties, challenges of sexual education in schools and themes that should be addressed.

The difficulties reported by the teachers were mainly about talking about homosexuality, masturbation and sexual violence. The very permissive or neglecting families were pointed as the biggest challenges. The topics indicated as required in sexual education were: "teaching about the typical changes of puberty", "advising the risks and how to protect of abuse and paedophilia" and "informing about contraceptive methods and prevention of STDs/AIDS”.

The conclusion is that the data found in this survey can contribute to justify and better direct and train teachers in sexual education.

**Keywords**

Sexual education, sexuality, teenagers, teachers training

#### P125 Breast cancer survivors: subjects and resources for information. A qualitative systematic review

##### Francisca Pinto^1,2^, Paulino Sousa^3^, Mª Raquel Esteves^2^

###### ^1^Centro de Investigação Interdisciplinar em Saúde, Universidade Católica Portuguesa/Porto, 4202-401 Porto, Portugal; ^2^Instituto de Investigação e Formação Avançada em Ciências e Tecnologias da Saúde, Cooperativa De Ensino Superior Politécnico Universitário, Instituto Politécnico de Saúde do Norte, 4585-116 Gandra, Portugal; ^3^Escola Superior de Enfermagem do Porto, Porto, 4200-072 Porto, Portugal & Centre for Health Technology and Services Research, Faculdade de Medicina da Universidade do Porto, 4200-450 Porto, Portugal

####### **Correspondence:** Francisca Pinto (franciscampinto@gmail.com) – Centro de Investigação Interdisciplinar em Saúde, Universidade Católica Portuguesa/Porto, 4202-401 Porto, Portugal

**Background**

The increase in survival for breast cancer (BC), problems in transition to a post-treatment phase that BC women face and a decrease in communication between patients and healthcare system justifies exploring the literature on information needs and sources for information used by breast cancer survivors (BCS) in this phase of cancer experience. The objective is to describe information needs and information resources used by BCS after primary treatment that emerged from qualitative evidence.

**Methods**

A qualitative systematic review. Eight databases were systematically searched for studies using an expression resulting from a combination of terms (breast cancer, survivor, information, literacy). Original articles which focused on information needs and information resources used by BCS, based on their cancer experience, were included.

**Results**

Thirty studies were select. Most of them were exploratory and used focus group methodology to collect data. BCS need information that: I) helps them to be effective in the self-management of the disease (references to information about side effects of BC treatment; II) helps them to learn to live with cancer (more references about supportive resources). Health professionals, support groups and written materials are the information resources of first preference for BCS, despite using others. There is some evidence about the approach of BCS in using the web as an information resource (young women and those who live in rural areas).

**Conclusions**

The information needs of BCS after primary treatment are multiple and characterised by the experience of living with BC. BCS use different information resources. This evidence seems to be the core of needs in BC survival and justifies thorough research on these topics.

**Keywords**

Breast cancer survivor, information needs, information resources, systematic review

#### P126 Relationship between health literacy and prevalence of STI in Biomedical Laboratory Science students

##### Sofia Galvão^1^, Ite Tytgat^2^, Isabel Andrade^1^, Nádia Osório^1^, Ana Valado^1^, Armando Caseiro^1^, António Gabriel^1^, Anabela C. Martins^1^, Fernando Mendes^1^

###### ^1^Escola Superior de Tecnologia da Saúde de Coimbra, São Martinho do Bispo, 3046-854 Coimbra, Portugal; ^2^University Thomas More, B - 2800 Mechelen, Belgium

####### **Correspondence:** Fernando Mendes (fjmendes@estescoimbra.pt) – Escola Superior de Tecnologia da Saúde de Coimbra, São Martinho do Bispo, 3046-854 Coimbra, Portugal

**Background**

Despite the continuum development in technology that facilitates patient access to health information and services, the level of health literacy (HL) and electronic health literacy (eHL) influences individuals’ full participation in their own healthcare management. Although many campaigns have been implemented to raise awareness of young people to sexual risk behaviours, there is still an increasing number of cases of sexually transmitted diseases (STD) worldwide. Besides there is still a communication gap between health professionals and patients. Objective: The main goal of this joint research project is to study the relationship between HL, sexual behaviours and prevalence of STD in students of the Biomedical Laboratory Sciences, comparing students from Belgium, University of Thomas More (UTM) and Portugal, ESTeSC-Coimbra Health School (CHS).

**Methods**

The methodology includes the collection of a venous blood sample for the screening of STD and application of a validated HL assessment tool (Pfizer, The Newest Vital Sign) along with a questionnaire to find out if there are any correlations between sexual behaviours and HL.

**Results**

It is expected that higher education students frequenting health science studies will show an adequate level of HL and of risk behaviours, and a low prevalence of STD.

**Conclusions**

Depending on the results, this study could be a valuable asset to entities responsible for awareness campaigns on sexual risk behaviours, translating into a new approach for the prevention of STD.

**Keywords**

Health literacy, Sexual behaviours, Sexually transmitted diseases, Students

#### P127 Health literacy, risk behaviours and sexually transmitted diseases among blood donors

##### Mónica Casas-Novas^1^, Helena Bernardo^1^, Isabel Andrade^1^, Gracinda Sousa^2^, Ana P. Sousa^2^, Clara Rocha^1^, Pedro Belo^1^, Nádia Osório^1^, Ana Valado^1^, Armando Caseiro^1^, António Gabriel^1^, Anabela C. Martins^1^, Fernando Mendes^1^

###### ^1^Escola Superior de Tecnologia da Saúde de Coimbra, São Martinho do Bispo, 3046-854 Coimbra, Portugal; ^2^Instituto Português do Sangue e da Transplantação, Lisboa, 1000-208 Lisboa, Portugal

####### **Correspondence:** Fernando Mendes (fjmendes@estescoimbra.pt) – Escola Superior de Tecnologia da Saúde de Coimbra, São Martinho do Bispo, 3046-854 Coimbra, Portugal

**Background**

An adequate level of health literacy (HL) and electronic health literacy (eHL) is paramount to understand health risks and take action to change behaviours. Having in mind the prevalence of sexually transmitted diseases (STD) it is fundamental to evaluate the level of HL/eHL and its association with risk behaviours, especially in restricted groups of socio-cultural and clinical interest, such as the blood donors. For the blood donation at the IPST, a blood sample is taken for screening tests and at the same time an e-mail is sent to the blood donor. This e-mail contains the online form for donor eligibility and this provides a unique opportunity to associate a questionnaire for the assessment of HL/eHL. Objective. The aim of this study is to characterize the level of functional HL and eHL of blood donors.

**Methods**

A questionnaire addressing demographic data, level of knowledge on risk behaviours and STD, the Portuguese version of the scales Newest Vital Sign (NVS-PT) and e-HEALS (e-Health Literacy Scale) will be used in a representative sample of the nationwide population of blood donors.

**Results**

It is expected that blood donors raise their level of confidence and safety in the donation procedure in Portugal, calling more and new donors, increasing the blood supplies.

**Conclusions**

This innovative research project will be the foundation for a model for the IPST’ to develop effective evidence-based strategies to empower citizens for the blood donation.

**Keywords**

Health literacy, e-health literacy, risk behaviours, blood donors

#### P128 Promoting literacy in pregnancy health-care

##### Fátima Martins^1^, Montserrat Pulido-Fuentes^2^

###### ^1^Escola Superior de Enfermagem, Universidade do Minho, Braga, 4710-057 Braga, Portugal; ^2^Facultad de Terapia Ocupacional, Logopedia y Enfermería, Universidad de Castilla-La Mancha, 45600 Talavera de la Reina, Toledo, España

####### **Correspondence:** Fátima Martins (fmartins@ese.uminho.pt) – Escola Superior de Enfermagem, Universidade do Minho, Braga, 4710-057 Braga, Portugal

Adequate literacy in pregnancy is critical to providing the pregnant woman with the adoption of healthy lifestyles. In order to know the perspective of the midwife about health literacy in prenatal surveillance, we developed a descriptive exploratory study of a qualitative approach in which we conducted semi-structured interviews with 8 midwives who played roles in health centres in rural and urban areas.

The data were subjected to content analysis according to Bardin. "*To satisfy the information requirements*", "*to promote the well-being of the pregnant woman and child*", "*to educate towards motherhood*" and "*to promote family well-being*" are the purposes of Health Education.

The results demonstrated that literacy allows one to qualify the pregnant woman with knowledge, skills and attitudes in order to promote responsibility and autonomy. The "*level of education*" associated with the social environment were conditional factors for its promotion. It should be noted that the knowledge of the pregnant woman was transmitted by different agents, with a highlight on healthcare providers (n = 38), the family (n = 30) and information technologies (n = 26) (internet, television, books and magazines).

Women with a High School and Licentiate degree primarily seek new information technologies. Those who have a lesser degree of literacy have less knowledge about pregnancy and inadequate self-management of the problems that may arise. It is confirmed that the prenatal surveillance check-up is an adequate space to prepare the woman positively and comprehensively for pregnancy, childbirth and motherhood, as well as to transmit knowledge that will lead her to the performance of an informed citizenship.

**Keywords**

Health literacy, pregnancy, health promotion

#### P129 The lifestyles of the operating assistants of education

##### Isabel Barroso^1^, Gil Cabral^2^, M. João Monteiro^1^, Conceição Rainho^1^

###### ^1^Universidade de Trás-os-Montes e Alto Douro, Vila Real, 5001-801 Vila Real, Portugal; ^2^Centro Hospitalar de Trás-os-Montes e Alto Douro, Vila Real, 5000-508 Vila Real, Portugal

####### **Correspondence:** Isabel Barroso (imbarroso@utad.pt) – Universidade de Trás-os-Montes e Alto Douro, Vila Real, 5001-801 Vila Real, Portugal

**Background**

Operating Assistants (OA) who work in schools should embrace healthy lifestyles due to the active role they play within the educational community. Objective: To measure lifestyles of the Operating Assistants in secondary schools in the north of the Portugal.

**Methods**

A descriptive study was used as well as a questionnaire on the physical, psychological and social components of lifestyles, consisting of ten domains which are identified with the "FANTASTICO”. The Portuguese version, consisting of 30 items, each with three answer options, with values ranging from 0 to 2, and the total score of 0 to 120 points, categorized by: “*need to improve*” (0-46); “*regular*” (47-72); “*good”* (73-84); “*very good*” (85-102) and “*excellent lifestyle*” (103-120).

**Results**

Of the 49 OA participating in the study, most were females 35 (71.4 %), with the average age of 50.6 years old (SD = 6.6) having 19.1 years of work experience (SD = 7.6). The lifestyles of the Operational Assistants were classified as following: excellent 7 (14.3 %); very good 29 (59.1 %); good 11 (22.4 %) and a regular lifestyle 2 (4.1 %). The ten domains that presented the lowest scores were: tobacco use, physical activity and association commitments, family and friends. Tobacco consumption had the lowest score (mean = 6.61; SD = 2.49), which indicates the poorest lifestyle.

**Conclusions**

The characterization of the OA’s lifestyles will allow the planning of interventions in order to promote healthy lifestyles for the areas that presented the lowest scores.

**Keywords**

Lifestyles, Fantastic, Operating Assistants of education

#### P130 Experiences of service-learning health and the literary art: reflections about the health education

##### Alessandro Prado, Yara M. Carvalho

###### Universidade de São Paulo, São Paulo, 05403-000, Brasil.

####### **Correspondence:** Alessandro Prado (yaramc@usp.br) – Universidade de São Paulo, São Paulo, 05403-000, Brasil; Yara M. Carvalho – Universidade de São Paulo, São Paulo, 05403-000, Brasil

**Background**

The programme “Educação Pelo Trabalho para a Saúde (PET-Saúde”) at the University of Sao Paulo (USP), has provided for students in the health field that participated in the activities, different experiences of teaching-service integration that has contributed the training process in health as it responds to the principles of the Sistema Único de Saúde (SUS). Teamwork, multidisciplinarity and attention to the health needs of populations are the principles that guide the PET-Saúde, complementing curricular activities in basic attention/primary health care. Objective: To investigate the experiences of the integration of service learning in interventions of the PET-Saúde at USP-Capital.

**Methods**

We developed a case study research with interventions of the PET-Saúde, with conversation wheels and field diaries. For theoretical and conceptual design, we adopted José Saramago’s Blindness and the Philosophical Writing of Martinich, aiming to discuss and describe the experience.

**Results**

Service-learning integration happened with the PET-Saúde and contributed to broadening perspectives about the health-disease process and supplementing training in health in order to qualify the performance of the professional in the field, considering above all social determinants as inseparable components of health practices.

**Conclusions**

Diversity and plurality are ways of thinking and carrying out health training is critical to experience teaching strategies that instigate and produce more connection between students, users and services and more commitment in defence of life.

**Keywords**

Health Education, primary health care, health practices

#### P131 Life long swimming – a European Erasmus + project

##### Maria Campos, Liliana Moreira, José Ferreira, Ana Teixeira, Luís Rama

###### Faculdade de Ciências do Desporto e Educação Física, Universidade de Coimbra, 3040-248 Coimbra, Portugal

####### **Correspondence:** Maria Campos (mjcampos@fcdef.uc.pt) – Faculdade de Ciências do Desporto e Educação Física, Universidade de Coimbra, 3040-248 Coimbra, Portugal

Senior exercise is a valuable tool for people, because it leads to a long, healthy life, and for governments, because it could significantly reduce health expenditure. The goal of reducing health costs, promotes policies of active and healthy aging, aiming at controlling the increase in health care expenses predicted for the senior population. In this context, the emergence of social risk prevention policies, including the promotion of exercise, make sense. Exercising in water provides many benefits for senior citizens, and can be used to increase recovery from surgeries and other problems, to gain strength, stamina and balance for performance of daily activities. Therefore, Life Long Swimming (LLS) is a project sponsored and funded by Erasmus + European Union, and developed by the Italian, Spanish and Turkish Swimming Federations, under the scientific supervision of the Faculty of Sport Science and Physical Education, University of Coimbra (Portugal). The LLS Project aims to develop: I) an organization model: adapting the sport centres to the needs of seniors; II) a teaching model: a swimming educational programme for adults and seniors; III) a swimmers certification standard: The Swimmer’s book; IV) a clubs and institutional certification standard: the quality certificate LLS partner club.

The project is the result of an innovative cultural process to produce guidelines aiming to develop senior-friendly swimming centres, with special interest for active people over 60. The desired impacts are to spread awareness of the importance of health enhancing exercise through swimming and enthuse a sustainable number of new senior swimmers.

**Keywords**

Life Long Swimming project, seniors, swimming, European project

